# Pretargeted imaging beyond the blood–brain barrier†

**DOI:** 10.1039/d2md00360k

**Published:** 2022-12-02

**Authors:** Vladimir Shalgunov, Sara Lopes van den Broek, Ida Vang Andersen, Rocío García Vázquez, Nakul Ravi Raval, Mikael Palner, Yuki Mori, Gabriela Schäfer, Barbara Herrmann, Hannes Mikula, Natalie Beschorner, Maiken Nedergaard, Stina Syvänen, Matthias Barz, Gitte Moos Knudsen, Umberto Maria Battisti, Matthias Manfred Herth

**Affiliations:** a Department of Drug Design and Pharmacology, Faculty of Health and Medical Sciences, University of Copenhagen Universitetsparken 2 2100 Copenhagen Denmark umberto.battisti@sund.ku.dk matthias.herth@sund.ku.dk; b Neurobiology Research Unit and Center for Integrated Molecular Brain Imaging, Rigshospitalet Copenhagen University Hospital Blegdamsvej 9 DK-2100 Copenhagen Denmark; c Faculty of Health and Medical Sciences, University of Copenhagen 2200 Copenhagen Denmark; d Center for Translational Neuromedicine, University of Copenhagen Blegdamsvej 3B DK-2200 Copenhagen Denmark; e Leiden Academic Centre for Drug Research, Leiden University Einsteinweg 55 2333CC Leiden The Netherlands; f Institute of Applied Synthetic Chemistry, Technische Universitat Wien (TU Wien) Getreidemarkt 9 1060 Vienna Austria; g Rudbeck Laboratory, Department of Public Health and Caring Sciences, Uppsala University Dag Hammarskjölds Väg 20 75185 Uppsala Sweden; h Department of Dermatology, University Medical Center of the Johannes Gutenberg University Mainz Langenbeckstraße 1 55131 Mainz Germany; i Department of Clinical Medicine, University of Copenhagen Denmark; j Department of Clinical Physiology, Nuclear Medicine & PET, Rigshospitalet Copenhagen University Hospital Blegdamsvej 9 2100 Copenhagen Denmark

## Abstract

Pretargeting is a powerful nuclear imaging strategy to achieve enhanced imaging contrast for nanomedicines and reduce the radiation burden to healthy tissue. Pretargeting is based on bioorthogonal chemistry. The most attractive reaction for this purpose is currently the tetrazine ligation, which occurs between *trans*-cyclooctene (TCO) tags and tetrazines (Tzs). Pretargeted imaging beyond the blood–brain barrier (BBB) is challenging and has not been reported thus far. In this study, we developed Tz imaging agents that are capable of ligating *in vivo* to targets beyond the BBB. We chose to develop ^18^F-labeled Tzs as they can be applied to positron emission tomography (PET) – the most powerful molecular imaging technology. Fluorine-18 is an ideal radionuclide for PET due to its almost ideal decay properties. As a non-metal radionuclide, fluorine-18 also allows for development of Tzs with physicochemical properties enabling passive brain diffusion. To develop these imaging agents, we applied a rational drug design approach. This approach was based on estimated and experimentally determined parameters such as the BBB score, pretargeted autoradiography contrast, *in vivo* brain influx and washout as well as on peripheral metabolism profiles. From 18 initially developed structures, five Tzs were selected to be tested for their *in vivo* click performance. Whereas all selected structures clicked *in vivo* to TCO-polymer deposited into the brain, [^18^F]18 displayed the most favorable characteristics with respect to brain pretargeting. [^18^F]18 is our lead compound for future pretargeted neuroimaging studies based on BBB-penetrant monoclonal antibodies. Pretargeting beyond the BBB will allow us to image targets in the brain that are currently not imageable, such as soluble oligomers of neurodegeneration biomarker proteins. Imaging of such currently non-imageable targets will allow early diagnosis and personalized treatment monitoring. This in turn will accelerate drug development and greatly benefit patient care.

## Abstract 2

By screening a library of ^18^F-labeled tetrazines, we identified tetrazines which showed high pretargeted PET imaging contrast beyond the blood–brain-barrier.

## Graphical abstract

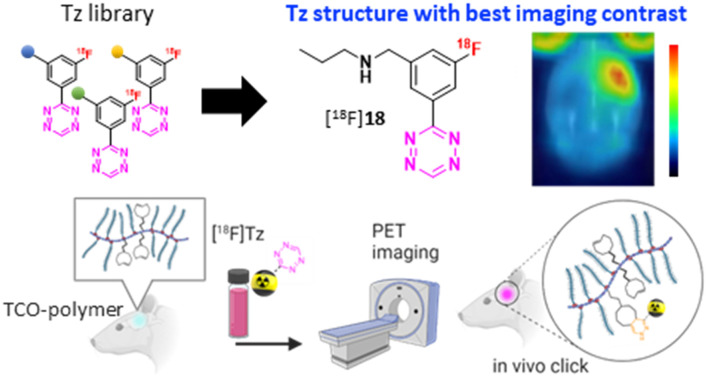

## Introduction

Positron emission tomography (PET) is a non-invasive molecular imaging method that relies on radiolabeled molecules (tracers) and is routinely used for clinical diagnosis, treatment monitoring and drug development.^1–4^ The key advantages of PET over other molecular imaging techniques are its quantitativity, high sensitivity, superior resolution and relatively low radiation dose for patients.^3–5^ Therefore, the development of new PET tracers is essential, especially for the emergence of new treatment forms.^6–8^ Monoclonal antibodies (mAbs) are particularly promising vectors for diagnostic imaging. This is because of their high target specificity and low non-displaceable binding.^9–12^ Recently, it became possible to use these vectors also for targets within the brain. Penetration of the blood–brain barrier (BBB) can be achieved by several methods such as active transport, for example by utilizing the transferrin receptor, or by opening the BBB, for example by using focused ultrasound-based (FUS) strategies.^13–18^ Despite these advances, the use of mAbs for diagnostic brain imaging is still in its infancy, as imaging is hindered by the slow pharmacokinetics of mAbs. Although there are long-lived radionuclides compatible with the long circulation time of mAbs,^19–22^ respective radionuclides provide inferior image quality and result in high radiation burden for the patient.^22^

**Fig. 1:**
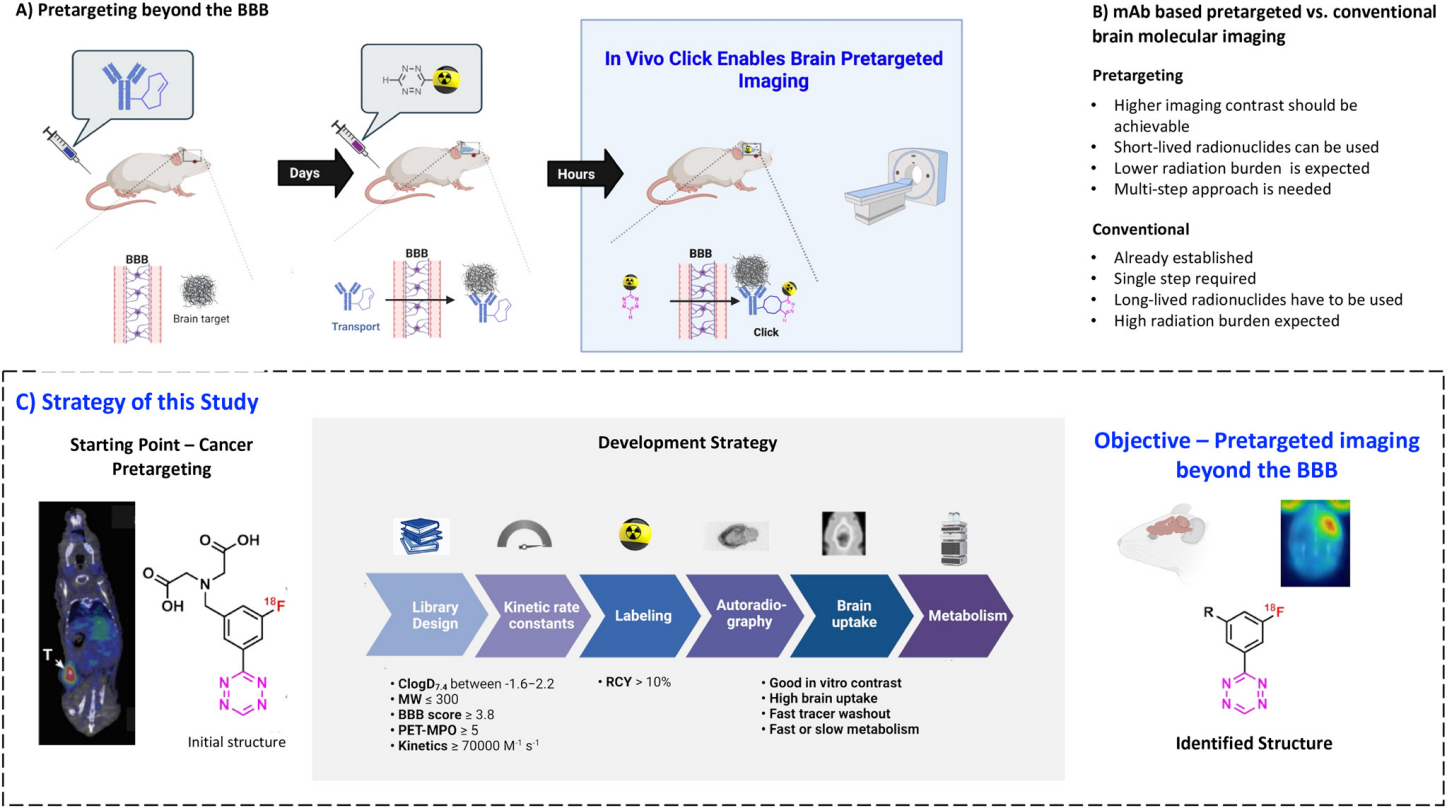
A) The concept of pretargeting beyond the BBB. In the first step, a BBB-penetrating TCO-tagged mAb is injected. The mAb is actively transported over the BBB and binds to its target. In the second step, a ^18^F-radiolabeled Tz is injected. The Tz clicks to the TCO-tagged mAbs, thus enabling the imaging of the selected target. B) Comparison between pretargeted and conventional imaging C) strategy and workflow of this study to develop BBB permeable Tz imaging agent that is able to click *in vivo* to targets within the brain. The starting point was a Tz probe developed for cancer pretargeting. A library was designed based on this scaffold to have optimal parameters to cross the BBB and click *in vivo*. All the designed molecules were synthesized to evaluate labeling feasibility and *in vitro* stability. The ^18^F-Tzs were then tested for imaging contrast with *in vitro* autoradiography. Finally, compounds were injected into rats to evaluate the brain uptake. Metabolism was then evaluated for the best compounds.

Pretargeting makes it possible to combine slow-circulating targeting vectors with short-lived PET radionuclides (Fig. 1A).^22–25^ In this approach, a tagged mAb is injected first and allowed sufficient time to accumulate at target-rich sites and to be eliminated from the blood. Next, a radiolabeled small molecule (effector molecule) is injected, which possesses fast pharmacokinetics and can rapidly and selectively react with the tag of the beforehand administered mAb.^25–27^ The use of *trans*-cyclooctene (TCO) derivatives as chemical tags and 1,2,4,5-tetrazine (Tz) derivatives as effector molecules has become state-of-the-art for pretargeted PET imaging due to the ultra-fast kinetics of the inverse electron demand Diels–Alder (IEDDA) “click” cycloaddition between these two structures, its bioorthogonality and compatibility with multiple scaffolds.^28–30^ Development of controlled drug delivery by means of Tz-triggered decaging of TCO-drug conjugates or *vice versa* (*click-to-release*) makes this chemistry even more attractive.^22,31–33^

Recently, we performed a systematic study of the relationship between the physicochemical properties of Tzs and their *in vivo* click performance in a colon tumor model. This study revealed that only hydrophilic Tzs with fast click kinetics were suitable for pretargeted tumor imaging.^34^ Hydrophilicity was crucial for fast clearance of unreacted Tzs from healthy tissues and excretion through kidneys and/or liver.^34^ Such Tzs are unsuitable for imaging of brain targets as their hydrophilicity prevents any reasonable BBB penetration. However, Tz uptake in peripheral organs is irrelevant for brain imaging contrast, because the brain is spatially separated from them. Therefore, we hypothesized that pretargeted imaging within the brain is possible with sufficient contrast using more lipophilic Tzs.

The aim of the present study was to explore the physicochemical parameters that influence the performance of Tzs for pretargeted brain imaging and apply this knowledge to develop a suitable imaging agent for this purpose (Fig. 1A). We decided to develop ^18^F-radiolabeled Tzs. First of all, fluorine-18 (^18^F) is widely considered to be the ideal PET radionuclide due to its convenient half-life (110 minutes), high positron branching ratio (97%) and short positron range in tissue (max. range 2.4 mm in H_2_O). These properties altogether result in high-resolution images with low patient radiation dose.^1,2^ Secondly, ^18^F can be produced on large scale in centralized facilities, and ^18^F-labeled radiopharmaceuticals consequently distributed for clinical use.^35^ Thirdly, introduction of ^18^F into the Tz scaffold can be used to fine-tune its reactivity, while the physiochemical properties of the fluorinated framework can be simultaneously manipulated with an extra handle (Fig. 1B).^36,37^

We have recently developed methods to prepare highly reactive Tzs labeled with fluorine-18.^37–40^ These methods were used to label all Tzs in this study. The respective development strategy is displayed in Fig. 1. In short, we designed and synthesized a panel of ^18^F-labeled Tzs guided by parameters reported to increase the chances to develop a successful tracer for brain imaging. Selection of the parameters was inspired by the BBB score – a parameter developed to identify brain-penetrating molecules.^41^ Subsequently, the Tz panel was subjected to *in vitro* and *in vivo* screening. Based on the results, a subset of Tzs was selected for final evaluation in a pretargeting model. In this model, rats were intracerebrally injected with a non-internalizing TCO-polymer. This polymer was then – in a second step – targeted by selected radiolabeled Tzs. We decided to use this invasive model as it allowed us to solely study the *in vivo* performance of the Tzs without challenges arising from a brain-targeting antibody, for example with respect to blood circulation, target engagement or metabolism. Obtained results were afterwards analyzed to identify possible relationships between the *in vivo* performance and physicochemical parameters of the Tzs. The aim of this work was to identify a Tz best suited for pretargeted brain imaging.

## Results and discussion

### Design of the tetrazine library

The development of brain imaging agents is often challenging. Good BBB permeability, acceptable non-displaceable binding and sufficient metabolic stability are only some of the criteria that must be met. Multiple trade-offs exist between parameters that influence these criteria. For example, high lipophilicity increases brain uptake of the tracer, but also its non-displaceable binding. Therefore, it is essential to balance these parameter values, *i.e.*, identify the value that positively affects one criterion without disrupting another.^42–46^ Nowadays, many physicochemical parameters can simply be estimated from the chemical structure.^42–46^ These estimations can be used to calculate a variety of composite scores.^47^ The CNS MPO and BBB scores, for example, are based on parameters such as MW, numbers of H-bond donors/acceptors, clog *D*_7.4_ or clog *P*. These scores can be used to estimate the probability of a certain structure to enter the brain and consequently be used as a brain tracer.^41,45,48,49^

**Fig. 2:**
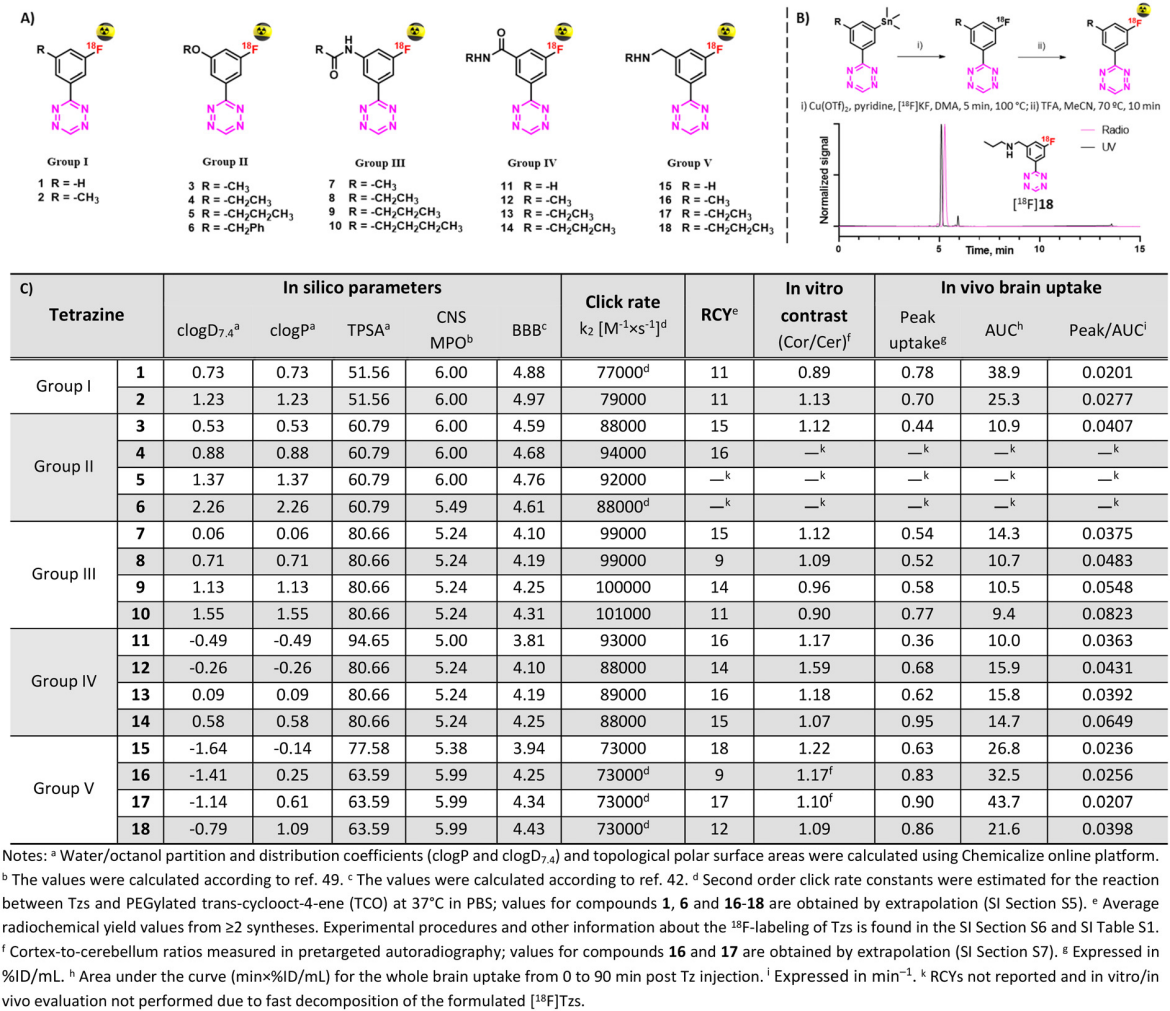
A) Designed Tz structures. B) Radiolabeling of Tzs [^18^F]1–18 and HPLC traces of [^18^F]18. C) *In silico* properties of Tzs along with key screening results.

In this work, we carefully designed a set of 18 Tzs, which were predicted to be suitable brain imaging agents. According to their CNS MPO and BBB scores, they possessed a probability of >75% to enter the brain in sufficient amounts (Fig. 2).^41,45^ All designed Tzs were based on the 3-fluorophenyltetrazine scaffold. This scaffold ensures fast click reaction kinetics, can be labeled with fluorine-18^37,38^ and modified with an extra handle to manipulate the physicochemical properties of Tzs.^37,39^ Consequently, it is an ideal starting point to develop BBB penetrant Tzs for pretargeted imaging. Since previous studies have shown that addition of a single methyl group can dramatically affect the pharmacokinetics and distribution of CNS tracers, a homologation approach was employed by stepwise increasing the length/bulk of the chosen side chain within five preselected Tz motifs (groups) (Fig. 2).^44,50,51^ Linkers within those motifs were designed based on the possibility to synthesize them easily and on similarities to endogenous structures known to enter the brain.

### Synthesis and characterization of the tetrazine library

Reference ^19^F-Tzs were synthesized *via* a Pinner-like synthesis from nitriles which were either commercially available or synthesized in-house.^52^ Click rate constants (*k*_2_) were determined with TCO by pseudo-first-order measurements in DPBS at 37 °C by stopped-flow spectrophotometry (ESI† Section S5).^38,53^ All tested Tzs displayed rate constant (*k*_2_) values above 70 000 M^−1^ × s^−1^ (Fig. 2). We have recently shown that such rate constants are strong indicators of high *in vivo* click performance of Tzs for pretargeting.^34^ Typical densities of brain targets are on the order of 100 nM,^54^ therefore the aforementioned rate constants will also be sufficient for pretargeted imaging beyond the BBB (ESI† Section S1).

### Radiolabeling

Stannane precursors were synthesized as described above ending with palladium-catalyzed stannylation (ESI† Section S4). Cu-mediated ^18^F-fluorination succeeded using our previously reported method (Fig. 2B, ESI† Section S6).^37^ If necessary, protecting groups were quantitatively removed in a second step by acid hydrolysis. After purification, all products were formulated in ethanol/phosphate buffer (0.1 M, pH 7.4). Obtained radiochemical yields (RCYs) were on the order of 9–19% within a synthesis time of approx. 90 min. Molar activities (*A*_m_) were on the order of 70–210 GBq μmol^−1^, radiochemical purities (RCP) >92%. Stability studies revealed that all compounds – except for [^18^F]5 and [^18^F]6 – were stable for at least 2 hours. Because of the low stability, possibly caused by radiolysis, [^18^F]5 and [^18^F]6 were not evaluated in further experiments.

### Pretargeted autoradiography

**Fig. 3:**
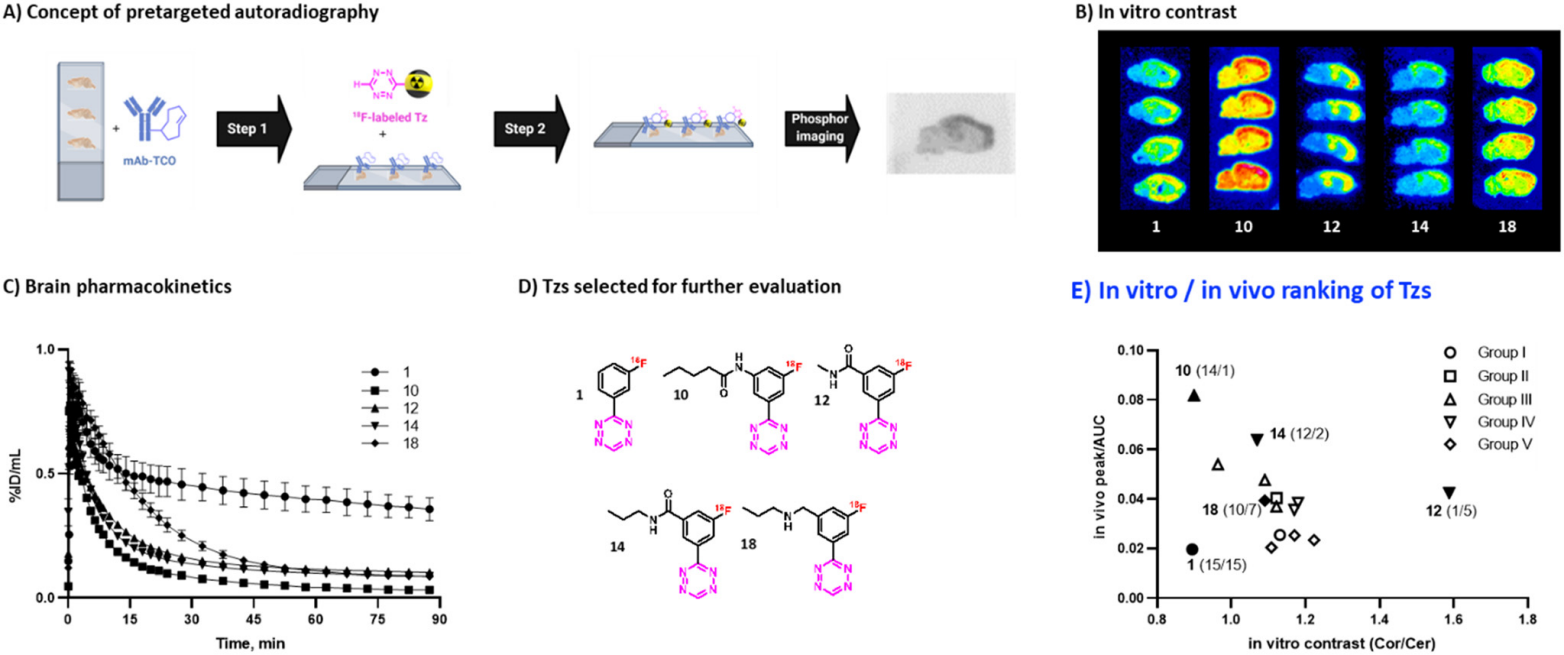
A) Workflow of pretargeted autoradiography. B) Pretargeted autoradiography images for selected Tzs. C) Brain time-activity curves for selected Tzs. D) Structures of Tzs selected for further evaluation. E) Ranking of evaluated Tzs by *in vitro* contrast and *in vivo* brain uptake kinetics. Tzs selected for further evaluation are marked with filled symbols, relative rankings (*in vitro*/*in vivo*) are shown in parentheses. *In vitro* contrast for [^18^F]18 was estimated by regression (see ESI† Section S7).

Autoradiography allows to identify radioligands with suitable imaging contrast for further *in vivo* studies.^55,56^ As Tzs do not possess a native target within the brain, direct autoradiography cannot be carried out. Instead, we developed an autoradiography protocol based on pretargeting (Fig. 3A, ESI† Section S7). In short, brain slices from Tg-ArcSwe mice – a mouse strain with high content of beta-amyloid (Aβ) fibrils in the cortex^57^ – were first incubated with TCO-modified 3D6 (anti-Aβ) mAbs. Excess of mAbs was then washed away. In a second step, these slices were incubated with ^18^F-Tzs. The Tzs clicked subsequently to Aβ-bound TCO-3D6. This strategy enabled us to visualize the binding of the mAb to Aβ, but, more importantly, it also allowed us to study the binding properties of applied ^18^F-labeled Tzs (^18^F-Tzs) with respect to their specific and non-specific binding. In order to mimic the *in vivo* situation as close as possible, so called “no-wash” autoradiographic experiments were carried out.^58^ The uptake values in cortex (Cor, Aβ-rich region) and in cerebellum (Cer, Aβ-poor region, assumed to represent non-specific binding) were determined. Cor/Cer ratios were used to rank ^18^F-Tzs – the higher the ratio the better the contrast (Fig. 2 and 3). [^18^F]12 possessed the best ratio, whereas [^18^F]1 the worst (Fig. 3B). Cor/Cer ratios inversely correlated with clog *P* (ESI† Fig. S4) and showed – as expected – that more lipophilic Tzs possess a higher non-displaceable component.

### Brain uptake

Hydrophilic Tzs result in the best *in vitro* contrast, whereas more lipophilic Tzs enter the brain better.^59^ To investigate this trade-off and identify a Tz that would provide an optimal combination of imaging contrast and brain uptake, we assessed the brain uptake and washout of all ^18^F-Tzs *in vivo* by dynamic PET scans in healthy Long–Evans rats (ESI† Section S8). Time activity curves (TACs) for the whole brain were expressed in % ID per mL tissue. We expected ^18^F-Tzs that show high peak brain uptake followed by fast washout to be best suited for pretargeted imaging. High initial uptake increases the possibility of the Tz to click to TCOs deposited in the brain, and fast washout decreases background levels. Consequently, Tzs with these properties should display high imaging contrast. We used the peak uptake as a measure of the brain uptake and the inverse area under the curve (1/AUC) as a measure for the washout. The uptake-washout index (equivalent to a ratio between peak uptake and AUC) was used to rank ^18^F-Tzs (Fig. 2). This value describes the interplay between high initial uptake and fast washout. The higher it is, the better the properties. Interestingly, peak/AUC ratios strongly correlated with clog *P* and clog *D*_7.4_ within the same Tz motif (ESI† Fig. S9). The most lipophilic Tzs from each class (2, 10, 14 and 18) displayed the best peak/AUC ratios.

### Candidate selection for pretargeted imaging beyond the BBB

In order to select Tzs for pretargeted imaging, we plotted the *in vitro* Cor/Cer ratios against the *in vivo* peak/AUC ratios (Fig. 3E). An ideal Tz with high ranks both *in vivo* and *in vitro* would be found in the top right corner of the resulting plot. Such a Tz was not identified. Consequently, we decided to test the three most promising Tzs, namely, [^18^F]12, [^18^F]10 and [^18^F]14. In order to verify that our ranking model can predict the performance of ^18^F-Tzs in pretargeted brain imaging, we also selected the lowest-ranking Tz [^18^F]1 and the middle-ranking Tz [^18^F]18 for testing. Thus, Tzs from 4 out of 5 Tz groups were tested in pretargeted imaging.

### Pretargeted imaging beyond the BBB

**Fig. 4:**
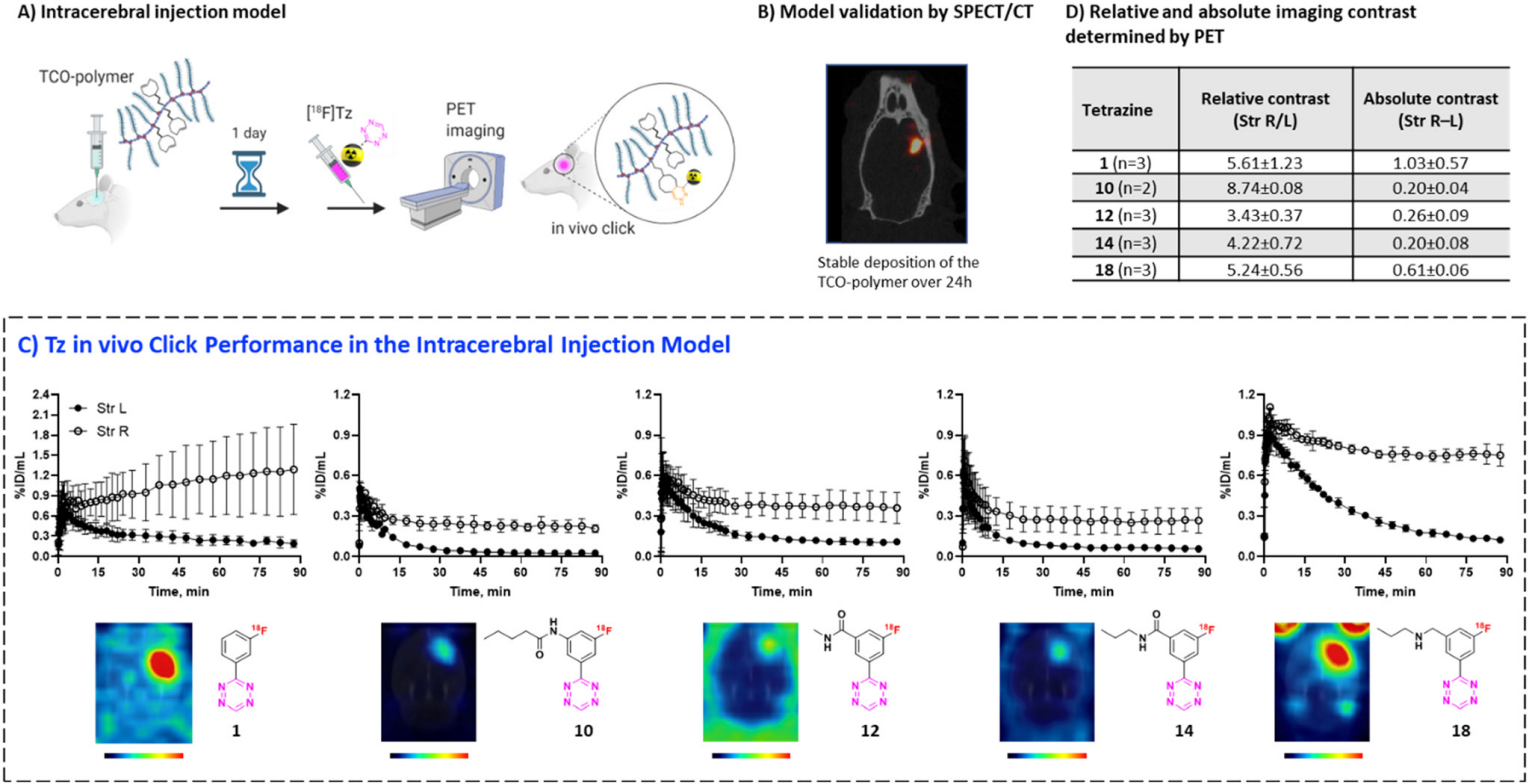
A) Intracerebral TCO-polymer injection model B) confirmation of TCO-polymer retention by SPECT/CT C) averaged TACs and representative PET images showing *in vivo* uptake of ^18^F-Tzs in the left (TCO-injected) and right (TCO-free) striata. D) Absolute and relative contrast between left and right striatum for selected Tzs. PET images and contrast measurements are based on average ^18^F-activity uptake at 60–90 min post-injection.

To evaluate the *in vivo* click performance of our Tzs, we used an invasive model based on intracerebral injection of a TCO-functionalized polymer (Fig. 4A). As mentioned beforehand, this model circumvents challenges that arise when administering a targeting vector systemically. For example, intracerebral injection does not create a large pool of blood circulating vector which reacts with administered Tz and prevents a significant fraction of it from reaching the brain.^60^ A detailed description of the model and its validation is reported in ESI† Section S9. In brief, Long–Evans rats were injected with TCO-decorated PeptoBrush (100 μg polymer, 15 nmol TCO in 4 μL of 10 mM phosphate buffered saline at pH 7.2) into the right striatum. This polymer has been shown to be non-internalizing, so challenges of extra diffusion barriers for Tzs and intracellular degradation of TCOs are not present.^61^ Moreover, in rodent plasma, which is similar in composition to interstitial fluid of the brain, at least 50% of the TCOs were stable for 24 h.^61^ Retention of PeptoBrush in the striatum was confirmed by SPECT/CT imaging using polymer batch labeled with indium-111 to high specific activity (290 MBq × mg^−1^, Fig. 4B). Restoration of the BBB integrity 18 h after polymer injection was confirmed by MRI imaging with the gadolinium-based contrast agent ProHance (ESI† Fig. S12).^62^ To quantify the retention of TCO-PeptoBrush at the injection site, the polymer was ^111^In-labeled to low specific activity (100–200 kBq × mg^−1^). 20–24 h after TCO-polymer injection, rats were injected with ^18^F-Tzs (10–30 MBq, 0.3–1.3 nmol) into the lateral tail vein and scanned in a PET scanner for 90 min. Radioactivity uptake in the right striatum was compared with the uptake in the left (polymer-free) striatum. After the scan, rats were sacrificed, their brains dissected into right and left hemispheres and gamma-counted 24 h later to measure the ^111^In-counts after the decay of ^18^F. On average, 49.4 ± 8.7% (*n* = 14) of the injected ^111^In-activity was found in the injected brain hemisphere, while only 0.5 ± 0.2% (*n* = 14) was found in the control hemisphere, indicating good and consistent retention of the TCO-polymer (ESI† Fig. S13). It is not known whether the observed loss of ^111^In-activity from the injection site reflects de-chelation of ^111^In or washout of the TCO-polymer. However, activity loss from the injection site was spread throughout the whole body. Therefore, local TCO concentration in the striatum was >1000-fold higher than elsewhere in the body and amounted to ≈92 μM assuming striatal volume of 40 μL per side,^63^ 50% polymer loss and 50% TCO isomerization after 24 h.

Preferential ^18^F-uptake in the TCO-polymer pre-injected striatum was clearly visible for all investigated Tzs, both on summed images as well as on registered TACs (Fig. 4C). We ranked ^18^F-Tzs in terms of their absolute and relative imaging contrast (Fig. 4D). Relative imaging contrast was defined as the ratio between ^18^F-uptake in the TCO-polymer injected *vs.* the polymer-free striatum, while the absolute contrast was defined as the difference between the uptake values, expressed in % ID per mL. The best relative contrast was observed for [^18^F]10, the best absolute contrast for [^18^F]1. These results are not expected from our prediction model displayed in Fig. 3E. For example, [^18^F]1 was suggested to be the least promising compound whereas good *in vivo* click performance was observed in pretargeted experiments. Although the absolute contrast for [^18^F]1 was high and appeared to be still growing at 90 min after Tz injection, the uptake in the polymer-injected striatum had a huge variation from scan to scan, which raises concerns for quantitative analysis and inter-subject comparison if [^18^F]1 is used for pretargeted imaging. Tz [^18^F]18 showed absolute and relative contrast comparable to [^18^F]1, but the respective values were much more robust. Therefore, we consider [^18^F]18 our prime candidate to be used in further studies.

### Metabolism

**Fig. 5:**
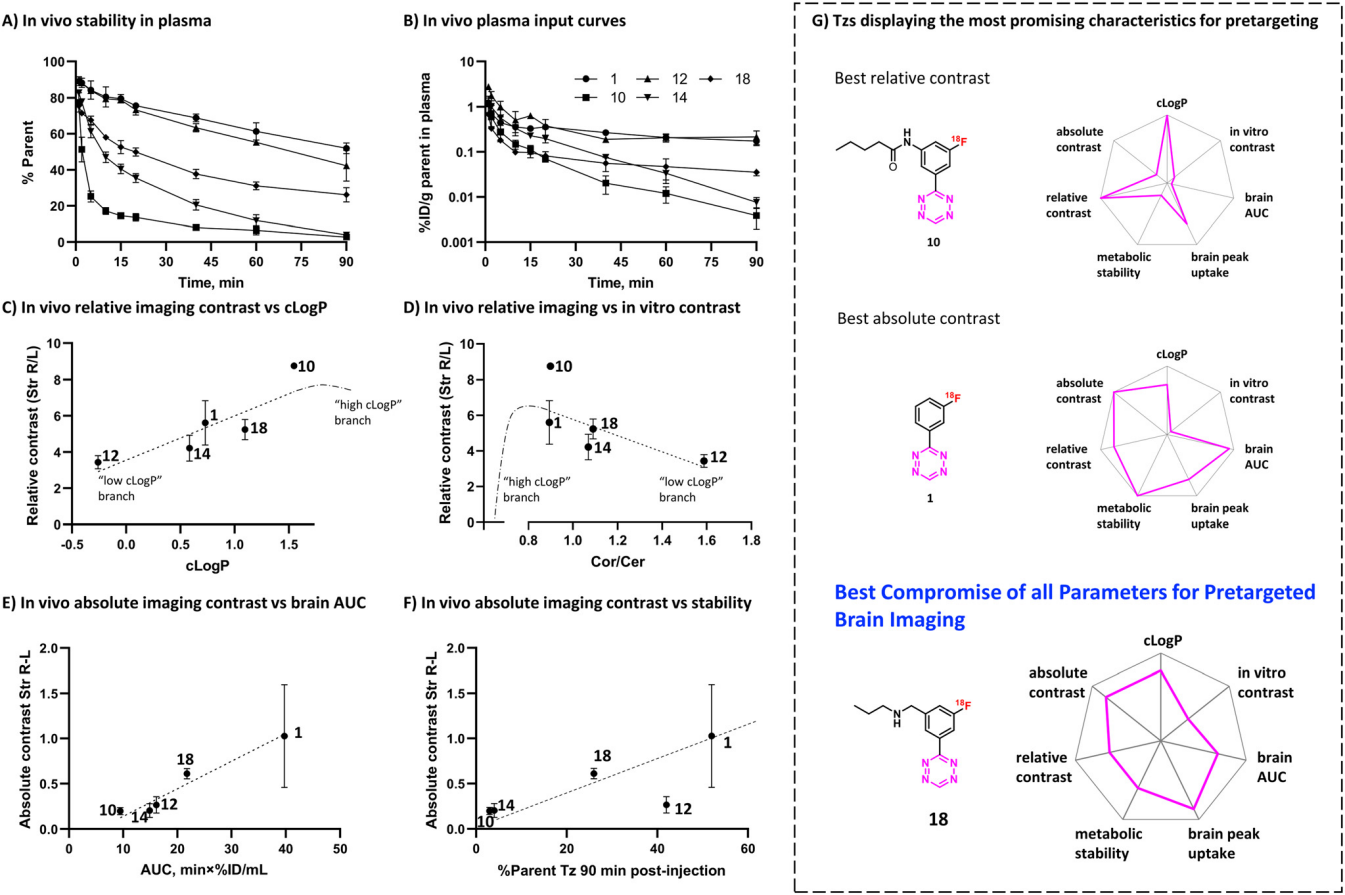
A) *In vivo* metabolic stability of selected Tzs in rat plasma B) metabolite-corrected arterial plasma input curves for selected Tzs. C and D) Correlation of relative imaging contrast with clog *P* and *in vitro* contrast, respectively. Dash-dot lines represent putative non-monotonous relationships for highly lipophilic Tzs, E) correlation of absolute imaging contrast with *in vivo* brain AUC, F) correlation of absolute imaging contrast with *in vivo* metabolic stability of ^18^F-Tzs. Dashed line represents putative trend without 12. G) Summary rankings of Tzs displaying the most promising characteristics for pretargeting beyond the BBB. Rankings are relative to other Tzs. [^18^F]18 was identified to possess the best parameters.

Metabolic stability is another important factor that influences the ability of Tzs to reach their targets in the brain and ligate to them *in vivo*.^34,42,64^ We decided therefore to determine the *in vivo* metabolism profile of our selected Tz tracers (Fig. 5A and B). Tzs [^18^F]14 and [^18^F]10 were the least stable: intact tracer comprised <5% plasma activity at 90 minutes post-injection (Fig. 5A). [^18^F]12 and [^18^F]1 showed the best stability (50% of intact tracer), while [^18^F]18 displayed an intermediate metabolism rate – approx. 30% intact tracer. All detected radiometabolites were much more hydrophilic than the parent (ESI† Fig. S15), indicating that these radiometabolites do not contribute to the brain activity as they are unlikely to pass the BBB. In general, rapid metabolism appeared to strongly influence the brain uptake of the studied Tzs, mainly due to less intact ^18^F-Tz being available to diffuse into the brain (Fig. 5B). Metabolic stability of Tz correlated with brain AUC (*r* 0.78, ESI† Fig. S16). The continued growth of [^18^F]1 uptake in the polymer-injected striatum 90 min after injection could be caused by continued influx of unmetabolized [^18^F]1 into that striatum, where it reacts with TCOs.

Both intact Tzs and their metabolites are eventually excreted through kidneys (renal clearance) or liver (hepatobiliary clearance). Examination of dynamic PET scans showed quick accumulation of radioactivity in the urinary bladder for all Tzs except [^18^F]1 (Fig. S10†). Presumably, bladder uptake represented mostly radiometabolites for [^18^F]14 and [^18^F]10 and mostly intact Tz for [^18^F]12. For [^18^F]18, radioactivity uptake in both bladder and liver increased rapidly, signifying greater preference for hepatobiliary clearance. [^18^F]1 showed slow clearance *via* both pathways. PET data for other Tzs confirmed the slow clearance of [^18^F]1 and showed that more hydrophilic tetrazines tended to have high radioactivity accumulation in the bladder, while for more lipophilic ones, radioactivity accumulated primarily in the liver and intestines (Fig. S11†).

### The *in vivo* click performance

In order to explain the observed *in vivo* click performance, we examined our data from *in silico* calculations, pretargeted autoradiography, *in vivo* brain uptake and metabolism studies. Relative *in vivo* imaging contrast showed positive correlation with clog *P* (*r* 0.88, Fig. 5C), but negative correlation with *in vitro* contrast from pretargeted autoradiography experiments (Cor/Cer ratio, *r* −0.67, Fig. 5D). This finding appears contradictory: the lower the *in vitro* contrast, the higher the *in vivo* contrast. We propose a speculative explanation for this finding, which implies the existence of a threshold clog *P* value, at which the trends for both *in vitro* and *in vivo* contrast should have a turning point (see ESI† Section S1). If the clog *P* values of the tested Tzs had been on both sides from this threshold value, we would have observed non-monotonous trends in both Fig. 5C and D. Independently of that, *in vitro* Cor/Cer ratios could not be used as an intuitive predictor of *in vivo* click performance.

Our initial assumption that *in vivo* click performance would positively correlate with the brain uptake-washout index of the Tzs – as determined by the peak/AUC ratios – was not confirmed (Fig. S14A and B†). However, AUC values (representing cumulative brain uptake) showed strong positive correlation with absolute imaging contrast (*r* 0.99, Fig. 5E). Absolute imaging contrast also strongly correlated with the BBB and CNS MPO scores, both of which are predictors of brain uptake (*r* 0.91–0.93, Fig. S14D and F†). Interestingly, absolute imaging contrast appeared to correlate with the metabolic stability for all ^18^F-Tzs except for [^18^F]12 (Fig. 5F). Rapid metabolism should result in less tracer being available to enter the brain from the blood, which can explain the low absolute imaging contrast of [^18^F]14 and [^18^F]10. Both tracers were rapidly metabolized (Fig. 5A and B). In contrast, [^18^F]18 and [^18^F]1 displayed slower metabolism and consequently, higher absolute imaging contrast (Fig. 5A, B and F). However, absolute imaging contrast of [^18^F]12 was not aligned with this trend. Its slow metabolism did not result in a high absolute imaging contrast (Fig. 5F). Apparently, the relatively low brain penetration of [^18^F]12, visible from its AUC (Fig. 5E) and possibly stemming from its low clog *P* (Fig. 2), turned out to be the determining factor for its click performance.

Our results indicate that fast Tz washout is not necessarily required for successful pretargeted brain imaging (Fig. 3E and 4C). Conversely, high cumulative brain uptake and slow metabolism seem important for good imaging contrast, as more Tz can enter the brain and click to TCOs (Fig. 5E and F).

## Conclusion

We investigated structure–activity relationships of a library of 18 different ^18^F-Tzs, their *in vitro* imaging contrast and *in vivo* brain pharmacokinetics to predict their *in vivo* click performance on targets beyond the BBB. Although we did not identify any single decisive parameter with a clear-cut relationship to pretargeted imaging contrast, slow metabolism as well as high lipophilicity appear to be beneficial for high brain uptake. Of the evaluated Tzs, [^18^F]18 showed the best imaging properties. High brain uptake combined with intermediate metabolic stability and good imaging contrast are the key properties of this tracer to be our prime candidate for further pretargeted imaging studies beyond the BBB (Fig. 5G). In the next step, we will design BBB penetrating and TCO-modified mAbs that can be targeted and imaged with [^18^F]18. TCO-mAbs with TCO isomerization half-lives over 4 days are already known, which allows long experiments.^65^ We believe that this approach ultimately will enable pretargeted imaging of brain targets, such as pathological protein isoforms and oligomers. These proteins are valuable drug targets for several neurodegenerative diseases and can currently not be imaged. Imaging would make it possible to diagnose these diseases, distinguish responders from non-responders or to monitor treatment. Consequently, imaging will provide valuable information to accelerate drug development and greatly benefit patient care.

## Ethical statement

All animal procedures were performed in accordance with the European Commission's Directive 2010/63/EU for animal research and approved by the Danish Council for Animal Ethics (license numbers 2017-15-0201-01283 and 2017-15-0201-01375) together with the Department of Experimental Medicine, University of Copenhagen.

## Author contributions

VS and SLB contributed equally. MMH and UMB conceived the study. Tz structures were designed by UMB. Organic syntheses were performed by UMB and RGV. Kinetic measurements were determined by BH and HM. Radiosyntheses were carried by RGV and VS. The pretargeted autoradiography protocol was designed by VS and SLB. *In vitro* screening of [^18^F]Tzs was conducted by SLB. *In vivo* PET experiments in rats were performed by IVA, NRR, NB and VS. Gadolinium MRI experiments were carried out by NRR, MP and YM. PeptoBrushes were designed by MB and synthesized by GS. *In vivo* and *ex vivo* data was analyzed by VS, SLB, IVA, SS, MN and GMK. Radiometabolite experiments were performed by IVA and VS. Manuscript was drafted by VS, UMB, RGV, NRR and SLB. VS, UMB and MMH edited the manuscript with critical feedback from all authors. All authors read and approved the final manuscript. MMH and UMB are the corresponding authors.

## Conflicts of interest

There are no conflicts to declare.

## Supplementary Material

MD-014-D2MD00360K-s001
