# Supplementary material for: Pretargeted imaging beyond the blood–brain barrier
Source: RSC Med Chem. 2022 Dec 2;14(3):444–53. doi: 10.1039/d2md00360k (PMC10034008; doi:10.1039/d2md00360k)
Supplement: MD-014-D2MD00360K-s001 [file MD-014-D2MD00360K-s001.pdf]

Electronic Supplementary Material (ESI) for RSC Medicinal Chemistry.
This journal is © The Royal Society of Chemistry 2022

Pretargeted Imaging beyond the Blood-Brain Barrier

Vladimir Shalgunov[a,*], Sara Lopes van den Broek[a,*], Ida Vang Andersen[a], Rocío García Vázquez[a], Nakul Ravi Raval[b], Mikael Palner[b,c], Yuki Mori[c], Gabriela Schäfer[d], Barbara Herrmann[e], Hannes Mikula[e], Natalie Beschorner[c], Maiken Nedergaard[c], Stina Syvänen[f], Matthias Barz[d], Gitte Moss Knudsen[b,g], Umberto Maria Battisti[a,#], Matthias Manfred Herth[a,h,#]

[a] Department of Drug Design and Pharmacology, Faculty of Health and Medical Sciences, University of Copenhagen, Universitetsparken 2, 2100 Copenhagen, Denmark; [b] Neurobiology Research Unit and Center for Integrated Molecular Brain Imaging, Rigshospitalet Copenhagen University Hospital, Blegdamsvej 9, DK-2100 Copenhagen, Denmark; [c] Center for Translational Neuromedicine, University of Copenhagen, Blegdamsvej 3B, DK-2200 Copenhagen, Denmark; [d] Leiden Academic Centre for Drug Research, Leiden University, Einsteinweg 55, 2333CC Leiden, the Netherlands; [e] Institute of Applied Synthetic Chemistry, Technische Universitat Wien (TU Wien), Getreidemarkt 9, 1060 Vienna, Austria, [f] Rudbeck Laboratory, Department of Public Health and Caring Sciences, Uppsala University, Dag Hammarskjölds väg 20, 75185 Uppsala, Sweden; [g] Department of Clinical Medicine, university of Copenhagen, Denmark; [h] Department of Clinical Physiology, Nuclear Medicine & PET, Rigshospitalet Copenhagen University Hospital, Blegdamsvej 9, 2100 Copenhagen, Denmark.

[*] Authors have contributed equally

[#] Corresponding authors

Supporting information

Section S1: Supplementary Discussion 2

Section S2: General Procedures 5

Section S3: Synthetic Schemes 7

Section S4: Organic Synthesis 9

Section S5: Estimation of click rate constant for ^{19}F-Tzs 54

Section S6: Radiochemistry 55

Section S7: Pretargeted autoradiography 62

Section S8: In vivo PET 67

Section S9: Intracerebral TCO-polymer injection (ic-TCO) model 76

Section S10: In vivo metabolism of ^{18}F-Tzs in healthy rats 82

Section S11. Statistical analysis 85

Section S12: Analytical Radio-HPLC and semi-preparative HPLC chromatograms of formulated [^{18}F]Tzs 86

Section S13. ^{1}H, ^{13}C and ^{19}F NMR spectra 105

References 188

Section S1: Supplementary Discussion

Implications of k_2 click rate constant values for pretargeted imaging contrast

The click reaction of Tz and TCO is irreversible, so pretargeted imaging with Tz and TCOs is comparable to imaging with irreversibly binding receptor tracers. The key parameter characterizing the imaging contrast for such tracers is the trapping rate (k_3), which is equivalent to the product of target site density (Bm) and receptor-tracer association rate (k_{on}), provided that tracer binding to proteins in the interstitial fluid can be disregarded[1].

Typical brain target concentrations are in the order of 100 nM.[2] For a k_2 of 70 000 $M^{-1}\times s^{-1}$ (4.2 mL/(nmol×min) and local TCO concentration (Bm_{TCO}) of 100 nM (0.1 nmol/mL), the trapping rate (k_3) will be equal to:

$$k_3 = k_2 \times Bm_{TCO} = 4.2 \times 0.1 = 0.42 \text{ min}^{-1}$$

This compares favorably with k_3 values reported for irreversible tracers such as [^{11}C]PMP (0.03-0.35 min^{-1}) and N-[^{11}C]methylspiperone (0.06 min^{-1}) in their respective target regions[3, 4].

In pretargeted imaging experiments (Section S9), the amount of intravenously injected ^{18}F-Tz was lower than the amount of intracerebrally injected TCO-polymer, even after correction for the possible loss of polymer from the injection site. This means that the ligation of ^{18}F-Tz to the TCO-polymer at the injection site in vivo should follow pseudo first-order kinetics, with the half-life determined by the local TCO polymer concentration and independent of the concentration of ^{18}F-Tz diffusing towards injection site.

Covariability of in silico parameters for the designed Tzs

The cross-correlation matrix of the in silico calculated parameters for the designed Tzs is shown in Figure S1. Only group V tetrazines contained a protonatable group, so ClogD and ClogP closely correlated with each other (rho 0.80). High covariance was also observed between BBB and CNS MPO scores (rho 0.84), and both scores were strongly inversely correlated with topological polar surface area (TPSA, rho -0.88 and -0.96, respectively). Many of the Tzs had equal TPSA and CNS MPO scores, because TPSA for homologous compounds is the same, while CNS MPO scores have a “binned” distribution, with physicochemically similar structures often falling into the same bin. Therefore, when looking for correlations between in silico properties of the designed Tzs and their in vitro and in vivo characteristics, we prioritized ClogD and ClogP values over other in silico parameters.

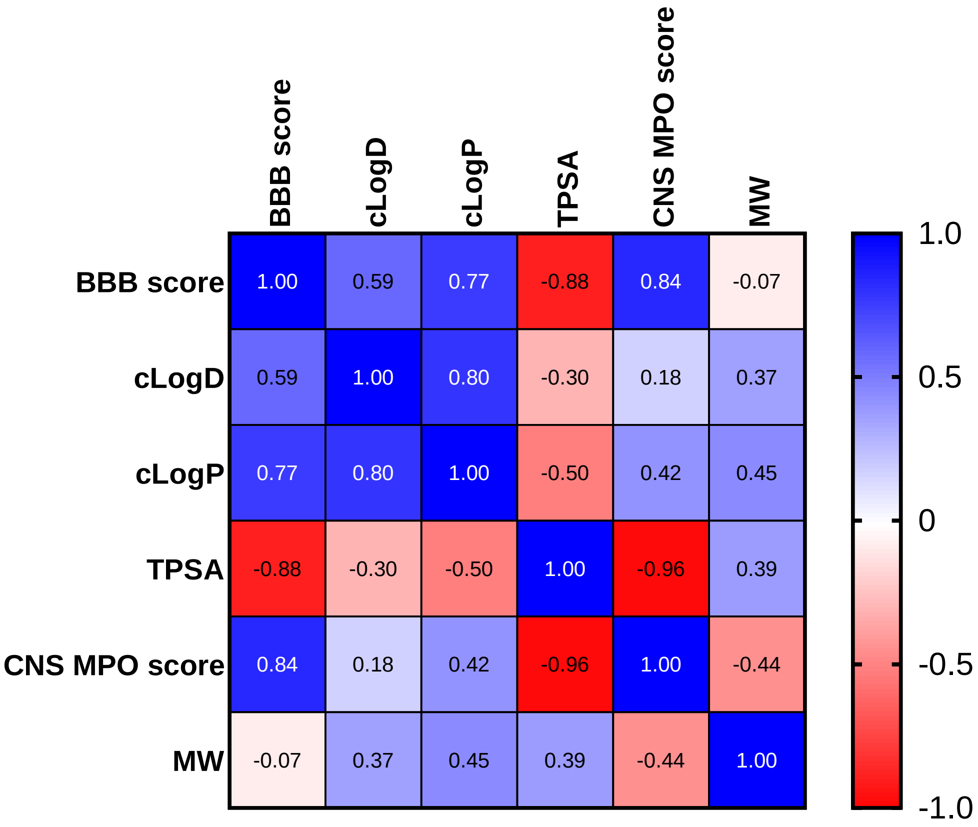

Figure S1. Correlation matrix for in silico properties of designed Tzs.

Spearman rank correlation coefficients (rho) between pairs of parameters are shown.

Putative relationship between Tz lipophilicity and relative contrast of in vitro pretargeted autoradiography and in vivo pretargeted imaging

In the pretargeted autoradiography setup (Section S7), there is no BBB which would limit the access of Tz to brain tissue. Therefore, the only effect of Tz lipophilicity (cLogP) increase is the increased non-specific binding, which results in a general negative correlation of the observed Cor/Cer ratios with cLogP (Figure S43). In the in vivo pretargeted imaging setup, increased cLogP not only increases non-displaceable binding of the Tz, but also improves its penetration across the BBB.

The existence of an optimal lipophilicity interval for brain imaging has long been discussed in the literature[5]. If such an optimal interval exists for cLogP values, then it should contain a threshold cLogP value, at which the relative imaging contrast trend is reversed. Namely, below the threshold, cLogP increase raises BBB penetration to a greater extent than non-displaceable binding, which leads to an improvement of relative imaging contrast. However, above the threshold, non-displaceable binding grows faster, and relative imaging contrast deteriorates. The exact cLogP value

corresponding to this threshold cannot be estimated from the current data, but apparently it was not exceeded within the current study. If it had been exceeded, we would have observed non-monotonous trends in both Figure 5C and Figure 5D.

Section S2: General Procedures

Methods. NMR spectra were acquired on a 600 MHz Bruker Avance III HD (600 MHz for ^{1}H and 151 MHz for ^{13}C), a 400 MHz Bruker Avance II (400 MHz for ^{1}H, 376 MHz for ^{19}F and 101 MHz for ^{13}C) and a 400 MHz Bruker Avance UltraShield (400 MHz for ^{1}H, 376 MHz for ^{19}F and 101 MHz for ^{13}C), using $CDCl_3$, MEOD or DMSO-d_6 as deuterated solvent and with the residual solvent as the internal reference. For all NMR experiences the deuterated solvent signal was used as the internal lock. Coupling constants (*J* values) are given in Hertz (Hz). Multiplicities of ^{1}H NMR signals are reported as follows: s, singlet; d, doublet; dd, doublet of doublets; ddd, doublet of doublets of doublets; dt, doublet of triplets; t, triplet; q, quartet; m, multiplet; br, broad signal. NMR spectra of all compounds are reprocessed in MestReNova software (version 12.0.22023) from original FID's files. Yields refer to isolated compounds estimated to be >90% pure as determined by ^{1}H NMR (25°C) and analytical HPLC (Supporting Information S12). Analytical HPLC method: Thermo Fisher UltiMate 3000 with a C-18 column (Luna 5 µm C18(2) 100 Å, 150 mm × 4.6 mm). Eluents: A, H_2O with 0.1% TFA; B, MeCN with 0.1% TFA. Gradient from 100% A to 100% B over 12 min, back to 100% A over 3 min, flow rate 2 mL/min. Detection by UV absorption at λ = 254 nm on a UVD 170U detector. Thin-layer chromatography (TLC) was carried out on silica gel 60 F_{254} plates from Merck (Germany). Visualization was accomplished by UV lamp (254 nm). Preparative HPLC was carried out on an UltiMate HPLC system (Thermo Scientific) consisting of an LPG-3200BX pump (10 mL/min), a Rheodyne 9725i injector, a 10 ml loop, a MWD-3000SD detector (254 nm), and an AFC-3000SD automated fraction collector, using a Gemini-NX C18 column (21.2 x 250 mm, 5 µm, 110Å) (Phenomenex) equipped with a guard. Purifications were performed using linear gradients of 0.1% TFA in MiliQ-H_2O (A) and 0.1% TFA, 10% MiliQ-H_2O in MeCN (B). Data was acquired and processed using Chromeleon Software v. 6.80. Semi-preparative HPLC was performed on the same system using a Luna 5µ C18 column (250 × 10 mm) with a flow rate of 3 mL/min. Automated Flash Column Chromatography was performed on a CombiFlash NextGen 300+ system supplied by TeleDyne ISCO, equipped with RediSep silica packed columns. Detection of the compounds was carried out by means of a UV-Vis variable wavelength detector operating from 200 to 800 nm and by Evaporative Light Scattering Detector (ELSD). Solvent systems for separation were particular for each compound but consisted of various mixtures of heptane, EtOAc, DCM and MeOH. Microwave-assisted synthesis was carried out in a Biotage Initiator apparatus operating in single mode; the microwave cavity producing controlled irradiation at 2.45 GHz (Biotage AB, Uppsala, Sweden). The reactions were run in sealed vessels. These experiments were performed by employing magnetic stirring and a fixed hold time using variable power to reach (during 1–2 min) and then maintain the desired temperature in the vessel for the programmed time period. The temperature was monitored

by an IR sensor focused on a point on the reactor vial glass. The IR sensor was calibrated to internal solution reaction temperature by the manufacturer. Mass spectra analysis was completed using MS-Acquity-A: Waters Acquity UPLC with QDa-detector.

Materials. All reagents and solvents were purchased from commercial suppliers and used without further purification. Anhydrous tetrahydrofuran (THF) was obtained from a SG Water solvent purification system (Pure Process Technology). Anhydrous dimethyl sulfoxide (DMSO), N,N-dimethylacetamide (DMA), acetonitrile (MeCN) and pyridine were purchased from commercial suppliers and stored under argon. Reactions requiring anhydrous conditions were carried out under inert atmosphere (nitrogen or argon) and using oven-dried glassware (152 ˚C). Syringes used to transfer anhydrous solvents or reagents were purged with argon prior to use. Other solvents were analytical or HPLC grade and were used as received.

The following abbreviations are used: MW: microwave; DCM: dichloromethane; DMSO: dimethyl sulfoxide; MeOH: methanol; EtOAc: ethyl acetate; PIDA: phenyliodine(III) diacetate; DMF: dimethylformamide; DEAD: diethyl azodicarboxylate; DIPEA: N,N-diisopropylethylamine; TEA: triethylamine; THF: tetrahydrofuran; EtOH: ethanol; Et_2O: diethyl ether; *m*CPBA: meta-chloroperoxybenzoic acid; MeCN/ACN: acetonitrile; TFA: trifluoroacetic acid; ^{me}CgPPh: 1,3,5,7-tetramethyl-6-phenyl-2,4,8-trioxa-6-phosphaadamantane;

Section S3: Synthetic Schemes

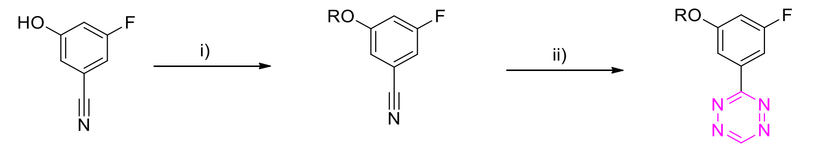

Scheme S1. Reagents and conditions: *i*) BrR or IR, K_2CO_3, MeCN, 1 h; *ii*) CH_2Cl_2, S_8, $NH_2NH_2 \cdot H_2O$, EtOH, 50 ºC, 24 h.

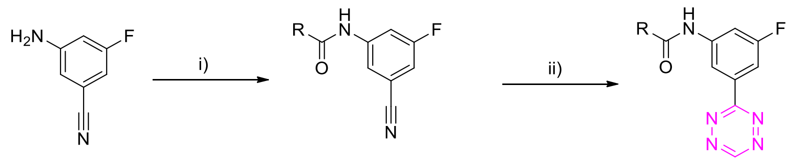

Scheme S2. Reagents and conditions: *i*) $(RCO)_2O$, K_2CO_3, DCM, 12 h; *ii*) CH_2Cl_2, S_8, $NH_2NH_2 \cdot H_2O$, EtOH, 50 ºC, 24 h.

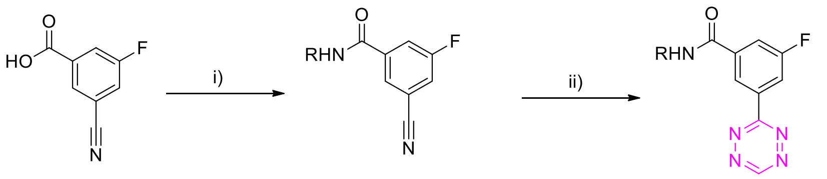

Scheme S3. Reagents and conditions: *i*) CDI, NH_2R, MeCN, 1 h; *ii*) CH_2Cl_2, S_8, $NH_2NH_2 \cdot H_2O$, EtOH, 50 ºC, 24 h.

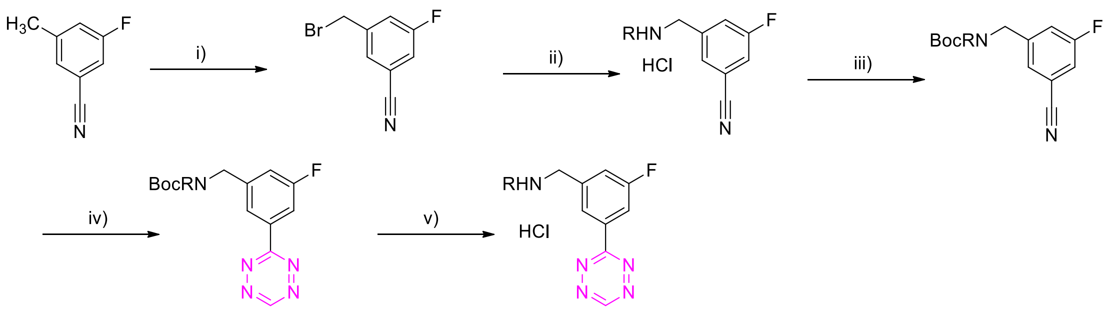

Scheme S4. Reagents and conditions: : *i*) NBS, AIBN, MeCN, reflux 12 h; *ii*) NH_4OH, THF, 50 ºC, 5 h; iii) Boc_2O, Et_3N, DCM, rt, 12 h; iv) CH_2Cl_2, S_8, $NH_2NH_2 \cdot H_2O$, EtOH, 50 ºC, 24 h; v) HCl, dioxane, rt, 2 h.

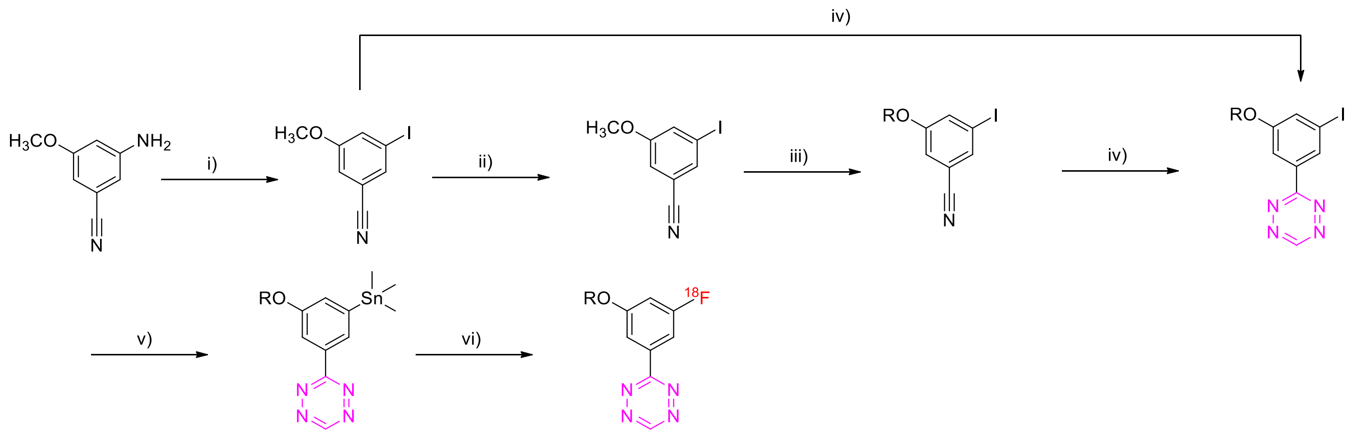

Scheme S5. Reagents and conditions: *i*) $NaNO_2$, HCl, KI, H_2O, 0 ºC to reflux, 2 h; *ii*) BBr_3, DCM, rt, 72 h; *iii*) BrR, K_2CO_3, MeCN, 1 h; *iv*) CH_2Cl_2, S_8, $NH_2NH_2 \cdot H_2O$, EtOH, 50 ºC, 24 h; *v*) $(Me_3Sn)_2$, $Pd(PPh_3)_4$, THF, 65 ºC, MW, 2 h; *vi*) $Cu(OTf)_2$, pyridine, [^{18}F]KF, DMA, 5 min, 100 °C.

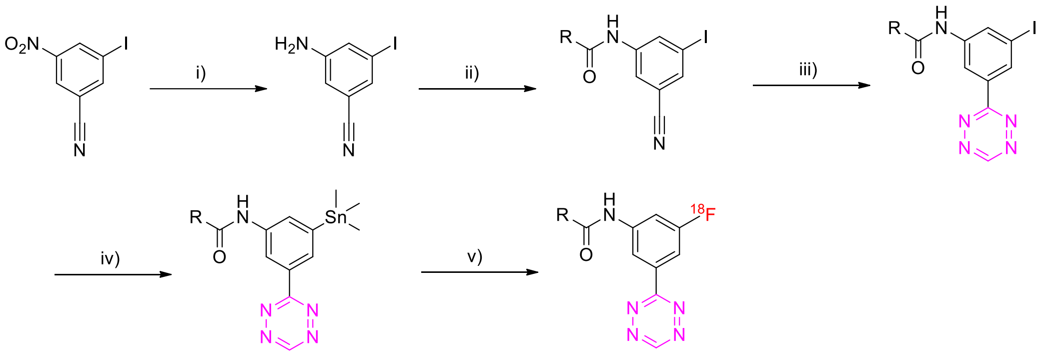

Scheme S6. Reagents and conditions: *i)* AcOH, Zn*,* MeOH, rt, 2 h; *ii*) $(RCO)_2O$, K_2CO_3, DCM, 12 h; i*ii*) CH_2Cl_2, S_8, $NH_2NH_2 \cdot H_2O$, EtOH, 50 ºC, 24 h; *iv*) $(Me_3Sn)_2$, $Pd(PPh_3)_4$, THF, 65 ºC, MW, 2 h; *v*) $Cu(OTf)_2$, pyridine, [^{18}F]KF, DMA, 5 min, 100 °C.

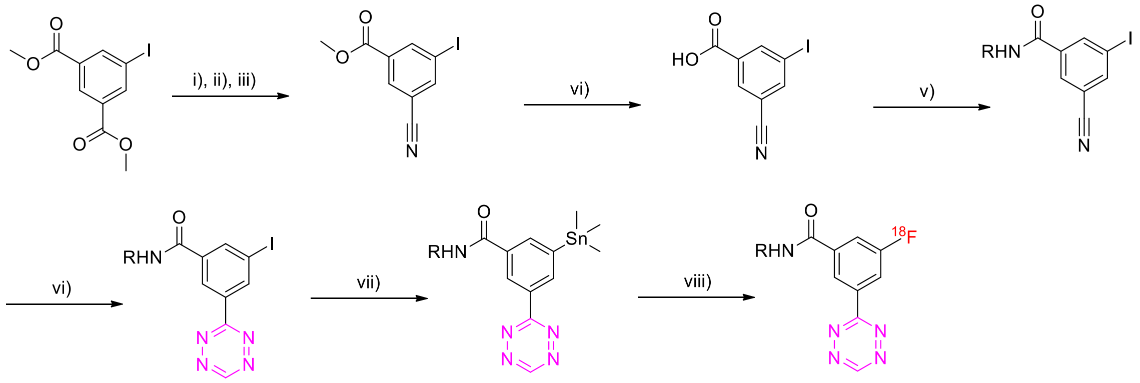

Scheme S7. Reagents and conditions: *i*) NaOH, MeOH, H_2O, rt, 48 h; *ii*) CDI, NH_4OH, MeCN, 1 h, 85%; *iii*) $SOCl_2$, reflux, 4 h; *iv*) *i*) NaOH, MeOH, H_2O, rt, 48 h; *v*) CDI, NH_4OH, MeCN, 1 h; *vi*)

CH_2Cl_2, S_8, $NH_2NH_2 \cdot H_2O$, EtOH, 50 ºC, 24 h; *vii*) $(Me_3Sn)_2$, $Pd(PPh_3)_4$, THF, 65 ºC, MW, 2 h; *viii*) $Cu(OTf)_2$, pyridine, [^{18}F]KF, DMA, 5 min, 100 °C.

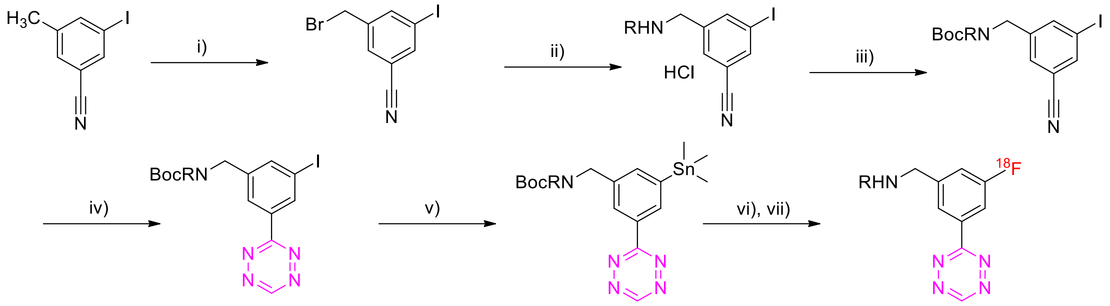

Reagents and conditions: *i*) NBS, AIBN, MeCN, reflux 12 h; *ii*) NH_2R, THF, 50 ºC, 5 h; iii) Boc_2O, Et_3N, DCM, rt, 12 h; iv) CH_2Cl_2, S_8, $NH_2NH_2 \cdot H_2O$, EtOH, 50 ºC, 24 h; v) $(Me_3Sn)_2$, $Pd(PPh_3)_4$, THF, 65 ºC, MW, 2 h; *vi*) $Cu(OTf)_2$, pyridine, [^{18}F]KF, DMA, 5 min, 100 °C; *vii)* TFA, MeCN, 70 ºC, 10 min.

Section S4: Organic Synthesis

General Procedure A.

The preparation of these intermediates was performed using a method described previously.[6] CH_2Cl_2 (0.256 mL, 4.00 mmol, 1 equiv.), sulfur (0.257 g, 1.00 mmol, 0.25 equiv.), hydrazine monohydrate (1.6 mL, 32.00 mmol, 8 equiv.) and ethanol (4.0 mL) along with the appropriate nitrile (4 mmol, 1 equiv.) were added to a microwave vial equipped with a stir bar. The vessel was sealed, and the reaction mixture was heated to 50 °C for 24 hours, before being allowed to cool to room temperature and unsealed. Then 3 ml of CH_2Cl_2 and $NaNO_2$ (2.8 g, 40.00 mmol, 10 equiv.) in water (40 ml) were added to the now yellow mixture followed by dropwise addition of acetic acid (14 mL), producing a mixture red in colour. The reaction mixture was extracted with CH_2Cl_2, washed with brine, dried with $MgSO_4$ and filtered before concentrating *in vacuo*. The crude was then purified via flash chromatography to afford the selected tetrazine.

General Procedure B

The selected phenol (1.0 mmol) and K_2CO_3 (2.5 mmol) were suspended in MeCN (10 mL). Subsequently the selected alkyl iodide or bromide (1.5 mmol) was added dropwise. The reaction mixture was refluxed for 3 h. Water (20 mL) was then added was resulting solution was extracted with CH_2Cl_2, washed with brine, dried with $MgSO_4$ and filtered before concentrating under reduced pressure. Purification by flash chromatography afforded the desired compound.

General Procedure C

To a solution of the corresponding aniline (1.0 mmol) in CH_2Cl_2 (10.0 mL) was added the selected anhydride (1.40 mmol). The mixture was stirred at room temperature for 12 h. The suspension was filtered, and the solvent removed under vacuum. Purification by flash chromatography afforded the desire compound.

General Procedure D

To a solution of the selected benzoic acid (1.0 mmol) in MeCN (3 mL) was added 1,1'-carbonyldiimidazole (1.5 mmol). The mixture was stirred at room temperature for 45 min, before addition of the selected amine (excess). The reaction mixture was stirred for 45 min and ice-cold water (15 ml) was added. The precipitate was collected by filtration and dried to give the desired compound.

General Procedure E

To a solution of the appropriate benzyl bromide (1.0 mmol) in THF (5 mL) was added the selected amine (10.0 mmol). The reaction was stirred at room temperature for 5 h. The solution was concentrated and taken up with water (20 mL) and the resulting solution was extracted with CH_2Cl_2 (2 x 20 mL), washed with brine, dried with $MgSO_4$ and filtered before concentrating under reduced pressure to afford the free amine as a yellow oil. The compound was resolubilized in Et_2O and HCl in Et_2O was added. The solid obtained was filtered and crystallized from MeOH/Et_2O to give the desired compound as HCl salt.

General Procedure F

The selected amine (1.0 mmol) and triethylamine (3.0 mmol) were dissolved in anhydrous CH_2Cl_2 (10 mL) at 0 °C. To this stirred solution was added di-tert-butyl dicarbonate (1.1 mmol), and the reaction allowed to warm to room temperature and stirred for 12 hours. The reaction mixture was evaporated under reduced pressure, and the residue was re-dissolved in diethyl ether (10 mL), which was washed successively with 0.5 M aq. HCl (2 x 5 mL), saturated $NaHCO_3$ (2 x 5 mL) and brine (5 mL). The organic layer was dried over anhydrous $MgSO_4$, filtered and evaporated under reduced pressure to give a white solid. The residue was purified by flash column chromatography to afford the desired compound.

General Procedure G

To a solution of the selected Boc protected amine (1.00 mmol) in CH_2Cl_2 (10 mL) was added a solution of HCl in dioxane (4.0 M, 3.0 mL). The mixture was stirred at room temperature for 2 h. The reaction was then concentrated under reduced pressure to give the desire amine as HCl salt.

General Procedure H

The preparation of these intermediates, was performed using a method described previously with minor modifications.[7] $Pd(PPh_3)_4$ (19.4 mg, 10%) and hexamethylditin (87 µL, 0.42 mmol, 2.5 equiv.) were successively added to a microwave vial equipped with a stir bar which was then sealed and purged with N_2. A solution of the appropriate iodo-phenyl-1,2,4,5-tetrazine (0.17 mmol) in dry THF (2.5 mL) was added via a syringe and the reaction allowed to stir at 65 ^{o}C in a microwave for 2 hours. The reaction was allowed to cool to room temperature and unsealed before being quenched with saturated aqueous KF (1 mL). The solution was extracted with CH_2Cl_2 washed with brine, dried over $MgSO_4$, filtered and concentrated under reduced pressure. The tetrazine was then purified via automatic flash chromatography to afford the desired product.

Synthesis of reference compounds

3-(3-Fluorophenyl)-1,2,4,5-tetrazine

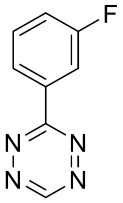

The compound was prepared as previously described by García-Vázquez et al.[7, 8] The final tetrazine was obtained from 3-fluorobenzonitrile (242 mg, 4 mmol) following general procedure A. The crude was purified using flash chromatography (90/10 n-Heptane/EtOAc) to yield 0.12 g (34%) of a red solid. *Rf* = 0.34 (n-Heptane:10%EtOAC); ^{1}H NMR (400 MHz, $CDCl_3$) δ 10.25 (s, 1H), 8.44 (dt, *J* = 7.8, 1.3 Hz, 1H), 8.33 (ddd, *J* = 9.7, 2.7, 1.6 Hz, 1H), 7.60 (td, *J* = 8.1, 5.7 Hz, 1H), 7.36 (tdd, *J* = 8.3, 2.7, 1.0 Hz, 1H); ^{13}C NMR (101 MHz, $CDCl_3$) δ 165.87 (d, *J* = 3.3 Hz), 163.47 (d, *J* = 247.6 Hz), 158.17, 133.91 (d, *J* = 8.2 Hz), 131.23 (d, *J* = 8.0 Hz), 124.18 (d, *J* = 3.2 Hz), 120.35 (d, *J* = 21.3 Hz), 115.32 (d, *J* = 24.1 Hz); ^{19}F NMR (376 MHz, $CDCl_3$) δ -107.77; MS (ESI) m/z $[M + H]^+$: 177.1.

3-(3-Fluoro-5-methylphenyl)-1,2,4,5-tetrazine

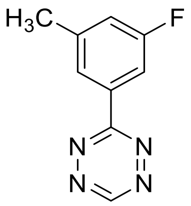

The compound was prepared as previously described by García-Vázquez et al.[7, 8] The final tetrazine was obtained from 3-fluoro-5-methylbenzonitrile (0.54 g, 4.00 mmol) following general procedure A. The crude was purified using flash chromatography (95/5 n-Heptane/EtOAc) to yield 0.26 g (34%) of a red oil. *Rf* = 0.39 (n-Heptane:20%EtOAc); ^{1}H NMR (400 MHz, $CDCl_3$) δ 10.16 (s, 1H), 8.19 (d, *J* = 1.4 Hz, 1H), 8.05 (d, *J* = 9.4 Hz, 1H), 7.10 (d, *J* = 9.2 Hz, 1H), 2.43 (s, 3H); ^{13}C NMR (101 MHz, $CDCl_3$) δ 165.83 (d, *J* = 3.4 Hz), 163.30 (d, *J* = 246.8 Hz), 157.95, 141.81 (d, *J* = 7.7 Hz), 133.31 (d, *J* = 8.9 Hz), 124.67 (d, *J* = 2.7 Hz), 120.79 (d, *J* = 21.2 Hz), 112.29 (d, *J* = 24.3 Hz), 21.45 (d, *J* = 1.8 Hz); ^{19}F NMR (376 MHz, $CDCl_3$) δ -109.14.; MS (ESI) m/z $[M + H]^+$: 191.1.

3-(3-fluoro-5-methoxyphenyl)-1,2,4,5-tetrazine

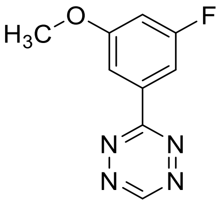

The compound was prepared as previously described by García-Vázquez et al.[7, 8] The final tetrazine was obtained from 3-fluoro-5-methoxylbenzonitrile (0.60 g, 4.00 mmol) following general procedure A. The crude was purified using flash chromatography (85/15 n-Heptane/EtOAc) to give 0.21 g (26%) of a red solid. *Rf* = 0.41 (n-Heptane:20%EtOAc); ^{1}H NMR (400 MHz, $CDCl_3$) δ 10.17 (s, 1H), 7.91 (d, *J* = 2.0 Hz, 1H), 7.87 (ddd, *J* = 9.1, 2.4, 1.4 Hz, 1H), 6.83 (dd, *J* = 10.1, 2.4 Hz, 1H), 3.86 (s, 3H); ^{13}C NMR (101 MHz, $CDCl_3$) δ 165.63 (d, *J* = 3.9 Hz), 164.07 (d, *J* = 246.5 Hz), 161.75 (d, *J* = 11.4 Hz), 158.02, 133.96 (d, *J* = 10.7 Hz), 108.96 (d, *J* = 2.8 Hz), 107.73 (d, *J* = 24.7 Hz), 106.98 (d, *J* = 24.9 Hz), 55.96; ^{19}F NMR (376 MHz, $CDCl_3$) δ -109.58; MS (ESI) m/z $[M + H]^+$: 207.1.

3-Ethoxy-5-fluorobenzonitrile

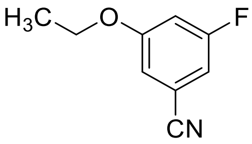

The compound was obtained from 3-hydroxy-5-fluorobenzonitrile (1.00 g, 7.29 mmol) and iodoethane (1.70 g, 10.94 mmol) following the general procedure B. The crude was purified using flash chromatography (85/15 n-Heptane/EtOAc) to give 1.21 g (99%) of 3-ethoxy-5-

fluorobenzonitrile as a white solid. *Rf* = 0.48 (n-Heptane:20%EtOAc); ^{1}H NMR (400 MHz, $CDCl_3$) δ 6.95 – 6.80 (m, 2H), 6.76 (dt, *J* = 10.5, 2.3 Hz, 1H), 3.97 (q, *J* = 7.0 Hz, 2H), 1.36 (t, *J* = 7.0 Hz, 3H); ^{13}C NMR (101 MHz, $CDCl_3$) δ 163.13 (d, *J* = 248.8 Hz), 160.69 (d, *J* = 11.6 Hz), 117.68 (d, *J* = 3.9 Hz), 114.21 (d, *J* = 3.3 Hz), 113.85 (d, *J* = 12.2 Hz), 111.16 (d, *J* = 25.2 Hz), 107.39 (d, *J* = 24.4 Hz), 64.52, 14.44; ^{19}F NMR (376 MHz, $CDCl_3$) δ -108.45.

3-(3-Ethoxy-5-fluorophenyl)-1,2,4,5-tetrazine

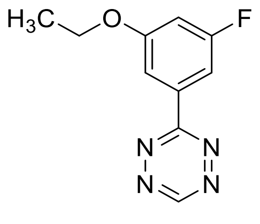

The final tetrazine was obtained from 3-fluoro-5-ethoxylbenzonitrile (1.14 g, 7.02 mmol) following general procedure A. The crude was purified using flash chromatography (85/15 n-Heptane/EtOAc) and recrystallized from n-Heptane to give 0.46 g (30%) of a red solid. *Rf* = 0.49 (n-Heptane:20%EtOAc); ^{1}H NMR (400 MHz, $CDCl_3$) δ 10.16 (s, 1H), 8.24 – 7.63 (m, 2H), 6.81 (dq, *J* = 10.2, 2.3 Hz, 1H), 4.09 (q, *J* = 7.0 Hz, 2H), 1.40 (t, *J* = 7.0 Hz, 3H); ^{13}C NMR (101 MHz, $CDCl_3$) δ 165.67 (d, *J* = 3.8 Hz), 164.06 (d, *J* = 246.4 Hz), 161.09 (d, *J* = 11.4 Hz), 133.87 (d, *J* = 10.5 Hz), 109.50 (d, *J* = 2.8 Hz), 107.57 (d, *J* = 14.7 Hz), 107.32 (d, *J* = 14.9 Hz), 64.36, 14.62; ^{19}F NMR (376 MHz, $CDCl_3$) δ -109.64; MS (ESI) m/z $[M + H]^+$: 221.0.

3-Fluoro-5-propoxybenzonitrile

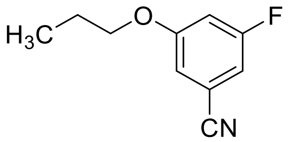

The compound was obtained from 3-hydroxy-5-fluorobenzonitrile (1.00 g, 7.29 mmol) and 1-bromopropane (1.34 g, 10.94 mmol) following the general procedure B. The crude was purified using flash chromatography (85/15 n-Heptane/EtOAc) to give 1.13 g (86%) of 3-propoxy-5-fluorobenzonitrile as a white solid. *Rf* = 0.31 (n-Heptane:10%EtOAc); ^{1}H NMR (400 MHz, $CDCl_3$) δ 6.92 – 6.83 (m, 2H), 6.77 (dt, *J* = 10.5, 2.4 Hz, 1H), 3.86 (t, *J* = 6.5 Hz, 2H), 1.75 (h, *J* = 7.1 Hz, 2H), 0.97 (t, *J* = 7.4 Hz, 3H); ^{13}C NMR (101 MHz, $CDCl_3$) δ 163.14 (d, *J* = 248.6 Hz), 160.88 (d, *J* = 11.5 Hz), 117.70 (d, *J* = 3.9 Hz), 114.24 (d, *J* = 3.1 Hz), 113.81 (d, *J* = 12.1 Hz), 111.14 (d, *J* = 25.2 Hz), 107.44 (d, *J* = 24.5 Hz), 70.44, 22.25, 10.35; ^{19}F NMR (376 MHz, $CDCl_3$) δ -108.47.

3-(3-Fluoro-5-propoxyphenyl)-1,2,4,5-tetrazine

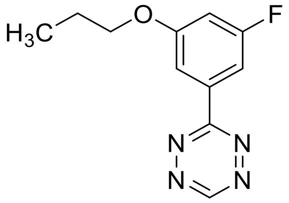

The final tetrazine was obtained from 3-fluoro-5-propoxylbenzonitrile (1.01 g, 5.63 mmol) following general procedure A. The crude was purified using flash chromatography (85/15 n-Heptane/EtOAc) to give 0.38 g (29%) of a red solid. *Rf* = 0.31 (n-Heptane:10%EtOAc); ^{1}H NMR (400 MHz, $CDCl_3$) δ 10.16 (s, 1H), 7.90 (t, *J* = 1.9 Hz, 1H), 7.87 – 7.81 (m, 1H), 6.81 (dq, *J* = 10.2, 2.3, 1.9 Hz, 1H), 3.97 (t, *J* = 6.5 Hz, 2H), 1.79 (h, *J* = 7.1 Hz, 2H), 1.00 (t, *J* = 7.4 Hz, 3H); ^{13}C NMR (101 MHz, $CDCl_3$) δ 165.68 (d, *J* = 3.9 Hz), 164.06 (d, *J* = 246.2 Hz), 161.30 (d, *J* = 11.3 Hz), 133.84 (d, *J* = 10.6 Hz), 109.56 (d, *J* = 2.7 Hz), 107.53 (d, *J* = 10.3 Hz), 107.29 (d, *J* = 10.4 Hz), 70.32, 22.42, 10.45; ^{19}F NMR (376 MHz, $CDCl_3$) δ -109.68; MS (ESI) m/z $[M + H]^+$: 235.1.

3-(Benzyloxy)-5-fluorobenzonitrile

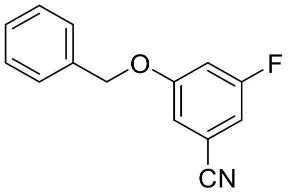

The compound was obtained from 3-hydroxy-5-fluorobenzonitrile (1.00 g, 7.29 mmol) and benzylbromide (1.84 g, 10.94 mmol) following the general procedure B. The crude was purified using flash chromatography (85/15 n-Heptane/EtOAc) to give 1.41 g (85%) of 3-(benzyloxy)-5-fluorobenzonitrile as a white solid. *Rf* = 0.48 (n-Heptane:20%EtOAc); ^{1}H NMR (400 MHz, $CDCl_3$) δ 7.41 – 7.22 (m, 5H), 6.96 (dt, *J* = 2.0, 0.9 Hz, 1H), 6.92 – 6.77 (m, 2H), 5.00 (s, 2H); ^{13}C NMR (101 MHz, $CDCl_3$) δ 163.11 (d, *J* = 249.3 Hz), 160.38 (d, *J* = 11.5 Hz), 135.23, 128.84, 128.59, 127.48, 117.59 (d, *J* = 3.8 Hz), 114.61 (d, *J* = 3.2 Hz), 114.00 (d, *J* = 12.0 Hz), 111.71 (d, *J* = 25.1 Hz), 107.94 (d, *J* = 24.5 Hz), 70.84; ^{19}F NMR (376 MHz, $CDCl_3$) δ -108.05.

3-(3-(Benzyloxy)-5-fluorophenyl)-1,2,4,5-tetrazine

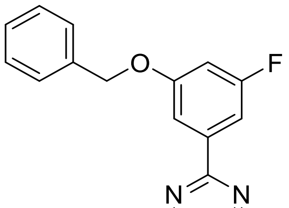

The final tetrazine was obtained from 3-(benzyloxy)-5-fluorobenzonitrile (1.36 g, 6.00 mmol) following general procedure A. The crude was purified using flash chromatography (85/15 n-

Heptane/EtOAc) to give 0.44 g (26%) of a red solid. *Rf* = 0.33 (n-Heptane:20%EtOAc); ^{1}H NMR (400 MHz, $CDCl_3$) δ 10.16 (s, 1H), 8.01 (t, *J* = 1.8 Hz, 1H), 7.88 (ddd, *J* = 9.1, 2.4, 1.4 Hz, 1H), 7.41 – 7.26 (m, 5H), 6.89 (dt, *J* = 10.0, 2.4 Hz, 1H), 5.11 (s, 2H); ^{13}C NMR (101 MHz, $CDCl_3$) δ 165.60 (d, *J* = 3.8 Hz), 162.82, 160.81 (d, *J* = 11.3 Hz), 158.03, 135.87, 134.00 (d, *J* = 10.6 Hz), 128.73, 128.35, 127.56, 109.90 (d, *J* = 2.8 Hz), 108.02 (d, *J* = 19.0 Hz), 107.78 (d, *J* = 19.2 Hz), 70.69; ^{19}F NMR (376 MHz, $CDCl_3$) δ -109.26; MS (ESI) m/z $[M + H]^+$: 283.1.

N-(3-Cyano-5-fluorophenyl)acetamide

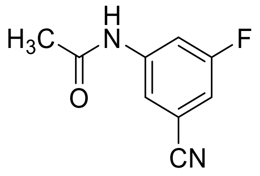

The compound was prepared as previously described by García-Vázquez et al.[7, 8] The compound was obtained from 3-amino-5-fluorobenzonitrile (0.82 g, 6.00 mmol) and acetic anhydride (0.80 mL, 8.40 mmol) following the general procedure C. The crude was purified using flash chromatography (70/30 n-Heptane/EtOAc) to give 0.92 g (86%) of N-(3-cyano-5-fluorophenyl)acetamide as a white solid. *Rf* = 0.31 (n-Heptane:40%EtOAc); ^{1}H NMR (400 MHz, DMSO-d_6) δ 10.45 (s, 1H), 7.86 – 7.70 (m, 2H), 7.57 – 7.37 (m, 1H), 2.09 (s, 3H); ^{13}C NMR (101 MHz, DMSO-d_6) δ 169.69, 162.24 (d, *J* = 244.3 Hz), 142.35 (d, *J* = 11.8 Hz), 118.65, 118.09 (d, *J* = 3.6 Hz), 113.70 (d, *J* = 25.5 Hz), 113.25 (d, *J* = 12.1 Hz), 110.95 (d, *J* = 26.2 Hz), 24.52; ^{19}F NMR (376 MHz, DMSO-d_6) δ -109.14.

N-(3-Fluoro-5-(1,2,4,5-tetrazin-3-yl)phenyl)acetamide

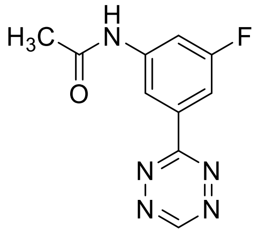

The compound was prepared as previously described by García-Vázquez et al.[7, 8] The final compound was obtained from N-(5-cyano-3-fluorophenyl)acetamide (0.58 g, 3.25 mmol) following general procedure A. The crude was purified using flash chromatography (60/40 n-Heptane/EtOAc) to yield 0.19 g (25%) of a red solid. *Rf* = 0.25 (n-Heptane:40%EtOAc); ^{1}H NMR (400 MHz, DMSO-d_6) δ 10.64 (s, 1H), 10.48 (s, 1H), 8.52 (t, *J* = 1.7 Hz, 1H), 7.98 – 7.81 (m, 2H), 2.12 (s, 3H); ^{13}C NMR (101 MHz, DMSO-d_6) δ 169.54, 165.03 (d, *J* = 3.8 Hz), 163.08 (d, *J* = 242.2 Hz), 158.83, 142.45 (d, *J* = 11.5 Hz), 134.61 (d, *J* = 10.1 Hz), 114.40 (d, *J* = 2.6 Hz), 109.87 (d, *J* = 26.6 Hz), 108.74 (d, *J* = 24.4 Hz), 24.58; ^{19}F NMR (376 MHz, DMSO-d_6) δ -110.23; MS (ESI) m/z $[M + H]^+$: 234.1.

N-(3-Cyano-5-fluorophenyl)propionamide

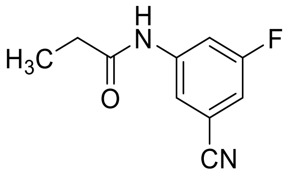

The compound was obtained from 3-amino-5-fluorobenzonitrile (0.82 g, 6.00 mmol) and propionic anhydride (1.08 mL, 8.40 mmol) following the general procedure C. The crude was purified using flash chromatography (70/30 n-Heptane/EtOAc) to give 0.92 g (80%) of N-(3-cyano-5-fluorophenyl)proprionamide as a white solid. *Rf* = 0.41 (n-Heptane:40%EtOAc); ^{1}H NMR (400 MHz, DMSO-d_6) δ 10.37 (s, 1H), 7.87 – 7.69 (m, 2H), 7.54 – 7.41 (m, 1H), 2.36 (q, *J* = 7.5 Hz, 2H), 1.09 (t, *J* = 7.5 Hz, 3H); ^{13}C NMR (101 MHz, DMSO-d_6) δ 173.33, 162.25 (d, *J* = 244.3 Hz), 142.42 (d, *J* = 11.9 Hz), 118.68 (d, *J* = 3.1 Hz), 118.10 (d, *J* = 3.7 Hz), 113.58 (d, *J* = 25.5 Hz), 113.23 (d, *J* = 12.0 Hz), 110.95 (d, *J* = 26.2 Hz), 30.02, 9.70; ^{19}F NMR (376 MHz, DMSO-d_6) δ -109.16.

N-(3-Fluoro-5-(1,2,4,5-tetrazin-3-yl)phenyl)propionamide

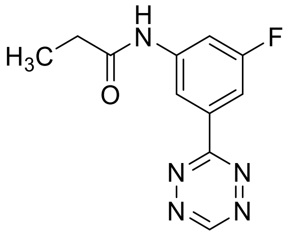

The final compound was obtained from N-(5-cyano-3-fluorophenyl)propionamide (0.77 g, 4.00 mmol) following general procedure A. The crude was purified using flash chromatography (60/40 n-Heptane/EtOAc) to yield 0.35 g (35%) of a red solid. *Rf* = 0.31 (n-Heptane:40%EtOAc); ^{1}H NMR (400 MHz, DMSO-d_6) δ 10.64 (s, 1H), 10.41 (s, 1H), 8.56 (d, *J* = 1.7 Hz, 1H), 8.00 – 7.81 (m, 2H), 2.40 (q, *J* = 7.5 Hz, 2H), 1.12 (t, *J* = 7.5 Hz, 3H); ^{13}C NMR (101 MHz, DMSO-d_6) δ 173.22, 165.03, 163.10 (d, *J* = 242.2 Hz), 158.84, 142.53 (d, *J* = 11.6 Hz), 134.61 (d, *J* = 10.1 Hz), 116.45 – 113.43 (m), 109.92 (d, *J* = 26.6 Hz), 108.65 (d, *J* = 24.3 Hz), 30.09, 9.86; ^{19}F NMR (376 MHz, DMSO-d_6) δ -110.24; MS (ESI) m/z $[M + H]^+$: 248.1.

N-(3-Cyano-5-fluorophenyl)butyramide

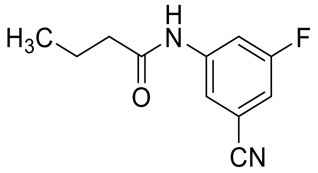

The compound was obtained from 3-amino-5-fluorobenzonitrile (0.82 g, 6.00 mmol) and butyric anhydride (1.37 mL, 8.40 mmol) following the general procedure C. The crude was purified using

flash chromatography (70/30 n-Heptane/EtOAc) to give 0.92 g (80%) of N-(3-Cyano-5-fluorophenyl)butyramide as a white solid. *Rf* = 0.43 (n-Heptane:40%EtOAc); ^{1}H NMR (400 MHz, DMSO-d_6) δ 10.37 (s, 1H), 8.34 – 7.69 (m, 2H), 7.47 (ddd, *J* = 8.4, 2.5, 1.4 Hz, 1H), 2.32 (t, *J* = 7.3 Hz, 2H), 1.62 (h, *J* = 7.4 Hz, 2H), 0.92 (t, *J* = 7.4 Hz, 3H); ^{13}C NMR (101 MHz, DMSO-d_6) δ 172.48, 162.24 (d, *J* = 244.3 Hz), 142.36 (d, *J* = 11.8 Hz), 118.69 (d, *J* = 3.0 Hz), 118.08 (d, *J* = 3.8 Hz), 113.60 (d, *J* = 25.6 Hz), 113.24 (d, *J* = 12.1 Hz), 110.96 (d, *J* = 26.1 Hz), 38.77, 18.70, 13.97; ^{19}F NMR (376 MHz, DMSO-d_6) δ -109.17.

N-(3-Fluoro-5-(1,2,4,5-tetrazin-3-yl)phenyl)butyramide

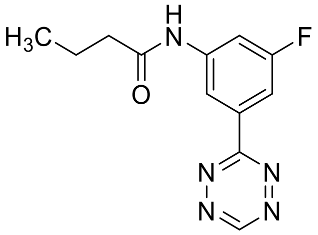

The final compound was obtained from N-(3-cyano-5-fluorophenyl)butyramide (0.82 g, 4.00 mmol) following general procedure A. The crude was purified using flash chromatography (60/40 n-Heptane/EtOAc) to yield 0.38 g (36%) of a red solid. *Rf* = 0.35 (n-Heptane:40%EtOAc); ^{1}H NMR (400 MHz, DMSO-d_6) δ 10.64 (s, 1H), 10.41 (s, 1H), 8.56 (t, *J* = 1.7 Hz, 1H), 8.01 – 7.77 (m, 2H), 2.36 (t, *J* = 7.3 Hz, 2H), 1.65 (h, *J* = 7.4 Hz, 2H), 0.94 (t, *J* = 7.4 Hz, 3H); ^{13}C NMR (101 MHz, DMSO-d_6) δ 172.38, 165.04 (d, *J* = 3.5 Hz), 163.08 (d, *J* = 242.2 Hz), 158.83, 142.46 (d, *J* = 11.5 Hz), 134.60 (d, *J* = 10.2 Hz), 114.50 (d, *J* = 2.6 Hz), 109.93 (d, *J* = 26.6 Hz), 108.69 (d, *J* = 24.5 Hz), 38.85, 18.84, 14.04; ^{19}F NMR (376 MHz, DMSO-d_6) δ -110.25; MS (ESI) m/z $[M + H]^+$: 262.1.

N-(3-Cyano-5-fluorophenyl)pentanamide

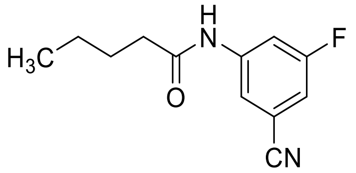

The compound was obtained from 3-amino-5-fluorobenzonitrile (0.35 g, 2.57 mmol) and valeric anhydride (0.71 mL, 3.60 mmol) following the general procedure C. The crude was purified using flash chromatography (70/30 n-Heptane/EtOAc) to give 0.51 g (91%) of N-(3-cyano-5-fluorophenyl)pentanamide as a white solid. *Rf* = 0.45 (n-Heptane:40%EtOAc); ^{1}H NMR (400 MHz, $CDCl_3$) δ 7.70 (dt, *J* = 10.6, 2.2 Hz, 1H), 7.62 (s, 1H), 7.50 (t, *J* = 1.8 Hz, 1H), 7.01 (ddd, *J* = 7.6, 2.4, 1.3 Hz, 1H), 2.37 – 2.28 (m, 2H), 1.64 (p, *J* = 7.5 Hz, 2H), 1.33 (h, *J* = 7.3 Hz, 2H), 0.88 (t, *J* = 7.4 Hz, 3H); ^{13}C NMR (101 MHz, $CDCl_3$) δ 172.02, 162.53 (d, *J* = 248.9 Hz), 140.69 (d, *J* = 11.4

Hz), 118.52 (d, *J* = 3.3 Hz), 117.45 (d, *J* = 3.7 Hz), 114.17 (d, *J* = 25.1 Hz), 113.71 (d, *J* = 11.2 Hz), 111.71 (d, *J* = 26.3 Hz), 37.37, 27.38, 22.29, 13.73; ^{19}F NMR (376 MHz, $CDCl_3$) δ -107.84.

N-(3-Fluoro-5-(1,2,4,5-tetrazin-3-yl)phenyl)pentanamide

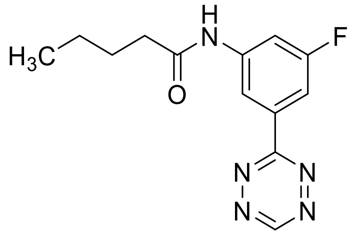

The final compound was obtained from N-(3-cyano-5-fluorophenyl)pentanamide (0.48 g, 2.18 mmol) following general procedure A. The crude was purified using flash chromatography (60/40 n-Heptane/EtOAc) to yield 0.15 g (25%) of a red solid. *Rf* = 0.38 (n-Heptane:40%EtOAc); ^{1}H NMR (400 MHz, $CDCl_3$) δ 10.17 (s, 1H), 8.20 (d, *J* = 1.7 Hz, 1H), 7.94 (ddd, *J* = 9.7, 4.9, 1.9 Hz, 2H), 7.66 (s, 1H), 2.37 (t, *J* = 7.6 Hz, 2H), 1.68 (p, *J* = 7.5 Hz, 2H), 1.36 (h, *J* = 7.4 Hz, 2H), 0.88 (t, *J* = 7.3 Hz, 3H); ^{13}C NMR (101 MHz, $CDCl_3$) δ 171.93, 165.37 (d, *J* = 3.7 Hz), 163.59 (d, *J* = 246.2 Hz), 158.05, 140.59 (d, *J* = 11.3 Hz), 133.56 (d, *J* = 10.0 Hz), 114.42, 111.85 (d, *J* = 27.1 Hz), 110.42 (d, *J* = 24.8 Hz), 37.49, 27.50, 22.34, 13.77; ^{19}F NMR (376 MHz, $CDCl_3$) δ -108.70; MS (ESI) m/z $[M + H]^+$: 276.1.

3-Cyano-5-fluorobenzamide

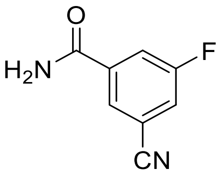

The compound was prepared as previously described by García-Vázquez et al. [7, 8] The compound was obtained from 5-cyano-3-fluorobenzoic acid (0.99 g, 6.0 mmol) and aqueous ammonium hydroxide solution (35%, 20 ml) following the general procedure D to give 0.77 g (78%) of 3-cyano-5-fluorobenzamide as a white solid. *Rf* = 0.31 (n-Heptane:60%EtOAc); ^{1}H NMR (400 MHz, DMSO-d_6) δ 8.21 (s, 1H), 8.16 (d, *J* = 1.5 Hz, 1H), 8.05 (ddd, *J* = 8.4, 2.6, 1.3 Hz, 1H), 8.01 (ddd, *J* = 9.6, 2.5, 1.4 Hz, 1H), 7.78 (s, 1H); ^{13}C NMR (101 MHz, DMSO-d_6) δ 165.12 (d, *J* = 2.4 Hz), 162.04 (d, *J* = 247.5 Hz), 138.39 (d, *J* = 7.3 Hz), 128.11 (d, *J* = 3.1 Hz), 122.37 (d, *J* = 25.7 Hz), 120.16 (d, *J* = 22.9 Hz), 117.69 (d, *J* = 3.1 Hz), 113.52 (d, *J* = 9.9 Hz).

3-Fluoro-5-(1,2,4,5-tetrazin-3-yl)benzamide

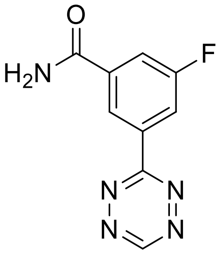

The compound was prepared as previously described by García-Vázquez et al. [7, 8] The final compound was obtained from 3-cyano-5-fluorobenzamide (0.75 g, 4.57 mmol) following general procedure A. The crude was purified using flash chromatography (98/2 CH_2Cl_2/MeOH) to yield 0.36 g (36%) of a pink solid. *Rf* = 0.31 (n-Heptane:60%EtOAC); ^{1}H NMR (400 MHz, DMSO-d_6) δ 10.69 (s, 1H), 8.88 (s, 1H), 8.48 – 8.20 (m, 2H), 8.16 – 7.92 (m, 1H), 7.71 (s, 1H); ^{13}C NMR (101 MHz, DMSO-d_6) δ 166.08 (d, *J* = 2.3 Hz), 164.94 (d, *J* = 3.3 Hz), 162.85 (d, *J* = 245.6 Hz), 158.89, 138.32 (d, *J* = 6.9 Hz), 134.93 (d, *J* = 8.2 Hz), 123.56 (d, *J* = 2.9 Hz), 118.85 (d, *J* = 23.0 Hz), 117.31 (d, *J* = 24.1 Hz); ^{19}F NMR (376 MHz, DMSO-d_6) δ -109.00; MS (ESI) m/z $[M + H]^+$: 220.0.

3-Cyano-5-fluoro-N-methylbenzamide

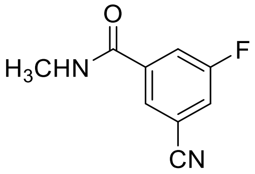

The compound was prepared as previously described by García-Vázquez et al.[7, 8] The compound was obtained from 5-cyano-3-fluorobenzoic acid (0.99 g, 6.0 mmol) and aqueous methylamine solution (40%, 20 ml) following the general procedure D to give 0.81 g (76%) of 5-cyano-3-fluoro-N-methylbenzamide as a white solid. *Rf* = 0.32 (n-Heptane:60%EtOAc); ^{1}H NMR (400 MHz, DMSO-d_6) δ 8.72 (d, *J* = 5.8 Hz, 1H), 8.11 (t, *J* = 1.5 Hz, 1H), 8.05 (ddd, *J* = 8.1, 2.7, 1.4 Hz, 1H), 7.97 (ddd, *J* = 9.5, 2.7, 1.5 Hz, 1H), 2.81 (d, *J* = 4.6 Hz, 3H); ^{13}C NMR (101 MHz, DMSO-d_6) δ 163.96 (d, *J* = 2.5 Hz), 162.02 (d, *J* = 247.5 Hz), 138.49 (d, *J* = 7.4 Hz), 127.76 (d, *J* = 3.3 Hz), 122.17 (d, *J* = 25.6 Hz), 119.85 (d, *J* = 23.1 Hz), 117.66 (d, *J* = 3.4 Hz), 113.55 (d, *J* = 10.0 Hz), 26.83.

3-Fluoro-N-methyl-5-(1,2,4,5-tetrazin-3-yl)benzamide

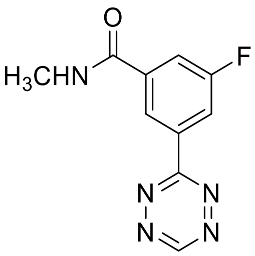

The compound was prepared as previously described by García-Vázquez et al.[7, 8] The final compound was obtained from 5-cyano-3-fluoro-N-methylbenzamide (0.62 g, 3.48 mmol) following general procedure A. The crude was purified using flash chromatography (98/2 CH_2Cl_2/MeOH) to afford 0.28 g (34%) of a pink solid. *Rf* = 0.31 (n-Heptane:60%EtOAC); ^{1}H NMR (400 MHz, DMSO-*d*$_6$) δ 10.70 (s, 1H), 8.87 (s, 2H), 8.39 (dd, *J* = 9.3, 1.9 Hz, 1H), 7.99 (dt, *J* = 9.5, 2.0 Hz, 1H), 2.85 (d, *J* = 4.5 Hz, 3H); ^{13}C NMR (101 MHz, DMSO-*d*$_6$) δ 164.88 (d, *J* = 11.1 Hz), 162.89 (d, *J* = 245.8 Hz), 158.92, 138.41 (d, *J* = 7.5 Hz), 135.00 (d, *J* = 8.2 Hz), 133.13, 123.08, 118.56 (d, *J* = 22.8 Hz), 117.16 (d, *J* = 24.0 Hz), 26.89; ^{19}F NMR (376 MHz, DMSO-*d*$_6$) δ -109.34; MS (ESI) m/z $[M + H]^+$: 234.1.

3-Cyano-N-ethyl-5-fluorobenzamide

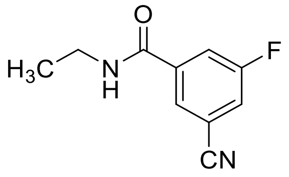

The compound was obtained from 5-cyano-3-fluorobenzoic acid (0.99 g, 6.0 mmol) and aqueous ethylamine solution (70%, 20 ml). following the general procedure D to give 1.05 g (91%) of 3-cyano-N-ethyl-5-fluorobenzamide as a white solid. *Rf* = 0.35 (n-Heptane:60%EtOAc); ^{1}H NMR (400 MHz, DMSO-*d*$_6$) δ 8.72 (t, *J* = 5.4 Hz, 1H), 8.13 (d, *J* = 1.4 Hz, 1H), 8.01 (dddd, *J* = 24.1, 9.5, 2.6, 1.5 Hz, 2H), 3.38 – 3.24 (m, 4H), 1.14 (t, *J* = 7.2 Hz, 3H); ^{13}C NMR (101 MHz, DMSO-*d*$_6$) δ 163.25, 162.01 (d, *J* = 244.1 Hz), 138.60 (d, *J* = 7.3 Hz), 127.79 (d, *J* = 3.4 Hz), 122.14 (d, *J* = 25.5 Hz), 119.91 (d, *J* = 22.9 Hz), 117.67 (d, *J* = 3.1 Hz), 113.51 (d, *J* = 10.0 Hz), 34.84, 14.91; ^{19}F NMR (376 MHz, DMSO-*d*$_6$) δ -109.90.

N-Ethyl-3-fluoro-5-(1,2,4,5-tetrazin-3-yl)benzamide

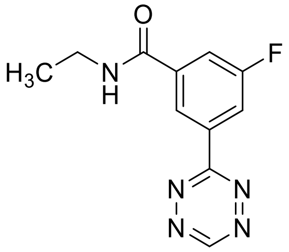

The final compound was obtained from 3-cyano-N-ethyl-5-fluorobenzamide (0.77 g, 4.00 mmol) following general procedure A. The crude was purified using flash chromatography (98/2 CH_2Cl_2/MeOH) to afford 0.28 g (28%) of a pink solid. *Rf* = 0.33 (n-Heptane:60%EtOAC); ^{1}H NMR (400 MHz, DMSO-*d*$_6$) δ 10.70 (s, 1H), 8.92 – 8-85 (m , 2H), 8.39 (ddd, *J* = 9.3, 2.6, 1.5 Hz, 1H), 8.00 (ddd, *J* = 9.5, 2.6, 1.5 Hz, 1H), 4.03 – 3.08 (m, 2H), 1.17 (t, *J* = 7.2 Hz, 3H); ^{13}C NMR (101 MHz,

DMSO-d_6) δ 164.95, 164.09, 162.88 (d, *J* = 247.7 Hz), 158.91, 138.57 (d, *J* = 7.0 Hz), 134.95 (d, *J* = 8.5 Hz), 123.12 (d, *J* = 2.7 Hz), 118.63 (d, *J* = 23.1 Hz), 117.13 (d, *J* = 24.3 Hz), 34.82, 15.06; ^{19}F NMR (376 MHz, DMSO-d_6) δ -111.04; MS (ESI) m/z [M + H]$^+$: 248.1.

3-Cyano-5-fluoro-N-propylbenzamide

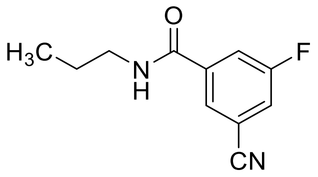

The compound was obtained from 5-cyano-3-fluorobenzoic acid (0.99 g, 6.0 mmol) and aqueous ethylamine solution (3.54 g, 60.0 mmol) following the general procedure D to give 1.21 g (97%) of 3-cyano-5-fluoro-N-propylbenzamide as a white solid. *Rf* = 0.46 (n-Heptane:60%EtOAc); ^{1}H NMR (400 MHz, DMSO-d_6) δ 8.71 (t, J = 5.7 Hz, 1H), 8.14 (s, 1H), 8.07 – 7.93 (m, 2H), 3.24 (q, J = 6.6 Hz, 2H), 1.54 (h, J = 7.3 Hz, 2H), 0.90 (t, J = 7.4 Hz, 3H); ^{13}C NMR (101 MHz, DMSO-d_6) δ 163.44 (d, *J* = 2.3 Hz), 162.02 (d, *J* = 247.7 Hz), 138.61 (d, *J* = 7.3 Hz), 127.80 (d, *J* = 3.2 Hz), 122.12 (d, *J* = 25.4 Hz), 119.93 (d, *J* = 22.9 Hz), 117.66 (d, *J* = 3.1 Hz), 113.50 (d, *J* = 10.0 Hz), 41.73, 22.58, 11.88. ^{19}F NMR (376 MHz, DMSO-d_6) δ -109.91.

3-Fluoro-N-propyl-5-(1,2,4,5-tetrazin-3-yl)benzamide

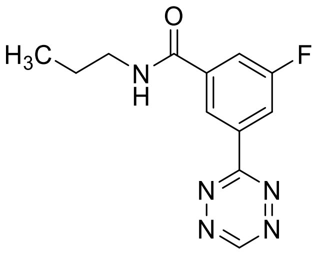

The final compound was obtained from 3-cyano-5-fluoro-N-propylbenzamide (0.82 g, 4.00 mmol) following general procedure A. The crude was purified using flash chromatography (98/2 CH_2Cl_2/MeOH) to afford 0.35 g (32%) of a pink solid. *Rf* = 0.35 (n-Heptane:60%EtOAC); ^{1}H NMR (400 MHz, DMSO-d_6) δ 10.70 (s, 1H), 8.91 – 8.85 (m, 2H), 8.39 (ddd, *J* = 9.3, 2.6, 1.5 Hz, 1H), 8.01 (ddd, *J* = 9.5, 2.6, 1.5 Hz, 1H), 3.32 – 3.24 (m, 3H), 1.58 (q, *J* = 7.2 Hz, 2H), 0.92 (t, *J* = 7.4 Hz, 3H); ^{13}C NMR (101 MHz, DMSO-d_6) δ 164.92, 164.31, 162.87 (d, *J* = 245.6 Hz), 158.91, 138.60 (d, *J* = 6.8 Hz), 134.99, 123.17, 118.66 (d, *J* = 23.0 Hz), 117.13 (d, *J* = 24.1 Hz), 41.72, 22.70, 11.94; ^{19}F NMR (376 MHz, DMSO-d_6) δ -111.05; MS (ESI) m/z [M + H]$^+$: 262.1.

3-(Bromomethyl)-5-fluorobenzonitrile

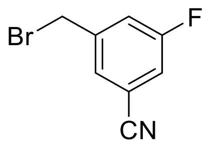

The compound was prepared as previously described by García-Vázquez et al.[7, 8] To a solution of 3-fluoro-5-methylbenzonitrile (2.61 g, 19.24 mmol) and N-bromosuccinimide (5.13 g, 28.86 mmol) in MeCN was added AIBN (1.26 g, 7.69 mmol). The reaction was refluxed for 24 h. The solvent was removed under vacuum and the crude purified by flash chromatography (heptane/EtOAc 95/5) to give 2.10 g (51%) of 3-(bromomethyl)-5-fluorobenzonitrile as a colorless oil. *Rf* = 0.32 (n-Heptane:5%EtOAc); ^{1}H NMR (400 MHz, Chloroform-*d*) δ 7.50 (t, *J* = 1.5 Hz, 1H), 7.38 (dt, *J* = 8.9, 2.0 Hz, 1H), 7.30 (ddd, *J* = 7.9, 2.5, 1.3 Hz, 1H), 4.46 (s, 2H); ^{13}C NMR (101 MHz, Chloroform-*d*) δ 162.12 (d, *J* = 251.3 Hz), 141.93 (d, *J* = 8.0 Hz), 128.52 (d, *J* = 3.4 Hz), 121.06 (d, *J* = 22.0 Hz), 118.97 (d, *J* = 24.7 Hz), 117.07 (d, *J* = 3.3 Hz), 114.26 (d, *J* = 9.9 Hz), 30.32 (d, *J* = 1.9 Hz).

3-(Aminomethyl)-5-fluorobenzonitrile hydrochloride

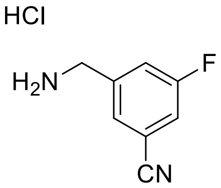

The compound was reported by Battisti et al.[8] The compound was obtained from 3-(bromomethyl)-5-fluorobenzonitrile (1.3 g, 5.7 mmol) and 7N solution of ammonia in MeOH (9 mL) following the general procedure E to give 0.71 g (67%) of 3-(aminomethyl)-5-fluorobenzonitrile hydrochloride as a white solid. ^{1}H NMR (400 MHz, MeOD) δ 7.76 (t, *J* = 1.4 Hz, 1H), 7.73 – 7.62 (m, 2H), 4.26 (s, 2H); ^{13}C NMR (101 MHz, MeOD) δ 163.76 (d, *J* = 249.8 Hz), 138.91 (d, *J* = 8.0 Hz), 130.12 (d, *J* = 3.8 Hz), 122.40 (d, *J* = 22.7 Hz), 120.79 (d, *J* = 25.2 Hz), 118.01, 115.74 (d, *J* = 10.1 Hz), 43.01 (d, *J* = 1.5 Hz).

***Tert*-butyl (3-cyano-5-fluorobenzyl)carbamate**

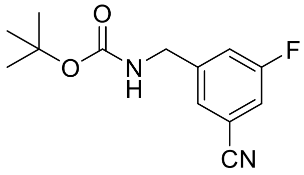

The compound was prepared as previously described by Battisti et al.[8] The compound was obtained from 3-(aminomethyl)-5-fluorobenzonitrile hydrochloride (0.71 g, 3.75 mmol) following the general procedure F. Purification by flash chromatography (Heptane/EtOAc = 85/15) afforded 0.51 g (54%) of the desired compound as an orange solid (mixture of rotamers). *Rf* = 0.26 (Heptane:20%EtOAc); ^{1}H NMR (400 MHz, $CDCl_3$) δ 7.32 (s, 1H), 7.26 – 7.10 (m, 2H), 5.57 (t, *J* = 6.2 Hz, 1H), 4.26 (d, *J*

= 6.2 Hz, 2H), 1.39 (s, 9H); ^{13}C NMR (101 MHz, $CDCl_3$) δ 162.26 (d, *J* = 250.4 Hz), 156.01, 144.21 (d, *J* = 7.5 Hz), 126.53 (d, *J* = 3.2 Hz), 119.09 (d, *J* = 21.7 Hz), 117.56 (d, *J* = 27.6 Hz), 117.45, 113.65 (d, *J* = 9.7 Hz), 79.98, 43.34, 28.23.

Tert-butyl (3-fluoro-5-(1,2,4,5-tetrazin-3-yl)benzyl)carbamate

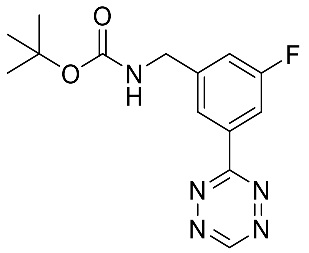

The compound was prepared as previously described by Battisti et al.[8] The compound was obtained from *tert*-butyl (3-cyano-5-fluorobenzyl)carbamate (0.40 g, 1.60 mmol) following the general procedure A. Purification by flash chromatography (95/5 Heptane/EtOAc) afforded 0.16 g (33%) of the desired compound as a red solid (mixture of rotamers). *Rf* = 0.26 (Heptane:20%EtOAc); ^{1}H NMR (400 MHz, $CDCl_3$) δ 10.26 (s, 1H), 8.35 (s, 1H), 8.20 (dt, *J* = 9.2, 2.0 Hz, 1H), 7.35 – 7.28 (m, 1H), 5.21 (t, *J* = 5.7 Hz, 1H), 4.45 (d, *J* = 5.7 Hz, 2H), 1.46 (s, 9H); ^{13}C NMR (101 MHz, $CDCl_3$) δ 165.56 (d, *J* = 3.3 Hz), 163.46 (d, *J* = 248.1 Hz), 158.00, 143.45 (d, *J* = 5.8 Hz), 133.75 (d, *J* = 8.6 Hz), 122.49, 118.86 (d, *J* = 22.2 Hz), 113.91 (d, *J* = 24.3 Hz), 81.41, 80.04, 43.99, 28.35.

(3-Fluoro-5-(1,2,4,5-tetrazin-3-yl)phenyl)methanamine hydrochloride

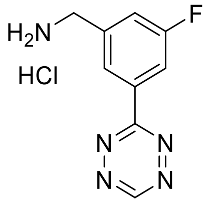

The compound was prepared as previously described by Battisti et al.[8] The compound was obtained from *tert*-butyl 3-fluoro-5-(1,2,4,5-tetrazin-3-yl)benzylcarbamate (0.140 g, 0.46 mmol) following general procedure G to give 0.07 g (63%) of a pink solid. ^{1}H NMR (400 MHz, MeOD) δ 10.33 (s, 1H), 8.48 (d, *J* = 1.6 Hz, 1H), 8.26 (dt, *J* = 9.4, 1.8 Hz, 1H), 7.52 (dt, *J* = 9.0, 1.9 Hz, 1H), 4.23 (s, 2H); ^{13}C NMR (101 MHz, MeOD) δ 165.15 (d, *J* = 3.3 Hz), 163.32 (d, *J* = 247.4 Hz), 158.31, 137.02 (d, *J* = 8.0 Hz), 135.37 (d, *J* = 8.5 Hz), 124.09 (d, *J* = 3.1 Hz), 119.87 (d, *J* = 23.0 Hz), 114.92 (d, *J* = 24.3 Hz), 42.20; MS (ESI) m/z $[M + H]^+$: 206.1.

3-Fluoro-5-((methylamino)methyl)benzonitrile hydrochloride

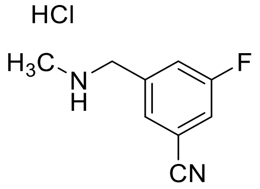

The compound was obtained from 3-(bromomethyl)-5-fluorobenzonitrile (1.0 g, 4.67 mmol) and aqueous methylamine solution (40%, 10 ml) following the general procedure E to give 0.74 g (79%) of 3-fluoro-5-((methylamino)methyl)benzonitrile hydrochloride as a white solid. ^{1}H NMR (400 MHz, DMSO-d_6) δ 9.84 – 9.33 (m, 2H), 8.01 – 7.78 (m, 3H), 4.20 (s, 2H), 2.55 – 2.52 (m, 2H); ^{13}C NMR (101 MHz, DMSO-d_6) δ 161.81 (d, J = 246.9 Hz), 139.61 – 134.15 (m), 130.84, 123.01 (d, J = 22.8 Hz), 120.19 (d, J = 24.4 Hz), 117.77, 113.47, 50.10, 32.43. ^{19}F NMR (376 MHz, DMSO-d_6) δ -109.98.

***Tert*-butyl (3-cyano-5-fluorobenzyl)(methyl)carbamate**

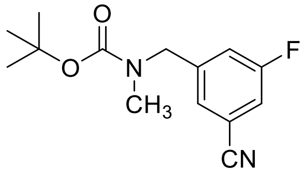

The compound was obtained from 3-fluoro-5-((methylamino)methyl)benzonitrile hydrochloride (0.74 g, 3.69 mmol) following the general procedure F. Purification by flash chromatography (Heptane/EtOAc = 85/15) afforded 0.51 g (54%) of the desired compound as a colorless oil (mixture of rotamers). *Rf* = 0.31 (Heptane:20%EtOAc); ^{1}H NMR (400 MHz, $CDCl_3$) δ 7.31 (s, 1H), 7.25 (d, J = 7.0 Hz, 1H), 7.19 (d, J = 9.1 Hz, 1H), 4.43 (s, 2H), 2.85 (s, 3H), 1.47 (s, 9H); ^{13}C NMR (101 MHz, CDCl3) δ 162.46 (d, J = 250.9 Hz), 156.30, 143.01, 126.73, 119.23, 117.90 (d, J = 24.9 Hz), 117.46, 113.98 (d, J = 9.5 Hz), 80.49, 51.31, 34.48, 28.36; ^{19}F NMR (376 MHz, $CDCl_3$) δ -109.62.

***Tert*-butyl (3-fluoro-5-(1,2,4,5-tetrazin-3-yl)benzyl)(methyl)carbamate**

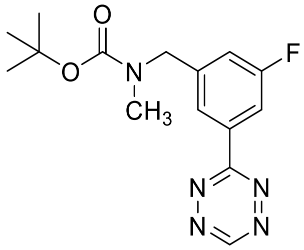

tert-butyl (3-fluoro-5-(1,2,4,5-tetrazin-3-yl)benzyl)(methyl)carbamate

The compound was obtained from *tert*-butyl (3-cyano-5-fluorobenzyl)(methyl)carbamate (0.91 g, 3.44 mmol) following the general procedure A. Purification by flash chromatography (95/5 Heptane/EtOAc) afforded 0.31 g (28%) of the desired compound as a red oil (mixture of rotamers).

Rf = 0.31 (Heptane:20%EtOAc); ^{1}H NMR (400 MHz, $CDCl_3$) δ 10.23 (s, 1H), 8.29 (s, 1H), 8.25 – 8.12 (m, 1H), 7.21 (d, *J* = 8.9 Hz, 1H), 4.52 (s, 2H), 2.89 (s, 3H), 1.48 (s, 9H); ^{13}C NMR (101 MHz, $CDCl_3$) δ 165.56 (d, *J* = 3.2 Hz), 163.55 (d, *J* = 248.2 Hz), 158.02, 156.10 142.53 (d, *J* = 6.7 Hz), 133.81 (d, *J* = 8.6 Hz), 122.79 (d, *J* = 2.7 Hz), 118.88, 113.98 (d, *J* = 24.3 Hz), 80.26, 51.65, 34.37, 28.39; ^{19}F NMR (376 MHz, $CDCl_3$) δ -110.71.

1-(3-Fluoro-5-(1,2,4,5-tetrazin-3-yl)phenyl)-N-methylmethanamine hydrochloride

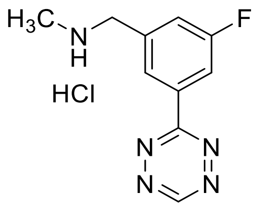

The compound was obtained from *tert*-butyl (3-fluoro-5-(1,2,4,5-tetrazin-3-yl)benzyl)(methyl)carbamate (0.22 g, 0.69 mmol) following general procedure G to give 0.17 g (96%) of a pink solid. ^{1}H NMR (400 MHz, MeOD) δ 10.33 (s, 1H), 8.37 – 8.10 (m, 1H), 7.54 (dt, *J* = 8.9, 2.1 Hz, 1H), 4.29 (s, 2H), 2.71 (s, 3H); ^{13}C NMR (101 MHz, MeOD) δ 165.08 (d, *J* = 3.2 Hz), 163.33 (d, *J* = 247.6 Hz), 158.34, 135.54 (d, *J* = 8.7 Hz), 135.10 (d, *J* = 7.8 Hz), 124.92 (d, *J* = 3.1 Hz), 120.62 (d, *J* = 22.8 Hz), 115.45 (d, *J* = 24.4 Hz), 48.23, 32.03; ^{19}F NMR (376 MHz, MeOD) δ -111.85; MS (ESI) m/z $[M + H]^+$: 220.1.

3-((Ethylamino)methyl)-5-fluorobenzonitrile hydrochloride

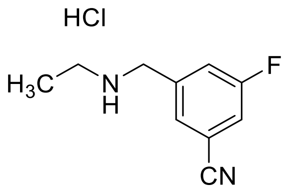

The compound was obtained from 3-(bromomethyl)-5-fluorobenzonitrile (1.0 g, 4.67 mmol) and aqueous ethylamine solution (70%, 10 ml) following the general procedure E to give 0.81 g (81%) of 3-fluoro-5-((ethylamino)methyl)benzonitrile hydrochloride as a white solid. ^{1}H NMR (400 MHz, DMSO-d_6) δ 9.66 (s, 2H), 9.00 – 6.92 (m, 2H), 4.20 (s, 2H), 2.93 (s, 2H), 1.25 (t, *J* = 7.2 Hz, 3H); ^{13}C NMR (101 MHz, DMSO-d_6) δ 161.78 (d, *J* = 247.2 Hz), 137.12 (d, *J* = 8.5 Hz), 130.88 (d, *J* = 3.4 Hz), 123.05 (d, *J* = 22.5 Hz), 120.06 (d, *J* = 25.3 Hz), 117.78 (d, *J* = 3.4 Hz), 113.33 (d, *J* = 10.4 Hz), 48.40, 42.23, 11.32; ^{19}F NMR (376 MHz, DMSO-d_6) δ -110.11.

***Tert*-butyl (3-cyano-5-fluorobenzyl)(ethyl)carbamate**

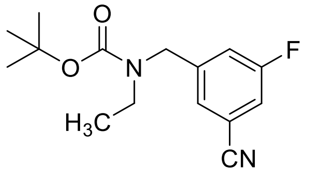

The compound was obtained from 3-fluoro-5-((ethylamino)methyl)benzonitrile hydrochloride (0.75 g, 3.49 mmol) following the general procedure F. Purification by flash chromatography (Heptane/EtOAc = 85/15) afforded 0.96 g (99%) of the desired compound as a colorless oil (mixture of rotamers). *Rf* = 0.37 (Heptane:20%EtOAc); ^{1}H NMR (400 MHz, $CDCl_3$) δ 7.32 (s, 1H), 7.26 – 7.07 (m, 2H), 4.42 (s, 2H), 3.24 (s, 2H), 1.47 – 1.37 (m, 9H), 1.09 (t, *J* = 7.1 Hz, 3H); ^{13}C NMR (101 MHz, $CDCl_3$) δ 162.41 (d, *J* = 250.6 Hz), 155.77, 143.75, 126.64, 117.75 (d, *J* = 24.9 Hz), 117.52, 113.91, 113.81, 80.34, 49.17, 42.16, 28.37, 27.41; ^{19}F NMR (376 MHz, $CDCl_3$) δ -109.74.

Tert-butyl (3-fluoro-5-(1,2,4,5-tetrazin-3-yl)benzyl)(ethyl)carbamate

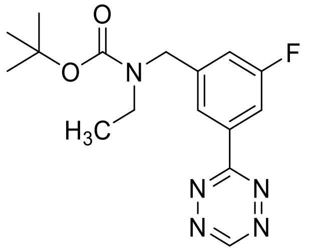

The compound was obtained from *tert*-butyl (3-cyano-5-fluorobenzyl)(ethyl)carbamate (0.92 g, 3.30 mmol) following the general procedure A. Purification by flash chromatography (95/5 Heptane/EtOAc) afforded 0.25 g (23%) of the desired compound as a red oil (mixture of rotamers). *Rf* = 0.36 (Heptane:20%EtOAc); ^{1}H NMR (400 MHz, $CDCl_3$) δ 10.24 (s, 1H), 8.31 (s, 1H), 8.20 (dt, *J* = 9.5, 2.1 Hz, 1H), 7.25 (d, *J* = 10.3 Hz, 1H), 4.53 (s, 2H), 3.29 (s, 2H), 1.48 (s, 9H), 1.12 (t, *J* = 7.1 Hz, 3H); ^{13}C NMR (101 MHz, $CDCl_3$) δ 165.62 (d, *J* = 3.4 Hz), 163.54 (d, *J* = 248.0 Hz), 158.01, 155.34, 143.26, 133.69 (d, *J* = 8.6 Hz), 122.69, 118.90, 113.88 (d, *J* = 24.3 Hz), 80.14, 49.53, 41.99, 28.42, 13.37; ^{19}F NMR (376 MHz, $CDCl_3$) δ -110.83.

N-(3-fluoro-5-(1,2,4,5-tetrazin-3-yl)benzyl)ethanamine hydrochloride

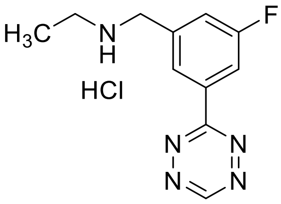

The compound was obtained from *tert*-butyl *(*3-fluoro-5-(1,2,4,5-tetrazin-3-yl)benzyl)(ethyl)carbamate (0.12 g, 0.36 mmol) following general procedure G to give 0.07 g (72%) of a pink solid. ^{1}H NMR (400 MHz, DMSO-d_6) δ 10.70 (s, 1H), 9.51 (s, 2H), 8.58 (s, 1H), 8.29 (d, *J*

= 9.4 Hz, 1H), 7.91 (d, *J* = 9.5 Hz, 1H), 4.34 (s, 2H), 3.00 (d, *J* = 9.8 Hz, 2H), 1.27 (t, *J* = 7.2 Hz, 3H); ^{13}C NMR (101 MHz, DMSO-d_6) δ 164.90 (d, *J* = 3.2 Hz), 162.78 (d, *J* = 245.3 Hz), 158.95, 136.83 (d, *J* = 8.1 Hz), 134.98 (d, *J* = 8.8 Hz), 126.01 (d, *J* = 3.0 Hz), 121.75 (d, *J* = 22.6 Hz), 115.13 (d, *J* = 24.2 Hz), 49.04, 42.34, 11.37; ^{19}F NMR (376 MHz, DMSO-d_6) δ -111.00; MS (ESI) m/z [M + H]$^+$: 234.1.

3-((Propylamino)methyl)-5-fluorobenzonitrile hydrochloride

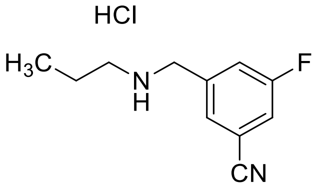

The compound was obtained from 3-(bromomethyl)-5-fluorobenzonitrile (1.0 g, 4.67 mmol) and propylamine (3.8 mL, 46.72 mmol) following the general procedure E to give 0.92 g (86%) of 3-fluoro-5-((propylamino)methyl)benzonitrile hydrochloride as a white solid. ^{1}H NMR (400 MHz, DMSO-d_6) δ 9.68 (s, 2H), 8.69 – 7.21 (m, 3H), 4.20 (s, 2H), 2.83 (t, *J* = 7.8 Hz, 2H), 1.70 (h, *J* = 7.5 Hz, 2H), 0.91 (t, *J* = 7.4 Hz, 3H); ^{13}C NMR (101 MHz, DMSO-d_6) δ 161.76 (d, *J* = 247.2 Hz), 137.04 (d, *J* = 8.6 Hz), 130.97 (d, *J* = 3.3 Hz), 123.15 (d, *J* = 22.5 Hz), 120.06 (d, *J* = 25.3 Hz), 117.80 (d, *J* = 3.3 Hz), 113.30 (d, *J* = 10.5 Hz), 48.80, 48.60, 19.35, 11.45; ^{19}F NMR (376 MHz, DMSO-d_6) δ -110.14.

***Tert*-butyl (3-cyano-5-fluorobenzyl)(propyl)carbamate**

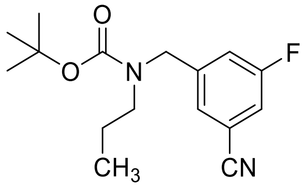

The compound was obtained from 3-fluoro-5-((propylamino)methyl)benzonitrile hydrochloride (0.85 g, 3.72 mmol) following the general procedure F. Purification by flash chromatography (Heptane/EtOAc = 85/15) afforded 1.05 g (97%) of the desired compound as a colorless oil (mixture of rotamers). *Rf* = 0.42 (Heptane:20%EtOAc); ^{1}H NMR (400 MHz, $CDCl_3$) δ 7.31 (s, 1H), 7.26 – 7.09 (m, 2H), 4.43 (s, 2H), 3.14 (s, 2H), 1.48 (br s, 11H), 0.87 (t, *J* = 7.4 Hz, 3H); ^{13}C NMR (101 MHz, $CDCl_3$) δ 162.41 (d, *J* = 250.6 Hz), 155.97, 143.78, 131.88 – 124.65 (m), 119.14, 117.73 (d, *J* = 25.0 Hz), 113.85 (d, *J* = 9.6 Hz), 80.33, 49.45, 49.11, 28.35, 21.53, 11.20; ^{19}F NMR (376 MHz, $CDCl_3$) δ -109.75.

***Tert*-butyl (3-fluoro-5-(1,2,4,5-tetrazin-3-yl)benzyl)(propyl)carbamate**

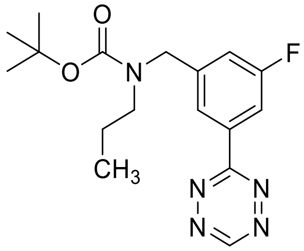

The compound was obtained from *tert*-butyl (3-cyano-5-fluorobenzyl)(propyl)carbamate (1.05 g, 3.59 mmol) following the general procedure A. Purification by flash chromatography (95/5 Heptane/EtOAc) afforded 0.36 g (29%) of the desired compound as a red oil (mixture of rotamers). *Rf* = 0.42 (Heptane:20%EtOAc); ^{1}H NMR (400 MHz, $CDCl_3$) δ 10.23 (s, 1H), 8.29 (s, 1H), 8.19 (d, *J* = 9.3 Hz, 1H), 7.23 (d, *J* = 8.9 Hz, 1H), 4.53 (s, 2H), 3.18 (s, 2H), 2.03 – 1.20 (m, 11H), 0.86 (td, *J* = 7.5, 4.0 Hz, 3H); ^{13}C NMR (101 MHz, $CDCl_3$) δ 165.60 (d, *J* = 3.4 Hz), 163.52 (d, *J* = 248.2 Hz), 158.00, 156.12, 143.19, 133.68 (d, *J* = 8.5 Hz), 122.64, 118.83, 113.83 (d, *J* = 24.2 Hz), 80.06, 49.74, 48.94, 28.38, 21.48, 11.23; ^{19}F NMR (376 MHz, $CDCl_3$) δ -110.85.

N-(3-Fluoro-5-(1,2,4,5-tetrazin-3-yl)benzyl)propylamine hydrochloride

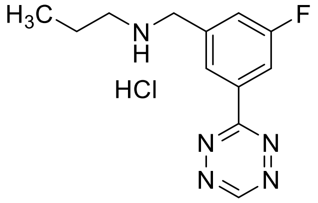

The compound was obtained from *tert*-butyl *(*3-fluoro-5-(1,2,4,5-tetrazin-3-yl)benzyl)(propyl)carbamate (0.30 g, 0.86 mmol) following general procedure G to give 0.22 g (90%) of a pink solid. ^{1}H NMR (400 MHz, DMSO-d_6) δ 10.70 (s, 1H), 9.53 (s, 2H), 8.59 (s, 1H), 8.49 – 8.15 (m, 1H), 7.93 (dt, *J* = 9.4, 2.0 Hz, 1H), 4.34 (t, *J* = 5.8 Hz, H), 2.90 (dd, *J* = 9.7, 5.1 Hz, H), 1.71 (q, *J* = 7.7 Hz, 2H), 0.93 (t, *J* = 7.5 Hz, 3H); ^{13}C NMR (101 MHz, DMSO-d_6) δ 164.89, 162.76 (d, *J* = 245.7 Hz), 158.94, 136.74 (d, *J* = 8.1 Hz), 134.95 (d, *J* = 8.6 Hz), 126.12 (d, *J* = 2.9 Hz), 121.85 (d, *J* = 22.5 Hz), 115.14 (d, *J* = 24.1 Hz), 49.44, 48.70, 19.39, 11.48; ^{19}F NMR (376 MHz, DMSO-d_6) δ -111.02; MS (ESI) m/z $[M + H]^+$: 248.1.

Synthesis of precursors

3-(3-Iodophenyl)-1,2,4,5-tetrazine

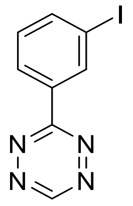

The compound was prepared as previously described by García-Vázquez et al.[7] The tetrazine was obtained from 3-iodobenzonitrile (458 mg, 4 mmol) following general procedure A. The crude was purified using flash chromatography (90/10 n-Heptane/EtOAc) to yield 0.19 g (33%) of a pink solid. *Rf* = 0.36 (n-Heptane:10%EtOAC); ^{1}H NMR (400 MHz, $CDCl_3$) δ 10.25 (s, 1H), 9.00 (t, *J* = 1.7 Hz, 1H), 8.60 (ddd, *J* = 7.9, 1.7, 1.1 Hz, 1H), 7.99 (ddd, *J* = 7.9, 1.8, 1.0 Hz, 1H), 7.35 (t, *J* = 7.9 Hz, 1H); ^{13}C NMR (101 MHz, $CDCl_3$) δ 165.47, 158.17, 142.11, 137.21, 133.63, 131.08, 127.51, 95.02.

3-(3-(Trimethylstannyl)phenyl)-1,2,4,5-tetrazine

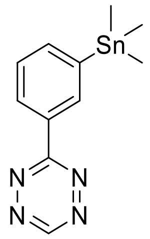

The compound was prepared as previously described by García-Vázquez et al.[7] The compound was obtained from 3-(3-iodophenyl)-1,2,4,5-tetrazine (0.05 g, 0.17 mmol) of the starting material, following the general procedure H. The crude was purified using flash chromatography (90/10 n-Heptane/EtOAc) to yield 0.28 g (58%) of a pink solid. *Rf* = 0.30 (n-Heptane:10%EtOAc); ^{1}H NMR (400Hz, Chloroform-*d*) δ 10.14 (s,1H), 8.67 (s, 1H), 8.48 (s, J=7.9 Hz, 1H) 7.72 (d, J=7.5 Hz, 1H) 7.50 (t, J=7.9, 15.8 Hz, 1H) 0.30 (s, 9H); ^{13}C NMR (101 MHz, Chloroform-*d*) δ 166.83, 157.75, 144.16, 140.60, 135.48, 130.96, 128.74 128.17, -9.38.; MS (ESI) m/z $[M + H]^{+}$: 323.0

3-(3-Iodo-5-methylphenyl)-1,2,4,5-tetrazine

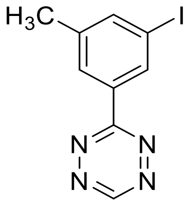

The compound was prepared as previously described by García-Vázquezet al.[7] The tetrazine was obtained from 3-iodo-5-methylbenzonitrile (0.97 g, 4.00 mmol) following general procedure A. The crude was purified using flash chromatography (95/5 n-Heptane/EtOAc) to yield after recrystallization with n-Heptane 0.27 g (22%) as a red solid. *Rf* = 0.45 (n-Heptane:20%EtOAc ^{1}H NMR (400 MHz, $CDCl_3$) δ 10.22 (s, 1H), 8.83 – 8.67 (m, 1H), 8.44 – 8.28 (m, 1H), 7.87 – 7.74 (m, 1H), 2.43 (s, 2H); ^{13}C NMR (151 MHz, $CDCl_3$) δ 165.49, 158.09, 142.71, 141.41, 134.34, 133.30, 128.22, 94.99, 21.21.

3-(3-Methyl-5-(trimethylstannyl)phenyl)-1,2,4,5-tetrazine

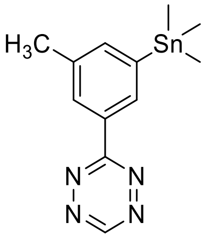

The compound was obtained from 3-(3-iodo-5-methylphenyl)-1,2,4,5-tetrazine (0.05 g, 0.17 mmol) following the general procedure H. The crude was purified using flash chromatography (90/10 n-Heptane/EtOAc) to yield 0.25 g (27%) of a pink solid. *Rf* = 0.34 (n-Heptane:20%EtOAc); ^{1}H NMR (400 MHz, $CDCl_3$) δ 10.20 (s, 1H), 8.65 – 8.50 (m, 1H), 8.48 – 8.33 (m, 1H), 7.73 – 7.43 (m, 1H), 2.48 (s, 3H), 0.36 (s, 9H); ^{13}C NMR (151 MHz, $CDCl_3$) δ 167.05, 157.86, 144.08, 141.58, 138.56, 132.79, 131.00, 128.91, 21.58, -9.25.; MS (ESI) m/z $[M + H]^+$: 337.1.

3-Iodo-5-methoxybenzonitrile

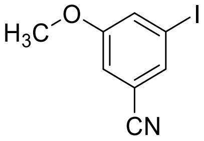

Concentrated. HCl (3 mL) was added to a solution of aniline (1.00 g, 6.75 mmol) in water (3 mL) at 0 °C. To this was added a chilled solution of sodium nitrite (0.84 g, 12.15 mmol) in water (4 mL), dropwise, with vigorous mechanical stirring. Stirring was continued at 0°C for 15 min. after the addition was complete, and then a solution of potassium iodide (2.24 g, 13.50 mmol) in water (4 mL) was added carefully. The cooling bath was removed, and the reaction heated to reflux. When the production of purple vapor ceased, the mixture was cooled to rt and extracted with DCM (3×20 mL). The combined organic extracts were dried ($MgSO_4$), filtered, and concentrated under reduced pressure. Purification by flash chromatography (90/10 Heptane/EtOAc) afforded 0.60 g (34%) of a white solid. *Rf* = 0.34 (n-Heptane:20%EtOAc); ^{1}H NMR (400 MHz, $CDCl_3$) δ 7.48 (t, *J* = 1.4 Hz, 1H), 7.40 (dd, *J* = 2.4, 1.4 Hz, 1H), 7.04 (dd, *J* = 2.4, 1.4 Hz, 1H), 3.75 (s, 3H); ^{13}C NMR (101 MHz, $CDCl_3$) δ 159.92, 132.68, 128.19, 116.85, 114.60, 94.14, 55.83.

3-(3-Iodo-5-methoxyphenyl)-1,2,4,5-tetrazine

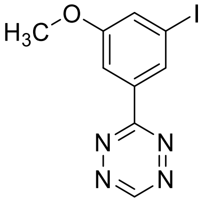

The compound was obtained from 3-iodo-5-methoxylbenzonitrile (0.52 g, 2.00 mmol) following general procedure A. The crude was purified using flash chromatography (85/15 n-Heptane/EtOAc)

and recrystallized from n-Heptane 0.19 g (30%) of a red solid. *Rf* = 0.25 (n-Heptane:20%EtOAc); ^{1}H NMR (400 MHz, $CDCl_3$) δ 10.17 (s, 1H), 8.49 (t, *J* = 1.4 Hz, 1H), 8.03 (dd, *J* = 2.4, 1.4 Hz, 1H), 7.44 (dd, *J* = 2.5, 1.5 Hz, 1H), 3.83 (s, 3H); ^{13}C NMR (101 MHz, $CDCl_3$) δ 165.13, 160.72, 158.02, 134.10, 129.53, 128.40, 112.47, 94.96, 55.80.

3-(3-Methoxy-5-(trimethylstannyl)phenyl)-1,2,4,5-tetrazine

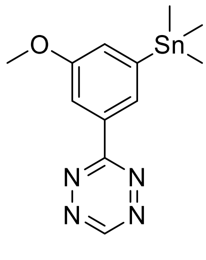

The compound was obtained from 3-(3-iodo-5-methoxyphenyl)-1,2,4,5-tetrazine (0.055 g (0.17 mmol) following the general procedure H. The crude was purified using flash chromatography (90/10 n-Heptane/EtOAc) to yield 0.040 g (65%) of UB-229 as a purple solid. *Rf* = 0.41 (n-Heptane:20%EtOAc); ^{1}H NMR (400 MHz, Chloroform-*d*) δ 10.21 (s, 1H), 8.35 (d, *J* = 1.5 Hz, 1H), 8.07 (dd, *J* = 2.7, 1.6 Hz, 1H), 7.32 (d, *J* = 2.7 Hz, 1H), 3.94 (s, 3H), 0.37 (s, 9H); ^{13}C NMR (101 MHz, Chloroform-*d*) δ 166.64, 157.77, 145.64, 132.10, 127.94, 127.56, 111.71, 55.45, -9.36; MS (ESI) m/z $[M + H]^+$: 353.0.

3-Hydroxy-5-iodobenzonitrile

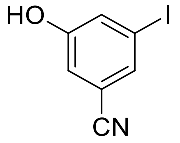

3-Iodo-5-methoxybenzonitrile (1.10 g, 4.26 mmol) was stirred in dry CH_2Cl_2 (20 mL) at -78 °C under N_2. A solution of BBr_3 (25.47 mL, 1 M in CH_2Cl_2, 25.47 mmol) was added over 20 minutes. After 5 minutes from the addition, the solution was allowed to warm to 20 °C and was stirred for 2 days. The solvent was then removed under reduced pressure. Purification by flash chromatography afforded 0.86 g (83%) of a white solid. *Rf* = 0.21 (n-Heptane:20%EtOAc); ^{1}H NMR (400 MHz, MeOD) δ 7.36 (t, *J* = 1.5 Hz, 1H), 7.33 (t, *J* = 1.9 Hz, 1H), 6.95 (dd, *J* = 2.3, 1.4 Hz, 1H); ^{13}C NMR (101 MHz, MeOD) δ 158.44, 130.89, 129.24, 117.85, 116.73, 114.06, 93.66.

3-Ethoxy-5-iodobenzonitrile

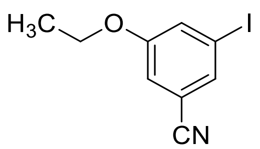

The compound was obtained from 3-hydroxy-5-iodobenzonitrile (0.60 g, 2.45 mmol) and iodoethane (0.57 g, 3.67 mmol) following the general procedure B. The crude was purified using flash chromatography (85/15 n-Heptane/EtOAc) to give 0.67 g (99%) of 3-ethoxy-5-iodobenzonitrile as a white solid. *Rf* = 0.38 (n-Heptane:20%EtOAc); ^{1}H NMR (400 MHz, $CDCl_3$) δ 7.51 (t, *J* = 1.4 Hz, 1H), 7.44 (dd, *J* = 2.5, 1.4 Hz, 1H), 7.08 (dd, *J* = 2.4, 1.3 Hz, 1H), 4.02 (q, *J* = 7.0 Hz, 2H), 1.41 (t, *J* = 7.0 Hz, 3H); ^{13}C NMR (101 MHz, $CDCl_3$) δ 159.30, 132.44, 128.67, 117.27, 115.79 (d, *J* = 252.3 Hz), 94.15, 64.38, 14.51.

3-(3-Ethoxy-5-iodophenyl)-1,2,4,5-tetrazine

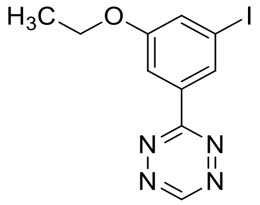

The tetrazine was obtained from 3-ethoxy-5-iodobenzonitrile (0.66 g, 2.41 mmol) following general procedure A. The crude was purified using flash chromatography (85/15 n-Heptane/EtOAc) and recrystallized from n-Heptane to give 0.17 g (21%) of a red solid. *Rf* = 0.37 (n-Heptane:20%EtOAc); ^{1}H NMR (400 MHz, $CDCl_3$) δ 10.15 (s, 1H), 8.44 (s, 1H), 8.12 – 7.89 (m, 1H), 7.42 (s, 1H), 4.05 (q, *J* = 7.0 Hz, 2H), 1.38 (t, *J* = 6.9 Hz, 3H); ^{13}C NMR (101 MHz, $CDCl_3$) δ 165.14, 160.07, 158.00, 134.04, 129.32, 128.88, 112.95, 94.97, 64.21, 14.67.

3-(3-Ethoxy-5-(trimethylstannyl)phenyl)-1,2,4,5-tetrazine

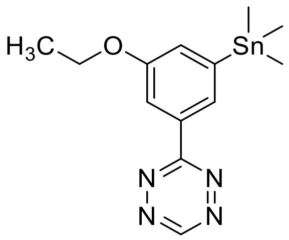

The compound was obtained from 3-(3-ethoxy-5-iodophenyl)-1,2,4,5-tetrazine **(**0.057 g, 0.17 mmol) of the starting material, following the general procedure H. The crude was purified using flash chromatography (90/10 n-Heptane/EtOAc) to yield 0.042 g (66%) of a purple solid. *Rf* = 0.51 (n-Heptane:20%EtOAc); ^{1}H NMR (400 MHz, $CDCl_3$) δ 10.20 (s, 1H), 8.33 (dd, *J* = 1.6, 0.7 Hz, 1H), 8.06 (dd, *J* = 2.7, 1.6 Hz, 1H), 7.32 (dd, *J* = 2.6, 0.7 Hz, 1H), 4.18 (q, *J* = 7.0 Hz, 2H), 1.48 (t, *J* = 7.0

Hz, 3H), 0.36 (s, 9H); ^{13}C NMR (101 MHz, $CDCl_3$) δ 166.68, 159.08, 157.74, 145.56, 132.04, 128.14, 127.81, 112.23, 63.69, 14.82, -9.37; MS (ESI) m/z $[M + H]^+$: 367.1.

3-Iodo-5-propoxybenzonitrile

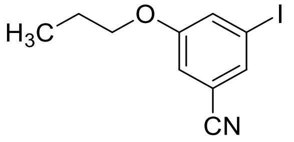

The compound was obtained from 3-hydroxy-5-iodobenzonitrile (0.22 g, 0.89 mmol) and 1-bromopropane (0.16 g, 1.35 mmol) following the general procedure B. The crude was purified using flash chromatography (85/15 n-Heptane/EtOAc) to give 0.25 g (97%) of 3-iodo-5-propoxybenzonitrile a white solid. *Rf* = 0.52 (n-Heptane:20%EtOAc); ^{1}H NMR (400 MHz, $CDCl_3$) δ 7.44 (s, 1H), 7.38 (s, 1H), 7.02 (d, *J* = 1.1 Hz, 1H), 3.83 (t, *J* = 6.5 Hz, 2H), 1.73 (h, *J* = 7.1 Hz, 2H), 0.96 (t, *J* = 7.4 Hz, 3H); ^{13}C NMR (101 MHz, $CDCl_3$) δ 159.49, 132.39, 128.68, 117.27, 114.51, 94.14, 70.28, 10.39.

3-(3-Iodo-5-propoxyphenyl)-1,2,4,5-tetrazine

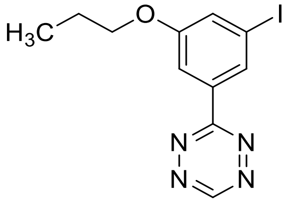

The tetrazine was obtained from 3-iodo-5-propoxybenzonitrile **(**0.27 g, 0.94 mmol) following general procedure A. The crude was purified using flash chromatography (85/15 n-Heptane/EtOAc) and recrystallized from n-Heptane to give 0.07 g (22%) of a red oil. *Rf* = 0.51 (n-Heptane:20%EtOAc); ^{1}H NMR (400 MHz, $CDCl_3$) δ 10.16 (s, 1H), 8.48 (t, *J* = 1.5 Hz, 1H), 8.04 (dd, *J* = 2.4, 1.5 Hz, 1H), 7.46 (dd, *J* = 2.4, 1.5 Hz, 1H), 3.95 (t, *J* = 6.5 Hz, 2H), 1.78 (h, *J* = 7.1 Hz, 2H), 1.00 (t, *J* = 7.4 Hz, 3H); ^{13}C NMR (101 MHz, $CDCl_3$) δ 165.20, 160.31, 158.00, 134.04, 129.32, 128.93, 113.02, 94.95, 70.17, 22.45, 10.46.

3-(3-Propoxy-5-(trimethylstannyl)phenyl)-1,2,4,5-tetrazine

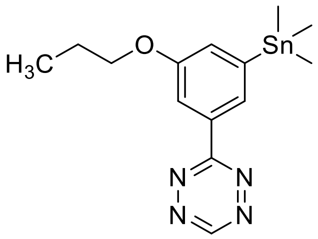

The compound was obtained from 3-(3-iodo-5-propoxyphenyl)-1,2,4,5-tetrazine (0.052 g, 0.15 mmol) of the starting material, following the general procedure H. The crude was purified using flash chromatography (90/10 n-Heptane/EtOAc) to yield 0.030 g (51%) of a purple oil. *Rf* = 0.53 (n-Heptane:20%EtOAc); ^{1}H NMR (600 MHz, $CDCl_3$) δ 10.20 (s, 1H), 8.38 – 8.26 (m, 1H), 8.06 (dd, *J* = 2.7, 1.6 Hz, 1H), 7.32 (d, *J* = 2.8 Hz, 1H), 4.06 (t, *J* = 6.6 Hz, 2H), 1.91 – 1.84 (m, 2H), 1.09 (t, *J* = 7.4 Hz, 3H), 0.36 (s, 9H); ^{13}C NMR (151 MHz, $CDCl_3$) δ 166.69, 159.29, 157.74, 145.53, 132.02, 128.08, 127.75, 112.31, 69.72, 22.62, 10.56, -9.36: MS (ESI) m/z $[M + H]^+$: 381.1.

3-(Benzyloxy)-5-iodobenzonitrile

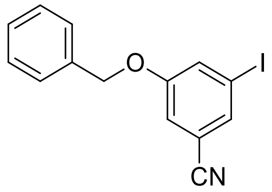

The compound was obtained from 3-hydroxy-5-iodobenzonitrile (0.23 g, 0.90 mmol) and benzylbromide (0.24 g, 1.35 mmol) following the general procedure B. The crude was purified using flash chromatography (85/15 n-Heptane/EtOAc) to give 0.29 g (97%) of 3-(benzyloxy)-5-iodobenzonitrile a white solid. *Rf* = 0.54 (n-Heptane:20%EtOAc); ^{1}H NMR (400 MHz, $CDCl_3$) δ 7.58 – 7.52 (m, 2H), 7.51 – 7.31 (m, 5H), 7.17 (dd, *J* = 2.3, 1.4 Hz, 1H), 5.06 (s, 2H); ^{13}C NMR (101 MHz, $CDCl_3$) δ 159.02, 135.26, 132.97, 129.15, 128.84, 128.60, 127.54, 117.59, 116.98, 114.64, 94.22, 70.68.

3-(3-(Benzyloxy)-5-iodophenyl)-1,2,4,5-tetrazine

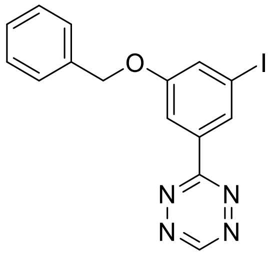

The tetrazine was obtained from 3-(benzyloxy)-5-iodobenzonitrile (0.29 g, 0.86 mmol) following general procedure A. The crude was purified using flash chromatography (85/15 n-Heptane/EtOAc) and recrystallized from n-Heptane to give 0.11 g (33%) of a red oil. *Rf* = 0.48 (n-Heptane:20%EtOAc); ^{1}H NMR (400 MHz, $CDCl_3$) δ 10.24 (s, 1H), 8.60 (t, *J* = 1.5 Hz, 1H), 8.22 (dd, *J* = 2.5, 1.4 Hz, 1H), 7.63 (dd, *J* = 2.5, 1.5 Hz, 1H), 7.49 – 7.32 (m, 5H), 5.17 (s, 2H); ^{13}C NMR (101 MHz, $CDCl_3$) δ 165.14, 159.84, 158.03, 135.84, 134.16, 129.84, 129.31, 128.73, 128.35, 127.58, 113.31, 95.00, 70.53.

3-(3-(Benzyloxy)-5-(trimethylstannyl)phenyl)-1,2,4,5-tetrazine

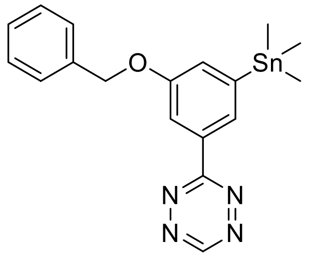

The compound was obtained from 3-(3-(benzyloxy)-5-iodophenyl)-1,2,4,5-tetrazine (0.06 g, 0.15 mmol) of the starting material, following the general procedure H. The crude was purified using flash chromatography (90/10 n-Heptane/EtOAc) to yield 0.045 g (68%) of a purple oil. *Rf* = 0.54 (n-Heptane:20%EtOAc); ^{1}H NMR (400 MHz, $CDCl_3$) δ 10.21 (s, 1H), 8.40 – 8.32 (m, 1H), 8.17 (dd, *J* = 2.7, 1.6 Hz, 1H), 7.50 (d, *J* = 7.0 Hz, 2H), 7.43 – 7.37 (m, 3H), 7.36 (d, *J* = 7.3 Hz, 1H), 5.20 (s, 2H), 0.36 (s, 9H); ^{13}C NMR (101 MHz, $CDCl_3$) δ 166.62, 158.91, 157.77, 145.76, 136.56, 132.12, 128.66, 128.34, 128.19, 128.15, 127.68, 112.69, 70.20, -9.35; MS (ESI) m/z $[M + H]^+$:429.1

3-Amino-5-iodobenzonitrile

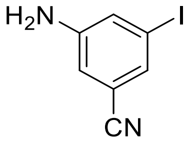

The compound was prepared as previously described by García-Vázquez et al.[7] To a solution of 3-iodo-5-nitrobenzonitrile (0.500 g, 1.82 mmol) and Zn (0.58 g, 8.87 mmol) in MeOH (20 mL) was added dropwise 1 mL of acetic acid. The reaction was stirred at room temperature for 2 h and then concentrated under reduced pressure. Purification by flash chromatography afforded 0.250 g (56%) of a white solid. *Rf* = 0.22 (n-Heptane:20%EtOAc); ^{1}H NMR (400 MHz, $CDCl_3$) δ 7.24 (d, *J* = 1.4 Hz, 1H), 7.15 (t, *J* = 1.9 Hz, 1H), 6.81 – 6.73 (m, 1H), 3.81 (s, 2H); ^{13}C NMR (101 MHz, $CDCl_3$) δ 147.74, 129.88, 127.63, 117.39, 116.80, 114.45, 94.45.

N-(5-Cyano-3-iodophenyl)acetamide

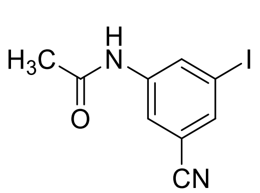

The compound was prepared as previously described by García-Vázquezet al.[7] The compound was obtained from 3-amino-5-iodobenzonitrile (0.20 g, 0.81 mmol) and acetic anhydride (0.1 mL, 1.15 mmol) following the general procedure C. The crude was purified using flash chromatography (70/30

n-Heptane/EtOAc) to afford 0.21 (90%) of a white solid. *Rf* = 0.29 (n-Heptane:40%EtOAc); ^{1}H NMR (400 MHz, MeOD) δ 8.13 (t, *J* = 1.8 Hz, 1H), 7.87 (t, *J* = 1.7 Hz, 1H), 7.66 (t, *J* = 1.5 Hz, 1H), 2.04 (s, 3H); ^{13}C NMR (101 MHz, MeOD) δ 170.51, 140.51, 134.84, 132.16, 121.42, 116.54, 113.80, 93.18, 22.51.

N-(3-Iodo-5-(1,2,4,5-tetrazin-3-yl)phenyl)acetamide

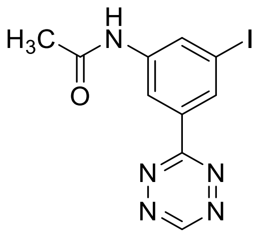

The compound was prepared as previously described by García-Vázquezet al.[7] The compound was obtained from N-(5-cyano-3-iodophenyl)acetamide (0.18 g, 0.63 mmol) following general procedure A. The crude was purified using flash chromatography (60/40 n-Heptane/EtOAc) to yield 0.055 g (26%) of a red solid. *Rf* = 0.21 (n-Heptane:40%EtOAc); ^{1}H NMR (600 MHz, DMSO-d_6) δ 10.63 (s, 1H), 10.35 (s, 1H), 8.73 (t, *J* = 1.7 Hz, 1H), 8.44 (t, *J* = 1.5 Hz, 1H), 8.38 (t, *J* = 1.8 Hz, 1H), 2.11 (s, 3H); ^{13}C NMR (151 MHz, DMSO-d_6) δ 169.44, 164.69, 158.81, 141.92, 134.71, 131.04, 130.64, 117.61, 95.93, 24.59.

N-(3-(1,2,4,5-tetrazin-3-yl)-5-(trimethylstannyl)phenyl)acetamide

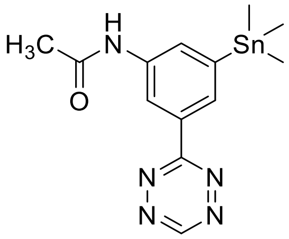

The compound was prepared as previously described by García-Vázquezet al.[7] The compound was obtained from N-(3-iodo-5-(1,2,4,5-tetrazin-3-yl)phenyl)acetamide (0.055 g, 0.17 mmol) of the starting material, following the general procedure H. The crude was purified using flash chromatography (70/30 n-Heptane/EtOAc) to yield 0.025 g (41%) as a purple oil. *Rf* = 0.35 (n-Heptane:50%EtOAc); ^{1}H NMR (400 MHz, $CDCl_3$) δ 10.20 (s, 1H), 8.56 (t, *J* = 2.1 Hz, 1H), 8.44 (s, 1H), 8.03 (d, *J* = 2.2 Hz, 1H), 7.63 (s, 1H), 2.24 (s, 3H), 0.36 (s, 9H); ^{13}C NMR (101 MHz, $CDCl_3$) δ 168.68, 166.46, 157.80, 145.49, 138.40, 131.68, 131.52, 131.18, 119.43, 24.60, -9.30; MS (ESI) m/z $[M + H]^+$: 380.0.

N-(3-Cyano-5-iodophenyl)propionamide

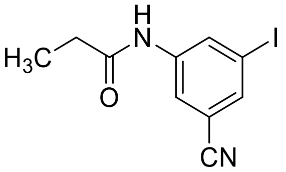

The compound was obtained from 3-amino-5-iodobenzonitrile (0.33 g, 1.35 mmol) and propionic anhydride (0.24 mL, 1.40 mmol) following the general procedure C. The crude was purified using flash chromatography (70/30 n-Heptane/EtOAc) to afford 0.37 (91%) of a white solid. *Rf* = 0.22 (n-Heptane:20%EtOAc); ^{1}H NMR (400 MHz, $CDCl_3$) δ 8.22 – 8.03 (m, 2H), 7.92 (t, *J* = 1.7 Hz, 1H), 7.65 (t, *J* = 1.5 Hz, 1H), 2.42 (q, *J* = 7.5 Hz, 2H), 1.23 (t, *J* = 7.5 Hz, 3H); ^{13}C NMR (101 MHz, $CDCl_3$) δ 173.09, 139.83, 135.48, 132.71, 122.12, 117.03, 114.04, 93.90, 30.61, 9.49.

N-(3-Iodo-5-(1,2,4,5-tetrazin-3-yl)phenyl)propionamide

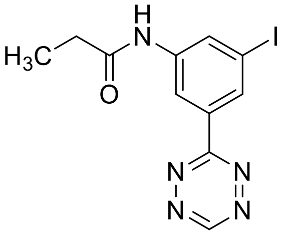

The compound was obtained from N-(5-cyano-3-iodophenyl)propionamide (0.36 g, 1.20 mmol) following general procedure A. The crude was purified using flash chromatography (70/30 n-Heptane/EtOAc) to yield 0.11 g (26%) of a red solid. *Rf* = 0.22 (n-Heptane:40%EtOAc); ^{1}H NMR (400 MHz, DMSO) δ 10.63 (s, 1H), 8.75 (d, *J* = 1.8 Hz, 1H), 8.48 – 8.35 (m, 2H), 2.38 (q, *J* = 7.5 Hz, 2H), 1.11 (t, *J* = 7.5 Hz, 3H); ^{13}C NMR (101 MHz, DMSO) δ 173.02, 164.69, 158.79, 141.85, 134.68, 131.02, 130.53, 117.58, 95.91, 30.04, 9.89.

N-(3-(1,2,4,5-tetrazin-3-yl)-5-(trimethylstannyl)phenyl)propionamide

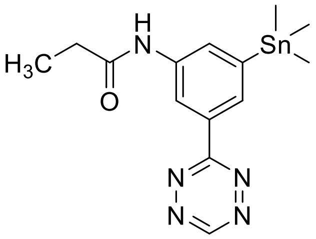

The compound was obtained from N-(3-iodo-5-(1,2,4,5-tetrazin-3-yl)phenyl)propionamide (0.056 g, 0.16 mmol) of the starting material, following the general procedure H. The crude was purified using flash chromatography (70/30 n-Heptane/EtOAc) to yield 0.036 g (57%) as a purple oil. *Rf* = 0.24 (n-Heptane:40%EtOAc); ^{1}H NMR (400 MHz, $CDCl_3$) δ 10.14 (s, 1H), 8.48 (s, 1H), 8.38 (s, 1H), 8.03 (s, 1H), 7.35 (s, 1H), 2.40 (q, *J* = 7.6 Hz, 2H), 1.22 (t, *J* = 7.6 Hz, 3H), 0.30 (s, 9H); ^{13}C NMR (101

MHz, $CDCl_3$) δ 172.23, 166.48, 157.82, 145.56, 138.48, 131.52, 131.01, 130.22, 119.21, 30.74, 9.55, -9.29; MS (ESI) m/z $[M + H]^+$: 394.0

N-(3-Cyano-5-iodophenyl)butyramide

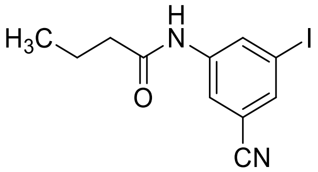

The compound was obtained from 3-amino-5-iodobenzonitrile (0.37 g, 1.5 mmol) and butyric anhydride (0.34 mL, 2.1 mmol) following the general procedure C. The crude was purified using flash chromatography (70/30 n-Heptane/EtOAc) to afford 0.37 (78%) of a white solid. *Rf* = 0.41 (n-Heptane:40%EtOAc); ^{1}H NMR (400 MHz, $CDCl_3$) δ 8.08 (t, J = 1.8 Hz, 1H), 7.96 (s, 1H), 7.84 (t, J = 1.7 Hz, 1H), 7.60 (t, J = 1.4 Hz, 1H), 2.30 (t, J = 7.4 Hz, 2H), 1.67 (h, J = 7.4 Hz, 2H), 0.92 (t, J = 7.4 Hz, 3H); ^{13}C NMR (101 MHz, $CDCl_3$) δ 172.30, 139.71, 135.56, 122.10, 116.97, 114.09, 93.89, 39.39, 18.89, 13.71.

N-(3-Iodo-5-(1,2,4,5-tetrazin-3-yl)phenyl)butyramide

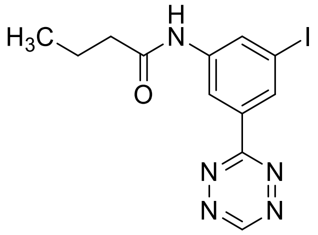

The compound was obtained from N-(5-cyano-3-iodophenyl)butyramide (0.37 g, 1.18 mmol) following general procedure A. The crude was purified using flash chromatography (70/30 n-Heptane/EtOAc) to yield 0.1 g (24%) of a red solid. *Rf* = 0.39 (n-Heptane:30%EtOAc); ^{1}H NMR (400 MHz, $CDCl_3$) δ 10.25 (s, 1H), 8.67 (t, *J* = 1.6 Hz, 1H), 8.53 (t, *J* = 1.8 Hz, 1H), 8.44 (t, *J* = 1.8 Hz, 1H), 7.56 (s, 1H), 2.42 (t, *J* = 7.4 Hz, 2H), 1.80 (h, *J* = 7.4 Hz, 2H), 1.04 (t, *J* = 7.4 Hz, 3H); ^{13}C NMR (101 MHz, $CDCl_3$) δ 171.58, 164.94, 158.06, 139.85, 133.61, 132.83, 132.50, 118.28, 95.05, 39.54, 18.89, 13.74.

N-(3-(1,2,4,5-Tetrazin-3-yl)-5-(trimethylstannyl)phenyl)butyramide

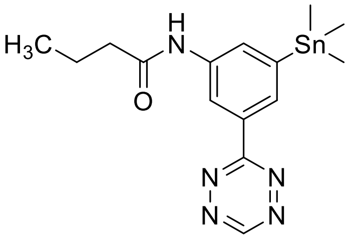

The compound was obtained from N-(3-iodo-5-(1,2,4,5-tetrazin-3-yl)phenyl)butyramide (0.059 g, 0.16 mmol) of the starting material, following the general procedure H. The crude was purified using flash chromatography (70/30 n-Heptane/EtOAc) to yield 0.035 g (54%) as a purple oil. *Rf* = 0.45 (n-Heptane:40%EtOAc); ^{1}H NMR (600 MHz, $CDCl_3$) δ 10.14 (s, 1H), 8.49 (t, *J* = 2.0 Hz, 1H), 8.44 – 8.32 (m, 1H), 8.07 – 7.93 (m, 1H), 7.36 (s, 1H), 2.33 (t, *J* = 7.4 Hz, 2H), 1.73 (h, *J* = 7.4 Hz, 2H), 0.96 (t, *J* = 7.4 Hz, 3H), 0.30 (s, 9H); ^{13}C NMR (151 MHz, $CDCl_3$) δ 171.49, 166.48, 157.82, 145.53, 138.47, 131.51, 131.02, 128.59, 119.23, 39.66, 18.96, 13.78, -9.28; MS (ESI) m/z $[M + H]^+$: 408.1

N-(3-Cyano-5-iodophenyl)pentanamide

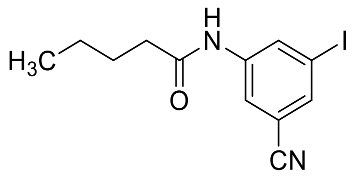

The compound was obtained from 3-amino-5-iodobenzonitrile (0.40 g, 1.64 mmol) and valeric anhydride (0.42 mL, 2.29 mmol) following the general procedure C. The crude was purified using flash chromatography (70/30 n-Heptane/EtOAc) to afford 0.41 (76%) of a white solid. *Rf* = 0.44 (n-Heptane:40%EtOAc); ^{1}H NMR (600 MHz, $CDCl_3$) δ 8.14 (s, 1H), 8.01 (s, 1H), 7.91 (s, 1H), 7.66 (s, 1H), 2.38 (t, *J* = 7.5 Hz, 2H), 1.69 (p, *J* = 7.5 Hz, 2H), 1.38 (h, *J* = 7.4 Hz, 2H), 0.92 (t, *J* = 7.3 Hz, 3H); ^{13}C NMR (151 MHz, $CDCl_3$) δ 172.37, 139.78, 135.51, 132.63, 122.07, 116.98, 114.11, 93.88, 37.28, 27.46, 22.32, 13.79.

N-(3-Iodo-5-(1,2,4,5-tetrazin-3-yl)phenyl)pentanamide

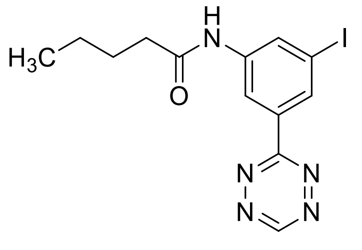

The compound was obtained from N-(5-cyano-3-iodophenyl)pentanamide (0.38 g, 1.18 mmol) following general procedure A. The crude was purified using flash chromatography (70/30 n-Heptane/EtOAc) to yield 0.12 g (26%) of a red solid. *Rf* = 0.45 (n-Heptane:30%EtOAc); ^{1}H NMR (400 MHz, CDCl3) δ 10.24 (s, 1H), 8.67 (s, 1H), 8.52 (s, 1H), 8.43 (s, 1H), 7.51 (s, 1H), 2.43 (t, J = 7.5 Hz, 2H), 1.74 (p, J = 7.6 Hz, 2H), 1.43 (h, J = 7.4 Hz, 2H), 0.96 (t, J = 7.4 Hz, 3H); ^{13}C NMR (101 MHz, $CDCl_3$) δ 171.73, 164.95, 158.06, 139.86, 133.61, 132.82, 132.49, 118.27, 95.05, 37.41, 27.48, 22.35, 13.80.

N-(3-(1,2,4,5-Tetrazin-3-yl)-5-(trimethylstannyl)phenyl)pentanamide

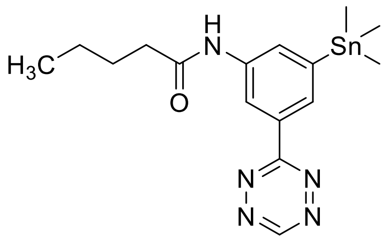

The compound was obtained from N-(3-iodo-5-(1,2,4,5-tetrazin-3-yl)phenyl)pentanamide (0.06 g, 0.16 mmol) of the starting material, following the general procedure H. The crude was purified using flash chromatography (70/30 n-Heptane/EtOAc) to yield 0.028 g (43%) as a purple oil. *Rf* = 0.47 (n-Heptane:40%EtOAc); ^{1}H NMR (400 MHz, $CDCl_3$) δ 10.20 (s, 1H), 8.55 (t, *J* = 2.0 Hz, 1H), 8.44 (d, *J* = 1.7 Hz, 1H), 8.16 – 7.98 (m, 1H), 7.47 (s, 1H), 2.42 (t, *J* = 7.5 Hz, 2H), 1.75 (p, *J* = 7.5 Hz, 2H), 1.43 (h, *J* = 7.3 Hz, 2H), 0.95 (t, *J* = 7.3 Hz, 3H), 0.36 (s, 9H); ^{13}C NMR (101 MHz, $CDCl_3$) δ 171.69, 166.47, 157.80, 145.51, 138.49, 131.49, 131.00, 119.24, 37.51, 27.58, 22.39, 13.82, -9.29; MS (ESI) m/z $[M + H]^+$: 422.1.

3-Iodo-5-(methoxycarbonyl)benzoic acid

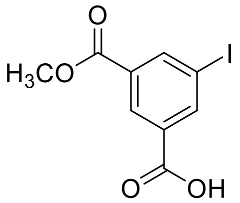

The compound was prepared as previously described by García-Vázquez et al.[7] The preparation of this intermediate, was performed using a method described previously.[7] To a solution of dimethyl 5-iodoisophthalate (12.8 g, 40 mmol), methanol (80 mL), and CH_2Cl_2 (40 mL) was added NaOH (1.68 g, 42 mmol). The mixture was allowed to stir at room temperature for 24 h. The solvents were removed under reduced pressure. Lots of white precipitate formed when water (9 mL), dichloromethane (10 mL), and ethyl acetate (10 mL) were added while stirring, which was collected by filtration, well washed with a mixture of dichloromethane (10 mL) and ethyl acetate (10 mL), and then with water (10 mL). After transferring the solid (mono sodium salt) to a separatory funnel, ethyl acetate (80 mL) and conc. HCl (3 mL) diluted with water (20 mL) were successively added. The mixture was vigorously shaken until the solid was disappeared. Then the organic layer was separated, and the aqueous layer was extracted by ethyl acetate (25 mL). The organic layers were combined and washed by brine (20 mL), dried over MgSO4, filtered, and concentrated. The solid obtained was washed recrystallized from MeOH to give 10.3 g (84%) as a white solid. *Rf* = 0.33 (CH_2Cl_2:5%MeOH:0.1%AcOH); ^{1}H NMR (400 MHz, DMSO-d_6) δ 13.57 (s, 1H), 8.49 – 8.27 (m,

3H), 3.89 (s, 3H); ^{13}C NMR (101 MHz, DMSO-d_6) δ 165.55, 164.68, 142.24, 141.62, 133.67, 132.31, 129.36, 95.42, 53.15.

Methyl 3-carbamoyl-5-iodobenzoate

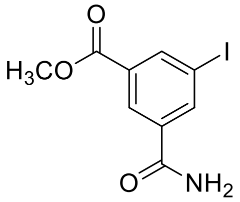

The compound was prepared as previously described by García-Vázquez et al.[7] A solution of 3-methoxycarbony5-iodobenzoic acid (10.3 g, 33.65 mmol) in thionyl chloride (30.0 mL) was heated for 2 hours at 60 °C The reaction mixture was cooled and concentrated under reduced pressure. The intermediate acid chloride was then diluted with tetrahydrofuran (40 mL) and cooled to 0 °C The mixture was then treated with a solution of 2M ammonia (60 mL, 120 mmol, methanol) and the reaction stirred for 1 hour at 0°C. The mixture was then filtered, and the solvent removed under reduced pressure. Recrystallization from methanol afforded 7.70 g (75%) as a white solid. *Rf* = 0.25 (n-Heptane:40%EtOAc); ^{1}H NMR (400 MHz, DMSO-d_6) δ 8.47 (t, J = 1.6 Hz, 1H), 8.44 (d, J = 1.6 Hz, 1H), 8.35 (t, J = 1.6 Hz, 1H), 8.24 (s, 1H), 7.61 (s, 1H), 3.89 (s, 3H); ^{13}C NMR (101 MHz, DMSO-d_6) δ 165.86, 164.96, 140.72, 140.21, 136.99, 132.10, 128.09, 95.18, 53.06.

Methyl 3-cyano-5-iodobenzoate

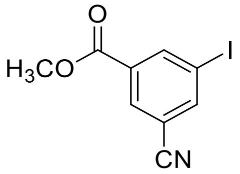

The compound was prepared as previously described by García-Vázquez et al.[7] At a temperature of about 0°C, a solution of 2.8 ml (16.5 mmol) of trifluoromethanesulphonic anhydride in 50 ml of dichloromethane was added dropwise to a solution of 2.80 g (9.18 mmol) of methyl 3-carbamoyl-5-iodobenzoate and 8 ml (45.9 mmol) of N,N-diisopropylethylamine in 150 ml of dichloromethane. After a reaction time of 30 min at 0°C, 50 ml of saturated aqueous sodium bicarbonate solution were added, and the mixture was stirred vigorously at room temperature for 10 minutes. The organic phase was separated off, dried over anhydrous $MgSO_4$, filtered and freed from the solvent on a rotary evaporator. Purification by flash chromatography (80/20 Heptane/EtOAc) afforded 2.4 g (91%) as a white solid. *Rf* = 0.5 (n-Heptane:20%EtOAc); ^{1}H NMR (400 MHz, $CDCl_3$) δ 8.58 (s, 1H), 8.27 (s, 1H), 8.14 (s, 1H), 3.96 (s, 3H); ^{13}C NMR (101 MHz, $CDCl_3$) δ 163.72, 144.07, 142.59, 132.70, 132.25, 116.25, 114.55, 93.68, 52.99.

3-Cyano-5-iodobenzoic acid

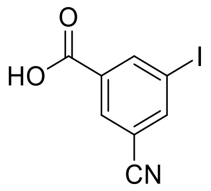

The compound was prepared as previously described by García-Vázquez et al.[7] A solution of methy3-cyano-5-iodobenzoate (2.36 g, 8.22 mmol) in THF (25 mL) was treated with 0.5M LiOH (20 mL, 9.86 mmol) and methanol. The reaction mixture was heated at reflux for 1 hour. The solvent was concentrated in vacuo and the mixture treated with 1N HCl. The resulting white precipitate was filtered, and the filtrate was extracted with dichloromethane. The residue and the extracted filtrate were combined and concentrated in vacuo to afford 2.1 g (94%) as a white solid. *Rf* = 0.33 (n-Heptane:40%EtOAc); ^{1}H NMR (400 MHz, $CDCl_3$) δ 10.73 (s, 1H), 8.65 (s, 1H), 8.35 (s, 1H), 8.22 (s, 1H); ^{13}C NMR (101 MHz, $CDCl_3$) δ 168.43, 145.01, 143.10, 132.80, 131.74, 116.04, 114.77, 93.84.

3-Cyano-5-iodobenzamide

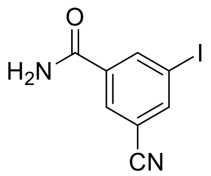

The compound was prepared as previously described by García-Vázquez et al.[7] The compound was obtained from 5-cyano-3-iodobenzoic acid (0.40 g, 1.46 mmol) and aqueous ammonium hydroxide solution (35%, 10 ml) following the general procedure D to give 0.35 g (88%) of a white solid. *Rf* = 0.44 (n-Heptane:40%EtOAc); ^{1}H NMR (400 MHz, MeOD) δ 8.41 (s, 1H), 8.18 (s, 1H), 8.11 (s, 1H); ^{13}C NMR (101 MHz, MeOD) δ 166.85, 142.88, 140.78, 136.39, 130.23, 116.17, 113.99, 93.36.

3-Iodo-5-(1,2,4,5-tetrazin-3-yl)benzamide

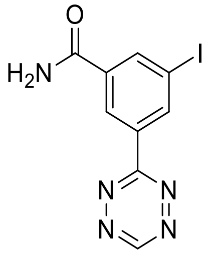

The compound was prepared as previously described by García-Vázquez et al.[7] The compound was obtained from 3-cyano-5-iodobenzamide (0.21 g, 0.77 mmol) following general procedure A. The

crude was purified using flash chromatography (98/2 CH_2Cl_2/MeOH) to yield 0.07 g (28%) as a pink solid. *Rf* = 0.41 (n-Heptane:60%EtOAC); ^{1}H NMR (400 MHz, DMSO-d_6) δ 10.68 (s, 1H), 8.99 (d, *J* = 1.7 Hz, 1H), 8.89 (d, *J* = 1.7 Hz, 1H), 8.55 (d, *J* = 1.7 Hz, 1H), 8.34 (s, 1H), 7.66 (s, 1H); ^{13}C NMR (101 MHz, DMSO-d_6) δ 165.96, 164.64, 158.84, 140.13, 138.75, 137.55, 134.60, 126.77, 95.96.

3-(1,2,4,5-Tetrazin-3-yl)-5-(trimethylstannyl)benzamide

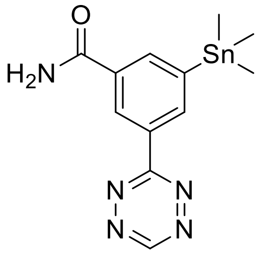

The compound was prepared as previously described by García-Vázquez et al.[7] The compound was obtained from 3-iodo-5-(1,2,4,5-tetrazin-3-yl)benzamide (0.055 g, 0.17 mmol) following the general procedure H. The crude was purified using flash chromatography (40/60 n-Heptane/EtOAc) to yield 0.025 g (41%) of a purple oil. *Rf* = 0.42 (n-Heptane:60%EtOAc); ^{1}H NMR (600 MHz, MeOD) δ 10.28 (s, 1H), 8.76 (dd, *J* = 1.8, 0.9 Hz, 1H), 8.20 (dd, *J* = 1.9, 0.8 Hz, 1H), 0.31 (s, 9H); ^{13}C NMR (151 MHz, MeOD) δ 170.47, 166.26, 158.03, 144.81, 138.52, 137.69, 134.02, 131.68, 126.83, -11.10; MS (ESI) m/z $[M + H]^+$: 366.0.

5-Cyano-3-iodo-N-methylbenzamide

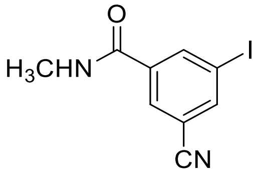

The compound was prepared as previously described by García-Vázquez et al.[7] The compound was obtained from 5-cyano-3-iodobenzoic acid (0.40 g, 1.46 mmol) and aqueous methylamine solution (80%, 5 ml) following the general procedure D to give 0.41 g (98%) of a white solid. *Rf* = 0.55 (n-Heptane:40%EtOAc); ^{1}H NMR (400 MHz, MeOD) δ 8.45 (d, *J* = 1.7 Hz, 1H), 8.27 (d, *J* = 1.9 Hz, 1H), 8.15 (s, 1H), 2.93 (s, 3H); ^{13}C NMR (101 MHz, MeOD) δ 165.38, 142.63, 140.38, 136.78, 129.83, 116.20, 113.97, 93.44, 25.67.

3-Iodo-N-methyl-5-(1,2,4,5-tetrazin-3-yl)benzamide

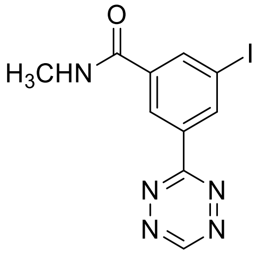

The compound was prepared as previously described by García-Vázquez et al.[7] The compound was obtained from 5-cyano-3-iodo-N-methylbenzamide (0.38 g, 1.32 mmol) following general procedure A. The crude was purified using flash chromatography (98/2 CH_2Cl_2/MeOH) to yield 0.11 g (24%) of a pink solid. *Rf* = 0.45 (n-Heptane:60%EtOAC); ^{1}H NMR (600 MHz, DMSO-d_6) δ 10.68 (s, 1H), 8.97 (s, 1H), 8.89 (s, 1H), 8.84 (q, *J* = 4.5 Hz, 1H), 8.51 (s, 1H), 2.83 (d, *J* = 4.5 Hz, 3H); ^{13}C NMR (151 MHz, DMSO-d_6) δ 164.67, 164.62 158.89, 139.78, 138.57, 137.63, 134.64, 126.31, 96.04, 26.89.

N-Methyl-3-(1,2,4,5-tetrazin-3-yl)-5-(trimethylstannyl)benzamide

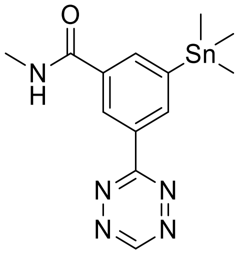

The compound was prepared as previously described by García-Vázquez et al.[7] The compound was obtained from 3-iodo-N-methyl-5-(1,2,4,5-tetrazin-3-yl)benzamide (0.05 g, 0.14 mmol) following the general procedure H. The crude was purified using flash chromatography (60/40 n-Heptane/EtOAc) to yield 0.035 g (63%) of a purple oil. *Rf* = 0.46 (n-Heptane:60%EtOAc); ^{1}H NMR (400 MHz, $CDCl_3$) δ 10.25 (s, 1H), 8.89 – 8.80 (m, 2H), 8.26 (dd, *J* = 1.9, 0.9 Hz, 1H), 6.37 (s, 1H), 3.08 (d, *J* = 4.8 Hz, 3H), 0.39 (s, 9H); ^{13}C NMR (101 MHz $CDCl_3$) δ 167.68, 166.22, 157.96, 145.55, 139.44, 138.02, 134.89, 130.88, 125.60, 26.97, -9.23; MS (ESI) m/z $[M + H]^+$: 380.0.

3-Cyano-N-ethyl-5-iodobenzamide

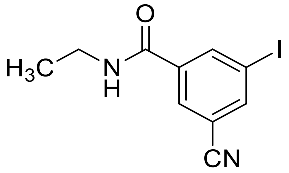

The compound was obtained from 5-cyano-3-iodobenzoic acid (0.40 g, 1.46 mmol) and aqueous ethylamine solution (70%, 5 ml) following the general procedure D to give 0.62 g (81%) of a white solid. *Rf* = 0.59 (n-Heptane:40%EtOAc); ^{1}H NMR (400 MHz, MeOD) δ 8.47 (t, *J* = 1.6 Hz, 1H), 8.28

(t, J = 1.6 Hz, 1H), 8.17 (t, J = 1.5 Hz, 1H), 3.42 (q, J = 7.3 Hz, 2H), 1.24 (t, J = 7.3 Hz, 3H); ^{13}C NMR (101 MHz, MeOD) δ 164.68, 142.57, 140.39, 137.04, 129.87, 116.19, 113.98, 93.38, 34.69, 13.22.

N-Ethyl-3-iodo-5-(1,2,4,5-tetrazin-3-yl)benzamide

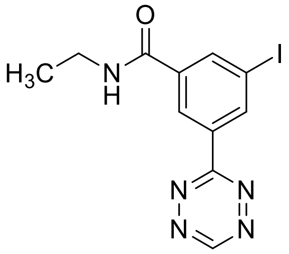

The compound was obtained from 3-cyano-N-ethyl-5-iodobenzamide (0.6 g, 2.00 mmol) following general procedure A. The crude was purified using flash chromatography (98/2 CH_2Cl_2/MeOH) to yield 0.23 g (32%) of a pink solid. *Rf* = 0.29 (n-Heptane:30%EtOAC); ^{1}H NMR (400 MHz, $CDCl_3$) δ 10.23 (s, 1H), 9.03 (t, J = 1.6 Hz, 1H), 8.83 (t, J = 1.6 Hz, 1H), 8.39 (t, J = 1.7 Hz, 1H), 6.22 (s, 1H), 3.47 (td, J = 7.2, 5.5 Hz, 2H), 1.24 (t, J = 7.3 Hz, 3H); ^{13}C NMR (101 MHz, $CDCl_3$) δ 164.75, 158.21, 140.95, 139.42, 137.57, 133.43, 124.98, 95.19, 35.31, 14.83.

N-Ethyl-3-(1,2,4,5-tetrazin-3-yl)-5-(trimethylstannyl)benzamide

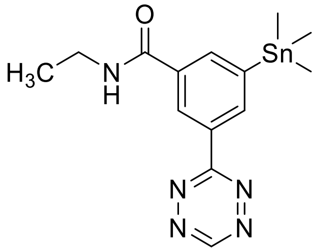

The compound was obtained from N-ethyl-3-iodo-5-(1,2,4,5-tetrazin-3-yl)benzamide (0.06 g, 0.17 mmol) following the general procedure H. The crude was purified using flash chromatography (60/40 n-Heptane/EtOAc) to yield 0.035 g (53%) of a purple oil. *Rf* = 0.23 (n-Heptane:30%EtOAc); ^{1}H NMR (400 MHz, $CDCl_3$) δ 10.26 (s, 1H), 8.85 (s, 1H), 8.81 (s, 1H), 8.27 (d, J = 1.0 Hz, 1H), 6.33 (s, 1H), 3.61 – 3.50 (m, 2H), 1.30 (t, J = 7.2 Hz, 3H), 0.39 (s, 9H); ^{13}C NMR (101 MHz, $CDCl_3$) δ 166.87, 166.24, 157.95, 145.54, 139.60, 138.00, 135.03, 130.81, 125.46, 35.16, 14.92, -9.23; MS (ESI) m/z $[M + H]^+$: 394.1.

3-Cyano-5-iodo-N-propylbenzamide

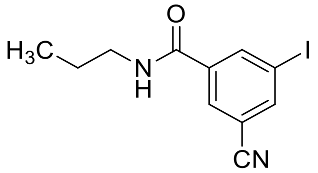

The compound was obtained from 5-cyano-3-iodobenzoic acid (0.20 g, 2.56 mmol) and propylamine (0.22 g, 3.75 mmol) following the general procedure D to give 0.79 g (98%) of a white solid. *Rf* = 0.61 (n-Heptane:40%EtOAc); ^{1}H NMR (400 MHz, MeOD) δ 8.46 (s, 1H), 8.28 (s, 1H), 8.16 (s, 1H), 3.41 – 3.27 (m, 2H), 1.65 (q, *J* = 7.3 Hz, 2H), 0.99 (t, *J* = 7.4 Hz, 3H); ^{13}C NMR (101 MHz, MeOD) δ 164.86, 142.58, 140.40, 137.05, 129.88, 116.20, 113.97, 93.41, 41.62, 22.12, 10.34.

3-Iodo-N-propyl-5-(1,2,4,5-tetrazin-3-yl)benzamide

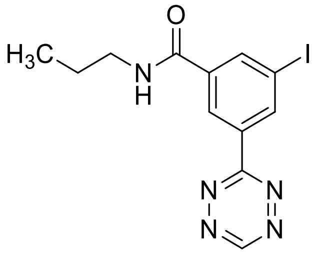

The compound was obtained from 3-cyano-5-iodo-N-propylbenzamide (0.77 g, 2.45 mmol) following general procedure A. The crude was purified using flash chromatography (98/2 CH_2Cl_2/MeOH) to yield 0.22 g (24%) of a pink solid. *Rf* = 0.31 (n-Heptane:30%EtOAC); ^{1}H NMR (400 MHz, $CDCl_3$) δ 10.22 (s, 1H), 8.98 (s, 1H), 8.80 (s, 1H), 8.36 (s, 1H), 6.43 (d, *J* = 5.9 Hz, 1H), 3.45 – 3.35 (m, 2H), 1.62 (h, *J* = 7.4 Hz, 2H), 0.94 (t, *J* = 7.4 Hz, 3H); ^{13}C NMR (101 MHz, $CDCl_3$) δ 164.95, 164.72, 158.18, 140.90, 139.34, 137.63, 133.39, 125.08, 95.14, 42.13, 22.86, 11.48.

N-propyl-3-(1,2,4,5-tetrazin-3-yl)-5-(trimethylstannyl)benzamide

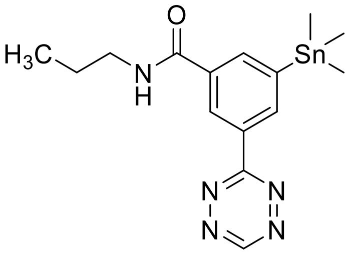

The compound was obtained from 3-iodo-N-propyl-5-(1,2,4,5-tetrazin-3-yl)benzamide (0.062 g, 0.17 mmol) following the general procedure H. The crude was purified using flash chromatography (60/40 n-Heptane/EtOAc) to yield 0.035 g (51%) of a purple oil. *Rf* = 0.27 (n-Heptane:30%EtOAc); ^{1}H NMR (400 MHz, $CDCl_3$) δ 10.25 (s, 1H), 8.85 (s, 1H), 8.81 (s, 1H), 8.28 (dd, *J* = 1.8, 0.9 Hz, 1H), 6.37 (s, 1H), 4.16 – 3.00 (m, 2H), 2.70 – 1.39 (m, 2H), 1.02 (t, *J* = 7.4 Hz, 3H), 0.39 (s, 9H); ^{13}C NMR (101

MHz, $CDCl_3$) δ 166.99, 166.24, 157.95, 145.54, 139.62, 138.00, 135.08, 130.82, 125.43, 41.98, 22.96, 11.49, -9.24; MS (ESI) m/z $[M + H]^+$: 408.0.

3-(Bromomethyl)-5-iodobenzonitrile

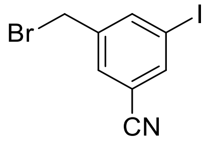

To a solution of 3-iodo-5-methylbenzonitrile (2.50 g, 10.28 mmol) and N-bromosuccinimide (2.28 g, 12.86 mmol) in $CHCl_3$ (40 mL) was added AIBN (0.67 g, 4.11 mmol). The reaction was refluxed for 24 h. The solvent was removed under vacuum and the crude purified by flash chromatography (heptane/EtOAc 95/5) to give 1.61 g (49%) of a white solid. *Rf* = 0.28 (n-Heptane:5%EtOAc); ^{1}H NMR (400 MHz, $CDCl_3$) δ 7.96 (d, *J* = 1.6 Hz, 1H), 7.89 (d, *J* = 1.6 Hz, 1H), 7.64 (t, *J* = 1.6 Hz, 1H), 4.38 (s, 2H) ^{13}C NMR (101 MHz, $CDCl_3$) δ 142.20, 140.90, 140.12, 131.67, 116.55, 114.56, 94.05, 29.90.

3-(Aminomethyl)-5-iodobenzonitrile hydrochloride

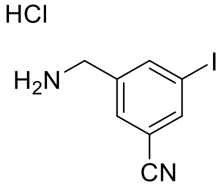

The compound was obtained from 3-(bromomethyl)-5-iodobenzonitrile (0.6 g, 1.86 mmol) and 7N solution of ammonia in MeOH (5 mL) following the general procedure E to give 0.43 g (78%) of 3-(aminomethyl)-5-iodobenzonitrile hydrochloride as a white solid. ^{1}H NMR (400 MHz, DMSO-d_6) δ 8.58 (br s, 3H), 8.29 – 8.26 (m, 2H), 8.03 (d, *J* = 1.6 Hz, 1H), 4.15 – 3.98 (m, 2H); ^{13}C NMR (101 MHz, DMSO-d_6) δ 143.04, 140.21, 137.94, 132.70, 117.50, 113.43, 95.78, 41.15.

***Tert*-butyl (3-cyano-5-iodobenzyl)carbamate**

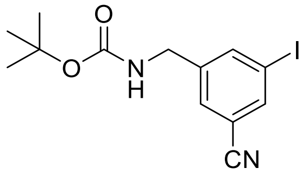

The compound was obtained from 3-(aminomethyl)-5-iodobenzonitrile hydrochloride (0.43 g, 1.46 mmol) following the general procedure F. Purification by flash chromatography (Heptane/EtOAc = 85/15) afforded 0.51 g (97%) of the desired compound as an orange solid (mixture of rotamers). *Rf* = 0.28 (Heptane:20%EtOAc); ^{1}H NMR (400 MHz, $CDCl_3$) δ 7.78 (br s, 2H), 7.47 (s, 1H), 5.05 (t, *J* =

6.5 Hz, 1H), 4.22 (d, *J* = 6.3 Hz, 2H), 1.39 (s, 9H); ^{13}C NMR (101 MHz, $CDCl_3$) δ 155.79, 142.77, 140.64, 139.10, 129.88, 117.02, 114.22, 94.04, 80.29, 43.25, 27.41.

***Tert*-butyl (3-iodo-5-(1,2,4,5-tetrazin-3-yl)benzyl)carbamate**

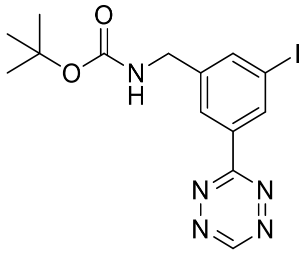

The compound was obtained from *tert*-butyl (3-cyano-5-iodobenzyl)carbamate (0.43 g, 1.20 mmol) following the general procedure A. Purification by flash chromatography (95/5 Heptane/EtOAc) afforded 0.11 g (22%) of the desired compound as a red solid (mixture of rotamers). *Rf* = 0.35 (Heptane:20%EtOAc); ^{1}H NMR (400 MHz, $CDCl_3$) δ 10.24 (s, 1H), 8.85 (d, *J* = 1.7 Hz, 1H), 8.49 (s, 1H), 7.90 (d, *J* = 1.8 Hz, 1H), 5.07 (s, 1H), 4.40 (d, *J* = 4.9 Hz, 2H), 1.47 (s, 9H); ^{13}C NMR (101 MHz, $CDCl_3$) δ 165.16, 158.03, 155.83, 142.66, 140.69, 135.88, 133.54, 126.13, 95.10, 80.12, 43.76, 28.37.

***Tert*-butyl (3-(1,2,4,5-tetrazin-3-yl)-5-(trimethylstannyl)benzyl)carbamate**

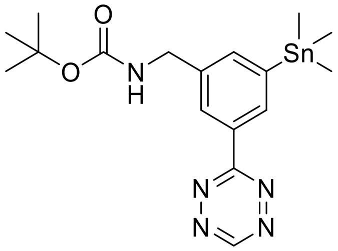

The compound was obtained from *tert*-butyl (3-iodo-5-(1,2,4,5-tetrazin-3-yl)benzyl)carbamate (0.072 g, 0.17 mmol) following the general procedure H. The crude was purified using flash chromatography (90/10 n-Heptane/EtOAc) to yield 0.040 g (51%) of a purple oil (mixture of rotamers). *Rf* = 0.43 (n-Heptane:30%EtOAc); ^{1}H NMR (600 MHz, $CDCl_3$) δ 10.21 (s, 1H), 8.69 – 8.56 (m, 1H), 8.45 (t, *J* = 1.9 Hz, 1H), 7.68 (d, *J* = 1.8 Hz, 1H), 5.00 (s, 1H), 4.44 (d, *J* = 6.1 Hz, 2H), 1.48 (s, 9H), 0.36 (s, 9H); ^{13}C NMR (151 MHz, $CDCl_3$) δ 166.67, 157.79, 157.77, 155.93, 144.72, 139.56, 134.42, 131.16, 126.93, 79.76, 44.51, 28.41, -9.33; MS (ESI) m/z $[M + H]^+$: 452.1.

3-Iodo-5-((methylamino)methyl)benzonitrile hydrochloride

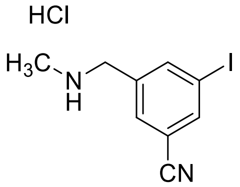

The compound was obtained from 3-(bromomethyl)-5-iodobenzonitrile (0.7 g, 2.17 mmol) and aqueous methylamine solution (40%, 5 ml) following the general procedure E to give 0.46 g (69%) of 3-iodo-5-((methylamino)methyl)benzonitrile hydrochloride as a white solid. ^{1}H NMR (400 MHz, DMSO-d_6) δ 9.28 (s, 2H), 8.32 (d, *J* = 1.6 Hz, 1H), 8.29 (d, *J* = 1.7 Hz, 1H), 8.04 (s, 1H), 4.13 (d, *J* = 5.0 Hz, 2H), 2.54 (s, 3H); ^{13}C NMR (101 MHz, DMSO-d_6) δ 143.91, 140.77, 135.86, 135.81, 133.59, 117.44, 113.59, 113.56, 95.88, 49.94, 32.54.

Tert-butyl (3-cyano-5-iodobenzyl)(methyl)carbamate

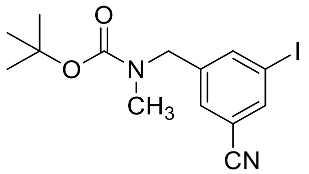

The compound was obtained from 3-iodo-5-((methylamino)methyl)benzonitrile hydrochloride (0.45 g, 1.45 mmol) following the general procedure F. Purification by flash chromatography (Heptane/EtOAc = 85/15) afforded 0.54 g (99%) of the desired compound as a colorless oil (mixture of rotamers). *Rf* = 0.37 (Heptane:20%EtOAc); ^{1}H NMR (600 MHz, $CDCl_3$) δ 7.80 (s, 1H), 7.74 (s, 1H), 7.41 (s, 1H), 4.32 (s, 2H), 2.87 – 2.66 (m, 3H), 1.48 – 1.36 (m, 9H); ^{13}C NMR (151 MHz, $CDCl_3$) δ 141.80, 140.84, 140.70, 140.62, 139.10, 130.07, 129.84, 116.96, 114.25, 94.12, 80.43, 80.40, 51.59, 50.97, 34.50, 28.35.

Tert-butyl (3-iodo-5-(1,2,4,5-tetrazin-3-yl)benzyl)(methyl)carbamate

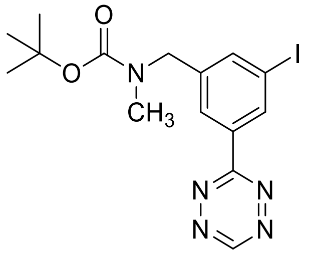

The compound was obtained from *tert*-butyl 3-cyano-5-iodobenzyl(methyl)carbamate (0.54 g, 1.45 mmol) following the general procedure A. Purification by flash chromatography (95/5 Heptane/EtOAc) afforded 0.18 g (29%) of the desired compound as a red oil (mixture of rotamers). *Rf* = 0.36 (Heptane:20%EtOAc); ^{1}H NMR (600 MHz, $CDCl_3$) δ 10.25 (s, 1H), 8.88 (s, 1H), 8.46 (s, 1H), 7.86 (s, 1H), 4.60 – 4.43 (m, 2H), 2.99 – 2.81 (m, 3H), 1.49 (s, 9H); ^{13}C NMR (151 MHz, $CDCl_3$)

δ 164.17, 157.02, 155.14, 154.45, 140.78, 139.81, 134.90, 132.57, 125.35, 94.11, 51.06, 50.37, 33.42, 27.41.

***Tert*-butyl (3-(1,2,4,5-tetrazin-3-yl)-5-(trimethylstannyl)benzyl)(methyl)carbamate**

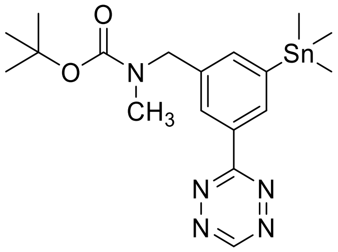

The compound was obtained from *tert*-butyl 3-iodo-5-(1,2,4,5-tetrazin-3-yl)benzyl(methyl)carbamate (0.074 g, 0.17 mmol) following the general procedure H. The crude was purified using flash chromatography (90/10 n-Heptane/EtOAc) to yield 0.042 g (52%) of a purple oil (mixture of rotamers). *Rf* = 0.37 (n-Heptane:20%EtOAc); ^{1}H NMR (400 MHz, $CDCl_3$) δ 10.21 (s, 1H), 8.63 (d, *J* = 1.7 Hz, 1H), 8.41 (s, 1H), 7.64 (s, 1H), 4.53 (s, 2H), 2.90 (s, 3H), 1.50 (s, 9H), 0.36 (s, 9H); ^{13}C NMR (101 MHz, $CDCl_3$) δ 166.70, 157.78, 144.62, 139.57, 138.75, 134.39, 131.12, 128.43, 127.21, 52.66, 34.20, 28.47, -9.34; MS (ESI) m/z $[M + H]^+$: 466.1.

3-((Ethylamino)methyl)-5-iodobenzonitrile hydrochloride

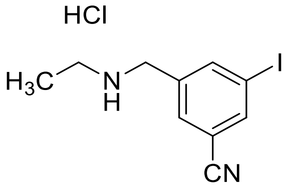

The compound was obtained from 3-(bromomethyl)-5-iodobenzonitrile (0.8 g, 2.48 mmol) and aqueous ethylamine solution (70%, 5 ml) following the general procedure E to give 0.46 g (57%) of 3-((ethylamino)methyl)-5-iodobenzonitrile hydrochloride as a white solid. ^{1}H NMR (400 MHz, DMSO-d_6) δ 9.20 (s, 2H), 8.37 – 8.27 (m, 2H), 8.06 (d, J = 1.7 Hz, 1H), 4.15 (t, J = 5.6 Hz, 2H), 2.95 (q, J = 6.5 Hz, 2H), 1.22 (t, J = 7.2 Hz, 3H); ^{13}C NMR (101 MHz, DMSO-d_6) δ 143.93, 143.90, 140.67, 136.02, 133.63, 117.44, 113.54, 95.83, 48.28, 42.38, 11.39, 11.37.

***Tert*-butyl (3-cyano-5-iodobenzyl)(ethyl)carbamate**

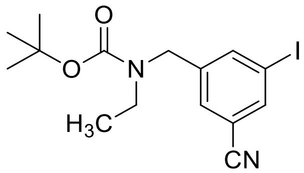

The compound was obtained from 3-((ethylamino)methyl)-5-iodobenzonitrile hydrochloride (0.45 g, 1.49 mmol) following the general procedure F. Purification by flash chromatography (Heptane/EtOAc = 85/15) afforded 0.53 g (98%) of the desired compound as a colorless oil (mixture of rotamers). *Rf* = 0.45 (Heptane:20%EtOAc); ^{1}H NMR (600 MHz, $CDCl_3$) δ 7.84 – 7.60 (m, 2H), 7.40 (s, 1H), 4.29 (s, 2H), 3.27 – 3.02 (m, 2H), 1.41 – 1.24 (m, 9H), 1.07 – 0.90 (m, 3H); ^{13}C NMR (151 MHz, $CDCl_3$) δ 155.68, 154.75, 142.56, 140.67, 138.86, 129.96, 116.95, 114.10, 94.01, 80.13, 48.77, 42.11, 28.33, 27.35.

***Tert*-butyl (3-iodo-5-(1,2,4,5-tetrazin-3-yl)benzyl)(ethyl)carbamate**

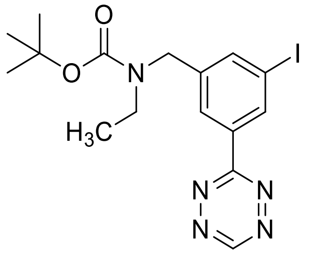

The compound was obtained from *tert*-butyl 3-cyano-5-iodobenzyl(ethyl)carbamate (0.53 g, 1.37 mmol) following the general procedure A. Purification by flash chromatography (95/5 Heptane/EtOAc) afforded 0.13 g (21%) of the desired compound as a red oil (mixture of rotamers). *Rf* = 0.39 (Heptane:20%EtOAc); ^{1}H NMR (600 MHz, $CDCl_3$) δ 10.23 (s, 1H), 8.85 (s, 1H), 8.45 (s, 1H), 7.86 (s, 1H), 4.60 – 4.32 (m, 2H), 3.40 – 3.12 (m, 2H), 1.48 (s, 9H), 1.11 (s, 3H); ^{13}C NMR (151 MHz, $CDCl_3$) δ 165.19, 158.02, 155.95, 155.09, 142.51, 140.79, 135.75, 133.48, 126.26, 95.05, 80.18, 49.42, 49.07, 41.96, 28.43, 13.34.

***Ter*t-butyl (3-(1,2,4,5-tetrazin-3-yl)-5-(trimethylstannyl)benzyl)(ethyl)carbamate**

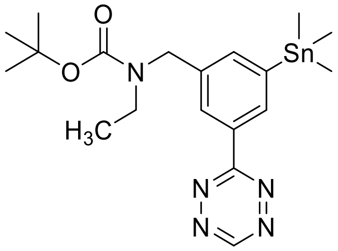

The compound was obtained from *tert*-butyl 3-iodo-5-(1,2,4,5-tetrazin-3-yl)benzyl(ethyl)carbamate (0.04 g, 0.09 mmol) following the general procedure H. The crude was purified using flash chromatography (90/10 n-Heptane/EtOAc) to yield 0.030 g (69%) of a purple oil (mixture of rotamers). *Rf* = 0.47 (n-Heptane:20%EtOAc); ^{1}H NMR (600 MHz, $CDCl_3$) δ 10.14 (s, 1H), 8.55 (s, 1H), 8.34 (s, 1H), 7.59 (s, 1H), 4.61 – 4.34 (m, 2H), 3.42 – 3.11 (m, 2H), 1.55 – 1.28 (m, 9H), 1.05 (s, 3H), 0.29 (s, 9H); ^{13}C NMR (151 MHz, $CDCl_3$) δ 165.72, 156.74, 155.02, 154.26, 143.44, 138.40,

133.22, 130.00, 126.07, 49.04, 48.62, 40.71, 27.45, 12.45, 12.22, -10.38; MS (ESI) m/z $[M + H]^+$: 480.1..

3-((Propylamino)methyl)-5-iodobenzonitrile hydrochloride

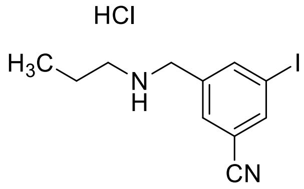

The compound was obtained from 3-(bromomethyl)-5-iodobenzonitrile (0.7 g, 2.17 mmol) and propylamine (2.1 mL, 24.84 mmol) following the general procedure E to give 0.48 g (66%) 3-((propylamino)methyl)-5-iodobenzonitrile hydrochloride as a white solid. ^{1}H NMR (600 MHz, DMSO) δ 9.39 (s, 2H), 8.33 (t, *J* = 1.6 Hz, 1H), 8.31 (t, *J* = 1.6 Hz, 1H), 8.10 (t, *J* = 1.5 Hz, 1H), 4.14 (t, *J* = 5.7 Hz, 2H), 2.89 – 2.76 (m, 2H), 1.73 – 1.59 (m, 2H), 0.91 (t, *J* = 7.4 Hz, 3H); ^{13}C NMR (151 MHz, DMSO) δ 144.01, 140.66, 135.94, 133.73, 117.47, 113.49, 95.80, 48.77, 40.55, 19.40, 11.45.

***Tert*-butyl (3-cyano-5-iodobenzyl)(propyl)carbamate**

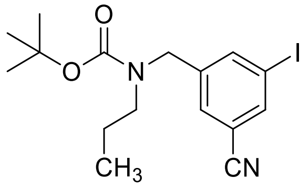

The compound was obtained from 3-iodo-5-((propylamino)methyl)benzonitrile hydrochloride (0.47 g, 1.40 mmol) following the general procedure F. Purification by flash chromatography (Heptane/EtOAc = 85/15) afforded 0.55 g (98%) of the desired compound as a colorless oil (mixture of rotamers). *Rf* = 0.55 (Heptane:20%EtOAc); ^{1}H NMR (400 MHz, $CDCl_3$) δ 7.80 (s, 1H), 7.76 (s, 1H), 7.43 (s, 1H), 4.34 (s, 2H), 3.09 (s, 2H), 1.53 – 1.27 (m, 11H), 0.82 (t, *J* = 7.4 Hz, 3H); ^{13}C NMR (101 MHz, $CDCl_3$) δ 155.91, 142.49, 140.63, 138.93, 129.83, 117.01, 114.15, 93.99, 80.25, 49.05, 28.33, 21.45, 11.19.

***Tert*-butyl (3-iodo-5-(1,2,4,5-tetrazin-3-yl)benzyl)(propyl)carbamate**

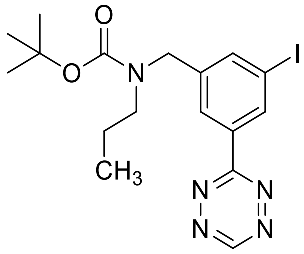

The compound was obtained from *tert*-butyl 3-cyano-5-iodobenzyl(propyl)carbamate (0.55 g, 1.37 mmol) following the general procedure A. Purification by flash chromatography (95/5 Heptane/EtOAc) afforded 0.15 g (24%) of the desired compound as a red oil (mixture of rotamers). *Rf* = 0.43 (Heptane:20%EtOAc); ^{1}H NMR (600 MHz, $CDCl_3$) δ 10.24 (s, 1H), 8.86 (s, 1H), 8.45 (s, 1H), 7.86 (s, 1H), 4.59 – 4.37 (m, 2H), 3.34 – 3.06 (m, 2H), 1.68 – 1.35 (m, 11H), 0.87 (t, *J* = 7.3 Hz, 3H); ^{13}C NMR (151 MHz, $CDCl_3$) δ 165.21, 158.02, 156.14, 155.32, 142.62, 142.37, 140.81, 140.59, 135.74, 133.47, 126.20, 95.11, 80.15, 49.97, 49.39, 48.94, 28.41, 21.53, 21.34, 11.26.

***Ter*t-butyl (3-(1,2,4,5-tetrazin-3-yl)-5-(trimethylstannyl)benzyl)(ethyl)carbamate**

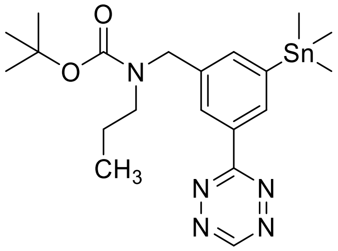

The compound was obtained from *tert*-butyl 3-iodo-5-(1,2,4,5-tetrazin-3-yl)benzyl(propyl)carbamate (0.05 g, 0.11 mmol) following the general procedure H. The crude was purified using flash chromatography (90/10 n-Heptane/EtOAc) to yield 0.025 g (46%) of a purple oil (mixture of rotamers). *Rf* = 0.48 (n-Heptane:20%EtOAc); ^{1}H NMR (600 MHz, $CDCl_3$) δ 10.21 (s, 1H), 8.64 (d, *J* = 22.2 Hz, 1H), 8.48 – 8.27 (m, 1H), 7.72 – 7.58 (m, 1H), 4.64 – 4.45 (m, 2H), 3.35 – 3.05 (m, 2H), 1.63 – 1.39 (m, 11H), 0.88 (t, *J* = 6.8 Hz, 3H, partially covered by n-Heptane signal), 0.36 (s, 9H); ^{13}C NMR (151 MHz, $CDCl_3$) δ 166.76, 157.78, 156.27, 155.56, 144.46, 139.63, 139.34, 135.23, 134.24, 131.02, 127.04, 50.60, 49.97, 48.74, 28.45, 21.60, 21.26, 11.30, -9.37; MS (ESI) m/z [M + H]$^+$: 494.1.

Section S5: Estimation of click rate constant for ^{19}F-Tzs

Stopped-flow measurements were performed as reported by Battisti et al.[8] (E)-Cyclooct-4-en-1-yl (2-(2-(2-(2-hydroxyethoxy)ethoxy)ethoxy)ethyl)carbamate (axTCO-PEG$_4$) the pegylated carbamate ester of axial TCO-alcohol [9] was used as standard TCO.

Kinetic rate constant for compound **1** was estimated by linear extrapolation from Hammett parameters as described previously [8].

Kinetic rate constants for compounds **6**, **16-18** were assumed to be equal to the lightest homologous Tzs from the same Tz groups [8]. As follows from Figure 2 in the main manuscript, the rate constants for different homologs in the same group do not differ much, therefore we believe that for the current purposes this is a valid assumption.

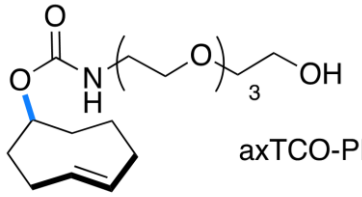

axTCO-PEG$_4$

Section S6: Radiochemistry

General information. Radiochemistry was performed at Department of Clinical Physiology, Nuclear Medicine & PET, Rigshospitalet, Denmark. [^{18}F]Fluoride was produced via the (p,n)-reaction in a cyclotron (60 mikroA CTI Siemens or 40 mikroA Scanditronix) by irradiating [^{18}O]H_2O with a 11 MeV (CTI siemens) or 16 MeV (Scanditronix) proton beam. Automated syntheses were performed on a Scansys Laboratorieteknik synthesis module housed in a hot cell. Analytical HPLC was performed on a Dionex system connected to a P680A pump, a UVD 170U detector and a Scansys radiodetector. Semi-preparative HPLC was performed on the built-in HPLC system in the synthesis module and the flow rate was set to 4 mL/min at all times.

Radiochemical conversion (RCC) of all radiolabeled compounds was determined by analyzing an aliquot labeling of the reaction mixture by radio-HPLC. The products were characterized by comparing the radio-HPLC trace of the reaction mixtures with the HPLC UV traces of the authentic ^{19}F-reference samples, respectively. The radiochemical yield (RCY) was determined using the activity of [^{18}F]fluoride received from the cyclotron at the beginning of the synthesis and that of the formulated product at the end of the synthesis, the decomposition was corrected and have been decay corrected (d.c.). The molar activity (A_m) was determined by integrating the area of the UV absorbance peak corresponding to the radiolabeled product on the HPLC chromatogram. This area was converted into a molar mass by comparison with an average of integrated areas (triplet) of a known concentration for the corresponding reference compounds.[10] The values for radiochemical yield (RCY), radiochemical purity (RCP) and molar activity (A_m) are given as mean values. This applies for all radiolabeled compounds described below.

Tetrazine labelling with ^{18}F

General procedure for the preparation of anhydrous [^{18}F]fluoride for radiolabeling. All QMA anion exchange cartridges (Sep-Pak Accell Plus QMA Plus Light, chloride form, Waters) were washed with EtOH (10 mL), 90 mg/mL KOTf (aq) (10 mL), water (10 mL) and dried with air before use. All C18 cartridges (Sep-Pak C18 Plus Short types) were washed with EtOH (10 mL), water (10 mL) and dried with air before use. Irradiated [^{18}O]water containing [^{18}F]F^- was passed through an anion exchange resin cartridge (Sep-Pak Accell Plus QMA Plus Light, chloride form). [^{18}F]Fluoride trapped on the QMA was then eluted with a mixture of KOTf (10 mg) and K_2CO_3 (50 ug) in 550 µL water. The resulting mixture was then gently concentrated to dryness at 100 °C by azeotropic drying with 2xMeCN (0.6 mL), under a nitrogen stream for 20 min, to give no-carrier-added K[^{18}F]F complex as a white semi-solid residue.

Manual labeling of [^{18}F]Tz 1-18. The preparation of the final compounds, was performed using a method described previously.[7] Samples were analysed via radio-HPLC to determine the radiochemical conversion (RCC) of [^{18}F]Tz.

Automated synthesis of [^{18}F]Tz 1-14 starting from stannane precursors.

One step synthesis procedure. Automated synthesis was performed on a Scansys Laboratorieteknik synthesis module. The same procedure was used as described previously.[7] The organotin precursor (0.01 mmol), $Cu(OTf)_2$ (7.2 mg, 0.02mmol), and pyridine (12 µL, 0.15 mmol) was dissolved in 1 mL DMA, and added to a reaction vial containing the dried [^{18}F]FK. The reaction proceed at 100 °C for 5 minutes. The solution was then cooled to 40 °C before quenched with 2 mL of H_2O/0.1%TFA. The crude reaction was purified via semi-preparative HPLC (Thermo Fisher UltiMate 3000) with a C-18 column (Luna 5 µm C18(2) 100 Å, 250 mm × 10 mm) using a flowrate of 4 mL/min. Different H_2O/MeCN (v/v) and H_2O/MeOH (v/v) solvent mixtures were used for the semi-preparative HPLC purification of each radiolabeled ^{18}F-Tz (Table S1). The fraction containing the [^{18}F]Tz was collected in H_2O (60 mL) and applied to a SPE and subsequently formulated by eluting with 1 mL EtOH into 0.1 M phosphate buffer (PBS, pH 7.4) to a final concentration of 15%EtOH/PBS. The automated synthesis from [^{18}F]fluoride from the cyclotron to formulation of the radiotracer was carried out within 60 minutes.

Automated synthesis of [^{18}F]Tz 15-18 starting from stannane precursors.

Two steps synthesis procedure. The same procedure was used as described above with minor differences.[7] After the reaction was completed, the reaction mixture was cooled before quenched with 2 mL of H_2O/0.1%TFA. The crude solution was put through a SPE cartridge. The SPE was washed with 10 mL water and dried with air before eluted with 3 mL of MeCN into a vial containing 1 mL TFA. The mixture containing the protected ^{18}F-Tz was heated during 10 minutes at 80 °C for fully deprotection. The resulting mixture was then gently concentrated and cooled before the addition of 3 mL of water. The crude was then purified via semi-preparative HPLC (Thermo Fisher UltiMate 3000) with a C-18 column (Luna 5 µm C18(2) 100 Å, 250 mm x 10 mm) using an isocratic method consisting on 15% EtOH in water 0.1% TFA, flowrate 4 mL/min. The automated synthesis from [^{18}F]fluoride from the cyclotron to formulation of the radiotracer was carried out within 90 minutes.

Radiochemical yield (RCY), radiochemical purity (RCP) and molar activity (A_m) of [^{18}F]Tz were determined according to the *General information* described above and can be found in Table S1. Radio-HPLC and UV HPLC analytical traces of the formulated product can be found in *Section S12: Analytical Radio-HPLC and semi-preparative HPLC chromatograms of formulated [^{18}F]Tzs.*

Table S1. Radiolabeling of tetrazines via Cu-mediated ^{18}F-fluorodestannylation.

Compound	*Structure*	*RCC (%)* [a]	*RCY (%)* [b]	*RCP (%)* [a]	*Am Gbq/µmol (d.c.)*	*Solvent Semi-prep HPLC*	*R.t. Semi-prep HPLC (s)*
*[^{18}F]**1***	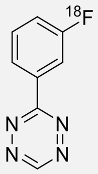	11 ± 4	11 ± 1	≥ 99	134 ± 13	50/50 [c]	900
*[^{18}F]**2***	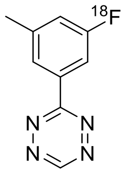	14 ± 3	11 ± 6	≥ 99	108 ± 13	50/50 [c]	720
*[^{18}F]**3***	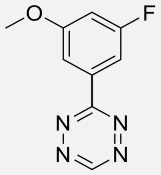	17 ± 3	15 ± 4	≥ 99	145 ±10	50/50 [c]	1500
*[^{18}F]**4***	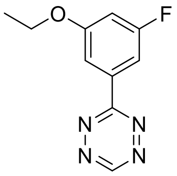	18 ± 1	16 ± 1	≥ 98	156 ± 8	60/40 [c]	850
*[^{18}F]**5***	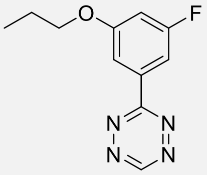	—[f]	—[f]	—[f]	—[f]	—[f]	—[f]
*[^{18}F]**6***	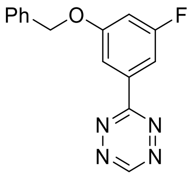	—[f]	—[f]	—[f]	—[f]	—[f]	—[f]
*[^{18}F]**7***	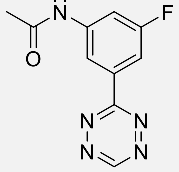	31 ± 4	16 ± 2	≥ 98	73 ± 21	35/65 [c]	1150
*[^{18}F]**8***	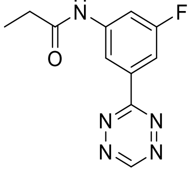	15 ± 3	14 ± 2	≥ 98	142 ± 6	50/50 [c]	1600

*[^{18}F]**9***	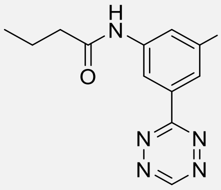	20 ± 3	16 ± 2	≥ 99	153 ± 5	50/40 [d]	2300
*[^{18}F]**10***	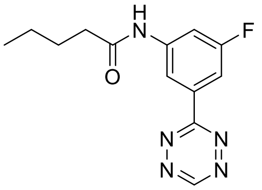	18 ± 3	15 ± 2	≥ 99	140 ± 5	50/40 [c]	1150
*[^{18}F]**11***	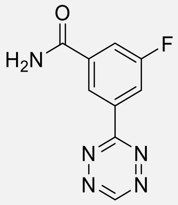	25 ± 3	15 ± 2	≥ 98	102 ± 18	23/77 [c]	850
*[^{18}F]**12***	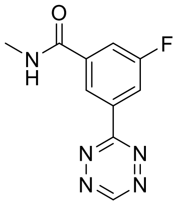	10 ± 6	9 ± 3	≥ 98	90 ± 10	25/75 [c]	1000
*[^{18}F]**13***	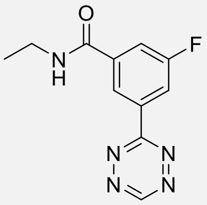	23 ± 6	14 ± 3	≥ 98	114 ± 22	30/70 [c]	1100
*[^{18}F]**14***	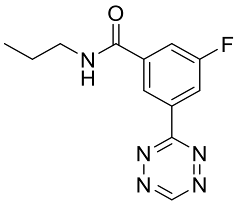	26 ± 1	11 ± 1	≥ 98	196 ± 14	35/65 [c]	1600
*[^{18}F]**15***	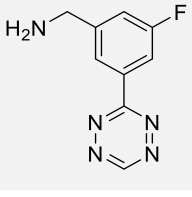	29 ± 2	18 ± 2	≥ 98	161 ± 12	[e]	600
*[^{18}F]**16***	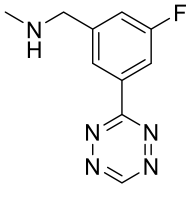	8 ± 3	5 ± 2	≥ 98	70 ± 23	50/50 [c]	1500
*[^{18}F]**17***	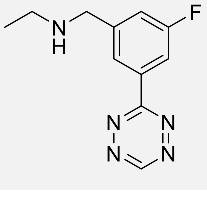	18 ± 3	17 ± 3	≥ 98	143 ± 11	[e]	600

*[^{18}F]**18***	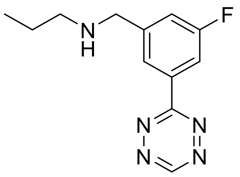	13 ± 3	12 ± 2	≥ 98	207 ± 7	[e]	1000

[a] Radiochemical conversion (RCC) and Radiochemical purity (RCP) were determined by radio-HPLC (n=3) (Section S12: Analytical Radio-HPLC and semi-preparative HPLC chromatograms of formulated [^{18}F]Tzs). [b] Radiochemical yield (RCY) was calculated from the starting amount of radioactivity received from the cyclotron and the formulated product, and was decay corrected (d.c.) (n=3). [c] MeCN/water (v/v) [d] MeOH/water 0.1% TFA [e] 15% EtOH/water 0.1% TFA. [f] [^{18}F]**5** and [^{18}F]**6** were found to decompose within minutes after formulation. Therefore, RCY and molar activity could not be measured.

Preparation of ^{111}In-labeled Tz

^{111}In-Labeled Tz **19** was used for the titration of TCO-modified antibodies (Section S7) and pre-labeling of the TCO-decorated PeptoBrush polymer (Section S9).

The labeling was performed as follows (Scheme S8). 1,4,7,10-tetraazacyclododecane-1,4,7,10-tetraacetic acid (DOTA)-PEG$_{11}$-tetrazine was dissolved (2 mg/mL) in metal-free water and stored at -80 °C before use. An aliquot of 50-100 µL (10-30 MBq) of [^{111}In]indium chloride in 0.05 M HCl was combined with 2 µL DOTA-PEG$_{11}$-tetrazine and 1 M NH4OAc buffer (pH 5.5) at a volume ratio of 1:10 was added. The mixture was shaken at 600 rpm for 5 min at 60 °C in an Eppendorf ThermoMixer C. Then, 10 mM diethylenetriamine-pentaacetic acid DTPA (volume ratio 1:10) and 2 µL 10 mg/mL gentisic acid in saline was added and the solution was shaken for an additional 5 min at 60 °C in an Eppendorf ThermoMixer C. Typically, a quantitative labeling yield and a radiochemical purity (RCP) greater than 95% were obtained with this method, as confirmed by radio-HPLC (Figure S2).

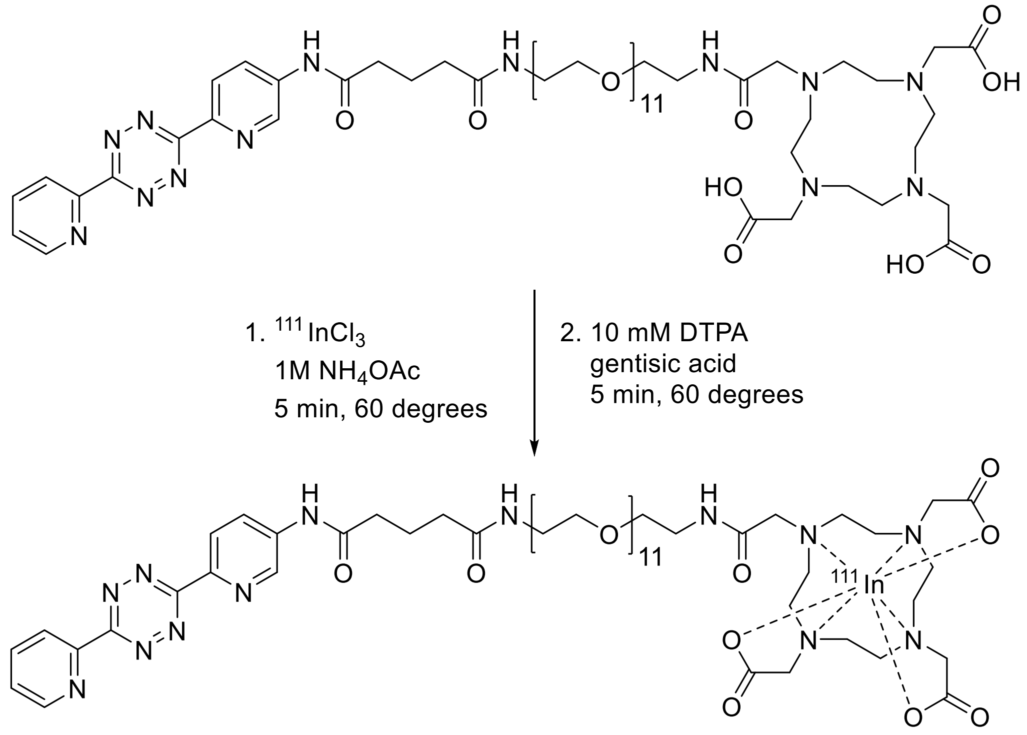

Scheme S8. Preparation of ^{111}In-labeled Tz **19**

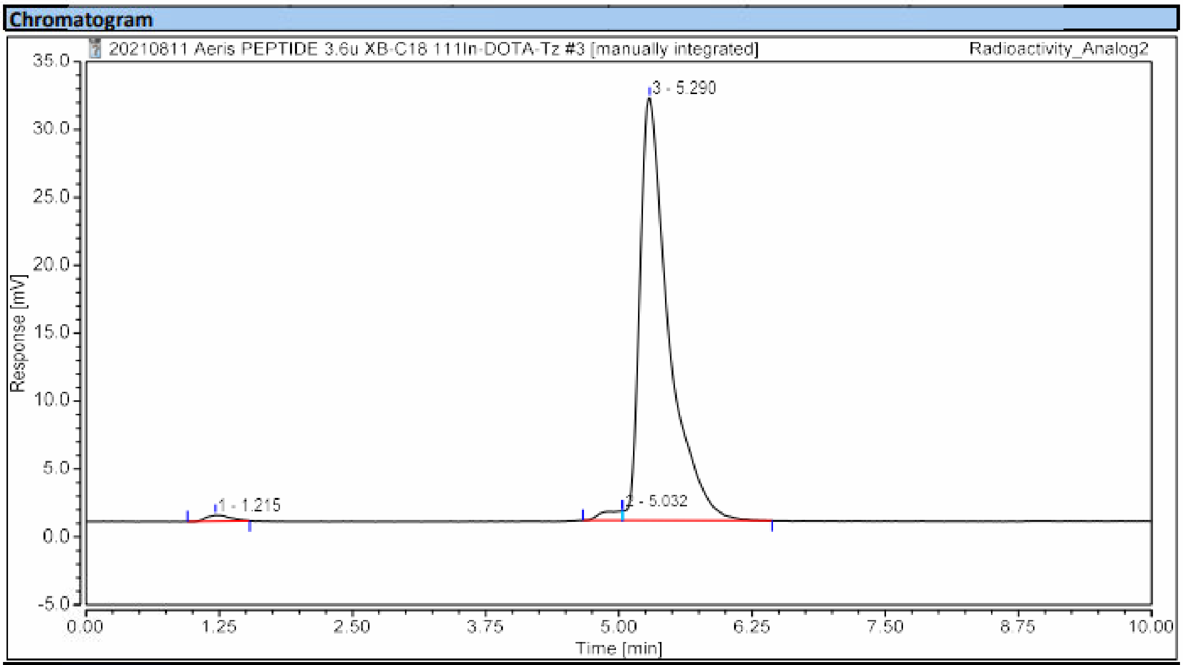

Integration Results

No.	Retention Time min	Area mV*min	Height mV	Relative Area %	Relative Height %	Amount n.a.
1	1.215	0.111	0.440	1.12	1.36	n.a.
2	5.032	0.154	0.698	1.55	2.16	n.a.
3	5.290	9.702	31.115	97.34	96.47	n.a.
**Total:**		**9.968**	**32.252**	**100.00**	**100.00**	

Figure S2. Example of radio-HPLC analysis of ^{111}In-labeled Tz **19**.

Aeris Peptide C18-XB 3.6 µm 150x4.6 mm column. Solvent A – 0.1% TFA in water, solvent B – 0.1% TFA in acetonitrile. HPLC elution method: 0-1 min – 5% B, 1-8 min - gradient from 5% B to 75% B, 8-9 min – 75% B, 9-9.5 min - back to 5% B, 9.5-10 min – 5% B; flow rate 1.5 mL/min.

Section S7: Pretargeted autoradiography

The 3D6 Aβ-antibody and tg-ArcSwe tissue sections used in this study was kindly provided by Stina Syvänen and Dag Sehlin (Uppsala University).

Modification of antibodies with TCO

Lysine residues in mAb 3D6 were functionalized with TCO-PEG$_4$-NHS. Sodium carbonate buffer (1 M, 3 µL, pH 8.0) was added to PBS (50 µL) containing mAb 3D6 (0.1-0.2 mg/mL), followed by addition of equatorial TCO-PEG$_4$-NHS (Broadpharm, BP-22418) in acetonitrile using 100−500 eq. TCO−antibody ligand molar ratio. The mixture was shaked at 600 rpm for 2.5 hours at room temperature in the dark. The solution was purified to remove the unreacted TCO-PEG$_4$-NHS with Zeba spin desalting columns (7K MWCO, 0.5 mL, 89882, Thermofisher) and eluted in PBS (pH 7.4). Final protein concentration was measured with Nanodrop (machine information).

Quantification of TCO-loading on the antibodies

Titration experiments were conducted to quantify the amount of reactive TCOs per TCO-mAb. Aliquots of TCO-mAb in PBS (5-6 µL of 0.1-1.0 mg/mL solutions) were mixed with aliquots of ^{111}In-labeled Tz **19** stock (5-6 µL) containing 2-3 eq. of Tz per expected amount of TCOs on the TCO-mAbs, and the mixed samples were shaken at 600 rpm for 1 hour at 37 °C. NuPAGE™ LDS Sample Buffer (3-4 µL, NP0007, Invitrogen) was added to each sample and the samples were heated to 70 °C and shaken vigorously for 10 minutes. After that, samples were applied onto SDS-PAGE gels (NuPAGE™ 4 to 12%, Bis-Tris, 1.0 mm, Mini Protein Gel, 12-well, NP0322BOX , Invitrogen). SDS-PAGE was run in MES-SDS buffer at 150V for 40-45 min. Separation was monitored by running SeeBlue® Plus2 Pre-Stained Protein Standard along with radiolabeled polymer samples. Control mixtures of 5 µL of ^{111}In-labeled Tz solution with 15 µL of PBS were prepared and processed in the same manner. SDS-PAGE gels were exposed to phosphor storage screens and read by a Cyclone Storage Phosphor System (PerkinElmer Inc.). Quantification of plate readings was done with Optiquant software (version 3.00, Packard Instruments Co). Radioactivity found in the section of the gel corresponding to molecular weights of 38 kDa and above (using SeeBlue standards as reference) was considered mAb-bound. The percentage of the total mAb-bound activity per lane of the SDS-PAGE gel was used to calculate the number of reactive TCOs per mAb using the formula below.

$$N_{TCO} = \frac{\%bound \times n_{Tz}}{RCP_{Tz} \times n_{mAb}},$$

Where N_{TCO} is the number of reactive TCOs per mAb;

%bound is the percentage of mAb-bound activity;

n_{Tz} is the amount of ^{111}In-labeled Tz in the aliquot;

n_{mAb} is the amount of TCO-mAb in the aliquot;

RCP_{Tz} is the radiochemical purity of ^{111}In-labeled Tz determined by radio-HPLC.

Pretargeted Autoradiography

Frozen brains of old (~18 months of age; 25-40 g body weight) male C57BL/6 mice and tg-ArcSwe model harboring the Arctic (AβPP E693G) and Swedish (AβPP KM670/671NL) mutations (provided by Uppsala University) were cut into halves along the sagittal symmetry plane [11]. Each half was mounted, lateral side up, on the cryostat sample holder pretreated by Tissue-Tek OCT Compound, and fixed by freezing in the cryostat. After fixing, brains were cut at −22 °C into sagittal slices 20 μm thick using a Leica microtome, and the slices were thaw-mounted on Superfrost (70 × 22 mm, Fischer) adhesive slides. Only slices containing both cortex (specific binding) and cerebellar regions (representing nonspecific binding) were used. The slices were allowed to dry and were then put into storage boxes and kept at −80 °C until they were used.

On the day of the experiment, the slides with mounted brain sections were taken out of storage and allowed to come to room temperature for 30 min. After that, a PAP pen was applied around the sections and 600 μL 1% BSA in PBS (pH 7.4) with 0.05% Tween 20 was added and incubated at room temperature for 30 minutes in incubation boxes. Afterwards, the BSA solution was removed and 600 μL of PBS (pH 7.4) with 0.05% Tween 20 was applied and incubated at room temperature for 5 minutes. The preincubation buffer was removed and 600 μL of TCO-3D6 (0.006 μg/mL and 0.06 μg/mL) in PBS (pH 7.4) with 0.05% Tween 20 was applied and incubated overnight at room temperature in a moist incubation box. On the second day, the TCO-mAb solution was removed and the slides were washed in cold PBS (pH 7.4) (3x15 min) and cold demineralized water (1x30 sec). Then, 600 μL 20 nM ^{18}F-Tz solution was applied to all sections and incubated for 60 minutes. Afterwards, the radioactive solution was removed and the slides were rinsed in demineralized water (1x30 sec) and dried by a flow of compressed air. After drying the plates, the slides were exposed on phosphor storage screens for 1-12 hours, depending on the amount of radioactivity on the slide.

Afterwards, the phosphor storage screens were read by a Cyclone Storage Phosphor System (Packard Instruments Co). Quantification of plate readings was done with Optiquant software (version 3.00, Packard Instruments Co) and ImageJ by drawing regions of interest on the cortex and cerebellum manually (Fig. S3). Excel was used to process the data and calculate cortex/cerebellum exposure ratios for slides incubated with 0.006 μg/mL TCO-3D6 (Table 2 in the main manuscript).[12, 13] Images from slides incubated with 0.06 μg/mL were used for presentation purposes (Figure 3B of the main manuscript).

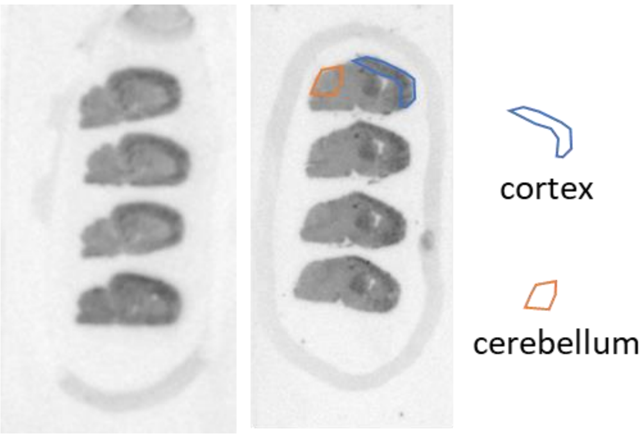

Figure S3. Representative pretargeted autoradiography images for designed ^{18}F-Tzs.

Cortical and cerebellar regions are highlighted on one of the slices.

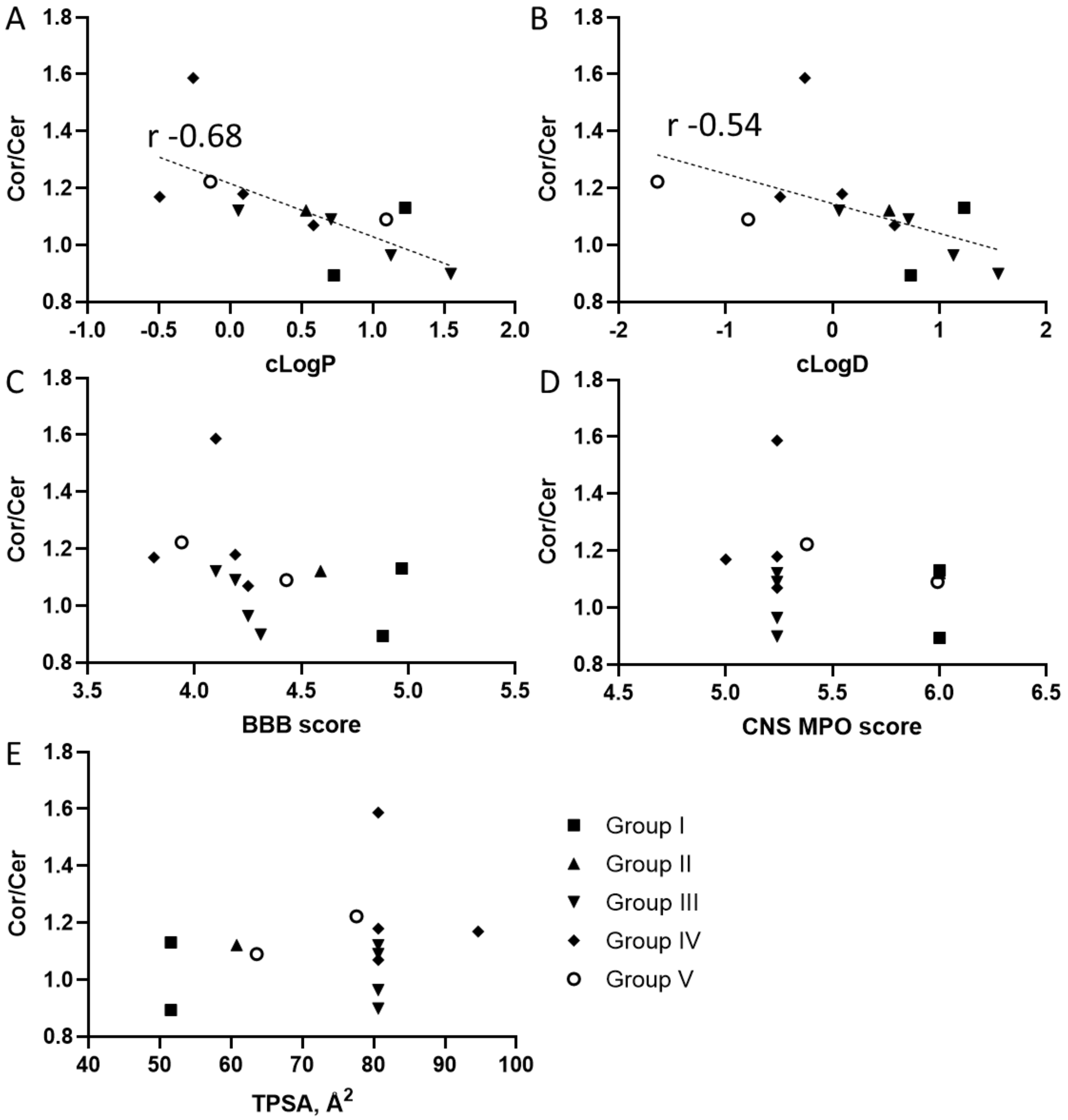

Figure S4. Correlations of in vitro cortex-to-cerebellum (Cor/Cer) uptake ratios with in silico properties of designed Tzs.

Extrapolation of Cor/Cer ratios for Group V Tzs

At the time that the pretargeted autoradiography data were examined and aggregated, the Cor/Cer ratio was only determined for the most and the least hydrophilic of the amino-Tzs (Group V, **15**-**18**), [^{18}F]**15** and [^{18}F]**18**, respectively. As there was a robust correlation of Cor/Cer ratios with cLogP across Tz classes (Fig. S4A), for ranking purposes we decided to extrapolate the expected Cor/Cer ratios for the remaining two ^{18}F- Tzs using the obtained linear trend equation for all evaluated ^{18}F-Tzs. The extrapolated Cor/Cer ratios for [^{18}F]**15** and [^{18}F]**18** were close to the experimental values

(1.24 vs 1.22 and 1.02 vs 1.09, respectively), and the experimental values did not break the negative correlation trend predicted from extrapolation (Fig. S5).

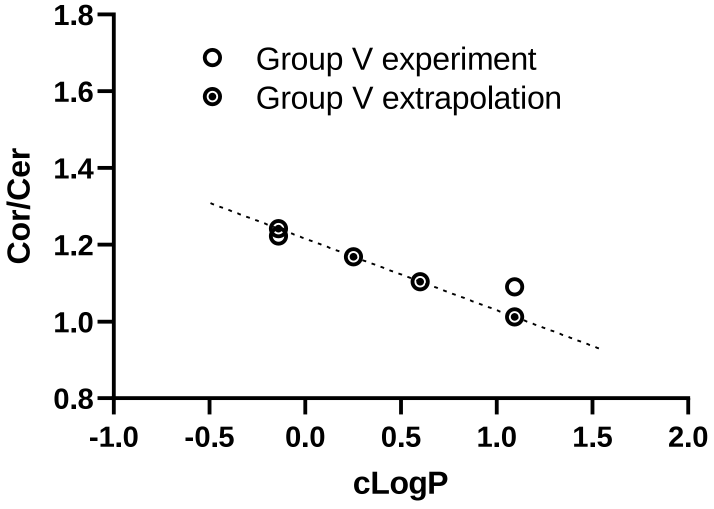

Figure S5. Extrapolated Cor/Cer values for Tz **15-18** plotted against general Cor/Cer-vs-cLogP trend for all evaluated Tzs. The general trend is shown as a dashed line.

Section S8: In vivo PET

Animals

Long-Evans female rats weighing 200 - 300 g (Charles River, Calco, Italy), were housed in a cages of 2-3 rats per cage and kept in a climate-controlled facility with a 12-hr light/dark cycle. The cages had environmental enrichment (nest box, biting stick). All rats were fed *ad libitum* with commercial breeding diet (1310 FORTI- Avlsfoder, Brogaarden, Altromin International) and had free access to water. All animal experiments were performed in accordance with the European Commission's Directive 2010/63/EU for animal research and with approval from The Danish Council for Animal Ethics (license numbers 2017-15-0201-01283 and 2017-15-0201-01375) together with the Department of Experimental Medicine, University of Copenhagen.

PET procedure

The scans were performed on the Siemens HRRT (High-Resolution Research Tomography) (CPS Innovations/Siemens, Malvern, PA, USA). On the day of experiment, the rats were weighted and then transported to the scanner at least 2 h before the scan. Anesthesia was induced using 3% isoflurane in oxygen flow. All rats were placed in a 2 x 2 custom made rat holder, enabling simultaneous scanning of four rats [14-16]. While in the custom-made rat holder, the rats were kept under anesthesia with a constant flow of isoflurane (~2% isoflurane in oxygen). The rats were canulated in the dorsal or lateral tail vein with BD Neoflon 24G vein catheter and flushed with a solution of 4 unit/mL heparin in saline. The rats were placed in the scanner and kept warm using an infrared lamp or heating pads and checked for respiration throughout the entire scan. A rotating point source ^{137}Cs transmission scan [17] was carried out before or after each emission scan and the emission scan was started at the time of injection.

Image reconstruction

From the images conducted from the HRRT scans, could an ordinary Poisson 3D subset reconstruct the images in an expectation maximization (OP-OSEM3D) algorithm with point spread function, containing a point spread algorithm, resulting in 207 planes consisting of 256 x 256 voxels (1.22 x 1.22 x 1.22 mm). The transmission attenuation map was regenerated using maximum posteriori

algorithm. The 90 min emission PET scans were converted into 35 dynamic frames (6 x 10, 8 x 30, 5 x 60, and 16 x 300 sec) and filtered with 0 mm.[16]

Data analysis

To analyze the data, the program PMOD 3.7 (PMOD Technologies, Zürich, Switzerland) was used. A standard rat brain MRI atlas was used to extract the preferred volumes of interest (VOIs). The time-activity curves (TACs) from all the collected VOIs were converted into percentage injected radioactivity dose per mL tissue (ID%/mL).

For the examination of radioactivity uptake in peripheral tissues, VOIs were drawn around urinary bladder and liver using the hot contouring tool and TACs were generated as described above. Maximum intensity projection (MIP) images were also prepared using summed PET data for 0-15 min post-injection.

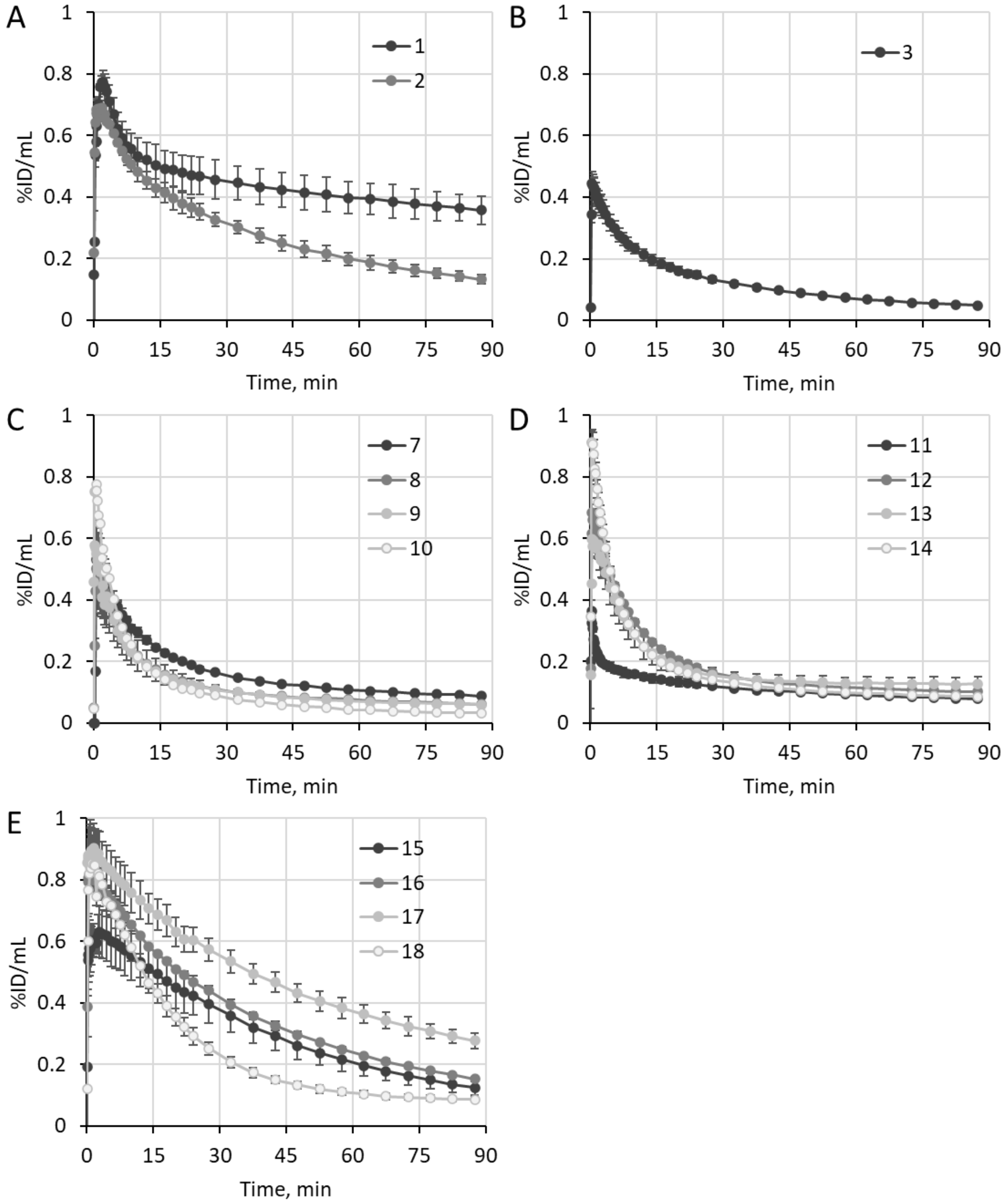

Figure S6. Average whole brain time-activity curves (TACs) for ^{18}F-Tzs in healthy rats.

A – Group I, B – Group II, C – Group III, D – Group IV, E – Group V.

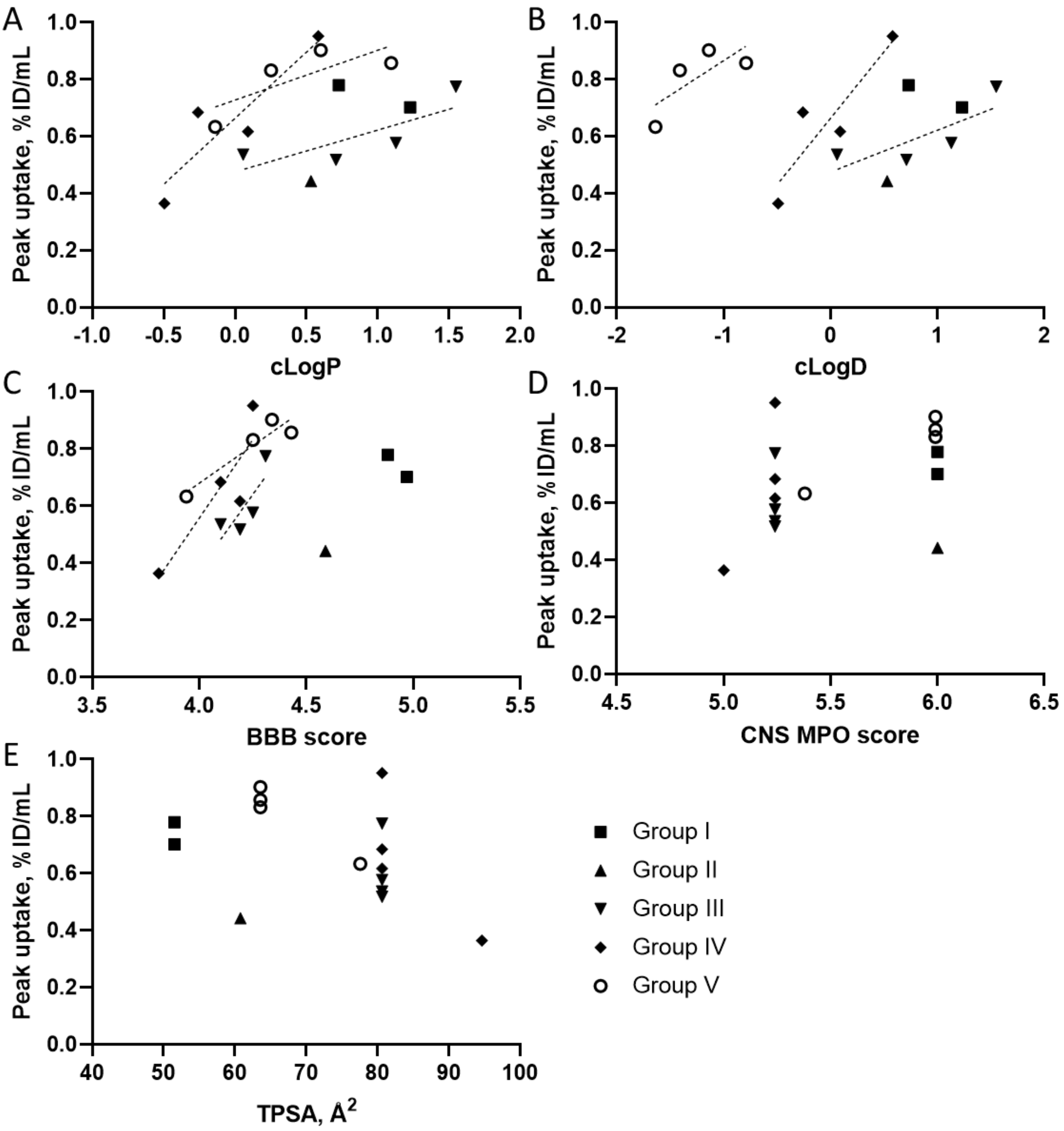

Figure S7. Correlations of in vivo peak brain uptake with in silico properties of designed Tzs.

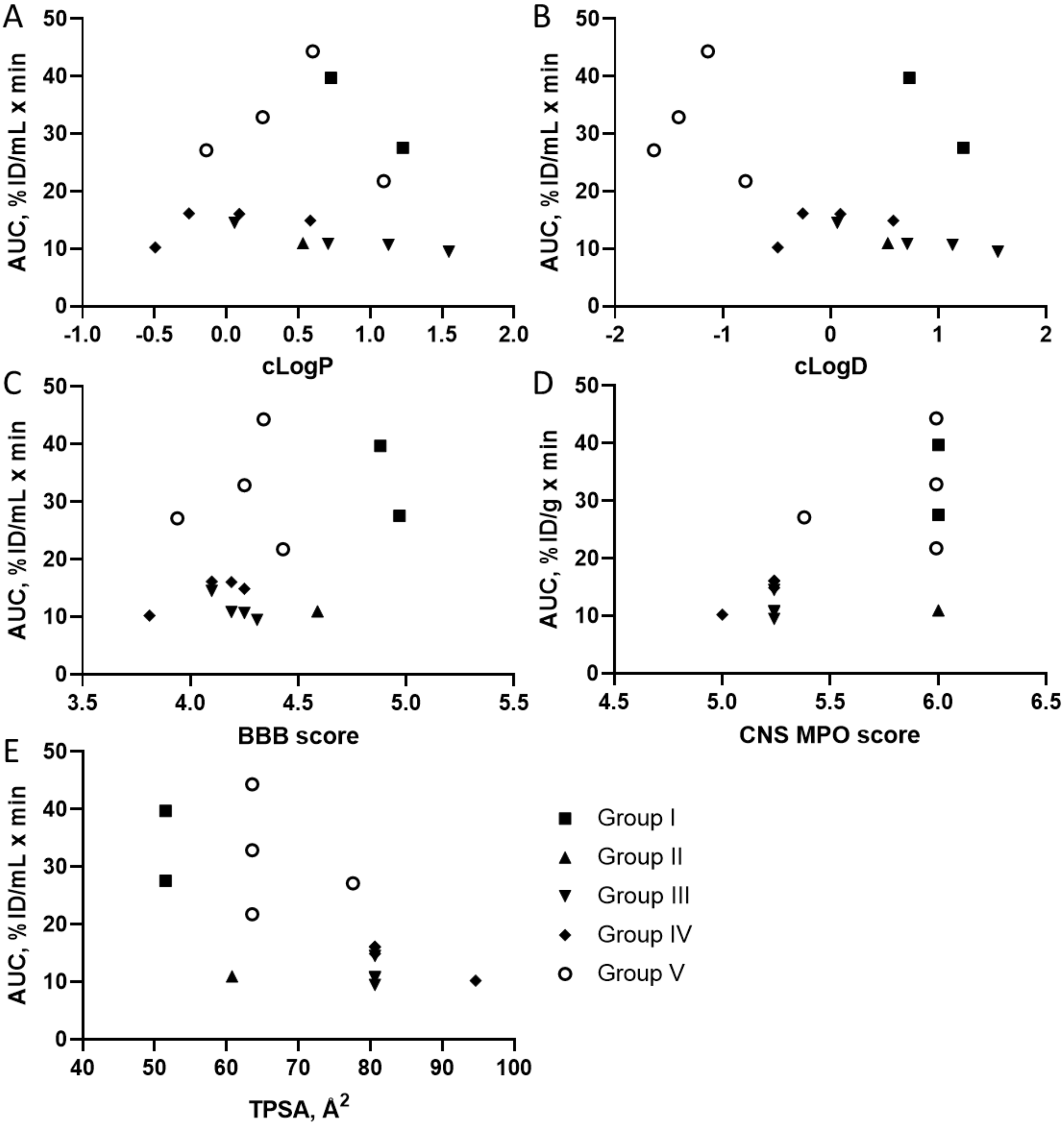

Figure S8. Correlations of AUC values for in vivo brain TACs with in silico properties of designed Tzs.

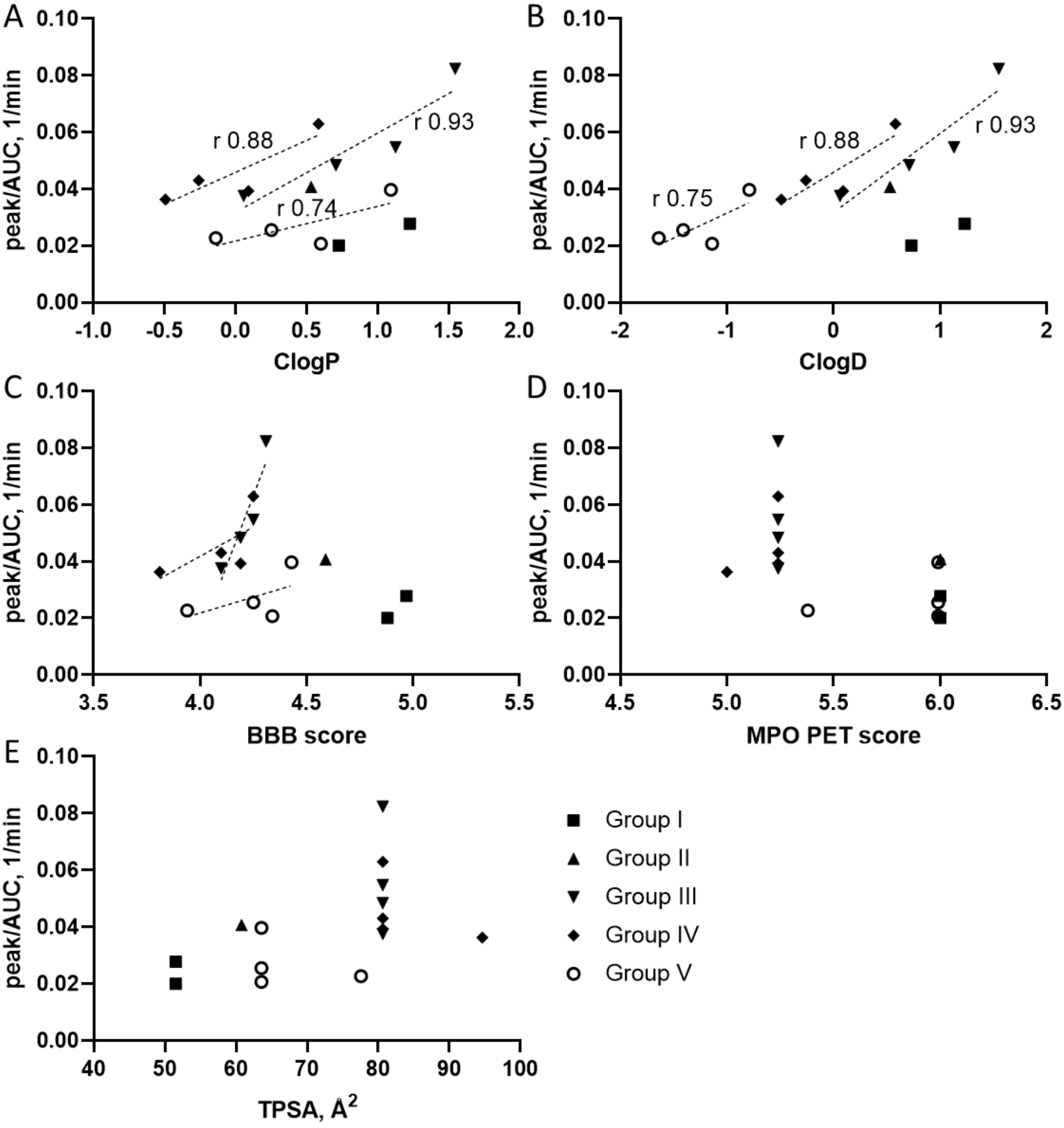

Figure S9. Correlations of in vivo peak/AUC ratios with in silico properties of designed Tzs.

Table S2. In vivo screening of ^{18}F-Tzs healthy rats: peak brain uptake, AUC values peak/AUC ratios.

**^{18}F-Tz**	**Peak brain uptake, %ID/mL**	**AUC, %ID/mL×min**	**Peak/AUC, min^{-1}**
[^{18}F]**1**	0.78±0.03	38.9±4.6	0.020±0.002
[^{18}F]**2**	0.70±0.01	25.3±2	0.028±0.002
[^{18}F]**3**	0.44±0.04	10.9±0.8	0.041±0.005
[^{18}F]**7**	0.54±0.06	14.3±0.3	0.038±0.004
[^{18}F]**8**	0.52±0.13	10.7±1.5	0.048±0.014
[^{18}F]**9**	0.58±0.08	10.5±0.1	0.055±0.008
[^{18}F]**10**	0.77[a]	9.4[a]	0.082[a]
[^{18}F]**11**	0.36±0.04	10.0±0.6	0.036±0.004
[^{18}F]**12**	0.68±0.03	15.9±0.2	0.043±0.002
[^{18}F]**13**	0.62±0.01	15.8±2.2	0.039±0.005
[^{18}F]**14**	0.95±0.01	14.7±0.7	0.065±0.003
[^{18}F]**15**	0.63±0.08	26.8±3.6	0.024±0.004
[^{18}F]**16**	0.83±0.02	32.5±0.6	0.026±0.001
[^{18}F]**17**	0.90±0.08	43.7±3.4	0.021±0.002
[^{18}F]**18**	0.86±0.04	21.6±1.7	0.040±0.004

Data are presented as means±SD (n = 2-4).

[a] No SD is given, data from a single animal.

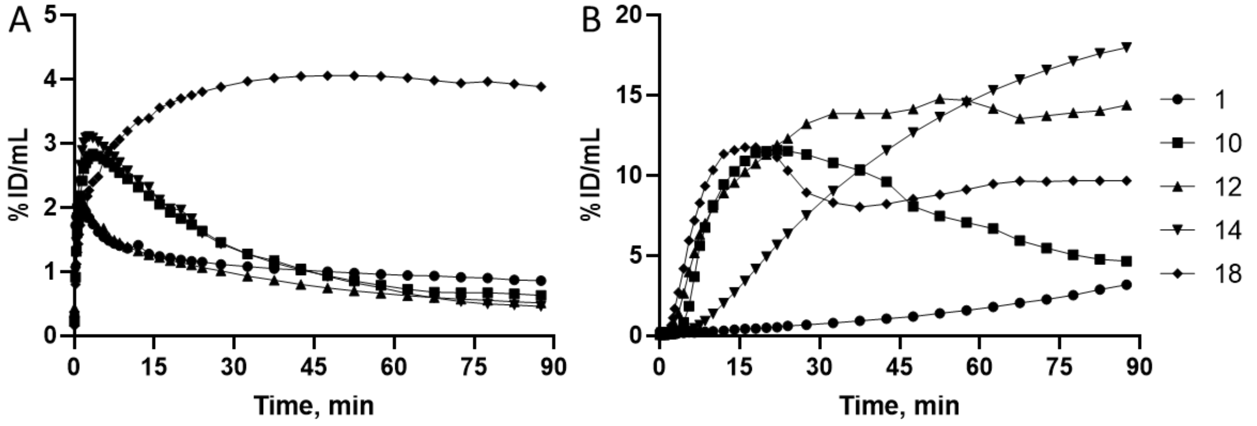

Figure S10. Liver (A) and urinary bladder (B) TACs for [^{18}F]**1**, [^{18}F]**10**, [^{18}F]**12**, [^{18}F]**14** and [^{18}F]**18**. Data from representative animals are shown.

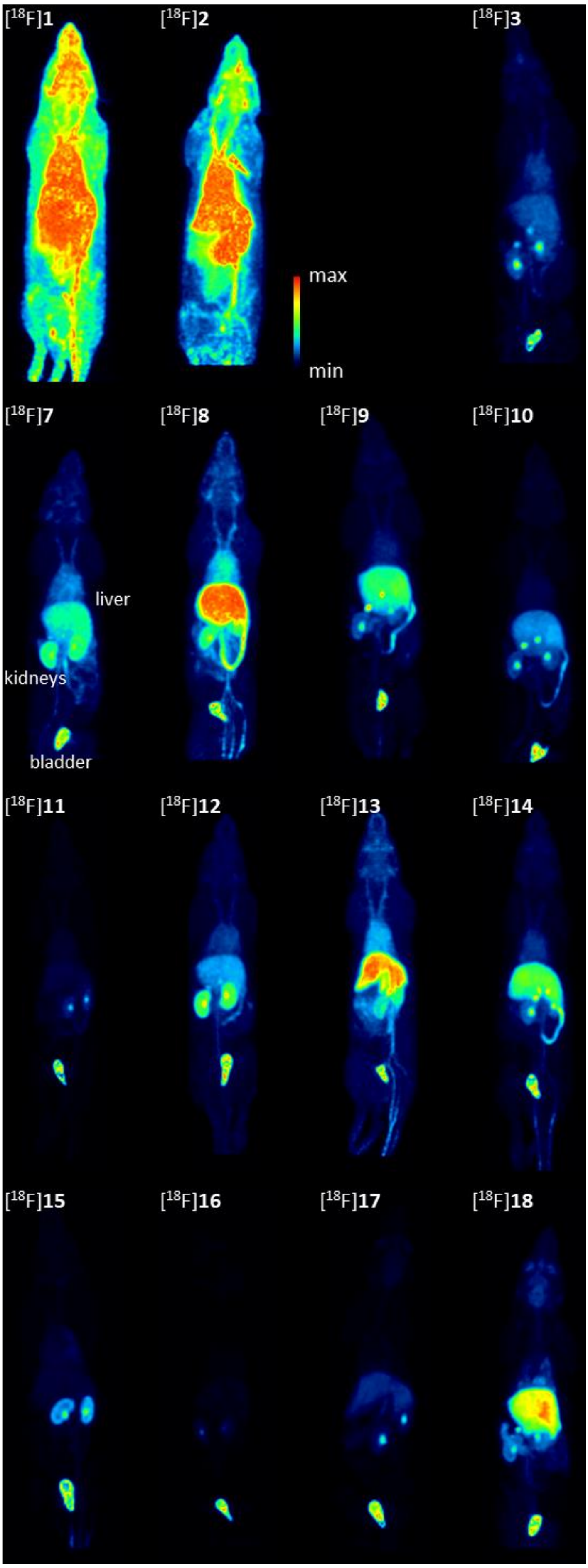

Figure S11. Radioactivity uptake in peripheral tissues for Tzs [^{18}F]**1-18**. Images are normalized maximum intensity projections (MIPs) of PET data summed for 0-15 min post-injection. Data from representative animals are shown.

Section S9: Intracerebral TCO-polymer injection (ic-TCO) model

PeptoBrush TCO-polymer was prepared as described previously [18]. Molecular weight of the polymer was 183000 g/mol, and it carried 27 TCOs per strand (1 TCO per ca. 6.7 kDa).

PeptoBrush labeling with ^{111}In for SPECT/CT imaging

PeptoBrush polymer solution (1 mg in 0.5 mL 10 mM PBS) was mixed with ^{111}In-Tz solution (400 MBq ^{111}In bound to 60 µg Tz in 1 mL of 0.2M ammonium acetate buffer pH 4.5). The mixture was left standing for 10 min and then applied onto a PD-10 column (14.5×50 mm, Cytiva) pre-equilibrated with phosphate buffer (0.1M pH7.2). The column was eluted with phosphate buffer, fractions from 2.0 to 3.5 mL cumulative elution volume were combined and concentrated with the aid of ultrafiltration spin columns (Vivaspin 500, 50 kDa mol weight cutoff, Sartorius). Activity yield of [^{111}In]In-PeptoBrush was 292 MBq in 120 µL solution.

PeptoBrush labeling with ^{111}In for pretargeted PET imaging

Aliquot of PeptoBrush polymer solution (20-50 µL of 50 mg/mL in 10 mM PBS) was quickly mixed with an equal-volume aliquot of ^{111}In-Tz solution (200 kBq/mL in 10 mM PBS, <0.01 eq Tz vs TCO in the TCO-polymer aliquot) and left standing at room temperature for 5 min. Polymer concentration in the final solution was 25 mg/mL. HPLC analysis showed full consumption of ^{111}In-Tz.

Intra-cranial polymer depositions in the rat brain

Female Long-Evans WT rats (300-350 g body weight, 9-15 weeks of age) (Janvier) were used in this study. The animals were held under standard laboratory conditions with 12-h light/12-h dark cycles and ad libitum access to food and water. All animal experiments conformed to the European Commission's Directive 2010/63/EU with approval from the Danish Council of Animal Ethics

(Journal no. 2017-15-0201-01375) and the Department of Experimental Medicine, University of Copenhagen.

The animals were acclimatized in the surgery room for at least 1 h. Analgesia was provided with carprofen (Rimadyl, Zoetis, NJ, USA) 5 mg/kg, subcutaneous (SC), 45 min before the surgery and post-operative. Anaesthesia was induced with 3-3.5 % isoflurane in oxygen and maintained through surgery with 1.5–2 % isoflurane in oxygen. The rats were fixed on a stereotaxic apparatus (Kopf Instruments, Tujunga, CA, USA) with the incisor bar set 3.3 mm below the level of the ear bars. After installation of local anaesthesia, an incision was made on the scalp, and two bur-holes were drilled on either side of the skull using a dental micromotor and round bur (0.5 mm). PeptoBrush polymer solution (25 mg/mL in PBS) was drawn into a 10 µL syringe with a 33 g needle (World Precision Instruments, Sarasota, FL, USA). 4 µL containing 100 µg polymer (15 nmol TCO) were infused into the right or left striatum (coordinates: AP= 1.1 mm, ML= ±3.3 mm, DV= 5 mm) relative to the bregma while a control injection of 4 µL PBS was injected in the contralateral hemisphere. The infusion was delivered at 150 nL/minutes driven by an infusion pump (World Precision Instruments, Sarasota, FL, USA), followed by a 7 min pause prior to a slow withdrawal of the syringe needle. The incision was sutured back. After recovery from anaesthesia, rats were returned to the recovery cage and housed alone until the scan.

Gadolinium-enhanced MRI to validate the integrity of the BBB:

Apart from the animals used for the PET scans, four animals (n = 4) underwent a gadolinium-enhanced MRI scans to validate the integrity of the BBB post-intra-cranial-injection. Conditions includes one control animal (no surgery), one animal 6h post-injection, one animal 18h post-injection, and one animal 30h post-injection. Animals were scanned in a 9.4 Tesla 30 cm bore system MRI scanner (Biospec 94/30, Bruker Biospin, Germany) (Gradient: B-GA12S, 240mT/m) with 2 x 2 element rat brain RF coil array (T10324_V3, Bruker Biospin, Ettlingen, Germany) combined with a volume resonator for transmit (iD 86 mm, model: T12054_V3, Bruker Biospin, Ettlingen, Germany). Before the scans, animals were anesthetized with 4 % isoflurane in air:oxygen mixture and maintained at 1.5-2 % isoflurane during the period of the scan. The animals were maintained at 37°C with a circulating warm-water-based heating system. Respiration rate and temperature can be monitored using a remote monitoring system (SA Instruments Inc., Stony Brook, NY, USA). The animals underwent two T_1-map scans: pre- and post-gadolinium. The protocol for the T_1-map scan was RAREVTR: TR = 9000, 2400, 1480, 940, 650, 400, 200, 160 ms, effective TE = 10 ms, rare factor = 4, FOV = 25.6 x 25.6, matrix = 128 x 128, slice thickness = 1 mm, number of slices = 26. After the pre-gadolinium T_1-map scan, animals received gadolinium IV (0.1 mmol/kg, 0.5M ProHance®

[gadoteridol], Bracco Imaging Scandinavia AB, Gothenburg, Sweden) through a tail-vein catheter and were rescanned 8 mins later with another T1-map scan. PMOD 3.7 (PMOD Technologies, Zürich, Switzerland) was used to visualize and make the representative MR images. The post-gadolinium T1-map was co-registered and resliced to the pre-gadolinium T1-map using PMOD. A %-difference map (ΔT1-map) was created from the resliced post-gadolinium and pre-gadolinium T1-maps (Equation 1).

$$\Delta T_1 \text{map } (\%) = \left(\frac{Post\ Gd - Pre\ Gd}{Pre\ Gd}\right) \times 100 \qquad \text{(Equation 1)}$$

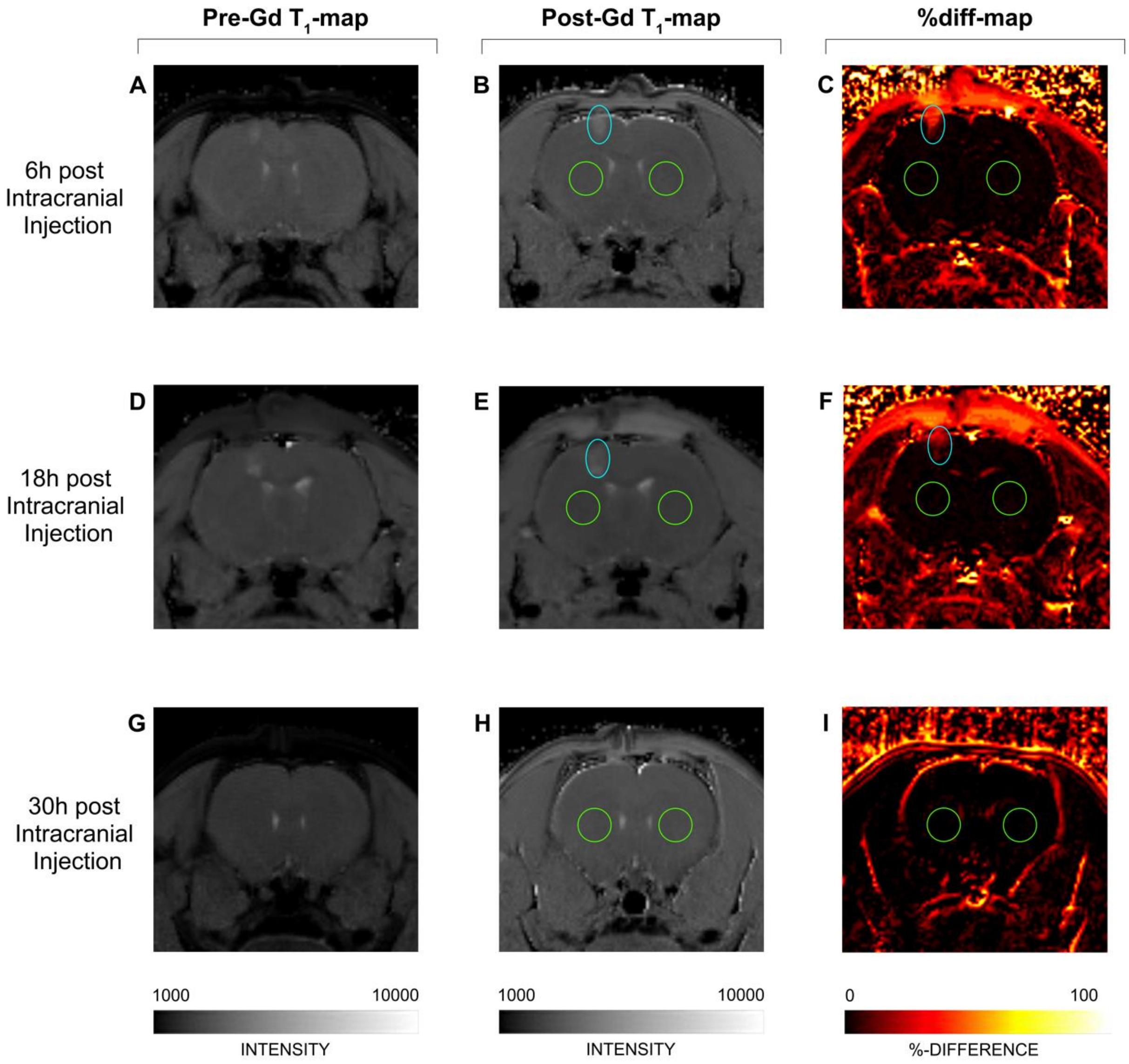

Figure S12. Representative pre- (A, D, G) and post- (B, E, H) gadolinium (Gd) enhanced T_1-map post intracranial injections of either polymer or saline in striata on both sides. ΔT_1 maps (C, F, I) are shown as % difference, i.e., % (post-Gd - pre-Gd)/pre-Gd.

Three animals scanned at different time-points including 6-hours, 18-hours, and 30-hours post intracranial injection. The post-Gd-enhanced T_1-map shows no uptake of gadolinium in the injection sites (approximately marked in red) showing that the injections do not cause a leakage in the BBB, nevertheless hematomas are visible in two animals at the site of the needle penetration (marked in dark blue) but no difference is noted in the pre- and post-Gd-enhanced T_1-map. No visible difference is also noted on the right and left injection sites.

SPECT/CT acquisition with ^{111}In-labeled PeptoBrush to confirm polymer retention at injection site

The Vector4CT (Milabs) system with a high energy ultra-high resolution rat 1.8mm pinhole collimator (HE-UHR-RM 1.8mm ph) was used for both CT and SPECT acquisition. The rat was moved to the imaging bed and a full-body CT scan was performed followed by a SPECT acquisition covering the brain and upper spinal cord. The full-body CT scan was acquired with 55 kV tube voltage, 75 ms exposure time, x11 binning and 360 projections with a step angle of 0.75 degrees over 9 minutes. The full-body SPECT scan of 1 frame acquired over 20 minutes was obtained. Head scans were acquired with 30 bed positions and full-body scans with 67 bed positions and the detection peak energy was set to 1200 keV. Scatter and CT-based non-uniform attenuation correction were performed, and the images were reconstructed using a Similarity-Regulated Ordered Subsets Estimation Maxization (SROSEM) with a voxel size of 0.8 mm, and 5 iterations. A 0.8 FWHM (mm) gaussian filter was added post reconstruction. Only decays at the [^{111}In]In photopeaks (176 (+/- 11%) keV and 277.2 (+/- 20%), background at 4% and 10% outside each photopeak window) were used for the reconstruction. CT and SPECT data was reconstructed with MiLabs reconstruction software (version 10.16, Milabs).

Pretargeted PET imaging and gamma counting

PET scanning was performed as described in Section S8. After the scan, the rats were sacrificed by decapitation, brains were extracted and cut into two halves along the sagittal symmetry plane. Left and right brain halves were put into gamma counting tubes, left at 4°C for 24 hours to ensure full

decay of ^{18}F and then counted on the gamma counter to measure the amount of ^{111}In activity in the brain halves. Aliquots of ^{111}In-labeled PeptoBrush solution used for intracranial injections (4 µL, same as injected volume) were counted along with brain halves to calculate the fraction of injected ^{111}In activity retained in the brain halves.

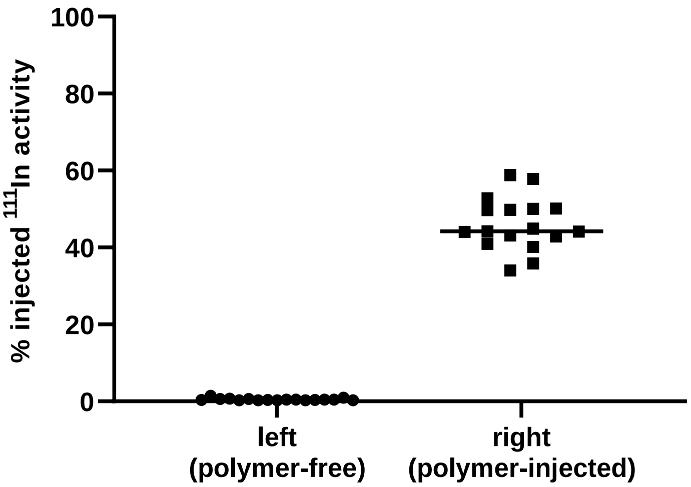

Figure S13. Ex vivo ^{111}In counts of brain halves from ic-TCO rats obtained 1 day after the PET imaging experiments.

The rats had total body volume of 200-300 mL. Therefore, even if some polymer was indeed washed out from the striatum, local TCO concentration in the striatum can still be assumed >1000-fold higher than elsewhere in the body.

Data analysis

Images were reconstructed and data was analyzed as described in Section S8. TACs for left and right striatum VOIs (caudate-putamen + globus pallidus) were extracted and expressed in %ID/mL. Relative and absolute imaging contrast was calculated from time-averaged ^{18}F-activity uptake on two sides of the striatum at 60-90 min post-injection (the last 30 min of the scan). Relative imaging contrast was defined as the ratio between ^{18}F-uptake in the TCO-polymer injected vs the polymer-free striatum, while the absolute contrast was defined as the difference between the uptake values.

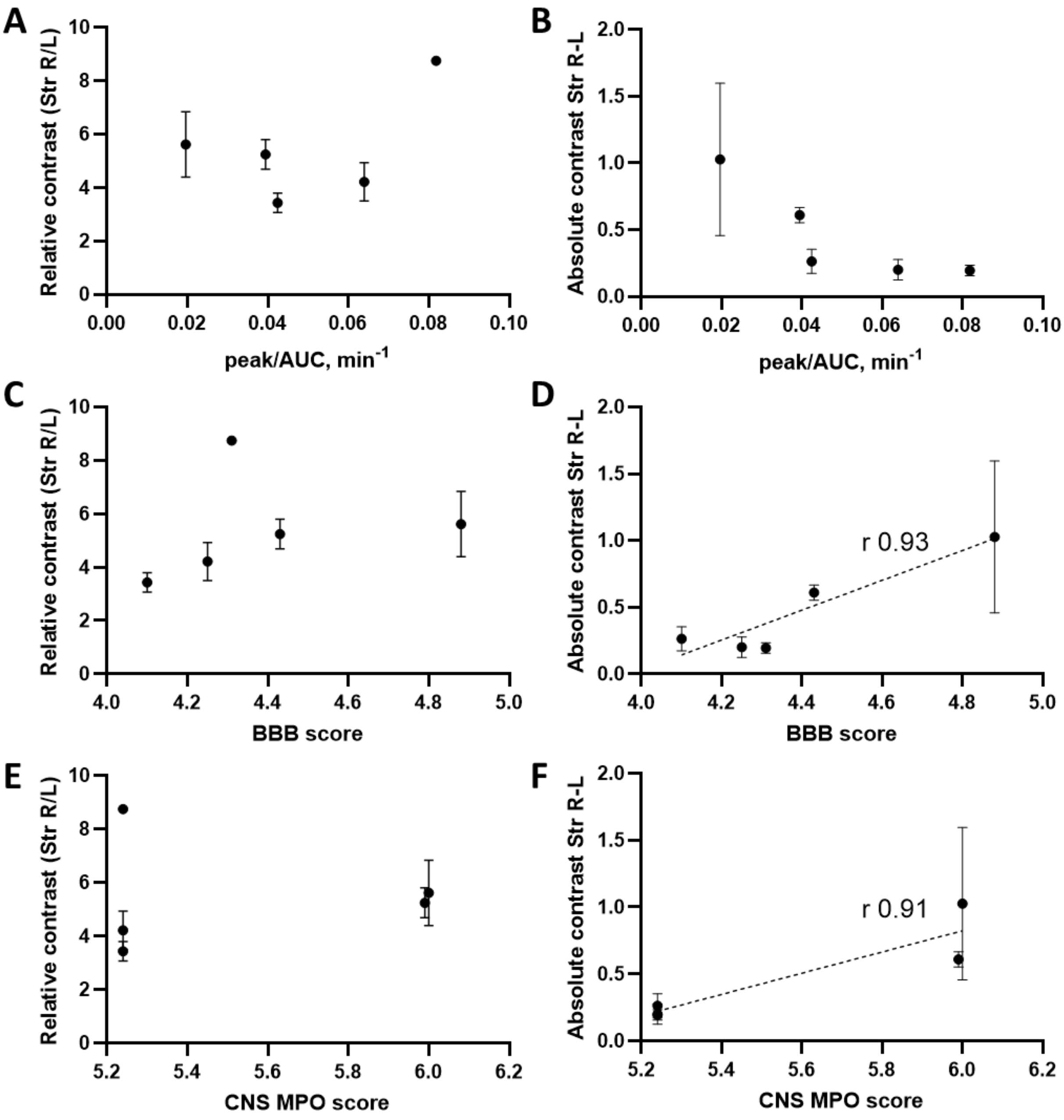

Figure S14. Correlations of relative and absolute imaging contrast in the ic-TCO model with peak/AUC ratios (A,B), BBB score (C,D) and CNS MPO score (E,F).

Section S10: In vivo metabolism of ^{18}F-Tzs in healthy rats

Healthy female Sprague-Dawley rats were anesthetized with a mixture of isoflurane/air (inhalation anesthesia, 5% ratio during induction, 2% at maintenance). Body temperature of the anesthetized animals was maintained by placing them on heating mats. For each rat, their femoral artery on one body side and their dorsal or lateral tail vein were cannulated with PE50 tubing (AD Instruments, USA) and BD Neoflon 24G vein catheter, respectively. Catheters were flushed with a solution of 4 unit/mL heparin in saline. The cannulations took 40–50 min.

After the cannulation, [^{18}F]Tz in 0.1M pH 7 phosphate buffer (0.3-0.5 mL) was injected through the venous catheter, and arterial blood samples of 0.10–0.15 mL were withdrawn through the arterial cannula right before [^{18}F]Tz injection (spiking sample) and at 1, 2, 5, 10, 15, 20, 40, 60 and 90 min after [^{18}F]Tz injection.

Whole blood samples were centrifuged at 3500 g for 5 min, and 25 µl supernatant (plasma) aliquots were withdrawn and deproteinated with 75 µl of ice-cold acetonitrile. From the resulting 100 µl, 5-15 µl portions (i.e. 5-15% of volume) were withdrawn for thin layer chromatography analysis (radio-TLC), to assess radiometabolite content. Radioactivity of plasma aliquots was measured with a gamma counter.

Radio-TLC was performed on silica plates. Plasma obtained from the spiking samples (before [^{18}F]Tz injection) was spiked with [^{18}F]Tz solution used for injection and worked up in the same manner. Spiking samples were run on radio-TLC plates along with the other samples to confirm the identity of the parent compound in plasma.

Table S3. TLC conditions for ^{18}F-Tz radiometabolite analysis

**Tz**	**Eluent**	**Parent R_f**
**1**	heptane/EtOAc 4/1	0.35
**10**	heptane/EtOAc 2/3	0.82
**12**	heptane/EtOAc 1/2	0.50
**14**	heptane/EtOAc 2/3	0.66
**18**	EtOAc/methanol/triethylamine 90/10/0.5	0.40

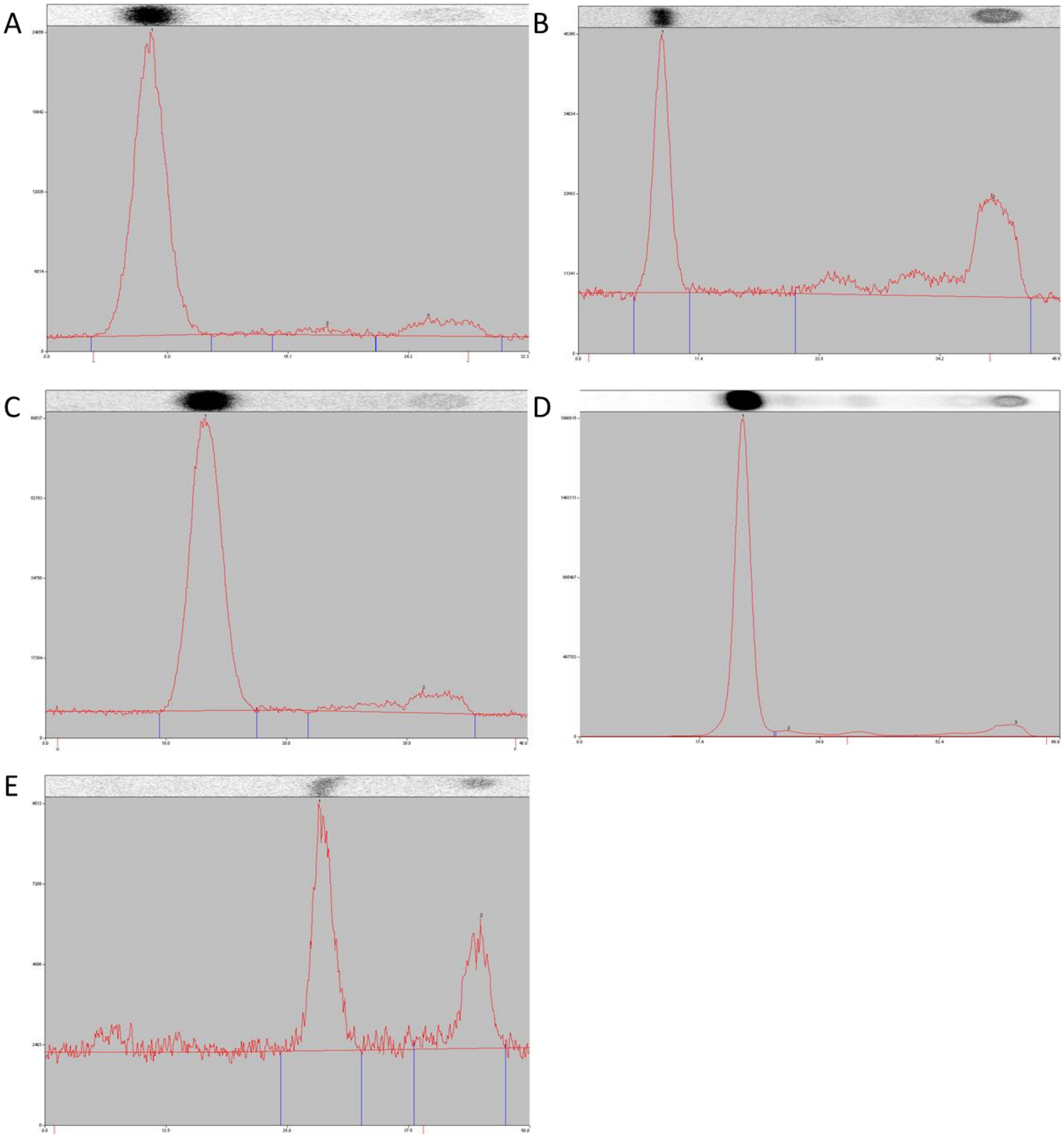

Figure S15. In vivo metabolism of ^{18}F-Tzs in healthy rats: examples of radio-TLC chromatograms.

A – [^{18}F]**1**, B – [^{18}F]**10**, C – [^{18}F]**12**, D – [^{18}F]**14**, E – [^{18}F]**18**.

Major peak on the left is the parent Tz.

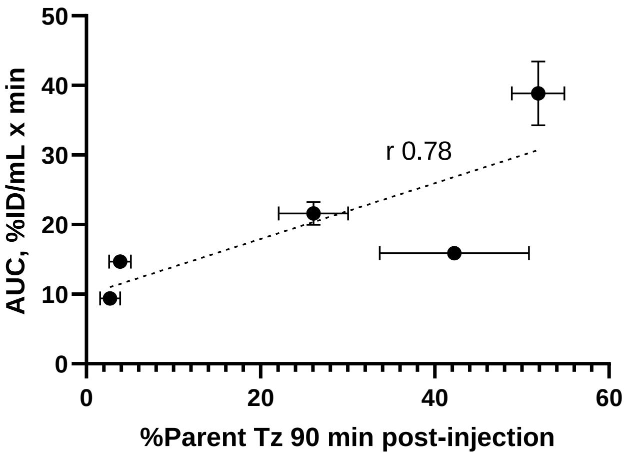

Figure S16. Correlation between metabolic stability and brain AUC for selected ^{18}F-Tzs.

Brain AUC values are taken from in vivo screening in healthy rats (Section S8). Error bars represent standard deviations.

Section S11. Statistical analysis

All measurements are reported as means±SD unless stated otherwise. Correlation coefficients were calculated with GraphPad Prism 9. P-values below 0.05 were considered statistically significant. No correction for multiple comparisons was performed.

Section S12: Analytical Radio-HPLC and semi-preparative HPLC chromatograms of formulated [^{18}F]Tzs

Radio-HPLC traces of the formulated [^{18}F]Tzs synthesized following the general procedure for the automated synthesis of the tetrazines as described in Section S6, with UV of ^{19}F-references overlaid are shown below. Unless stated otherwise, analytical HPLC for all samples were run on the Thermo Fisher UltiMate 3000 system with a C18 column (Luna 5 µm C18(2) 100 Å, 150 mm × 4.6 mm) using the following method: Eluents: A, H_2O with 0.1% TFA; B, MeCN with 0.1% TFA. Gradient from 100% A to 100% B over 12 minutes, back to 100% A over 3 min, flow rate 2 mL/min. UV absorption was recorded at λ = 254 nm on a UVD 170U detector. The solid red line indicates the radioactivity trace and the solid black line indicates the UV trace for the cold reference compound. Semi-preparative HPLC was performed on a Luna 5µ C18(2) (100Å 250 x 10 mm) column using H_2O/MeCN (v/v) mixtures as eluent (Section S6), flow rate 4 mL/min in the Scansys module.

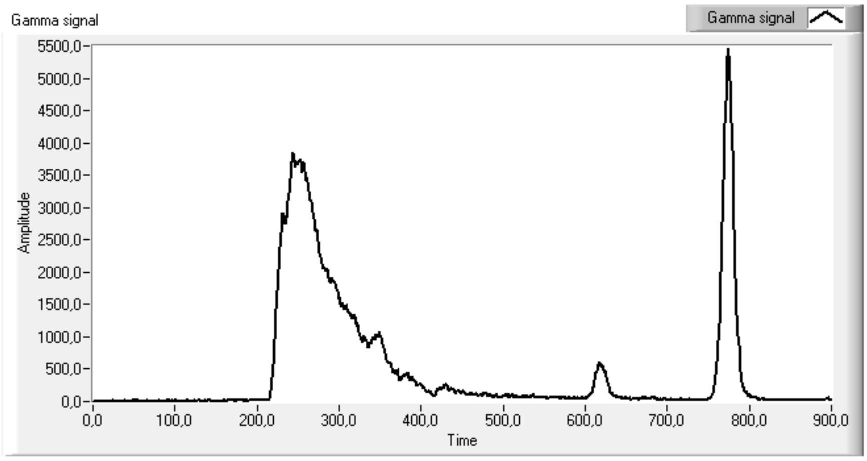

Figure S17. *Semi-preparative HPLC chromatogram for* ***[^{18}F]1*** *(R_t = 790 sec).*

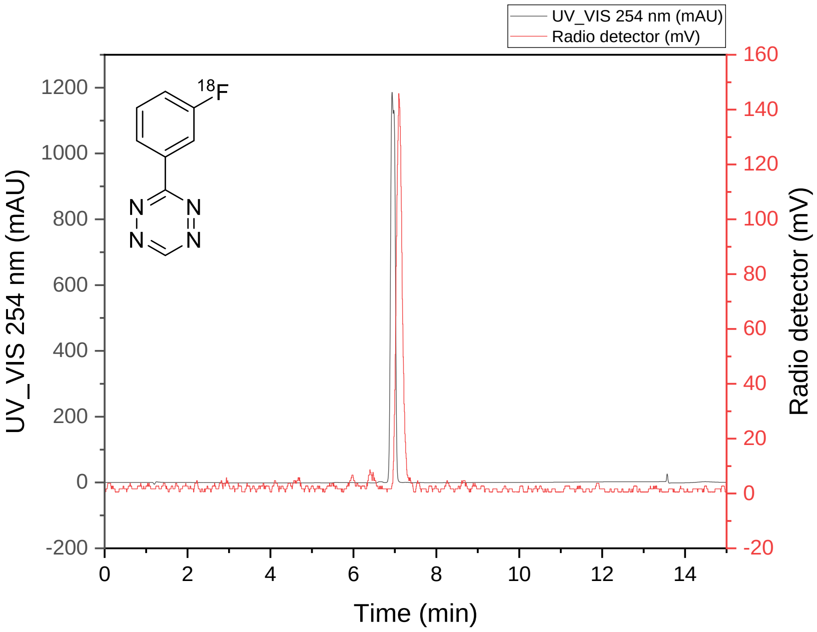

Figure S18. *Analytical-HPLC chromatogram of reference compound* ***1*** *(UV/Vis, 254 nm) and radio-HPLC chromatogram of formulated* ***[^{18}F]1*** *(Rt = 6.87 minutes).*

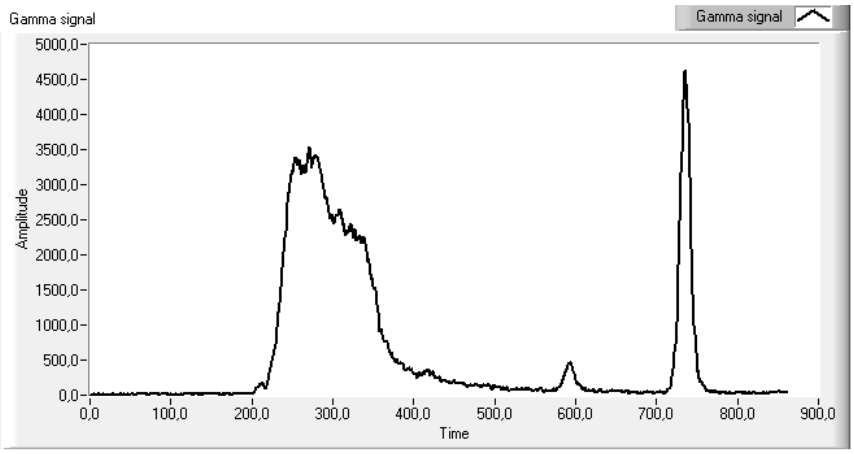

Figure S19. *Semi-preparative HPLC chromatogram for* ***[^{18}F]2*** *(R_t = 720 sec).*

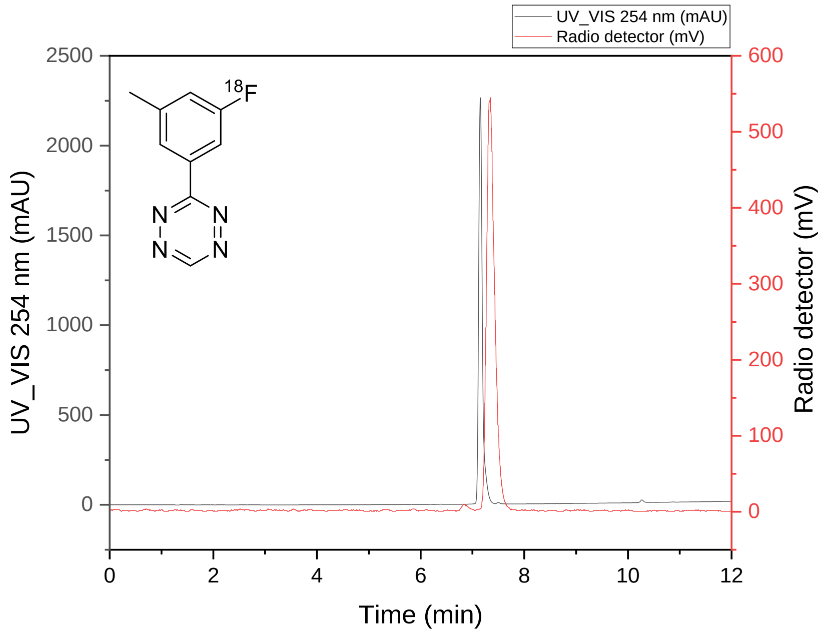

Figure S20. *Analytical-HPLC chromatogram of reference compound* ***2*** *(UV/Vis, 254 nm) and radio-HPLC chromatogram of formulated* ***[^{18}F]2*** *(Rt = 7.35 minutes).*

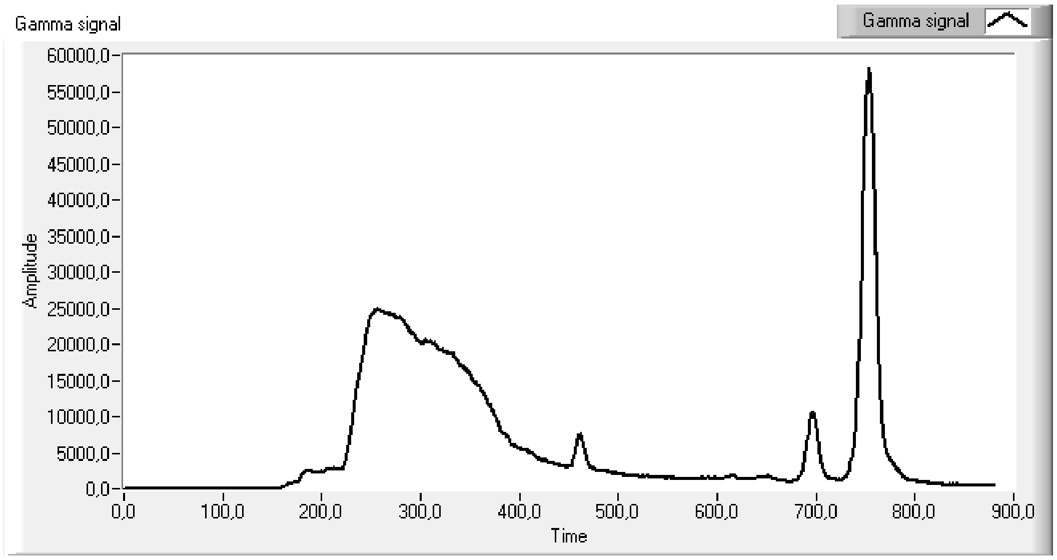

Figure S21. *Semi-preparative HPLC chromatogram for* ***[^{18}F]3*** *(R_t = 750 sec).*

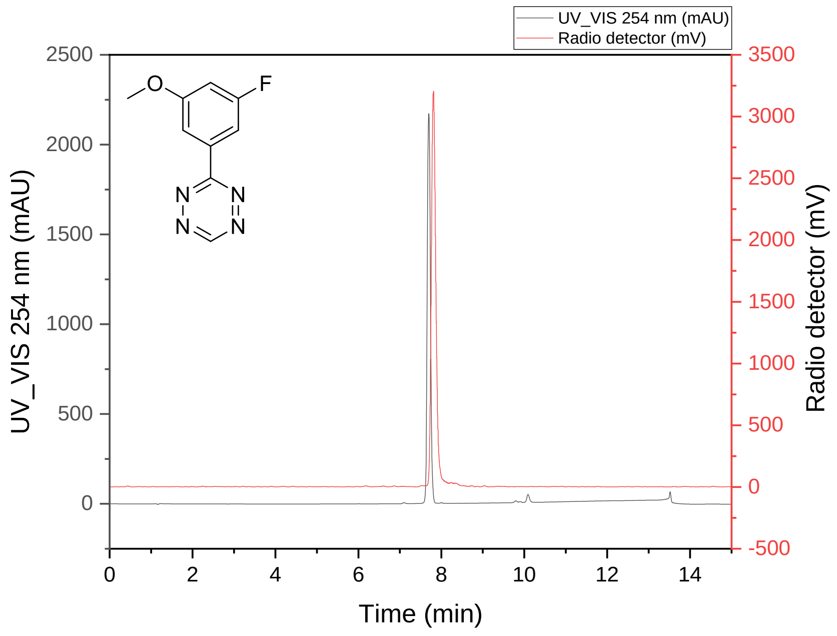

Figure S22. *Analytical-HPLC chromatogram of reference compound **3** (UV/Vis, 254 nm) and radio-HPLC chromatogram of formulated **[^{18}F]3** (Rt = 7.67 minutes).*

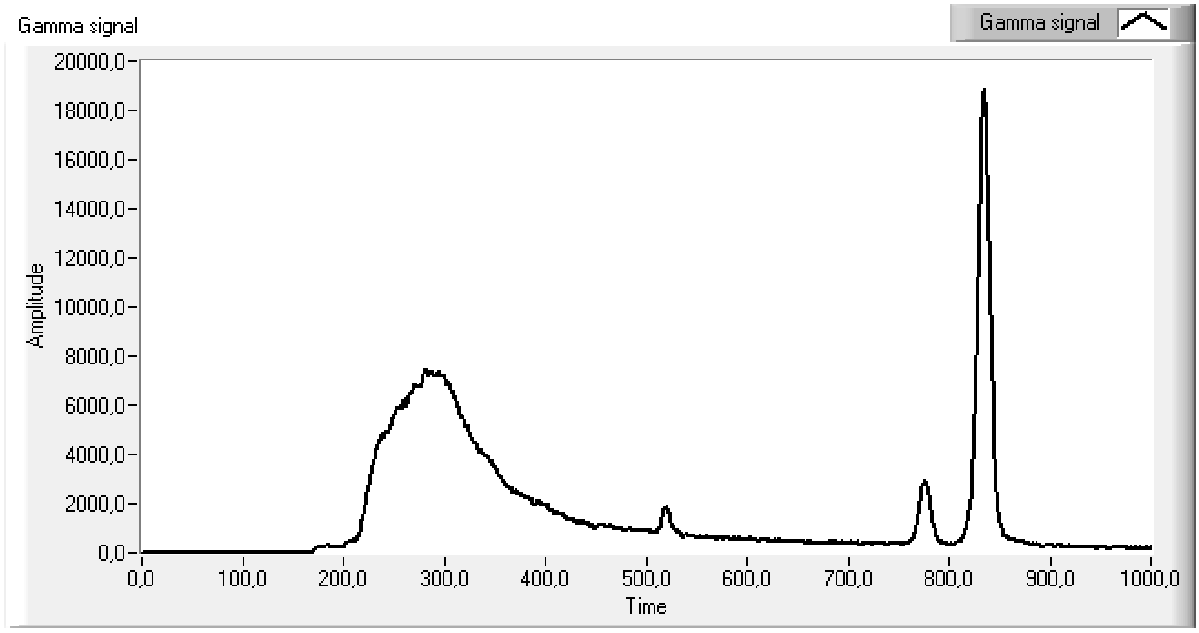

Figure S23. *Semi-preparative HPLC chromatogram for **[^{18}F]4** (R_t = 820 sec).*

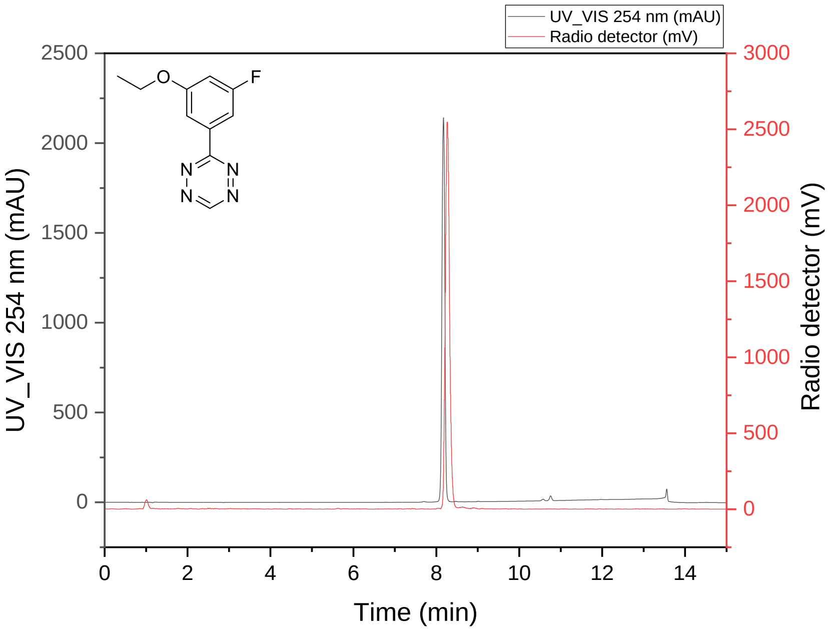

Figure S24. *Analytical-HPLC chromatogram of reference compound* ***4*** *(UV/Vis, 254 nm) and radio-HPLC chromatogram of formulated* ***[¹⁸F]4*** *(Rt = 8.25 minutes).*

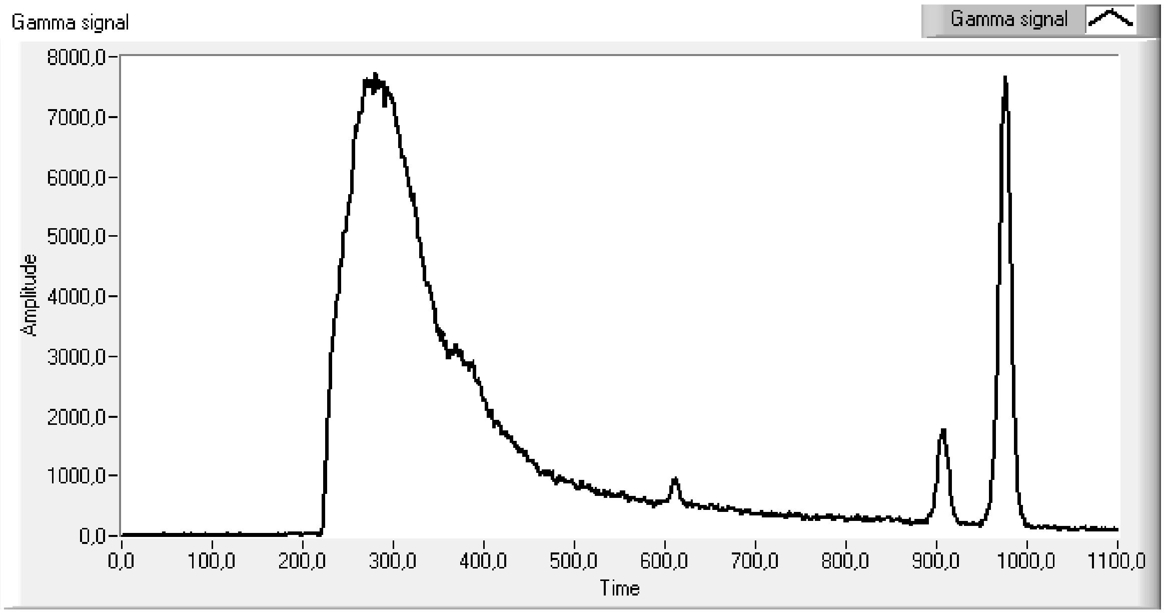

Figure S25. *Semi-preparative HPLC chromatogram for* ***[^{18}F]5*** *(R_t = 990 sec).*

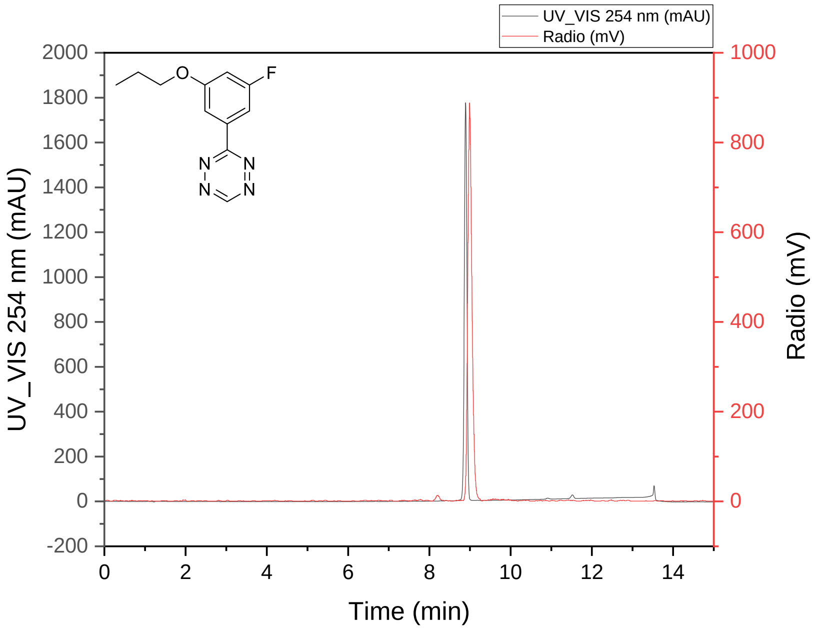

Figure S26. *Analytical-HPLC chromatogram of reference compound **5** (UV/Vis, 254 nm) and radio-HPLC chromatogram of formulated **[^{18}F]5** (Rt = 9.00 minutes).*

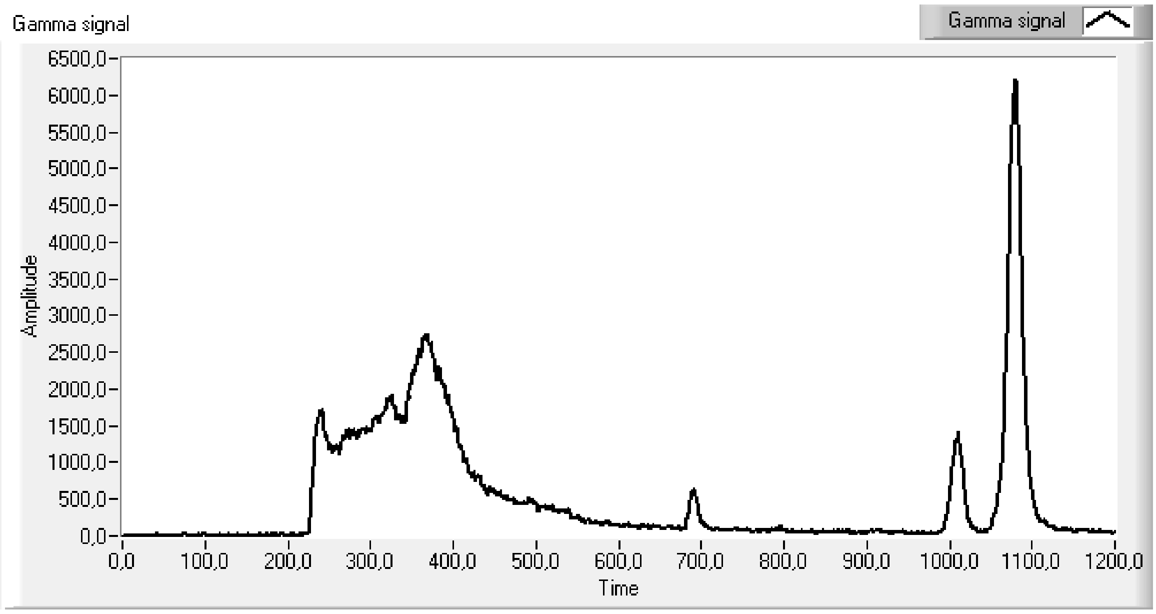

Figure S27. *Semi-preparative HPLC chromatogram for **[^{18}F]6** (R_t = 1070 sec).*

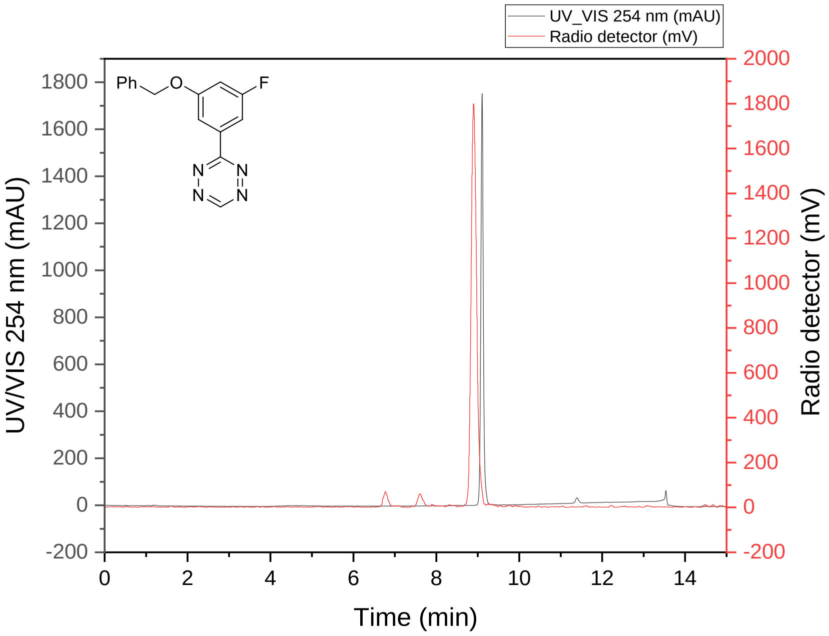

Figure S28. *Analytical-HPLC chromatogram of reference compound **6** (UV/Vis, 254 nm) and radio-HPLC chromatogram of formulated **[¹⁸F]6** (Rt = 9.10 minutes).*

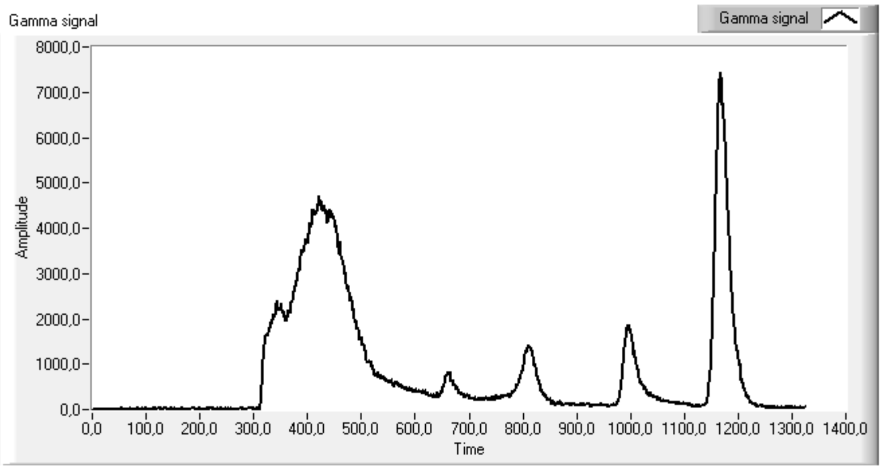

Figure S29. *Semi-preparative HPLC chromatogram for **[^{18}F]7** (R_t = 1150 sec).*

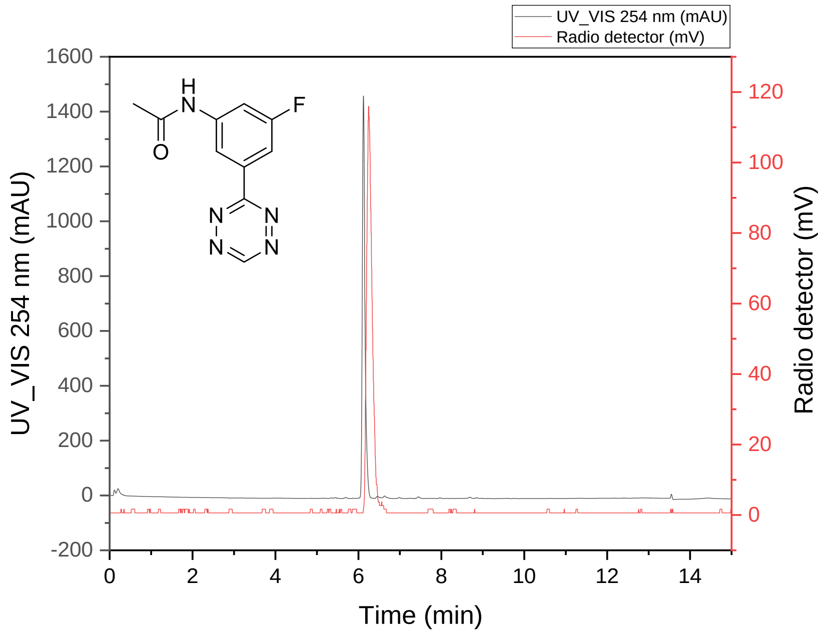

Figure S30. *Analytical-HPLC chromatogram of reference compound **7** (UV/Vis, 254 nm) and radio-HPLC chromatogram of formulated **[18F]7** (Rt = 6.23 minutes).*

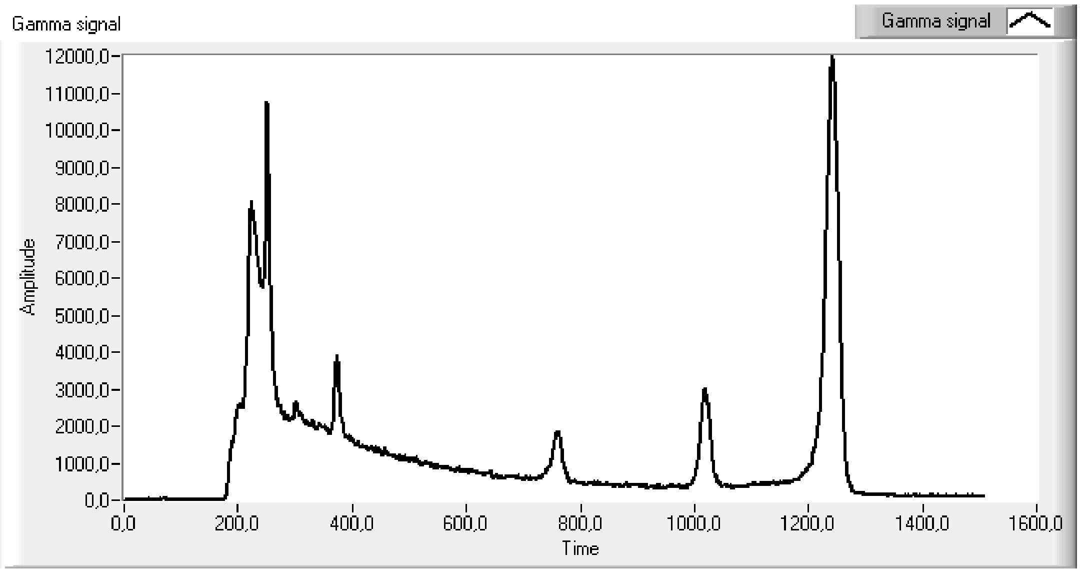

Figure S31. *Semi-preparative HPLC chromatogram for **[18F]8** (R_t = 1200 sec).*

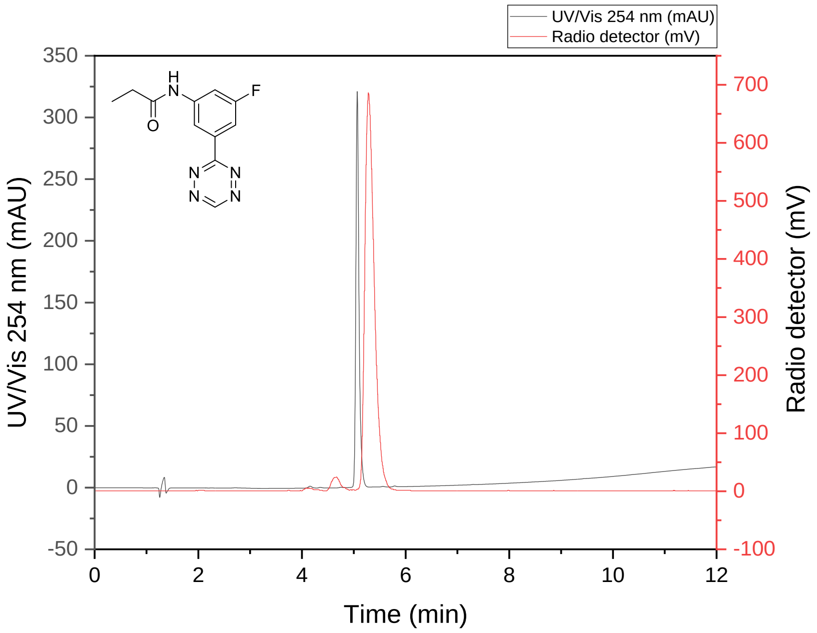

Figure S32. *Analytical-HPLC chromatogram of reference compound* ***8*** *(UV/Vis, 254 nm) and radio-HPLC chromatogram of formulated* ***[^{18}F]8*** *(Rt = 5.30 minutes).*

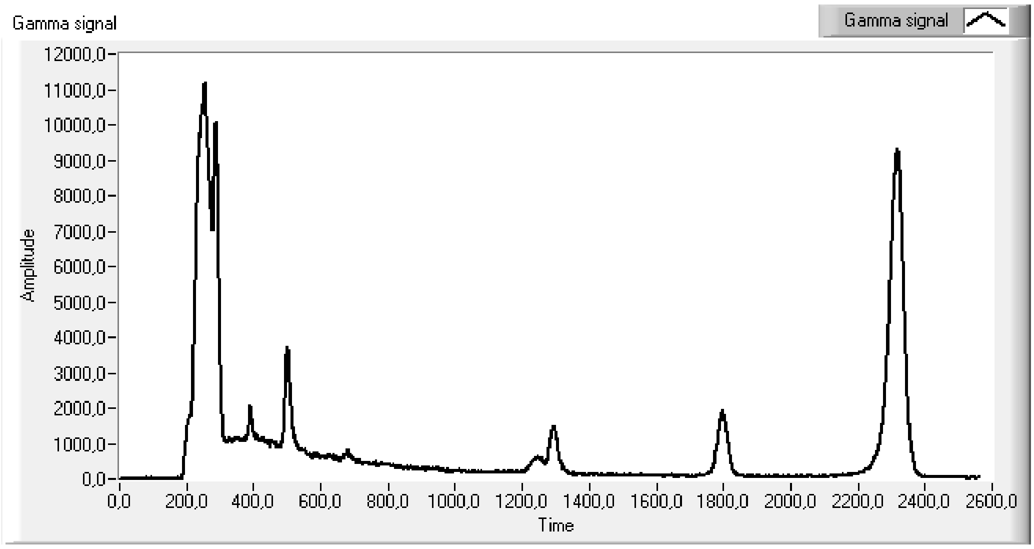

Figure S33. *Semi-preparative HPLC chromatogram for* ***[^{18}F]9*** *(R_t = 2300 sec).*

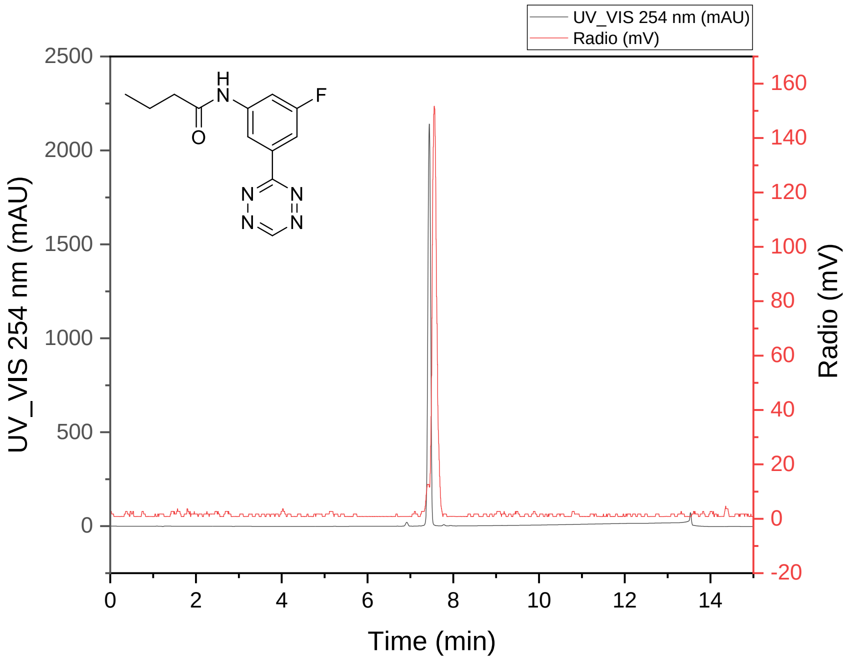

Figure S34. *Analytical-HPLC chromatogram of reference compound* ***9*** *(UV/Vis, 254 nm) and radio-HPLC chromatogram of formulated* ***[18F]9*** *(Rt = 7.55 minutes).*

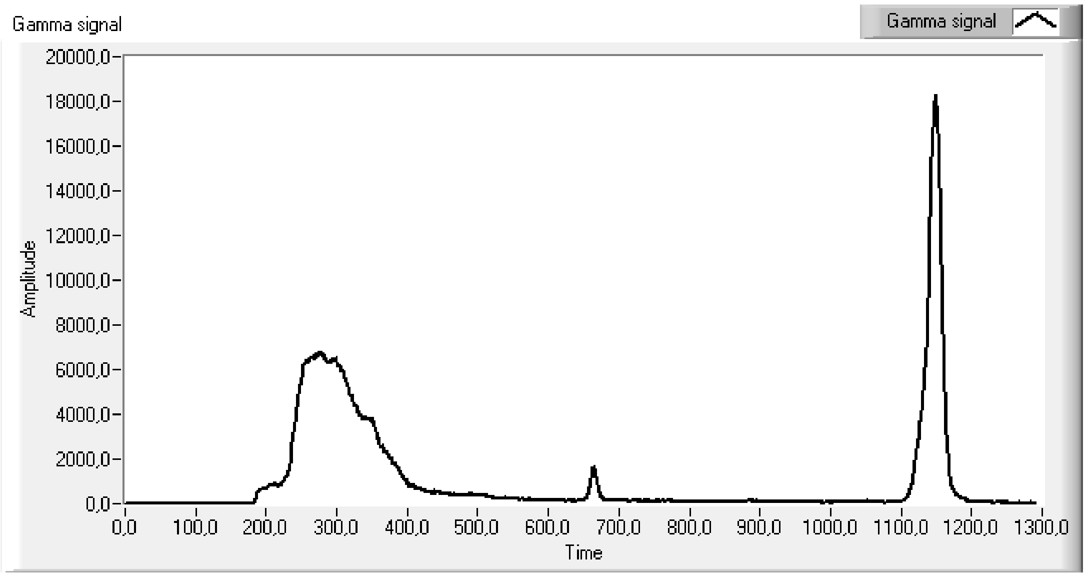

Figure S35. *Semi-preparative HPLC chromatogram for* ***[18F]10*** *(R_t = 1150 sec).*

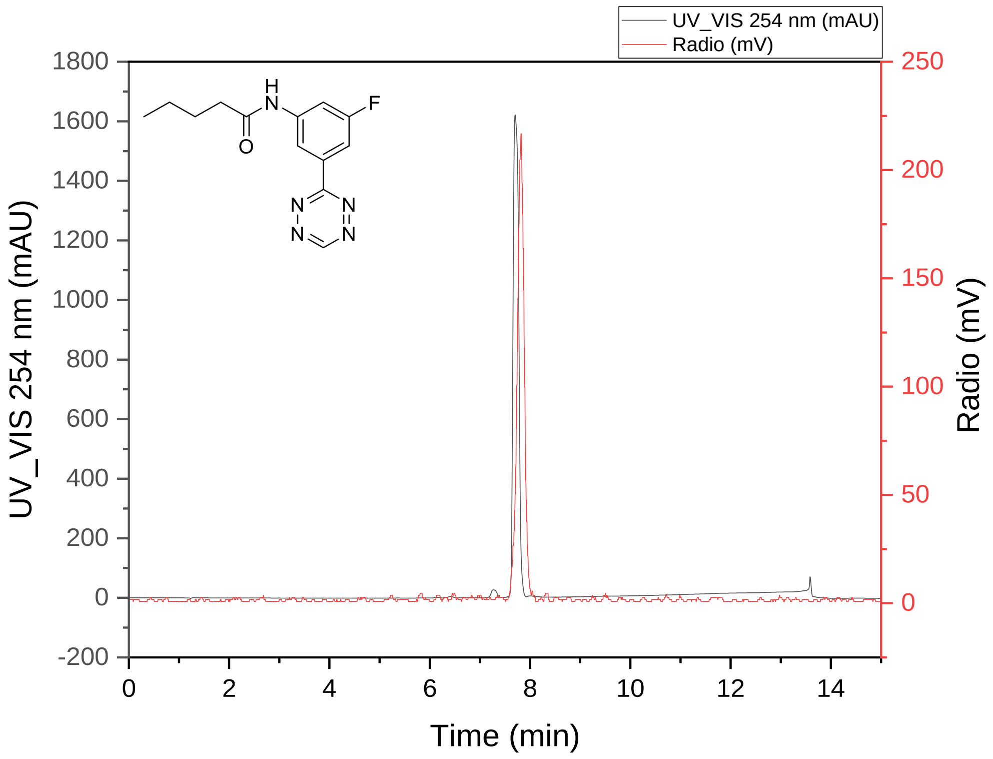

Figure S36. *Analytical-HPLC chromatogram of reference compound* ***10*** *(UV/Vis, 254 nm) and radio-HPLC chromatogram of formulated* ***[18F]10*** *(Rt = 7.82 minutes).*

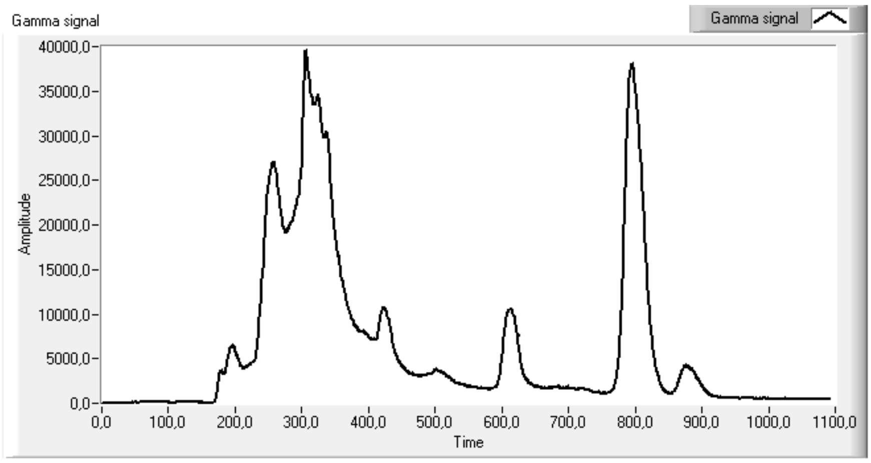

Figure S37. *Semi-preparative HPLC chromatogram for* ***[18F]11*** *(R_t = 790 sec).*

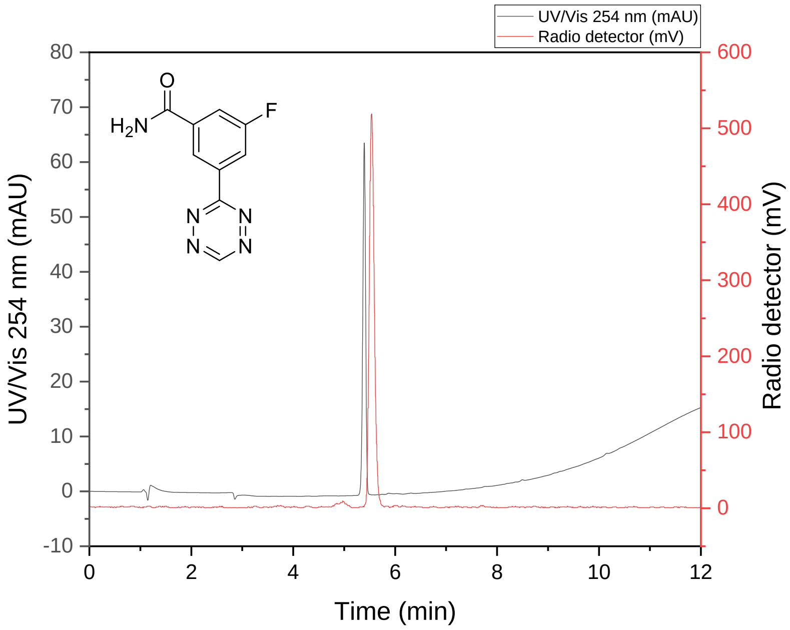

Figure S38. *Analytical-HPLC chromatogram of reference compound **11** (UV/Vis, 254 nm) and radio-HPLC chromatogram of formulated **[^{18}F]11** (Rt = 5.51 minutes).*

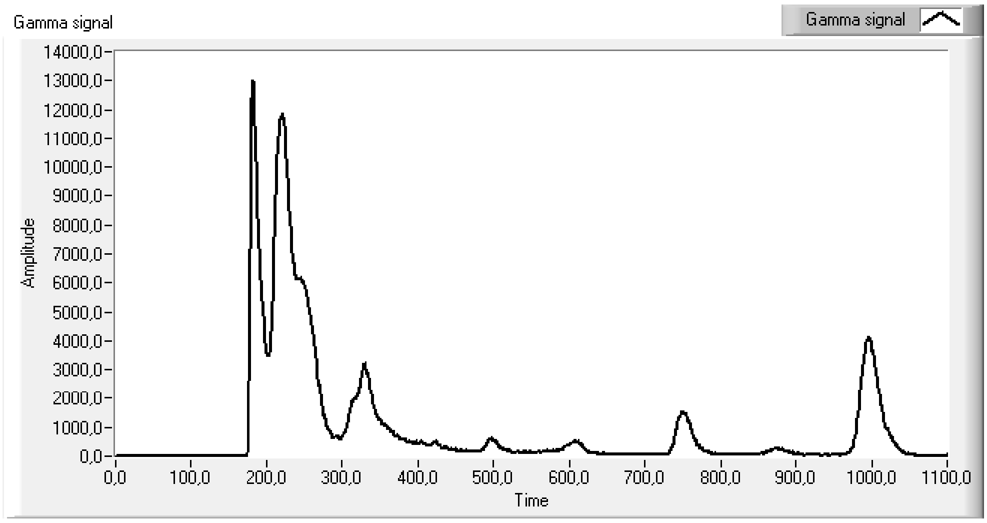

Figure S39. *Semi-preparative HPLC chromatogram for **[^{18}F]12** (R_t = 990 sec).*

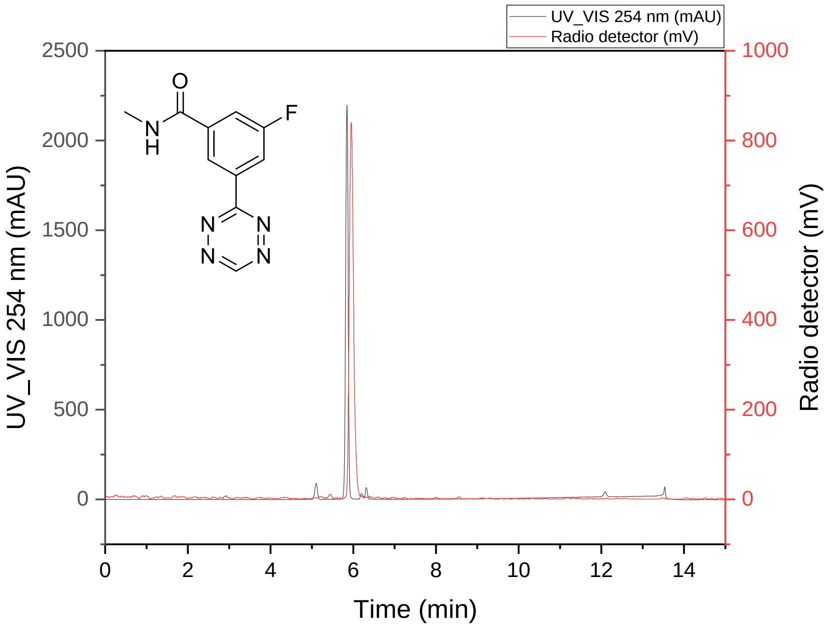

Figure S40. *Analytical-HPLC chromatogram of reference compound* ***12*** *(UV/Vis, 254 nm) and radio-HPLC chromatogram of formulated* ***[18F]12*** *(Rt = 5.93 minutes).*

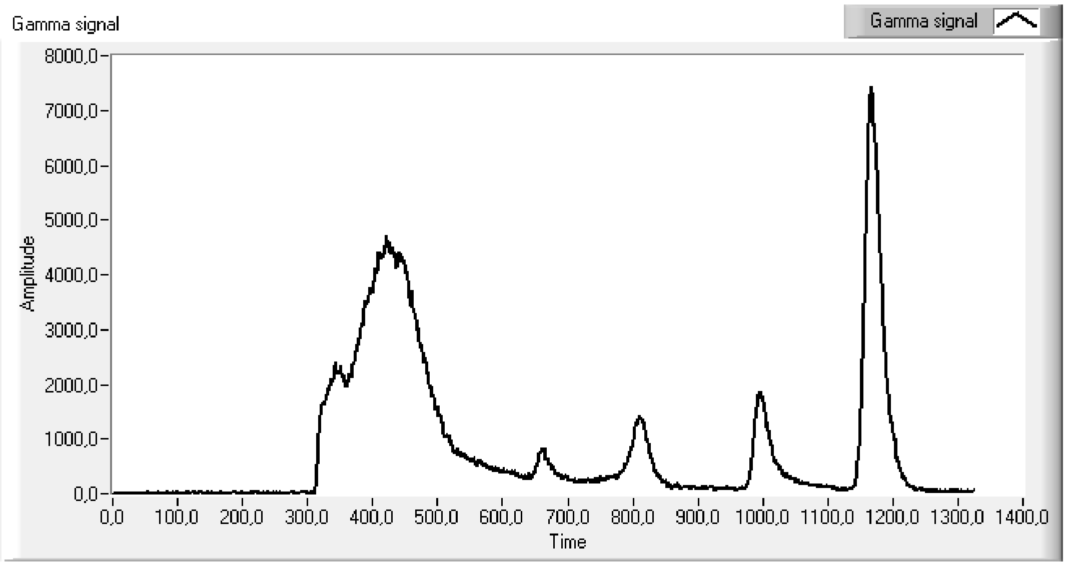

Figure S41*. Semi-preparative HPLC chromatogram for* ***[18F]13*** *(R_t = 1150 sec).*

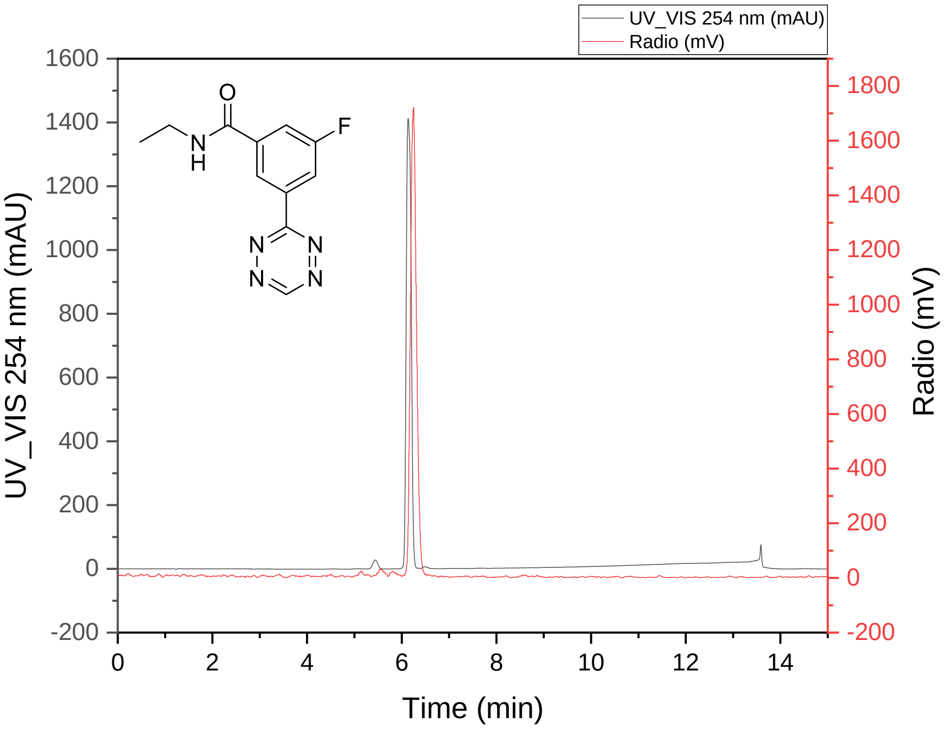

Figure S42. *Analytical-HPLC chromatogram of reference compound **13** (UV/Vis, 254 nm) and radio-HPLC chromatogram of formulated **[^{18}F]13** (Rt = 6.24 minutes).*

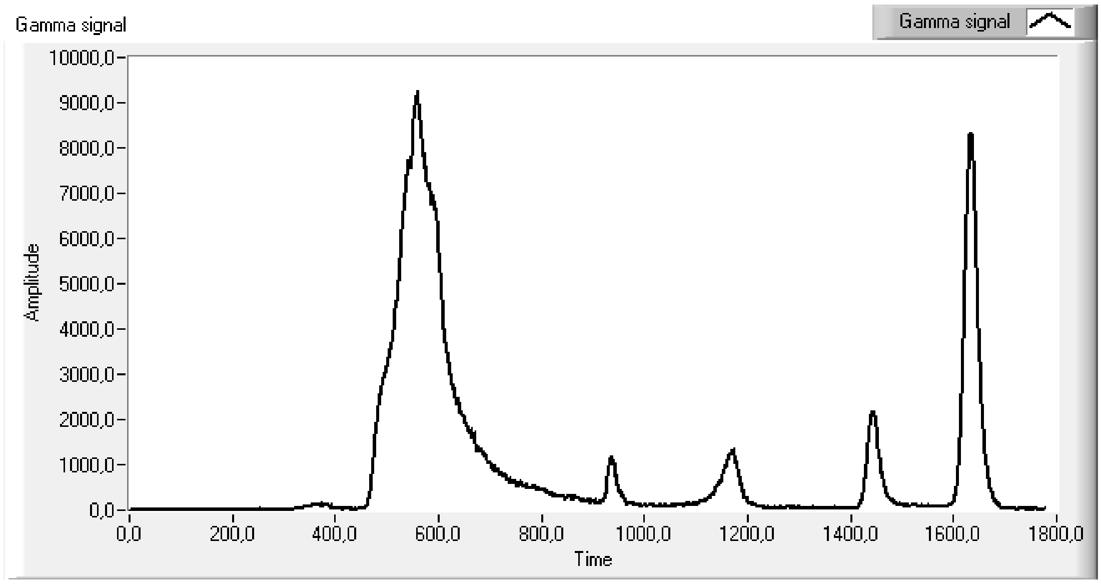

Figure S43. *Semi-preparative HPLC chromatogram for **[^{18}F]14** (R_t = 1600 sec).*

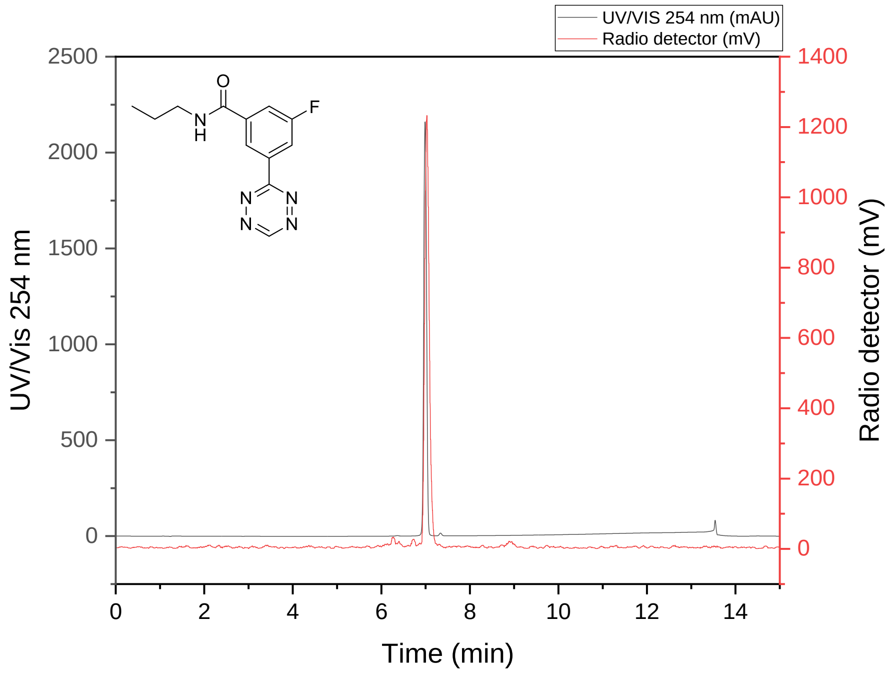

Figure S44. *Analytical-HPLC chromatogram of reference compound* ***14*** *(UV/Vis, 254 nm) and radio-HPLC chromatogram of formulated* ***[18F]14*** *(Rt = 7.02 minutes).*

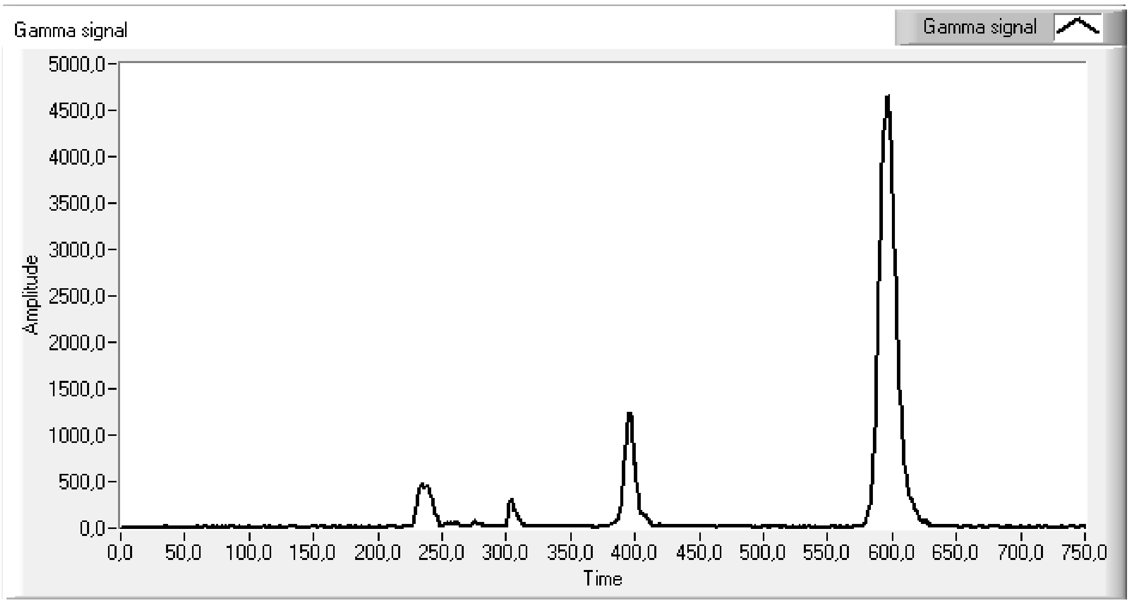

Figure S45. *Semi-preparative HPLC chromatogram for* ***[18F]15*** *(R_t = 590 sec).*

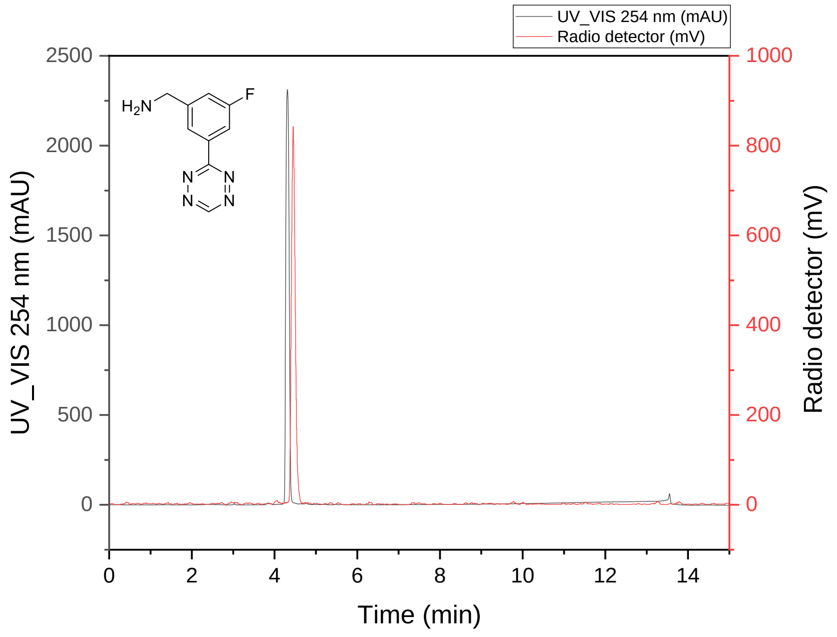

Figure S46. *Analytical-HPLC chromatogram of reference compound **15** (UV/Vis, 254 nm) and radio-HPLC chromatogram of formulated **[^{18}F]15** (Rt = 4.44 minutes).*

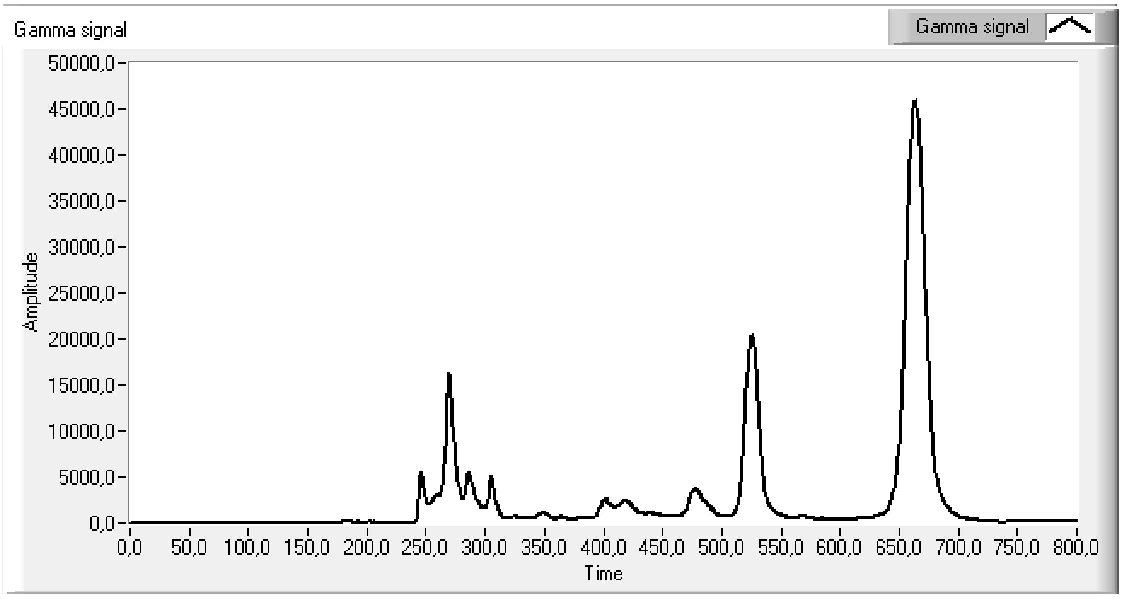

Figure S47. *Semi-preparative HPLC chromatogram for **[^{18}F]16** (R_t = 650 sec).*

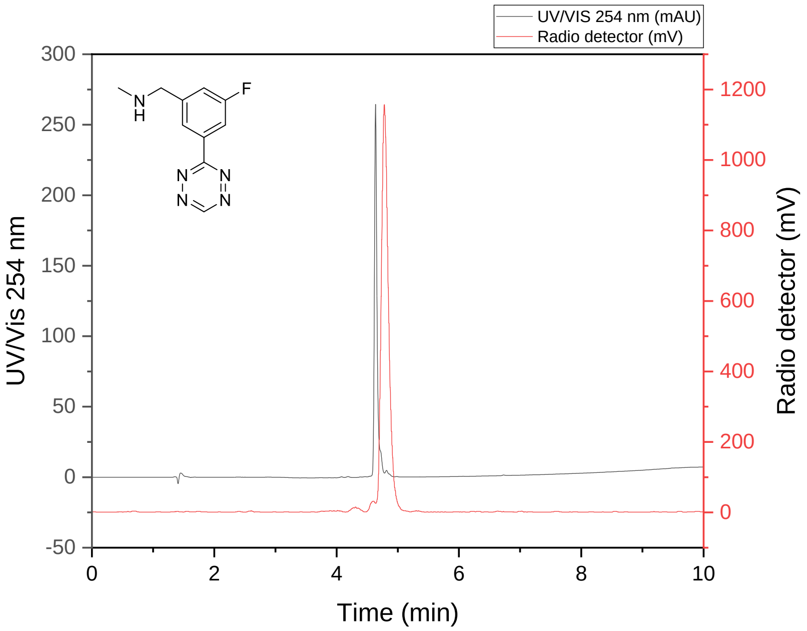

Figure S48. *Analytical-HPLC chromatogram of reference compound* ***16*** *(UV/Vis, 254 nm) and radio-HPLC chromatogram of formulated* ***[^{18}F]16*** *(Rt = 4.78 minutes).*

HPLC elution method: 0-1 min – 5% B, 1-8 min - gradient from 5% B to 75% B, 8-9 min – 75% B, 9-9.5 min - back to 5% B, 9.5-10 min – 5% B; flow rate 1.5 mL/min.

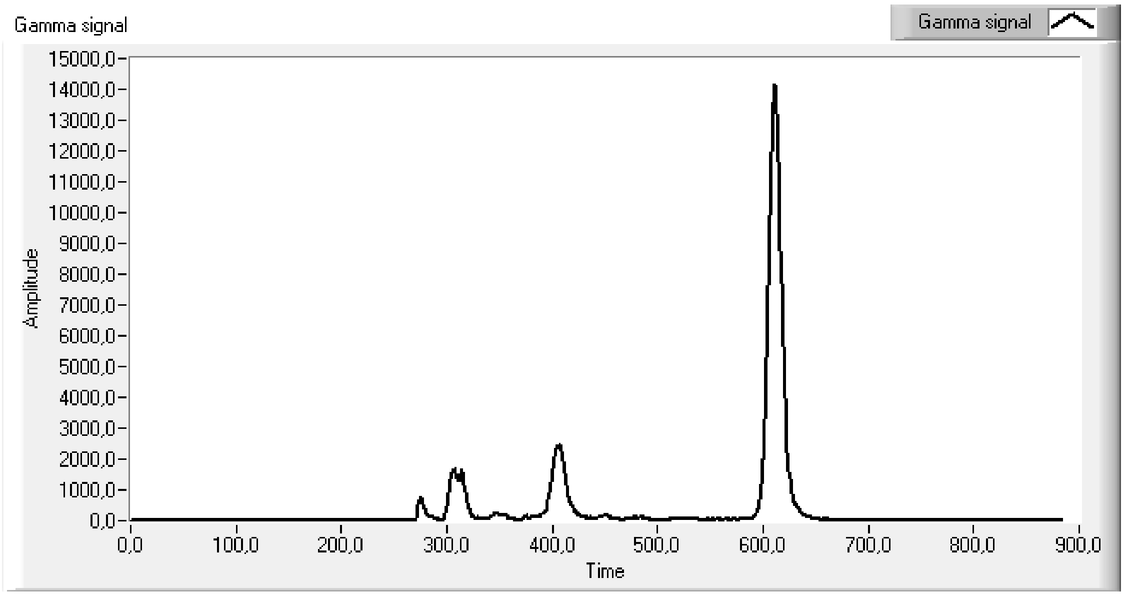

Figure S49. *Semi-preparative HPLC chromatogram for* ***[^{18}F]17*** *(R_t = 600 sec).*

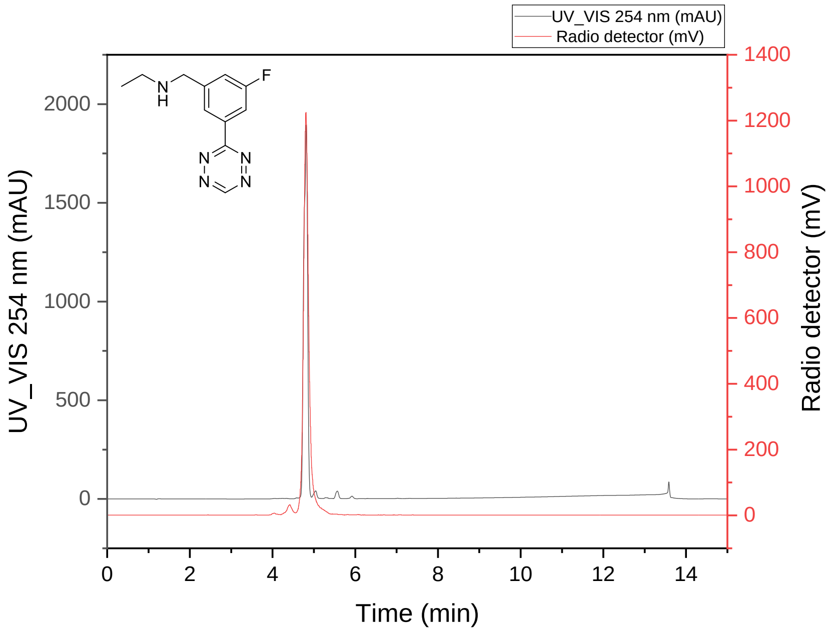

Figure S50. *Analytical-HPLC chromatogram of reference compound* ***17*** *(UV/Vis, 254 nm) and radio-HPLC chromatogram of formulated* ***[18F]17*** *(Rt = 4.81 minutes).*

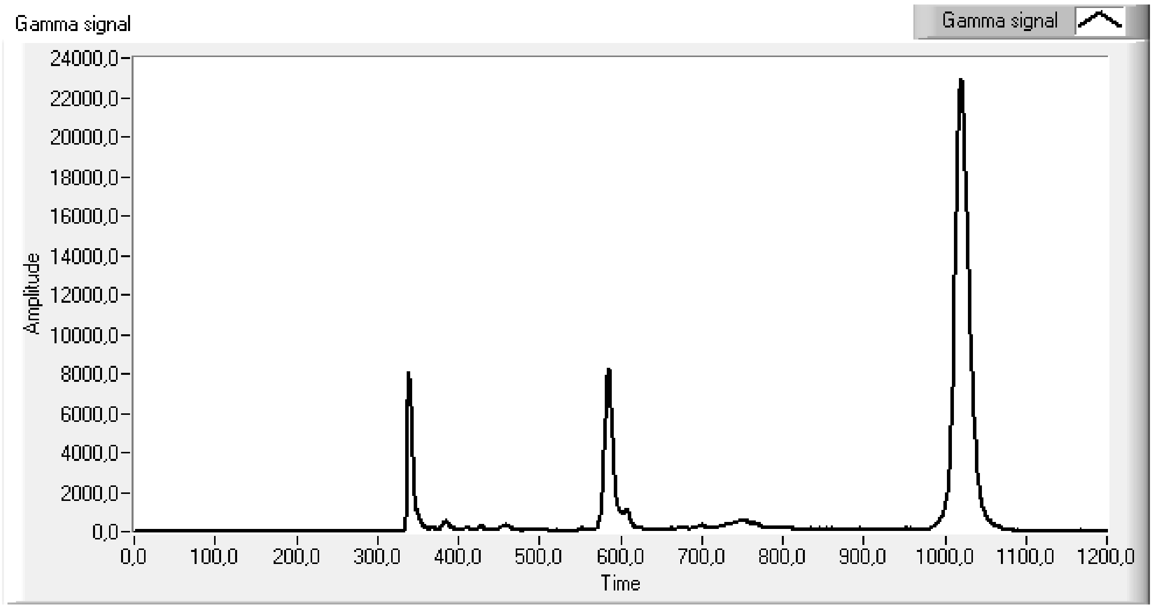

Figure S51. *Semi-preparative HPLC chromatogram for* ***[18F]18*** *(R_t = 1000 sec).*

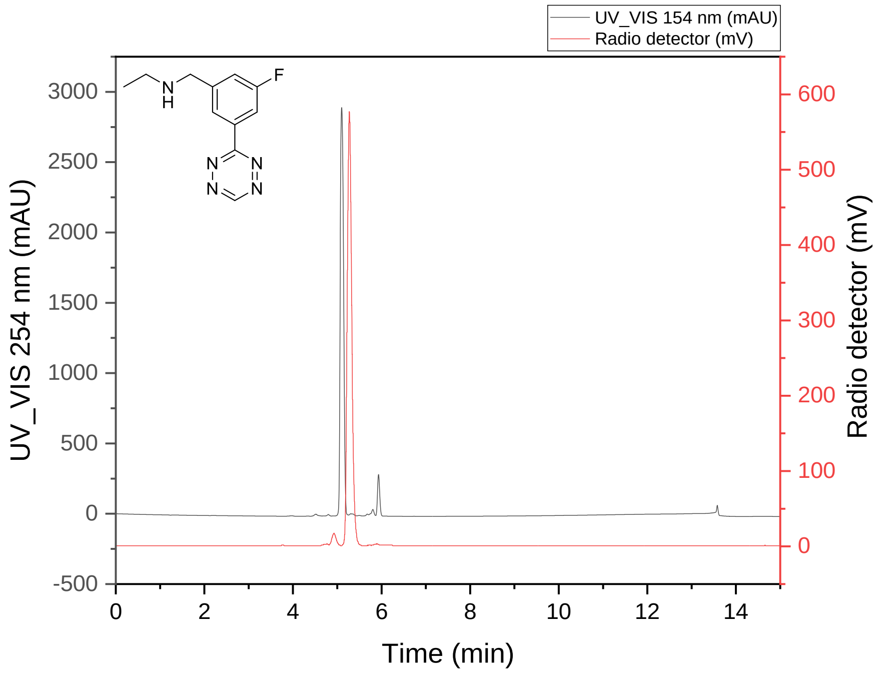

Figure S52. *Analytical-HPLC chromatogram of reference compound* ***18*** *(UV/Vis, 254 nm) and radio-HPLC chromatogram of formulated* ***[^{18}F]18*** *(Rt = 5.26 minutes).*

Section S13. ^{1}H, ^{13}C and ^{19}F NMR spectra

3-Ethoxy-5-fluorobenzonitrile

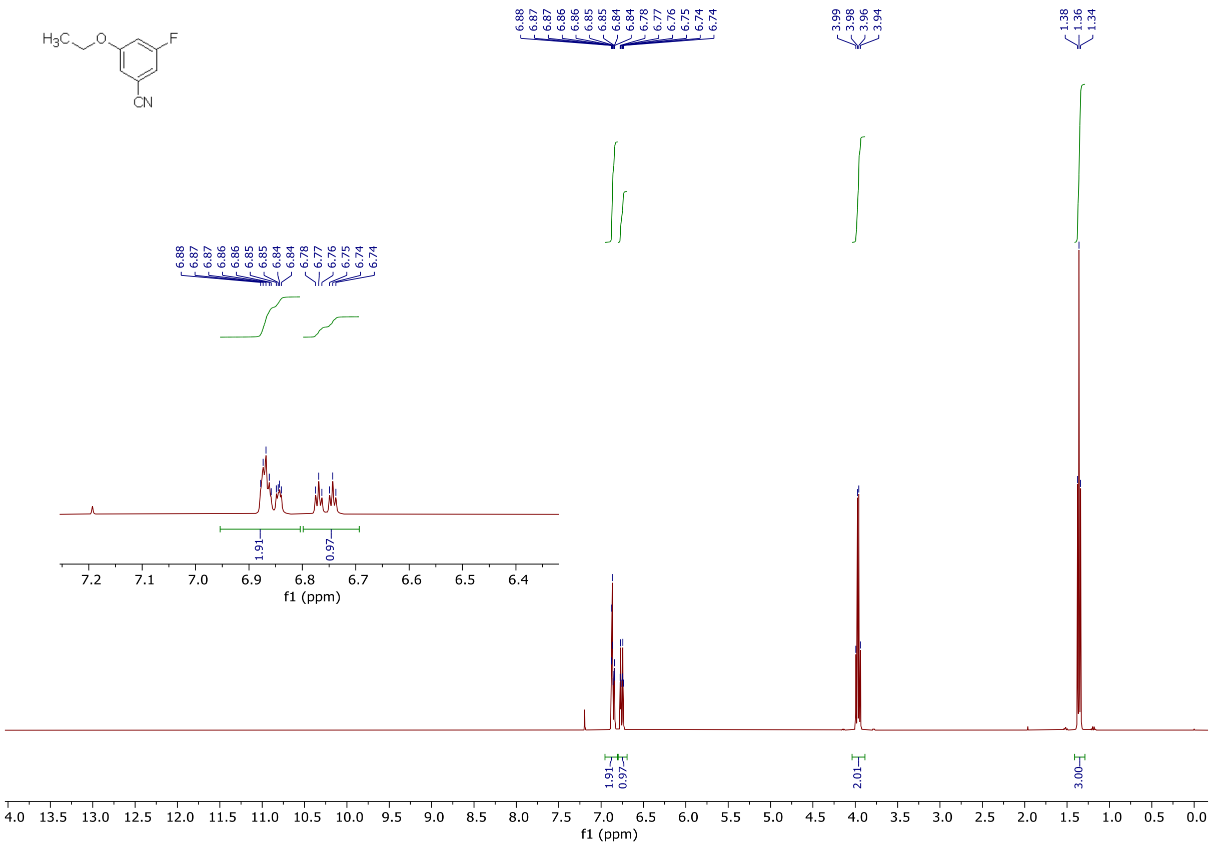

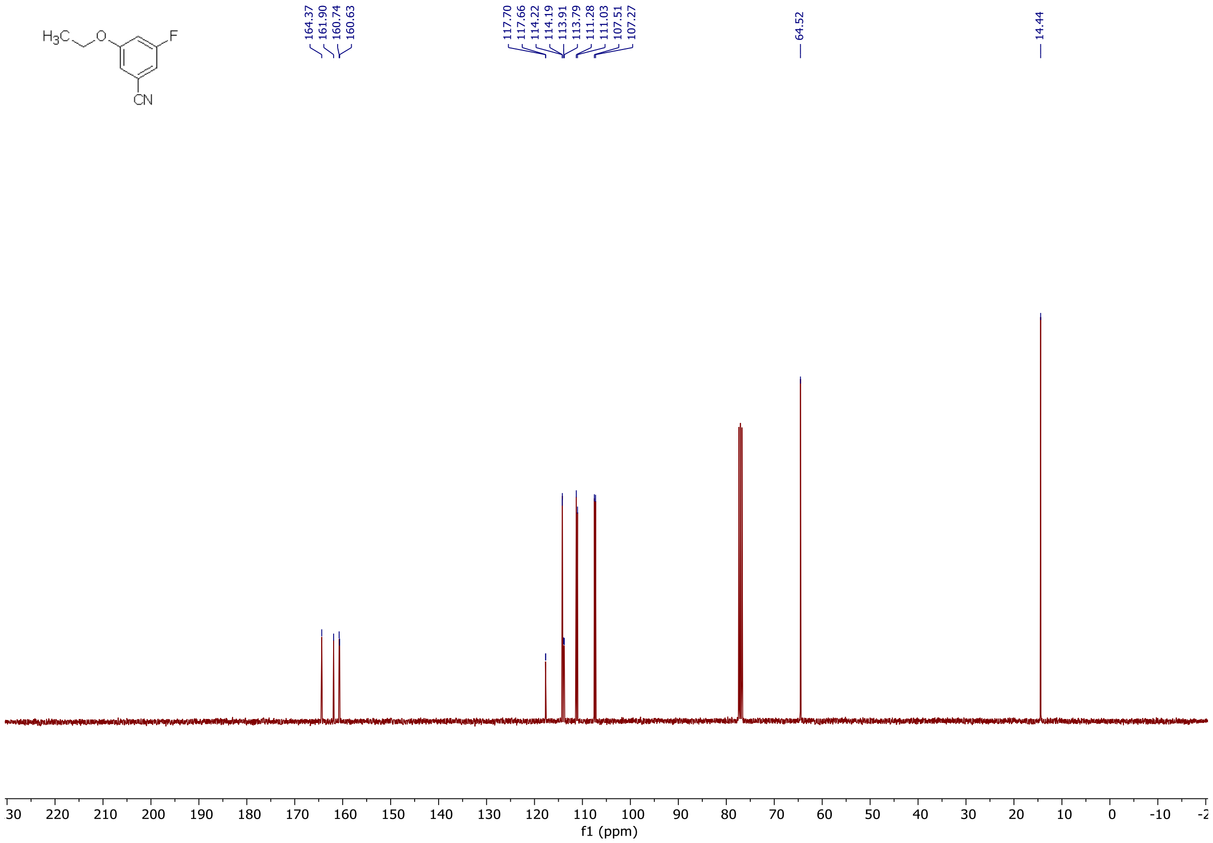

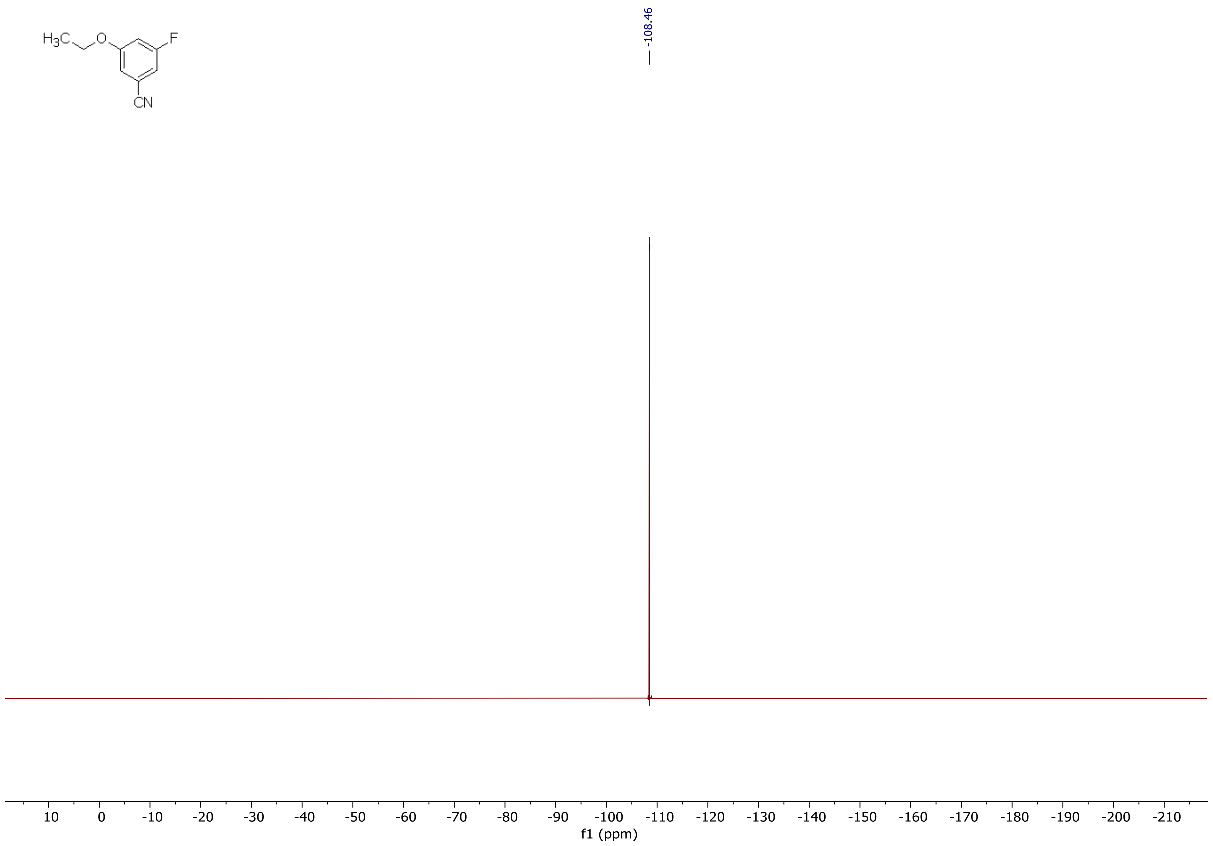

3-(3-Ethoxy-5-fluorophenyl)-1,2,4,5-tetrazine

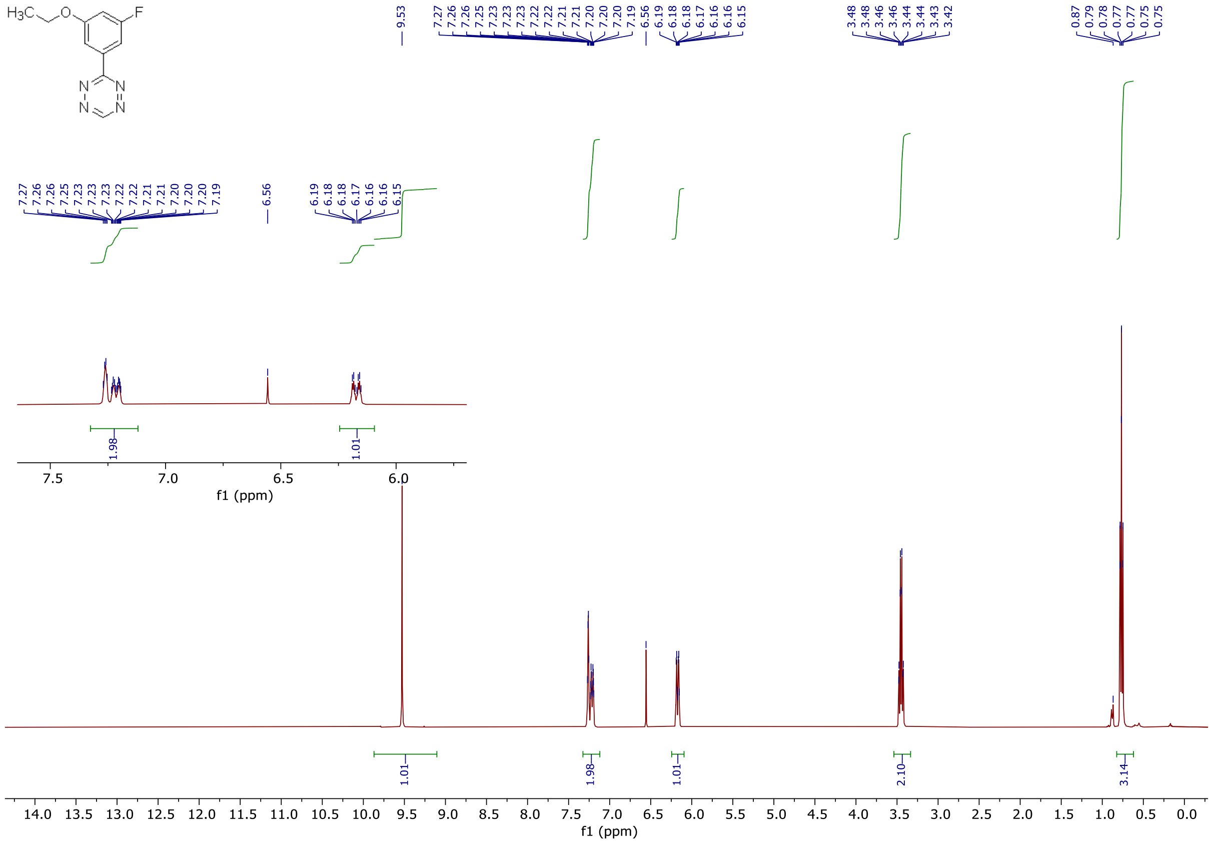

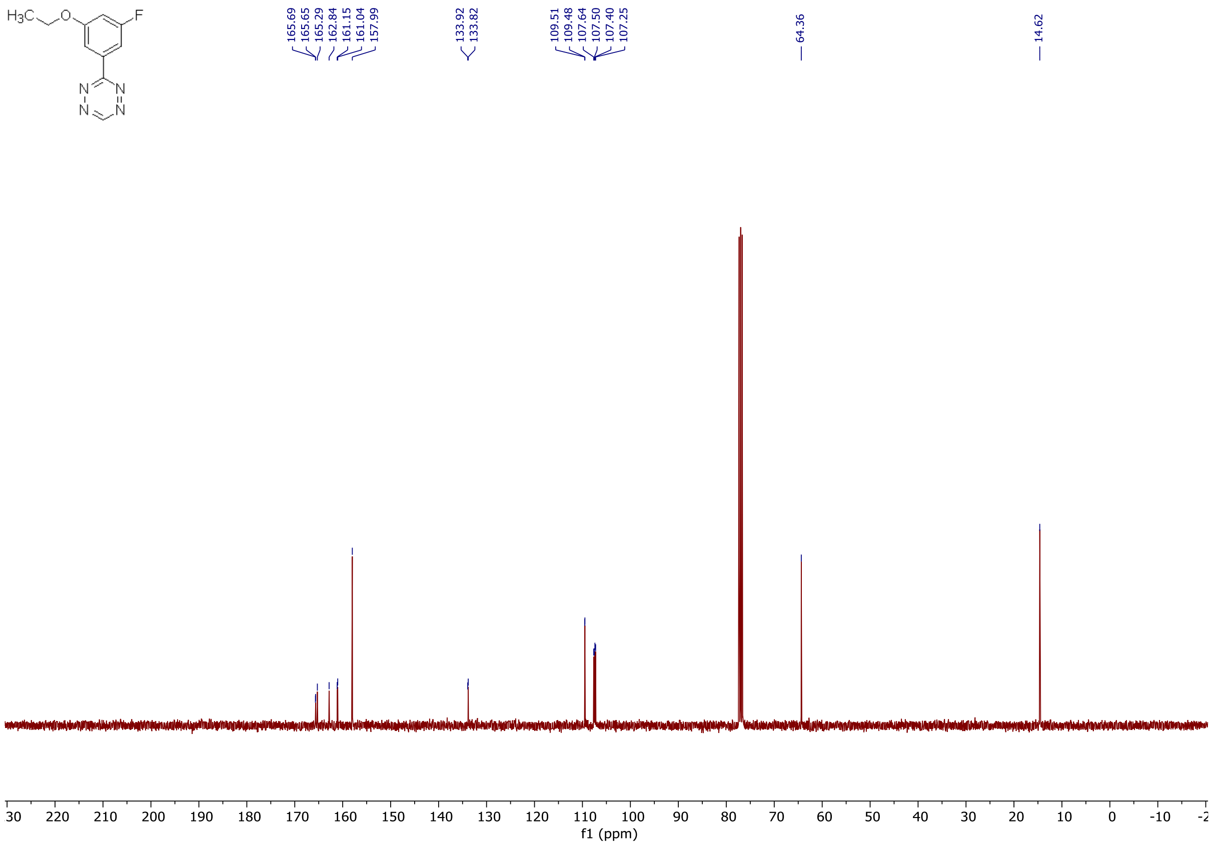
H3C O F
N N
N N
165.69
165.65
165.29
162.84
161.15
161.04
157.99
133.92
133.82
109.51
109.48
107.64
107.50
107.40
107.25
64.36
14.62
f1 (ppm)

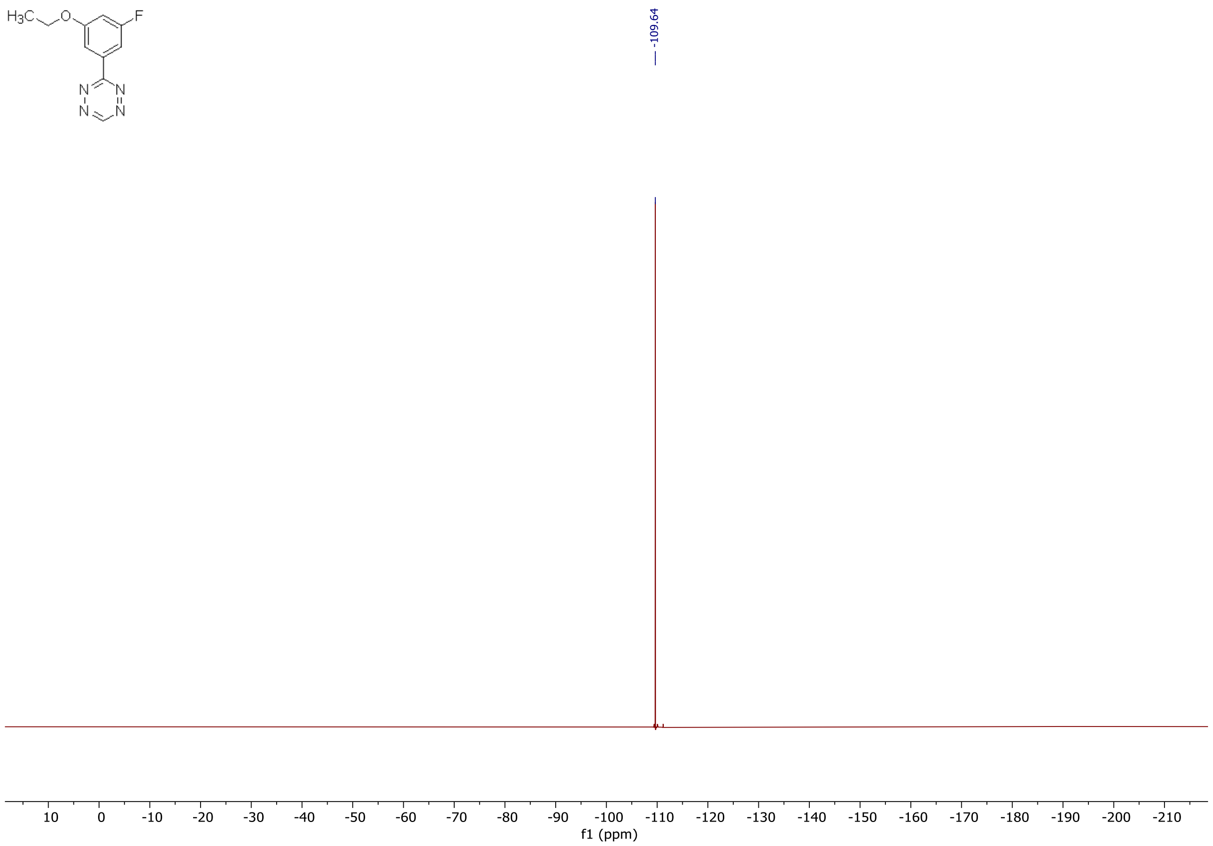
H3C O F
N N
N N
-109.64
f1 (ppm)

3-Fluoro-5-propoxybenzonitrile

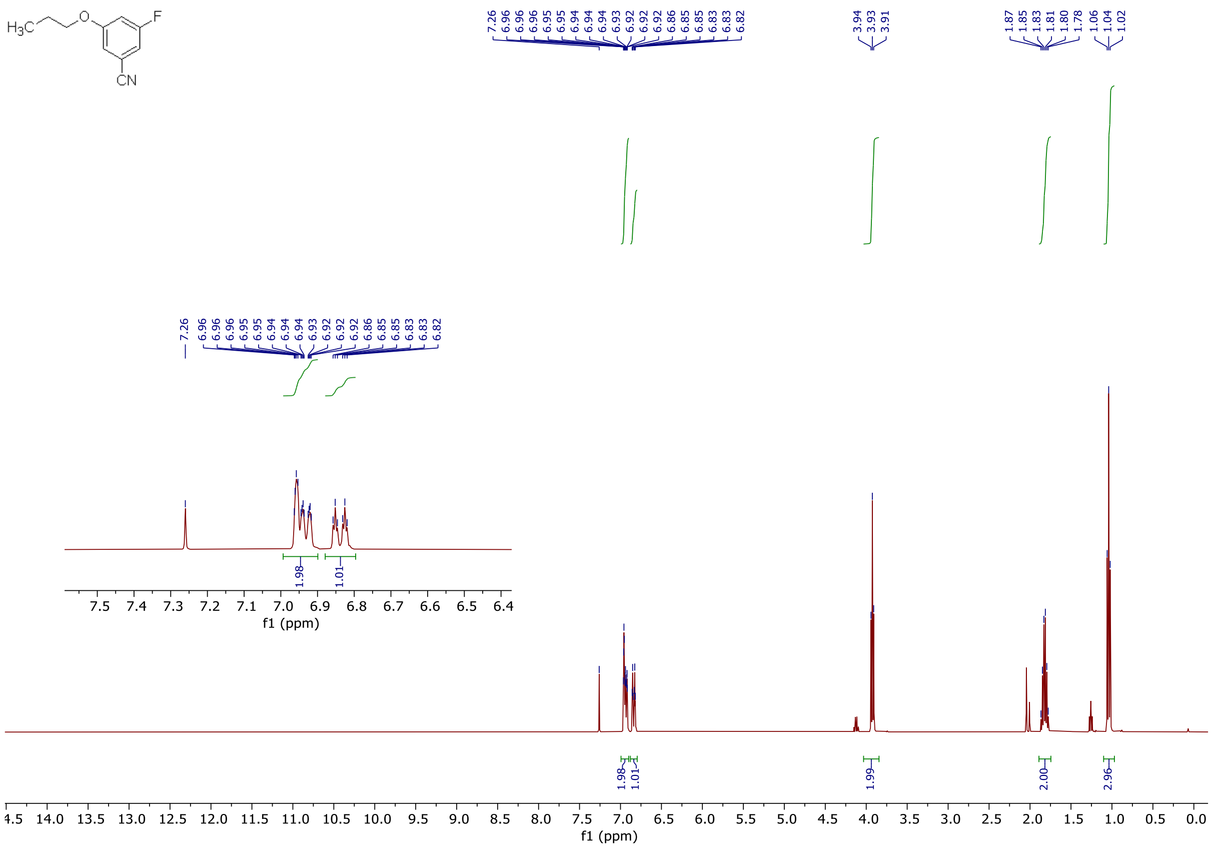

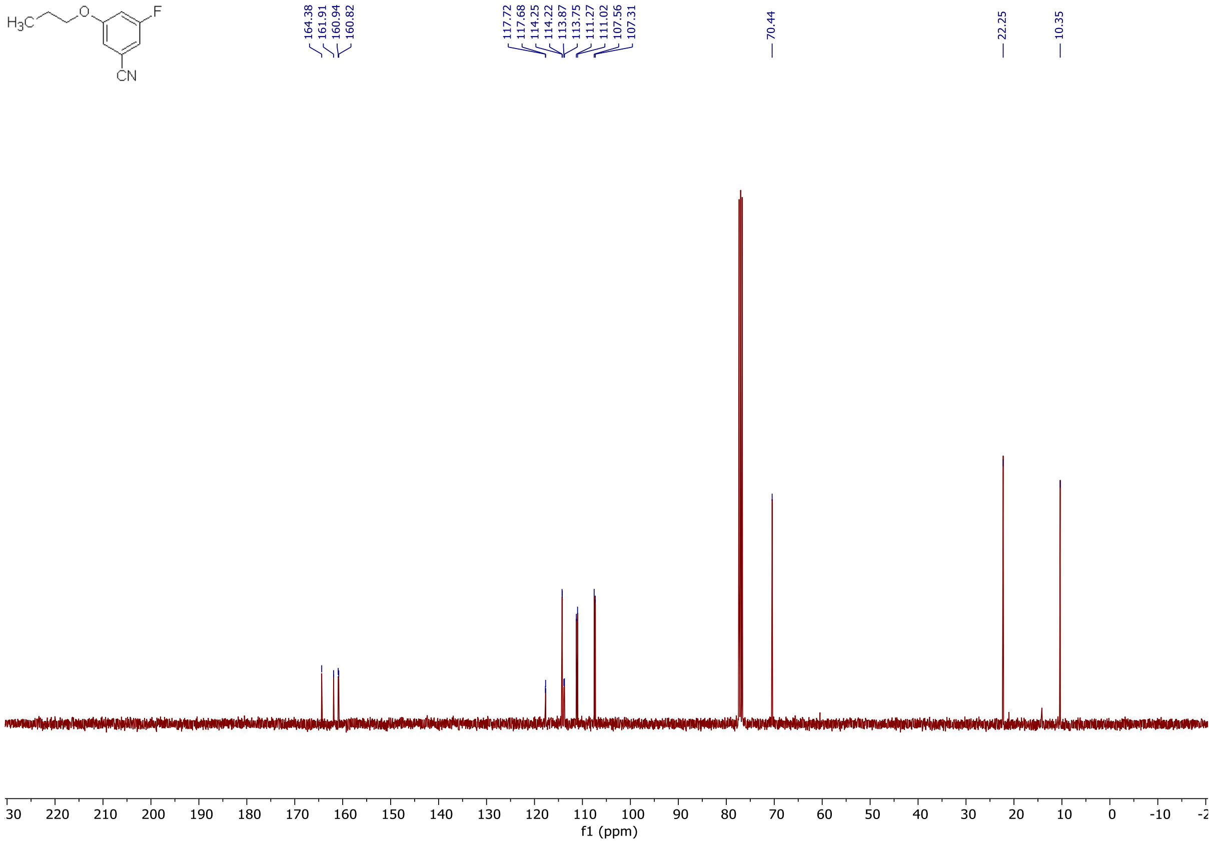

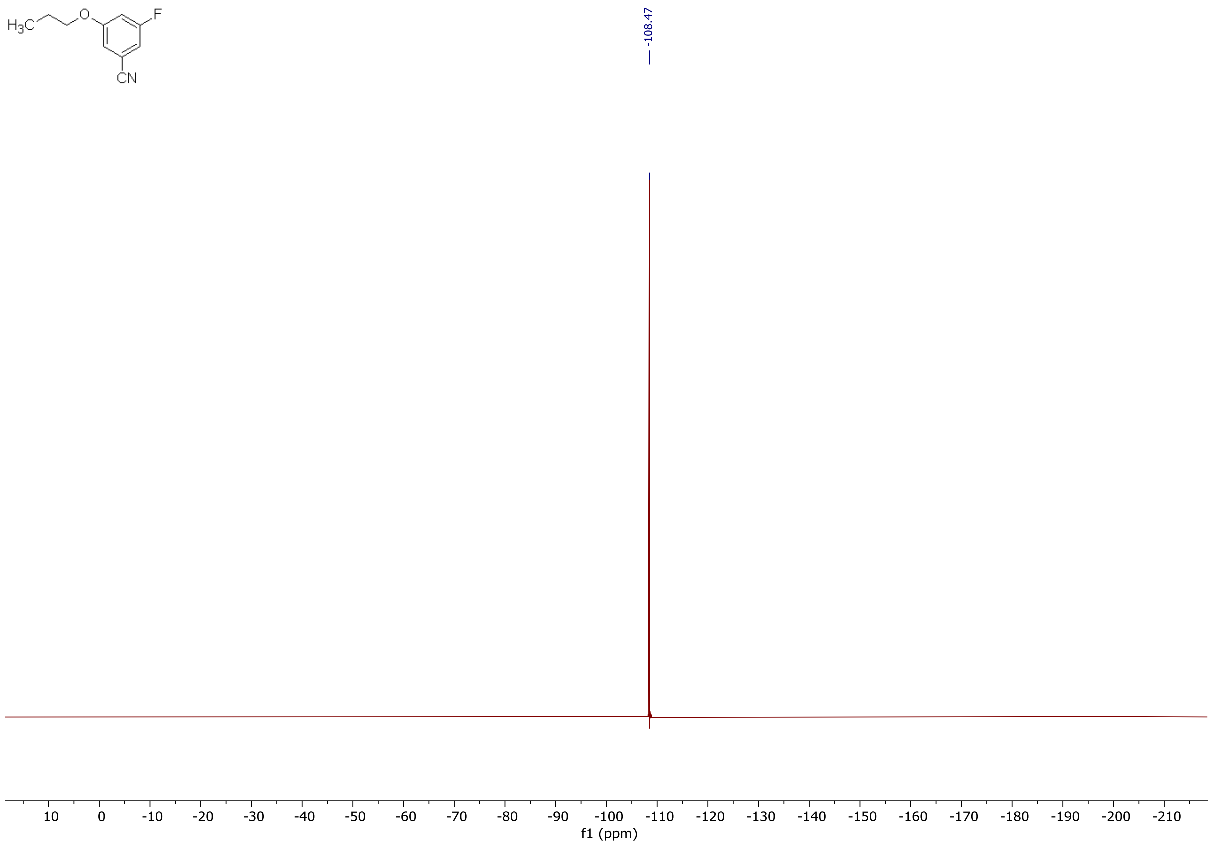

3-(3-Fluoro-5-propoxyphenyl)-1,2,4,5-tetrazine

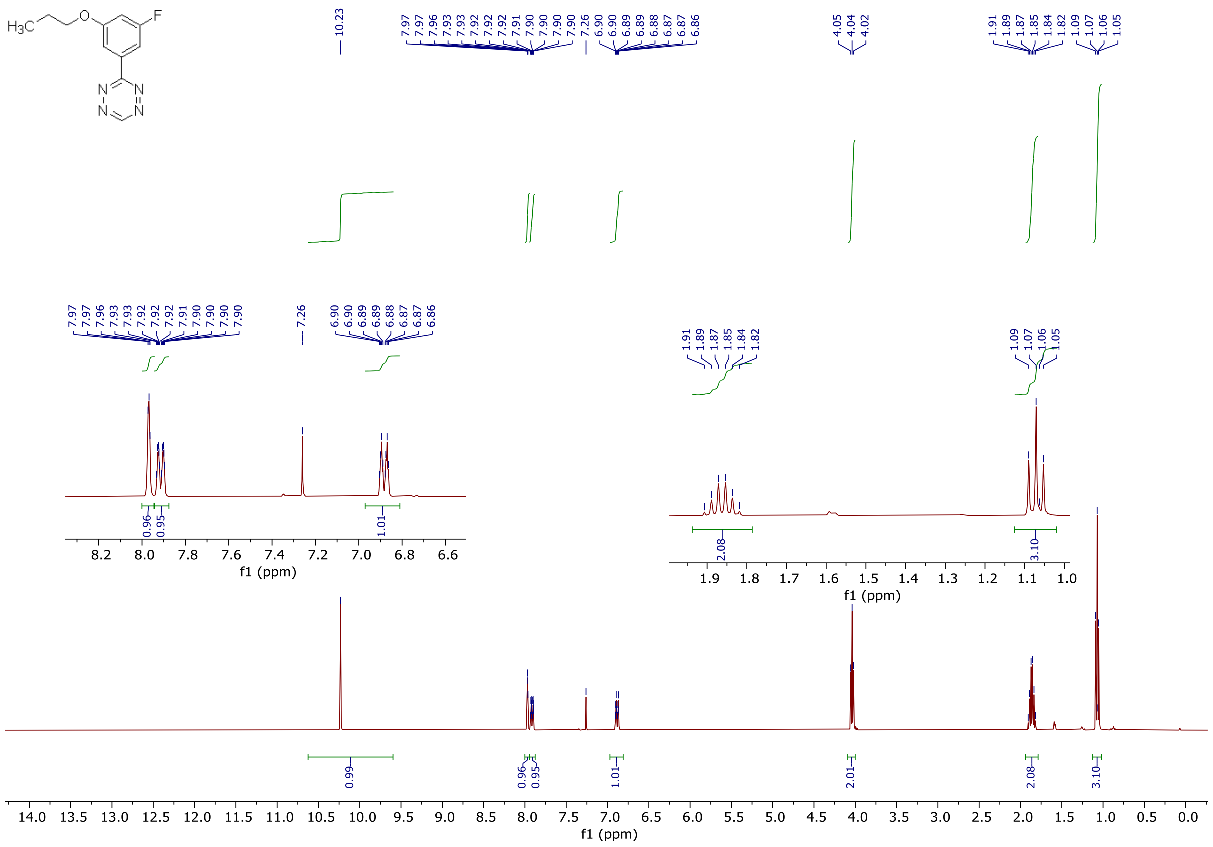

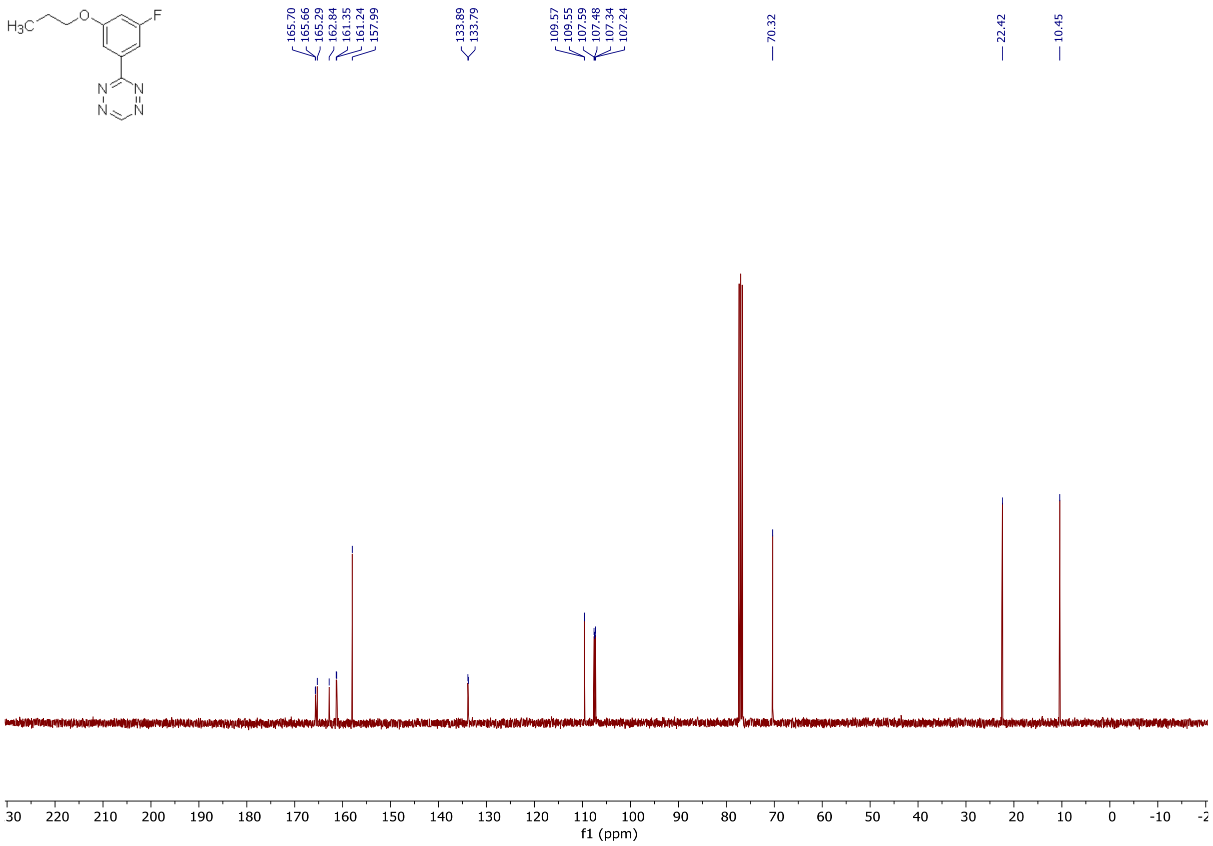
H3C
O
F
N
N
N
N
165.70
165.66
165.29
162.84
161.35
161.24
157.99
133.89
133.79
109.57
109.55
107.59
107.48
107.34
107.24
70.32
22.42
10.45
f1 (ppm)

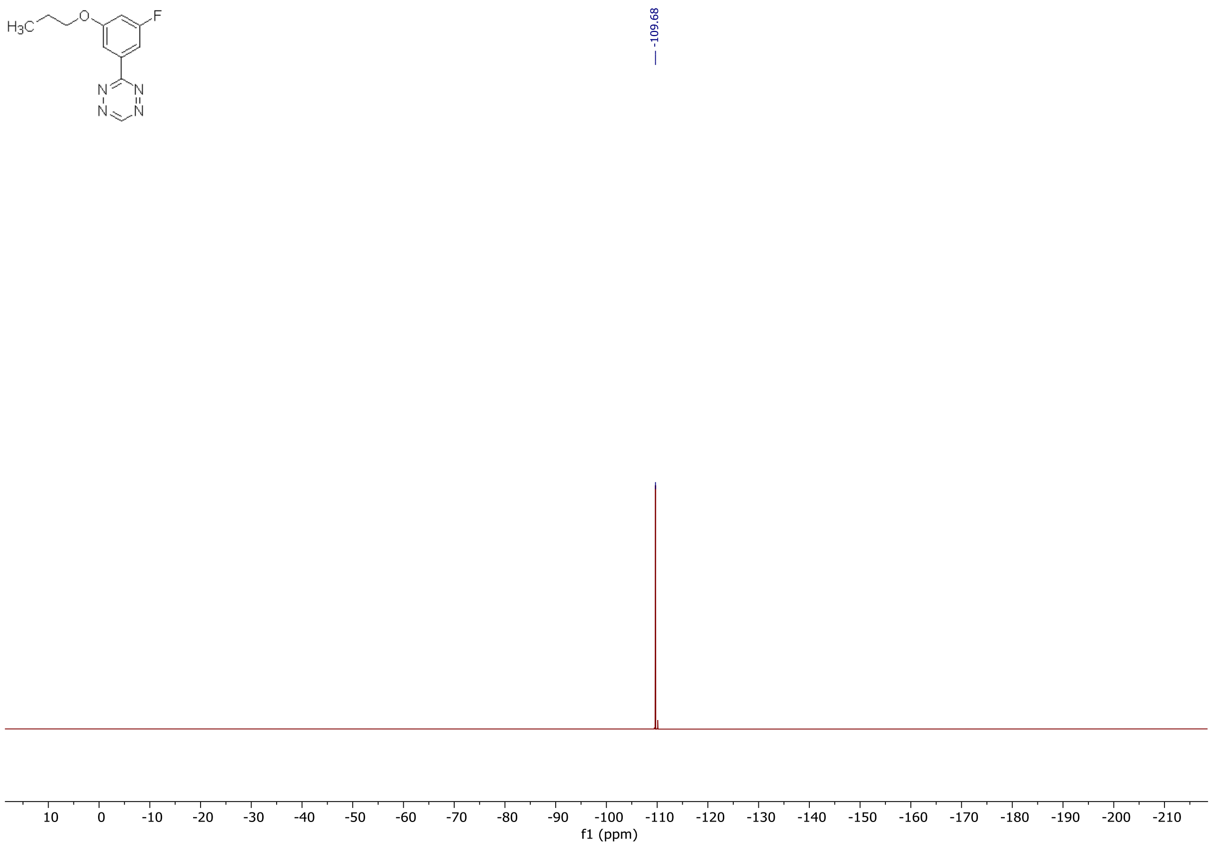
H3C
O
F
N
N
N
N
-109.68
f1 (ppm)

3-(Benzyloxy)-5-fluorobenzonitrile

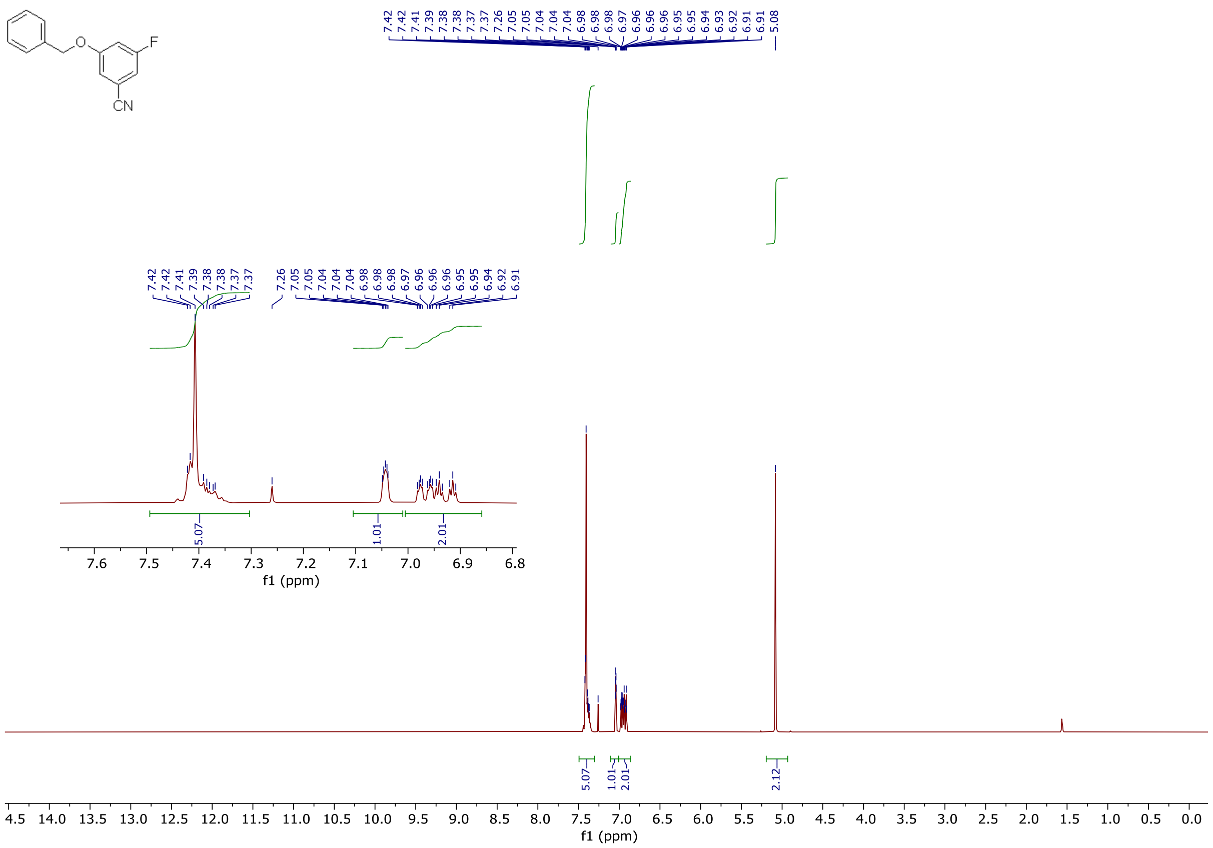

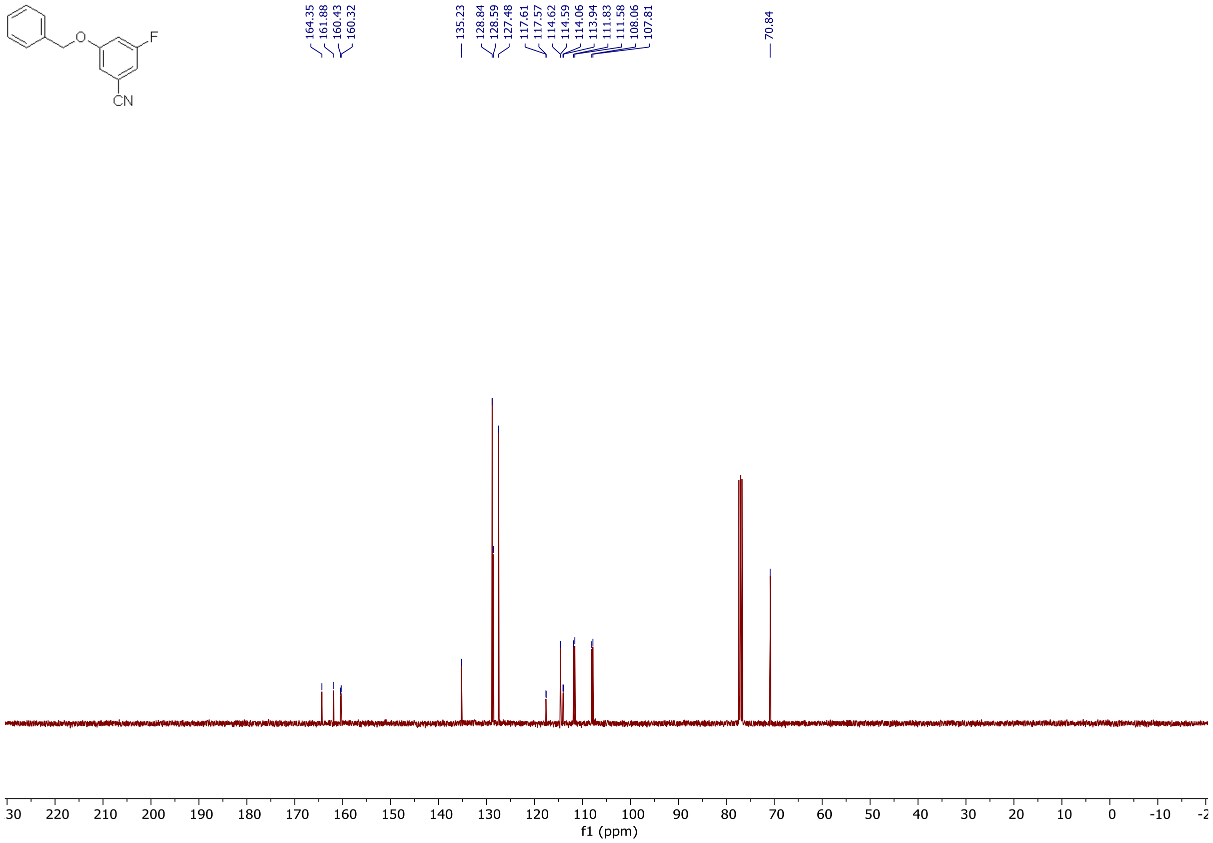

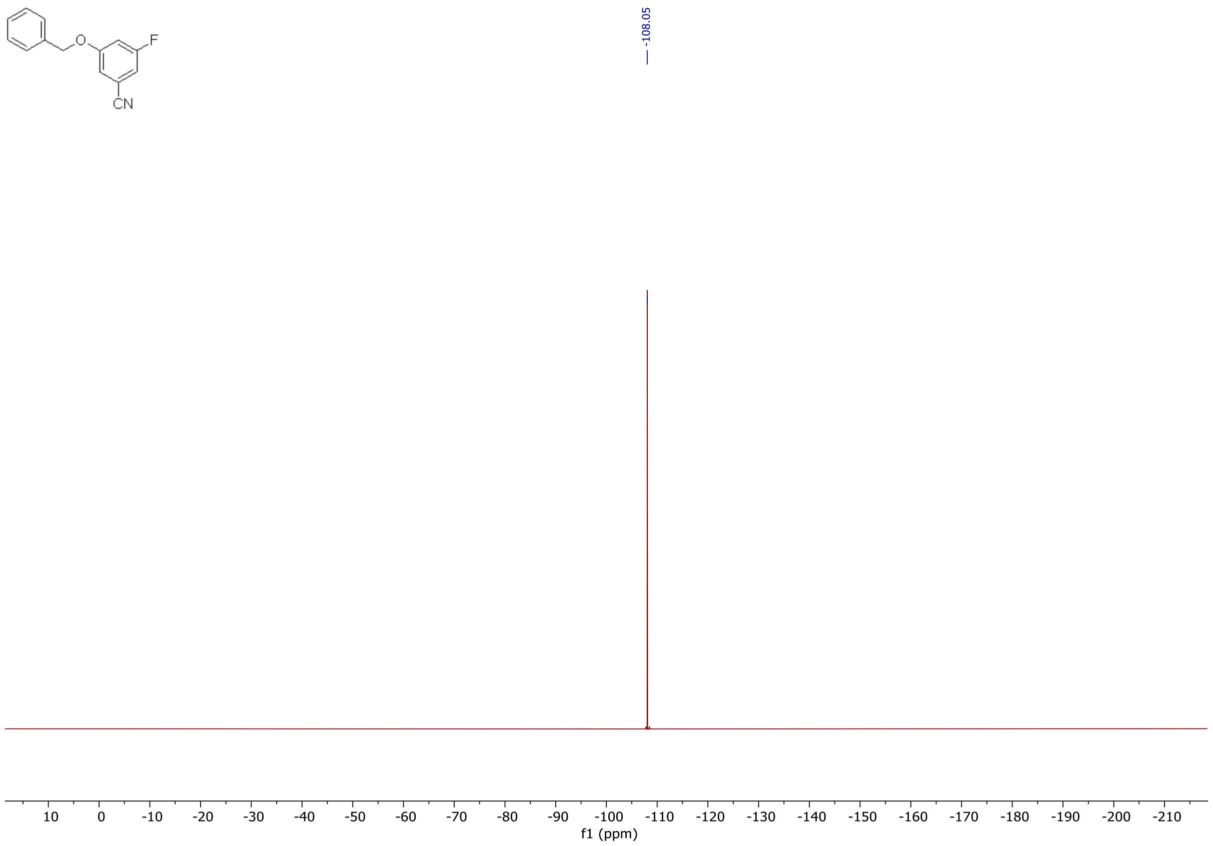

3-(3-(Benzyloxy)-5-fluorophenyl)-1,2,4,5-tetrazine

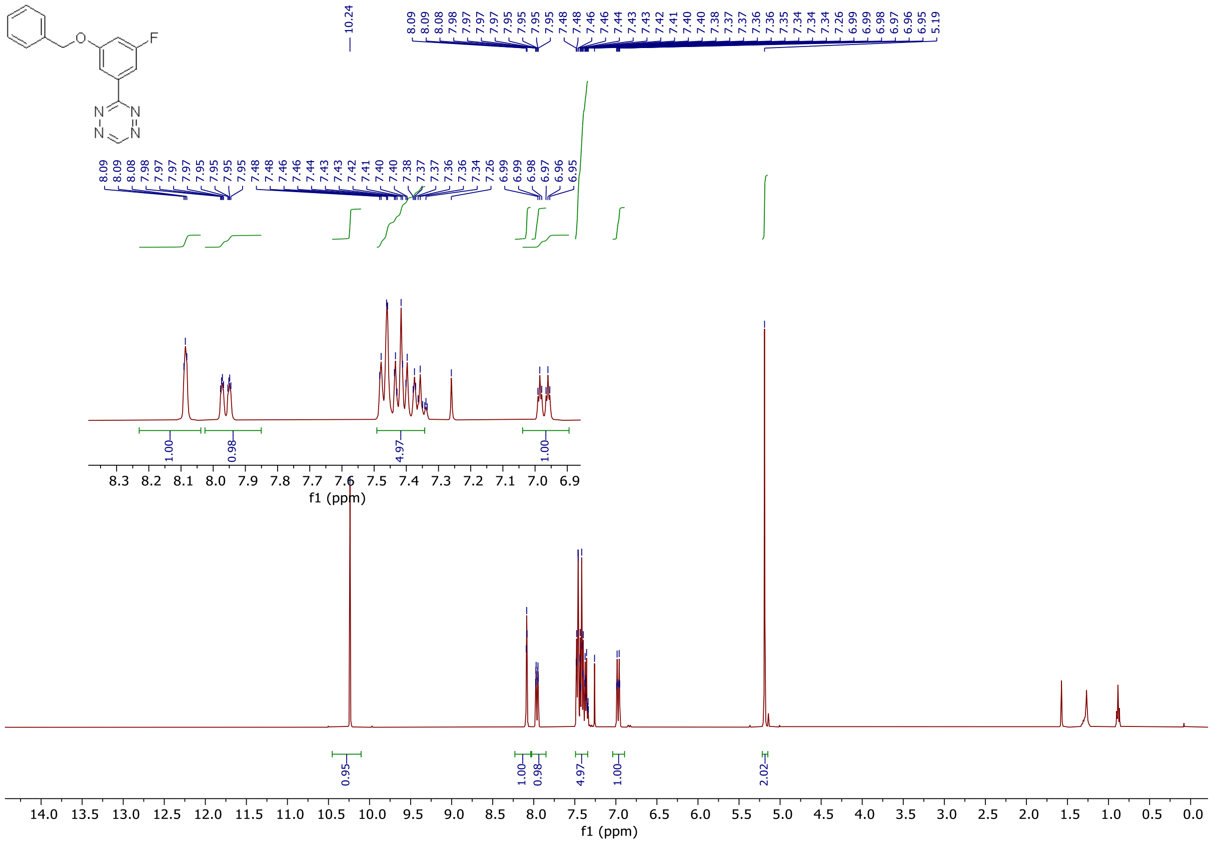

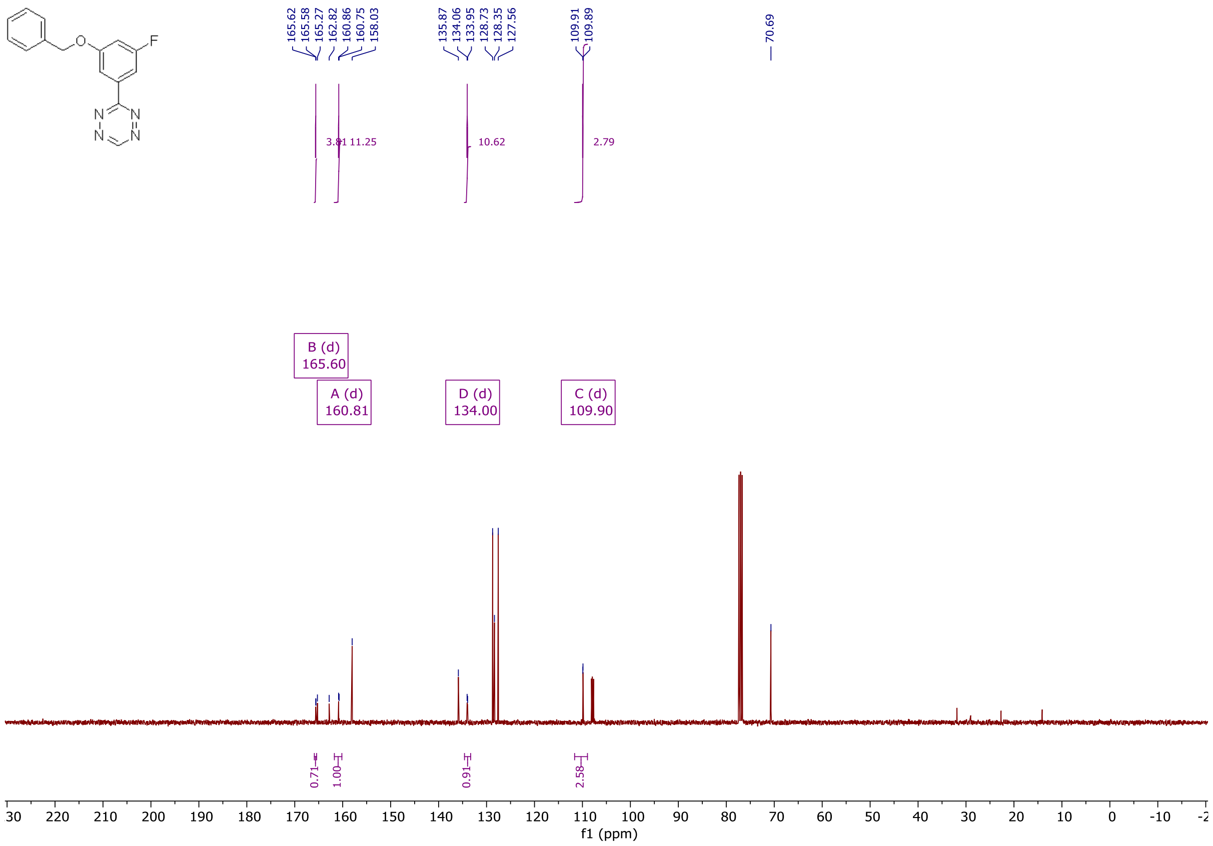
165.62
165.58
165.27
162.82
160.86
160.75
158.03
135.87
134.06
133.95
128.73
128.35
127.56
109.91
109.89
70.69
B (d)
165.60
A (d)
160.81
D (d)
134.00
C (d)
109.90
f1 (ppm)

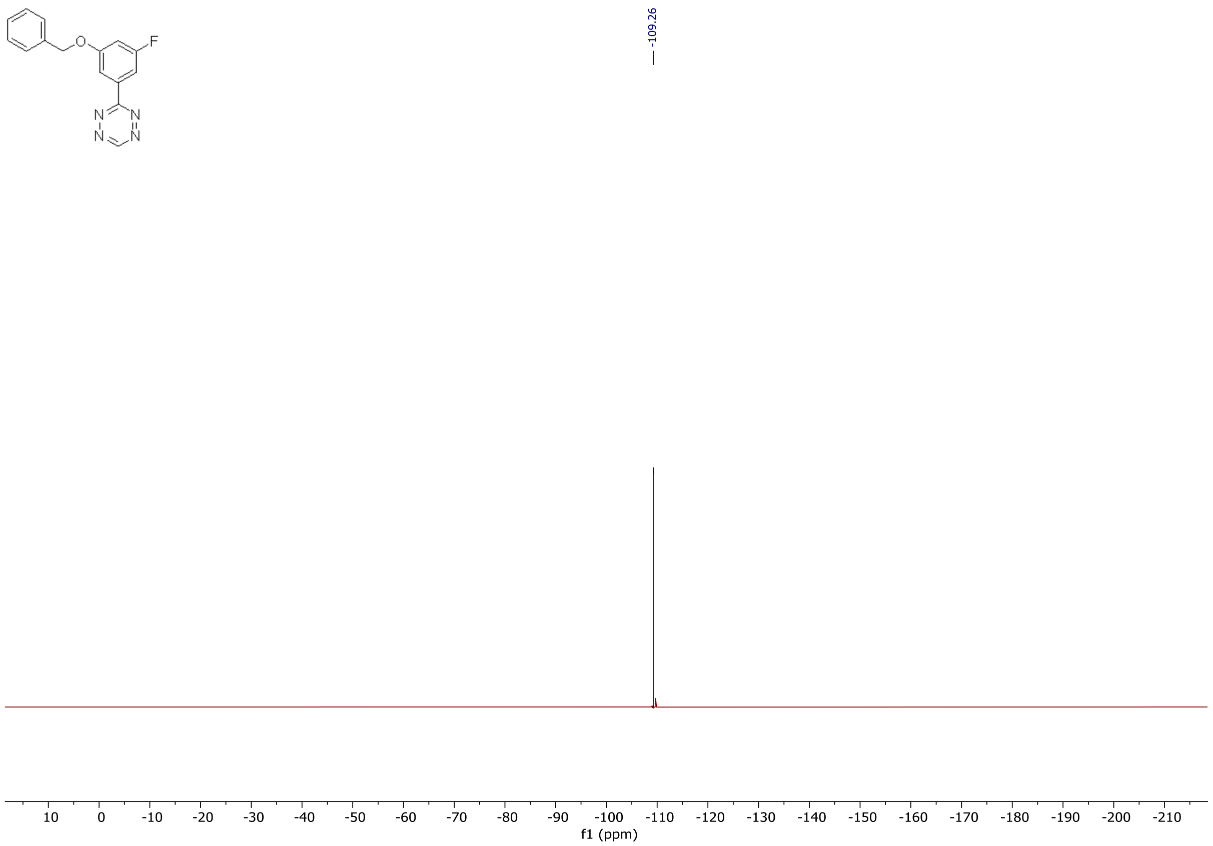
-109.26
f1 (ppm)

N-(3-Cyano-5-fluorophenyl)propionamide

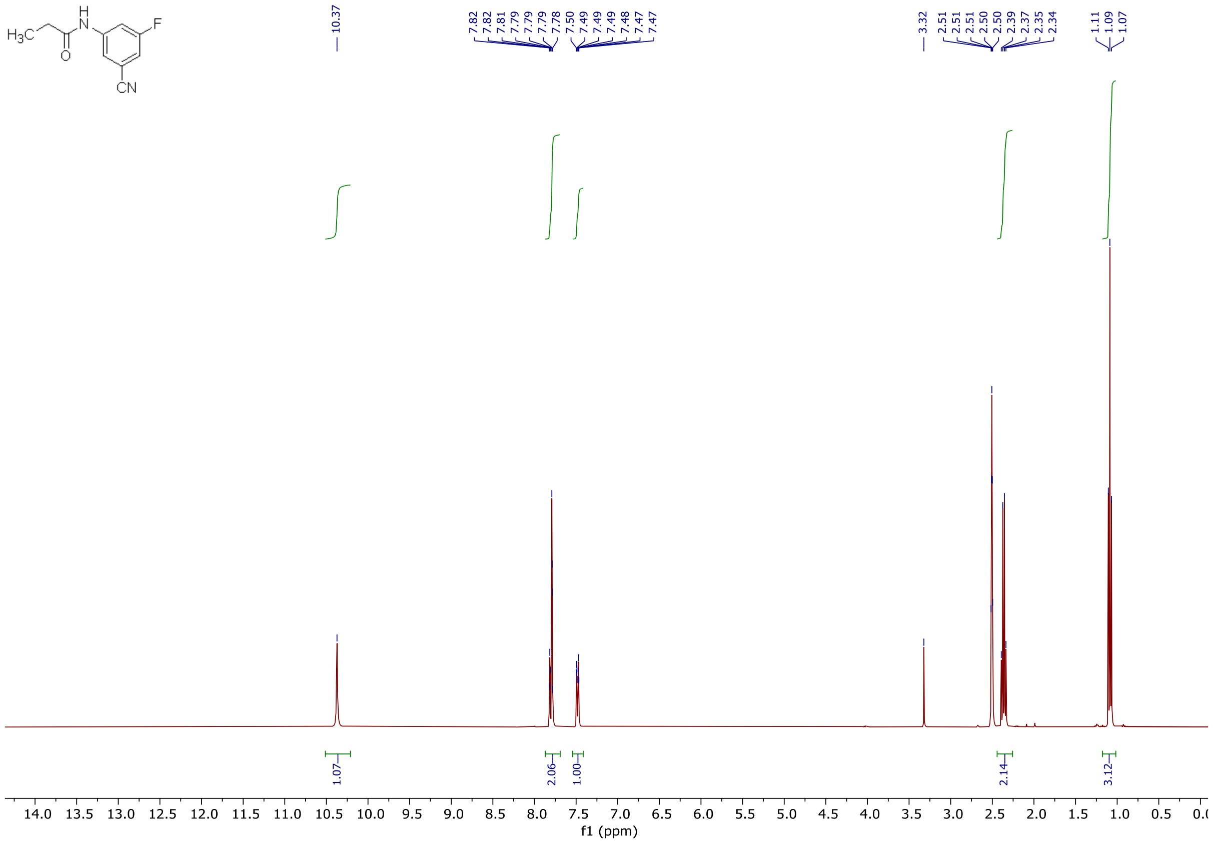

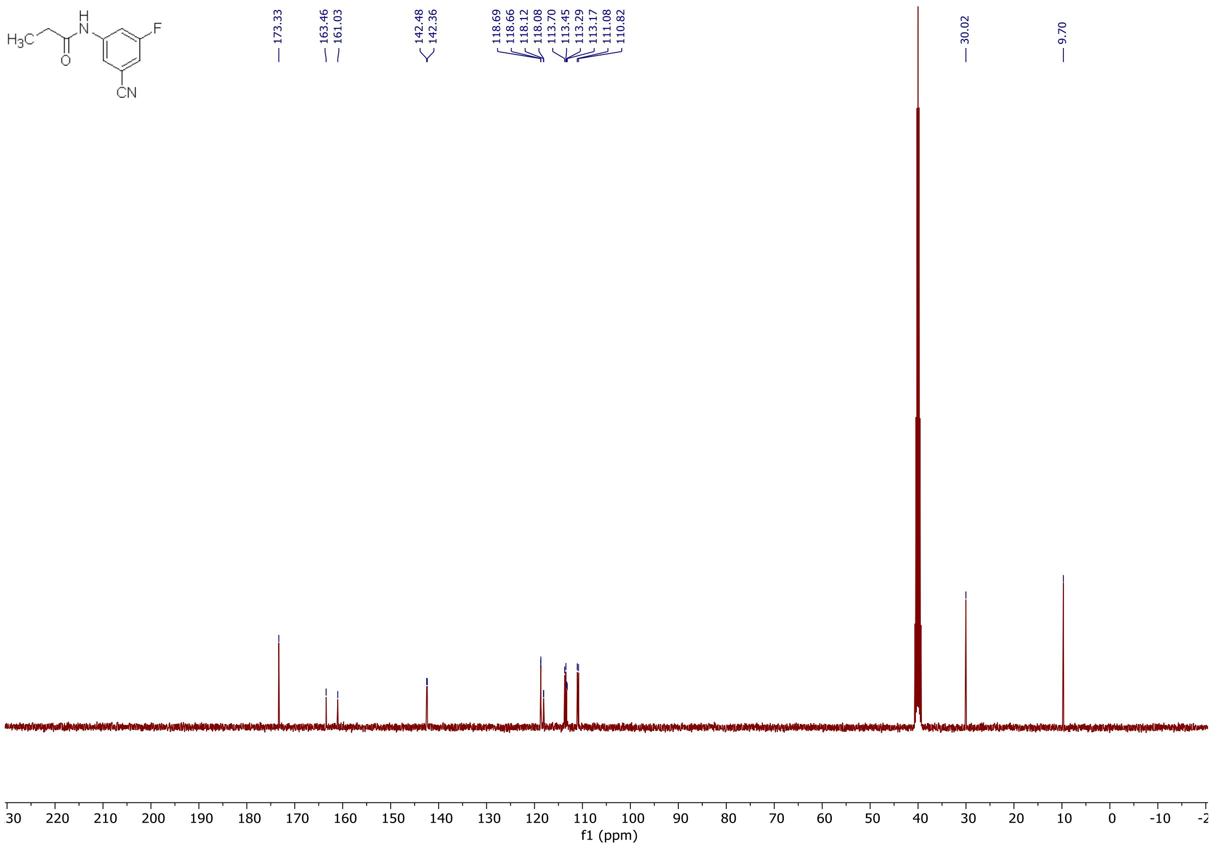

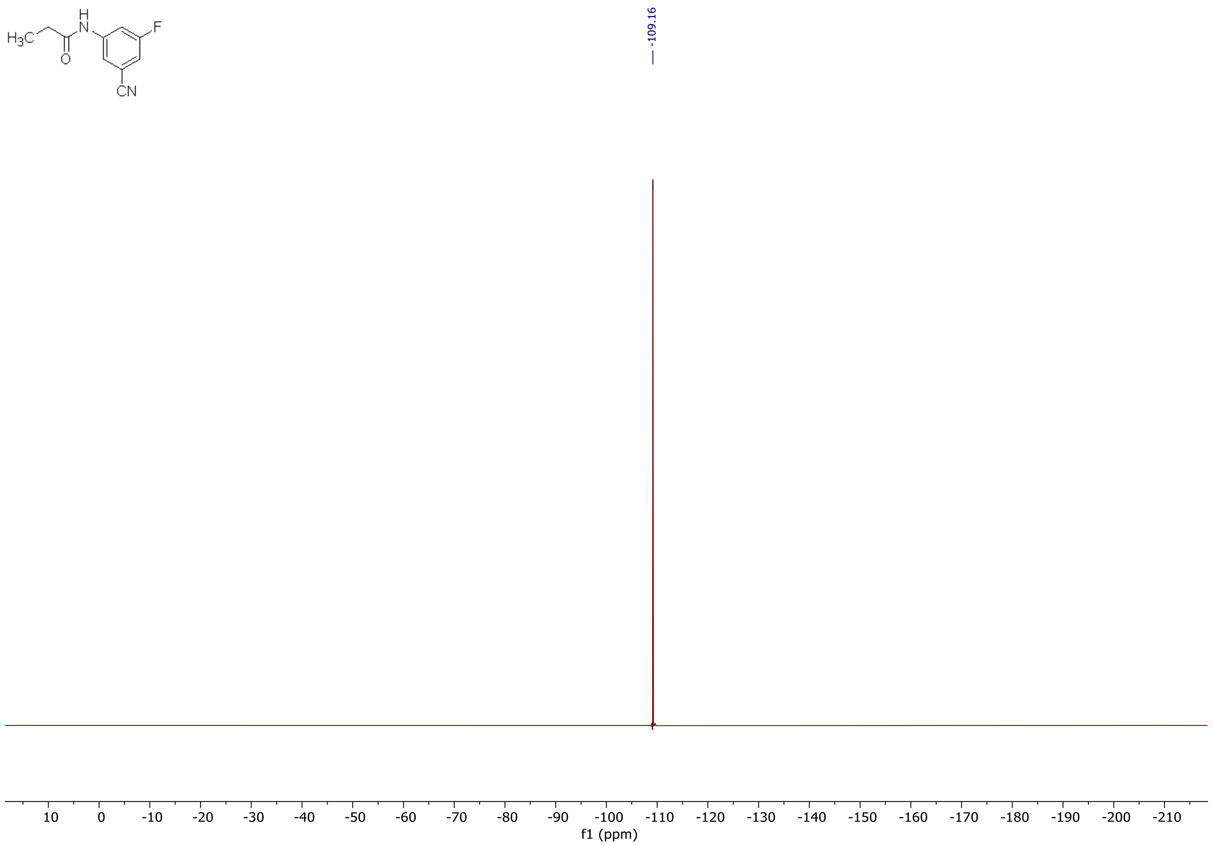

N-(3-Fluoro-5-(1,2,4,5-tetrazin-3-yl)phenyl)propionamide

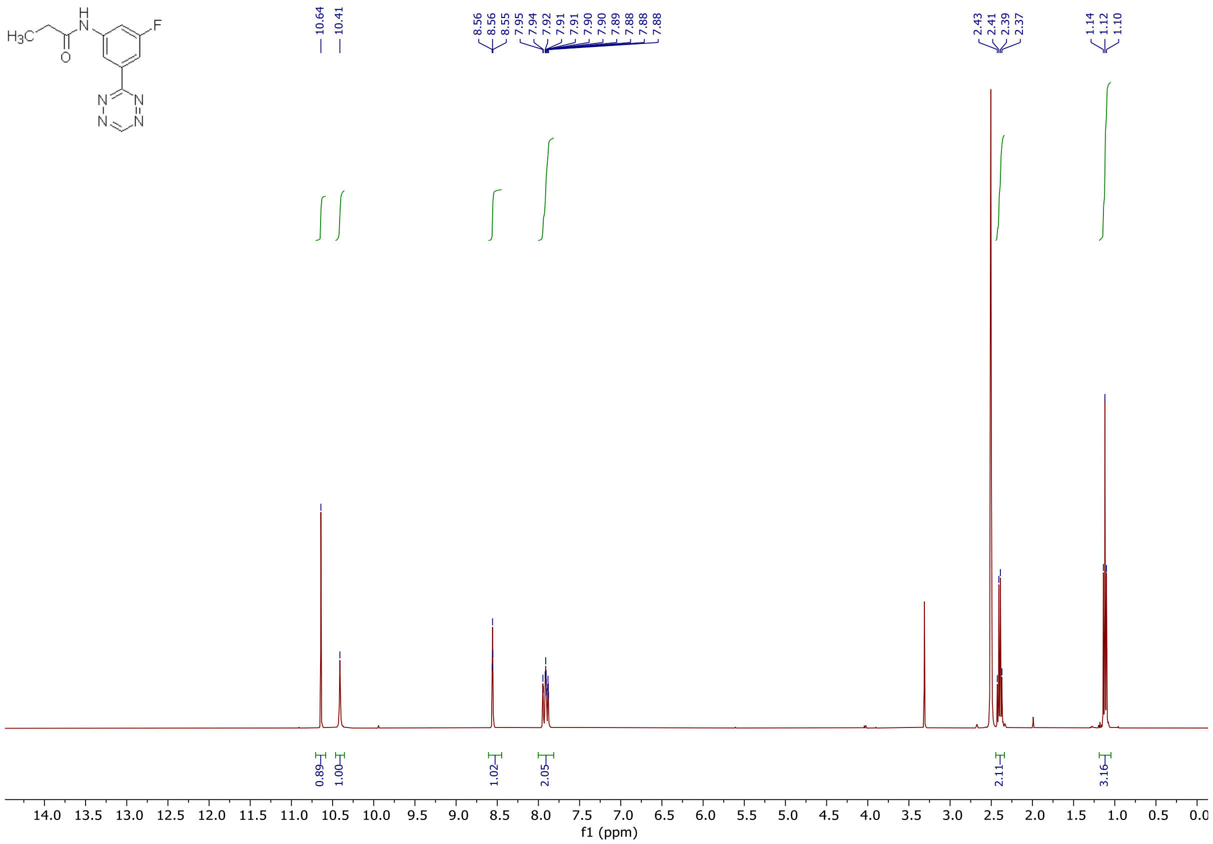

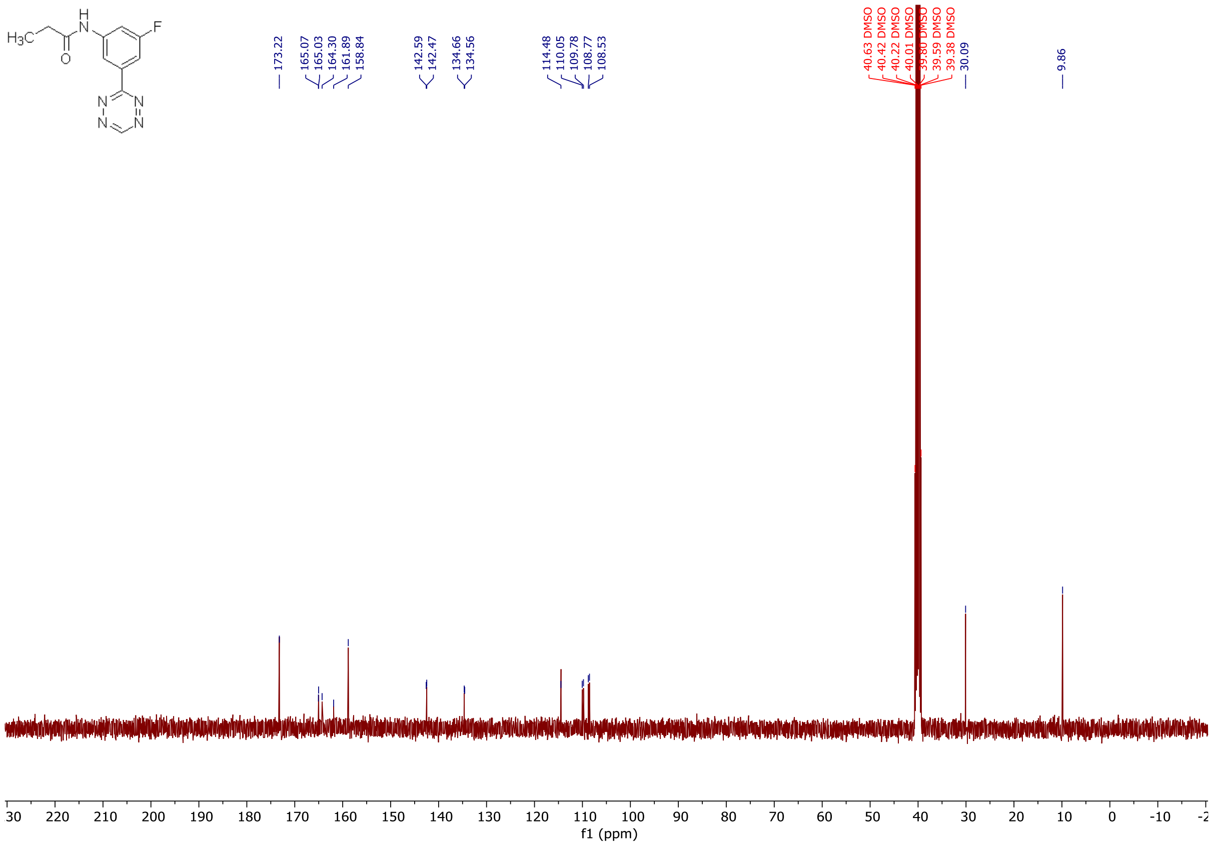
173.22
165.07
165.03
164.30
161.89
158.84
142.59
142.47
134.66
134.56
114.48
110.05
109.78
108.77
108.53
40.63 DMSO
40.42 DMSO
40.22 DMSO
40.01 DMSO
39.80 DMSO
39.59 DMSO
39.38 DMSO
30.09
9.86
f1 (ppm)

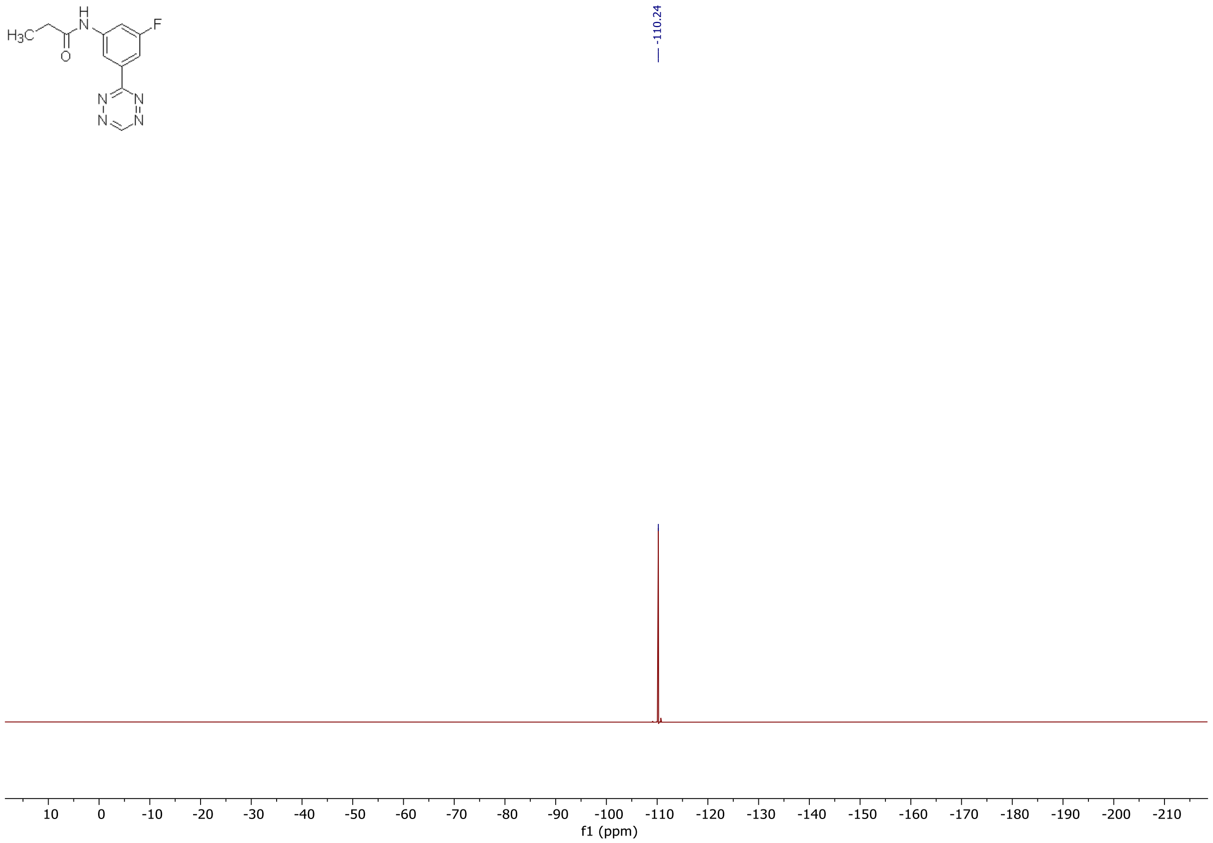
-110.24
f1 (ppm)

N-(3-Cyano-5-fluorophenyl)butyramide

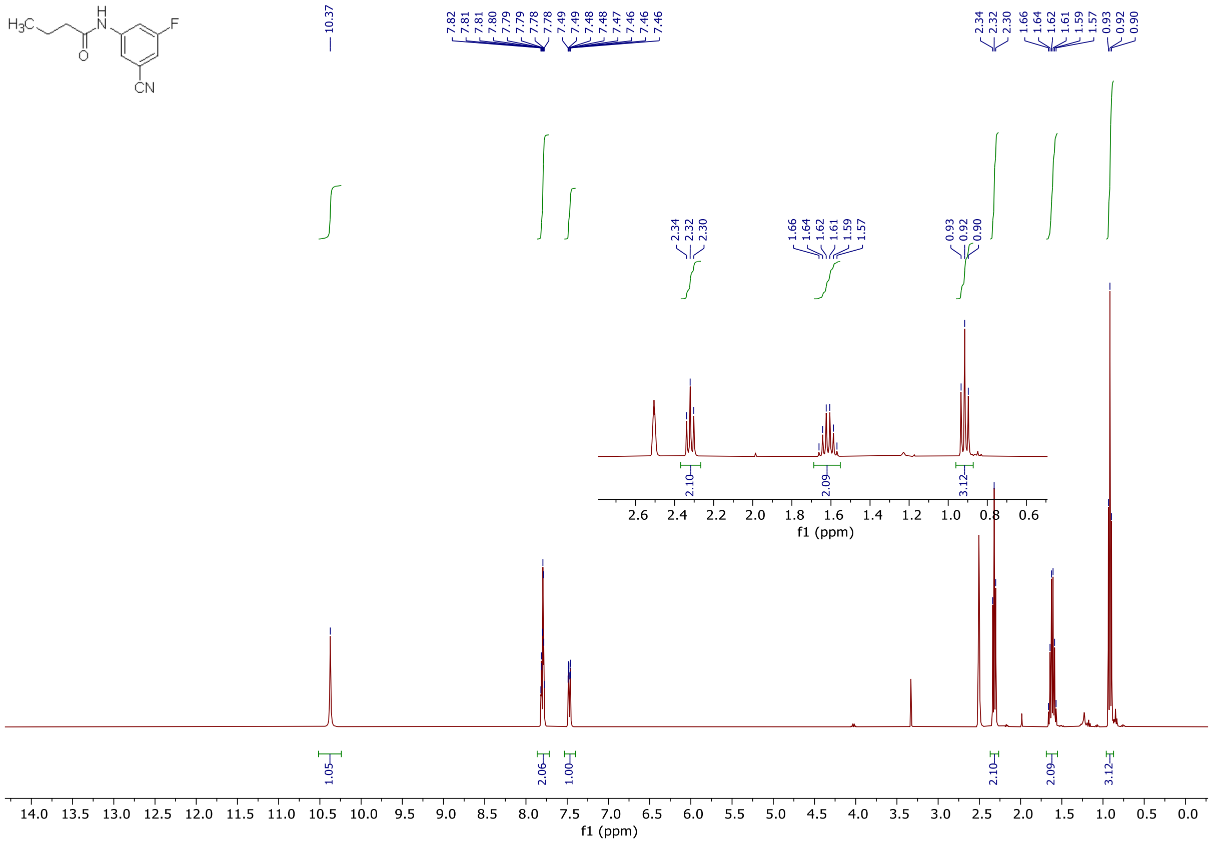

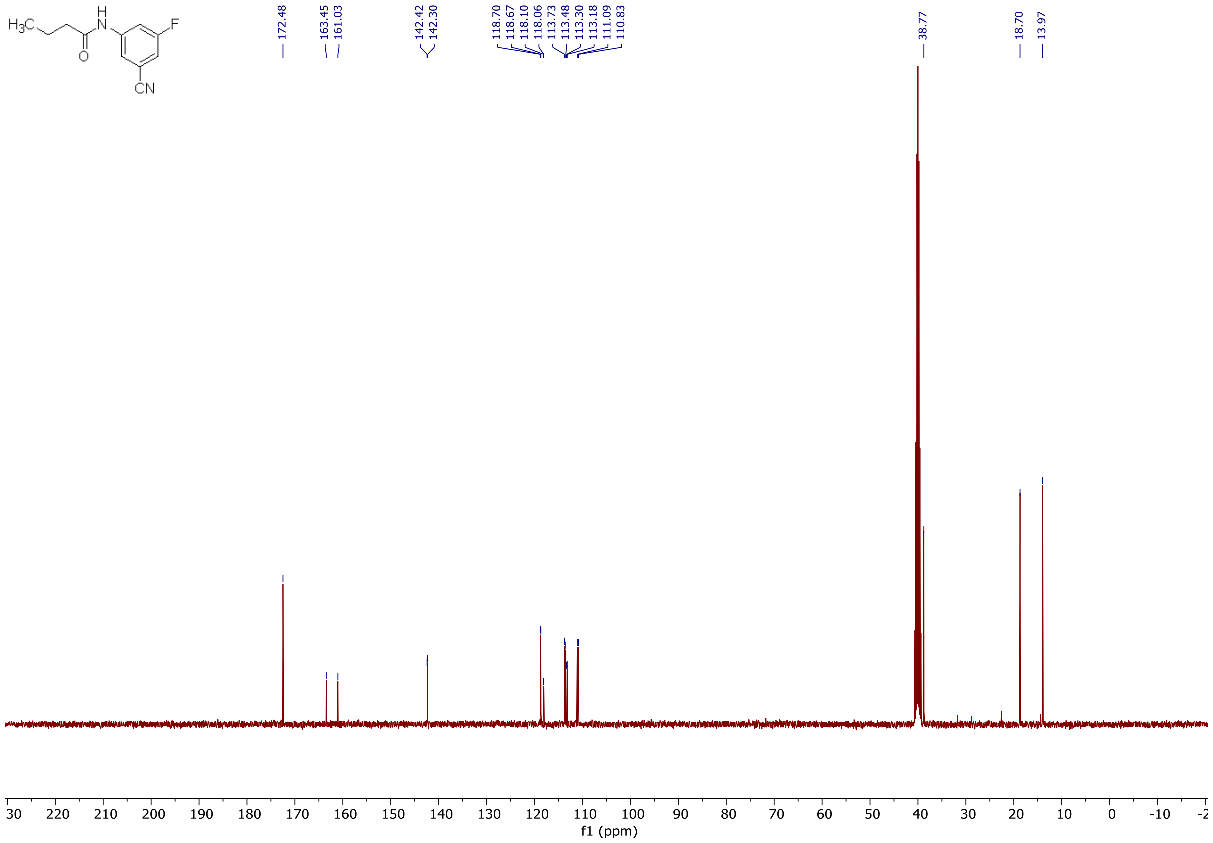

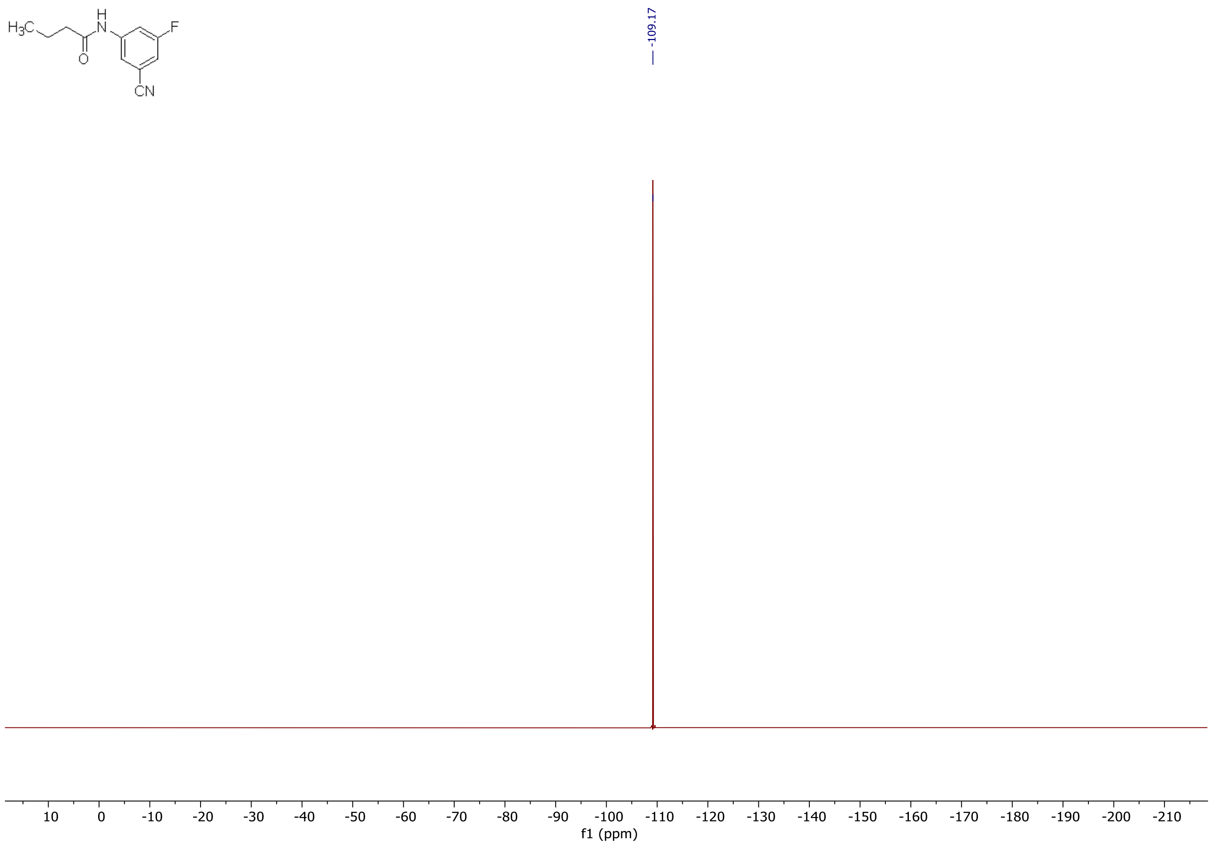

N-(3-Fluoro-5-(1,2,4,5-tetrazin-3-yl)phenyl)butyramide

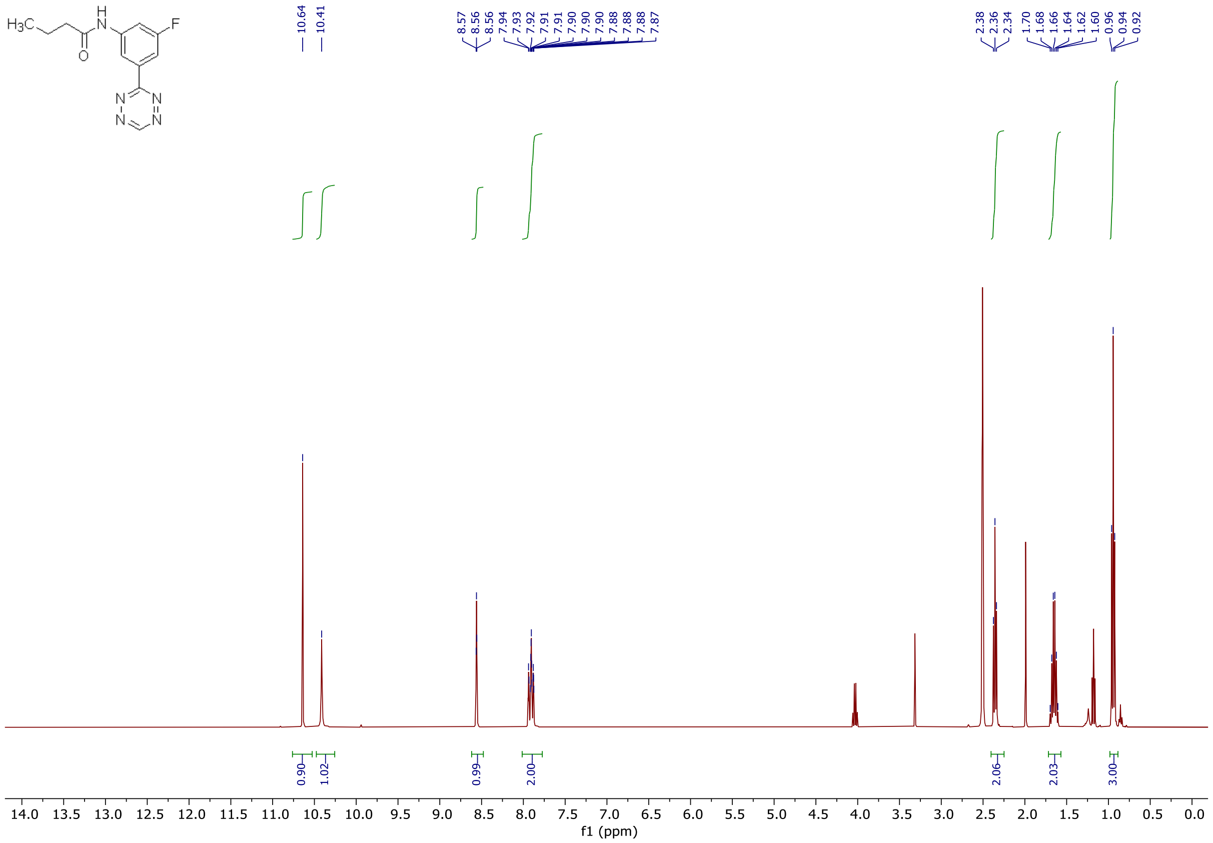

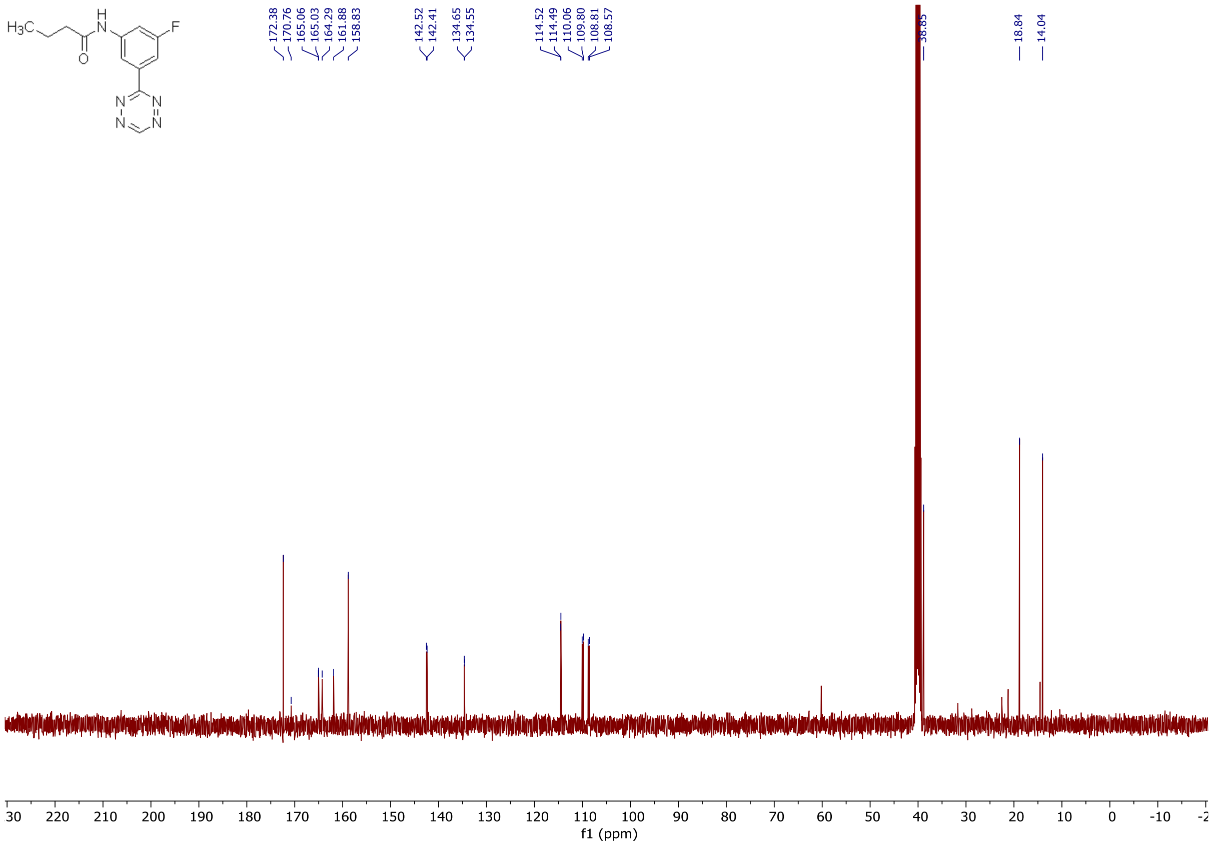
H3C
H
N
F
O
N
N
N
N
172.38
170.76
165.06
165.03
164.29
161.88
158.83
142.52
142.41
134.65
134.55
114.52
114.49
110.06
109.80
108.81
108.57
38.85
18.84
14.04
f1 (ppm)

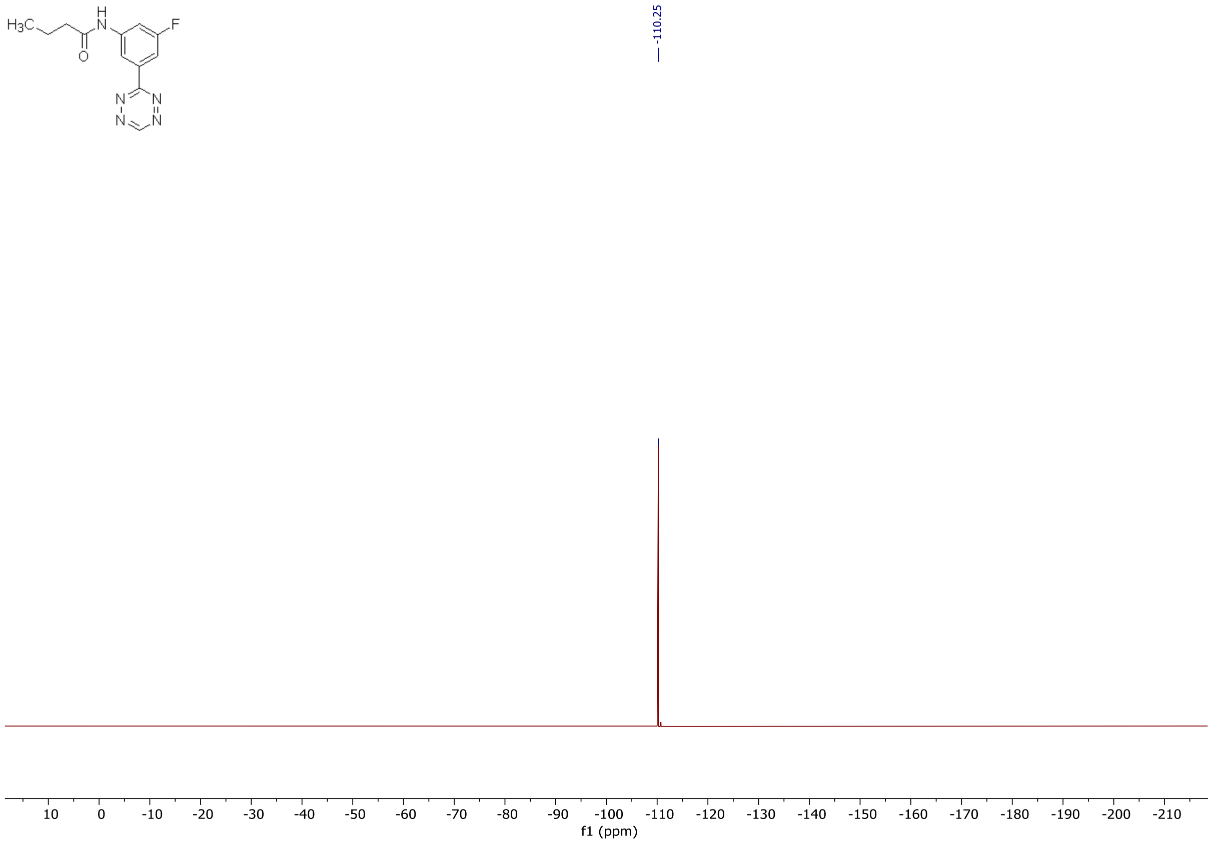
H3C
H
N
F
O
N
N
N
N
-110.25
f1 (ppm)

N-(3-Cyano-5-fluorophenyl)pentanamide

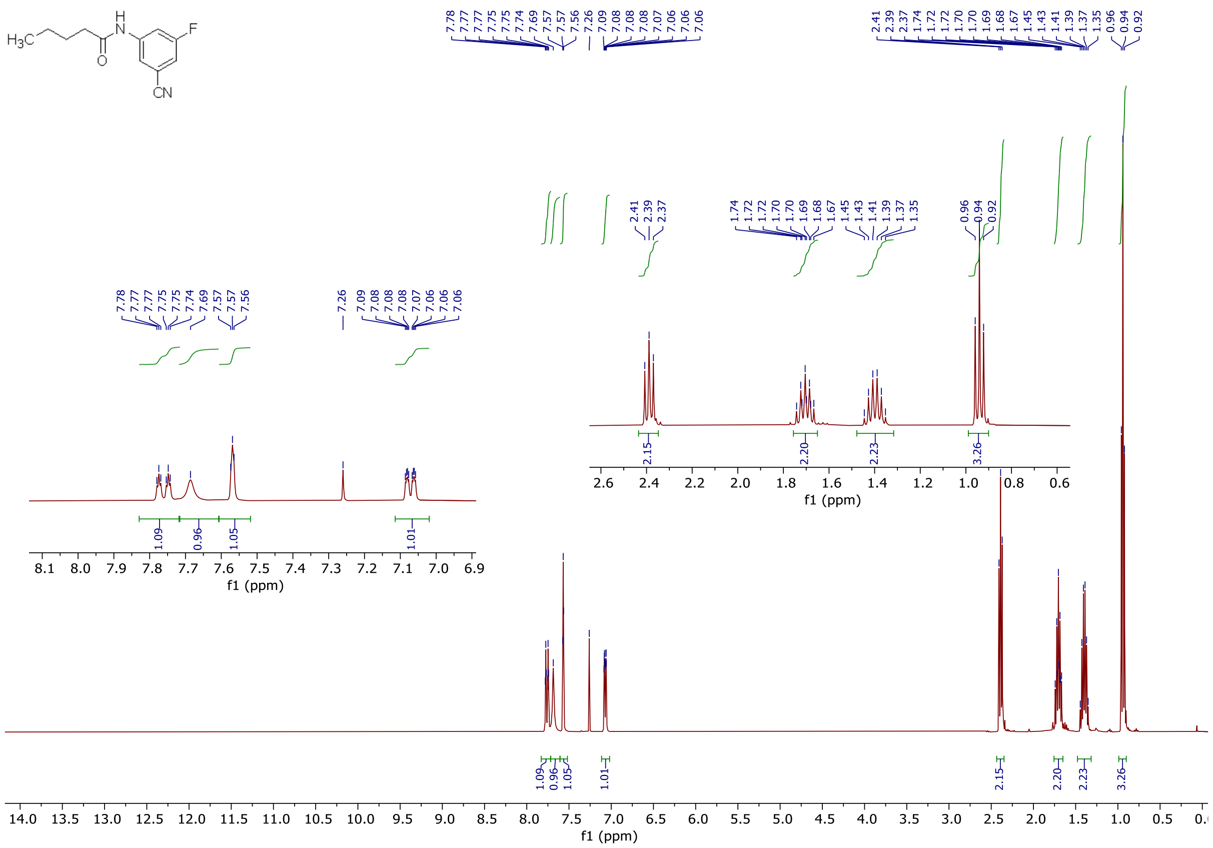

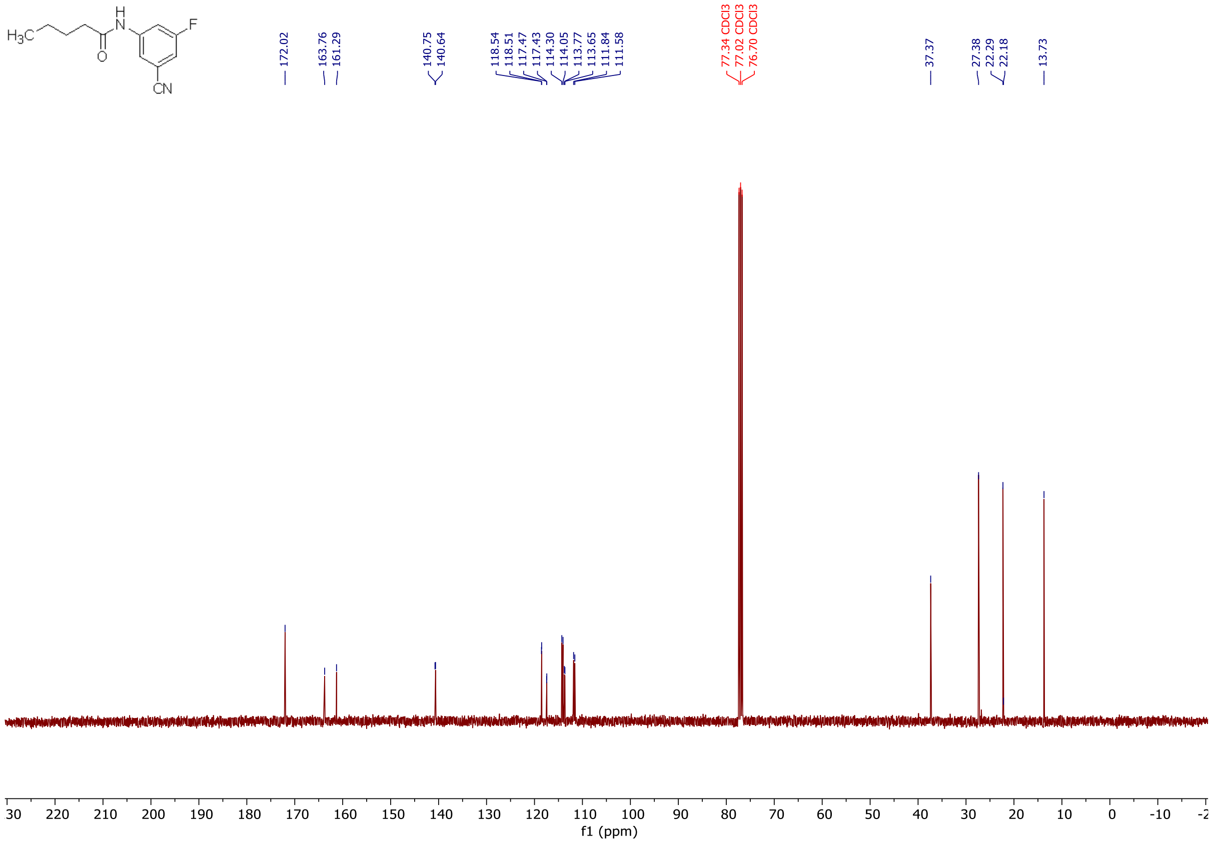

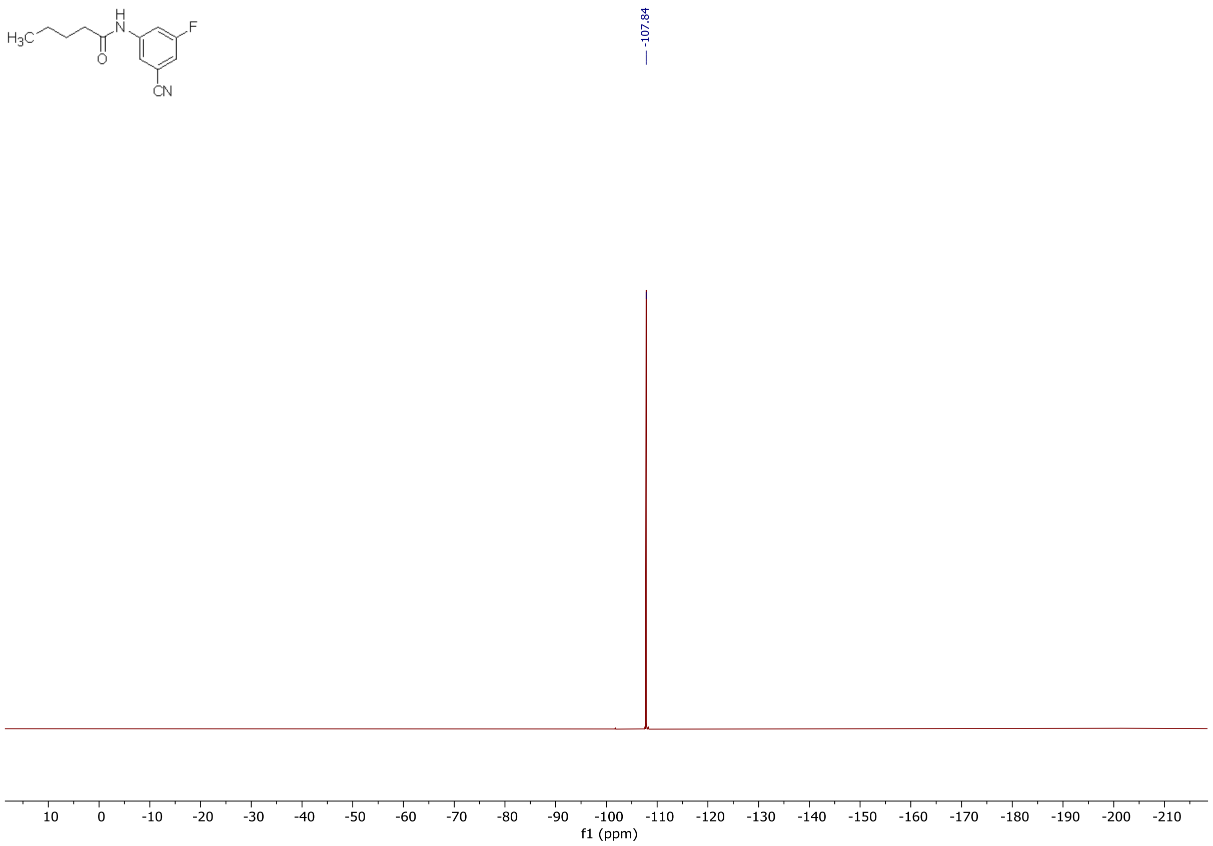

N-(3-Fluoro-5-(1,2,4,5-tetrazin-3-yl)phenyl)pentanamide

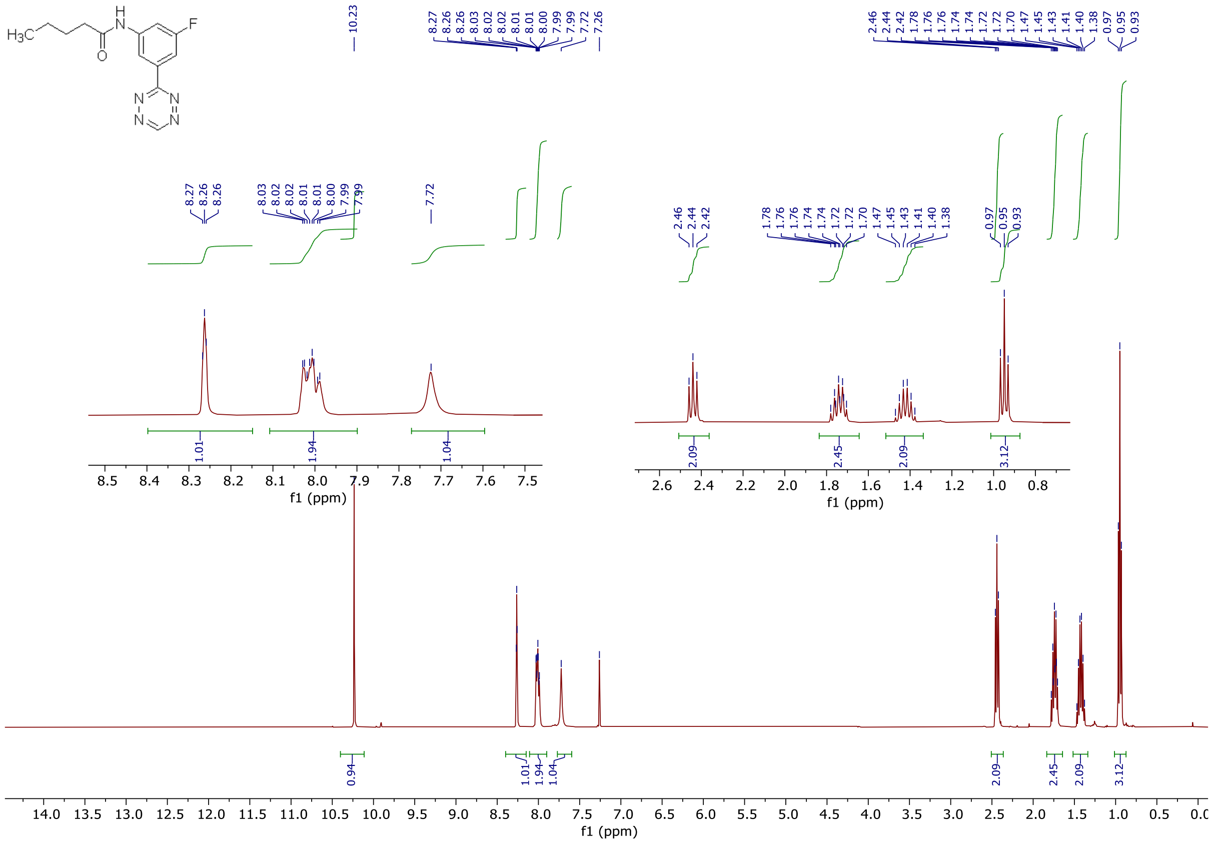

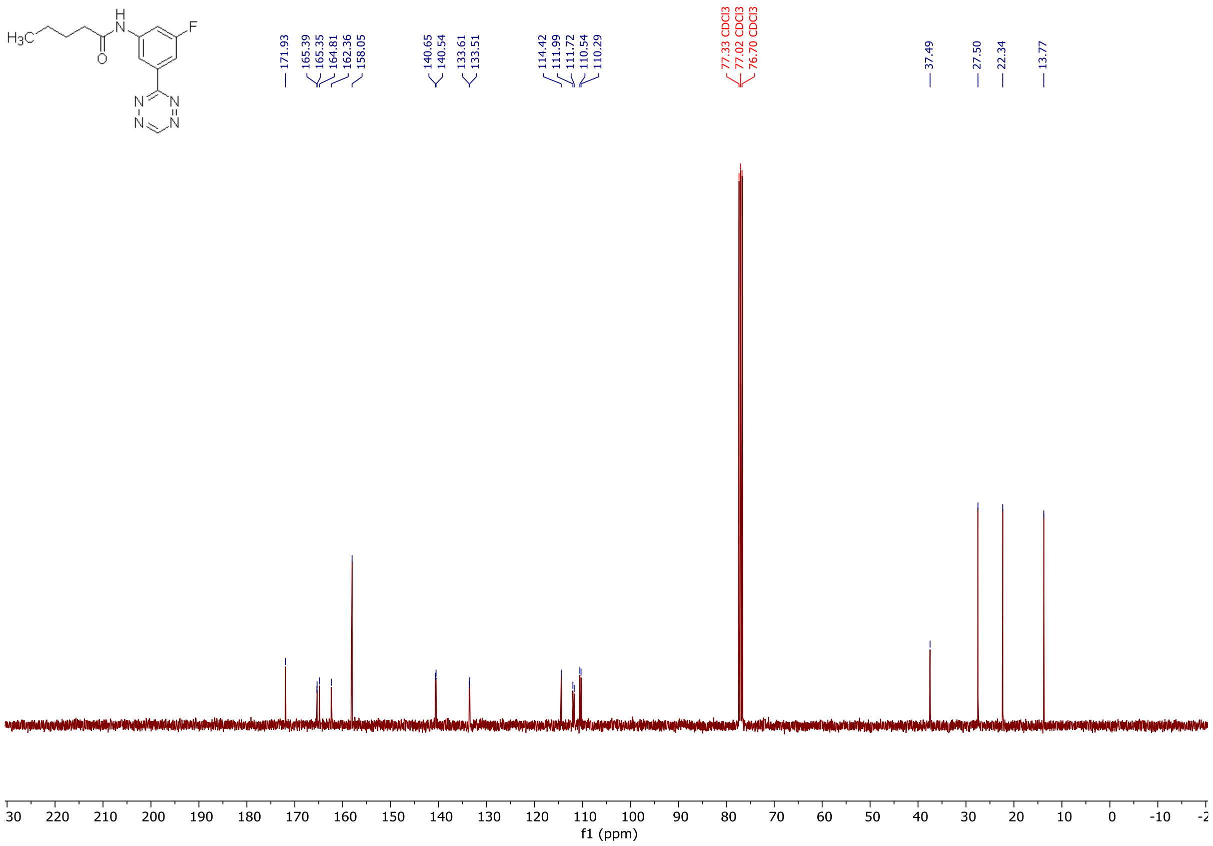
H3C
H
N
F
O
N
N
N
N
171.93
165.39
165.35
164.81
162.36
158.05
140.65
140.54
133.61
133.51
114.42
111.99
111.72
110.54
110.29
77.33 CDCl3
77.02 CDCl3
76.70 CDCl3
37.49
27.50
22.34
13.77
f1 (ppm)

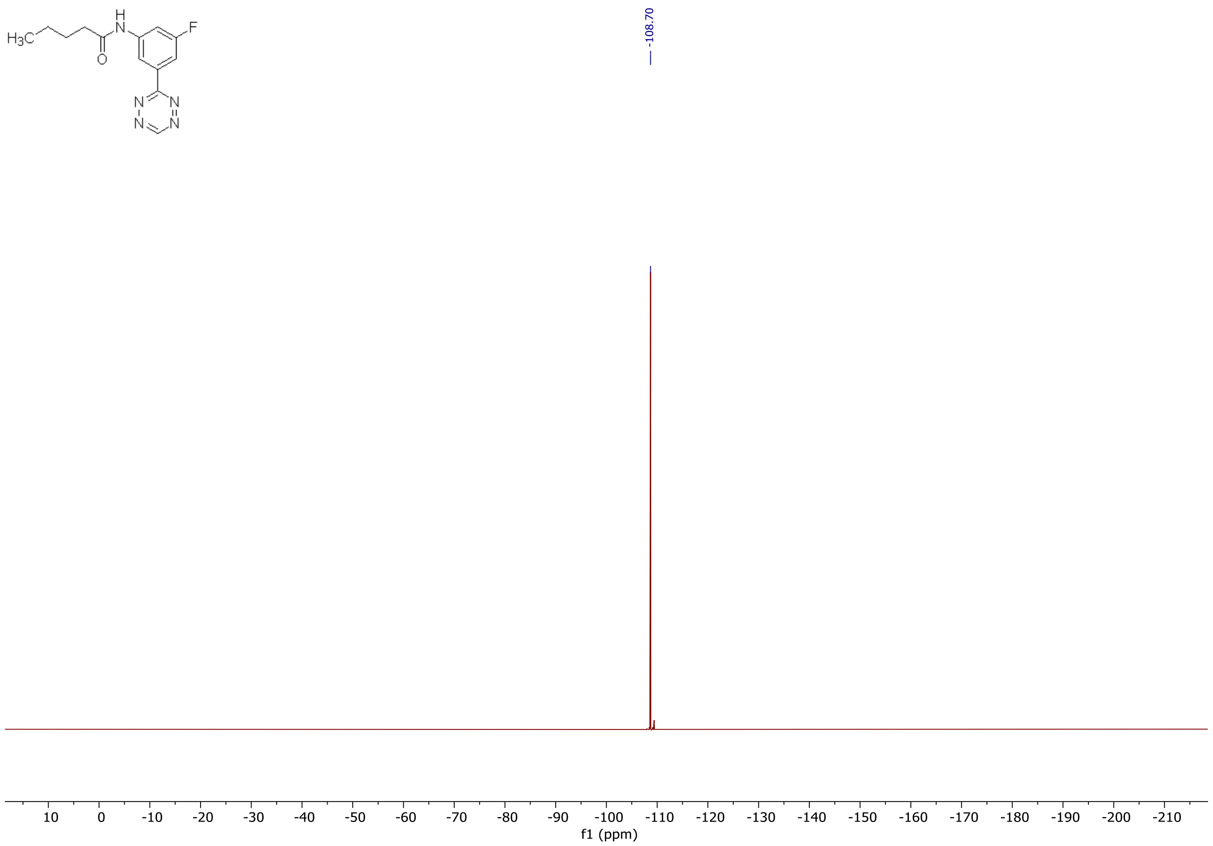
H3C
H
N
F
O
N
N
N
N
-108.70
f1 (ppm)

3-Cyano-N-ethyl-5-fluorobenzamide

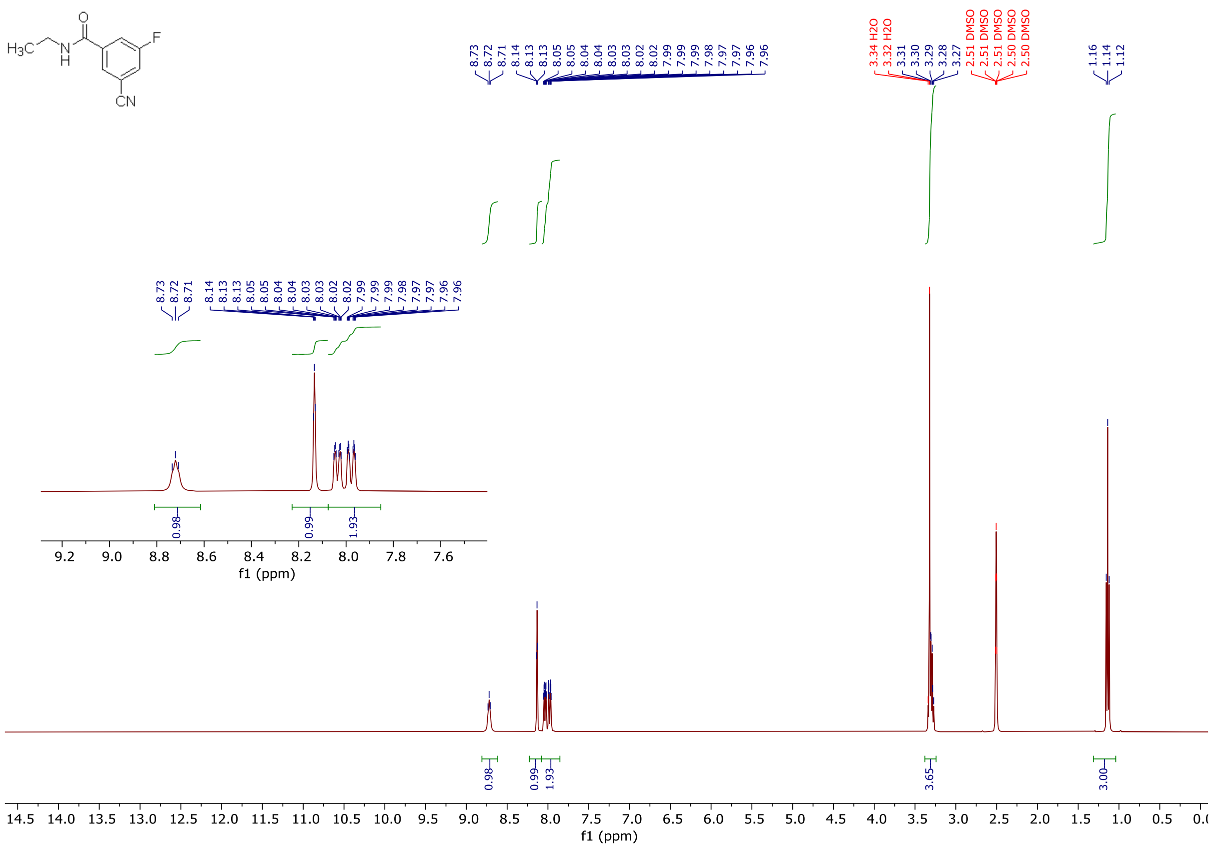

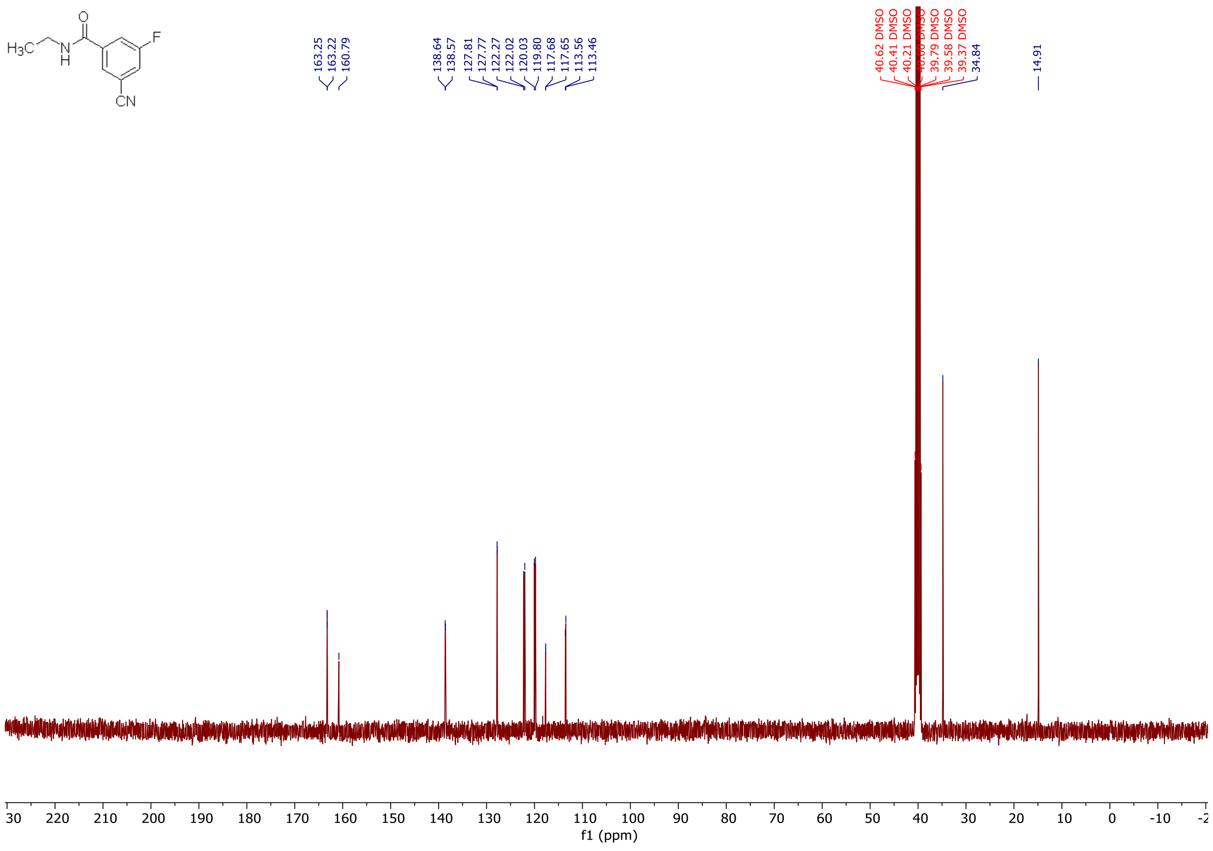

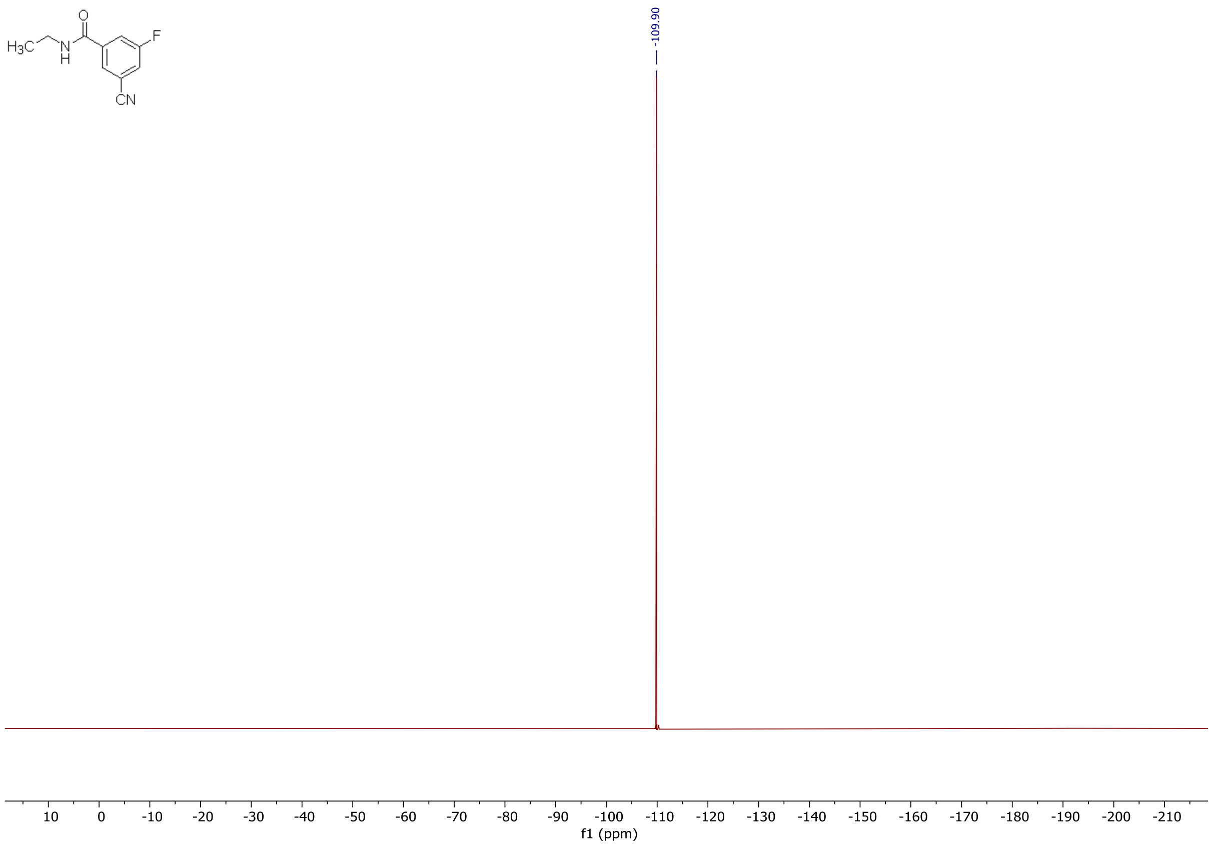

N-Ethyl-3-fluoro-5-(1,2,4,5-tetrazin-3-yl)benzamide

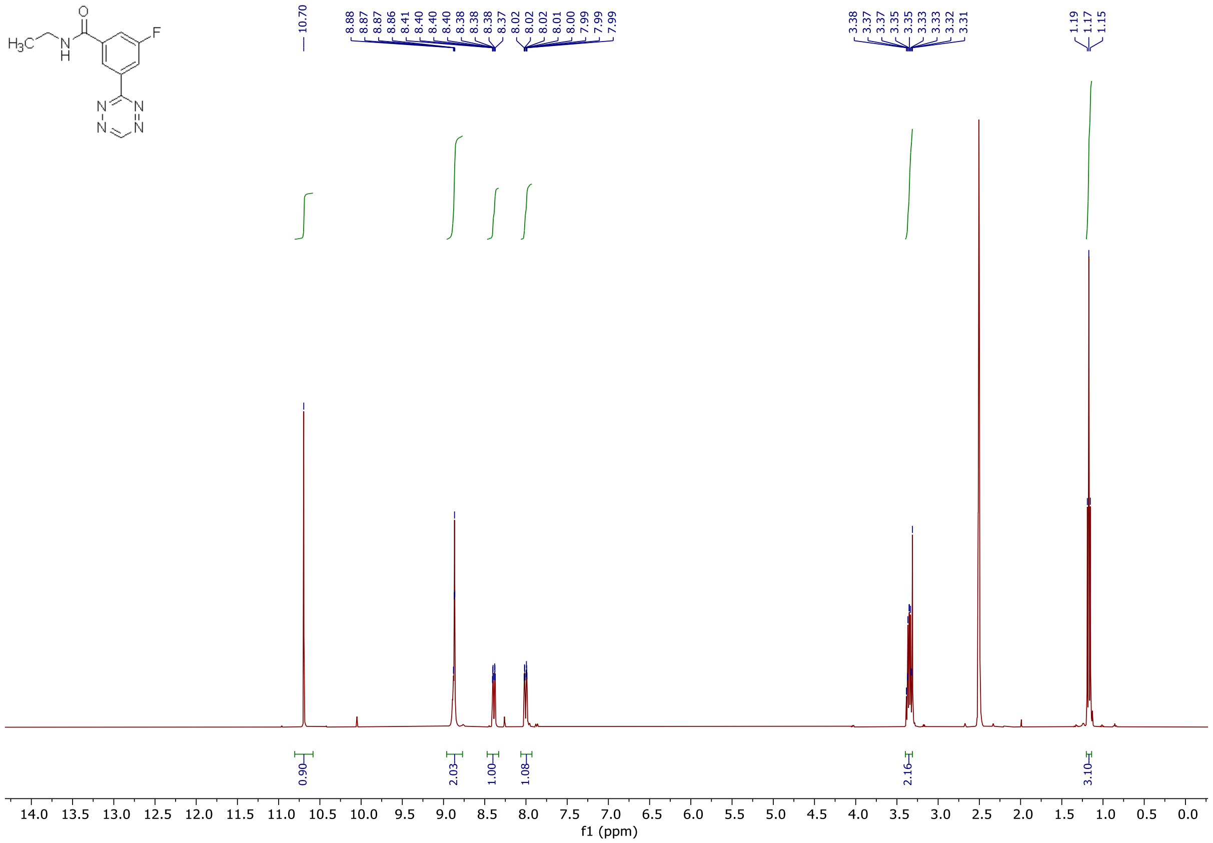

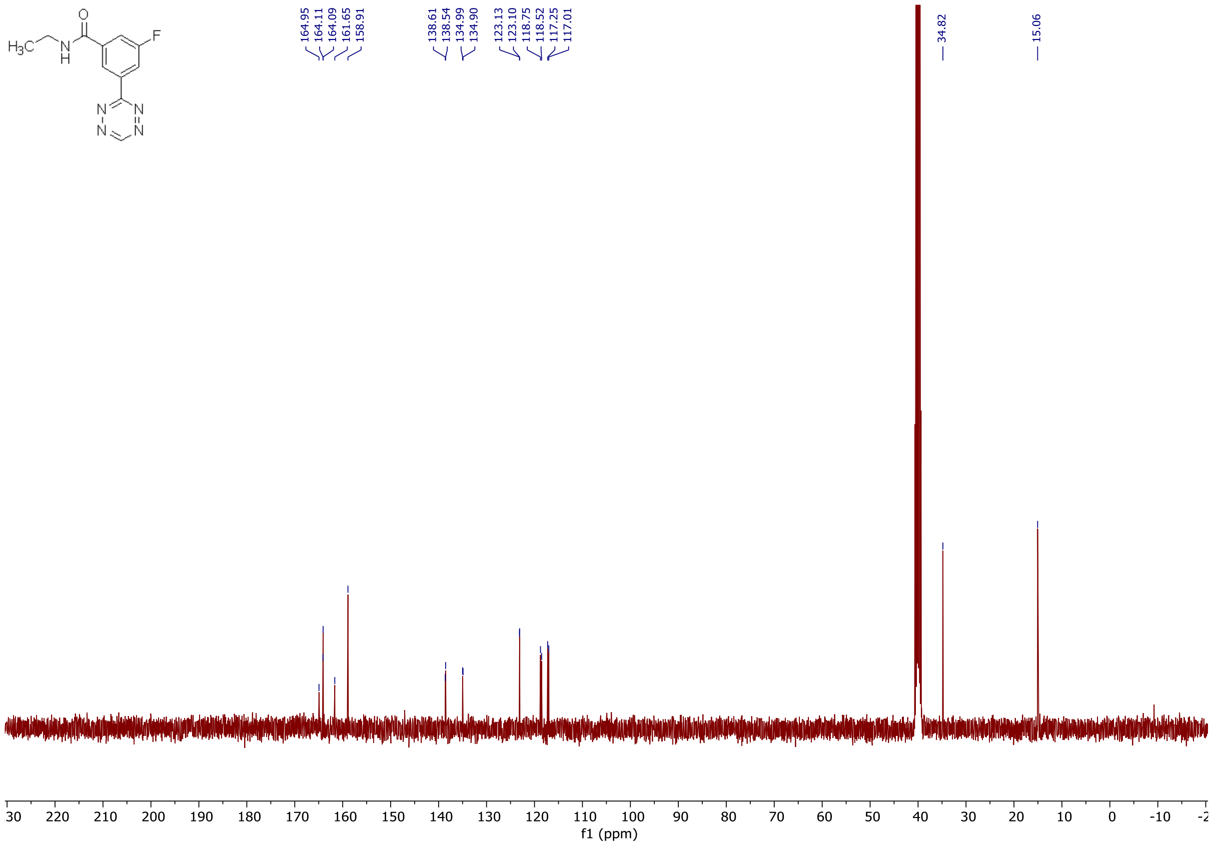
164.95
164.11
164.09
161.65
158.91
138.61
138.54
134.99
134.90
123.13
123.10
118.75
118.52
117.25
117.01
34.82
15.06
f1 (ppm)

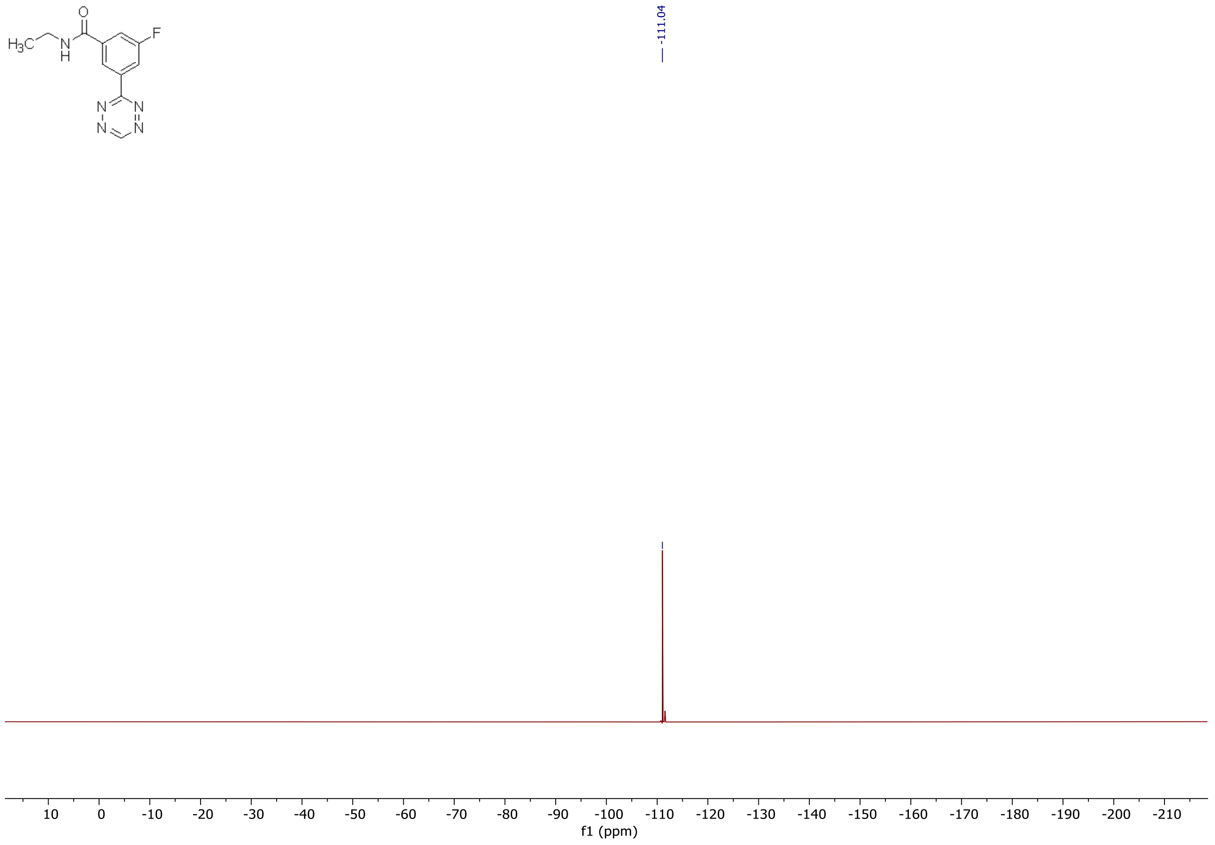
-111.04
f1 (ppm)

3-Cyano-5-fluoro-N-propylbenzamide

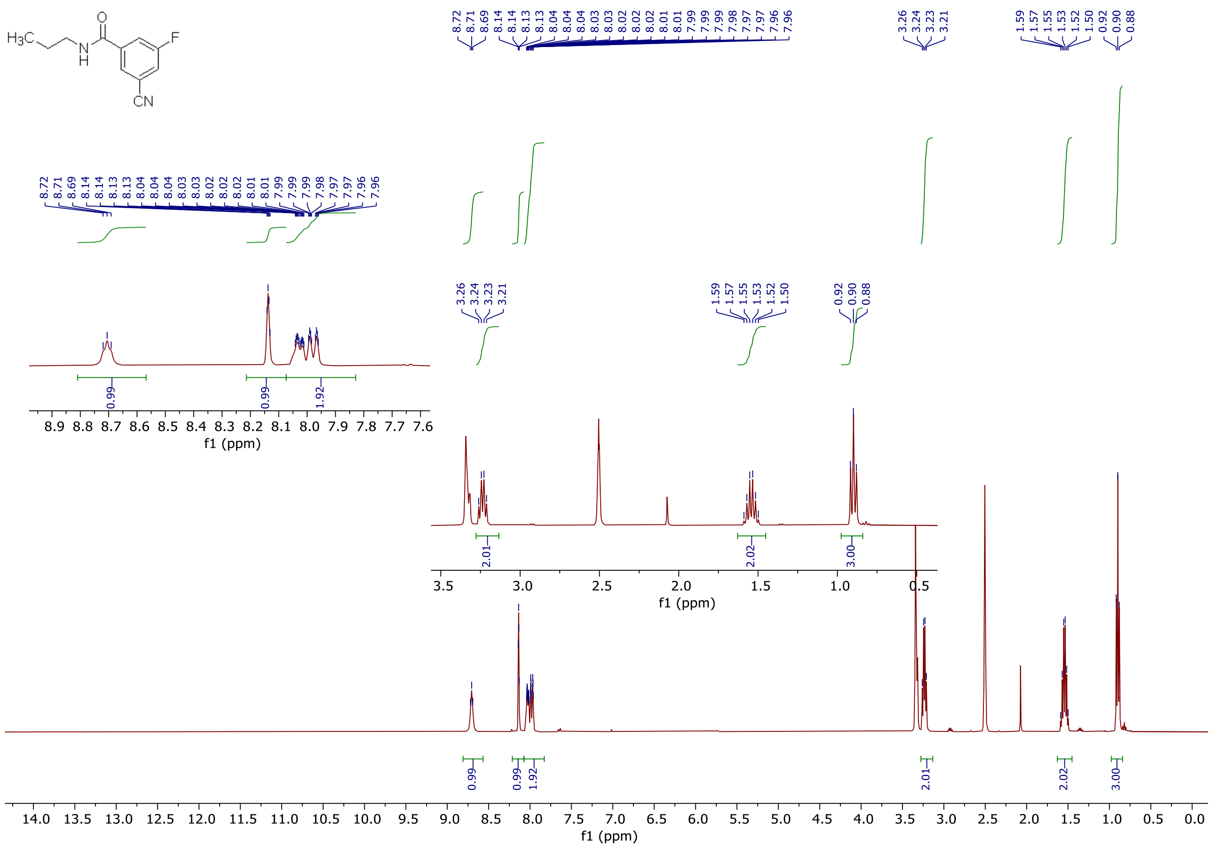

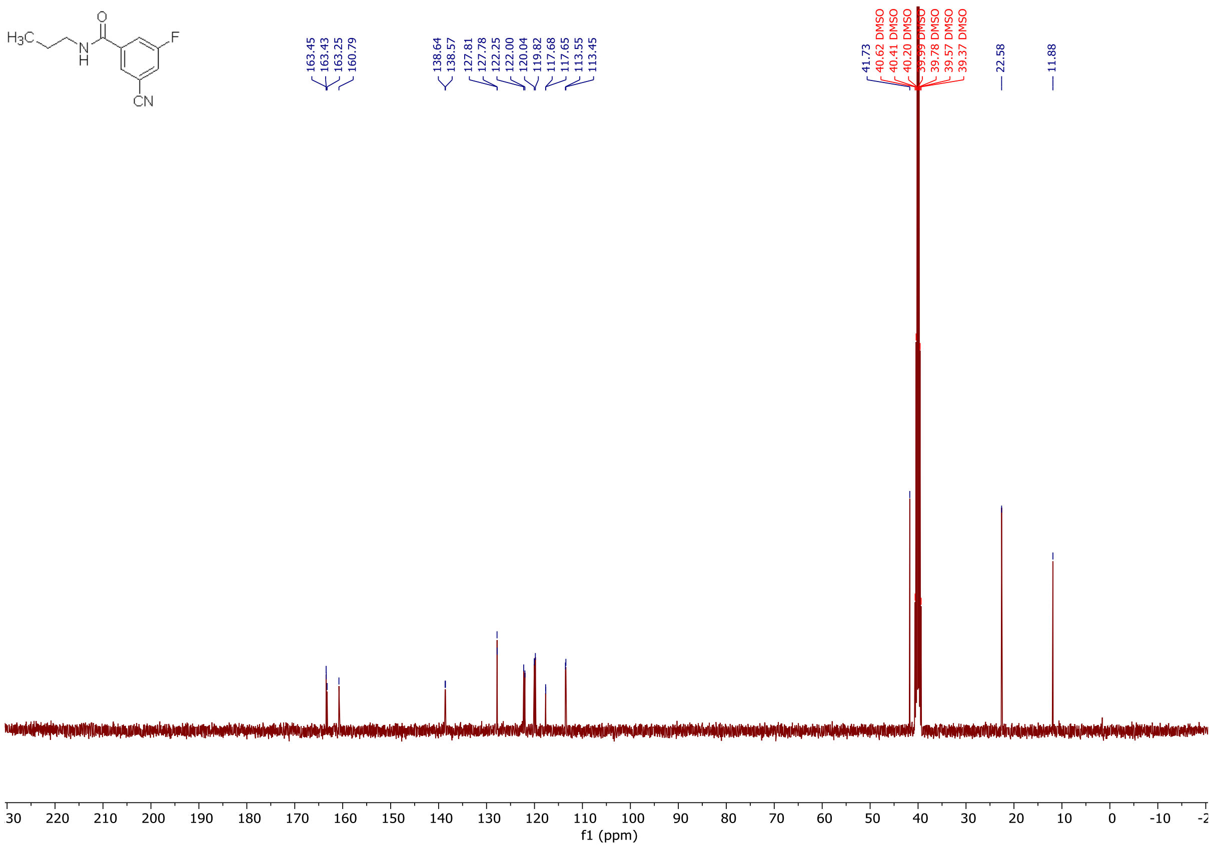

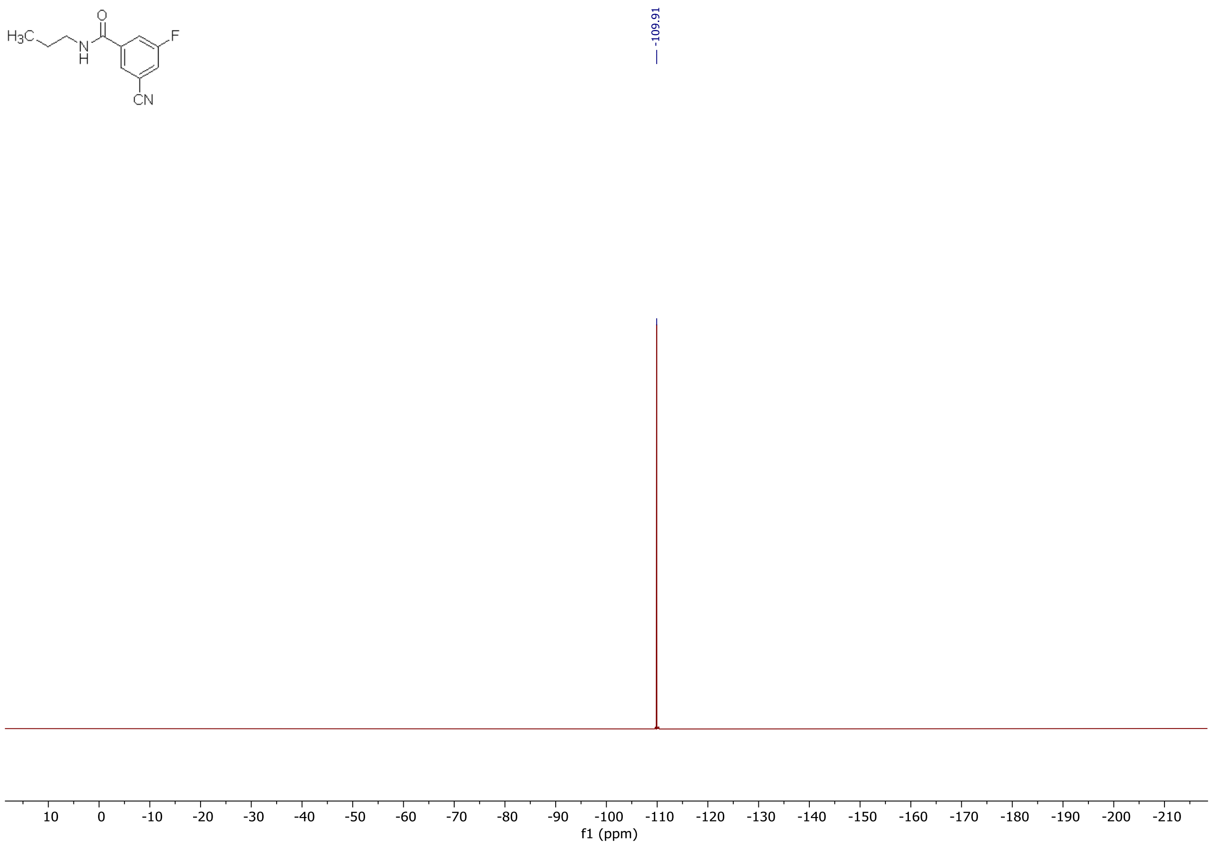

3-Fluoro-N-propyl-5-(1,2,4,5-tetrazin-3-yl)benzamide

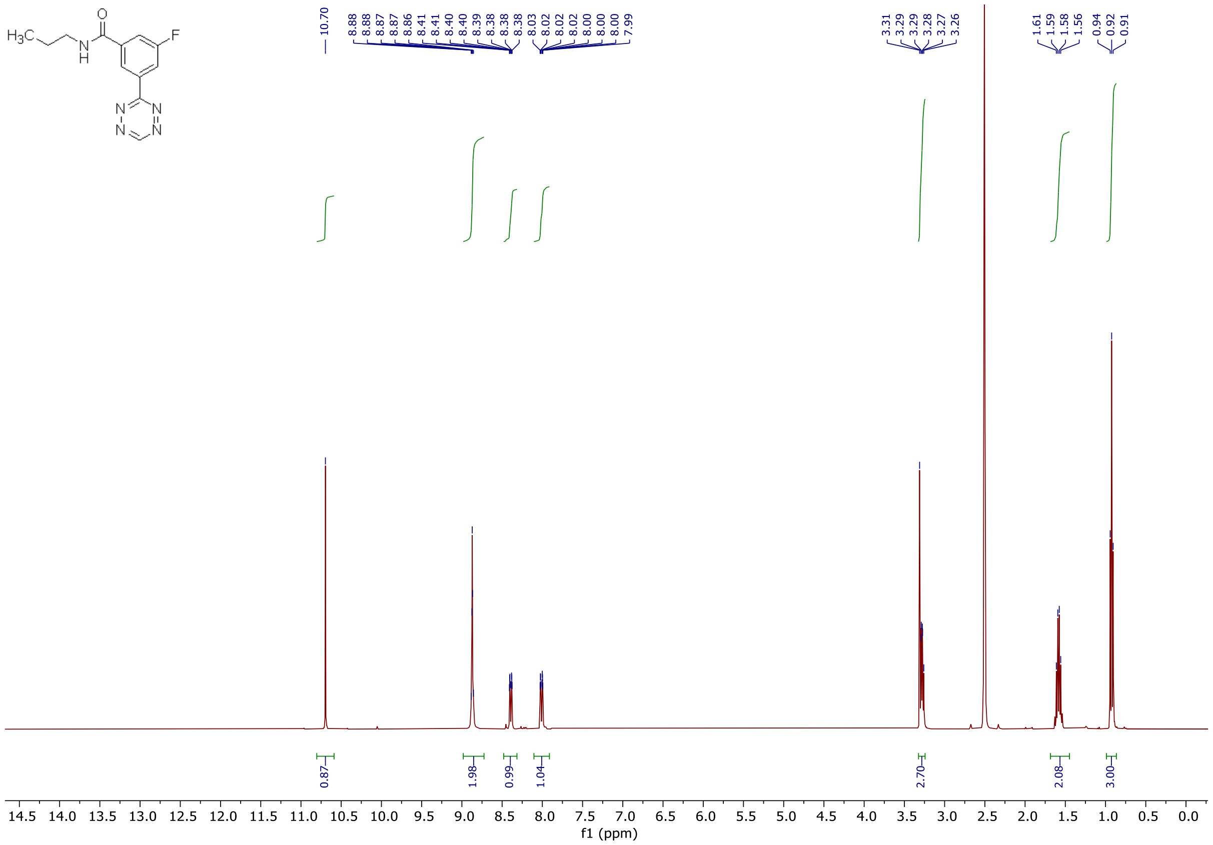

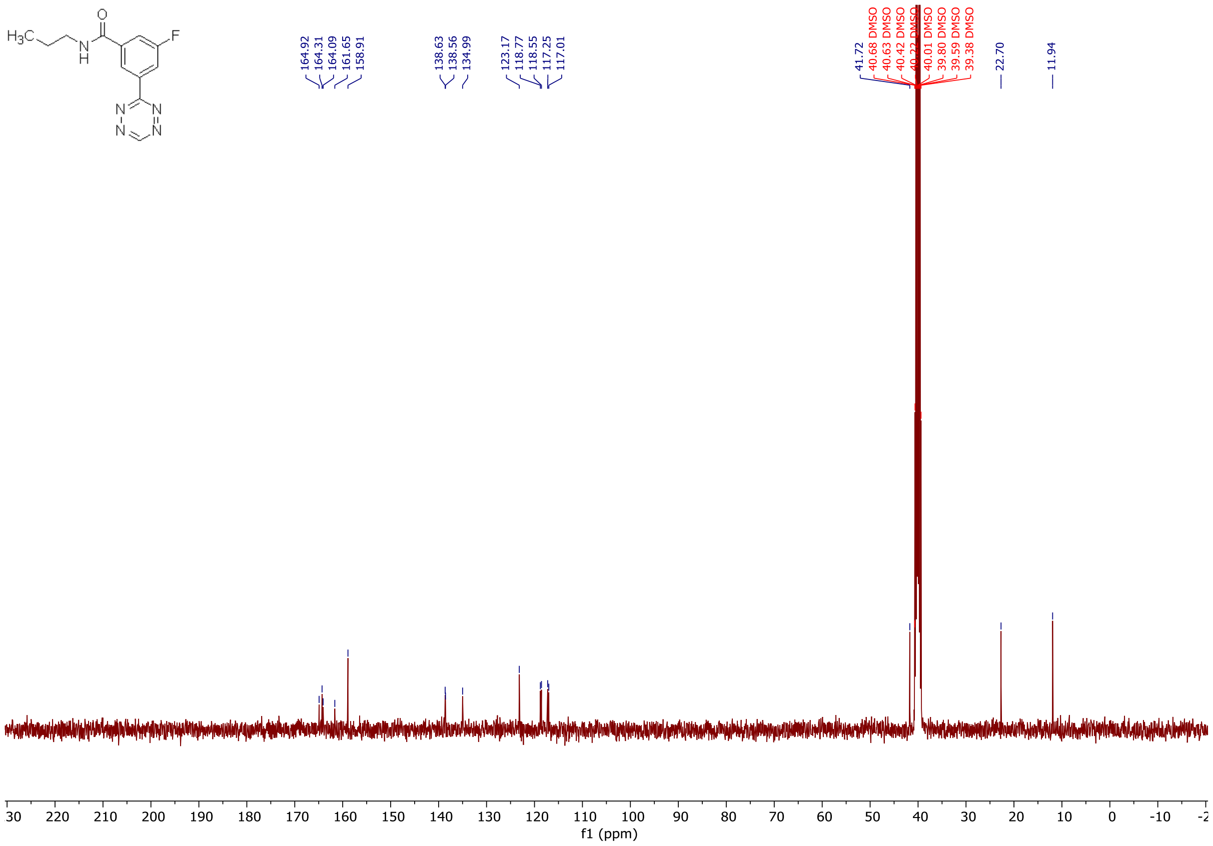
164.92
164.31
164.09
161.65
158.91
138.63
138.56
134.99
123.17
118.77
118.55
117.25
117.01
41.72
40.68 DMSO
40.63 DMSO
40.42 DMSO
40.22 DMSO
40.01 DMSO
39.80 DMSO
39.59 DMSO
39.38 DMSO
22.70
11.94
f1 (ppm)

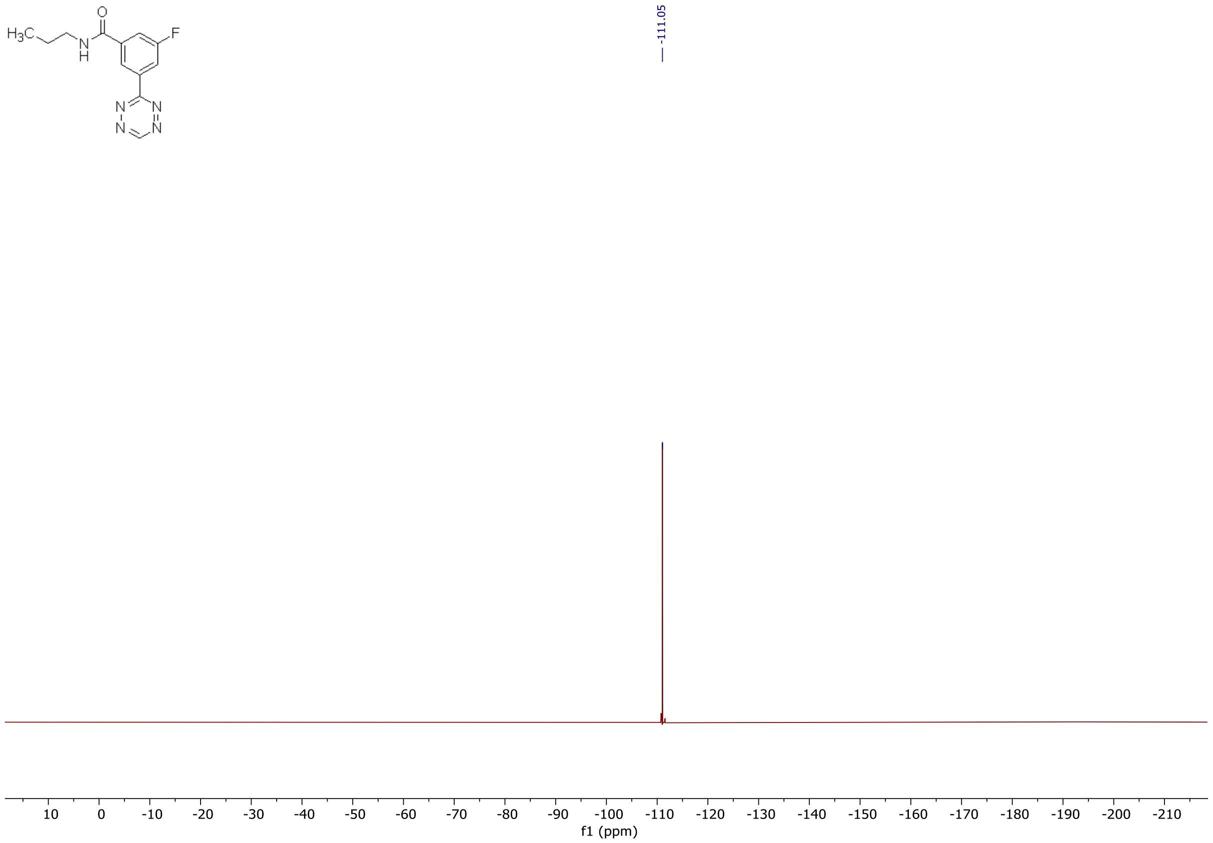
-111.05
f1 (ppm)

3-Fluoro-5-((methylamino)methyl)benzonitrile hydrochloride

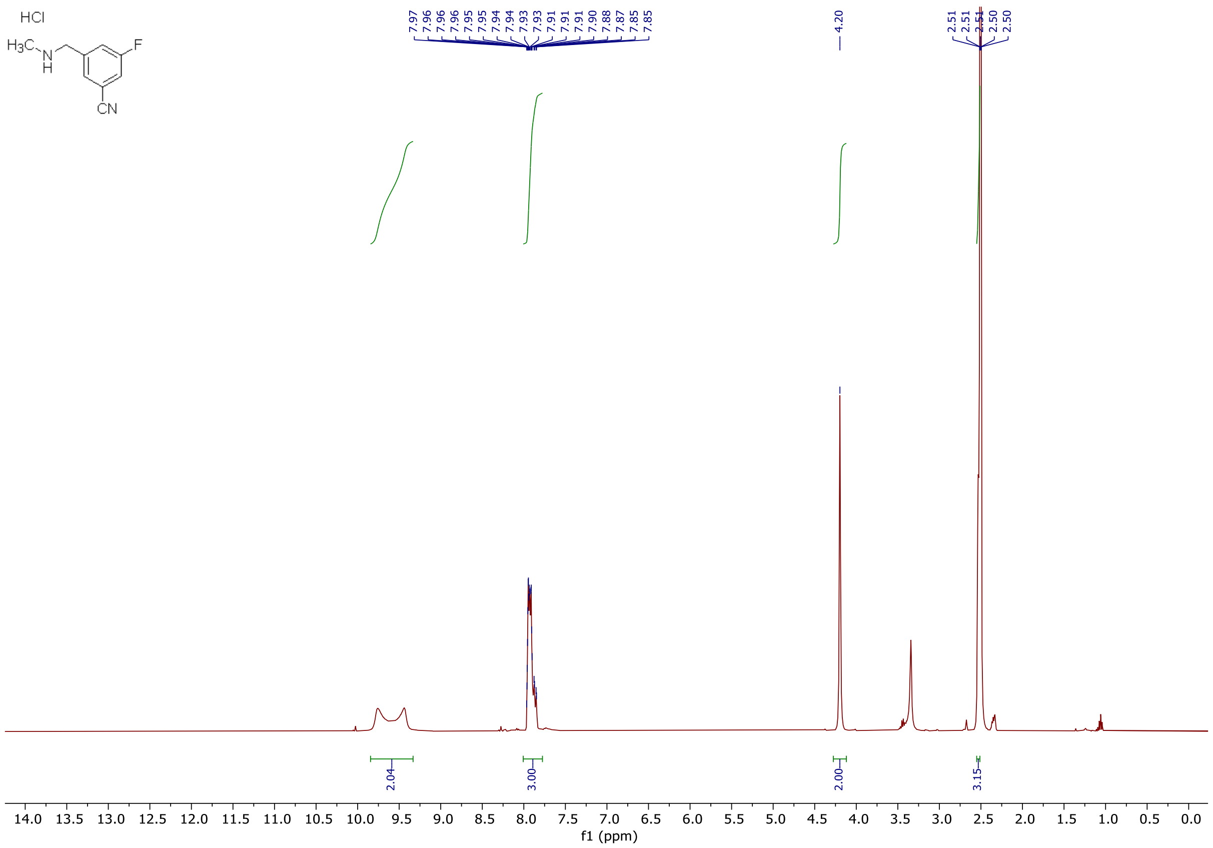

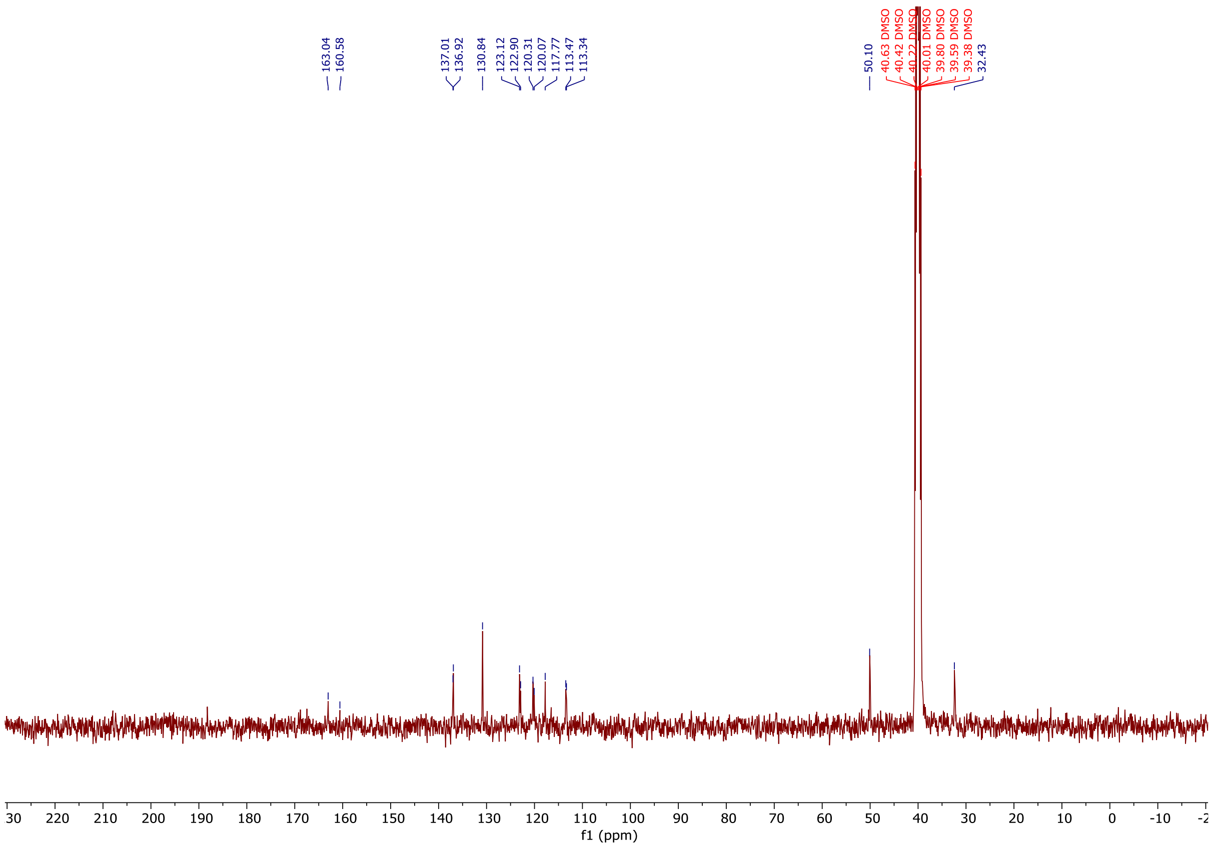

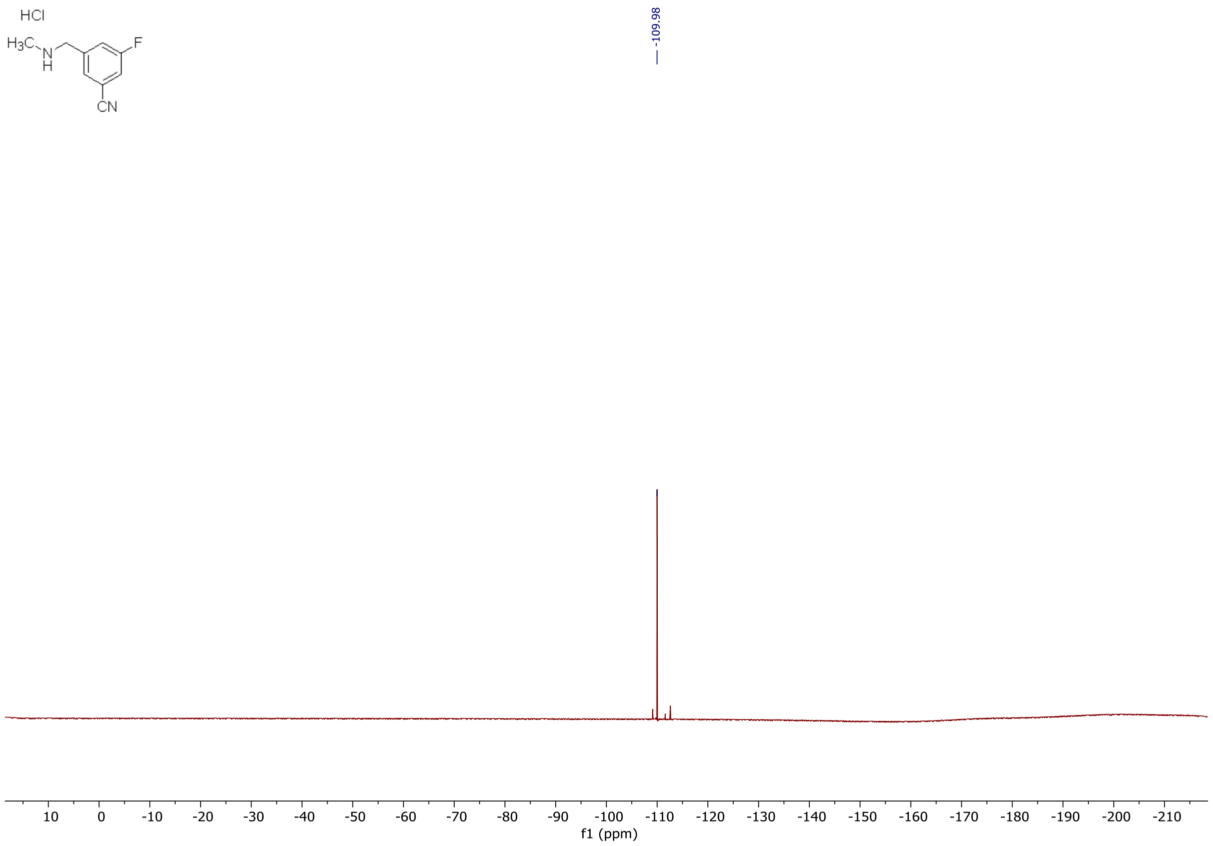

***Tert*-butyl 3-cyano-5-fluorobenzyl(methyl)carbamate**

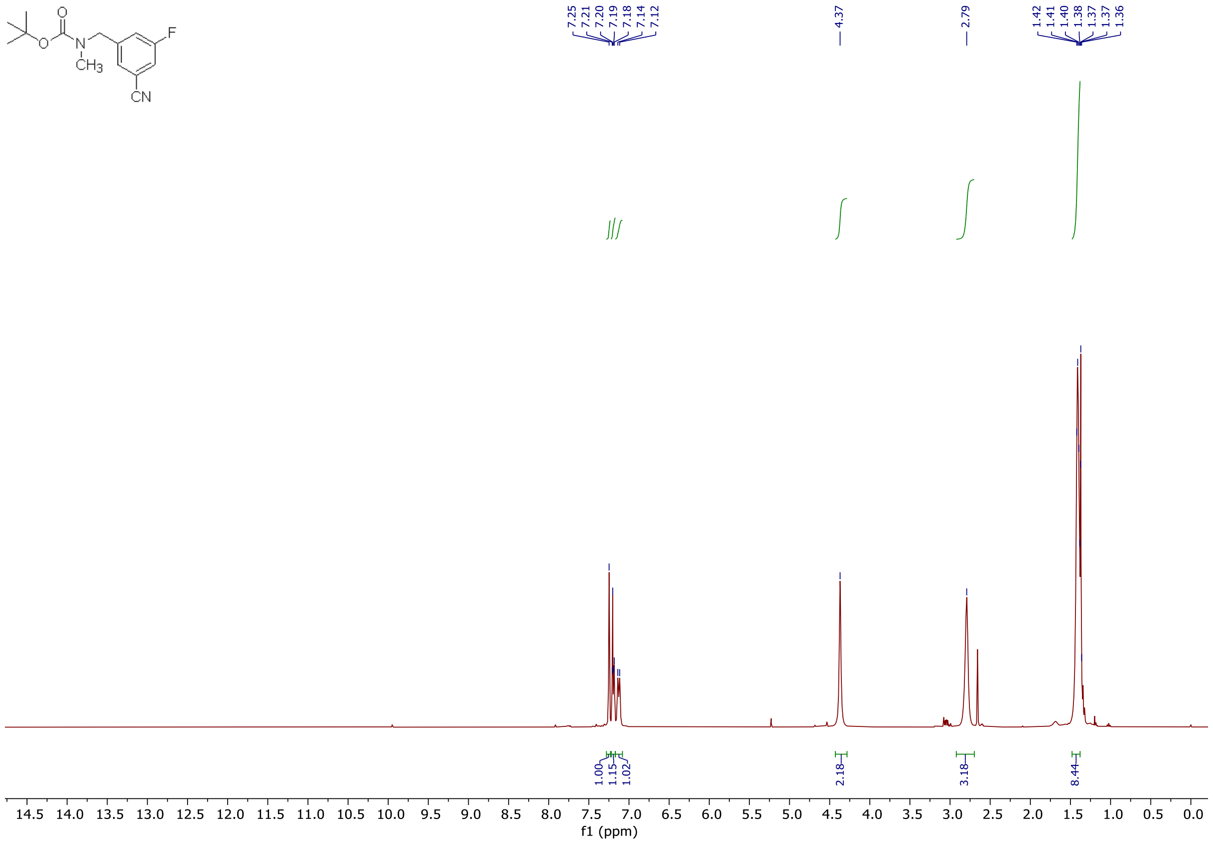

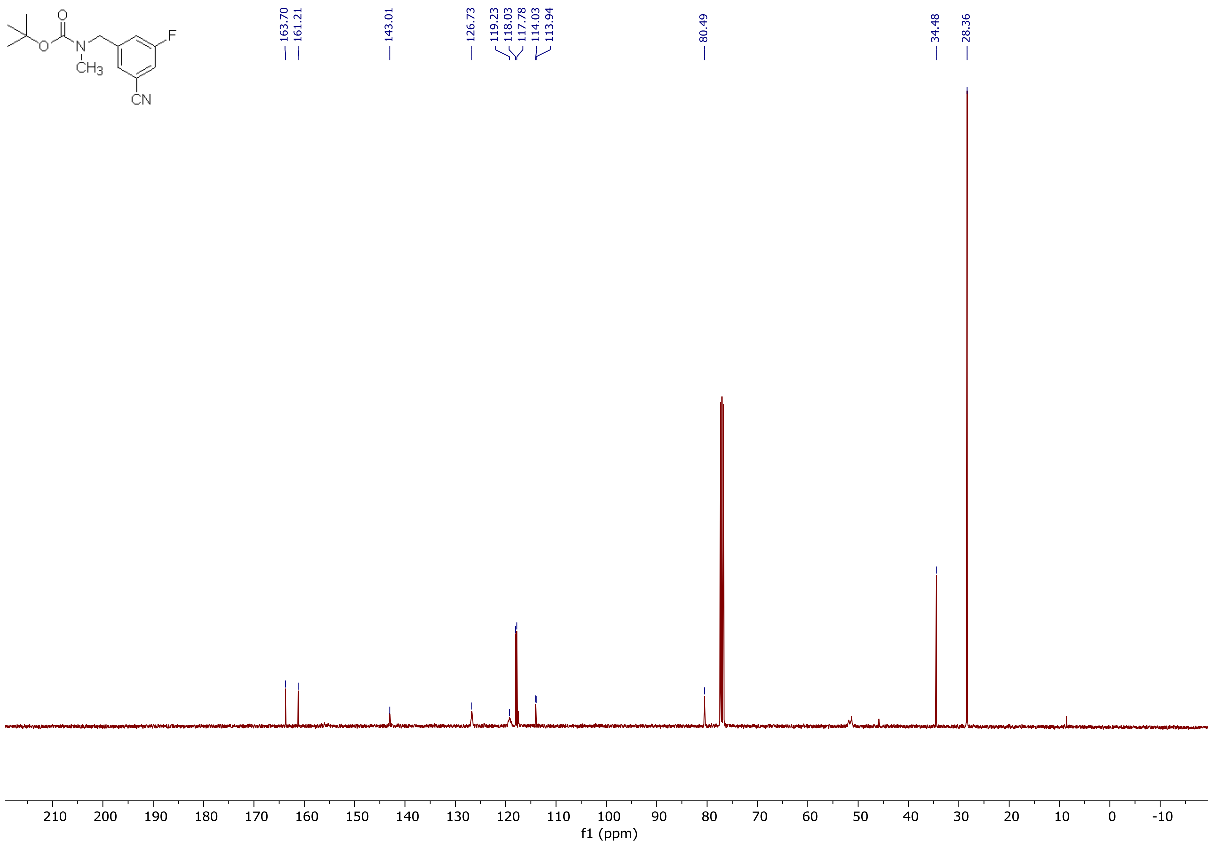
F
CN
O
N
CH3
163.70
161.21
143.01
126.73
119.23
118.03
117.78
114.03
113.94
80.49
34.48
28.36
210 200 190 180 170 160 150 140 130 120 110 100 90 80 70 60 50 40 30 20 10 0 -10
f1 (ppm)

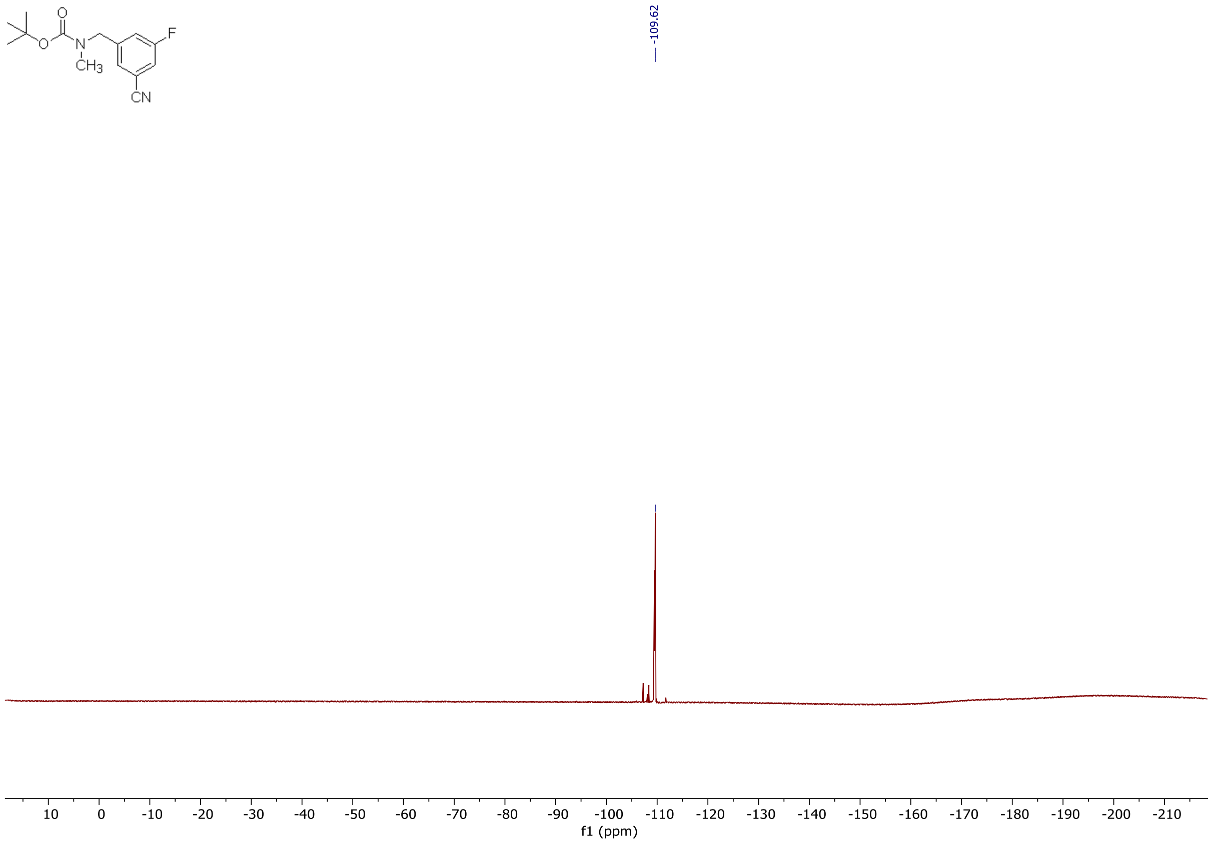
F
CN
O
N
CH3
-109.62
10 0 -10 -20 -30 -40 -50 -60 -70 -80 -90 -100 -110 -120 -130 -140 -150 -160 -170 -180 -190 -200 -210
f1 (ppm)

Tert-butyl 3-fluoro-5-(1,2,4,5-tetrazin-3-yl)benzyl(methyl)carbamate

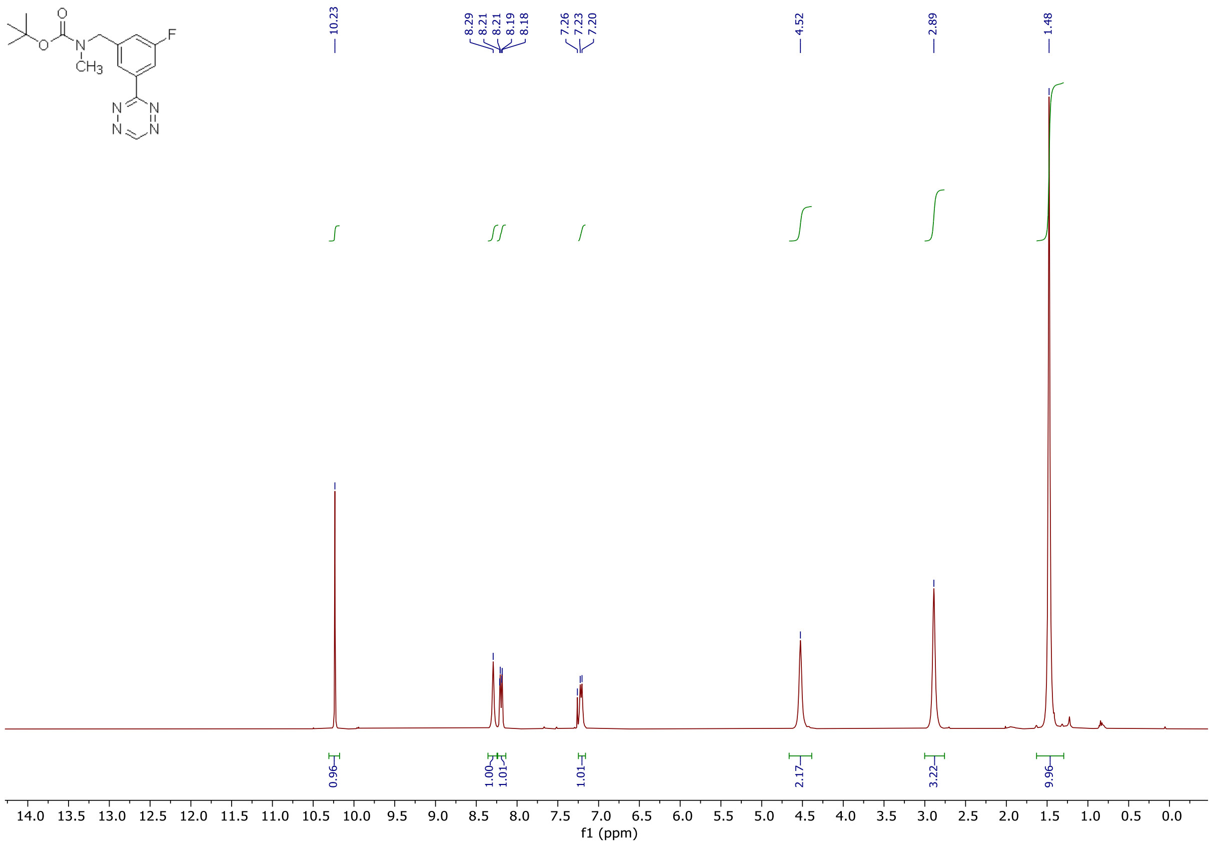

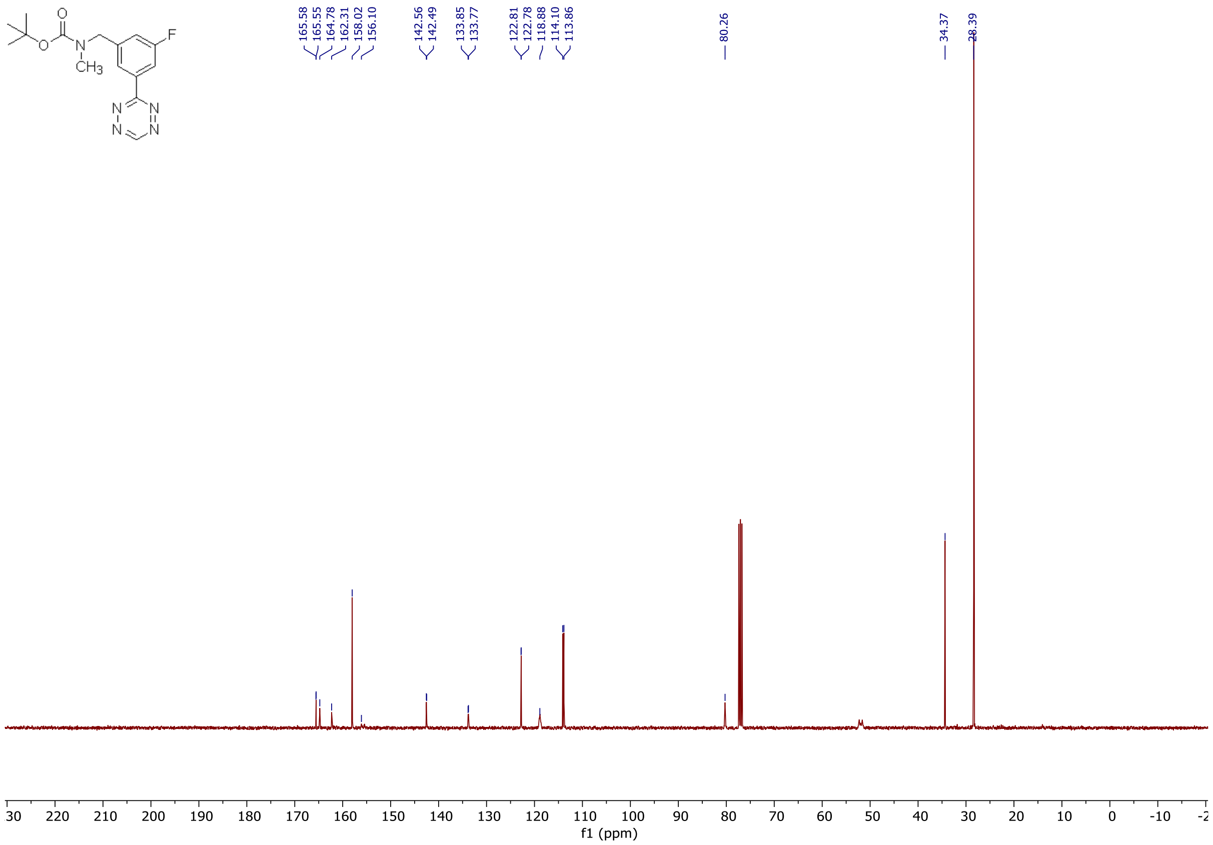

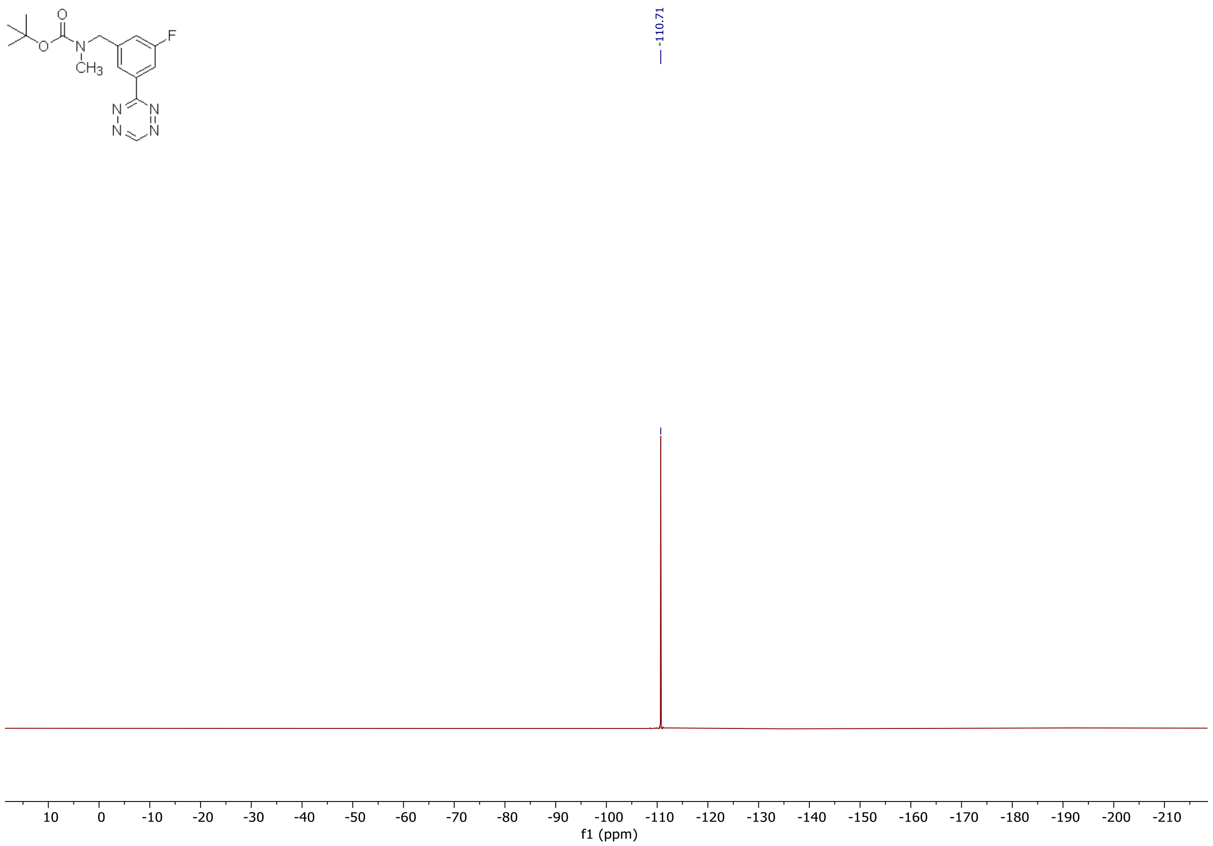

1-(3-Fluoro-5-(1,2,4,5-tetrazin-3-yl)phenyl)-N-methylmethanamine hydrochloride

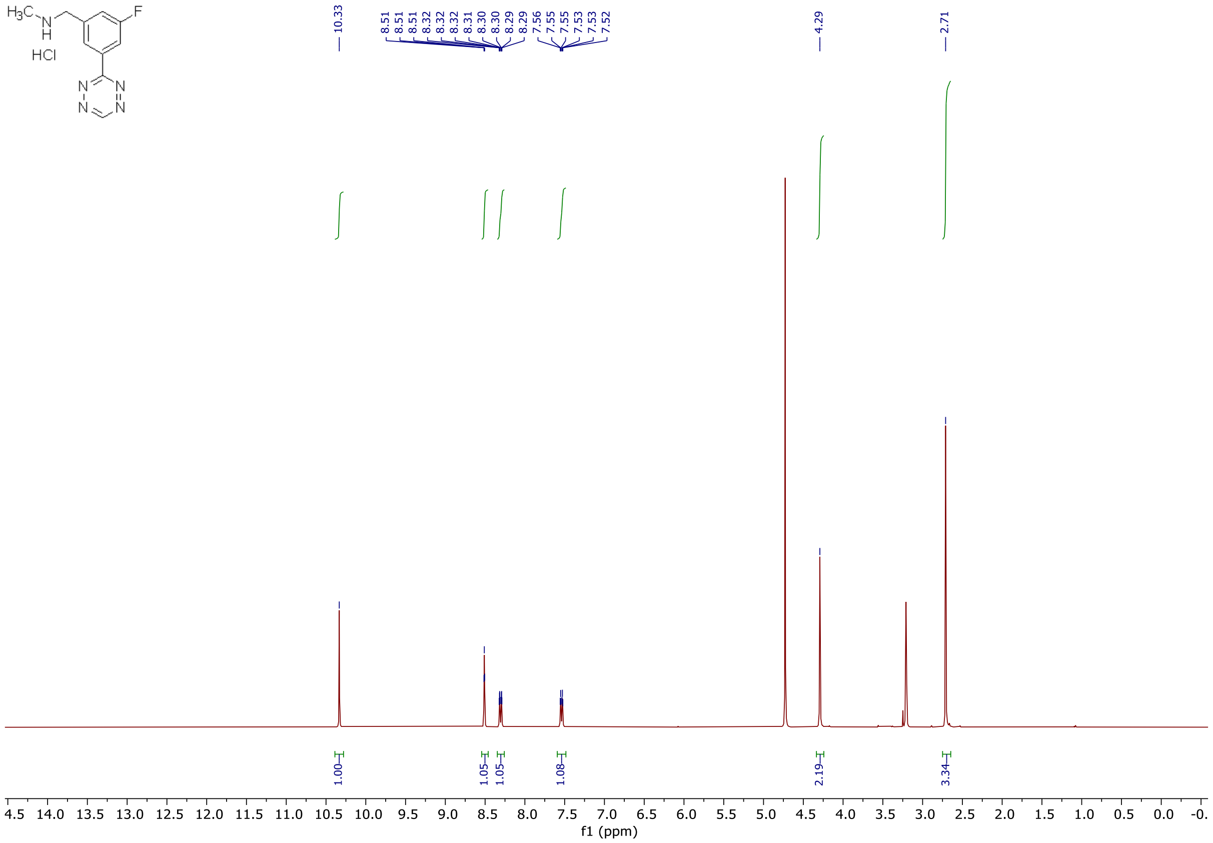

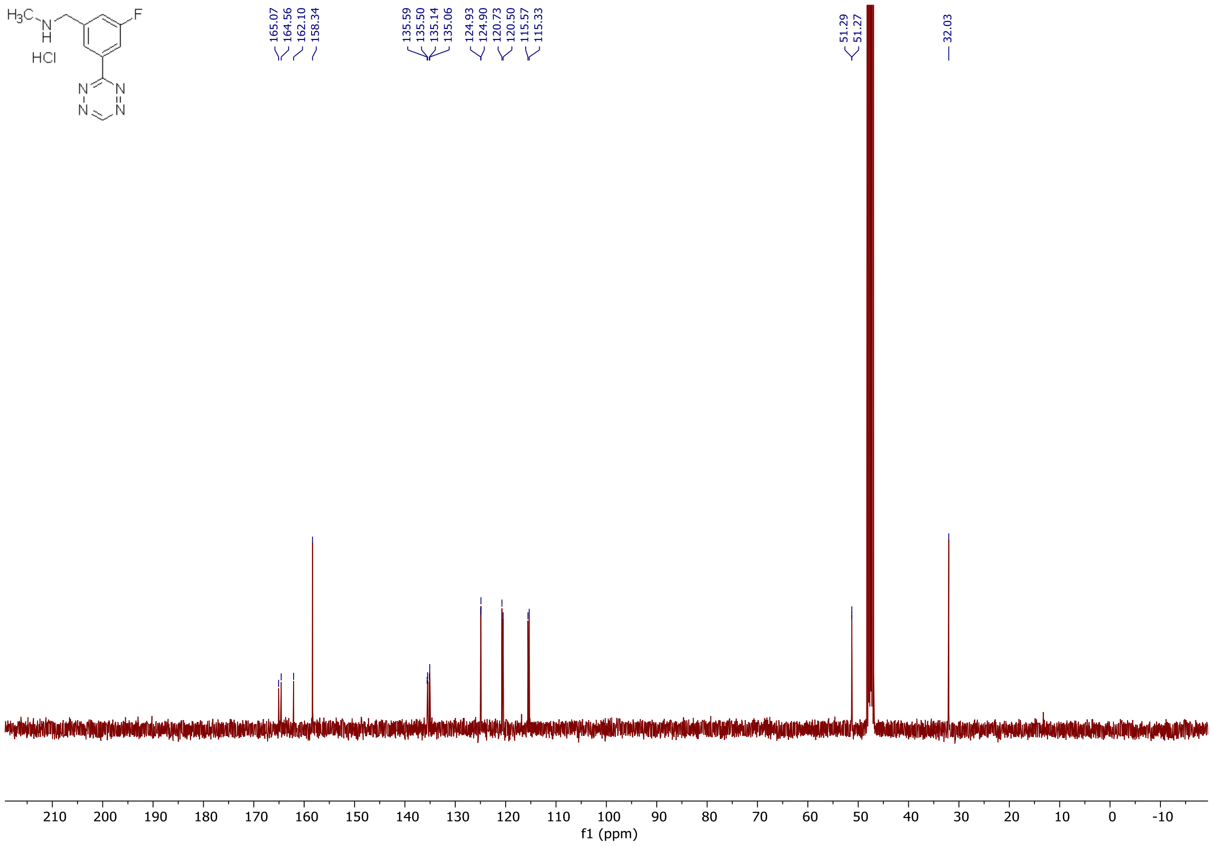
H3C
N
H
HCl
F
N
N
N
N
165.07
164.56
162.10
158.34
135.59
135.50
135.14
135.06
124.93
124.90
120.73
120.50
115.57
115.33
51.29
51.27
32.03
210 200 190 180 170 160 150 140 130 120 110 100 90 80 70 60 50 40 30 20 10 0 -10
f1 (ppm)

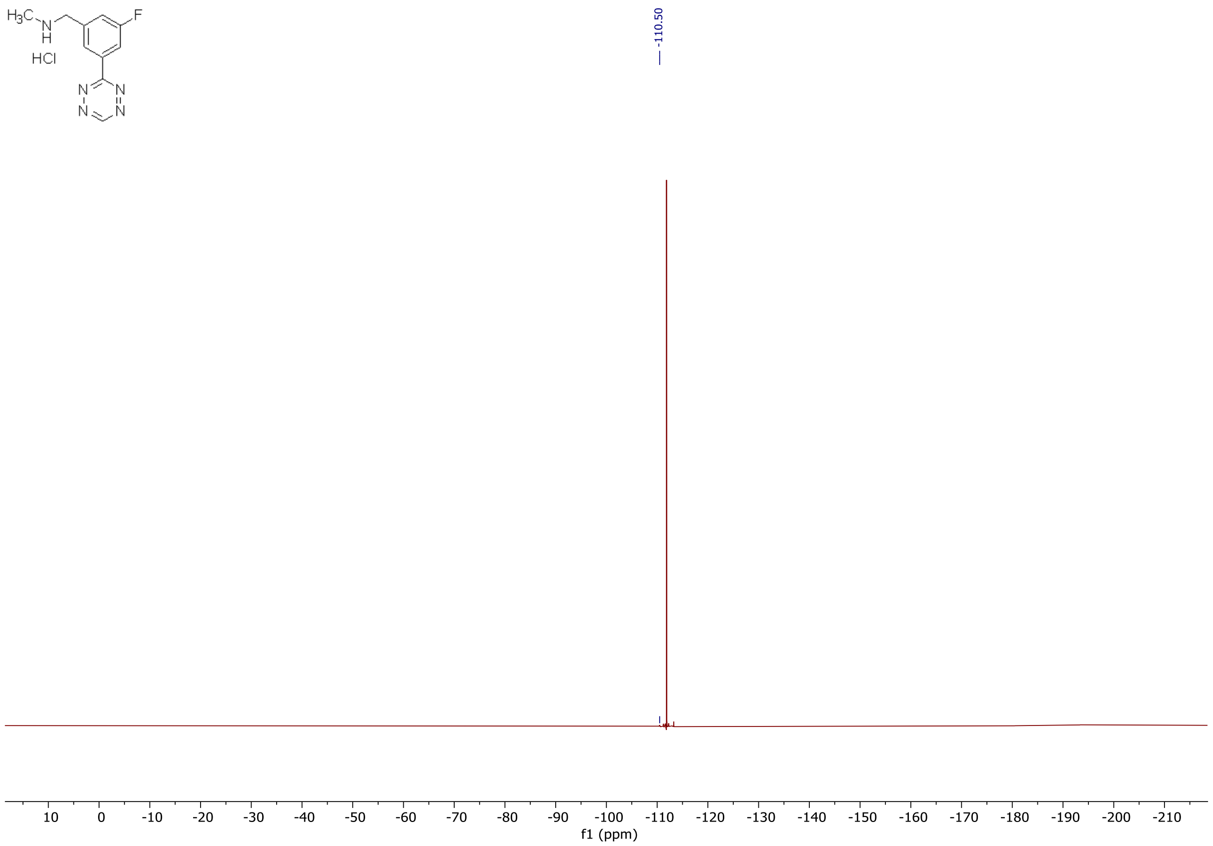
H3C
N
H
HCl
F
N
N
N
N
-110.50
10 0 -10 -20 -30 -40 -50 -60 -70 -80 -90 -100 -110 -120 -130 -140 -150 -160 -170 -180 -190 -200 -210
f1 (ppm)

3-((Ethylamino)methyl)-5-fluorobenzonitrile hydrochloride

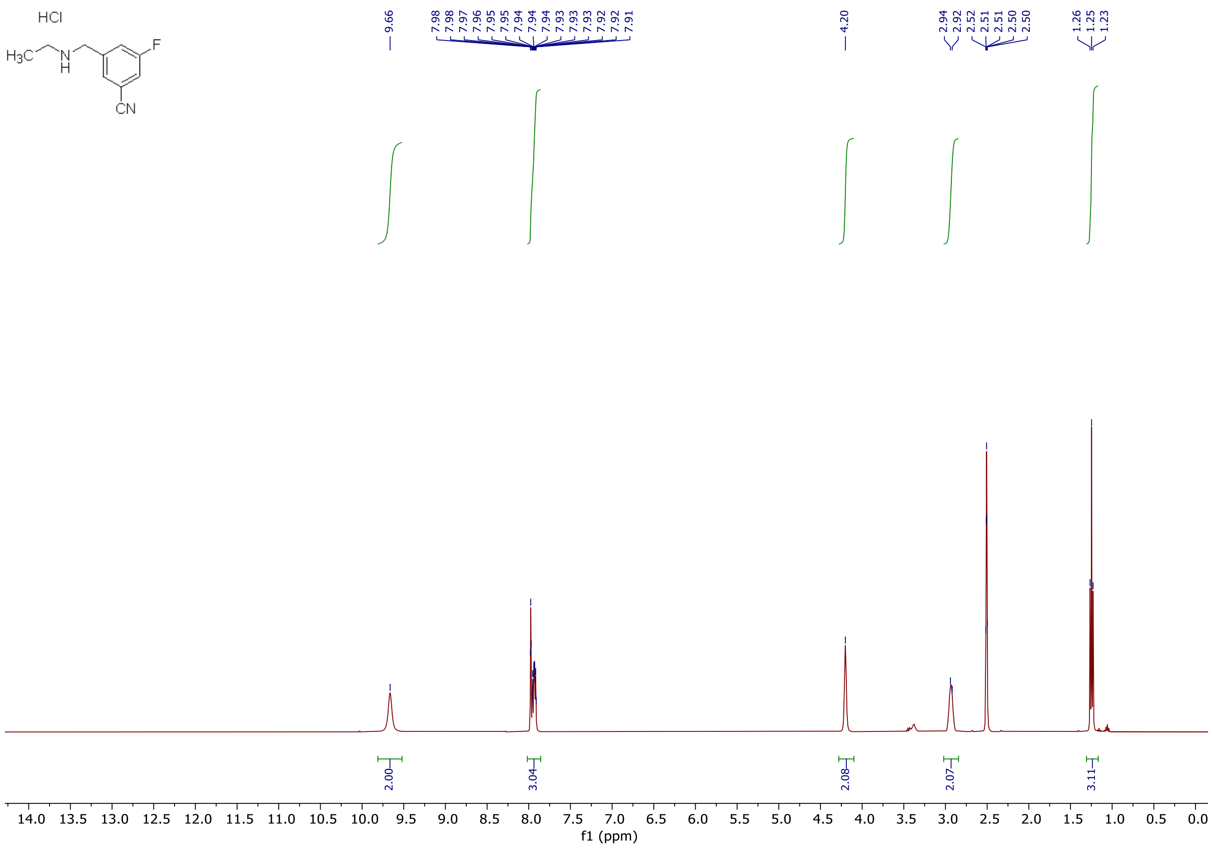

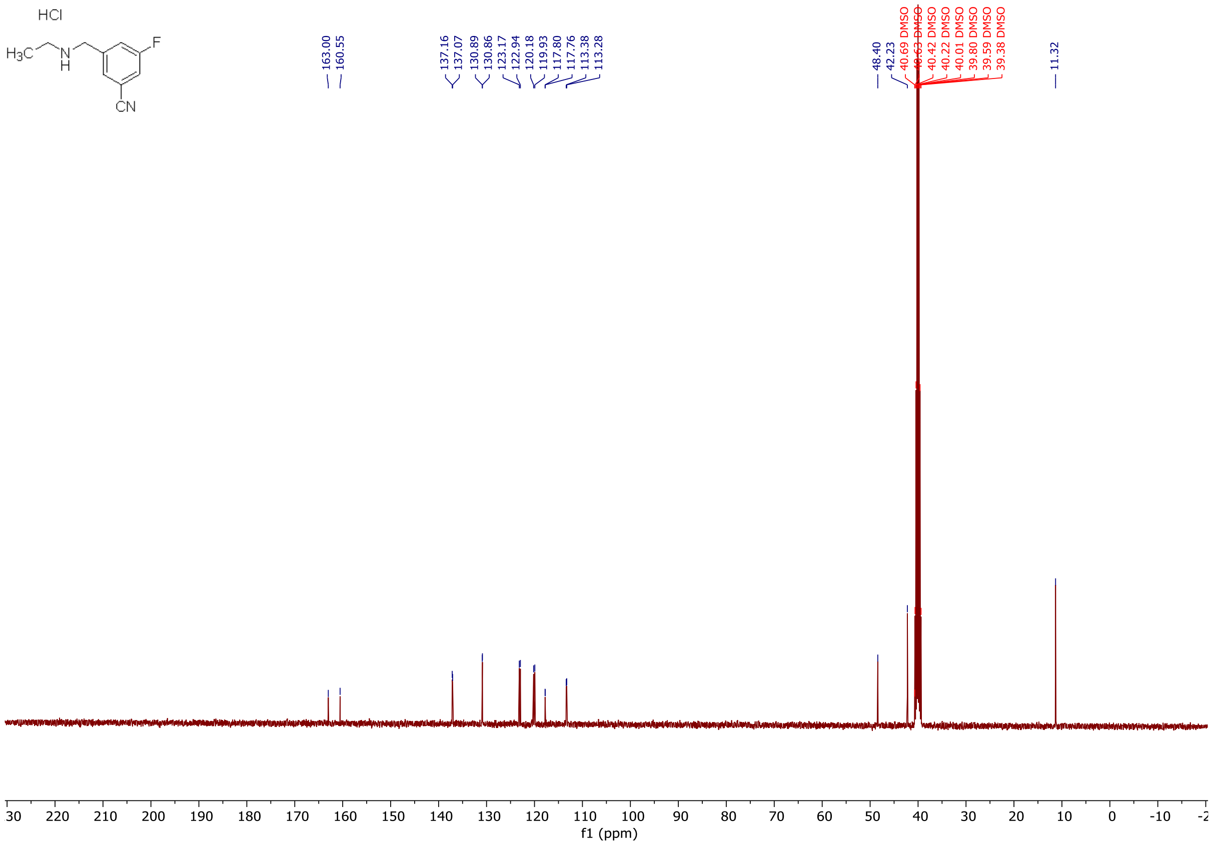

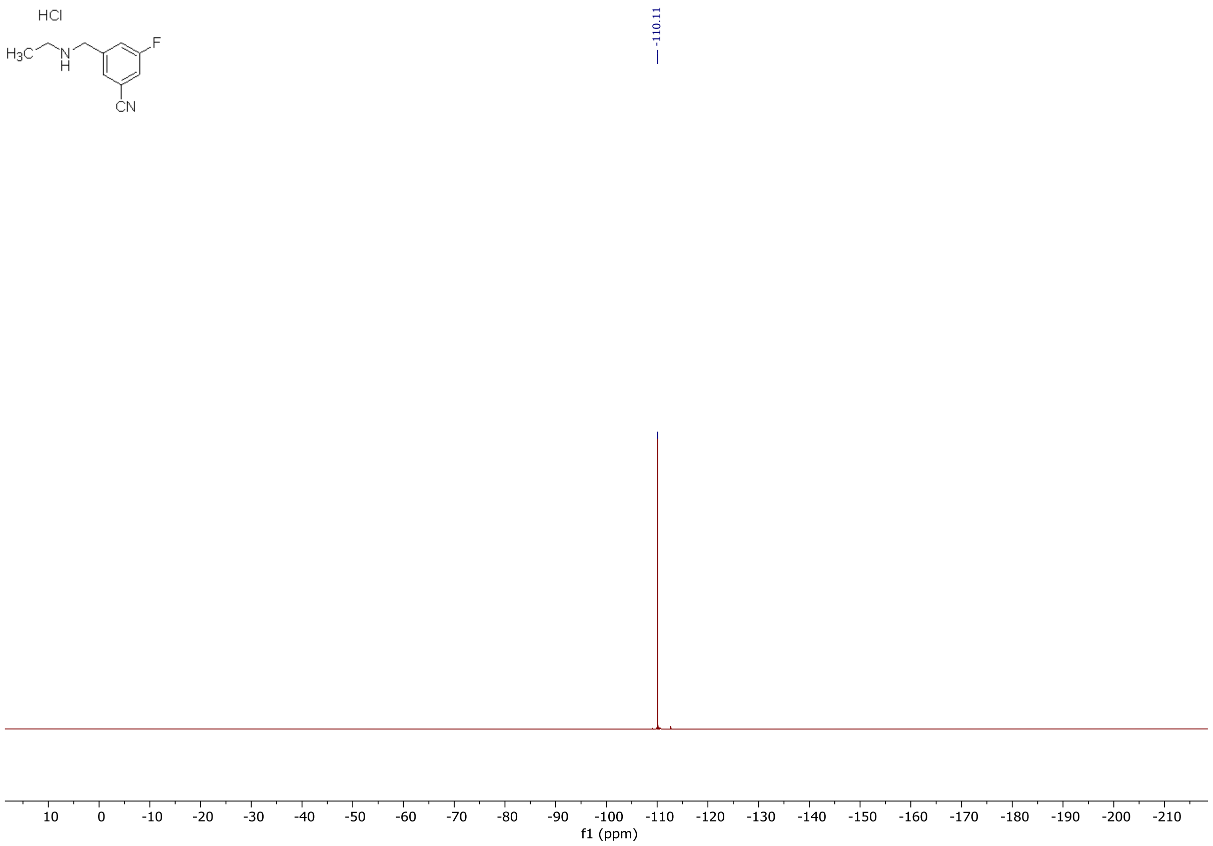

Tert-butyl 3-cyano-5-fluorobenzyl(ethyl)carbamate

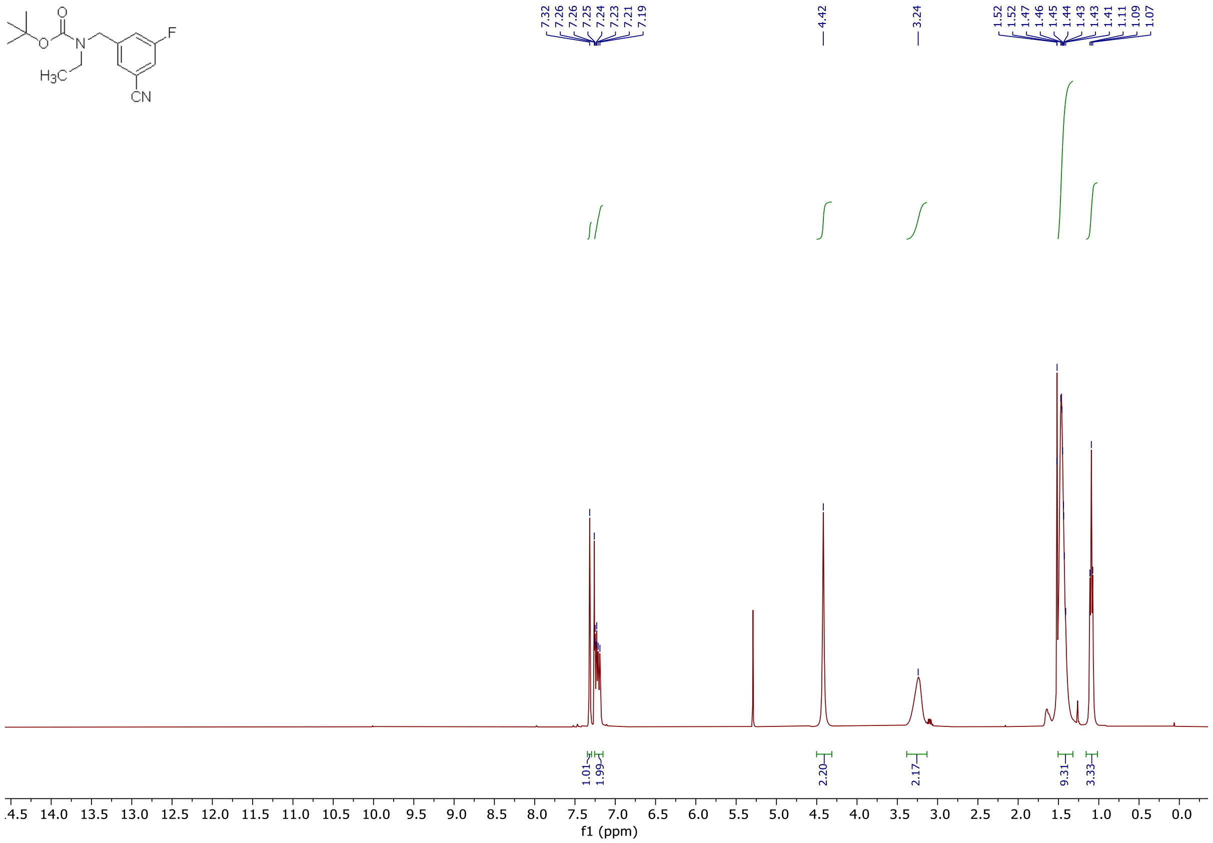

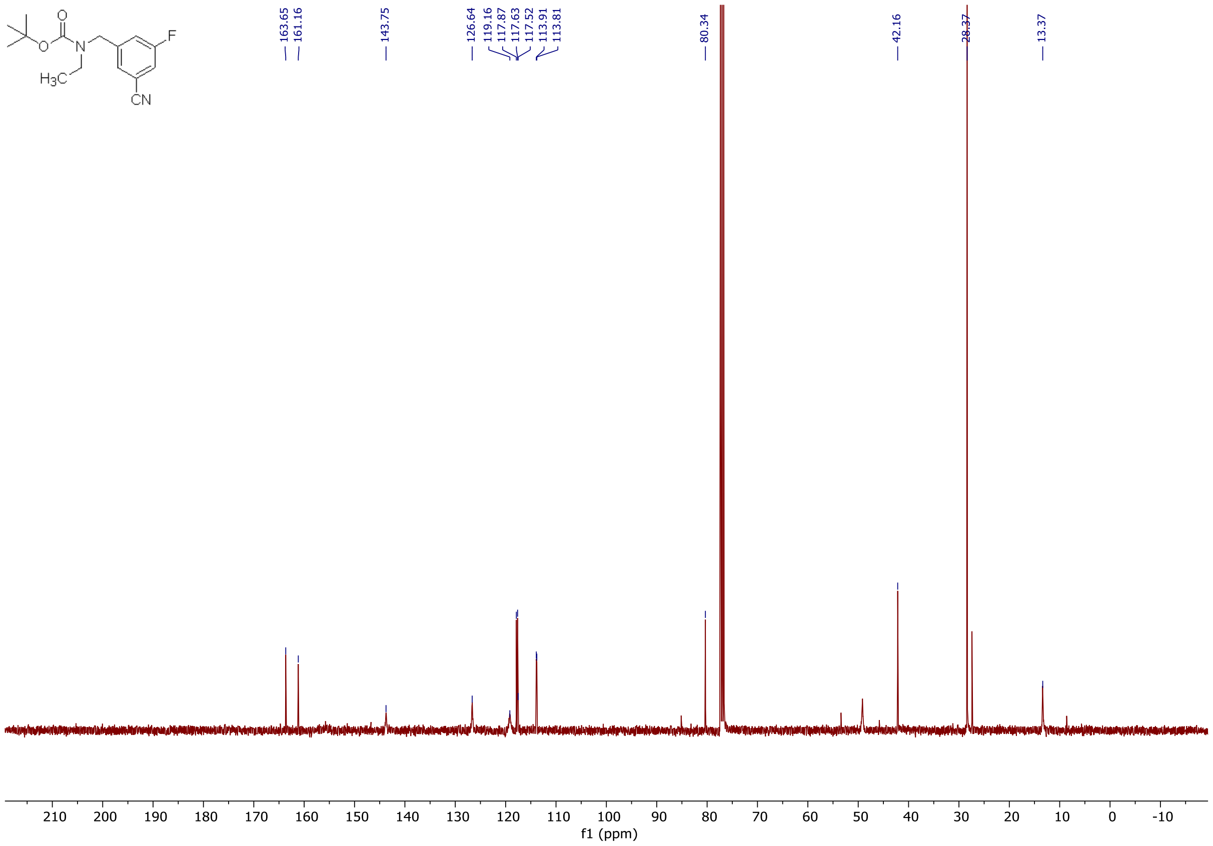

F
N
O
O
H3C
CN
163.65
161.16
143.75
126.64
119.16
117.87
117.63
117.52
113.91
113.81
80.34
42.16
28.37
13.37
f1 (ppm)

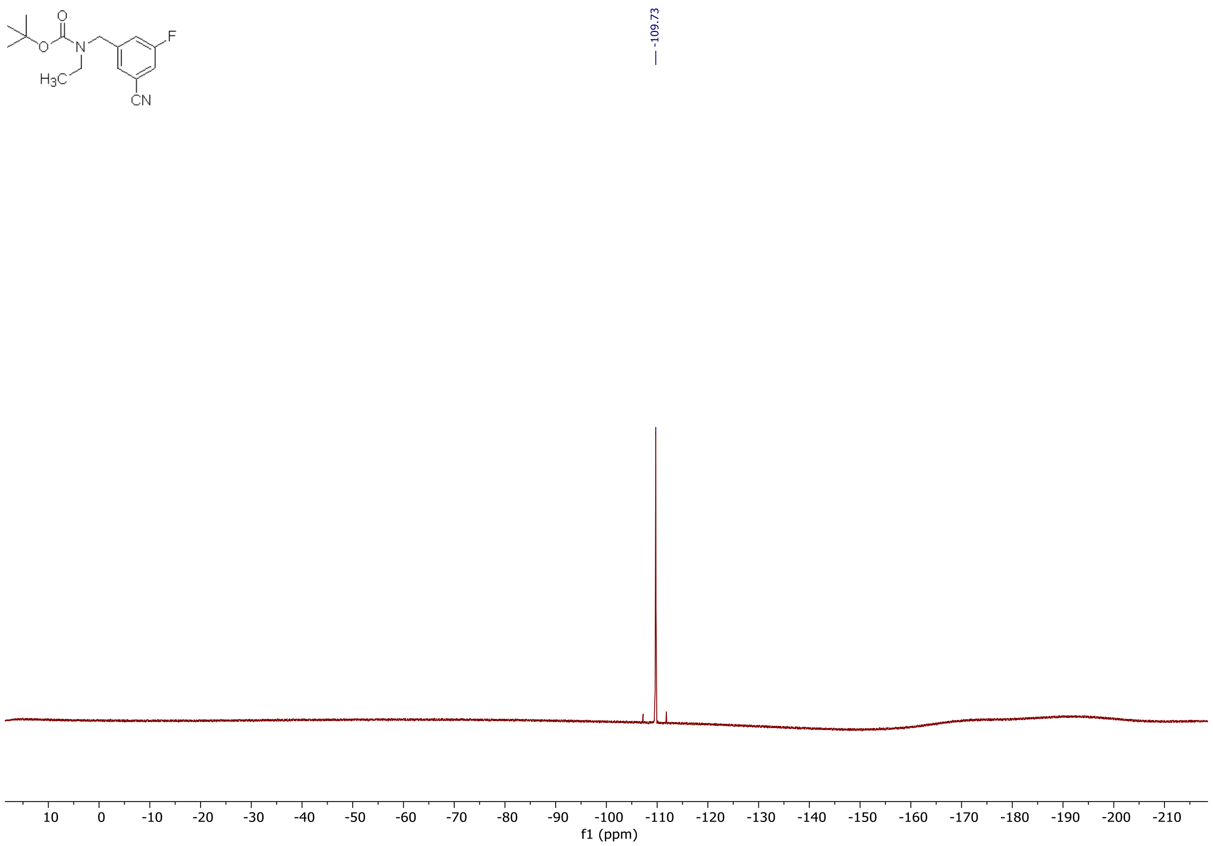

F
N
O
O
H3C
CN
-109.73
f1 (ppm)

Tert-butyl 3-fluoro-5-(1,2,4,5-tetrazin-3-yl)benzyl(ethyl)carbamate

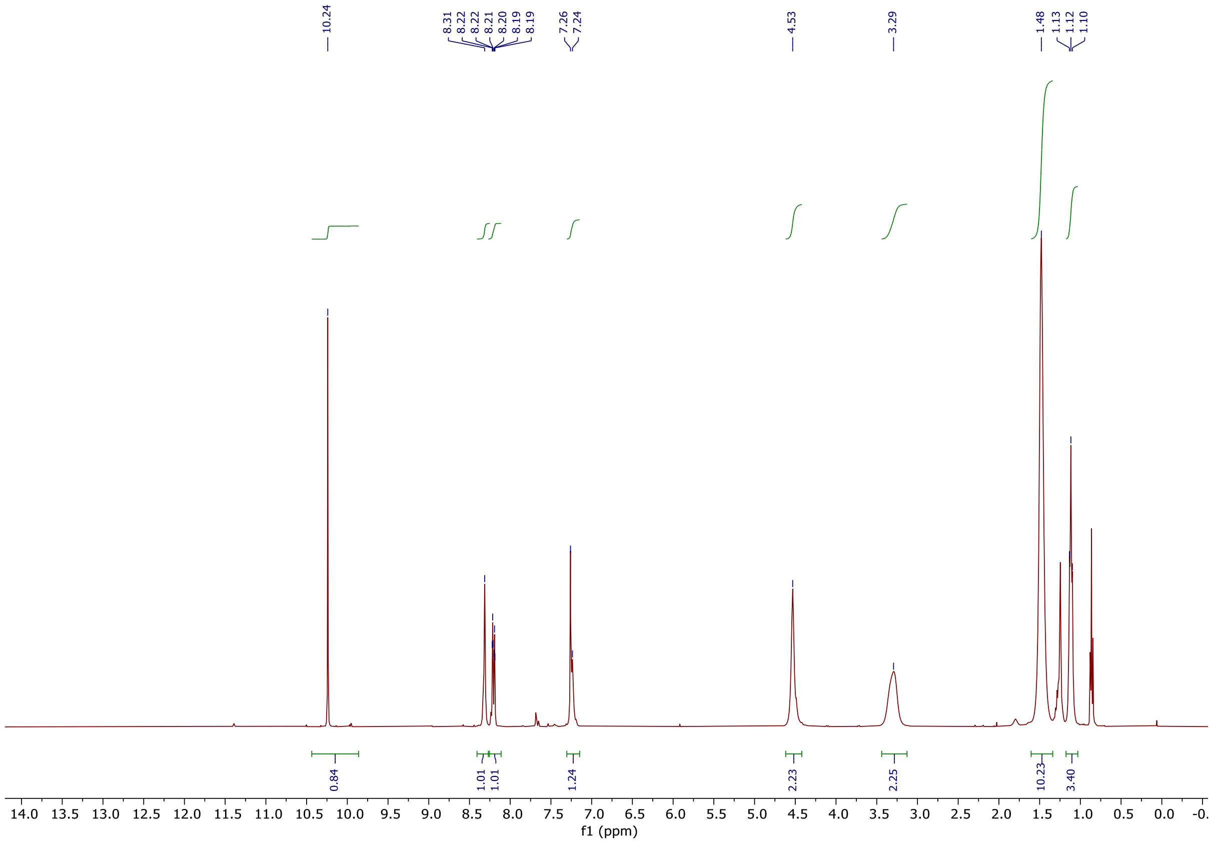

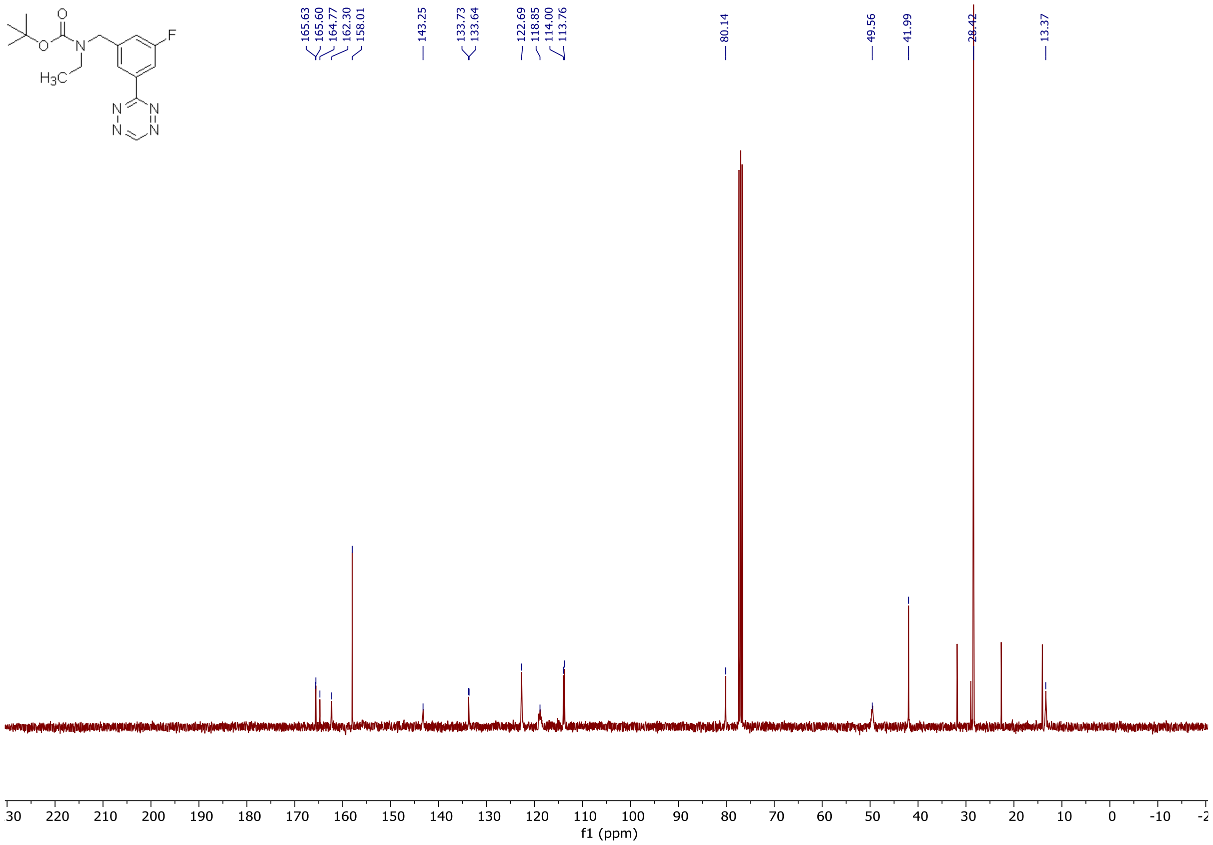

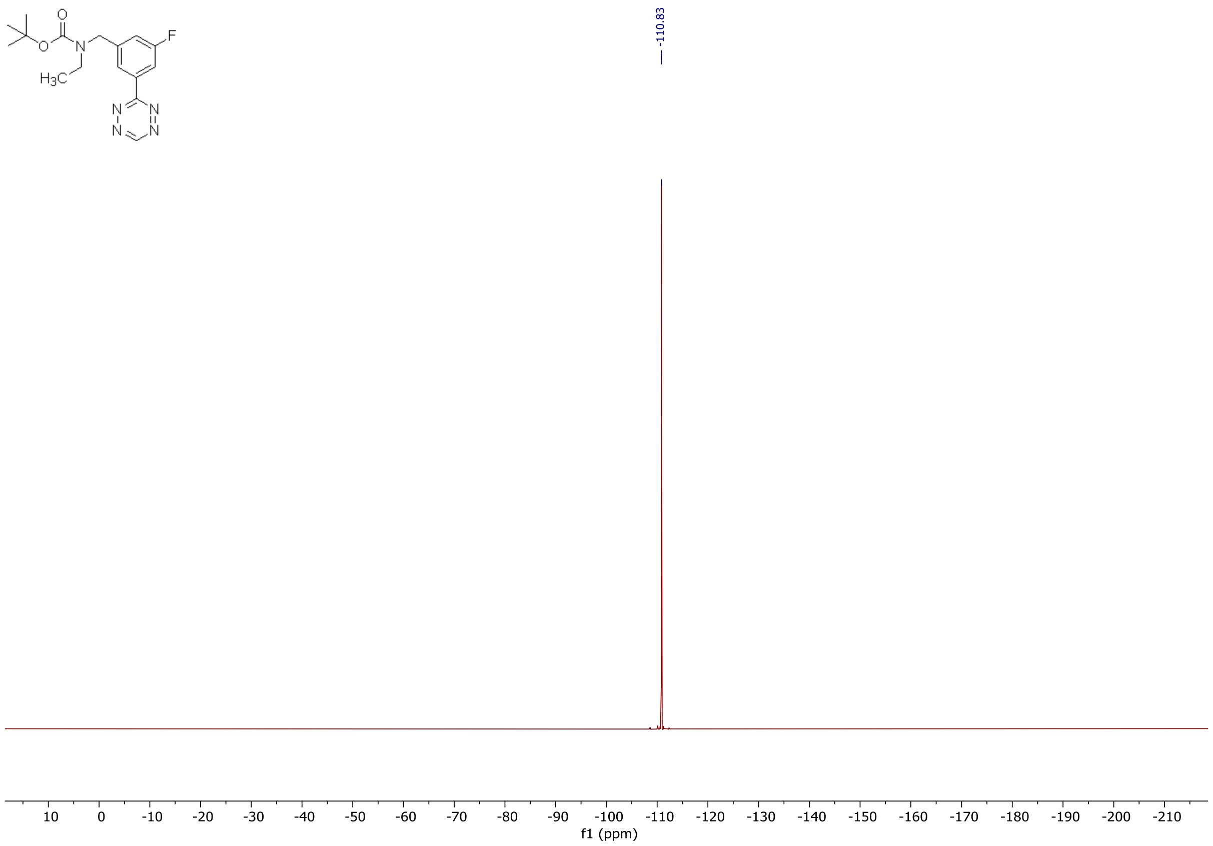

N-(3-fluoro-5-(1,2,4,5-tetrazin-3-yl)benzyl)ethanamine hydrochloride

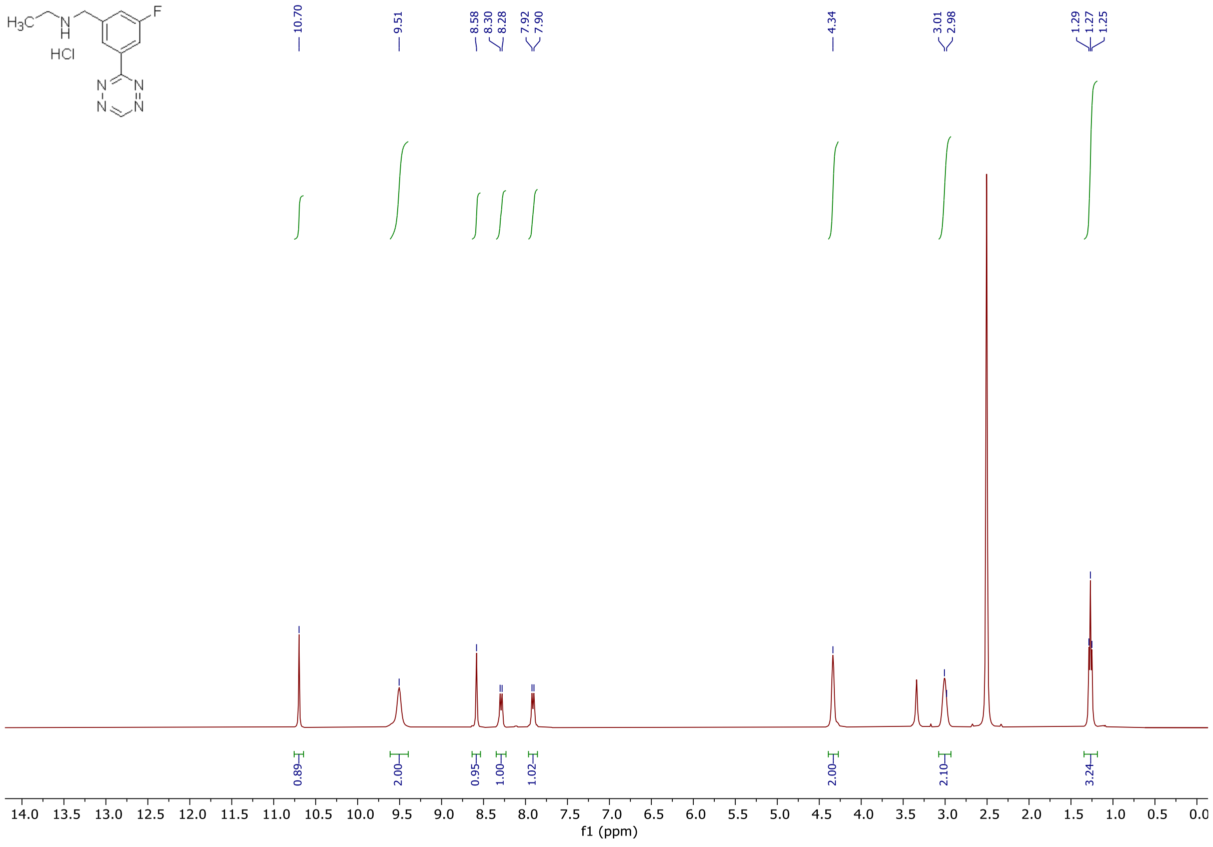

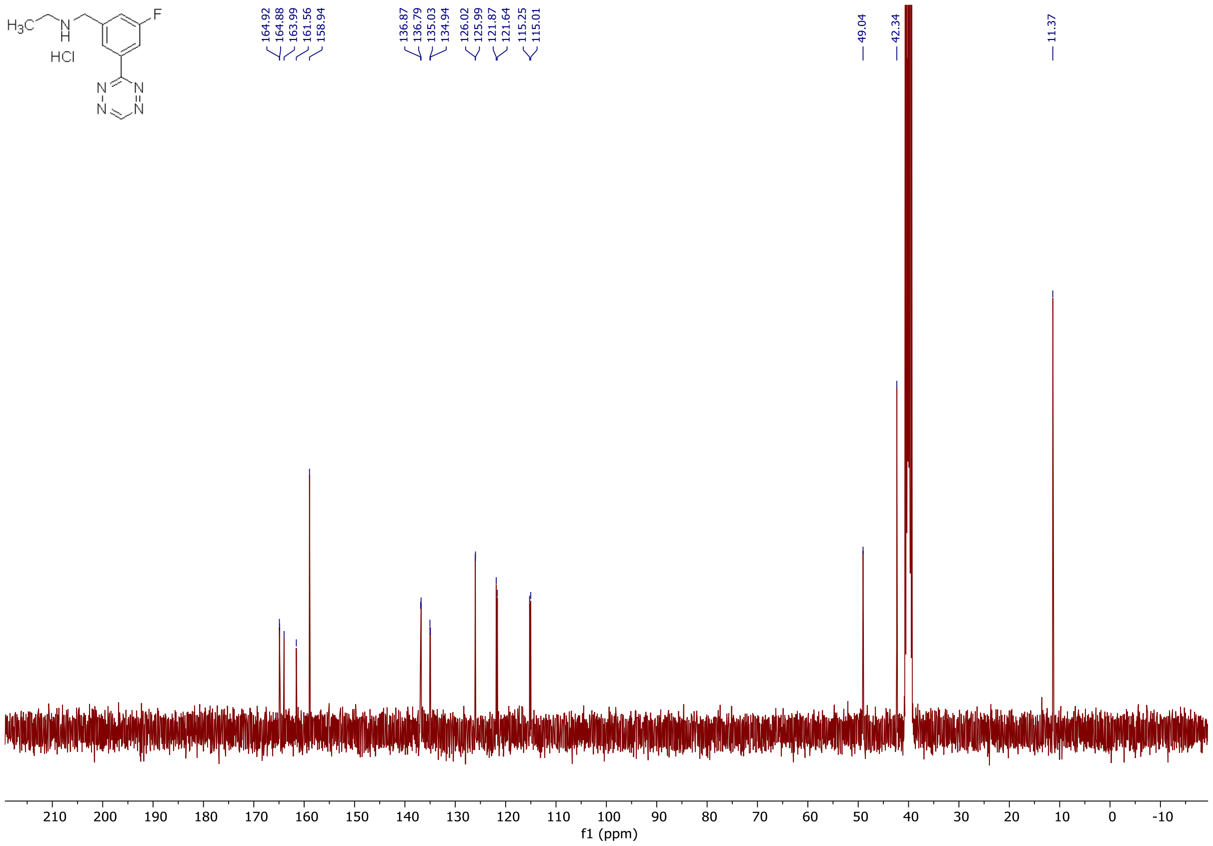

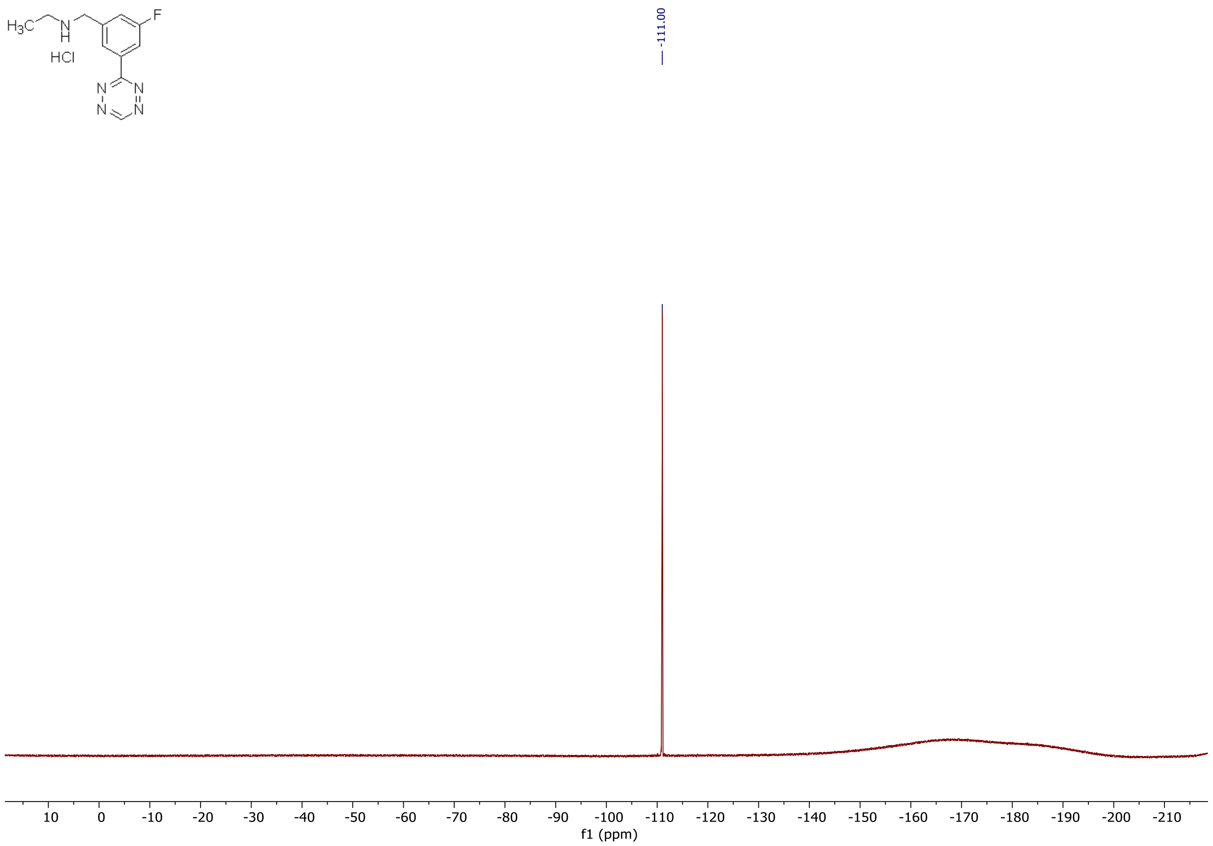

3-((Propylamino)methyl)-5-fluorobenzonitrile hydrochloride

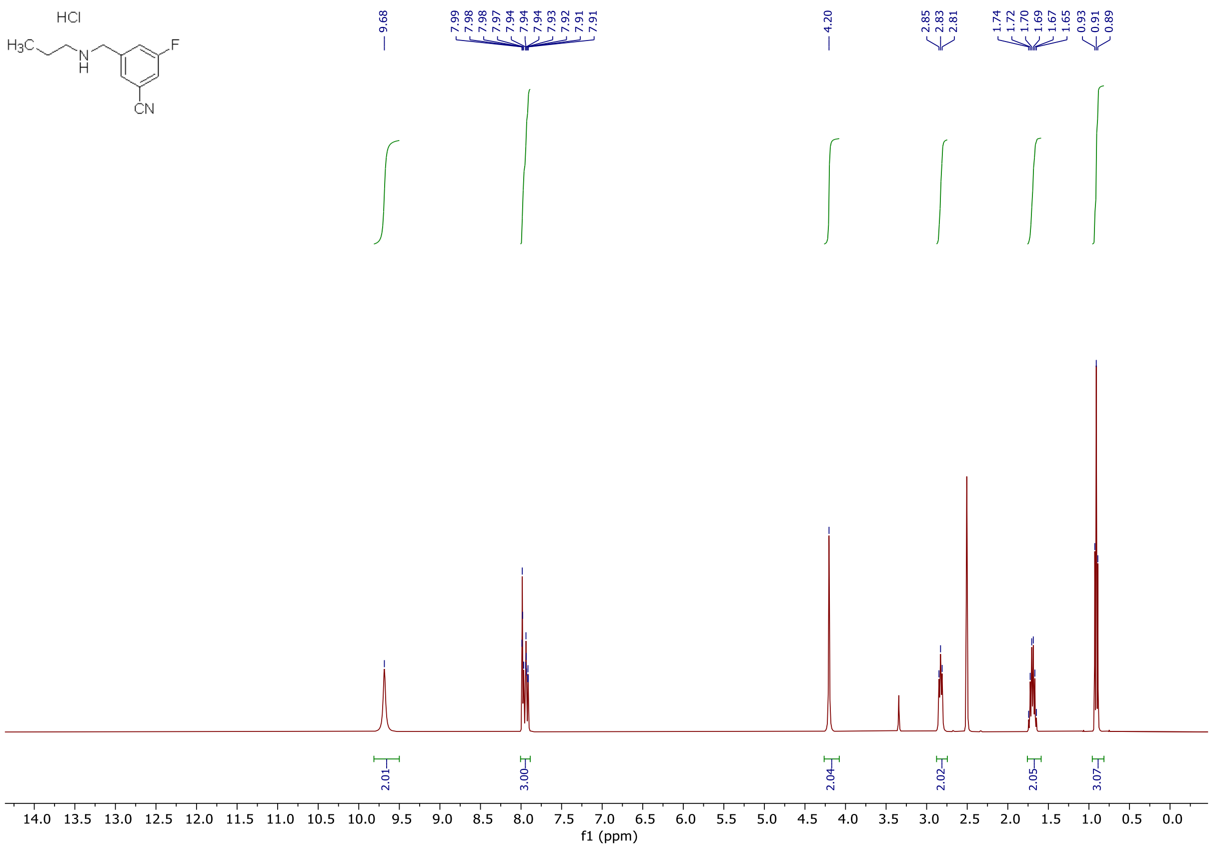

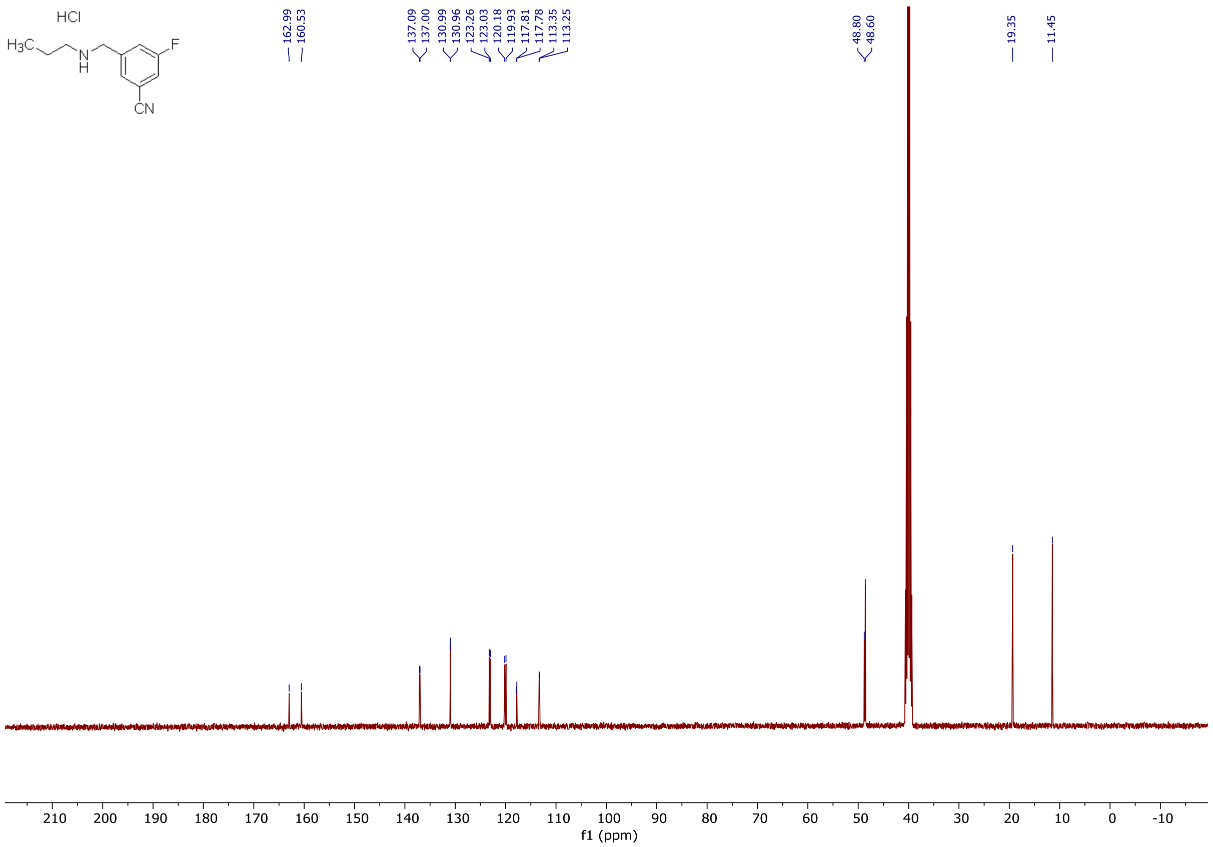

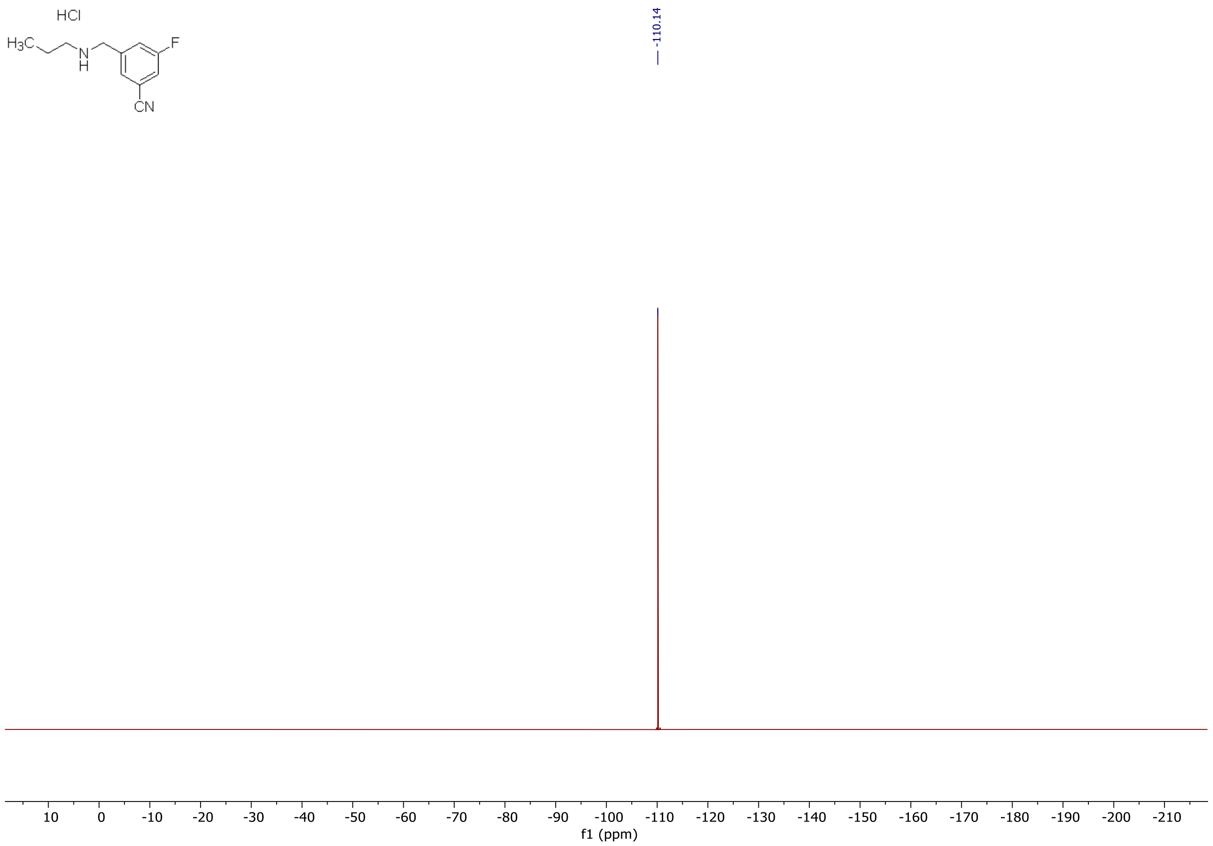

Tert-butyl 3-cyano-5-fluorobenzyl(propyl)carbamate

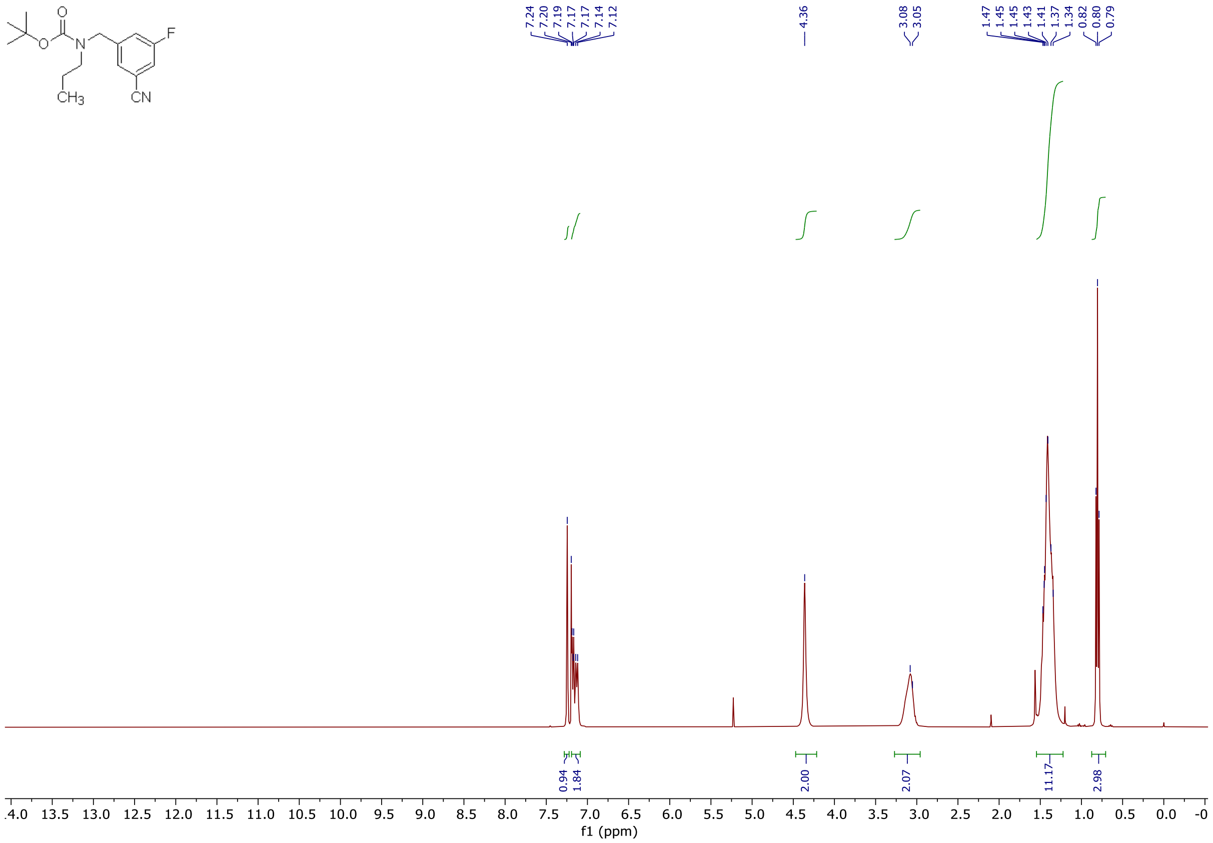

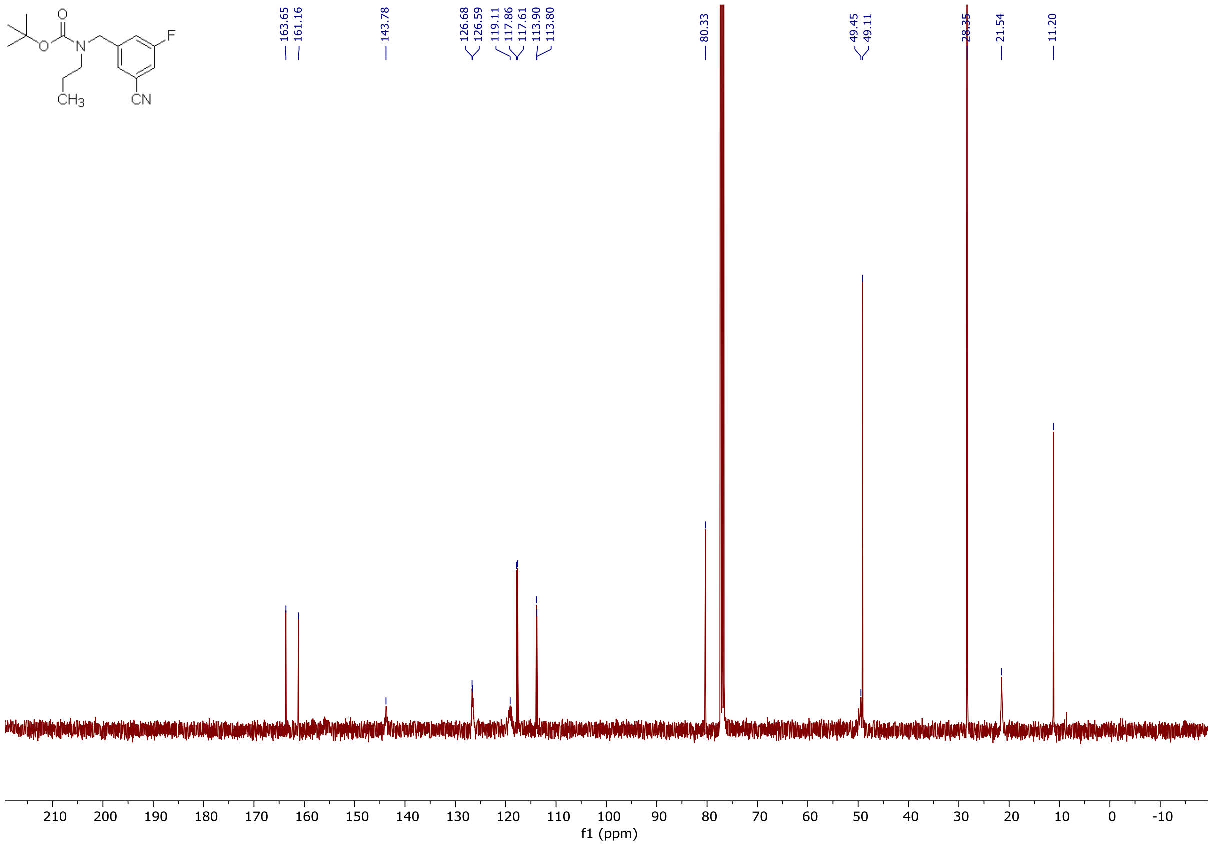
O
O
N
F
CH3
CN
163.65
161.16
143.78
126.68
126.59
119.11
117.86
117.61
113.90
113.80
80.33
49.45
49.11
28.35
21.54
11.20
f1 (ppm)

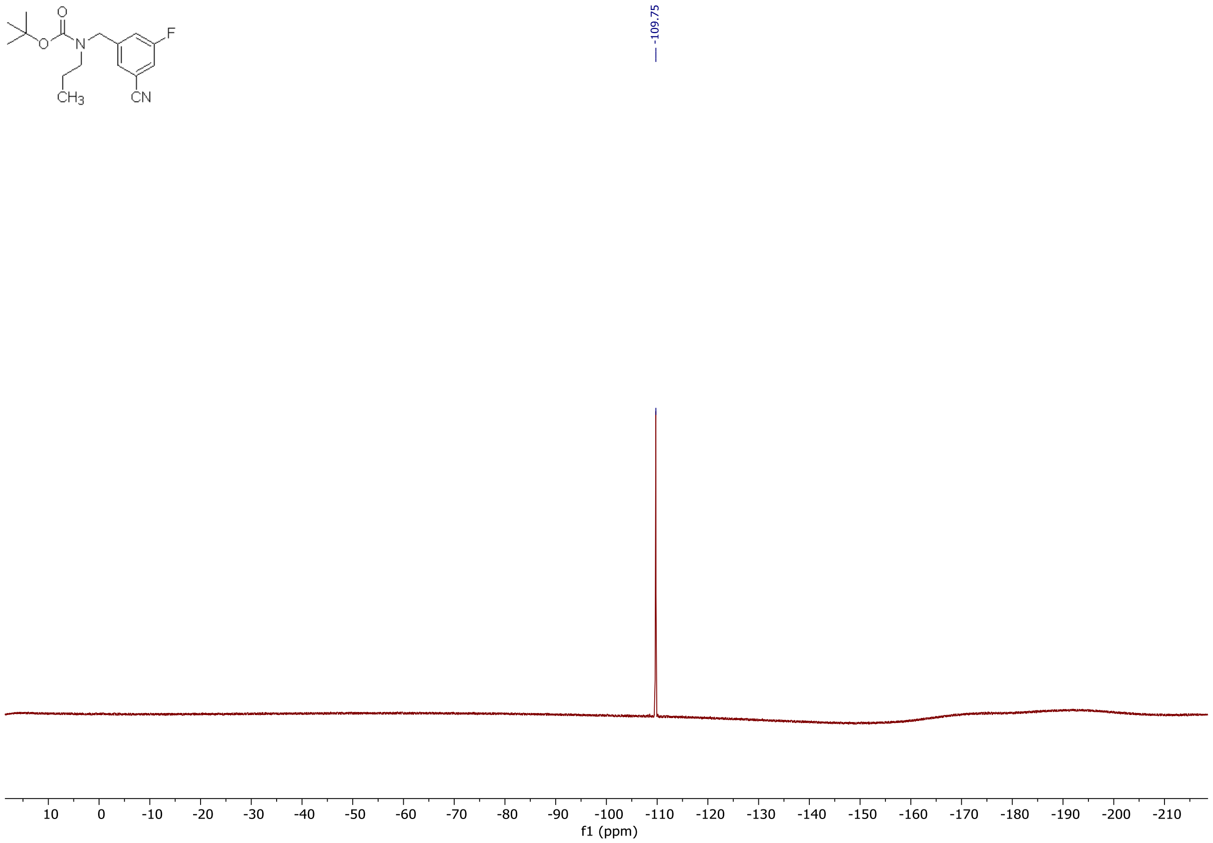
O
O
N
F
CH3
CN
-109.75
f1 (ppm)

Tert-butyl 3-fluoro-5-(1,2,4,5-tetrazin-3-yl)benzyl(propyl)carbamate

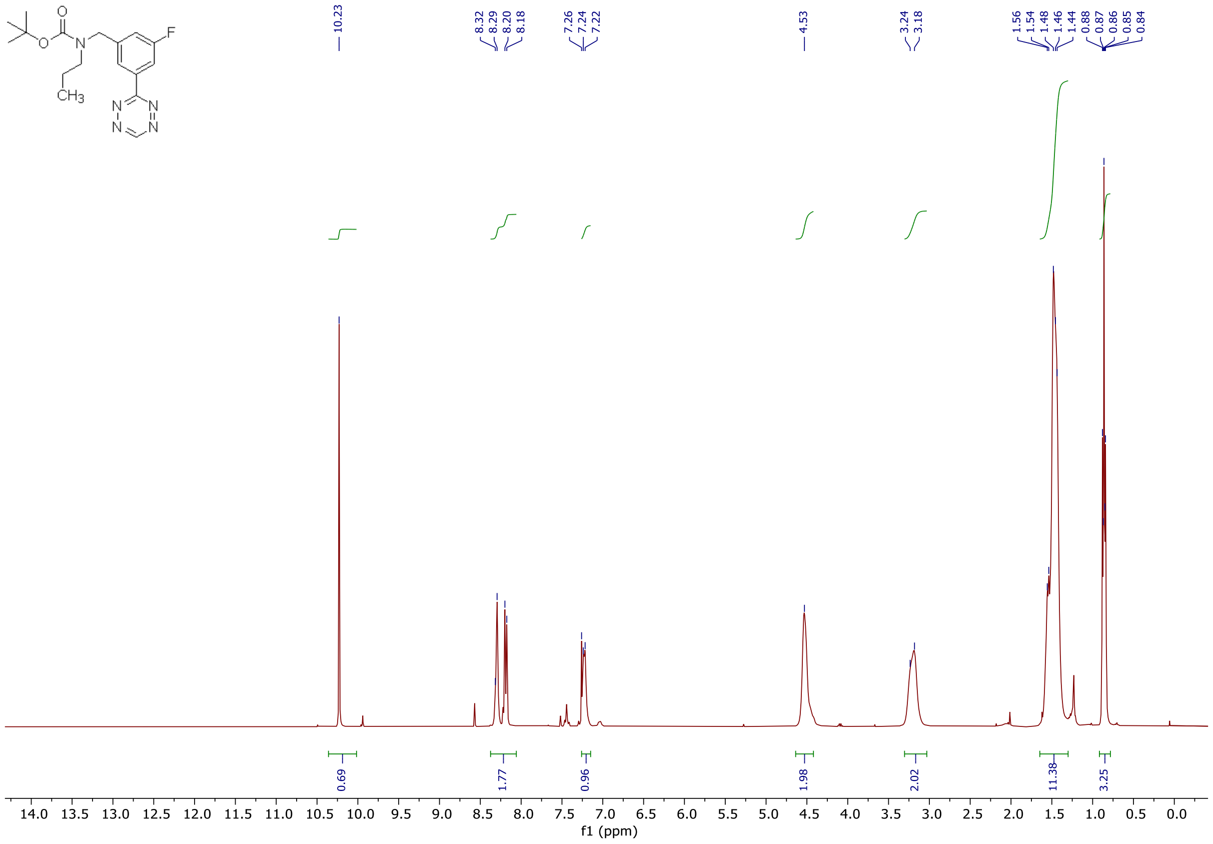

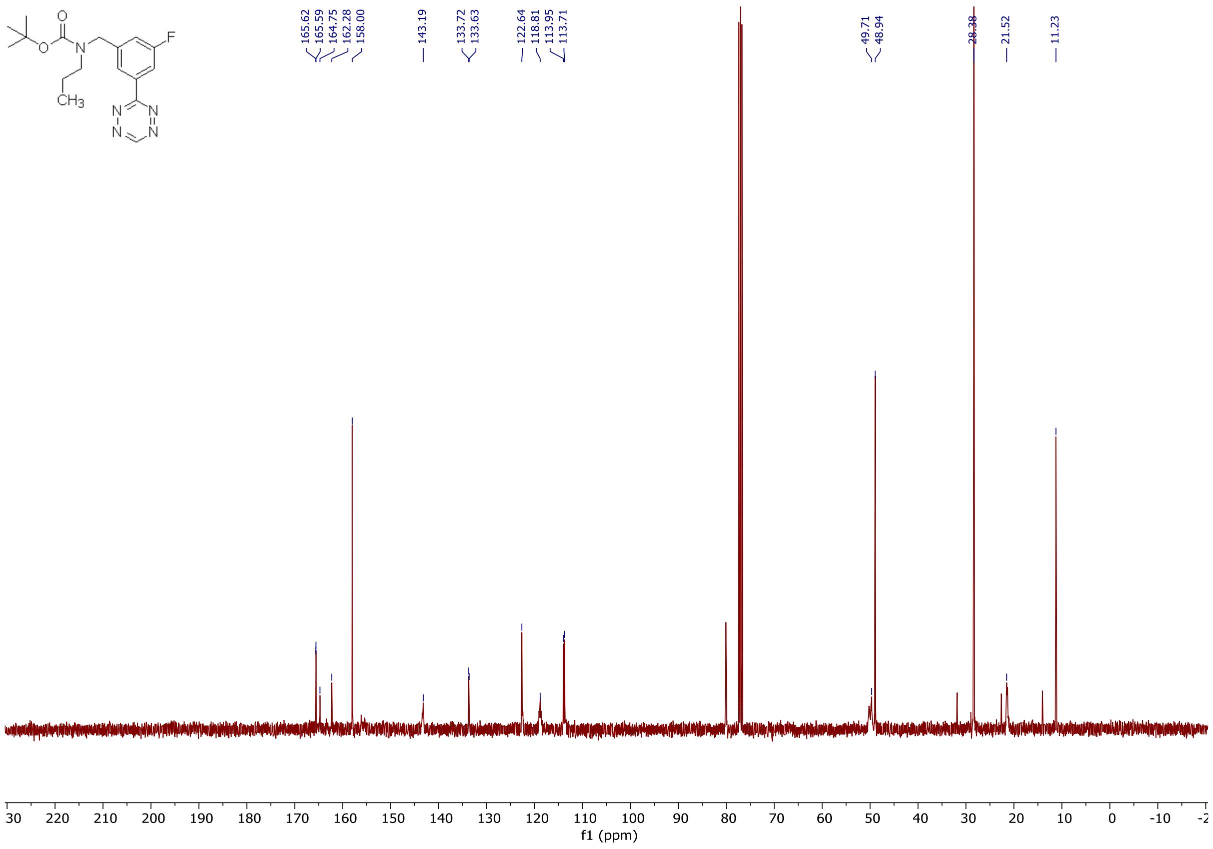

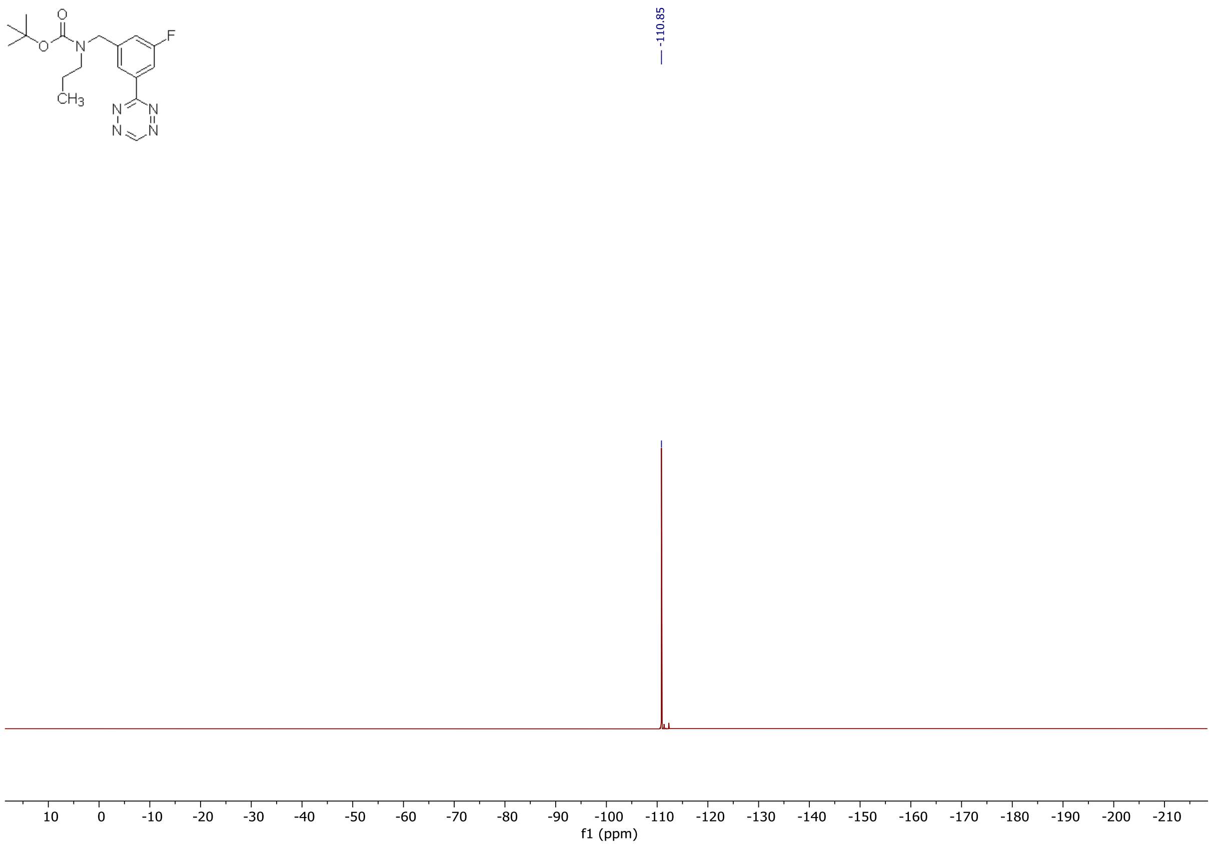

N-(3-Fluoro-5-(1,2,4,5-tetrazin-3-yl)benzyl)propylamine hydrochloride

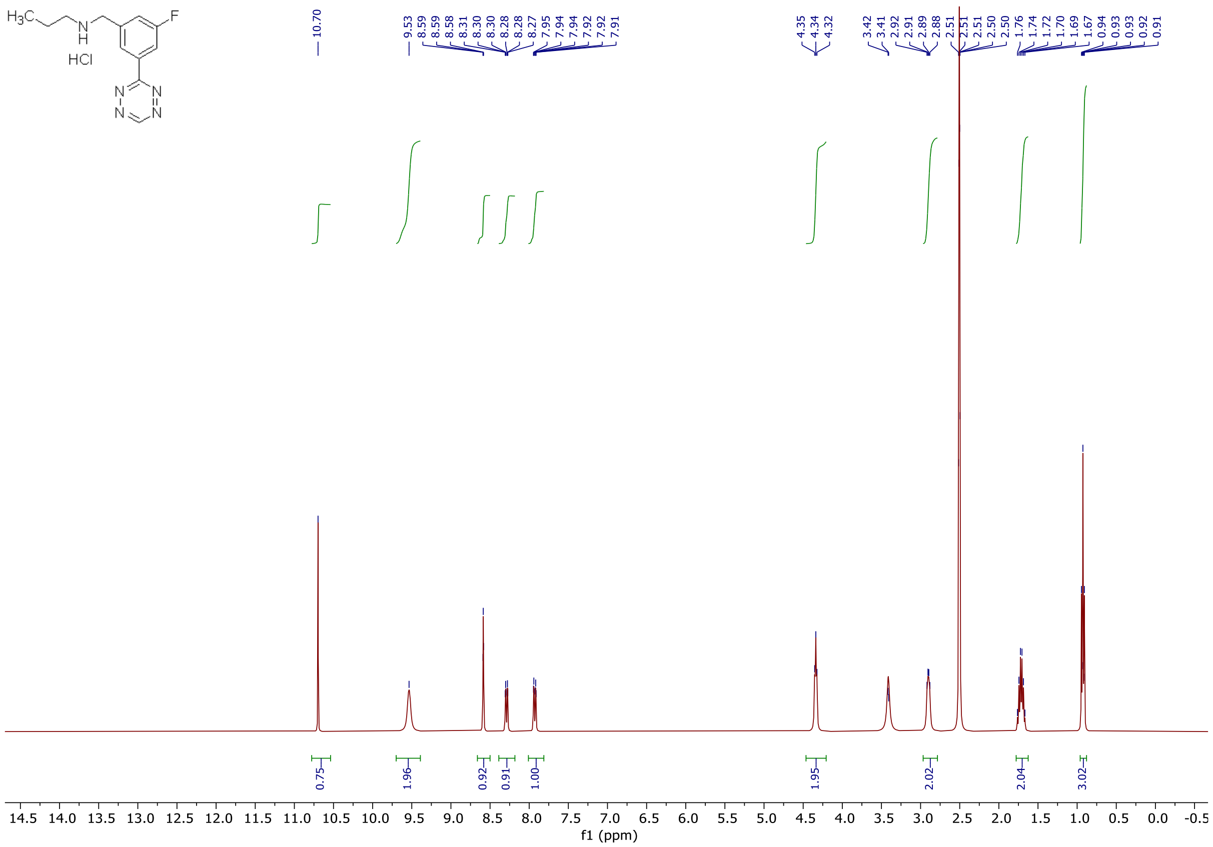

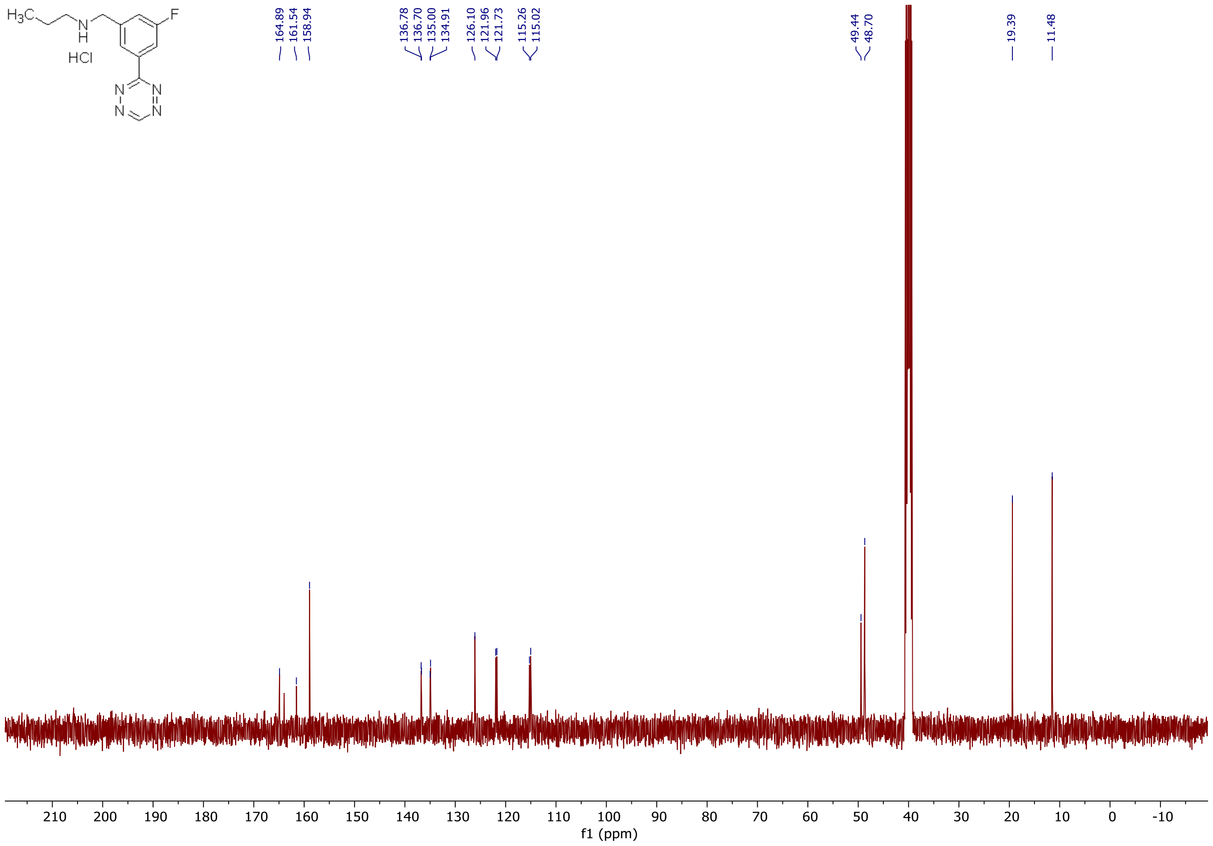
H3C
N
H
F
HCl
N
N
N
N
164.89
161.54
158.94
136.78
136.70
135.00
134.91
126.10
121.96
121.73
115.26
115.02
49.44
48.70
19.39
11.48
210 200 190 180 170 160 150 140 130 120 110 100 90 80 70 60 50 40 30 20 10 0 -10
f1 (ppm)

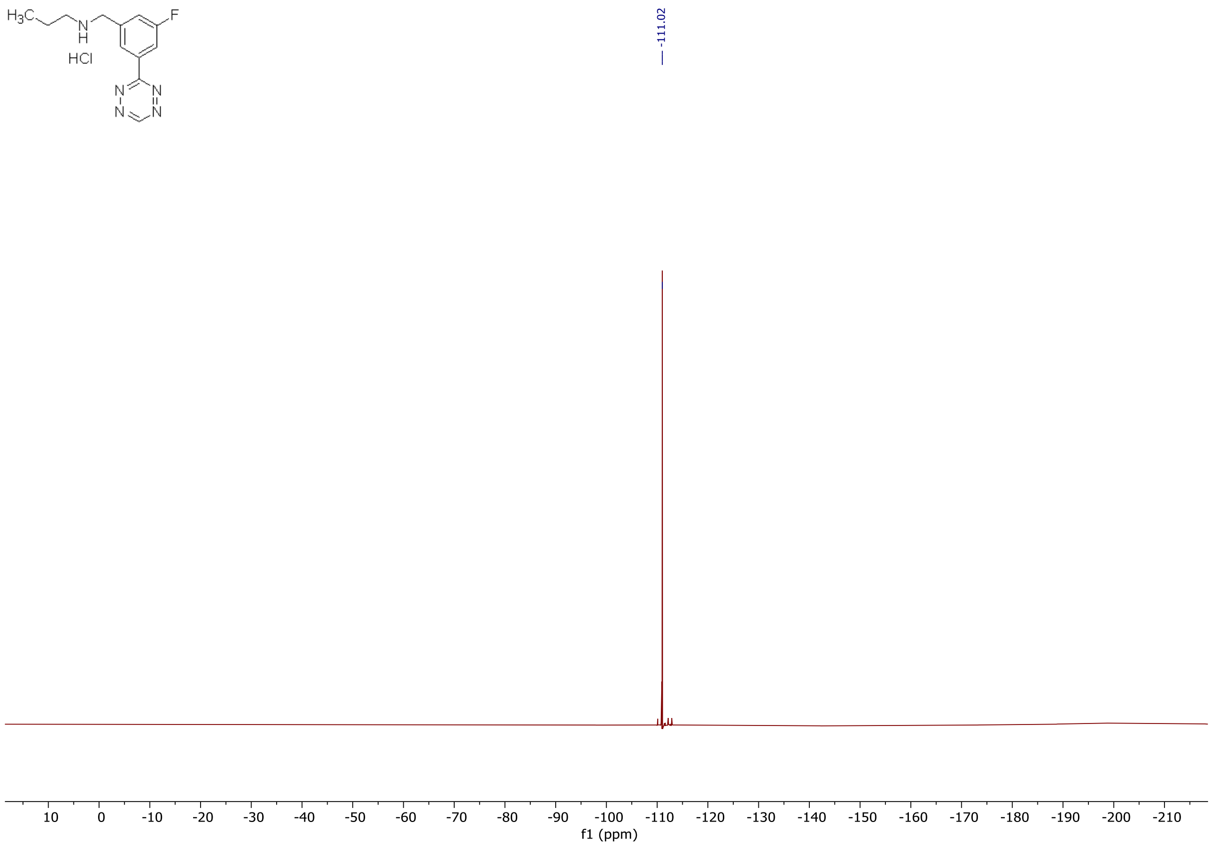
H3C
N
H
F
HCl
N
N
N
N
-111.02
10 0 -10 -20 -30 -40 -50 -60 -70 -80 -90 -100 -110 -120 -130 -140 -150 -160 -170 -180 -190 -200 -210
f1 (ppm)

3-Hydroxy-5-iodobenzonitrile

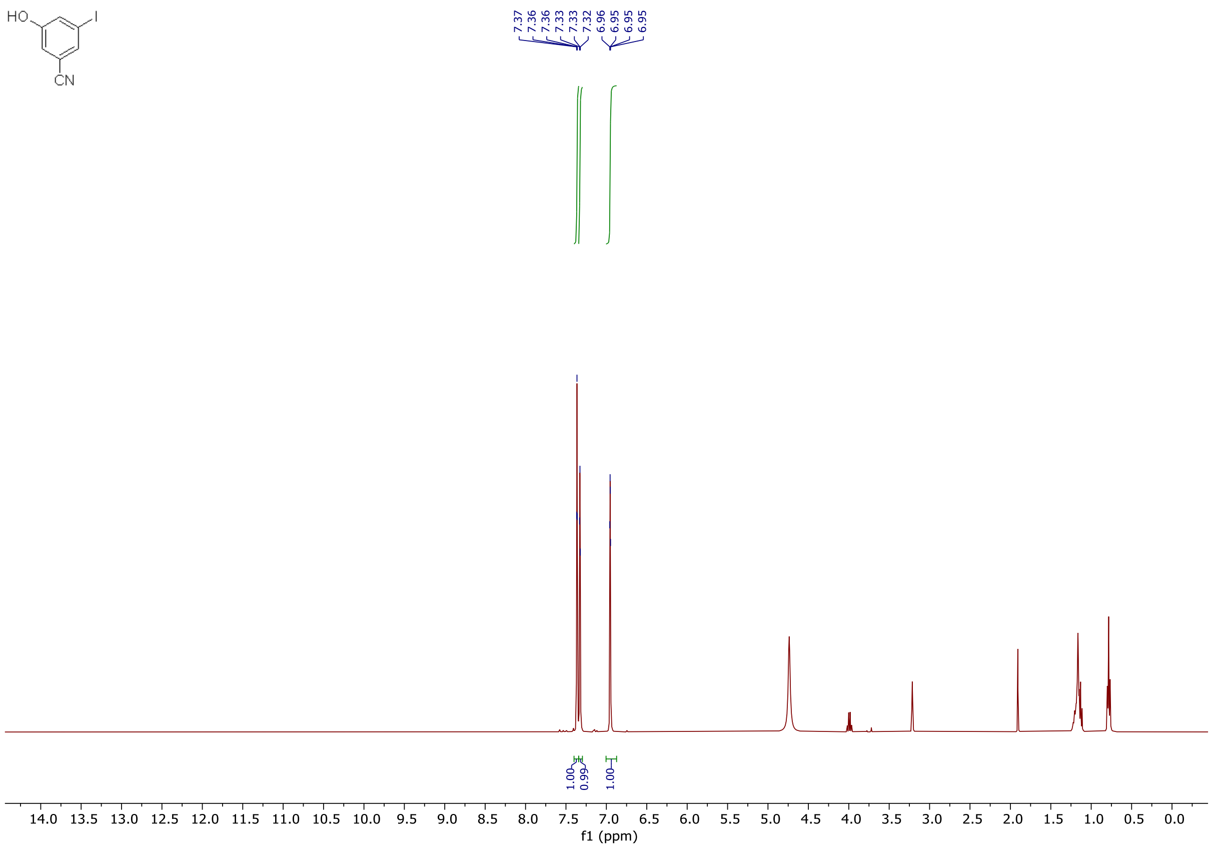

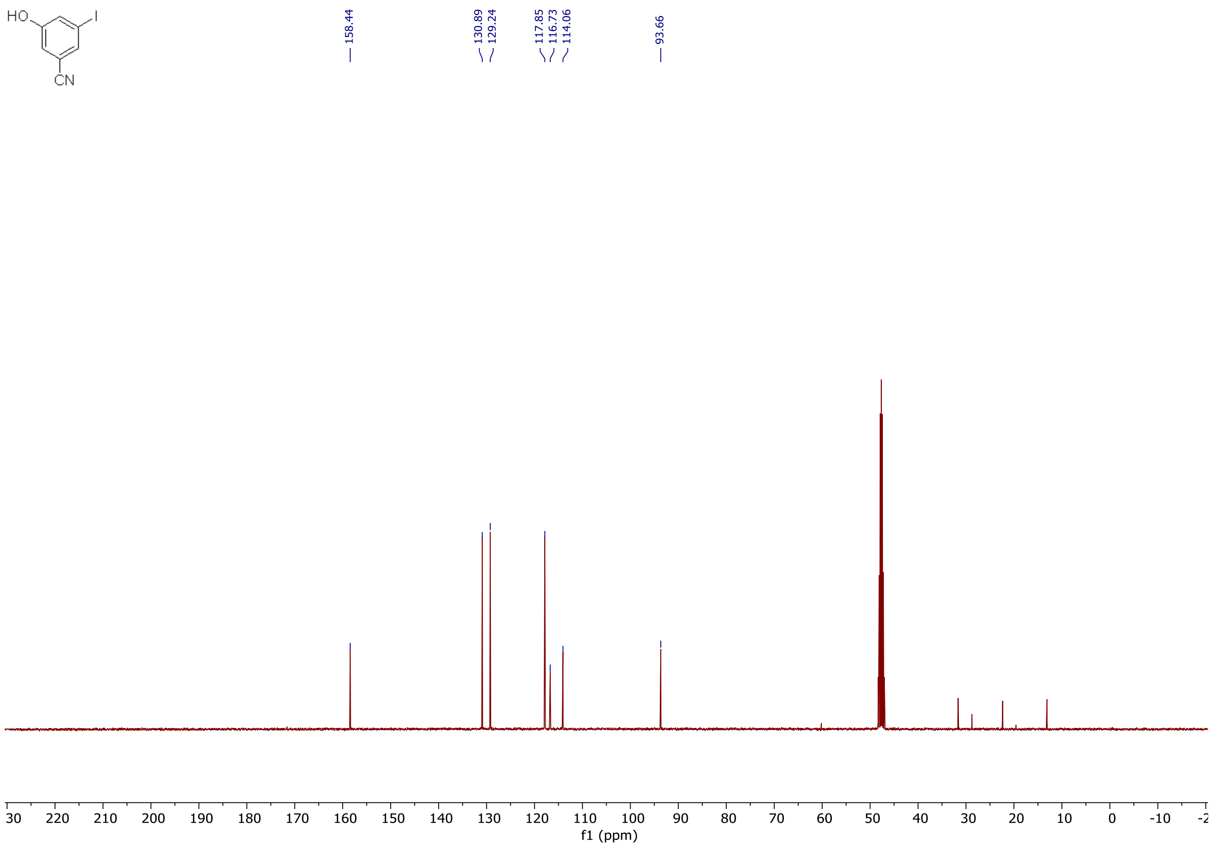

3-Ethoxy-5-iodobenzonitrile

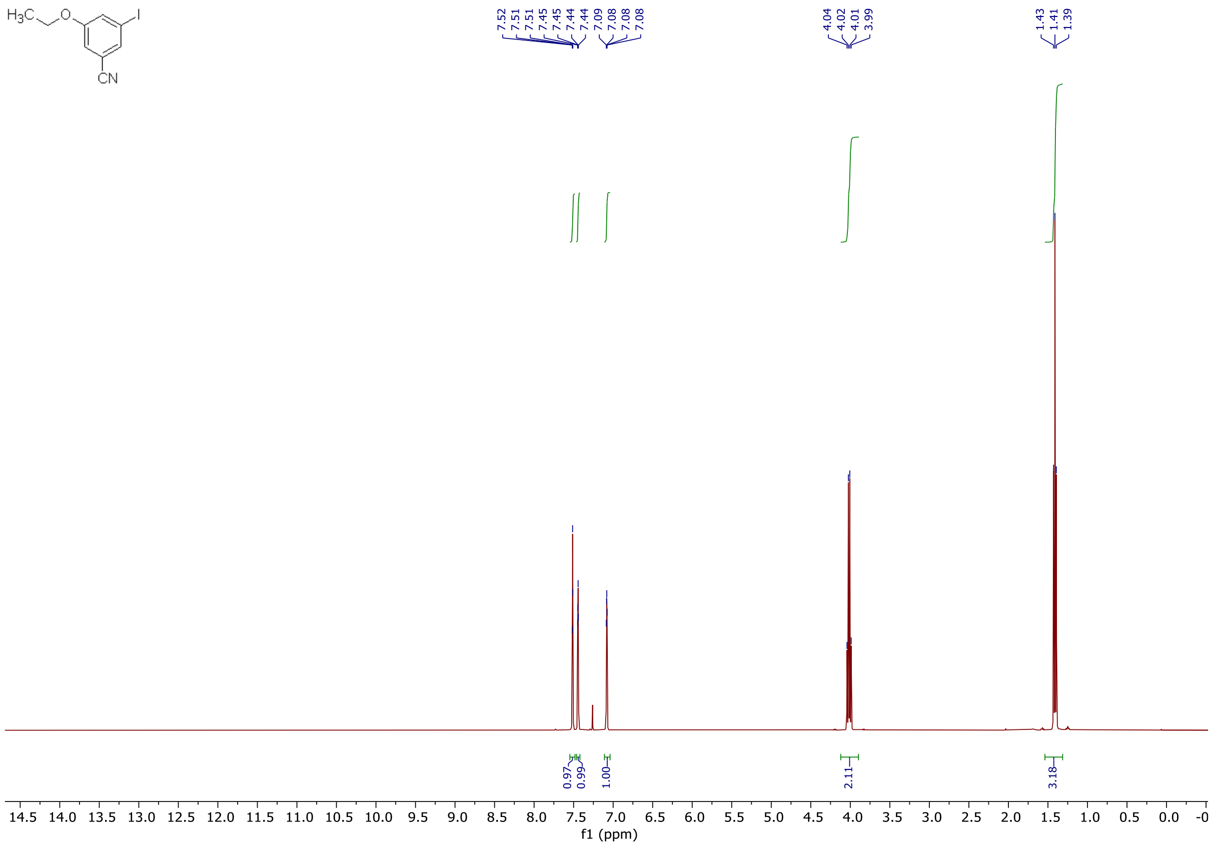

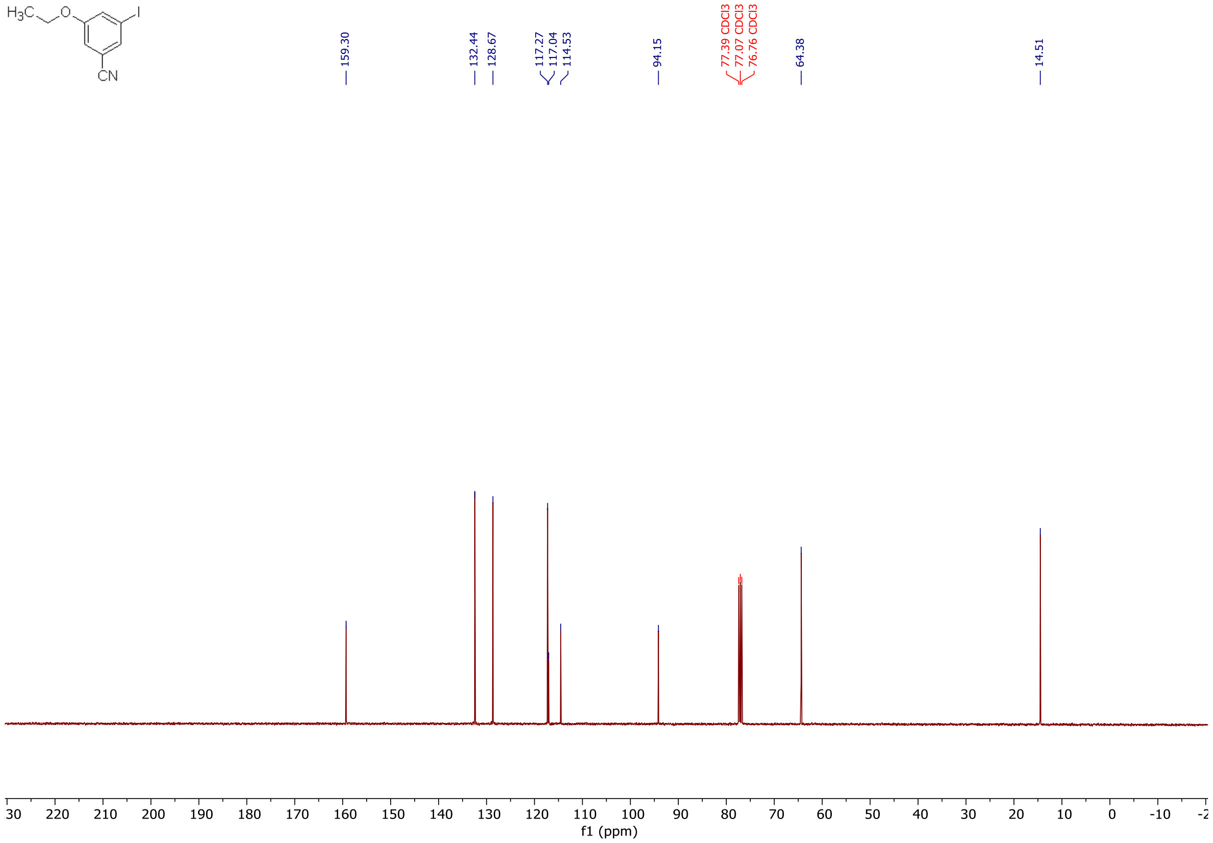

3-(3-Ethoxy-5-iodophenyl)-1,2,4,5-tetrazine

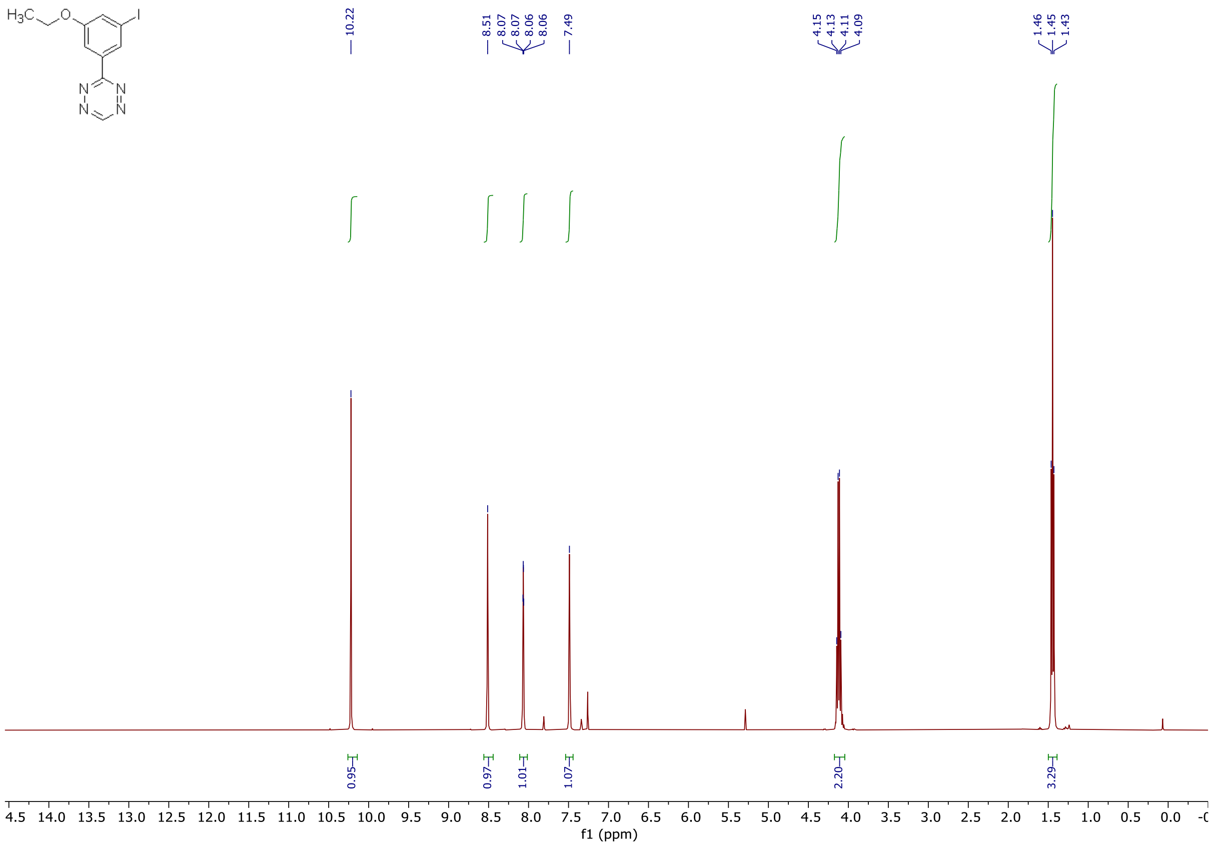

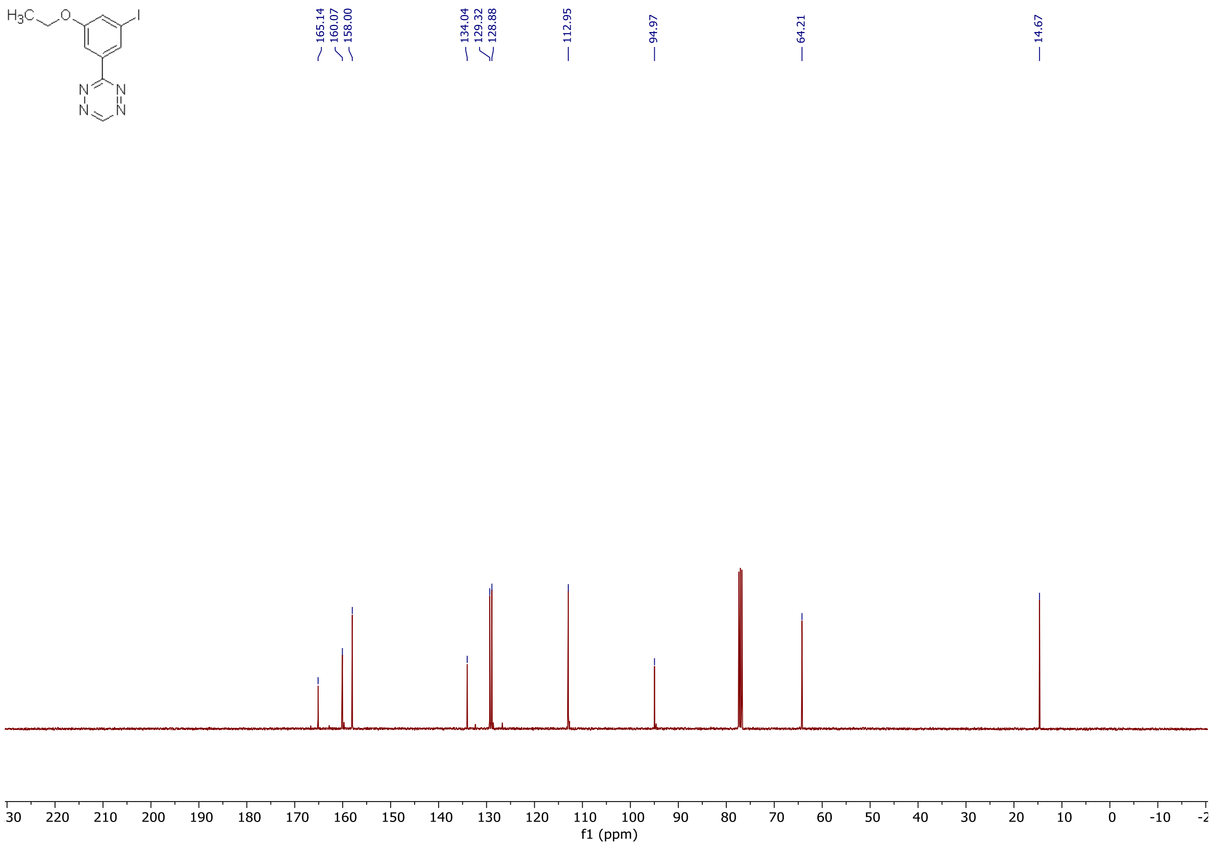

3-(3-Ethoxy-5-(trimethylstannyl)phenyl)-1,2,4,5-tetrazine

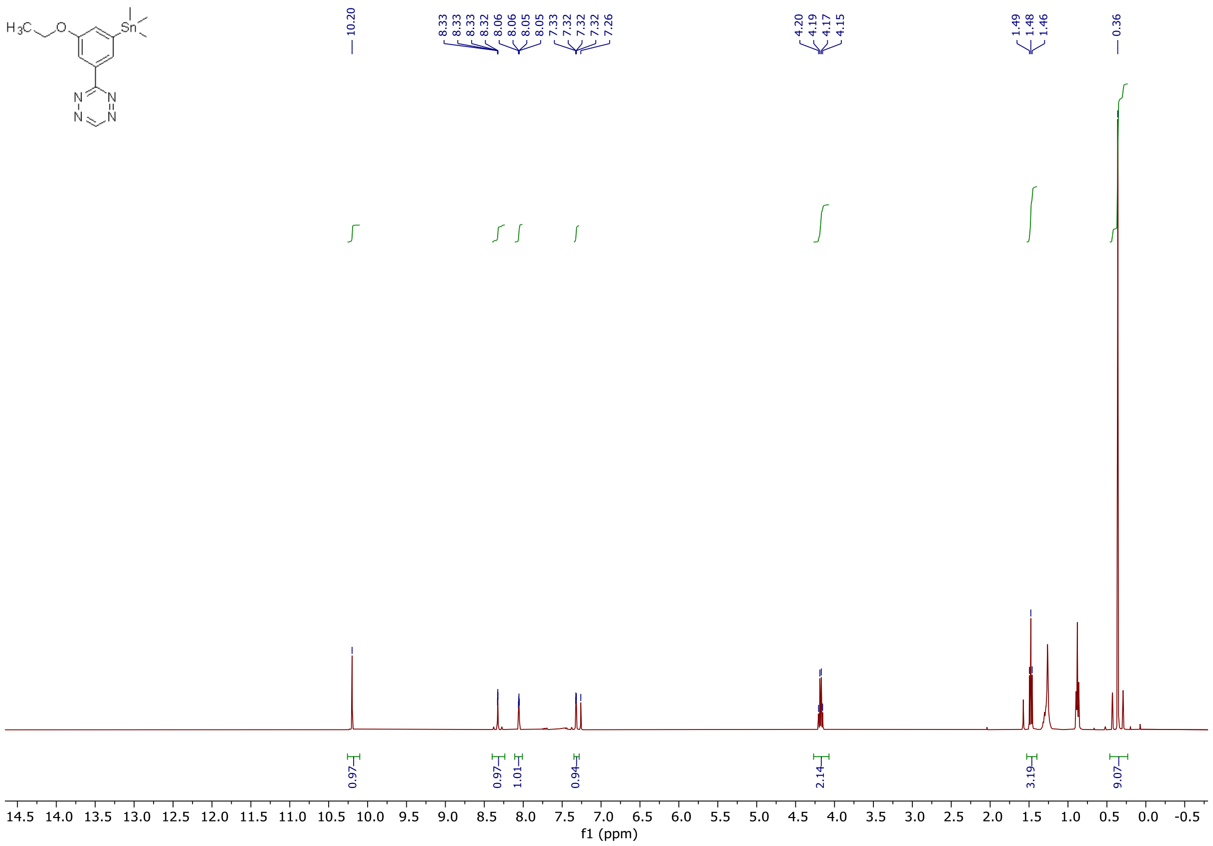

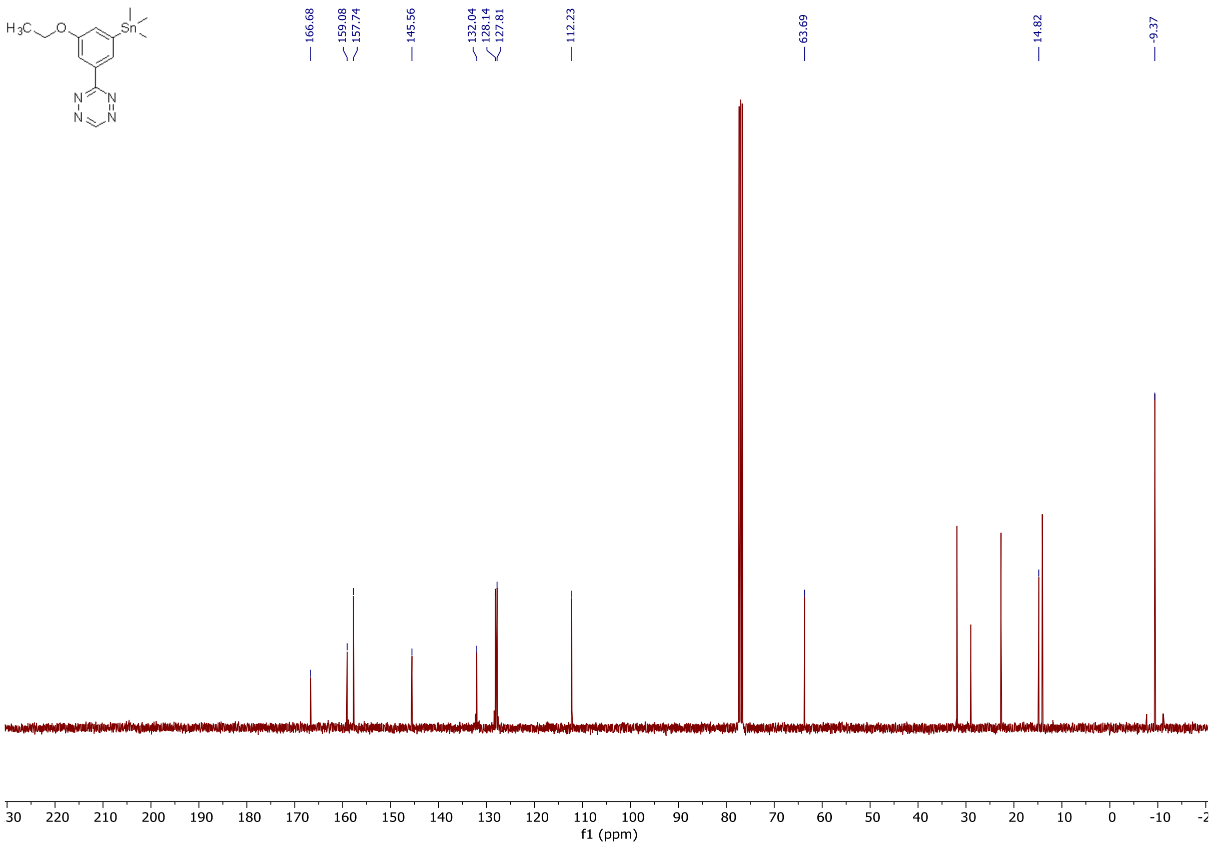

3-Iodo-5-propoxybenzonitrile

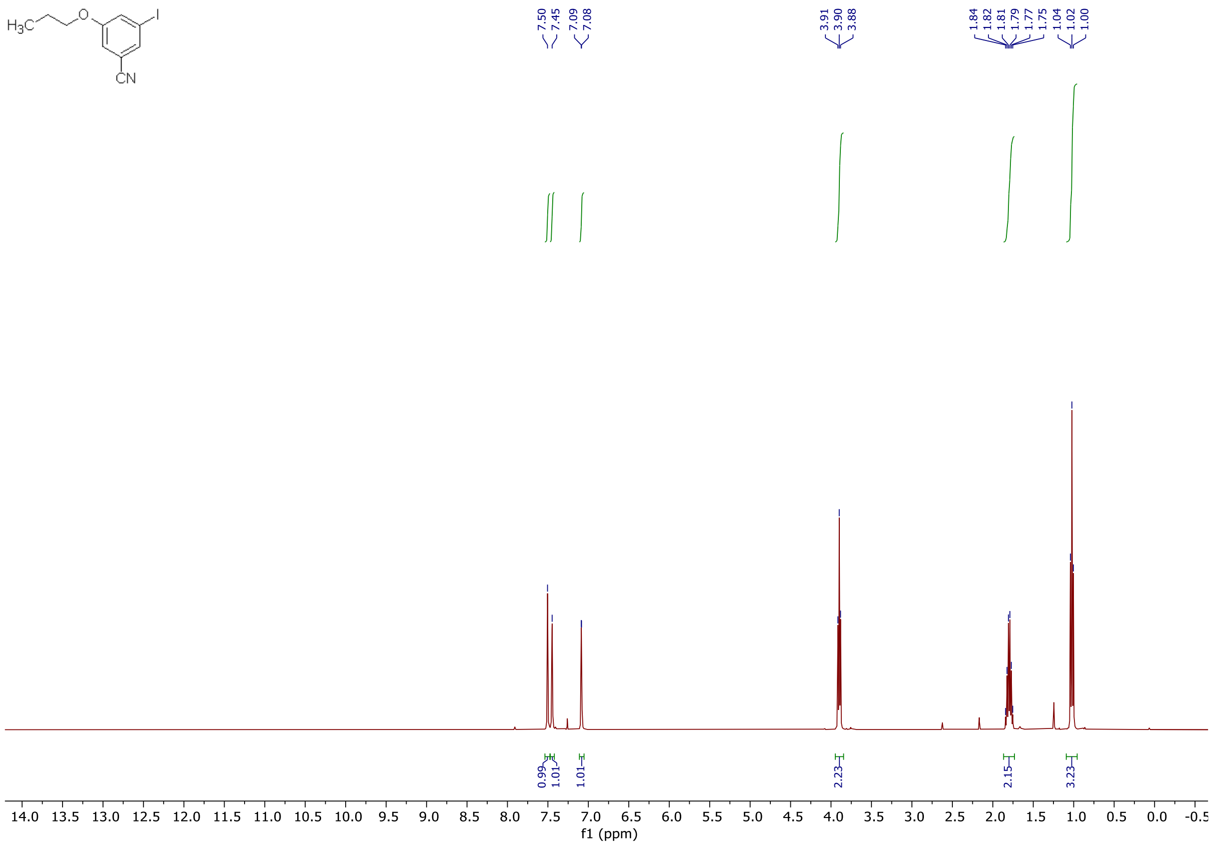

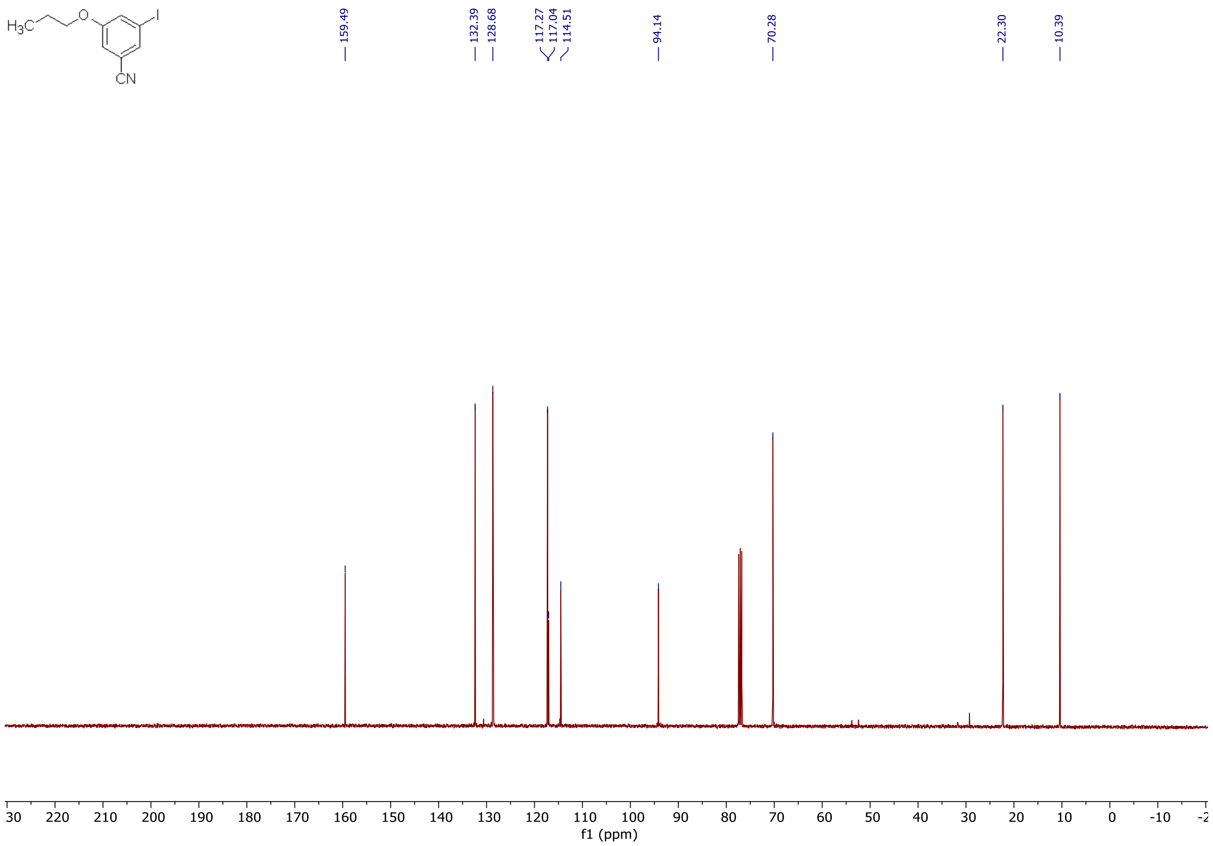

3-(3-Iodo-5-propoxyphenyl)-1,2,4,5-tetrazine

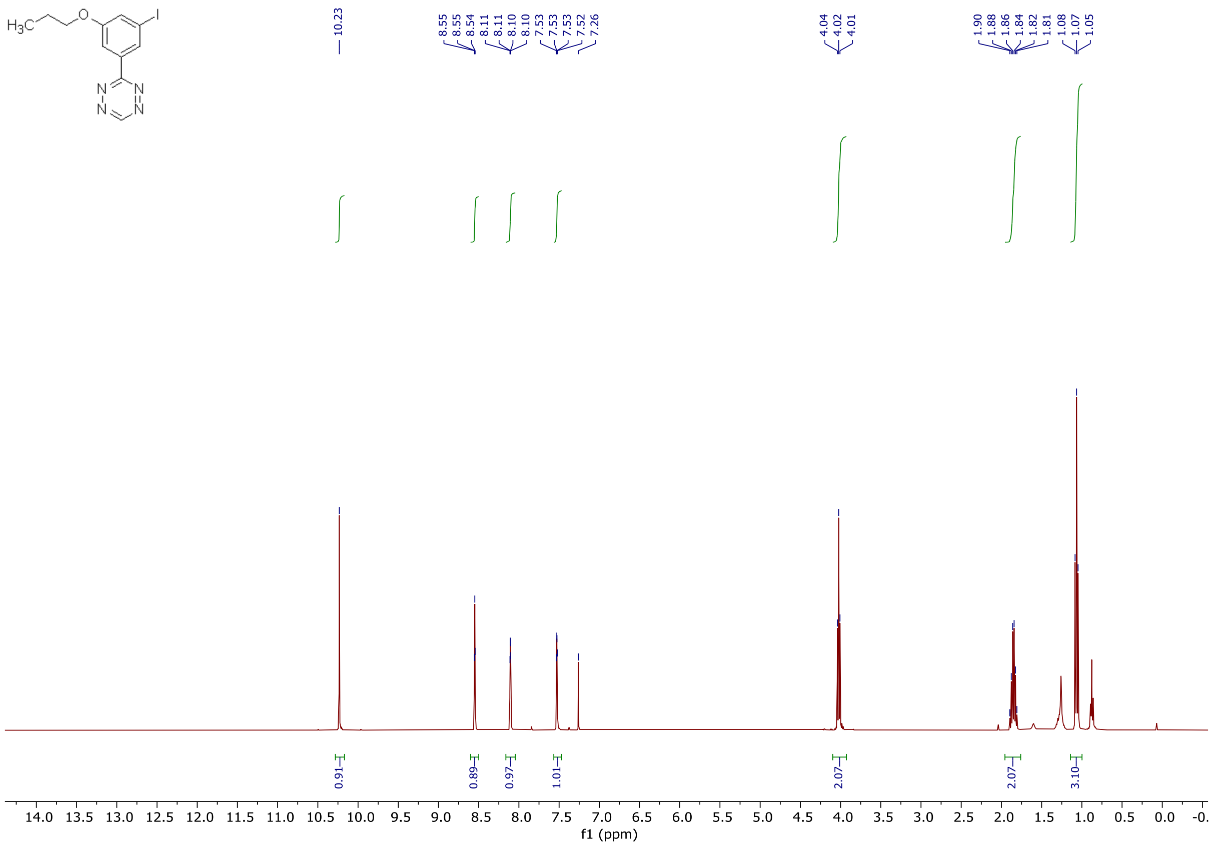

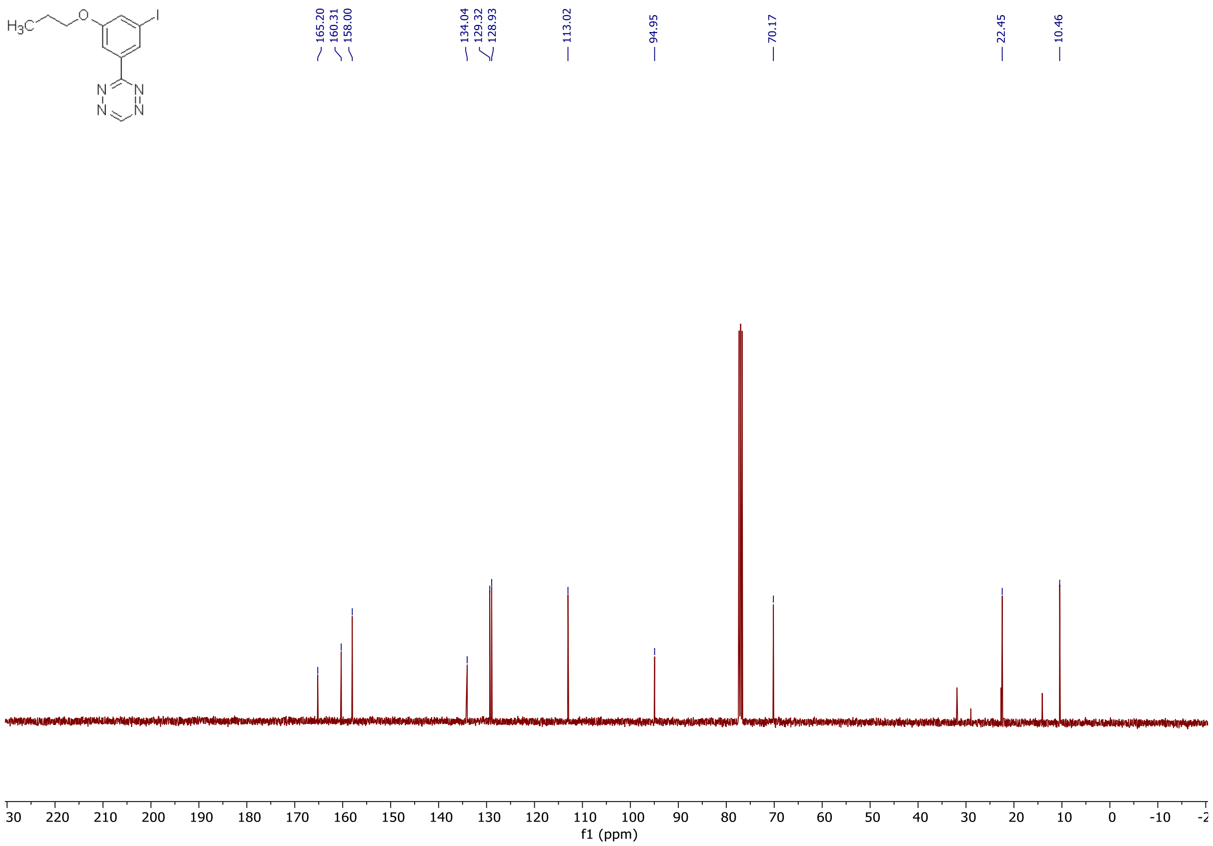

3-(3-Propoxy-5-(trimethylstannyl)phenyl)-1,2,4,5-tetrazine

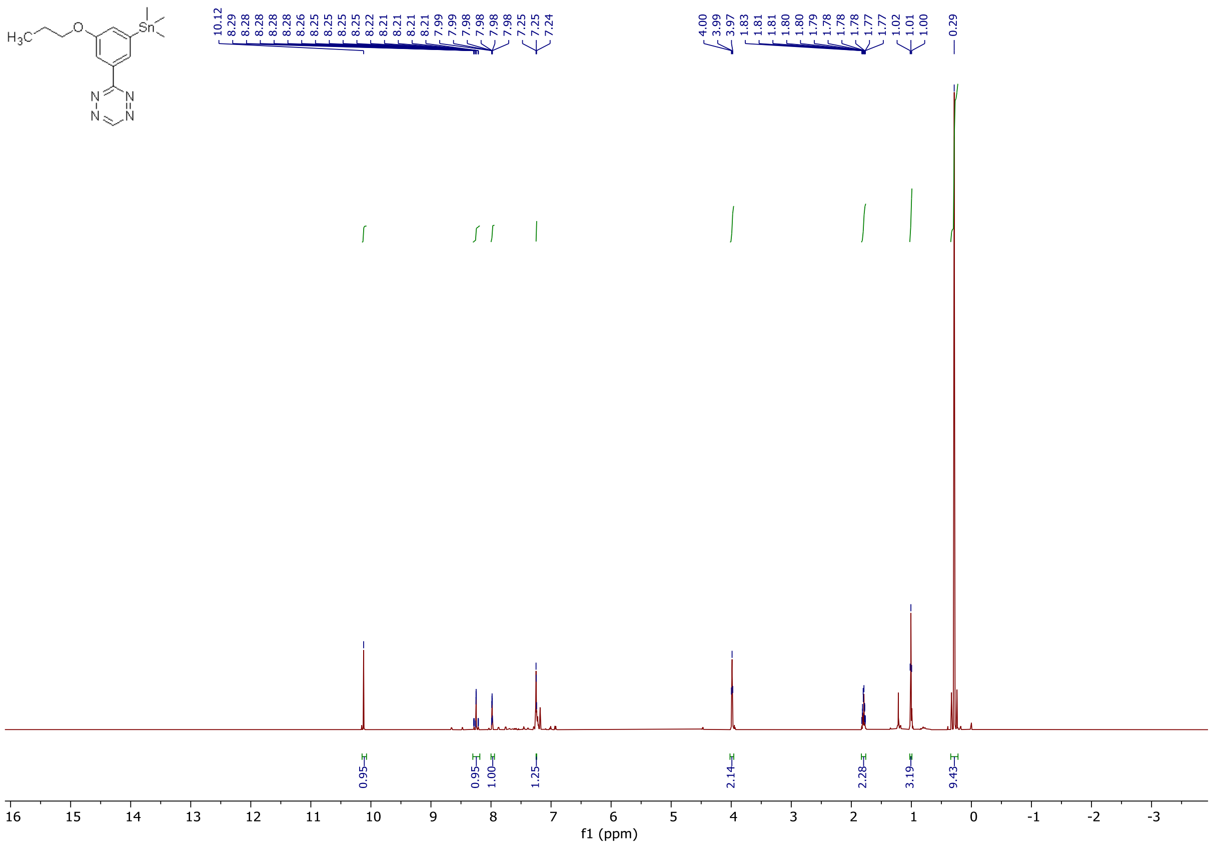

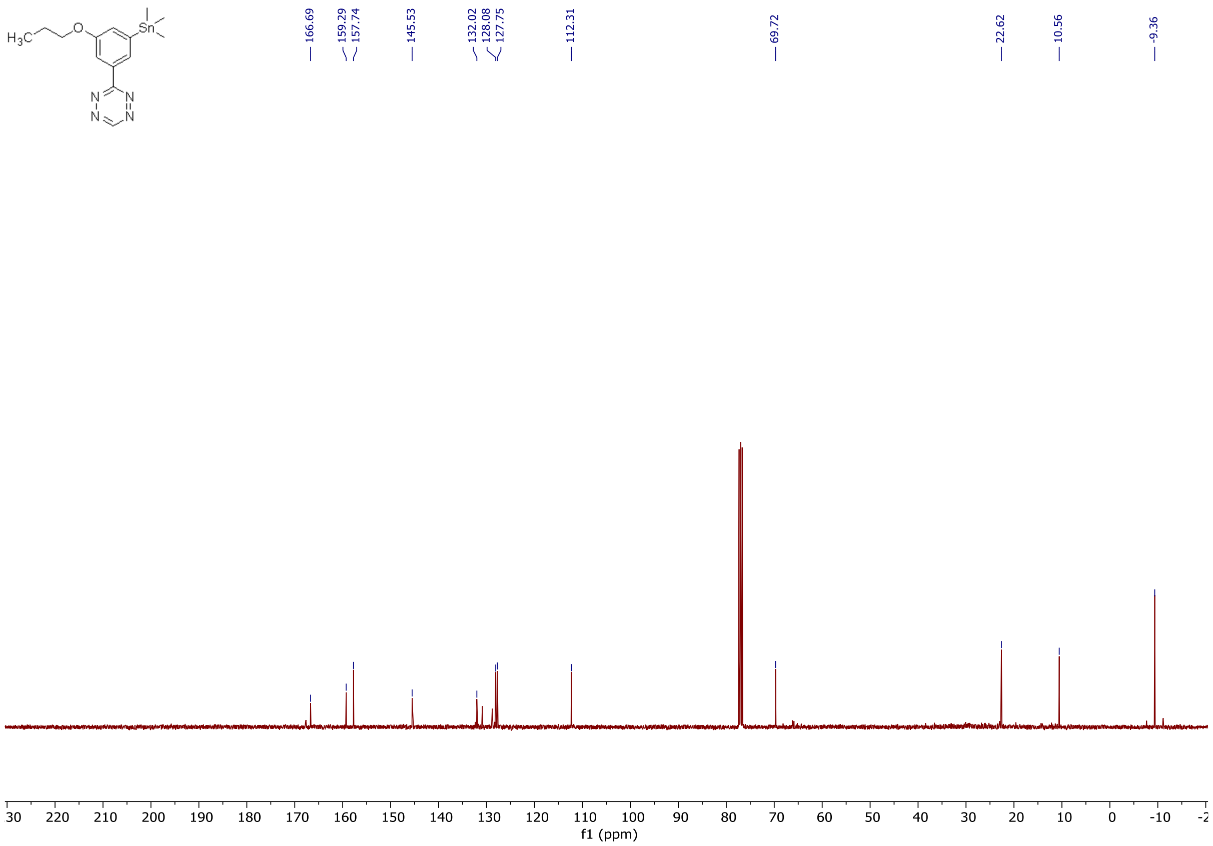

3-(Benzyloxy)-5-iodobenzonitrile

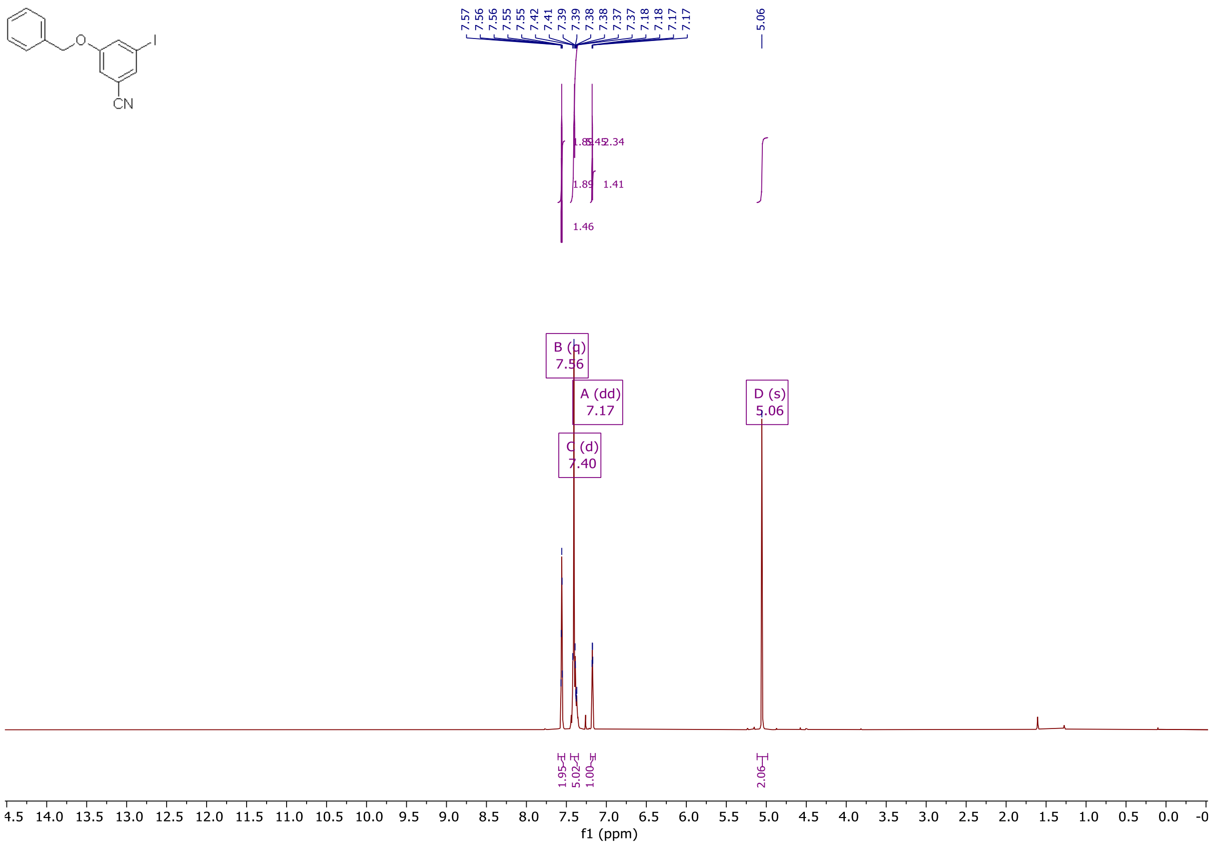

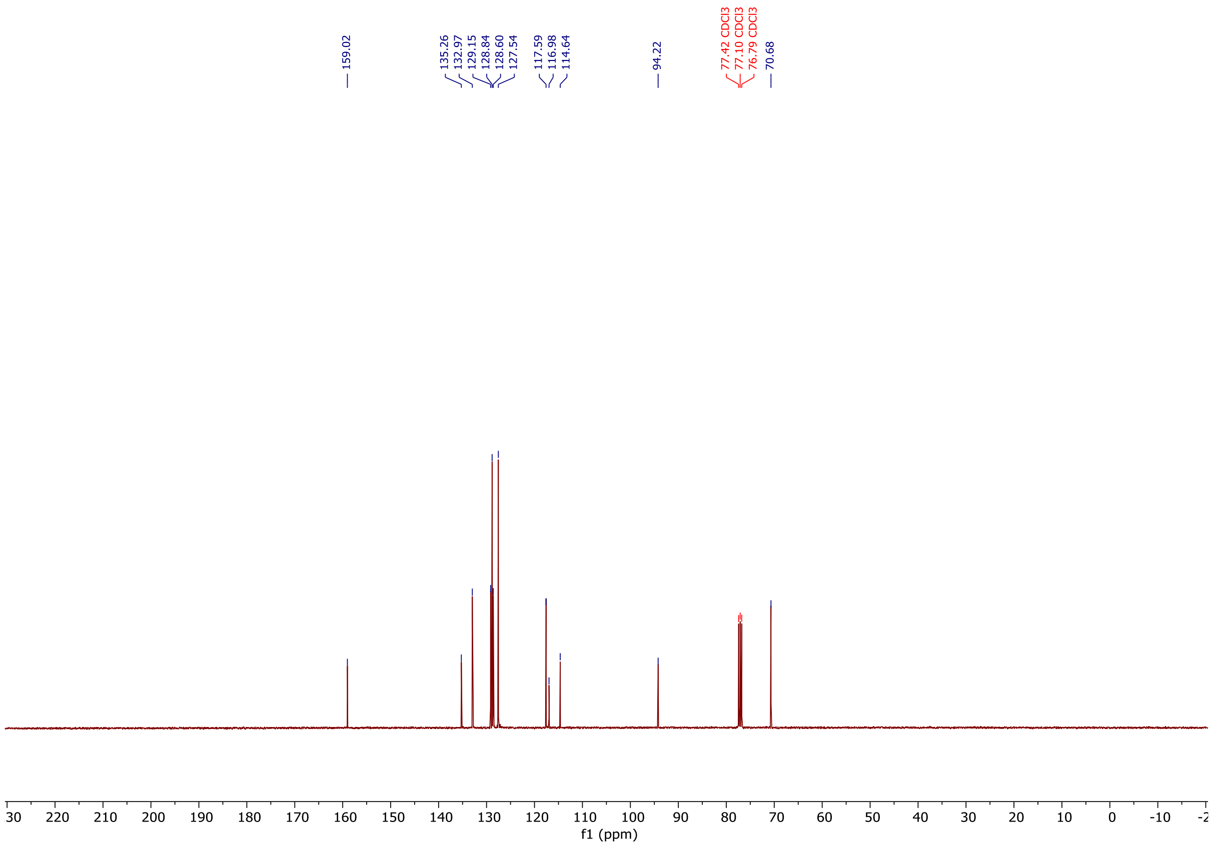

3-(3-(Benzyloxy)-5-iodophenyl)-1,2,4,5-tetrazine

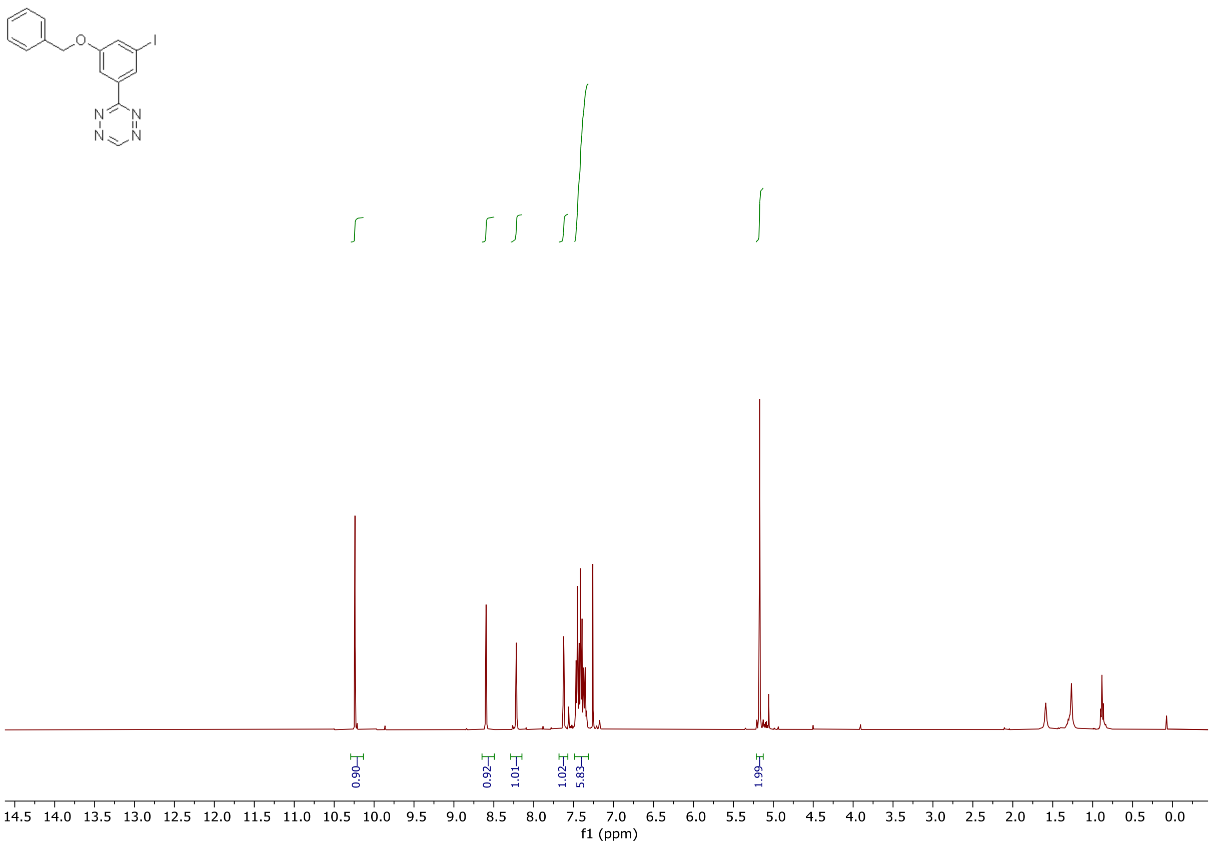

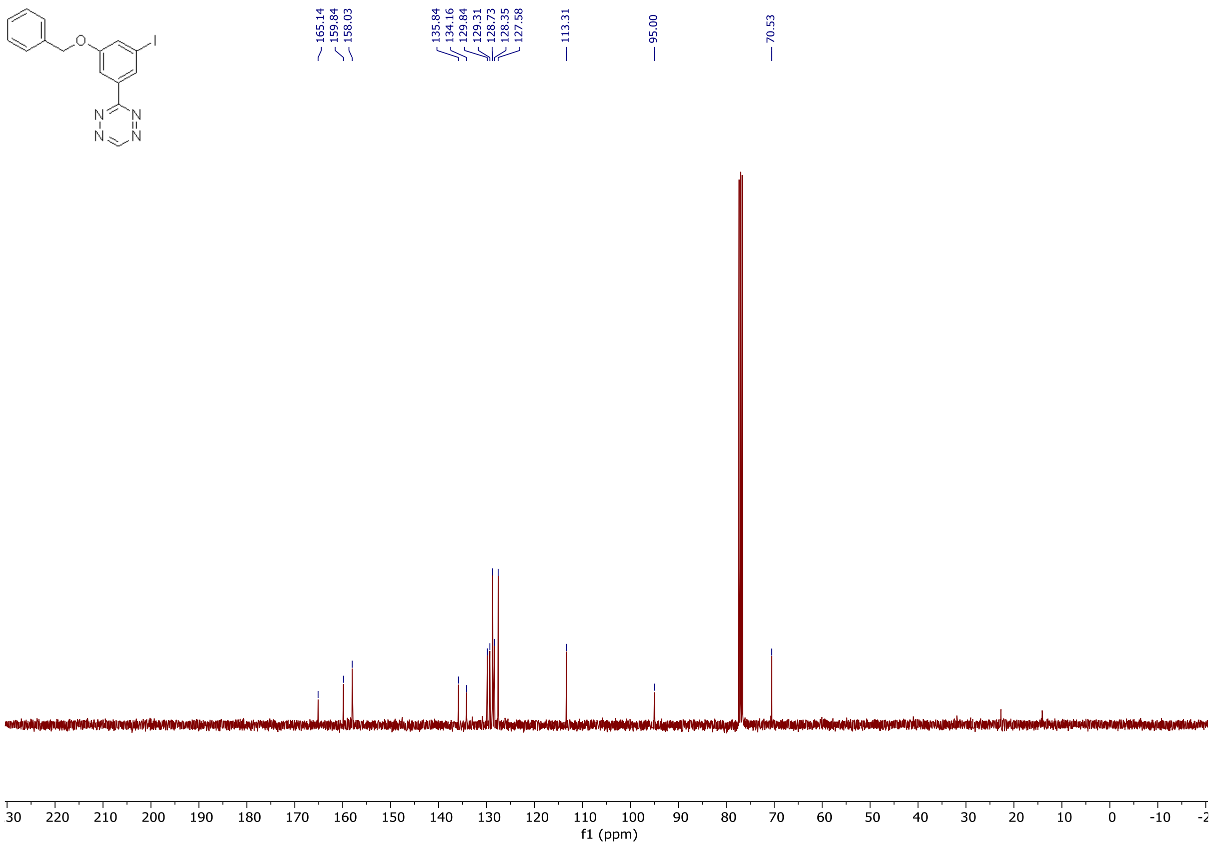

3-(3-(Benzyloxy)-5-(trimethylstannyl)phenyl)-1,2,4,5-tetrazine

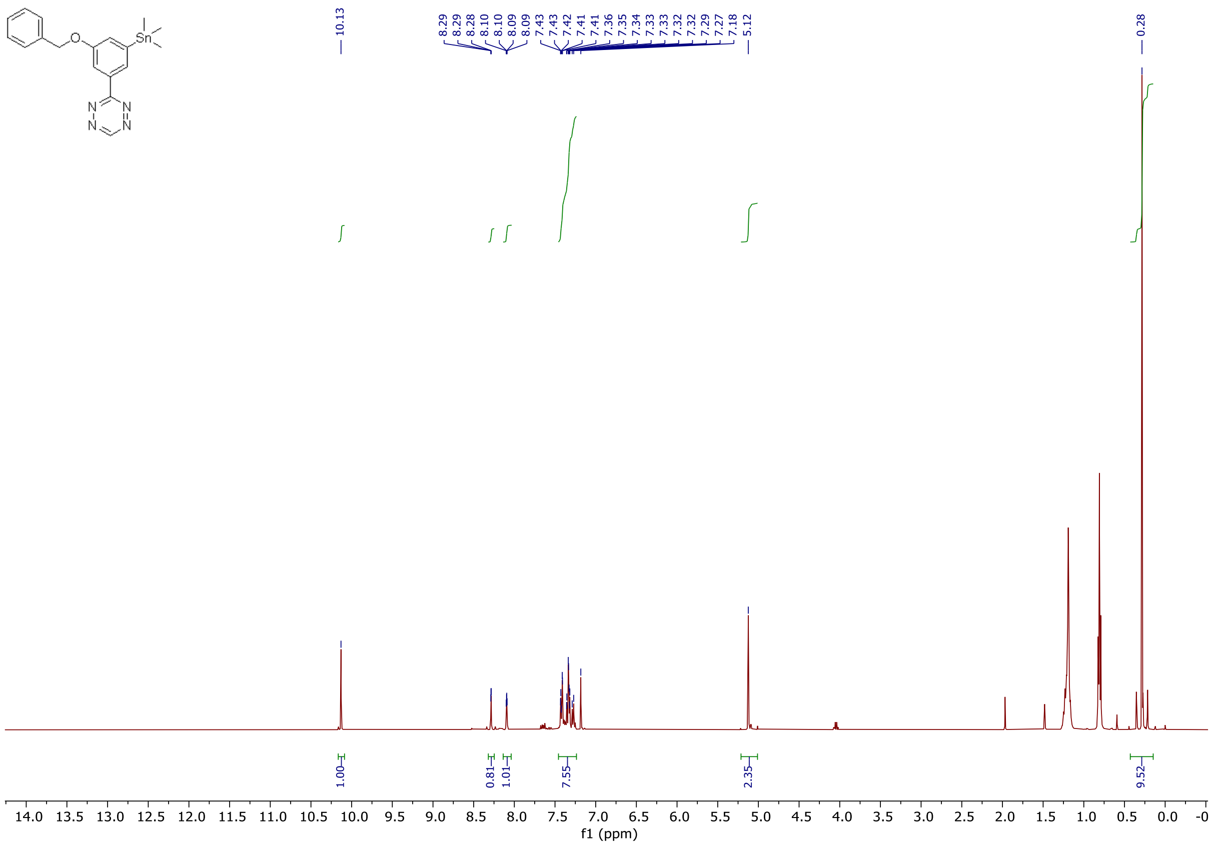

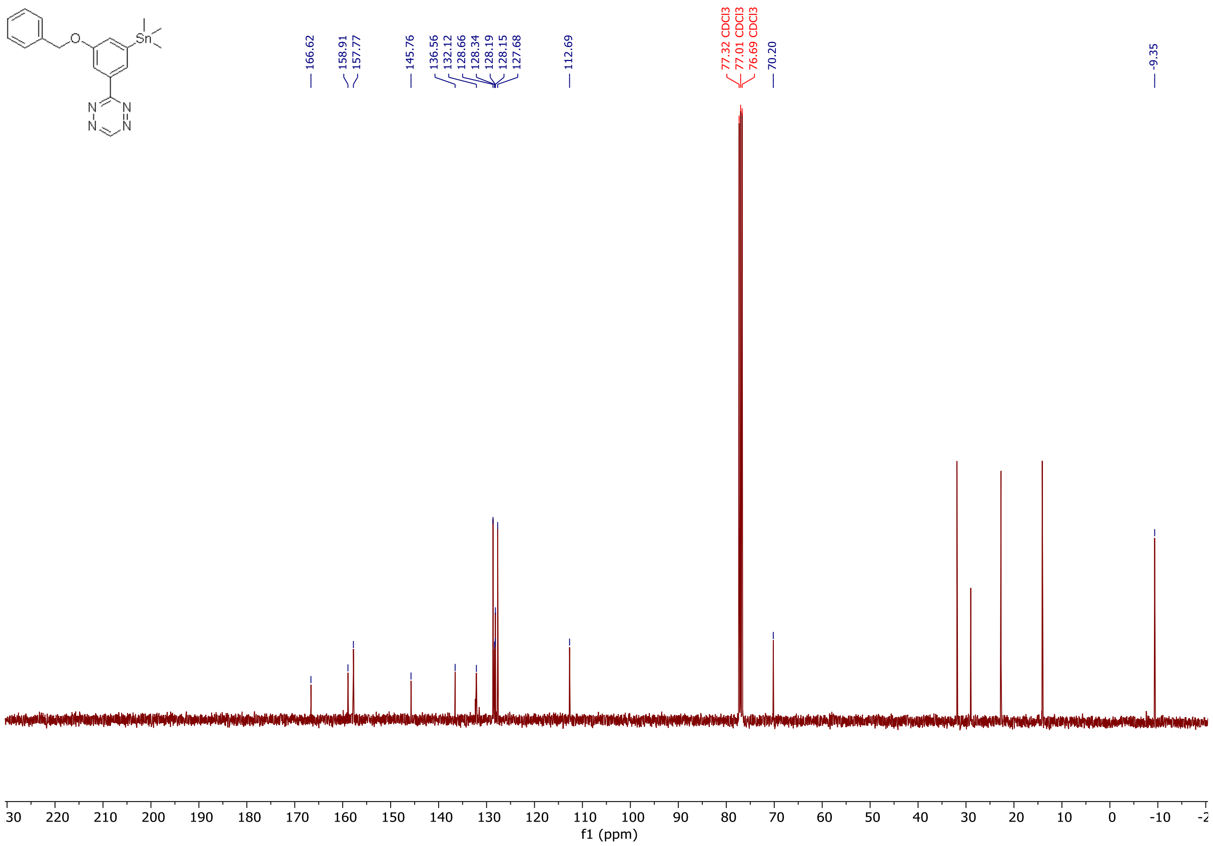

N-(3-Cyano-5-iodophenyl)propionamide

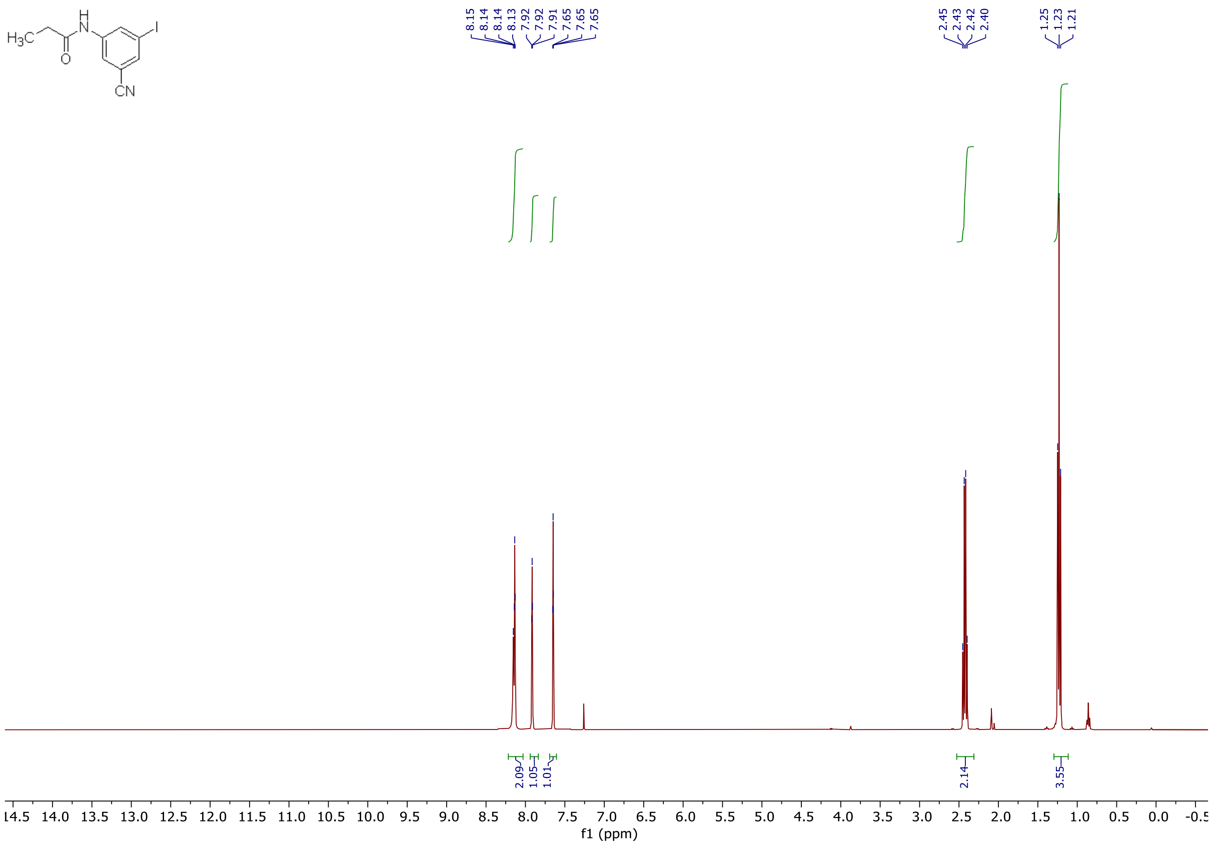

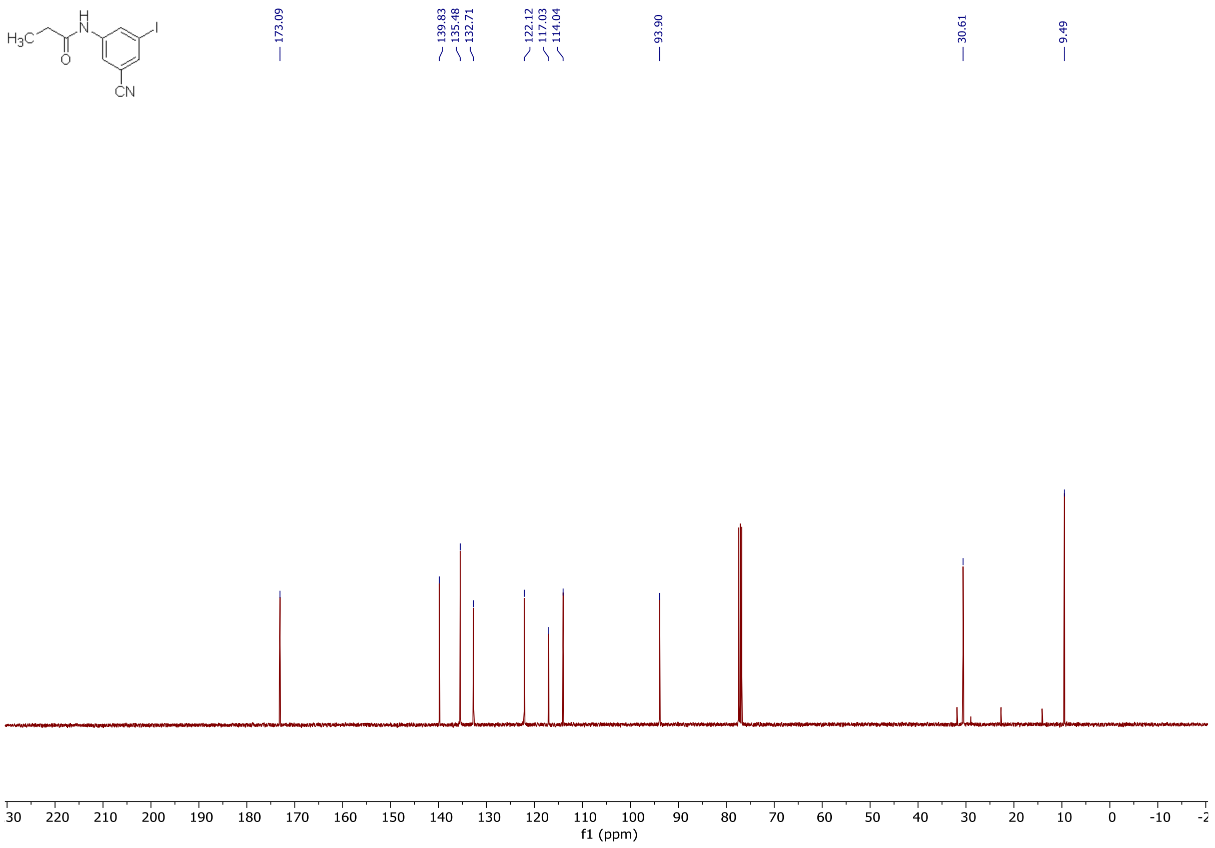

N-(3-Iodo-5-(1,2,4,5-tetrazin-3-yl)phenyl)propionamide

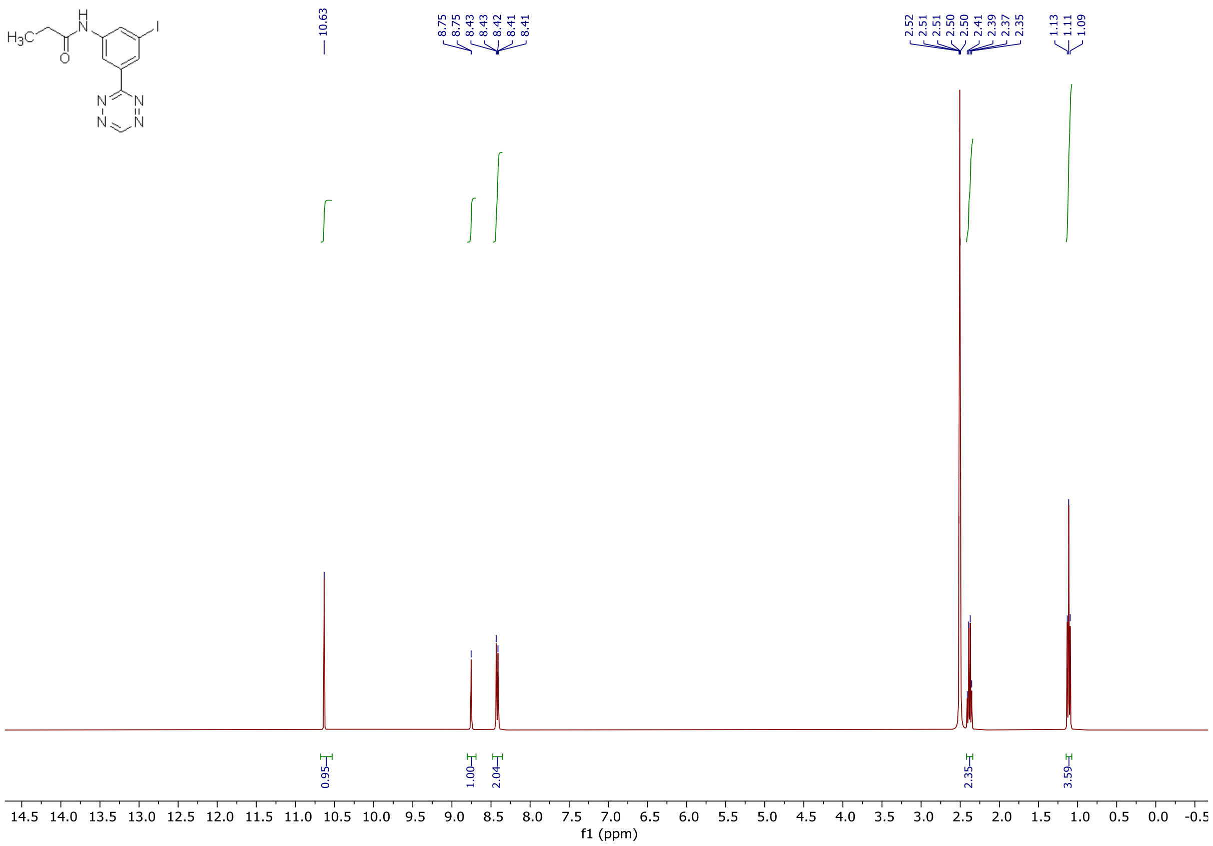

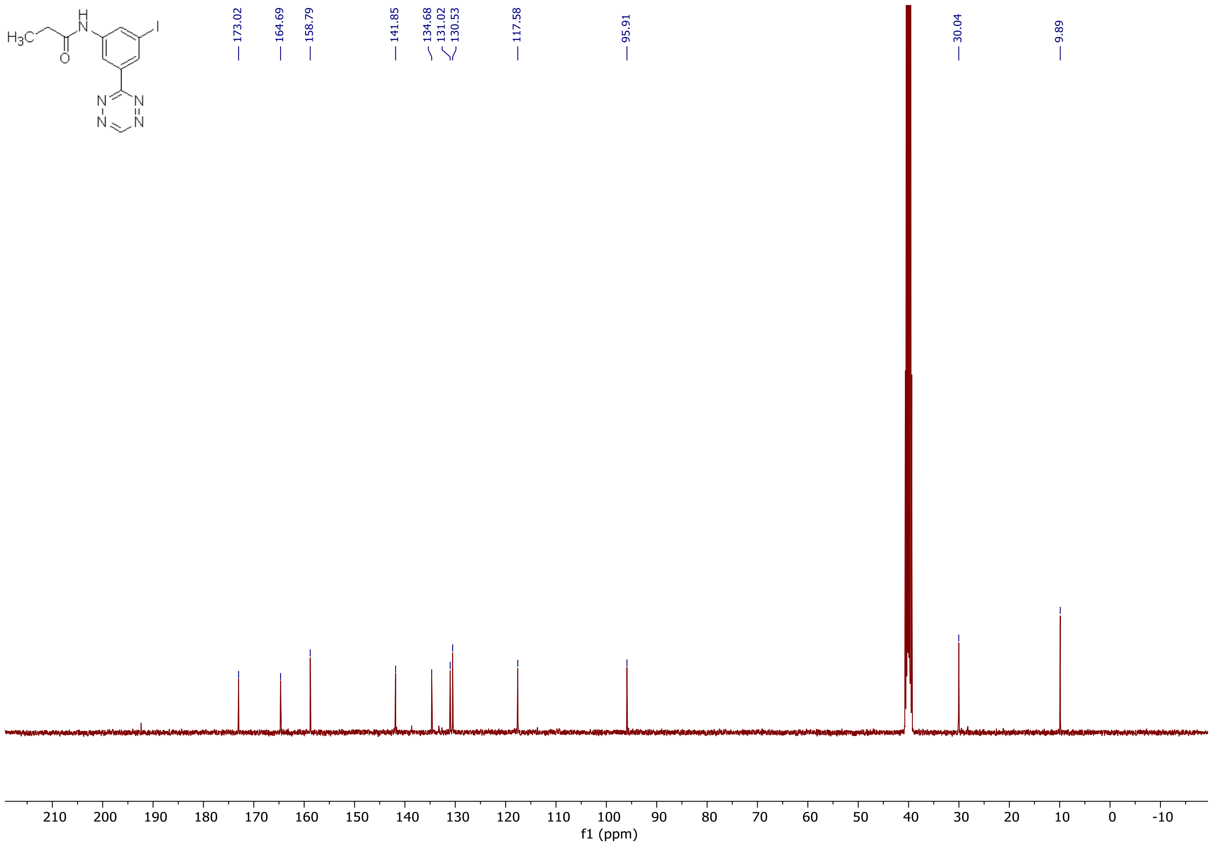

N-(3-(1,2,4,5-tetrazin-3-yl)-5-(trimethylstannyl)phenyl)propionamide

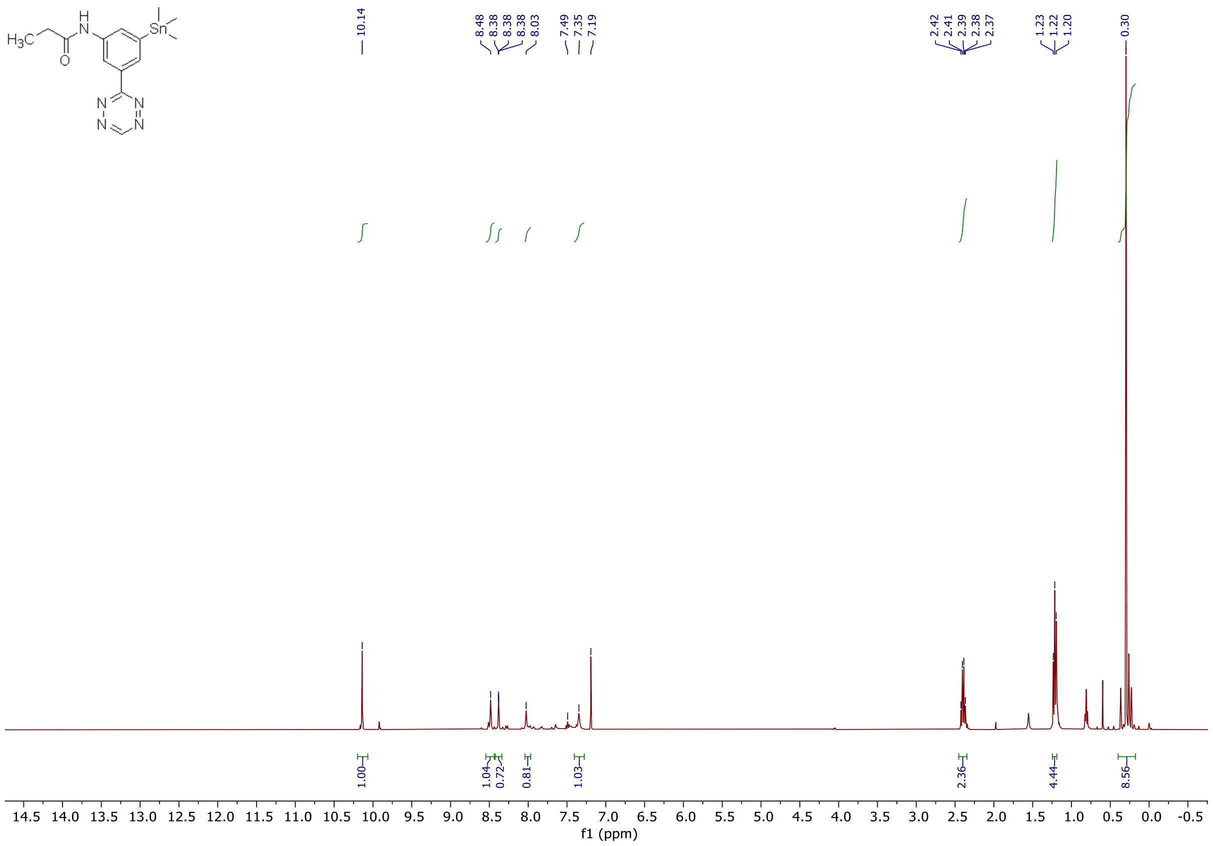

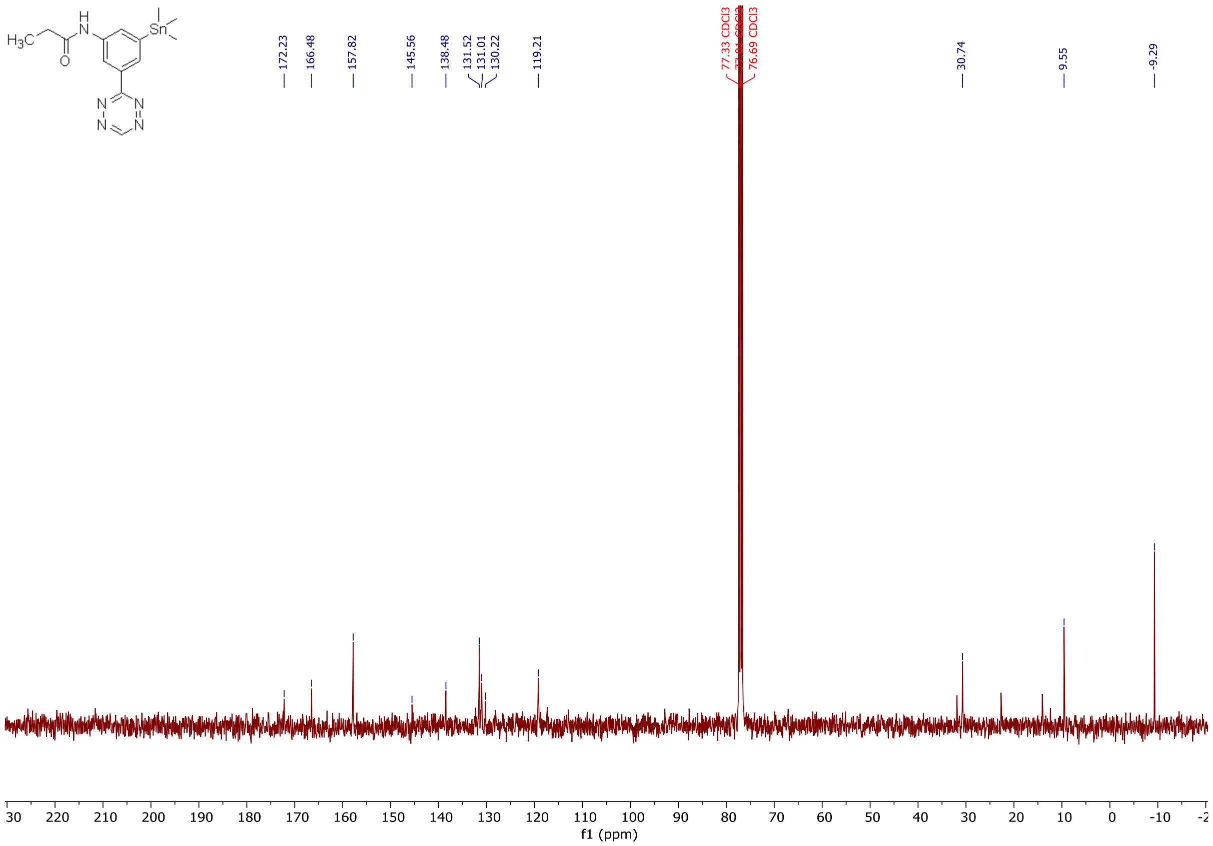

N-(3-Cyano-5-iodophenyl)butyramide

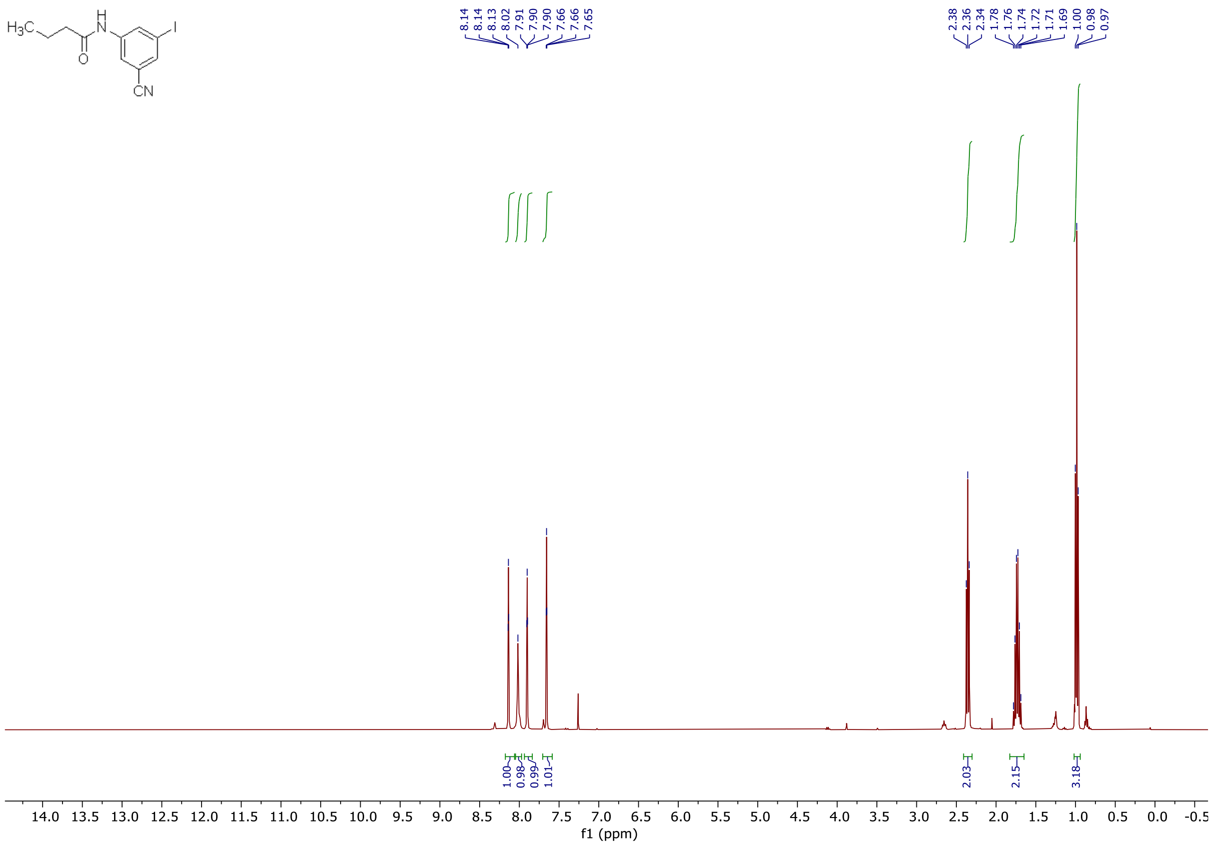

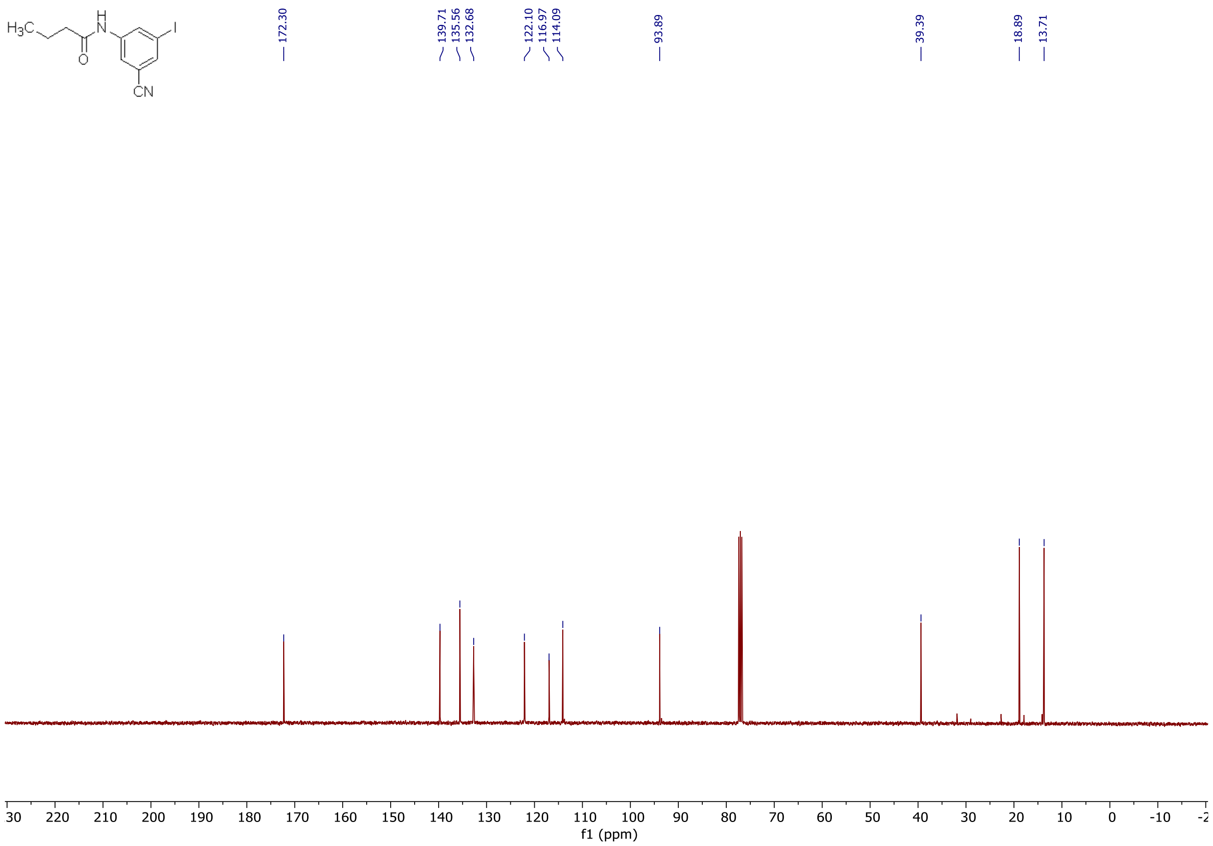

N-(3-Iodo-5-(1,2,4,5-tetrazin-3-yl)phenyl)butyramide

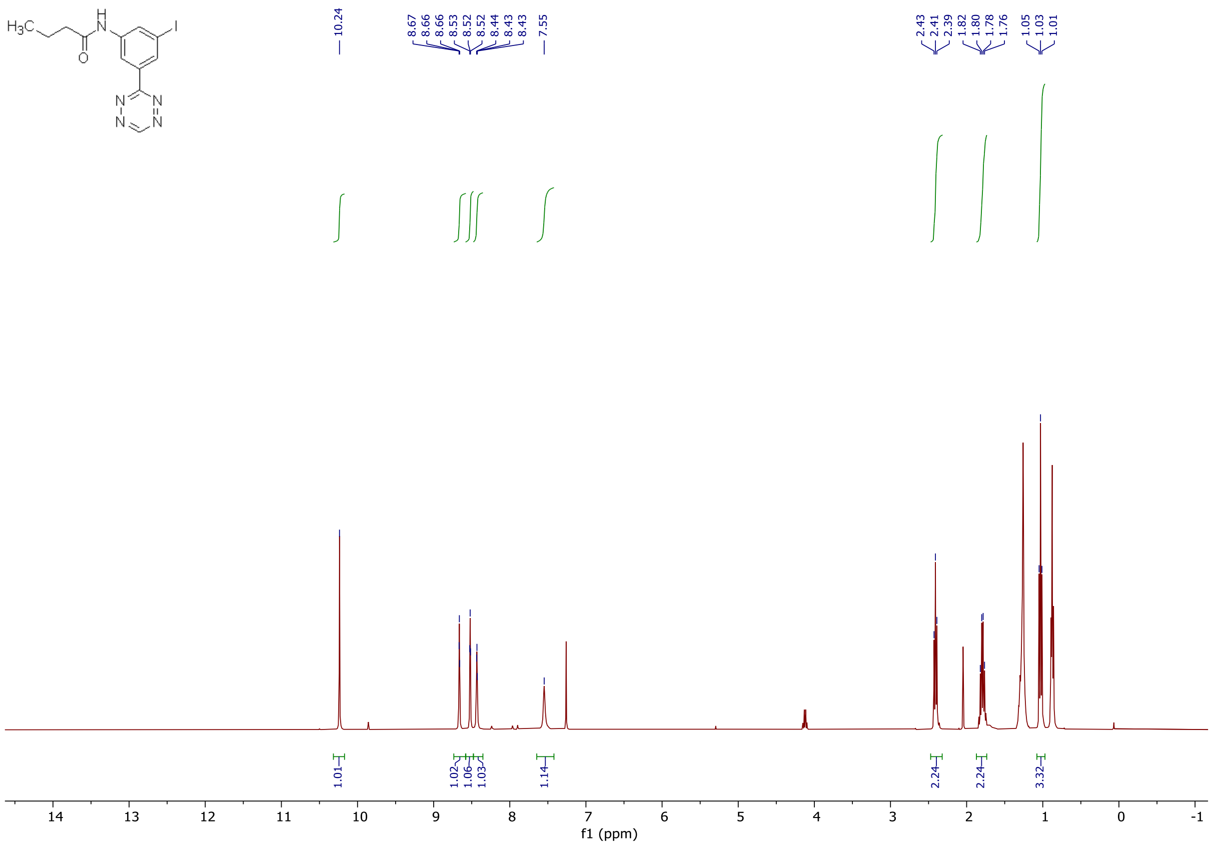

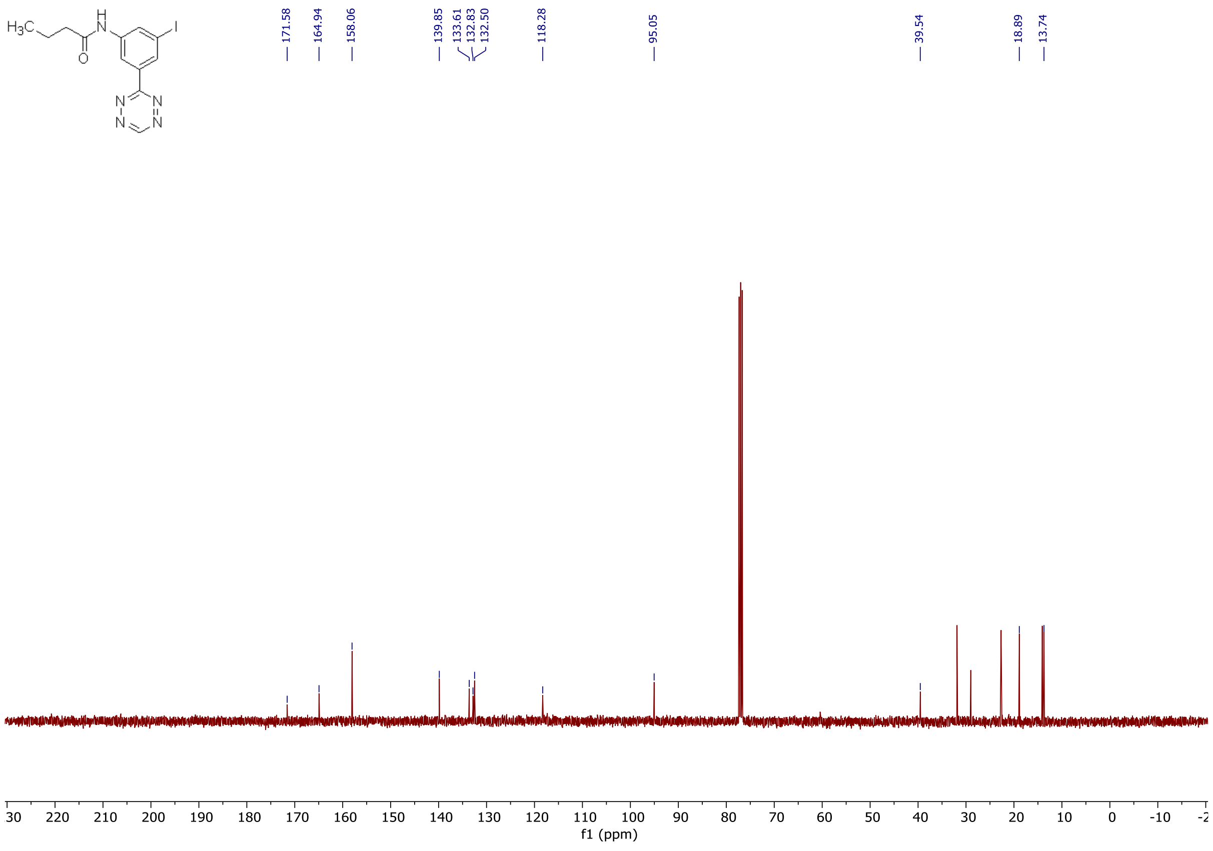

N-(3-(1,2,4,5-Tetrazin-3-yl)-5-(trimethylstannyl)phenyl)butyramide

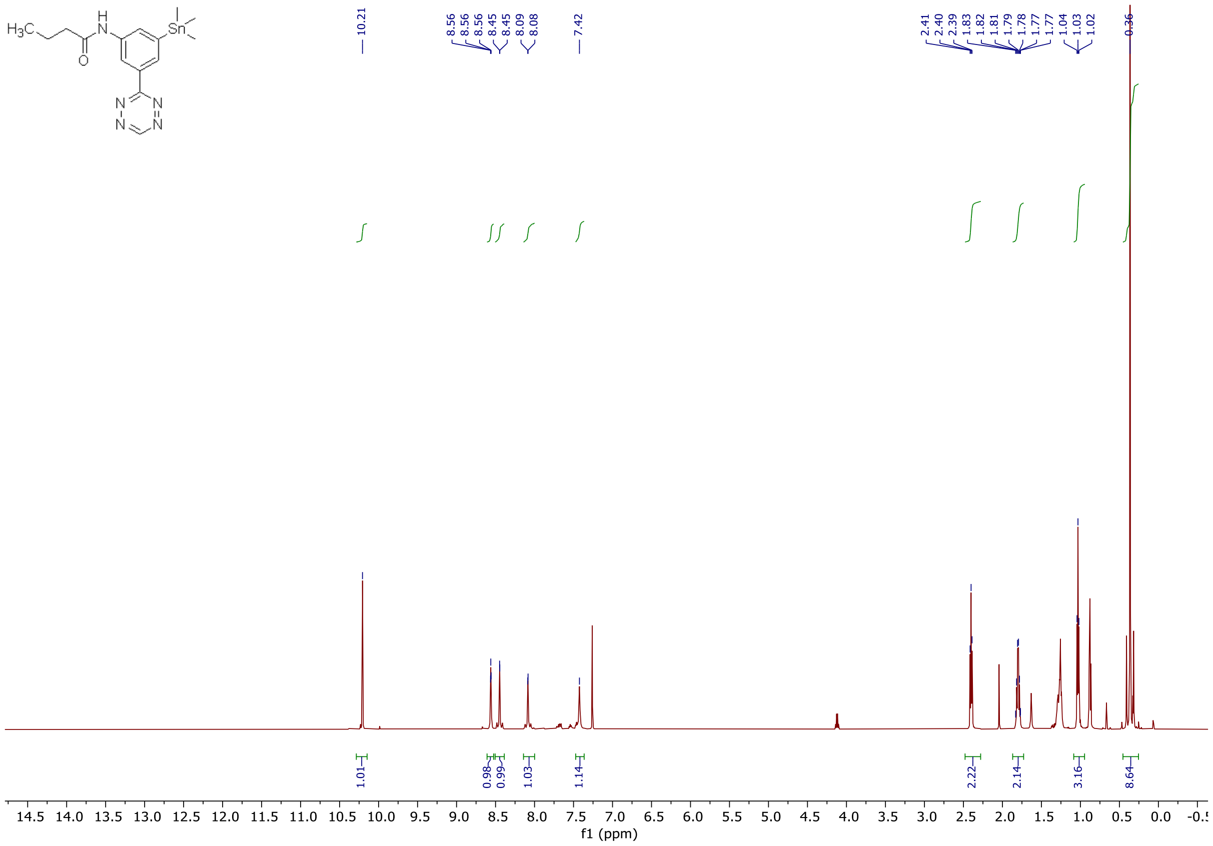

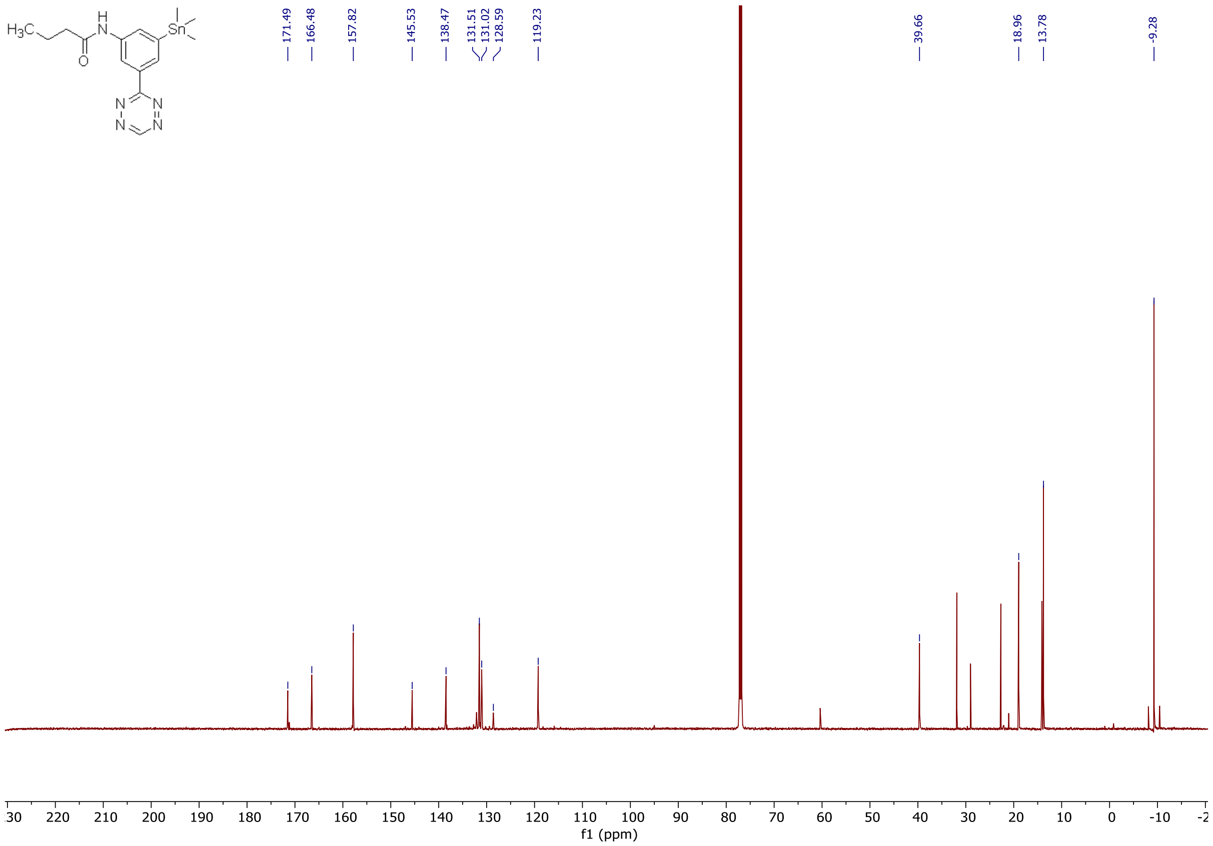

N-(3-Cyano-5-iodophenyl)pentanamide

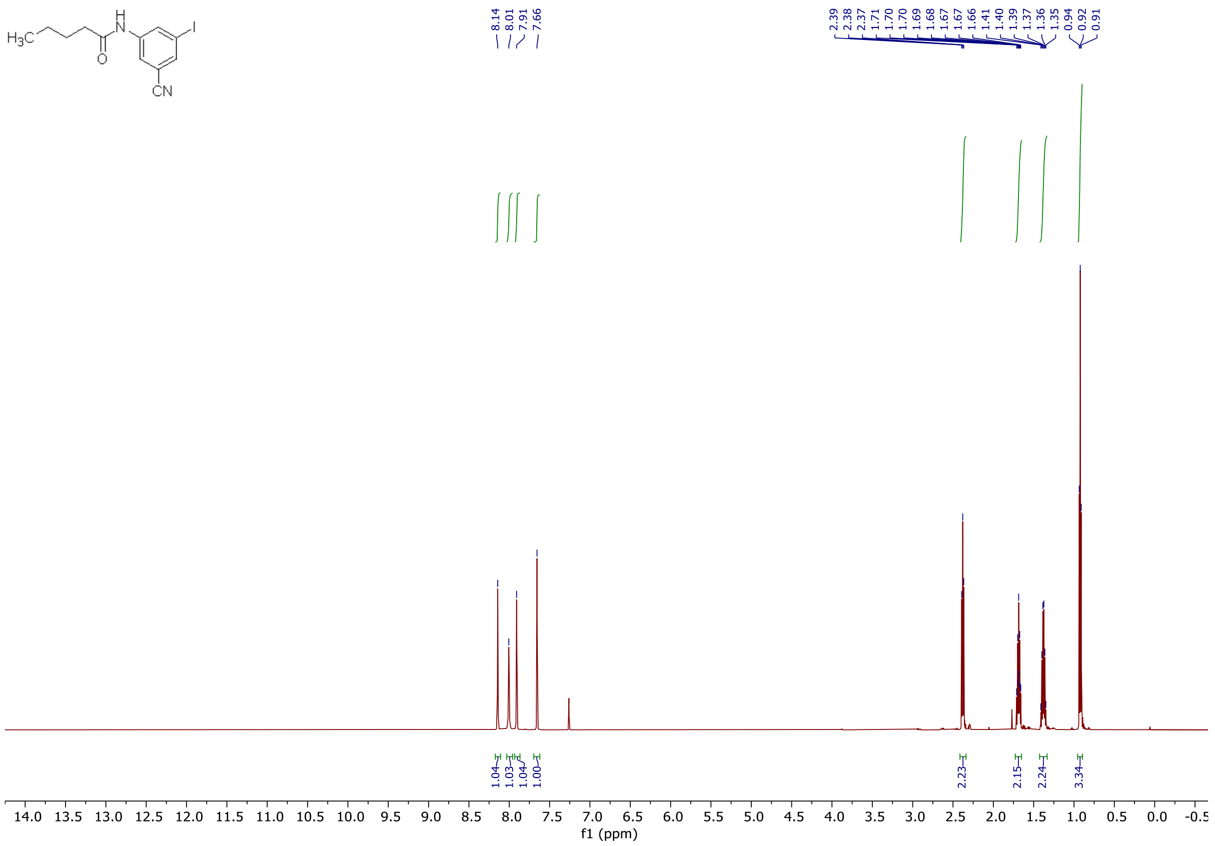

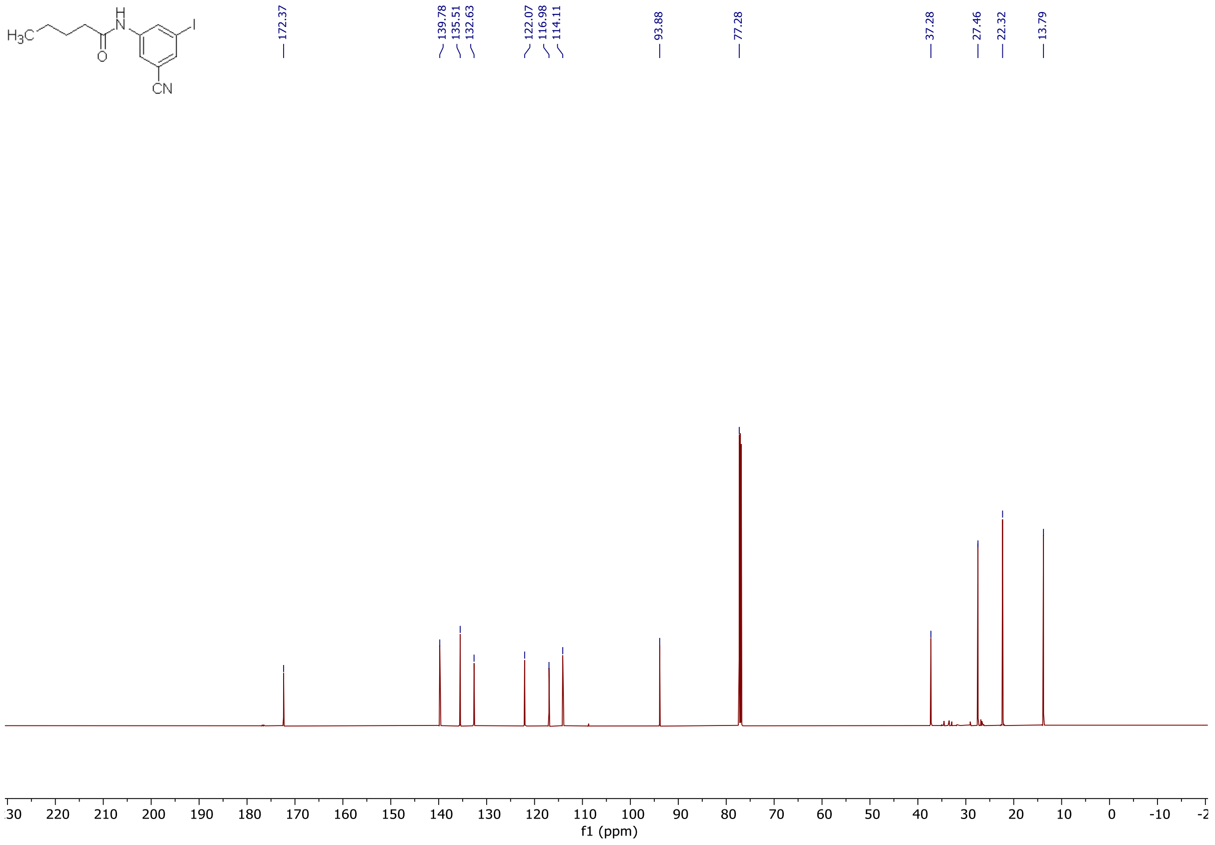

N-(3-Iodo-5-(1,2,4,5-tetrazin-3-yl)phenyl)pentanamide

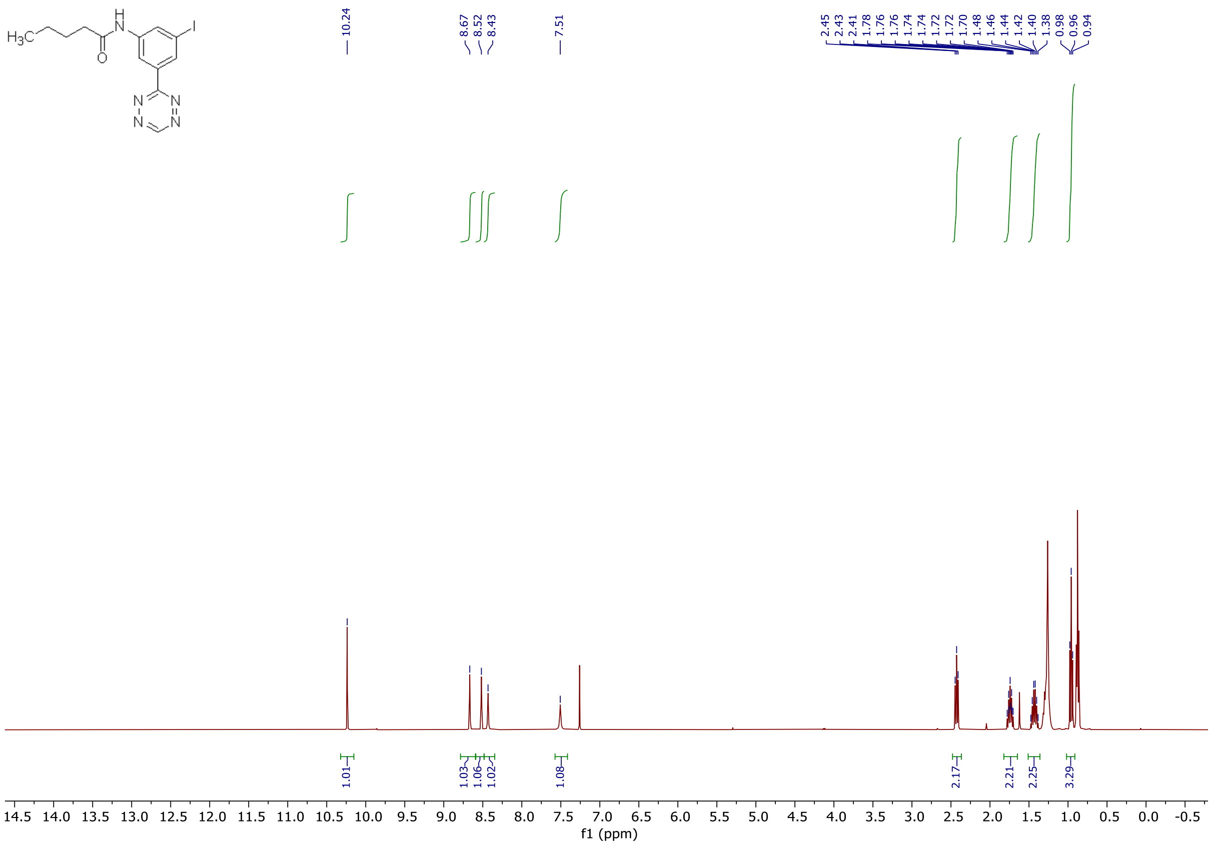

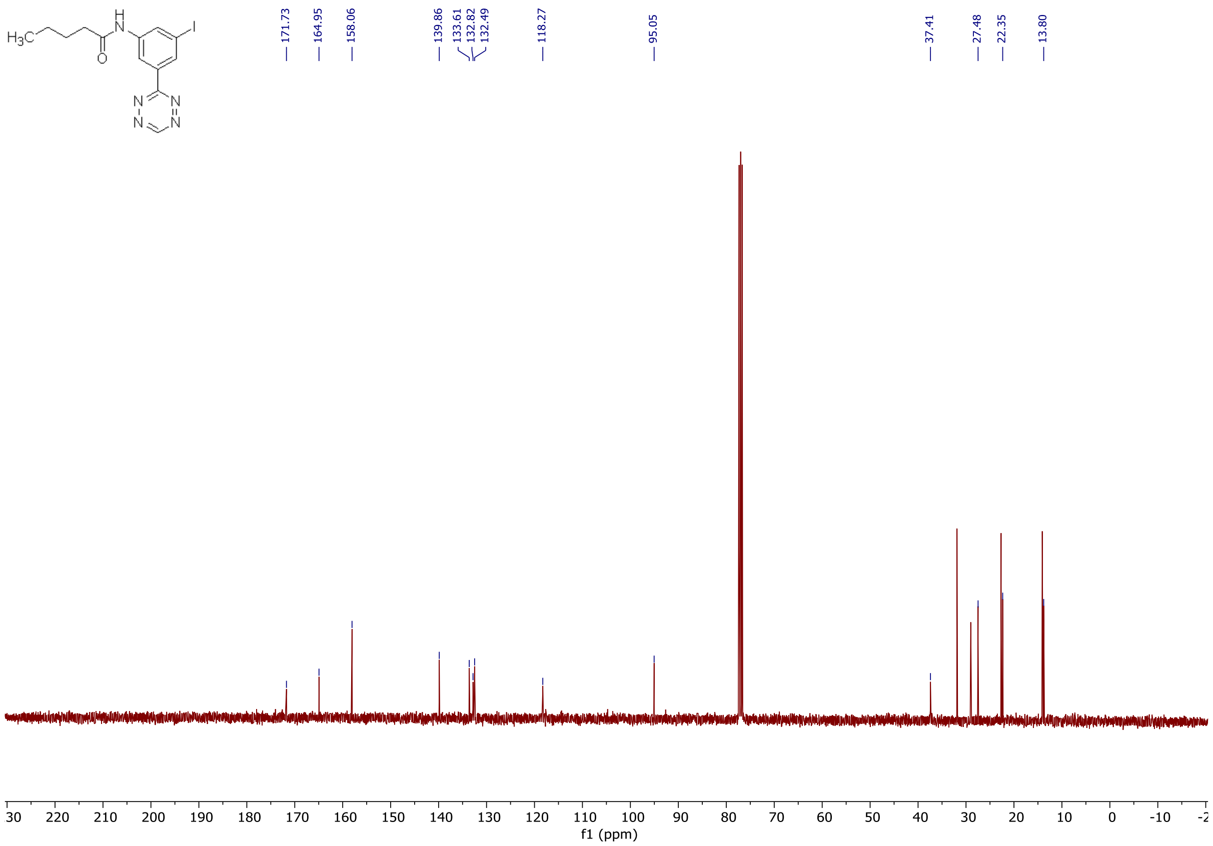

N-(3-(1,2,4,5-Tetrazin-3-yl)-5-(trimethylstannyl)phenyl)pentanamide

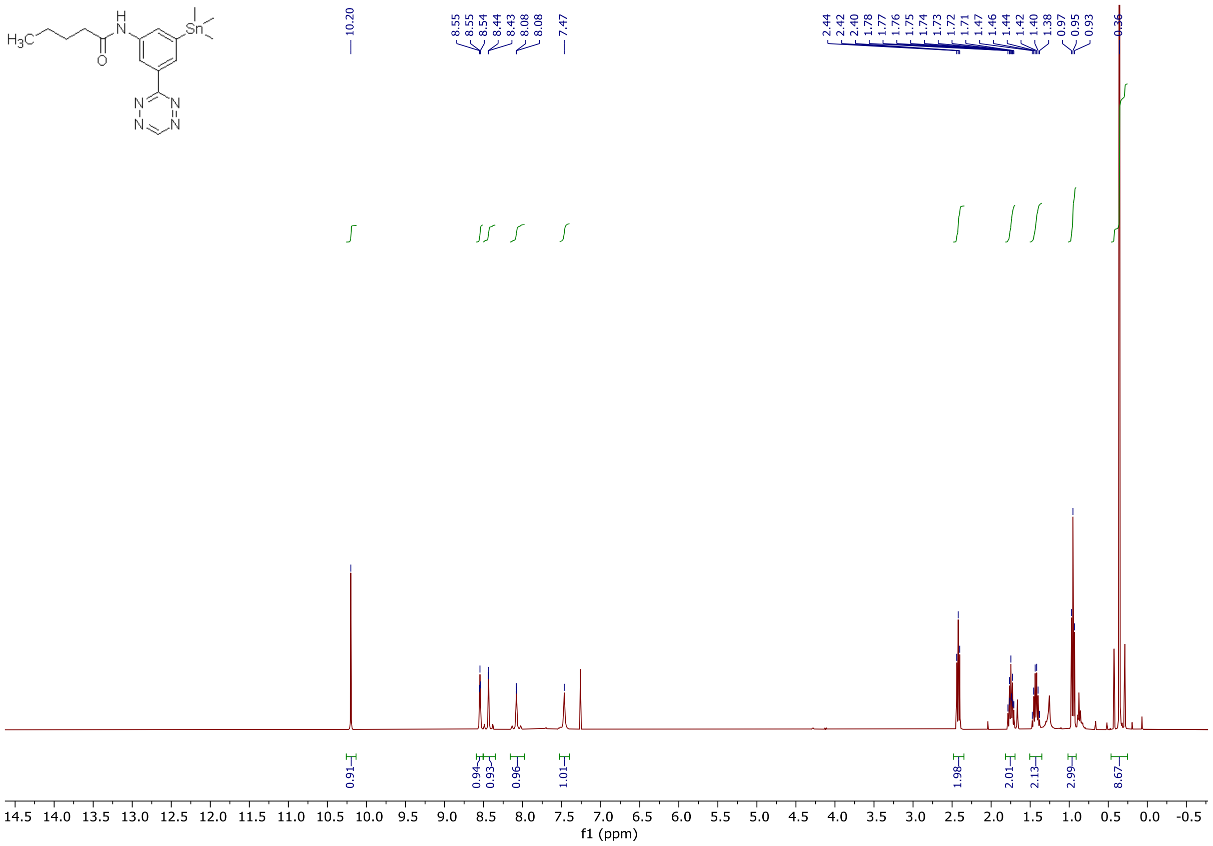

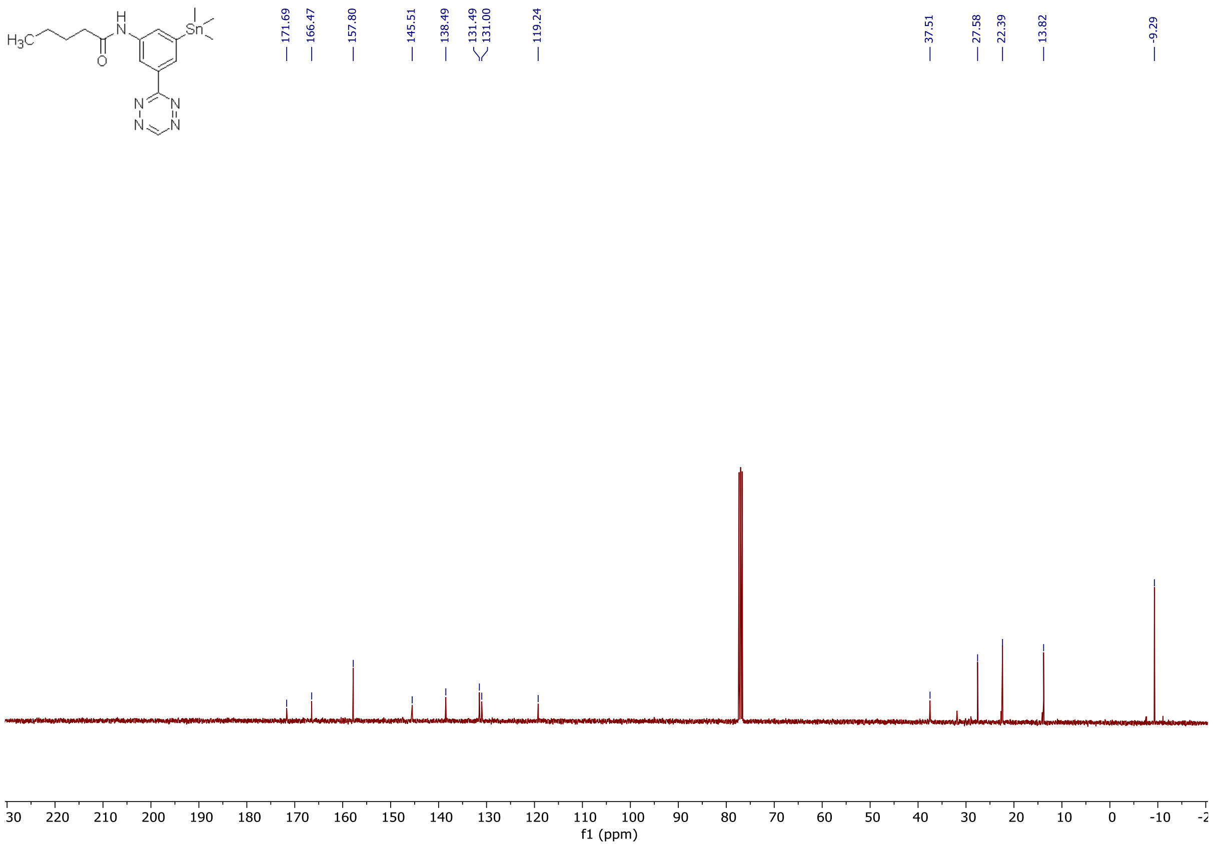

3-Cyano-N-ethyl-5-iodobenzamide

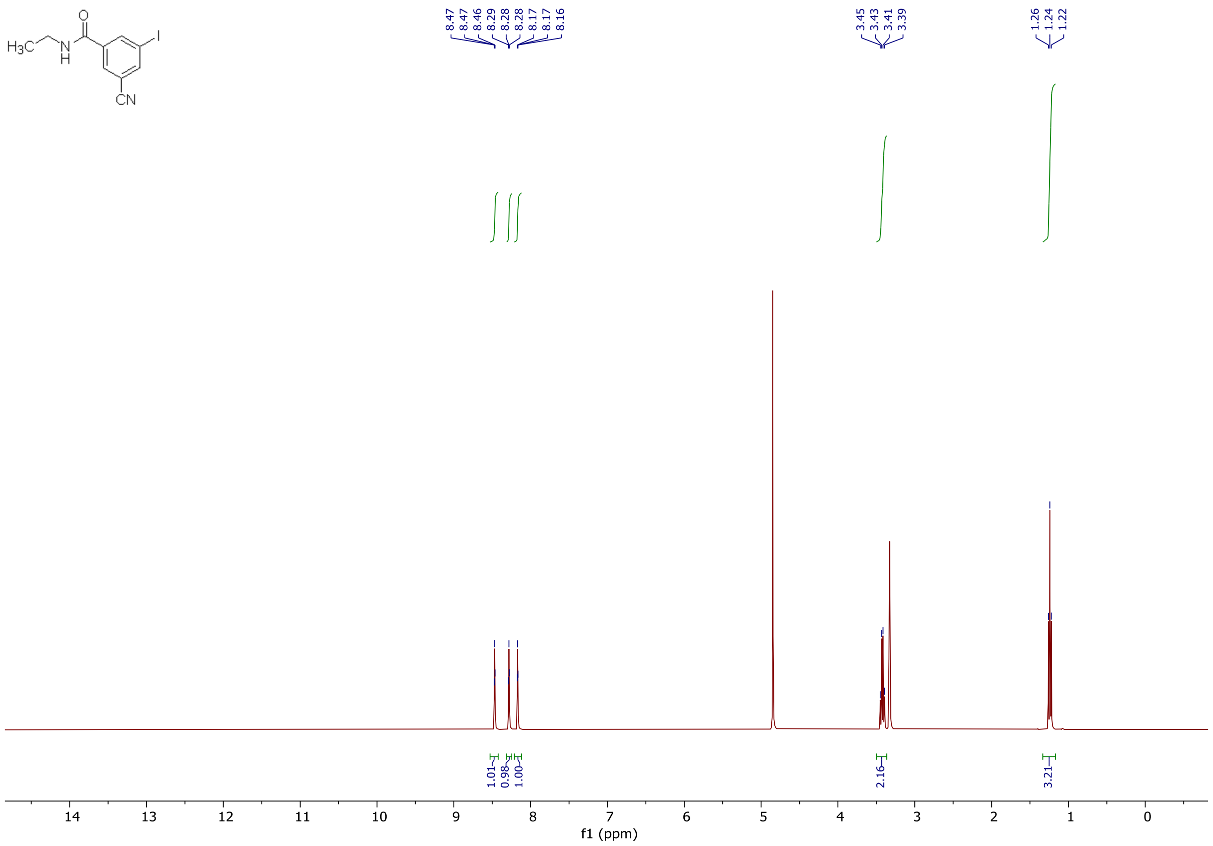

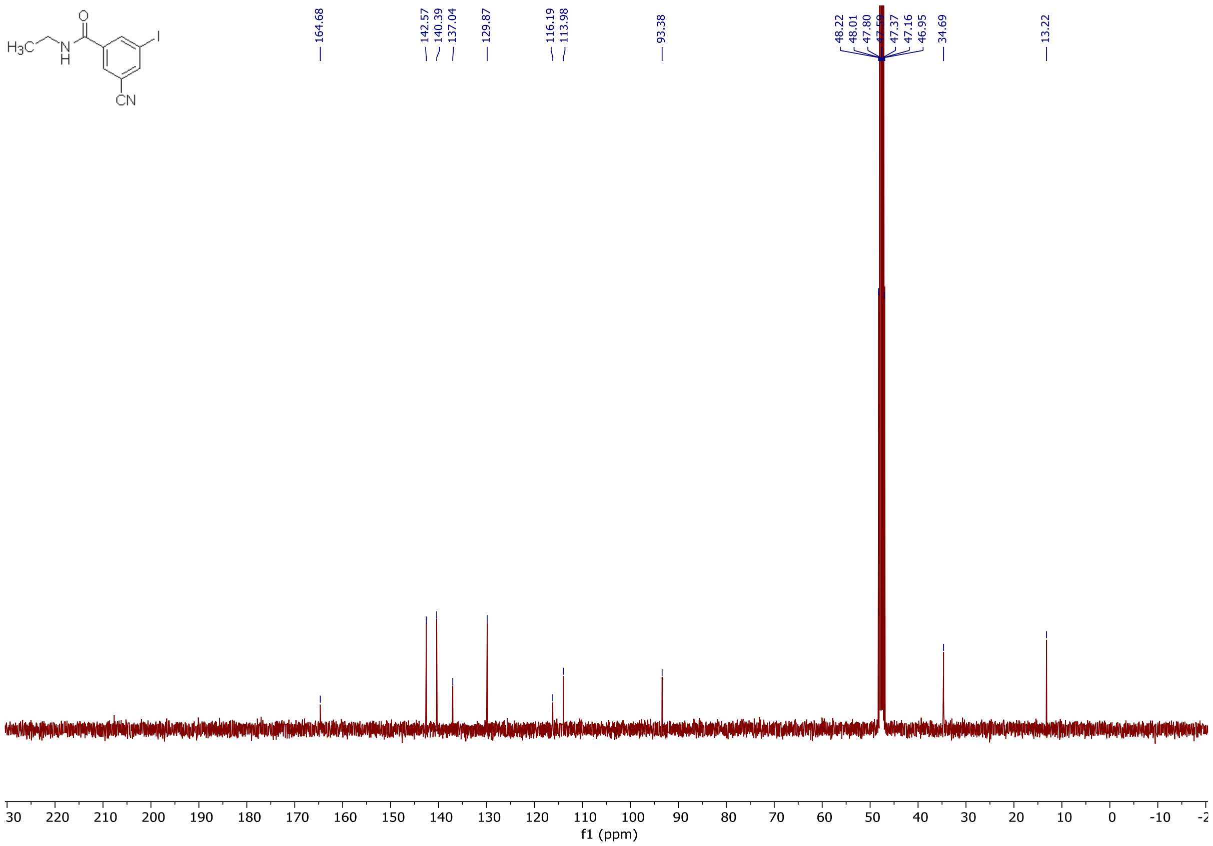

N-Ethyl-3-iodo-5-(1,2,4,5-tetrazin-3-yl)benzamide

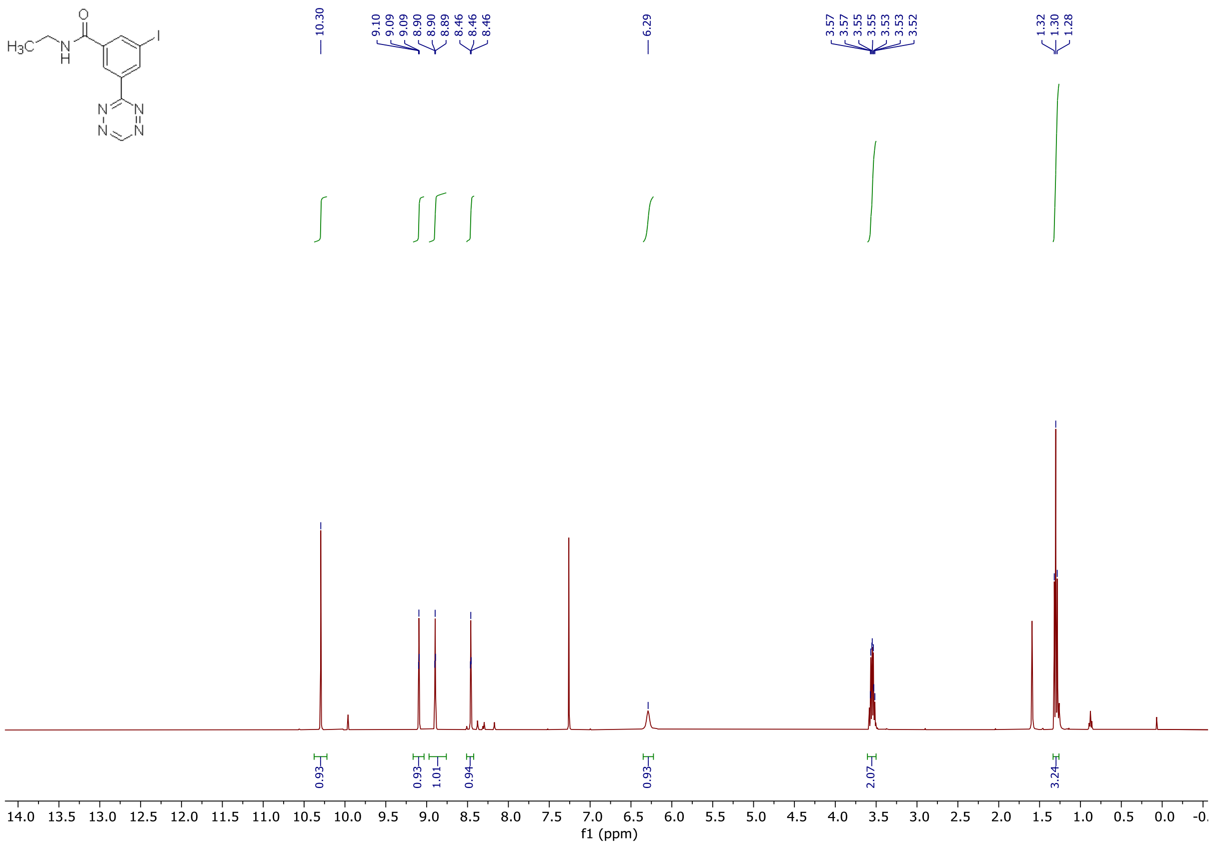

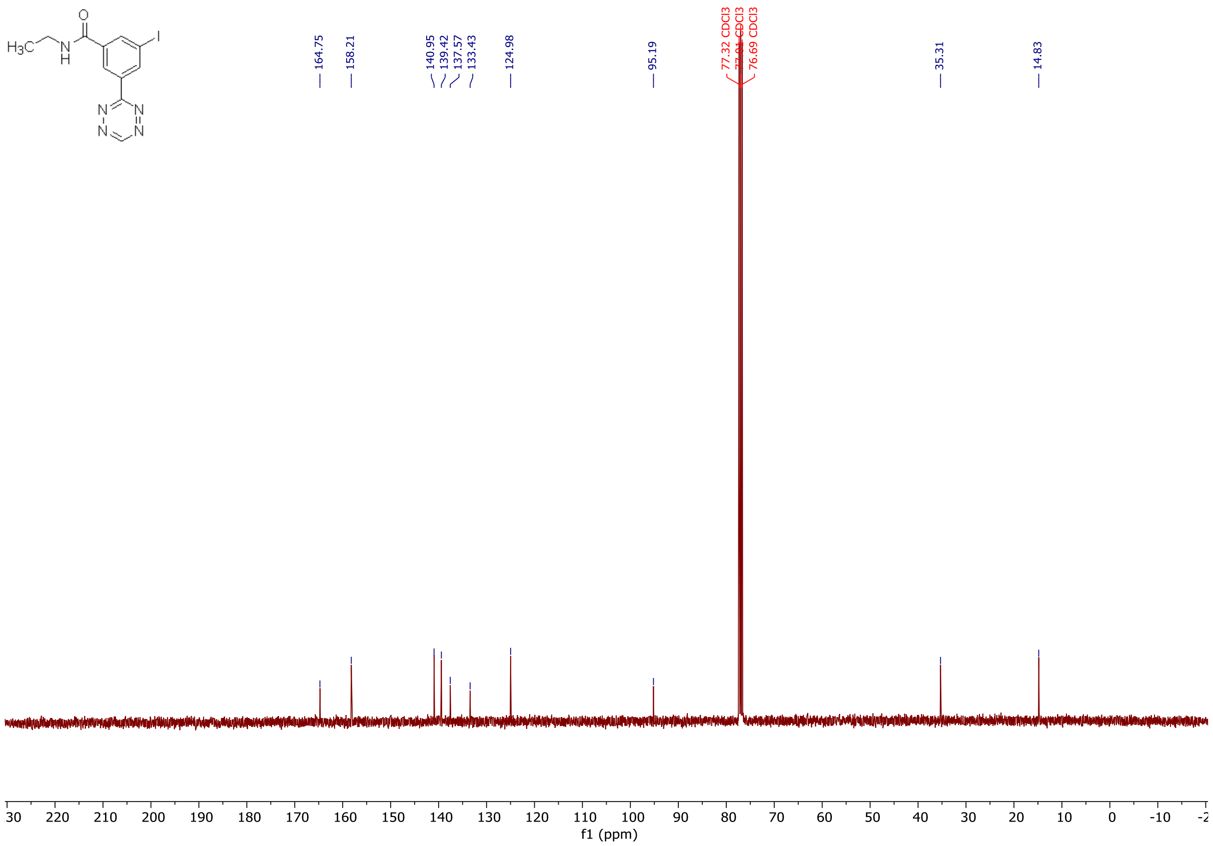

N-Ethyl-3-(1,2,4,5-tetrazin-3-yl)-5-(trimethylstannyl)benzamide

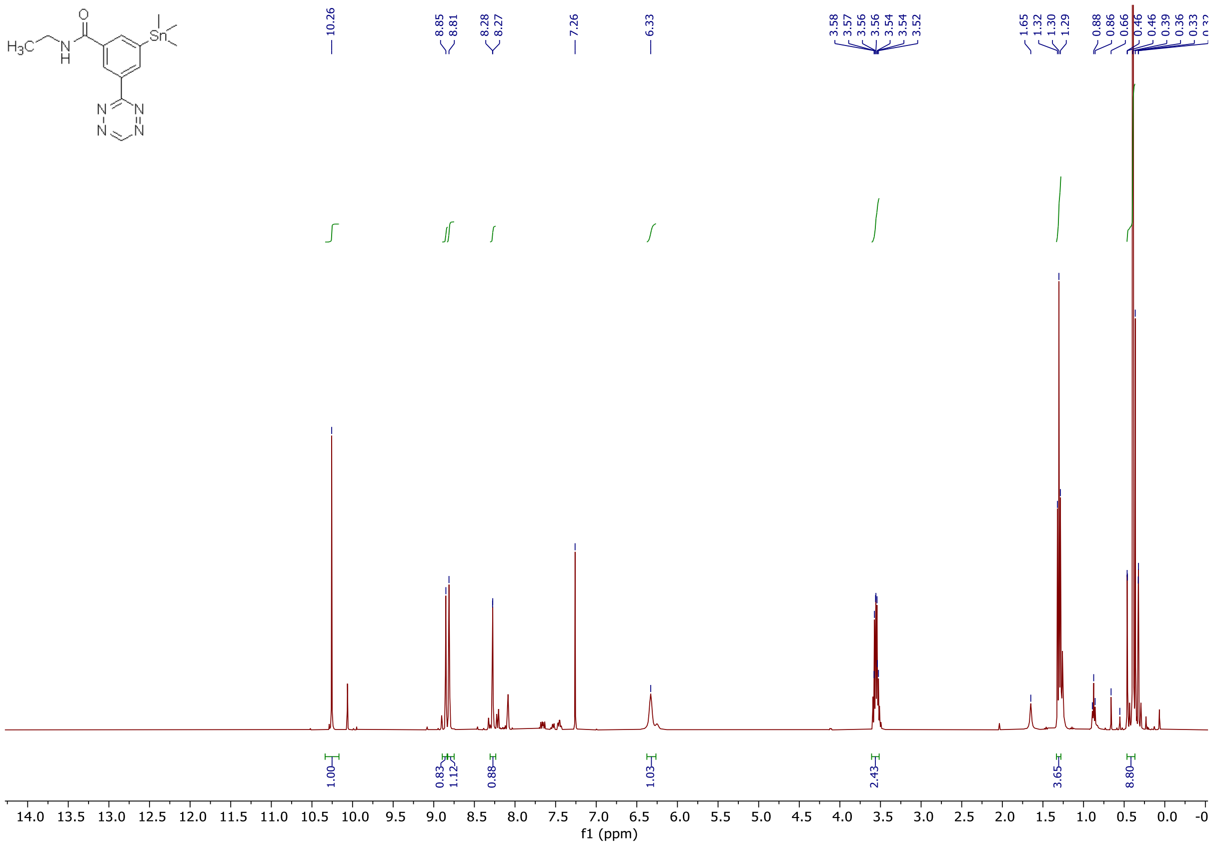

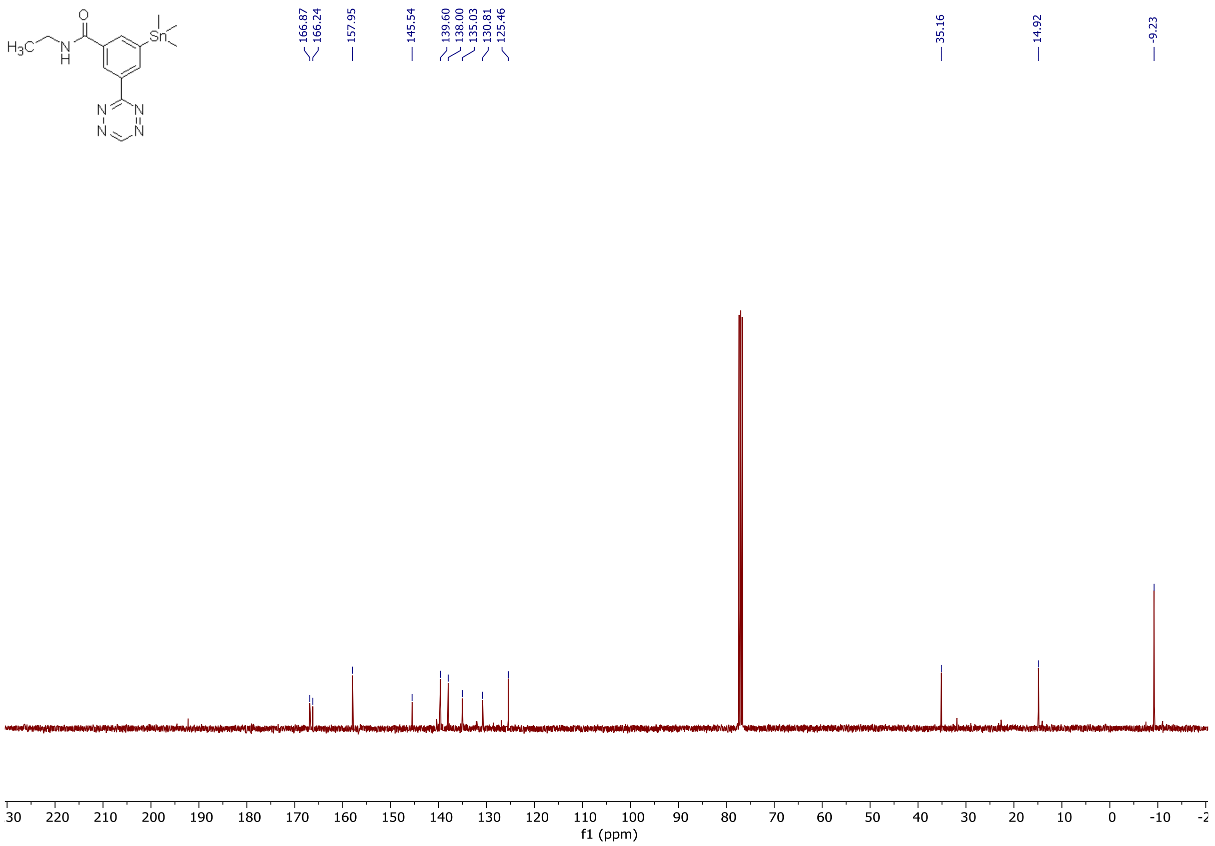

3-Cyano-5-iodo-N-propylbenzamide

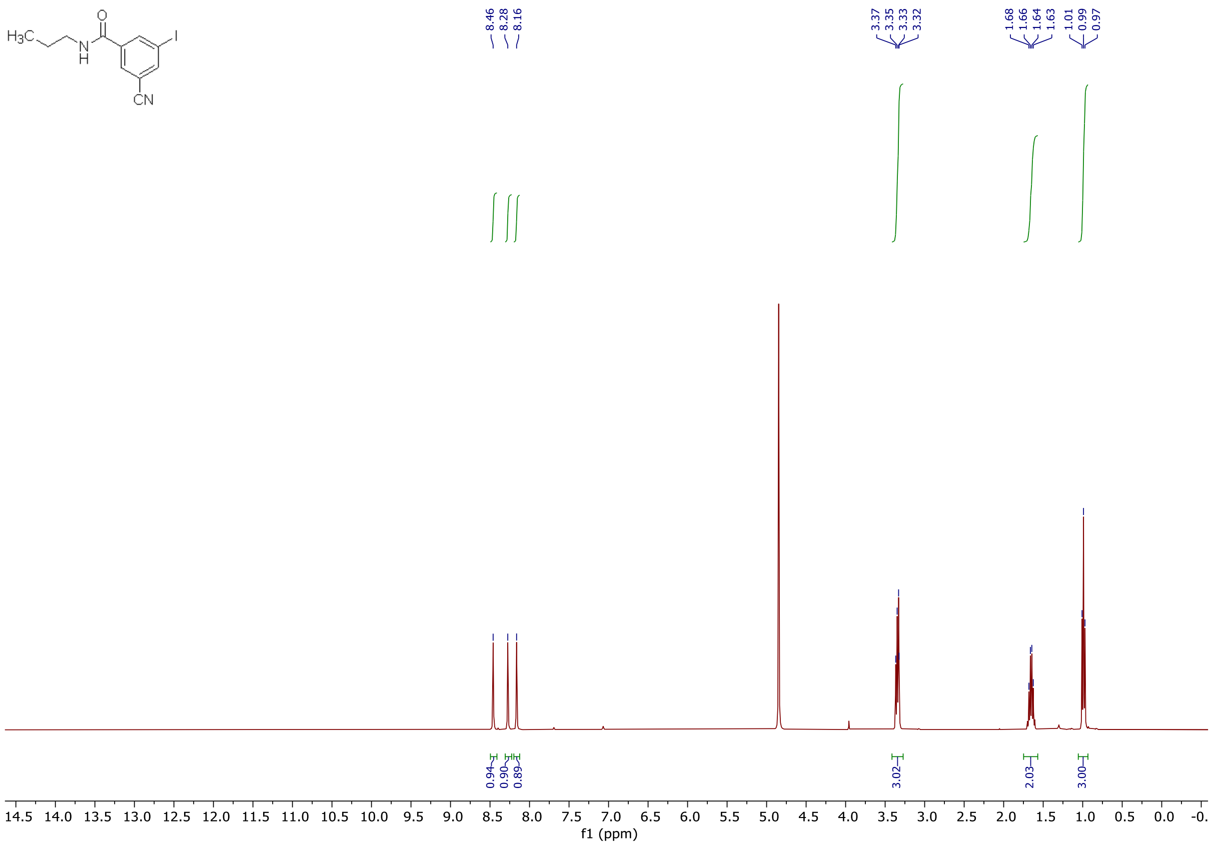

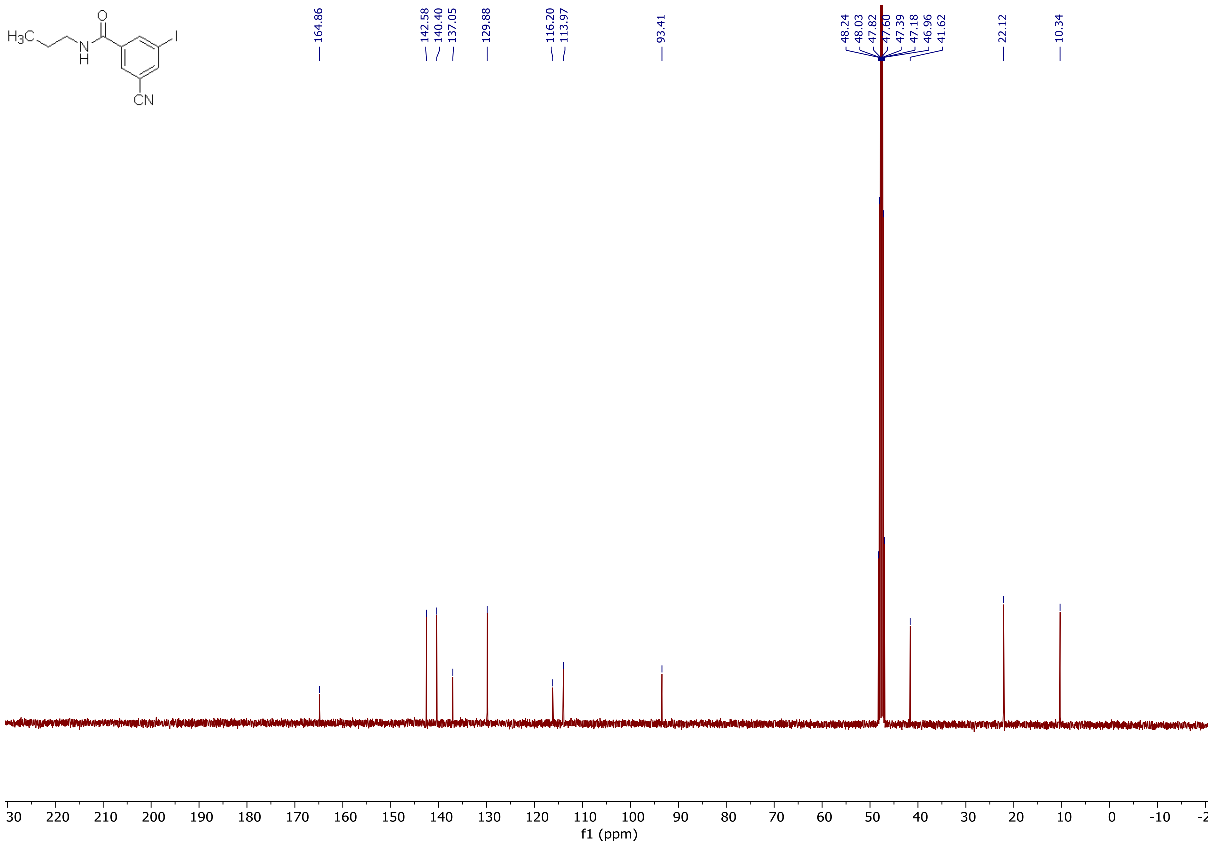

3-Iodo-N-propyl-5-(1,2,4,5-tetrazin-3-yl)benzamide

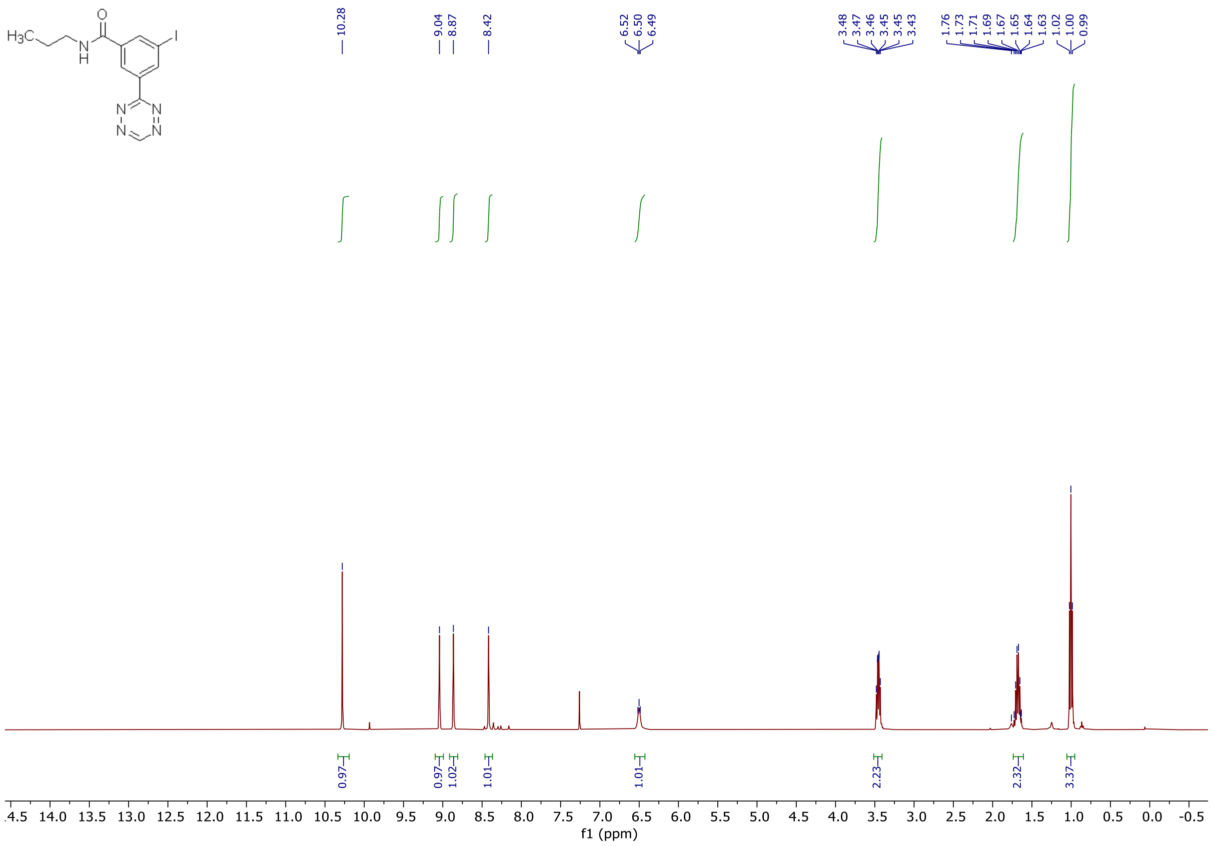

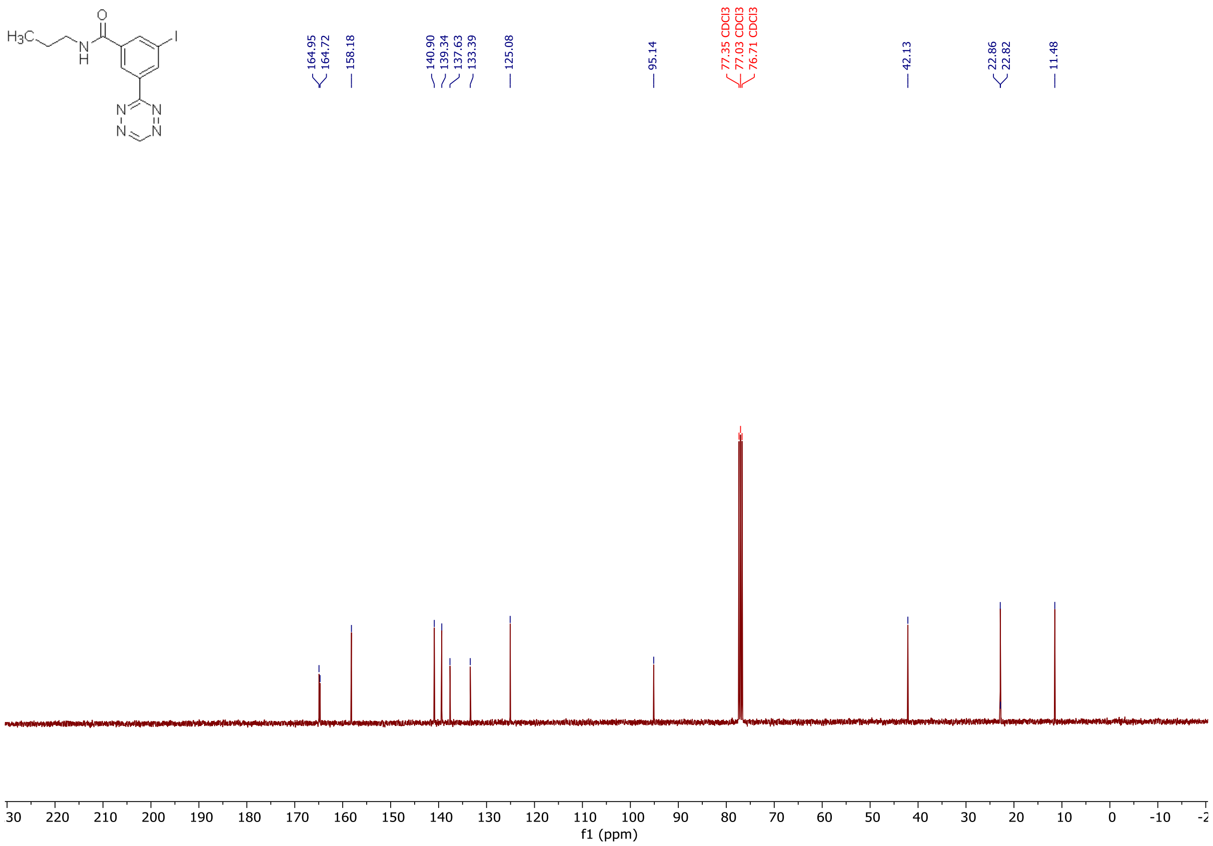

N-Propyl-3-(1,2,4,5-tetrazin-3-yl)-5-(trimethylstannyl)benzamide

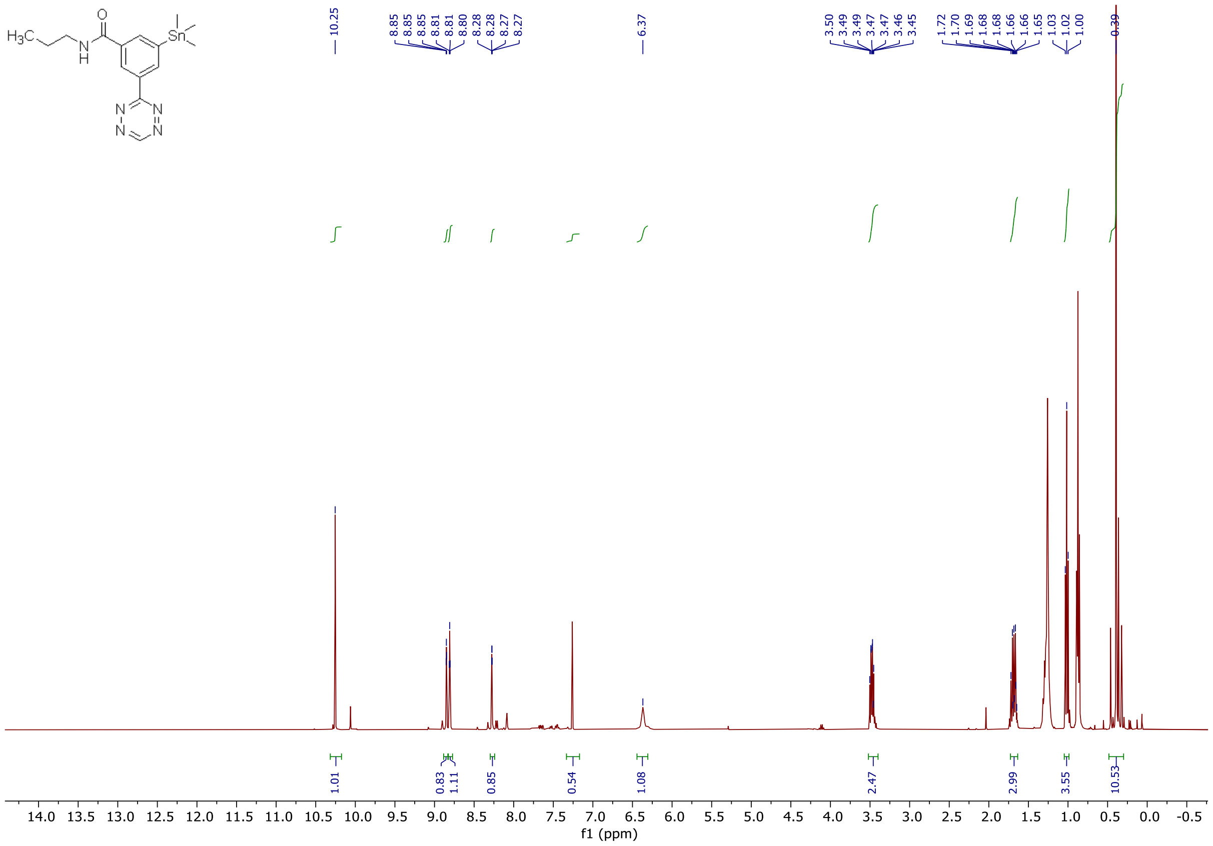

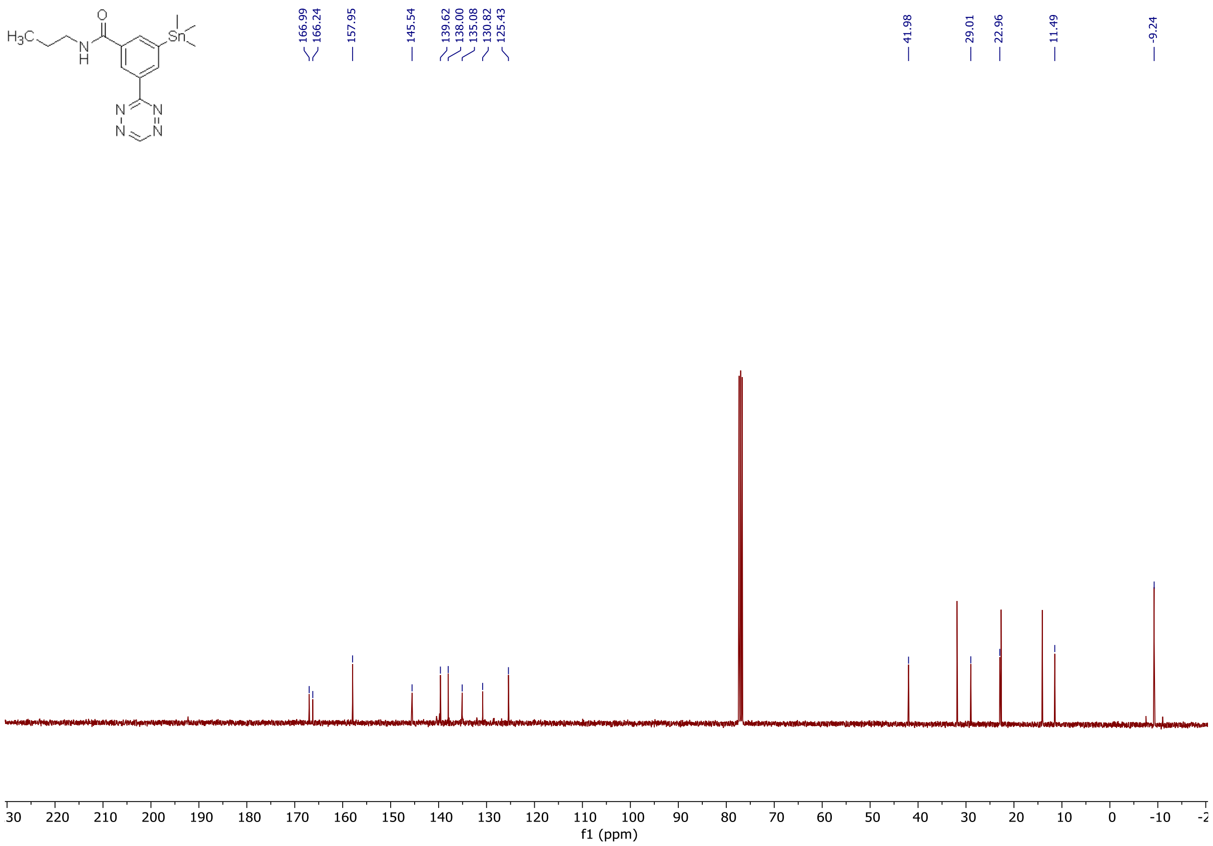

3-(Aminomethyl)-5-iodobenzonitrile hydrochloride

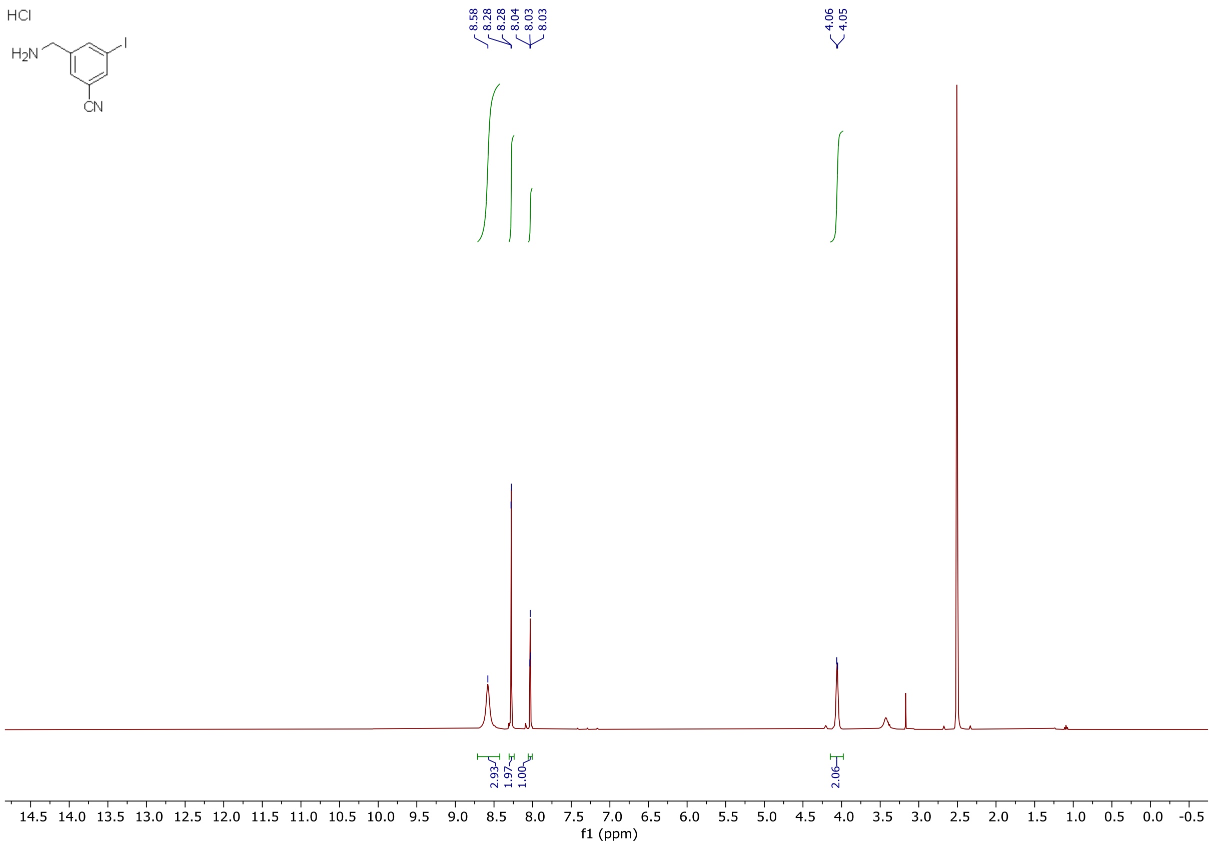

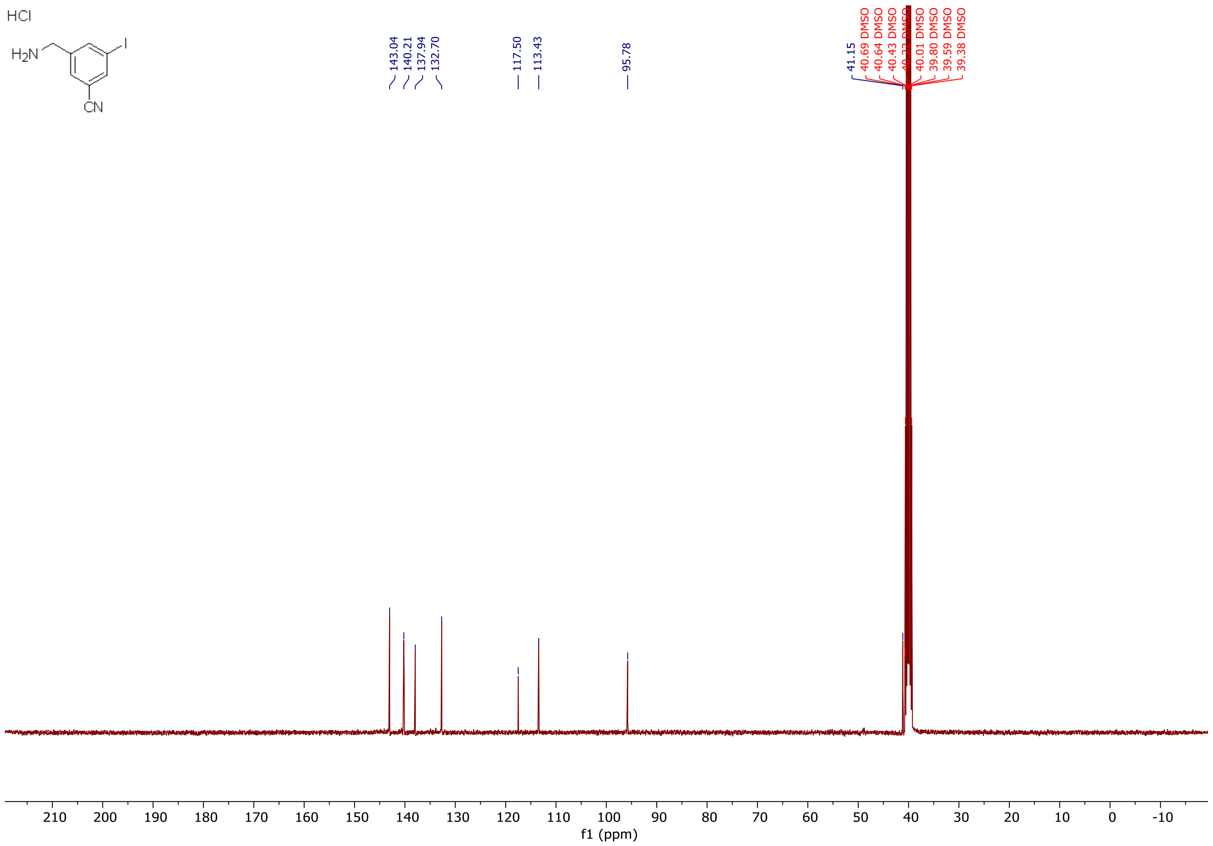

***Tert*-butyl 3-cyano-5-iodobenzylcarbamate**

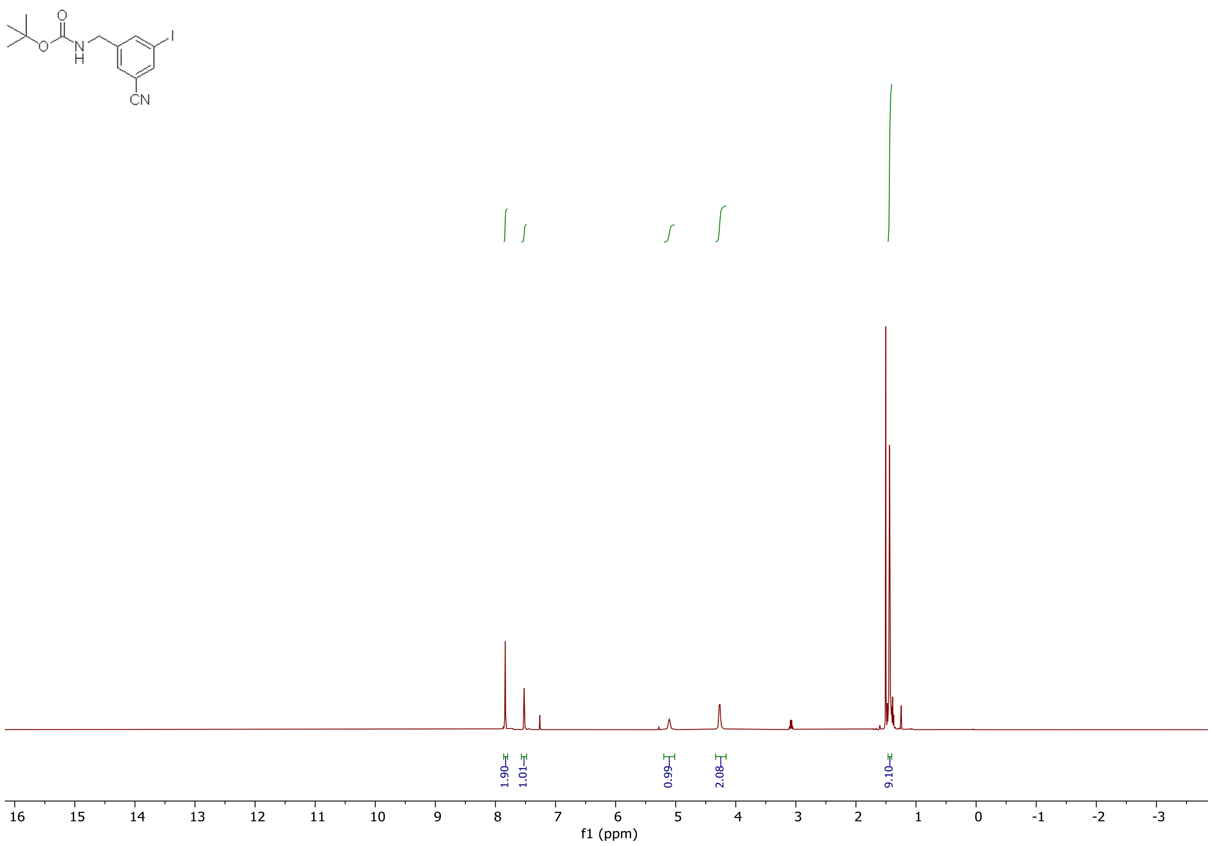

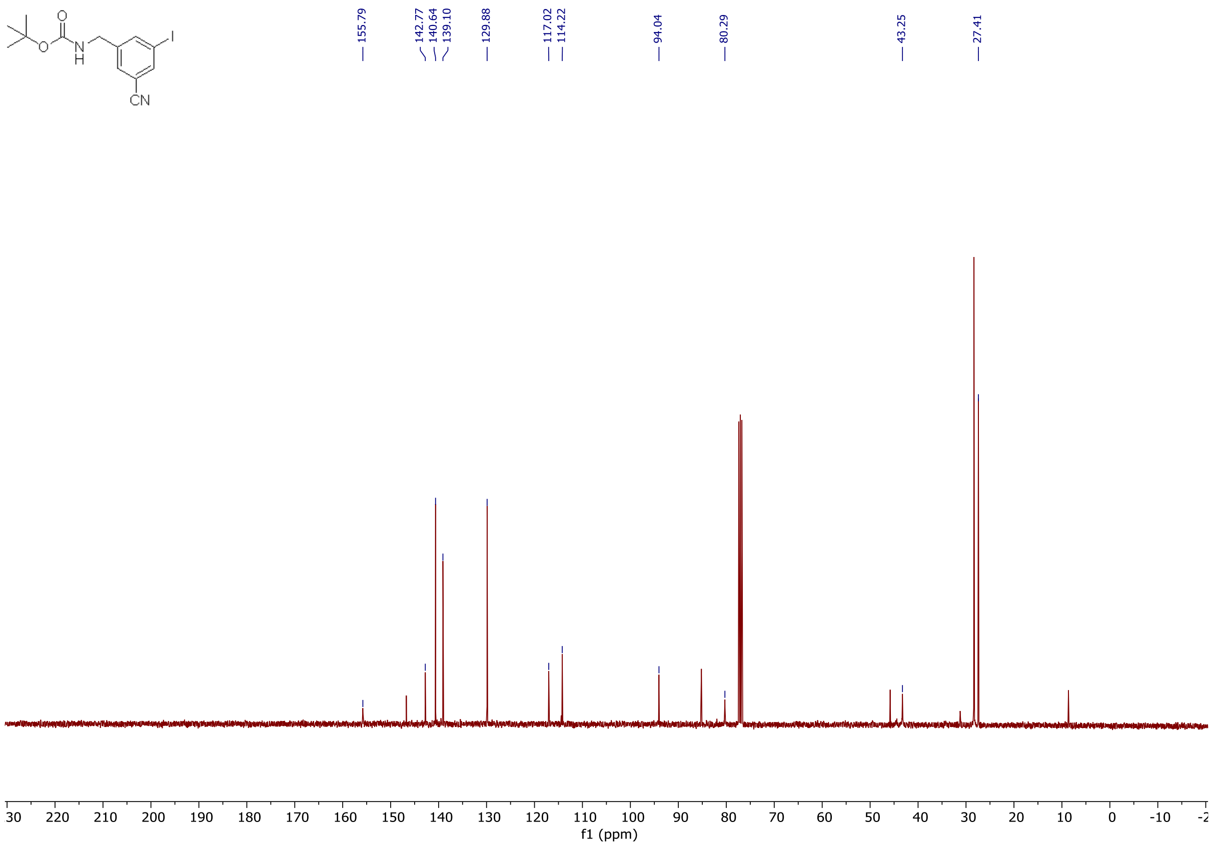

Tert-butyl 3-iodo-5-(1,2,4,5-tetrazin-3-yl)benzylcarbamate

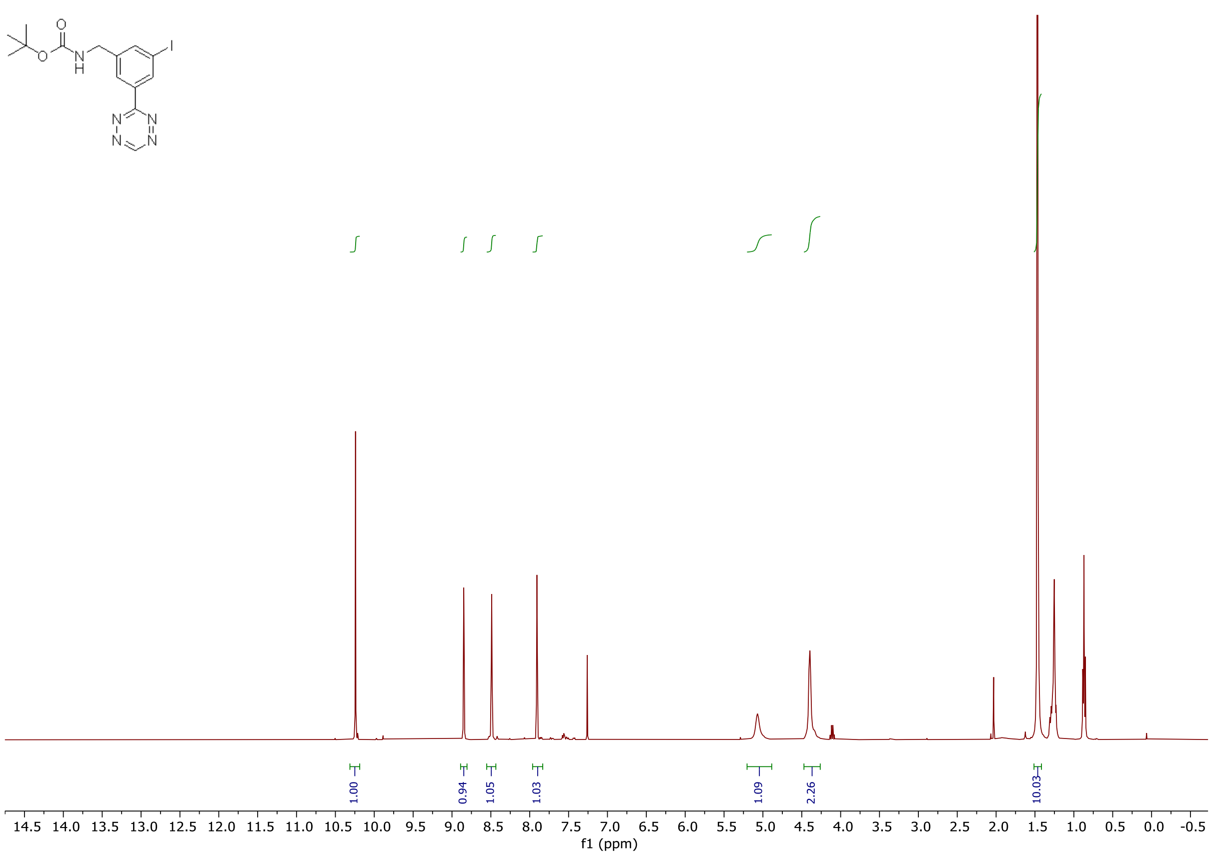

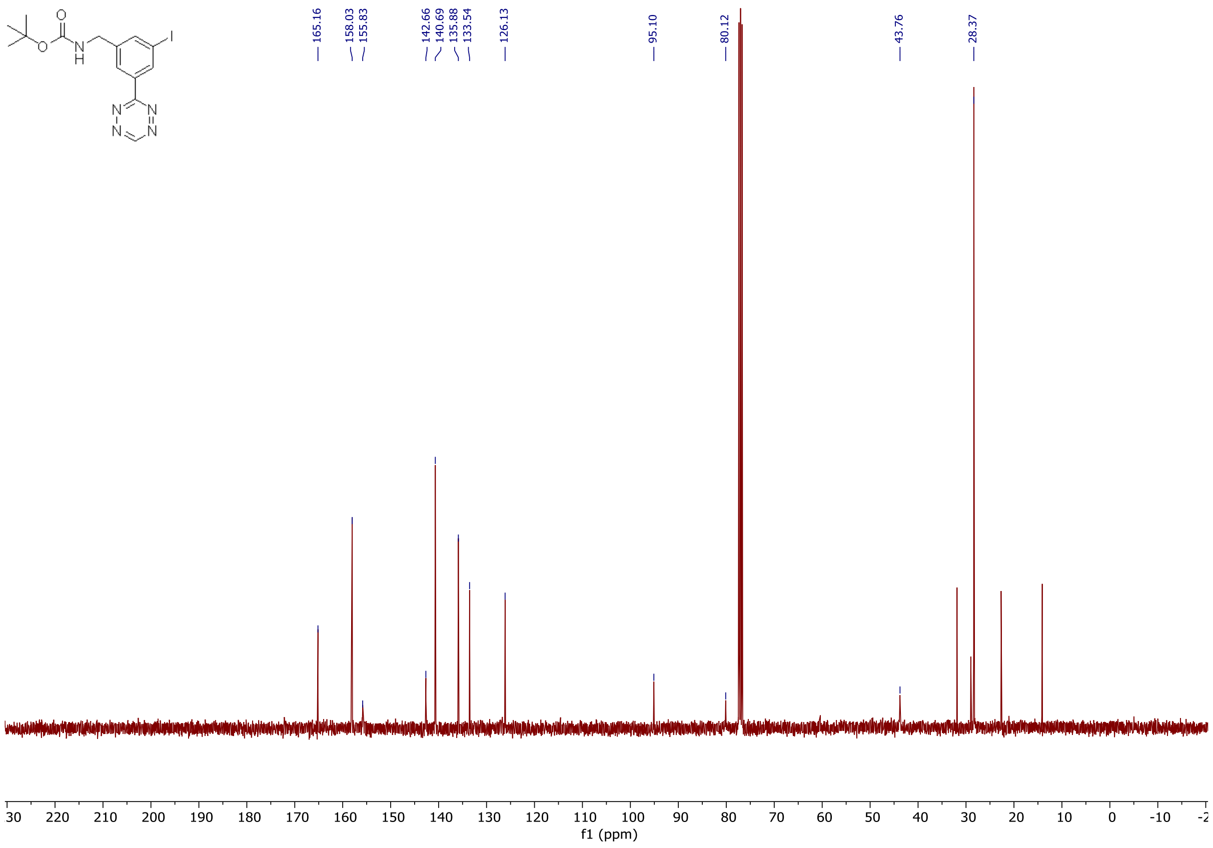

***Tert*-butyl 3-(1,2,4,5-tetrazin-3-yl)-5-(trimethylstannyl)benzylcarbamate**

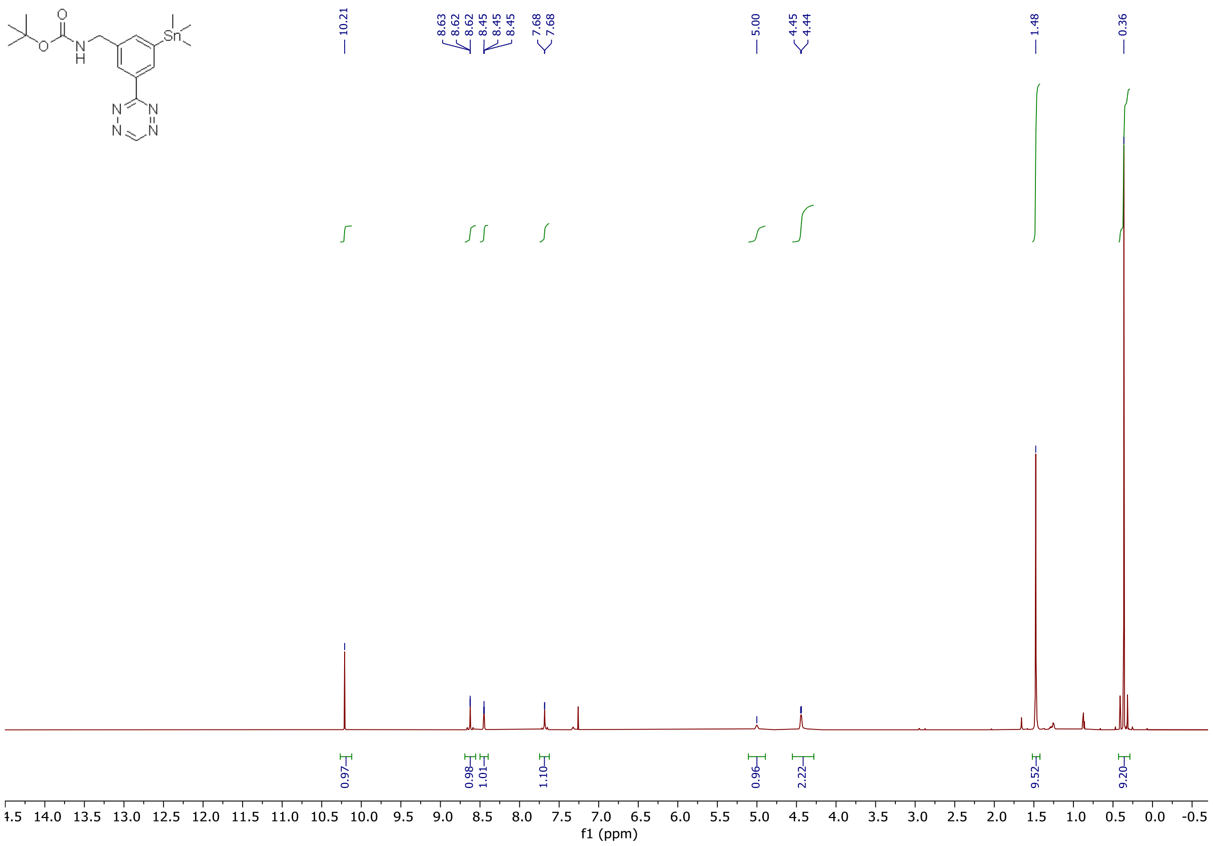

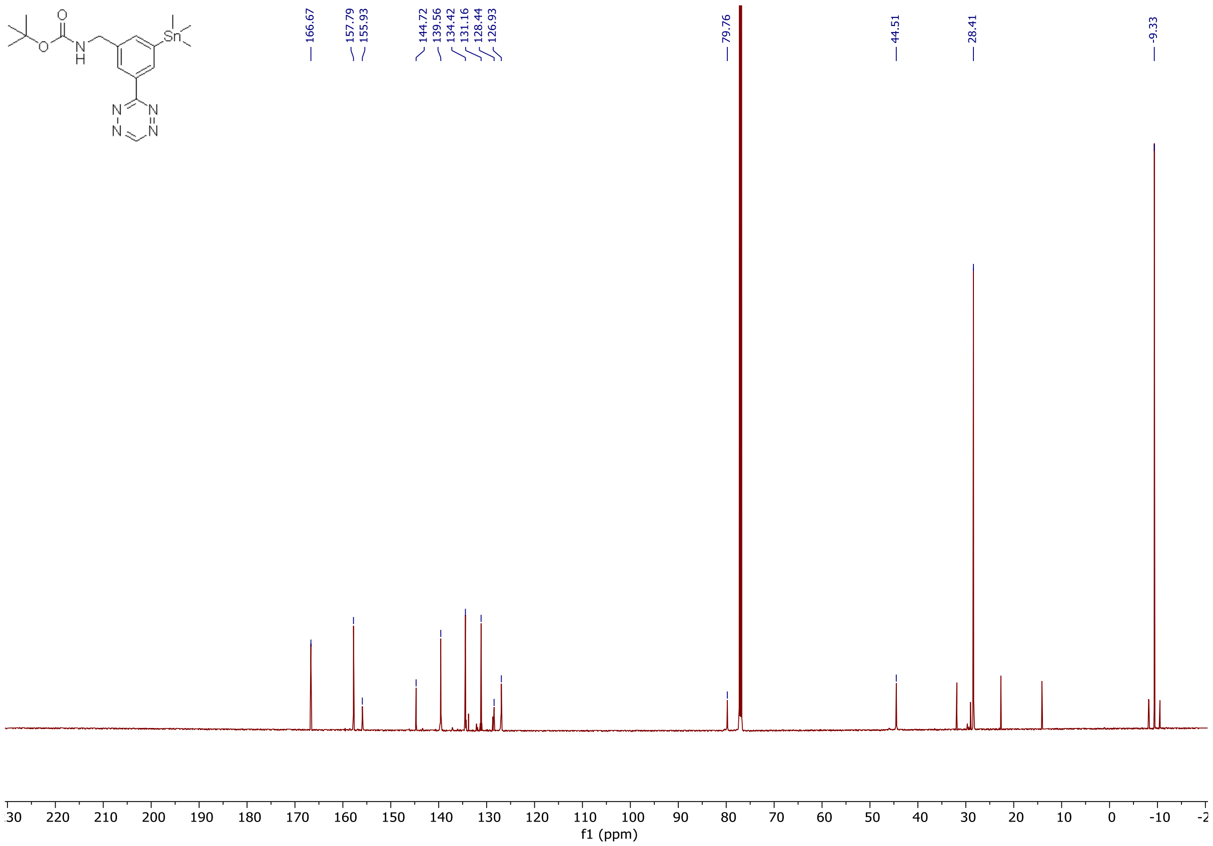

3-Iodo-5-((methylamino)methyl)benzonitrile hydrochloride

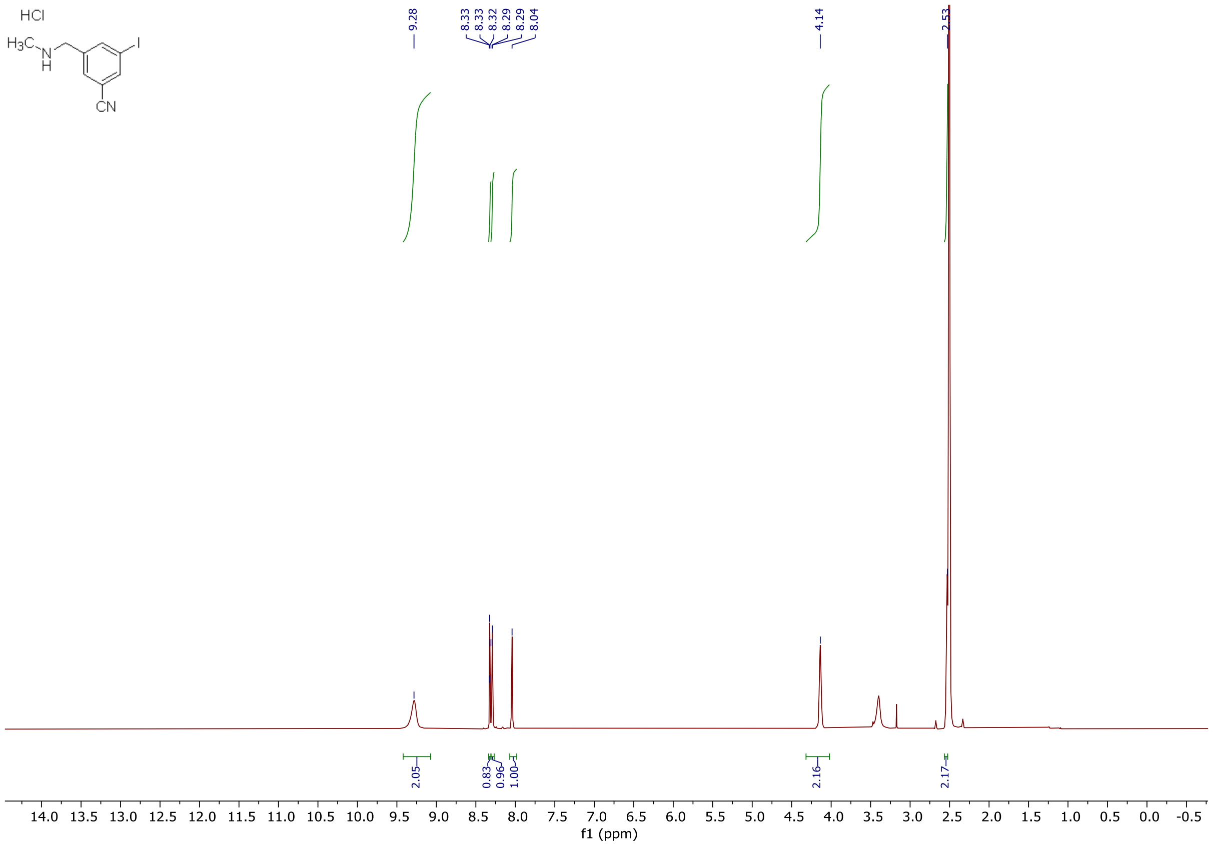

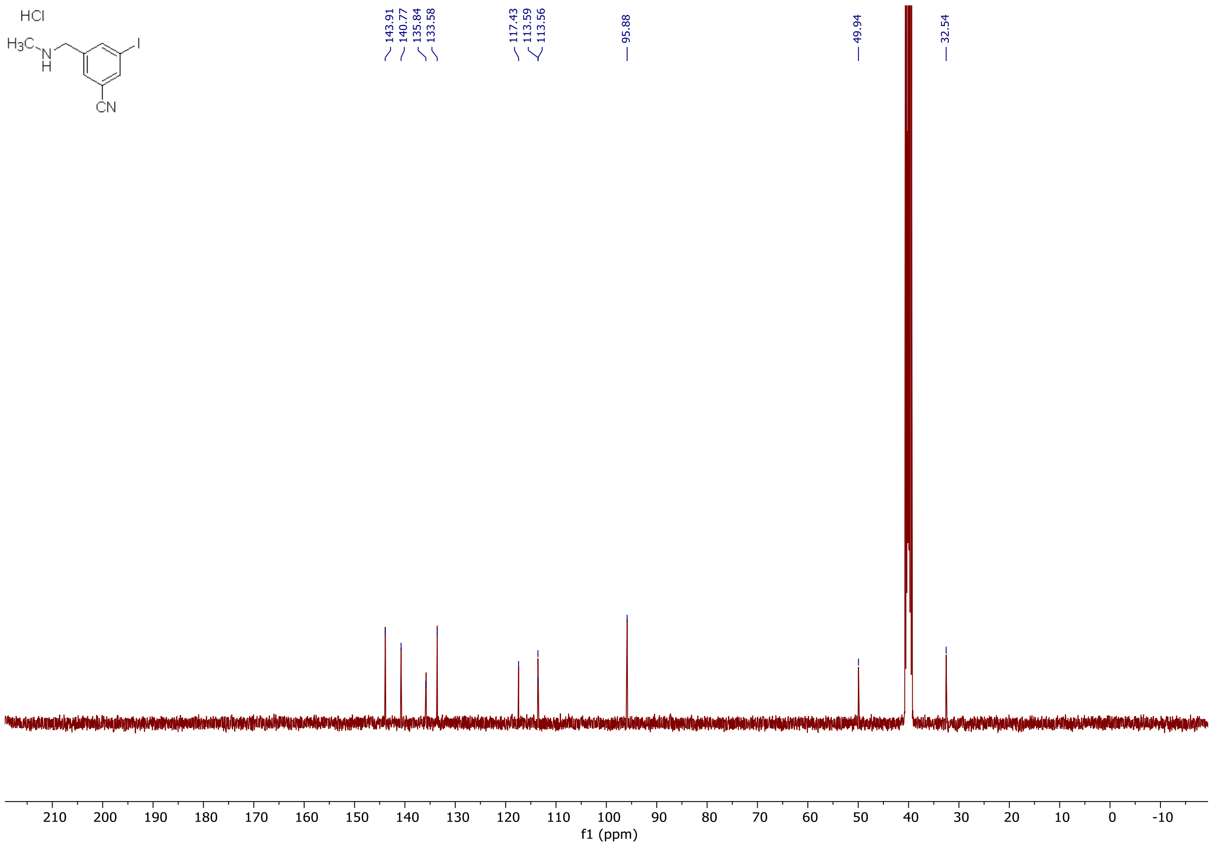

***Tert*-butyl 3-cyano-5-iodobenzyl(methyl)carbamate**

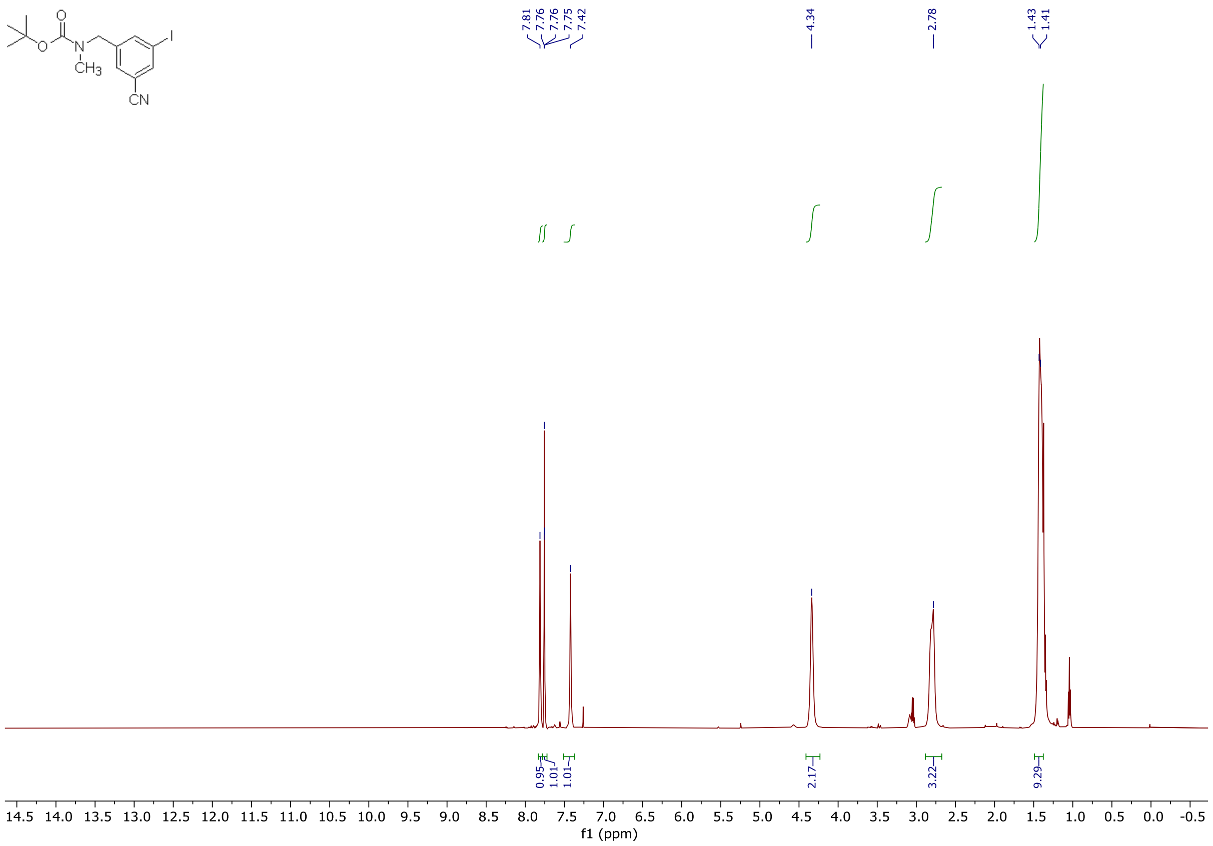

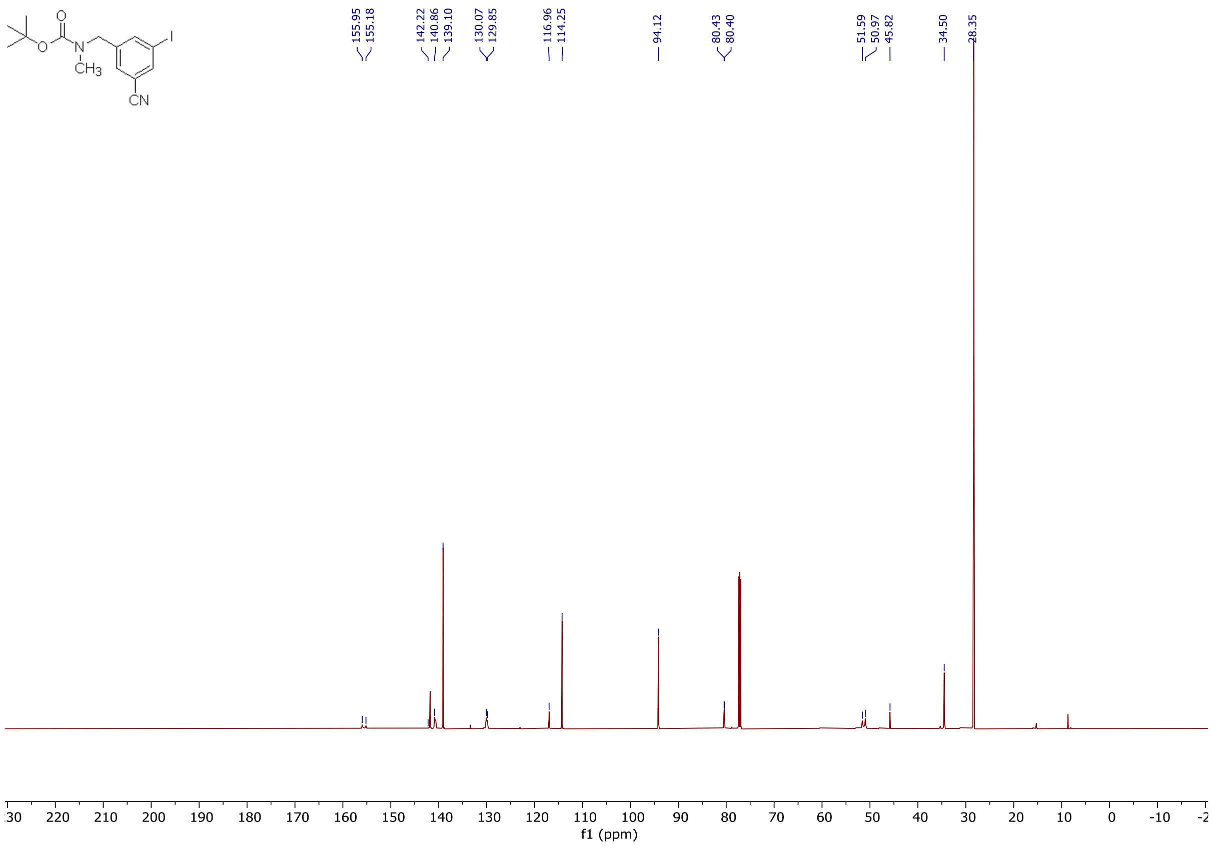

***Tert*-butyl 3-iodo-5-(1,2,4,5-tetrazin-3-yl)benzyl(methyl)carbamate**

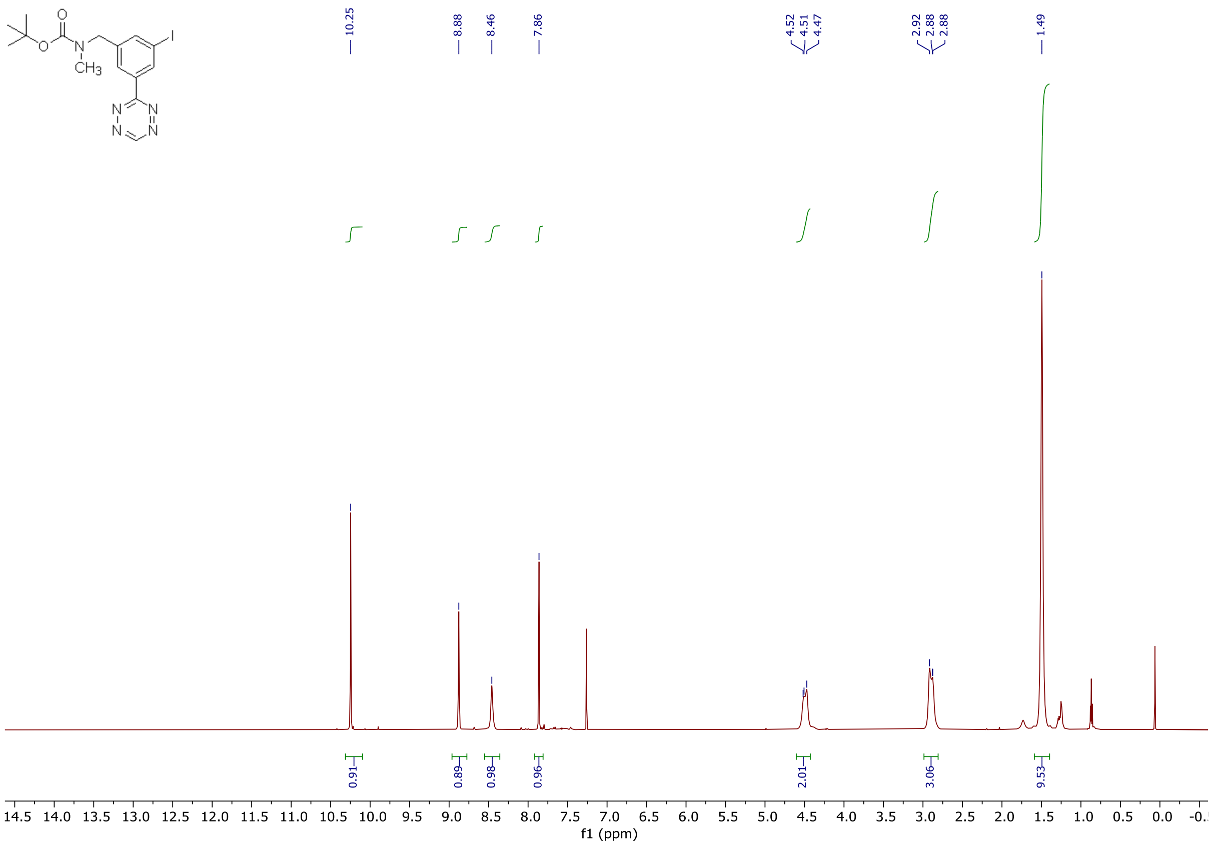

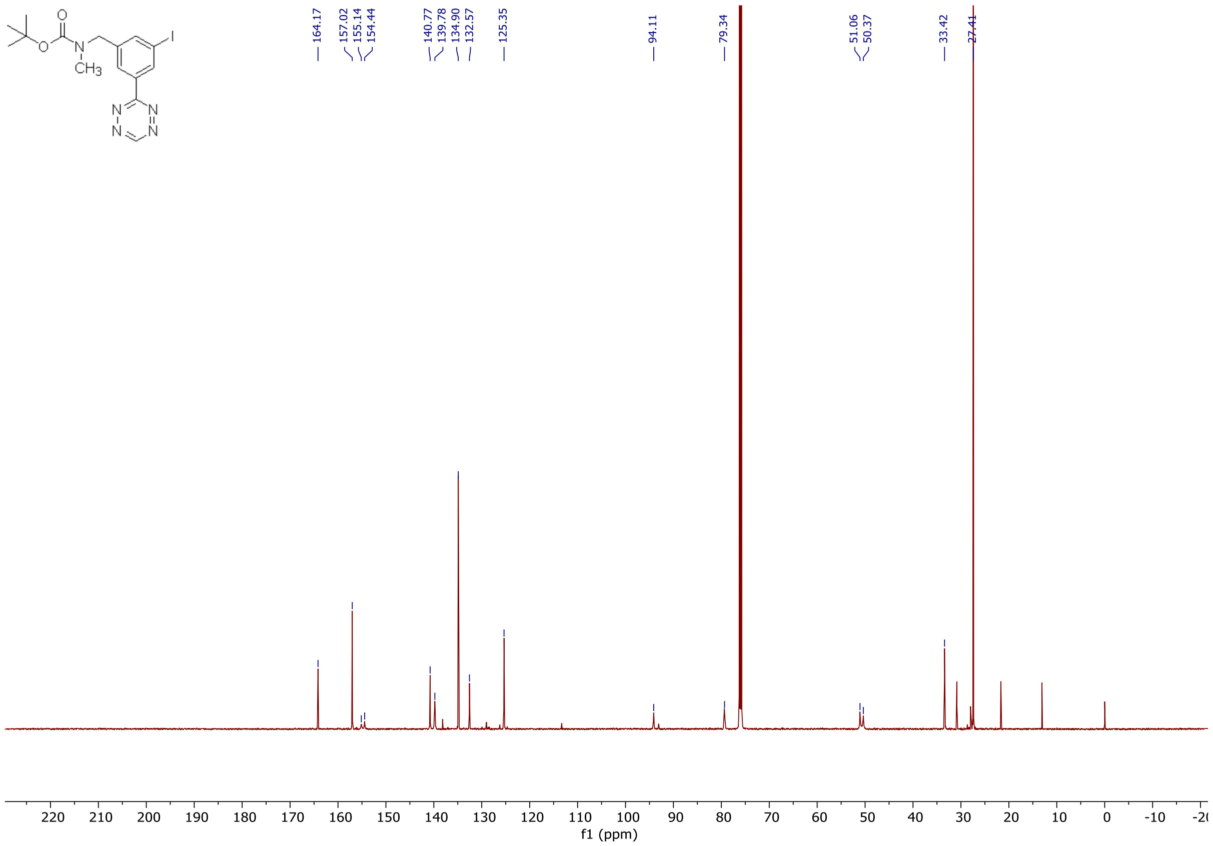

Tert-butyl 3-(1,2,4,5-tetrazin-3-yl)-5-(trimethylstannyl)benzyl(methyl)carbamate

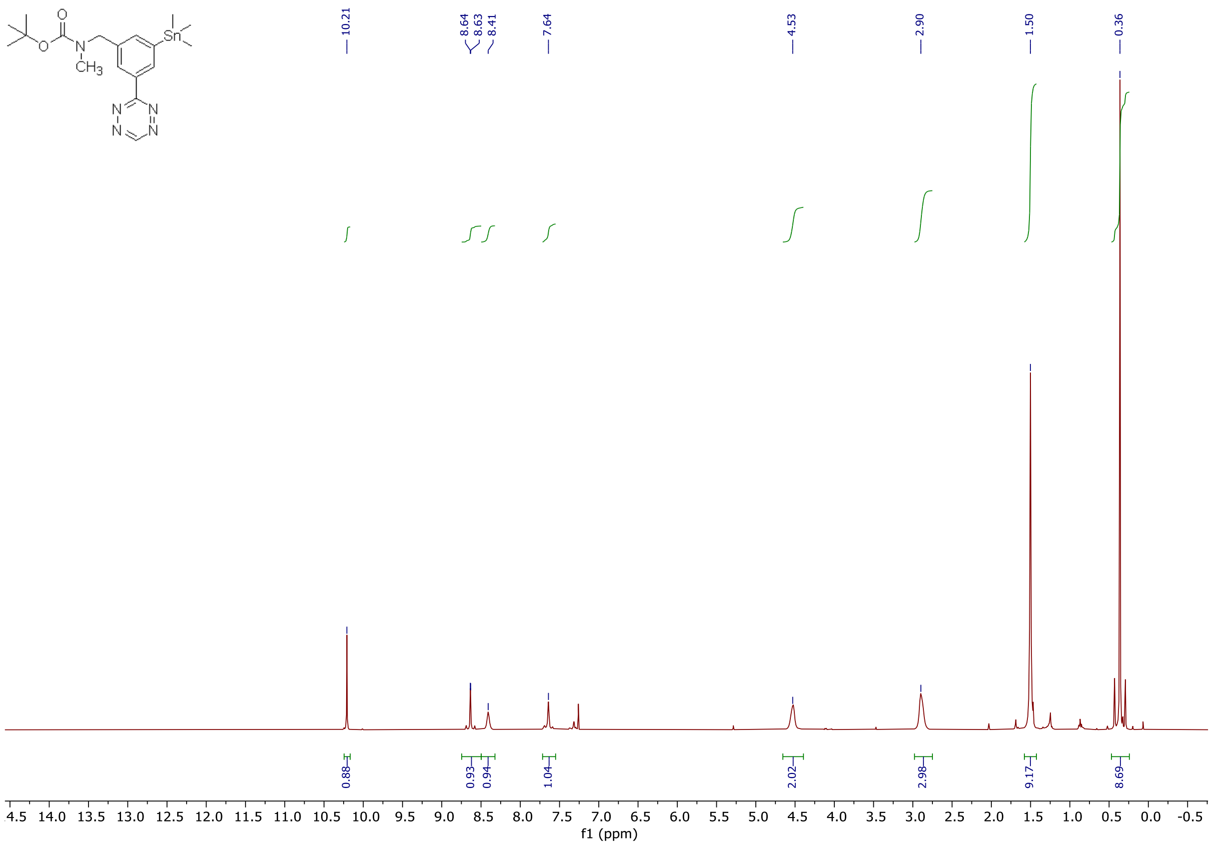

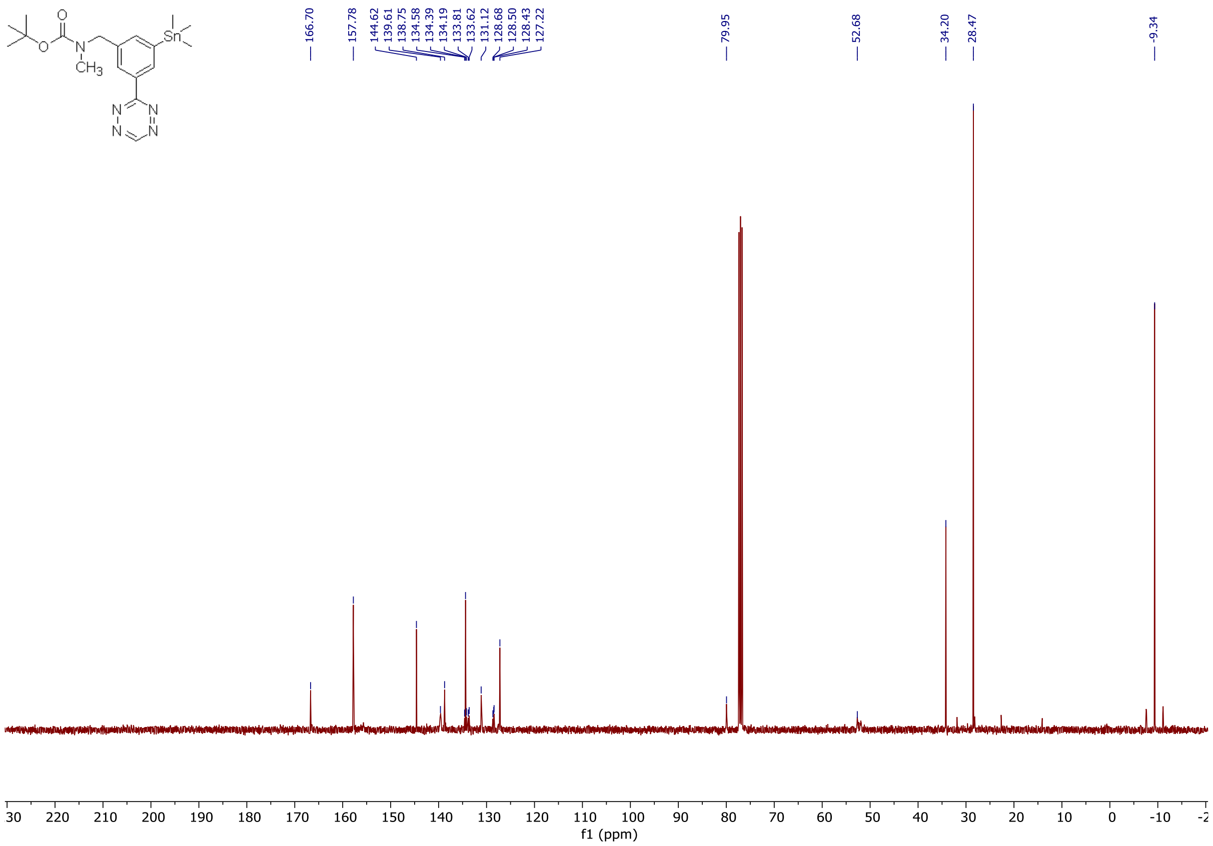

3-((Ethylamino)methyl)-5-iodobenzonitrile hydrochloride

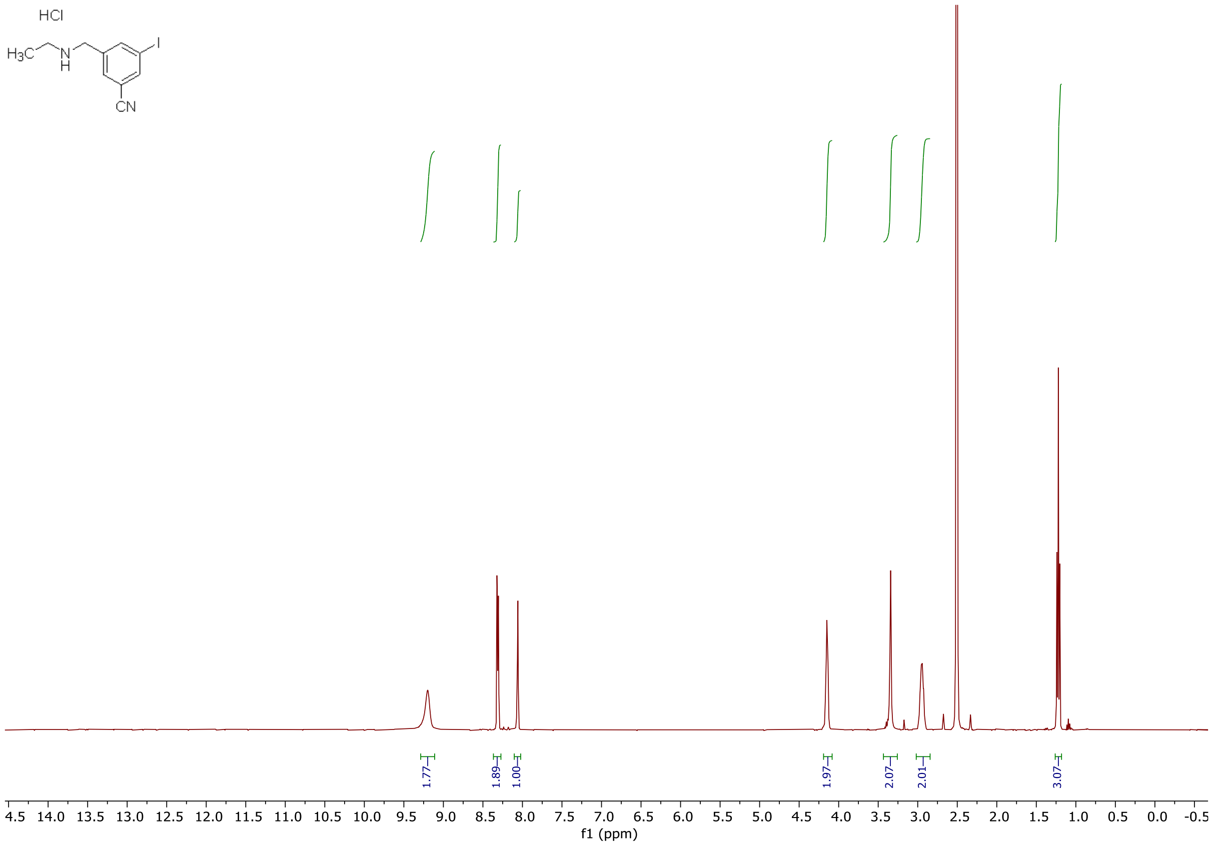

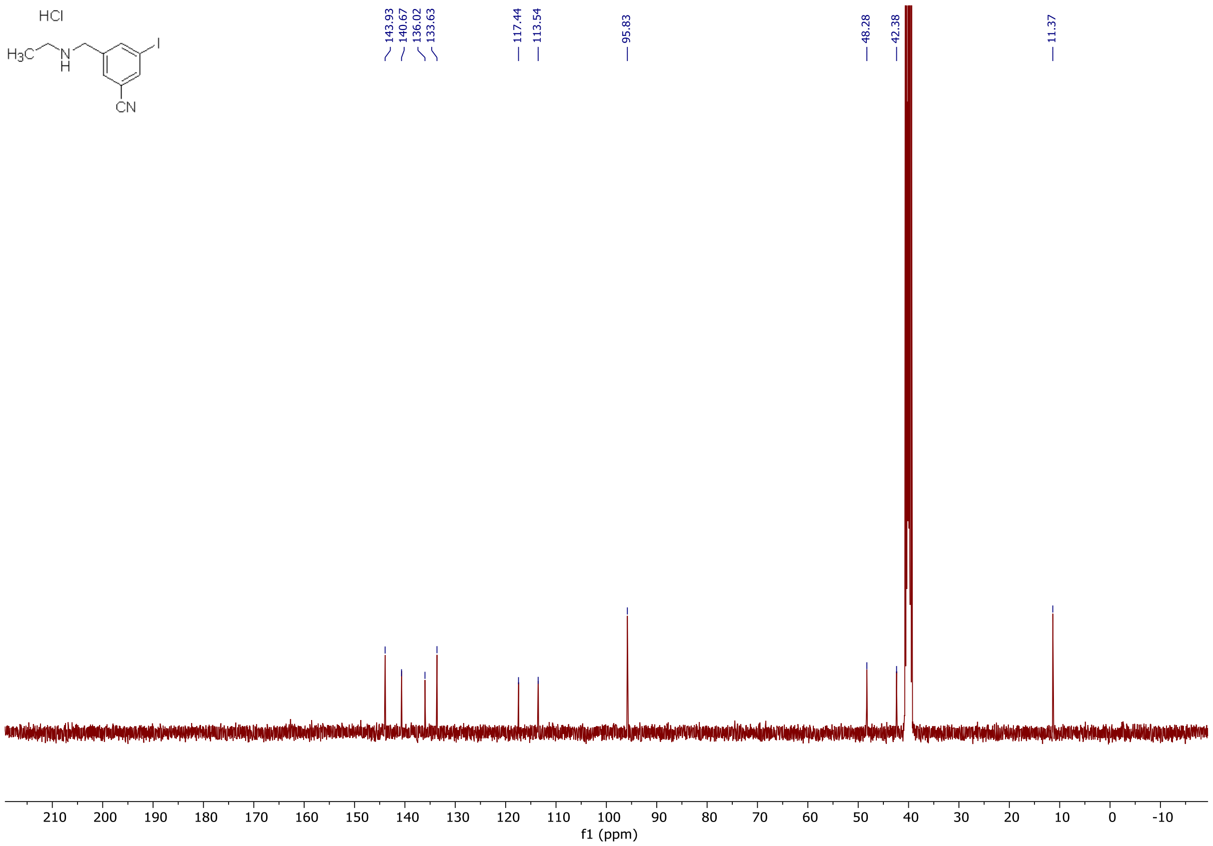

***Tert*-butyl 3-cyano-5-iodobenzyl(ethyl)carbamate**

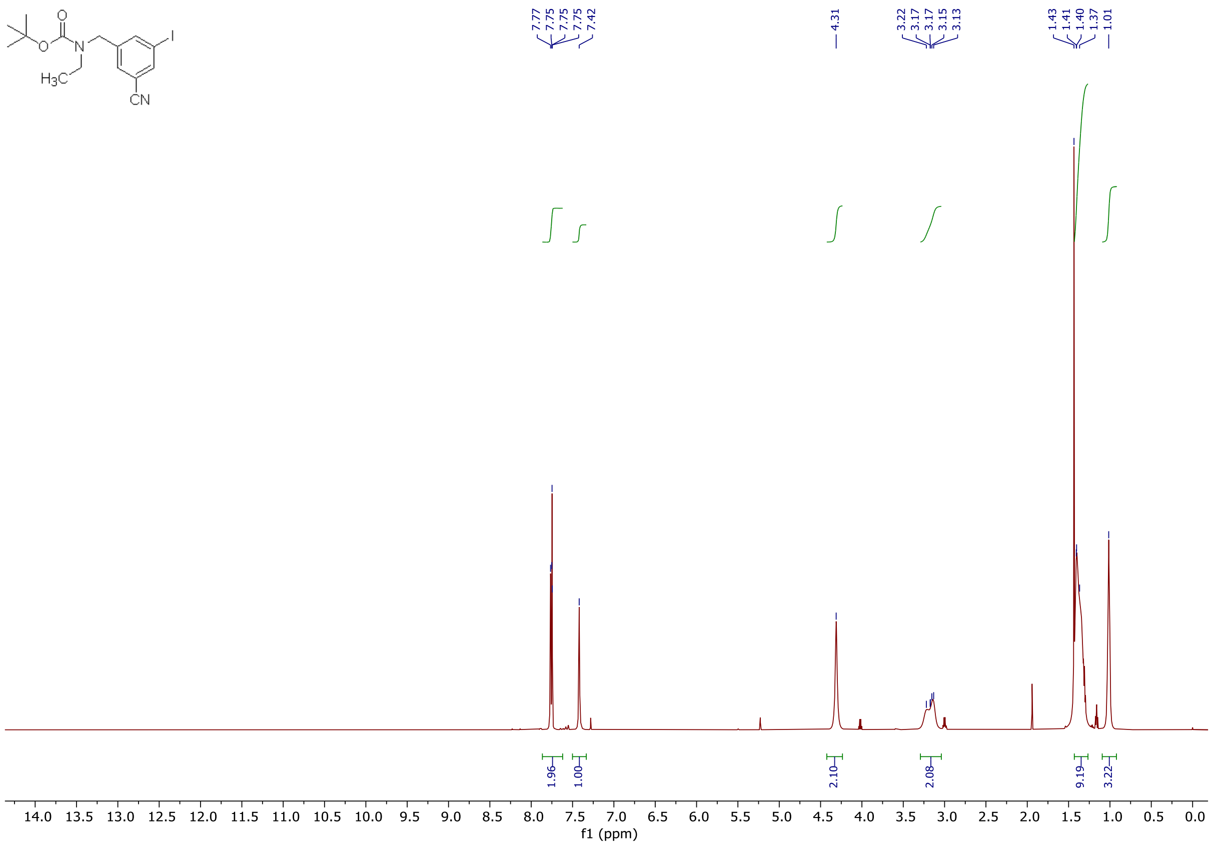

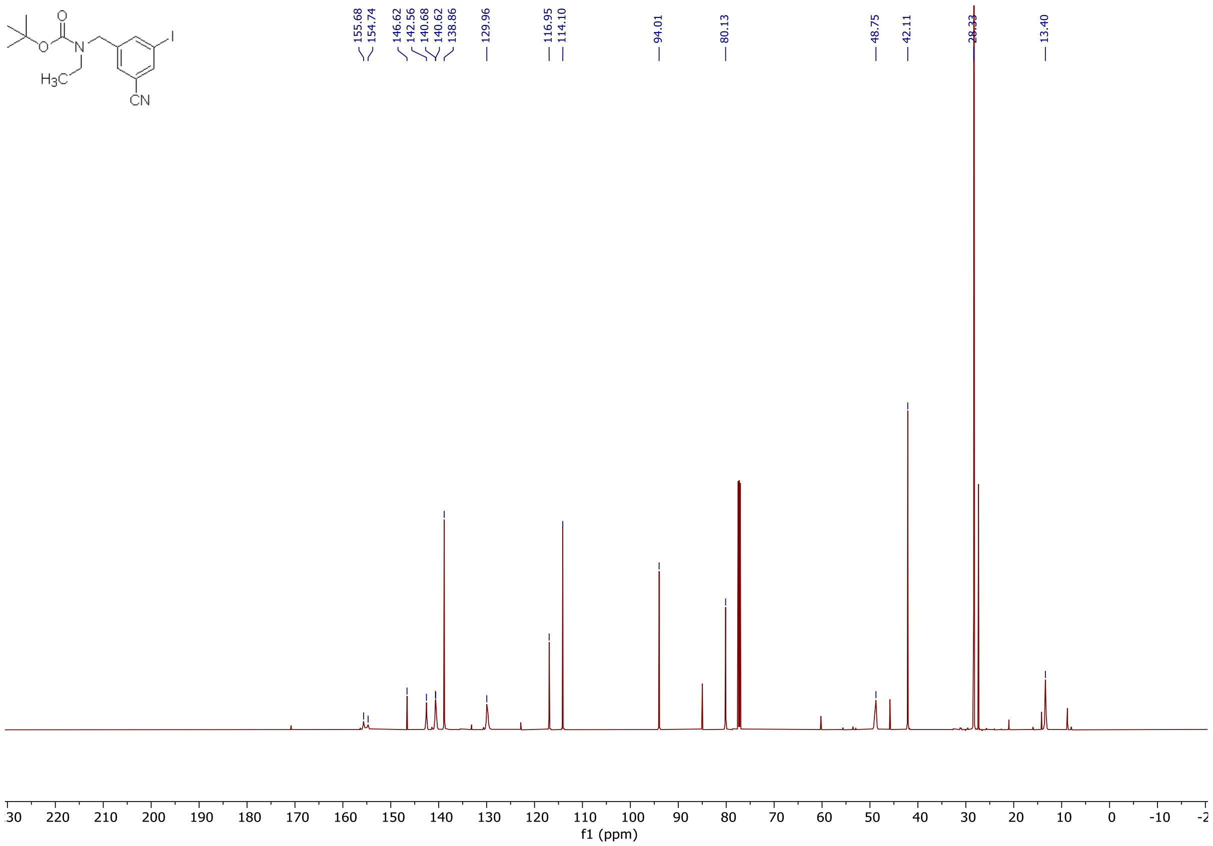

***Tert*-butyl 3-iodo-5-(1,2,4,5-tetrazin-3-yl)benzyl(ethyl)carbamate**

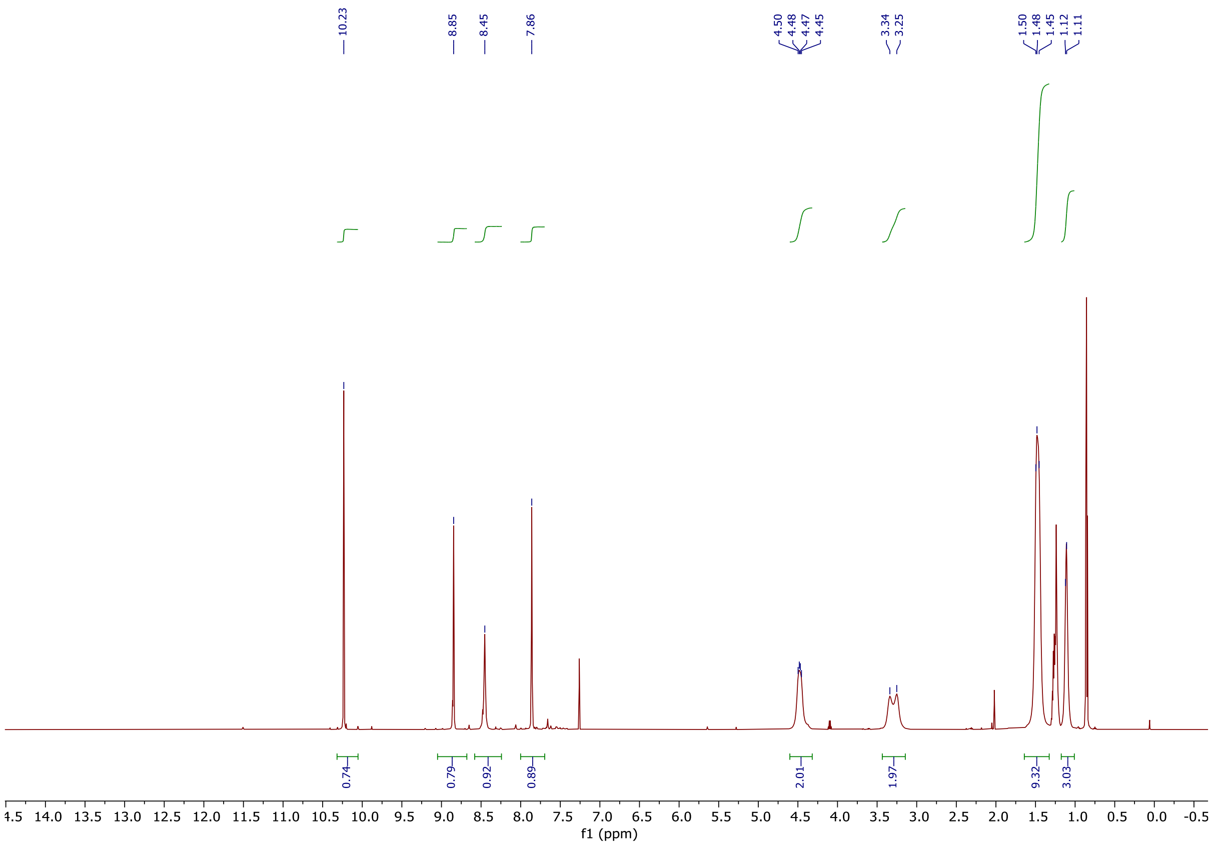

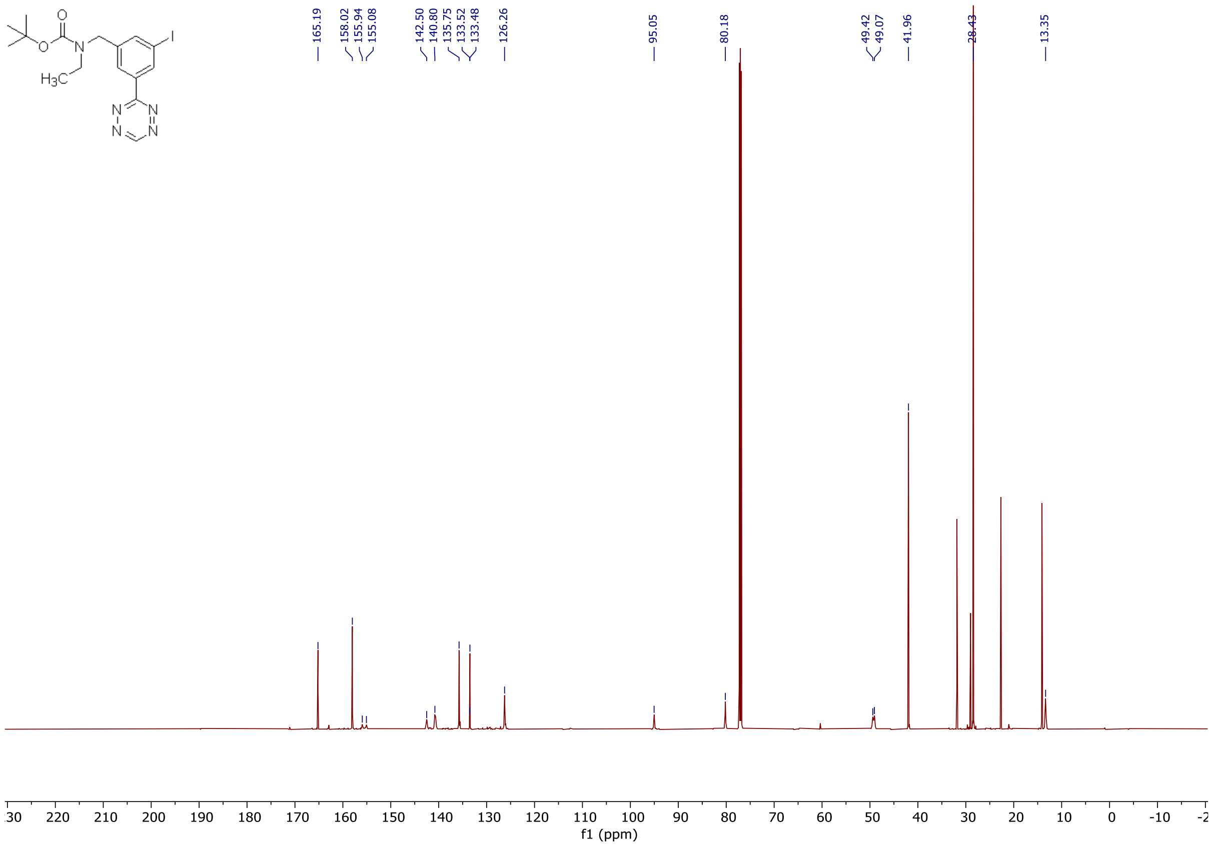

***Ter*t-butyl 3-(1,2,4,5-tetrazin-3-yl)-5-(trimethylstannyl)benzyl(ethyl)carbamate**

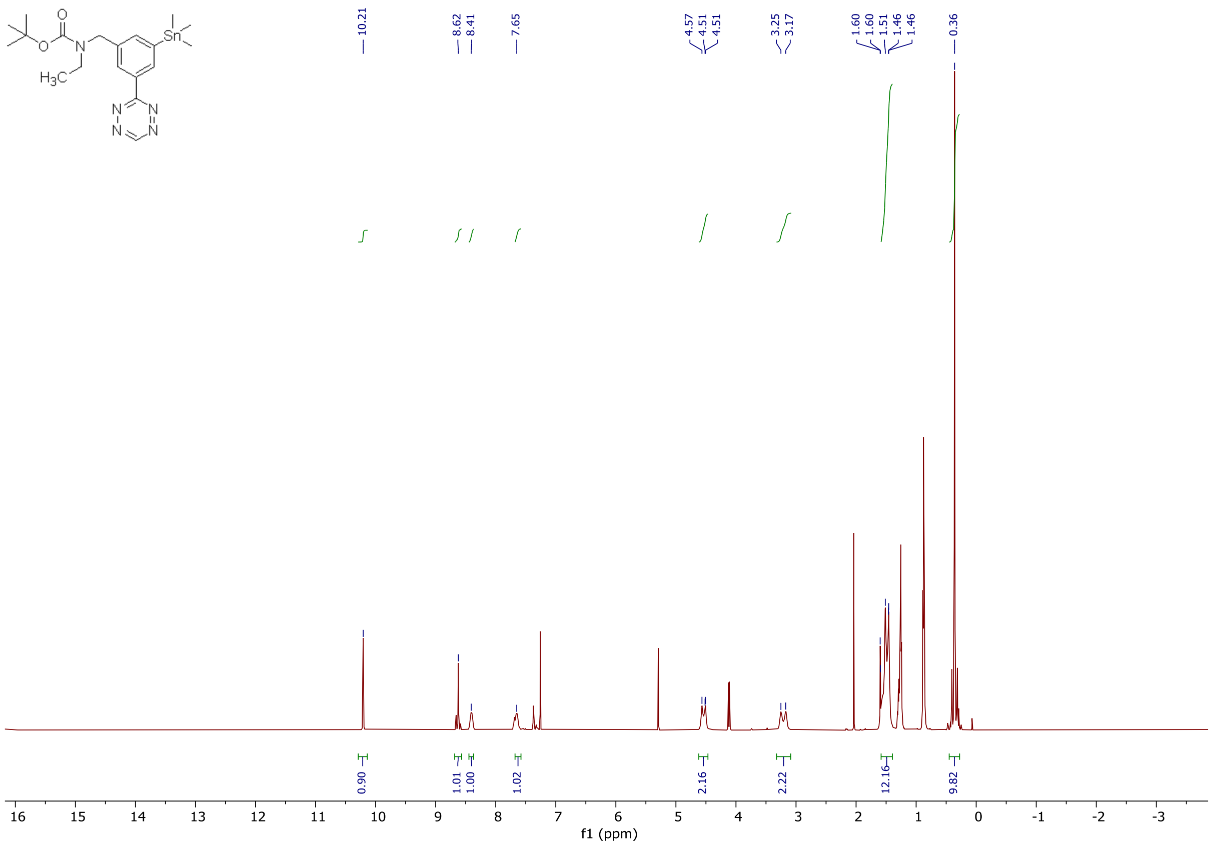

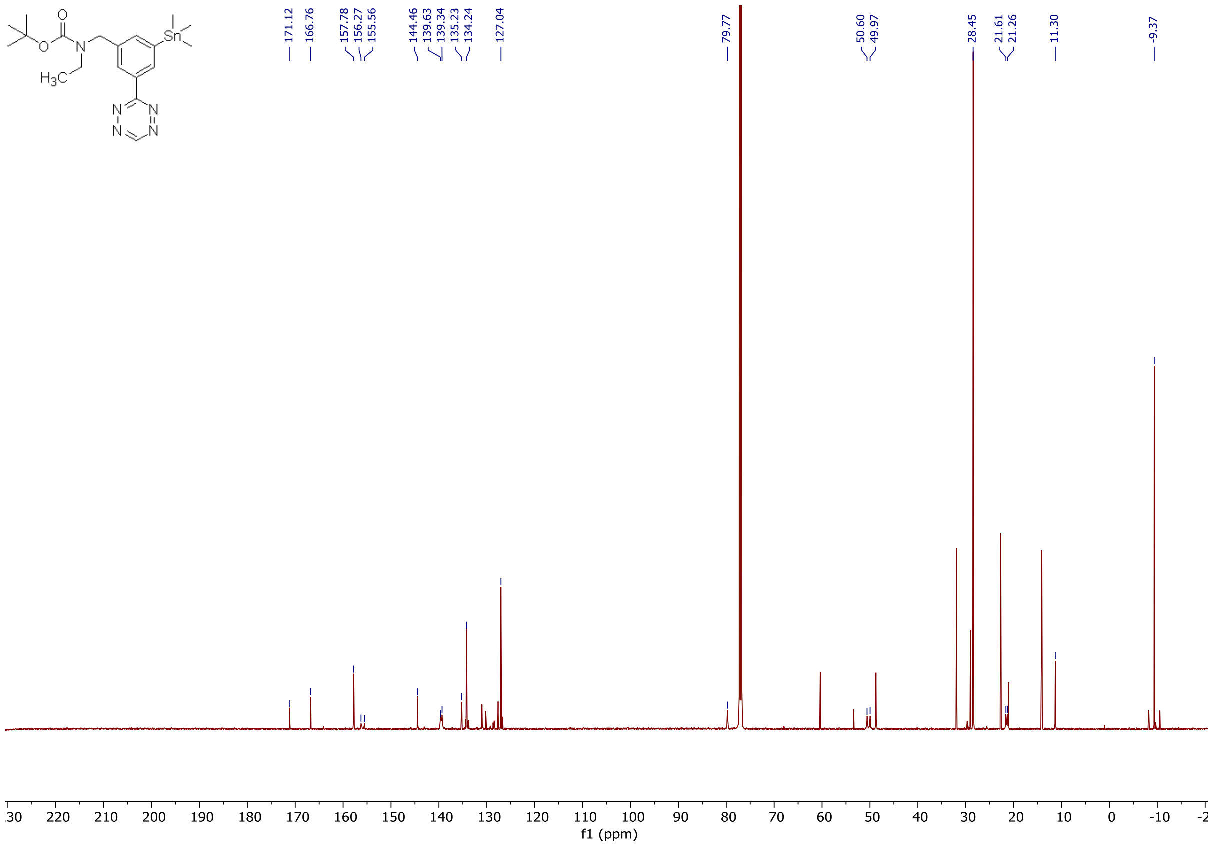

3-((Propylamino)methyl)-5-iodobenzonitrile hydrochloride

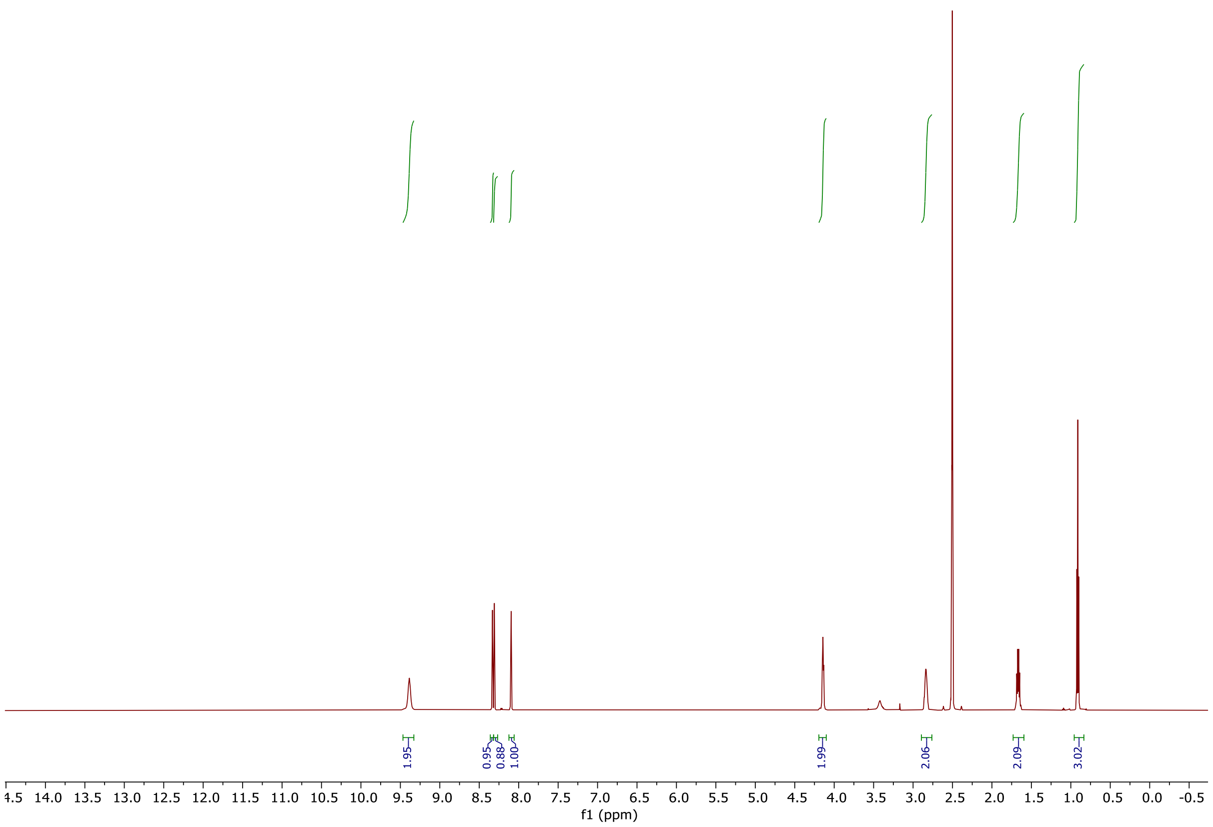

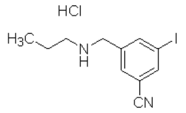

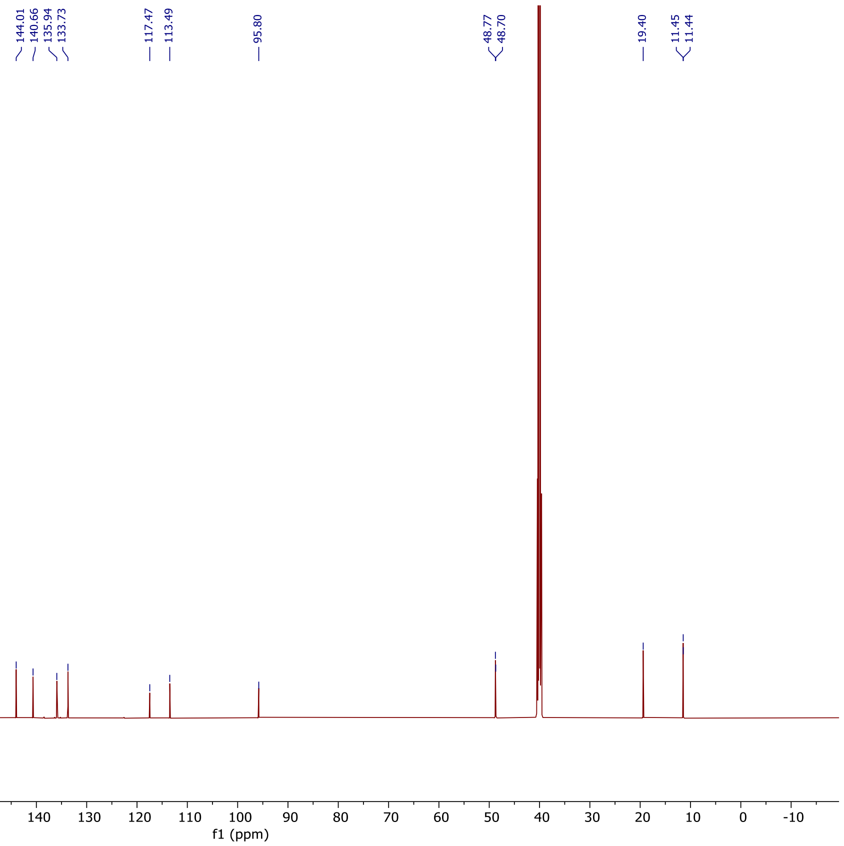

***Tert*-butyl 3-cyano-5-iodobenzyl(propyl)carbamate**

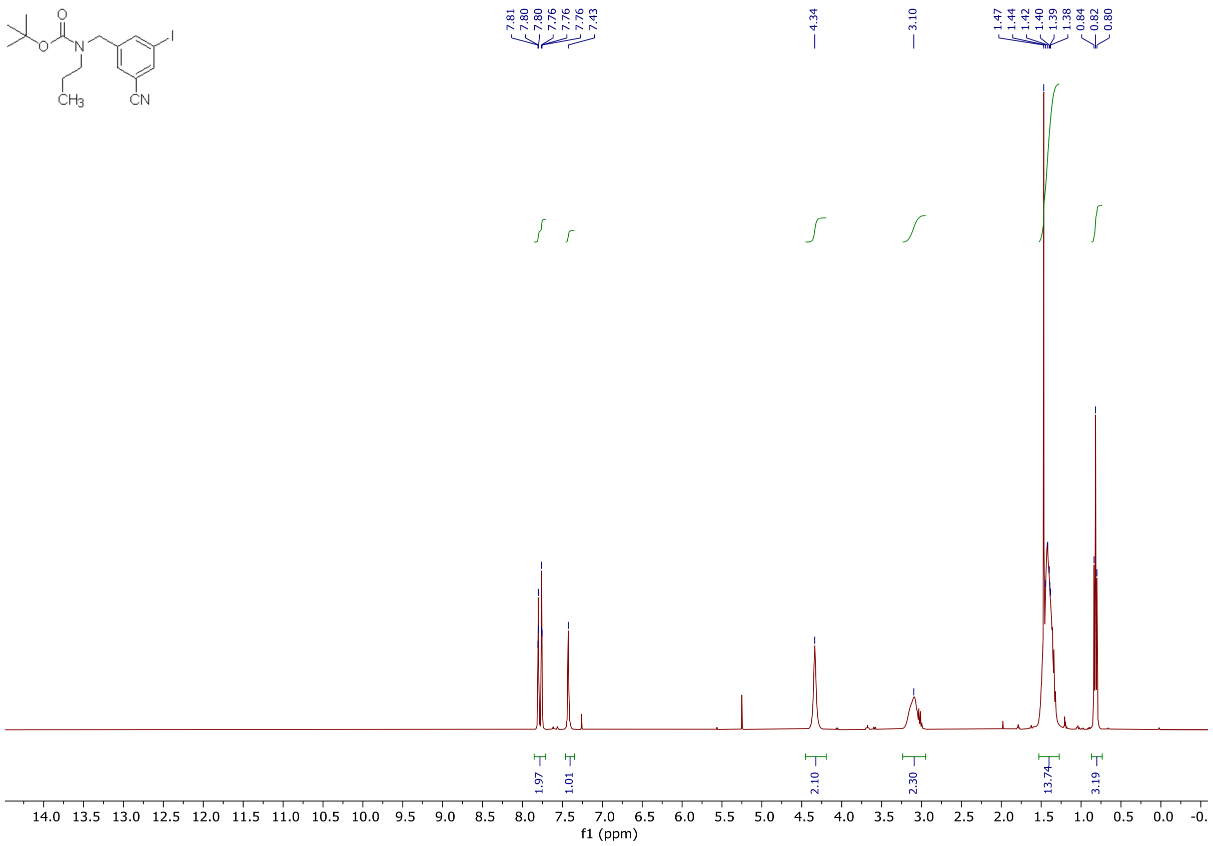

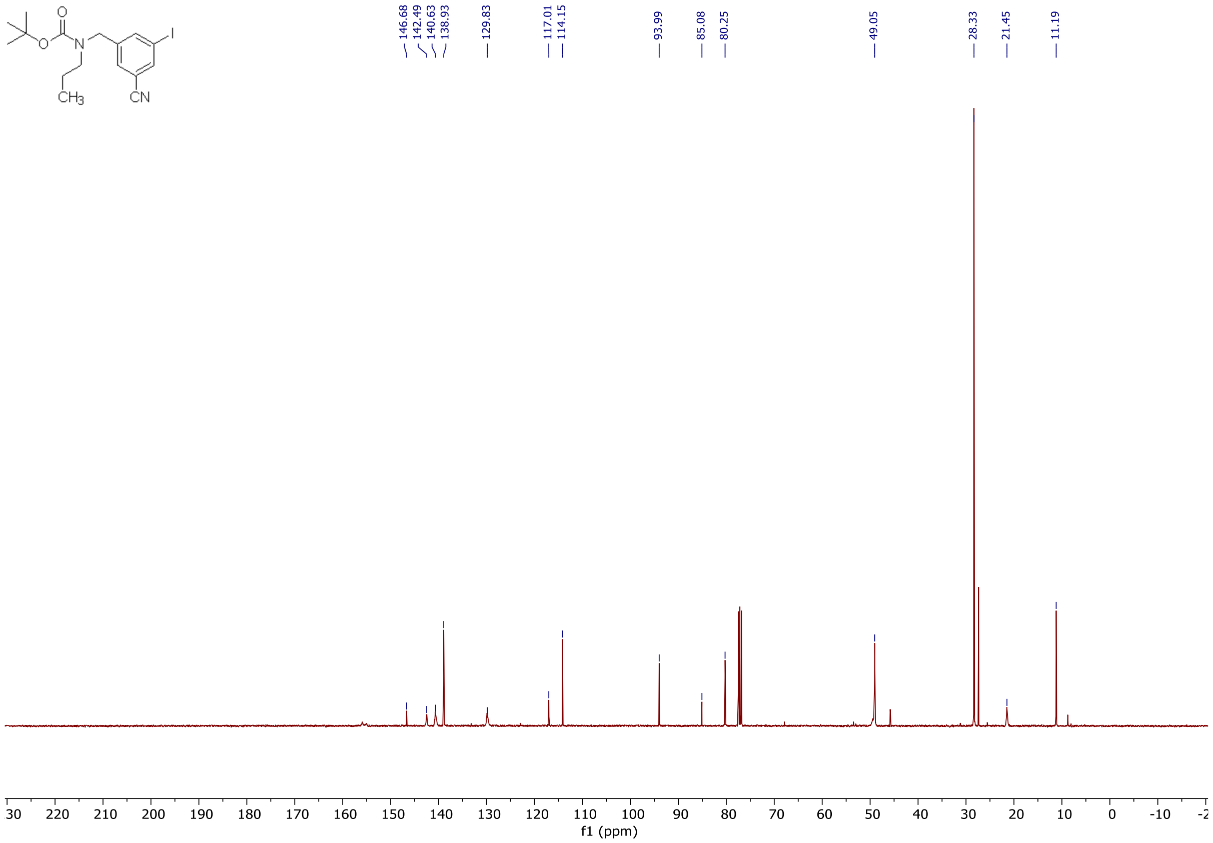

Tert-butyl 3-iodo-5-(1,2,4,5-tetrazin-3-yl)benzyl(propyl)carbamate

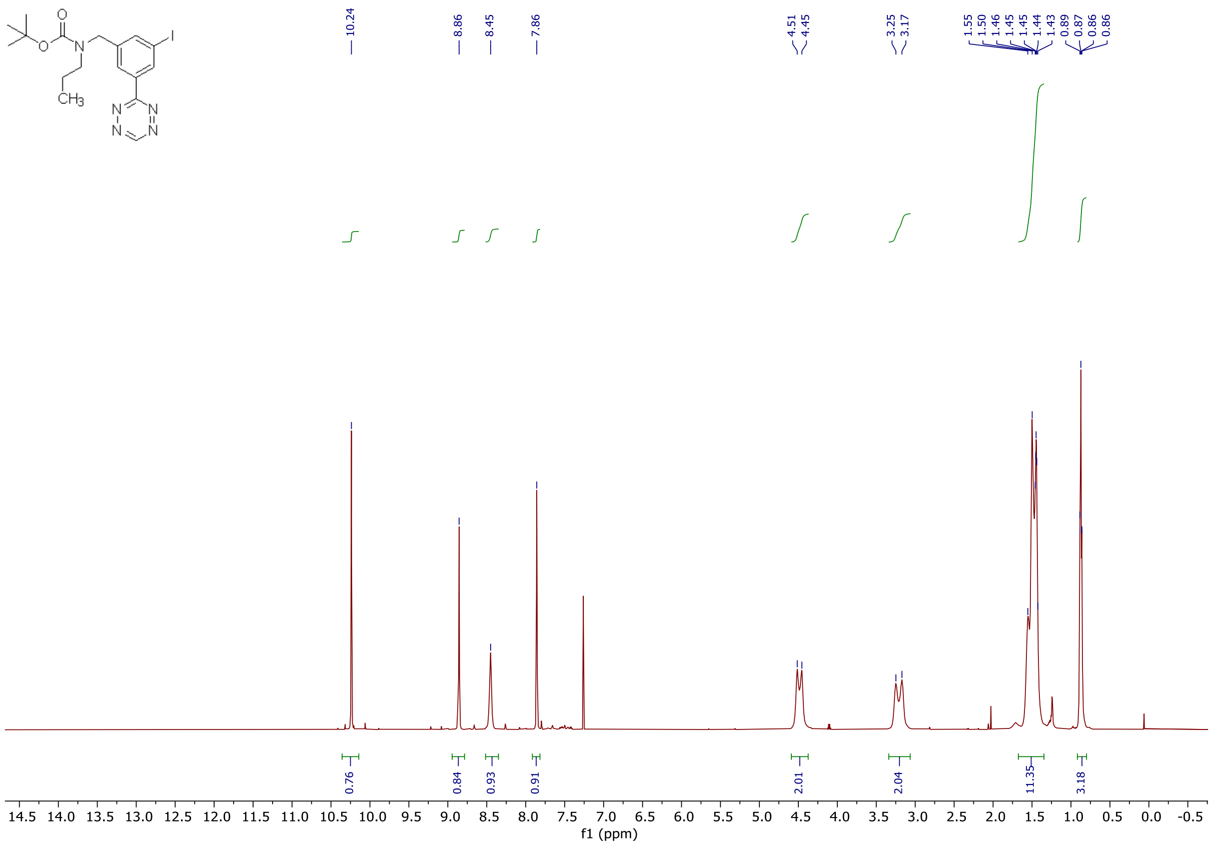

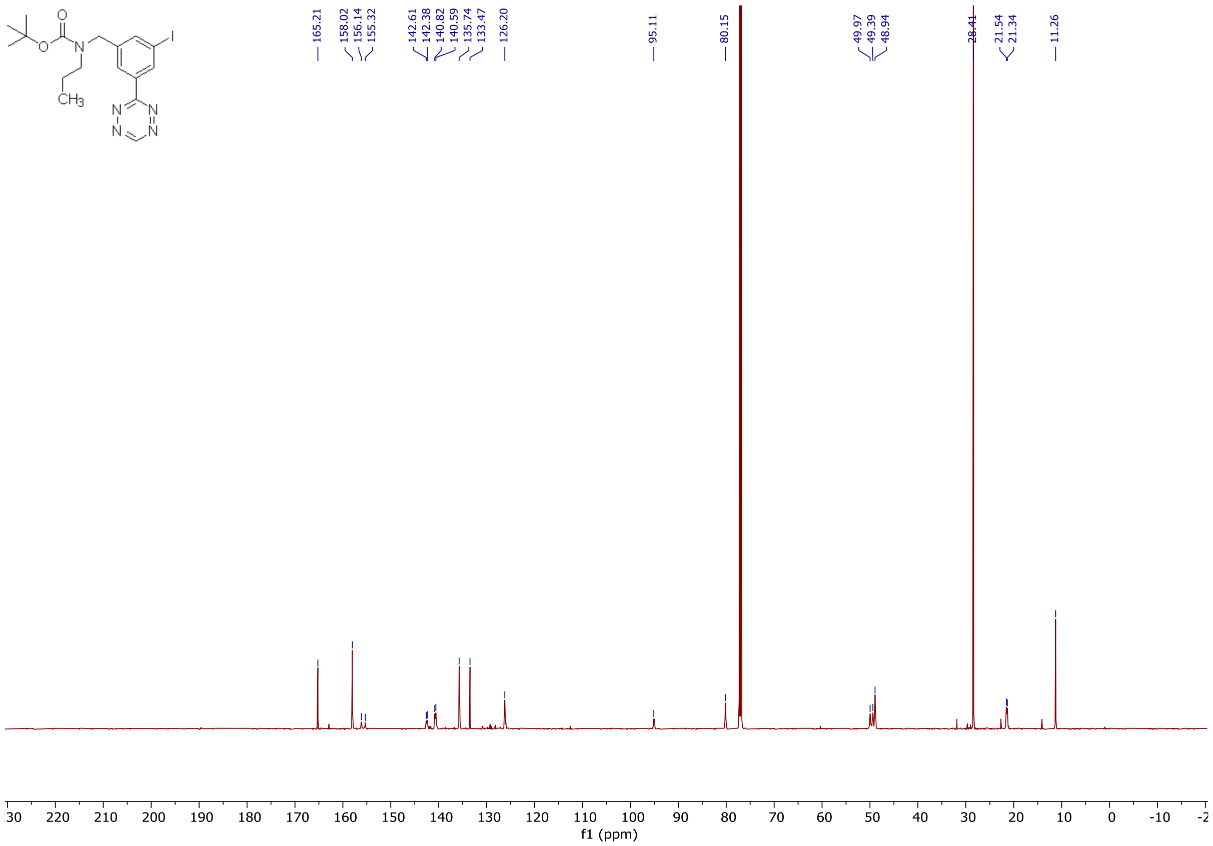

***Tert*-butyl 3-(1,2,4,5-tetrazin-3-yl)-5-(trimethylstannyl)benzyl(ethyl)carbamate**

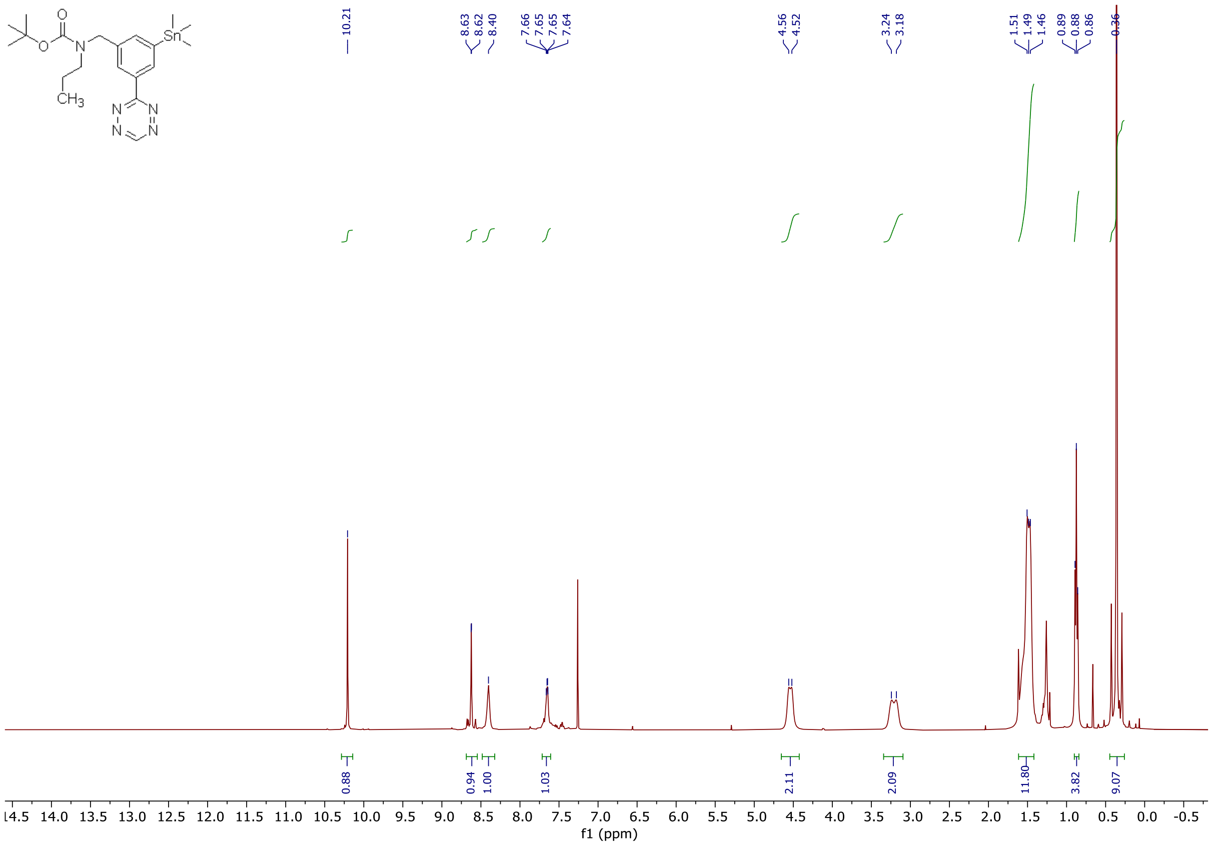
10.21
8.63
8.62
8.40
7.66
7.65
7.65
7.64
4.56
4.52
3.24
3.18
1.51
1.49
1.46
0.89
0.88
0.86
0.36
0.88
0.94
1.00
1.03
2.11
2.09
11.80
3.82
9.07
f1 (ppm)

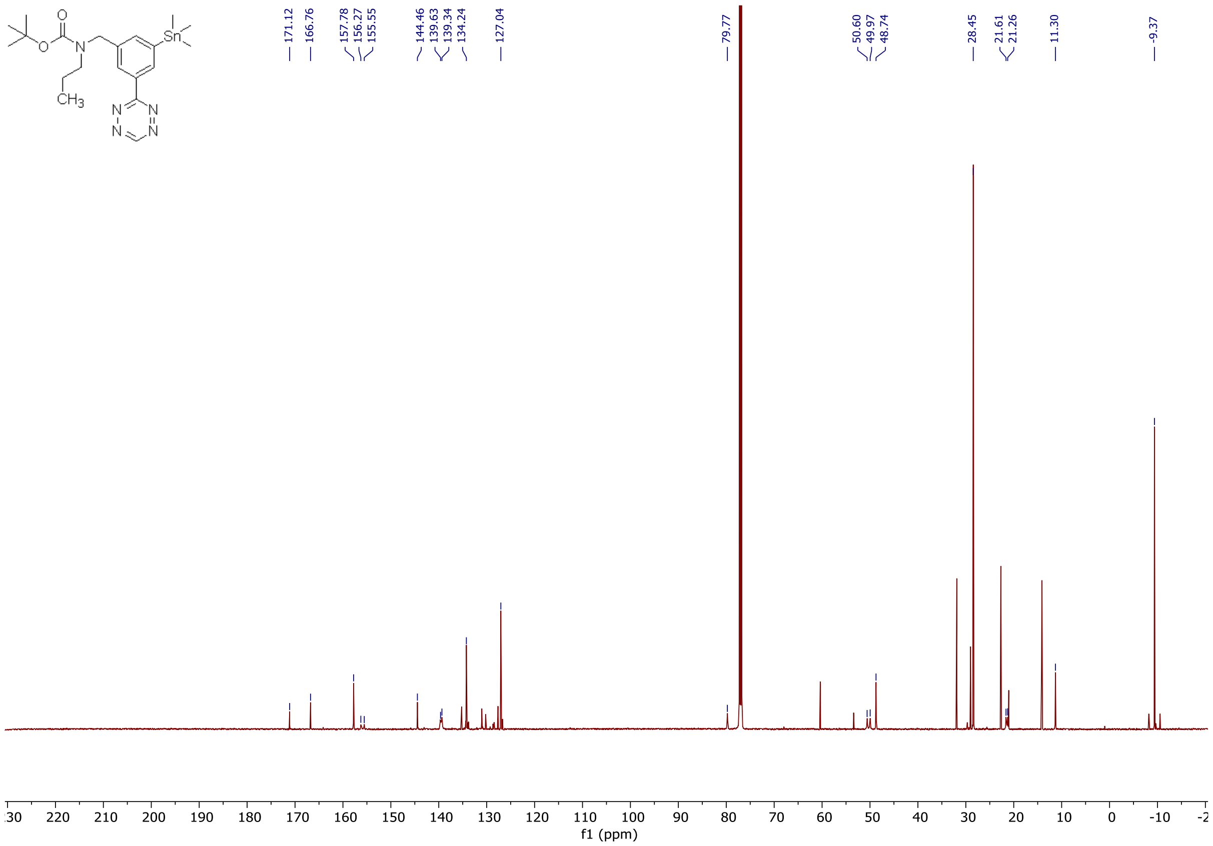
171.12
166.76
157.78
156.27
155.55
144.46
139.63
139.34
134.24
127.04
79.77
50.60
49.97
48.74
28.45
21.61
21.26
11.30
-9.37
f1 (ppm)

References

1. P. T. Meyer, D. Elmenhorst, K. Zilles and A. Bauer, *Synapse*, 2005, **55**, 212-223.
2. E. T. L'Estrade, M. Erlandsson, F. G. Edgar, T. Ohlsson, G. M. Knudsen and M. M. Herth, *Neuropharmacology*, 2020, **172**, 107830.
3. R. A. Koeppe, K. A. Frey, S. E. Snyder, P. Meyer, M. R. Kilbourn and D. E. Kuhl, *J Cereb Blood Flow Metab*, 1999, **19**, 1150-1163.
4. D. F. Wong, A. Gjedde and H. N. Wagner, Jr., *J Cereb Blood Flow Metab*, 1986, **6**, 137-146.
5. R. N. Waterhouse, *Mol Imaging Biol*, 2003, **5**, 376-389.
6. Y. Qu, F. X. Sauvage, G. Clavier, F. Miomandre and P. Audebert, *Angew Chem Int Ed Engl*, 2018, **57**, 12057-12061.
7. R. Garcia-Vazquez, U. M. Battisti, J. T. Jorgensen, V. Shalgunov, L. Hvass, D. L. Stares, I. N. Petersen, F. Crestey, A. Loffler, D. Svatunek, J. L. Kristensen, H. Mikula, A. Kjaer and M. M. Herth, *Chem Sci*, 2021, **12**, 11668-11675.
8. U. M. Battisti, R. Garcia-Vazquez, D. Svatunek, B. Herrmann, A. Loffler, H. Mikula and M. M. Herth, *Bioconjug Chem*, 2022, DOI: 10.1021/acs.bioconjchem.2c00042.
9. B. J. Levandowski, D. Svatunek, B. Sohr, H. Mikula and K. N. Houk, *J Am Chem Soc*, 2019, **141**, 2224-2227.
10. M. M. Herth, S. Ametamey, D. Antuganov, A. Bauman, M. Berndt, A. F. Brooks, G. Bormans, Y. S. Choe, N. Gillings, U. O. Häfeli, M. L. James, K. Kopka, V. Kramer, R. Krasikova, J. Madsen, L. Mu, B. Neumaier, M. Piel, F. Rösch, T. Ross, R. Schibli, P. J. H. Scott, V. Shalgunov, N. Vasdev, W. Wadsak and B. M. Zeglis, *Nucl Med Biol*, 2021, **93**, 19-21.
11. D. Sehlin, X. T. Fang, L. Cato, G. Antoni, L. Lannfelt and S. Syvänen, *Nature Communications*, 2016, **7**, 10759.
12. N. R. Raval, A. Johansen, L. L. Donovan, N. F. Ros, B. Ozenne, H. D. Hansen and G. M. Knudsen, *International journal of molecular sciences*, 2021, **22**, 835.
13. N. Griem-Krey, A. B. Klein, M. Herth and P. Wellendorph, *JoVE (Journal of Visualized Experiments)*, 2019, e58879.
14. S. H. Keller, E. N. L'Estrade, B. Dall, M. Palner and M. Herth, *Ieee Nucl Sci Conf R*, 2016, DOI: 10.1109/NSSMIC.2016.8069467.
15. A. Casado-Sainz, F. Gudmundsen, S. L. Baerentzen, D. Lange, A. Ringsted, I. Martinez-Tajada, S. Medina, H. Lee, C. Svarer, S. H. Keller, M. Schain, C. Kjaerby, P. M. Fisher, P. Cumming and M. Palner, *BioRxiv*, 2021, DOI: 10.1101/2021.02.11.430770.
16. V. Shalgunov, M. Xiong, E. T. L'Estrade, N. R. Raval, I. V. Andersen, F. G. Edgar, N. R. Speth, S. L. Baerentzen, H. D. Hansen, L. L. Donovan, A. Nasser, S. T. Peitersen, A. Kjaer, G. M. Knudsen, S. Syvanen, M. Palner and M. M. Herth, *EJNMMI Res*, 2020, **10**, 124.
17. S. H. Keller, C. Svarer and M. Sibomana, *IEEE Trans Med Imaging*, 2013, **32**, 1611-1621.
18. E. J. L. Stéen, J. T. Jørgensen, K. Johann, K. Nørregaard, B. Sohr, D. Svatunek, A. Birke, V. Shalgunov, P. E. Edem, R. Rossin, C. Seidl, F. Schmid, M. S. Robillard, J. L. Kristensen, H. Mikula, M. Barz, A. Kjær and M. M. Herth, *ACS Nano*, 2020, **14**, 568-584.
